# Analysis of SARS-CoV-2 known and novel subgenomic mRNAs in cell culture, animal model, and clinical samples using LeTRS, a bioinformatic tool to identify unique sequence identifiers

**DOI:** 10.1093/gigascience/giac045

**Published:** 2022-05-26

**Authors:** Xiaofeng Dong, Rebekah Penrice-Randal, Hannah Goldswain, Tessa Prince, Nadine Randle, I'ah Donovan-Banfield, Francisco J Salguero, Julia Tree, Ecaterina Vamos, Charlotte Nelson, Jordan Clark, Yan Ryan, James P Stewart, Malcolm G Semple, J Kenneth Baillie, Peter J M Openshaw, Lance Turtle, David A Matthews, Miles W Carroll, Alistair C Darby, Julian A Hiscox

**Affiliations:** Institute of Infection, Veterinary and Ecological Sciences, University of Liverpool, Liverpool, L3 5RF, UK; Institute of Infection, Veterinary and Ecological Sciences, University of Liverpool, Liverpool, L3 5RF, UK; Institute of Infection, Veterinary and Ecological Sciences, University of Liverpool, Liverpool, L3 5RF, UK; Institute of Infection, Veterinary and Ecological Sciences, University of Liverpool, Liverpool, L3 5RF, UK; Institute of Infection, Veterinary and Ecological Sciences, University of Liverpool, Liverpool, L3 5RF, UK; Institute of Infection, Veterinary and Ecological Sciences, University of Liverpool, Liverpool, L3 5RF, UK; NIHR Health Protection Research Unit in Emerging and Zoonotic Infections, Liverpool, Liverpool, L69 7BE, UK; UK-Health Security Agency, Salisbury, SP4 0JG, UK; UK-Health Security Agency, Salisbury, SP4 0JG, UK; Institute of Infection, Veterinary and Ecological Sciences, University of Liverpool, Liverpool, L3 5RF, UK; Institute of Infection, Veterinary and Ecological Sciences, University of Liverpool, Liverpool, L3 5RF, UK; Institute of Infection, Veterinary and Ecological Sciences, University of Liverpool, Liverpool, L3 5RF, UK; Institute of Infection, Veterinary and Ecological Sciences, University of Liverpool, Liverpool, L3 5RF, UK; Institute of Infection, Veterinary and Ecological Sciences, University of Liverpool, Liverpool, L3 5RF, UK; Institute of Infection, Veterinary and Ecological Sciences, University of Liverpool, Liverpool, L3 5RF, UK; NIHR Health Protection Research Unit in Emerging and Zoonotic Infections, Liverpool, Liverpool, L69 7BE, UK; The Roslin Institute, University of Edinburgh, Edinburgh, EH25 9RG, UK; National Heart and Lung Institute, Imperial College London, London, SW3 6LY, UK; Institute of Infection, Veterinary and Ecological Sciences, University of Liverpool, Liverpool, L3 5RF, UK; NIHR Health Protection Research Unit in Emerging and Zoonotic Infections, Liverpool, Liverpool, L69 7BE, UK; University of Bristol, Bristol, BS8 1QU, UK; NIHR Health Protection Research Unit in Emerging and Zoonotic Infections, Liverpool, Liverpool, L69 7BE, UK; UK-Health Security Agency, Salisbury, SP4 0JG, UK; Institute of Infection, Veterinary and Ecological Sciences, University of Liverpool, Liverpool, L3 5RF, UK; Institute of Infection, Veterinary and Ecological Sciences, University of Liverpool, Liverpool, L3 5RF, UK; NIHR Health Protection Research Unit in Emerging and Zoonotic Infections, Liverpool, Liverpool, L69 7BE, UK; Infectious Diseases Horizontal Technology Centre (ID HTC), A*STAR, 138632, Singapore

**Keywords:** SARS-CoV-2, coronavirus, COVID-19, subgenomic mRNA, sgmRNA, transcriptional regulatory sequences, nanopore, direct RNA sequencing, RNA modification

## Abstract

Severe acute respiratory syndrome coronavirus 2 (SARS-CoV-2) has a complex strategy for the transcription of viral subgenomic mRNAs (sgmRNAs), which are targets for nucleic acid diagnostics. Each of these sgmRNAs has a unique 5′ sequence, the leader–transcriptional regulatory sequence gene junction (leader–TRS junction), that can be identified using sequencing. High-resolution sequencing has been used to investigate the biology of SARS-CoV-2 and the host response in cell culture and animal models and from clinical samples. LeTRS, a bioinformatics tool, was developed to identify leader–TRS junctions and can be used as a proxy to quantify sgmRNAs for understanding virus biology. LeTRS is readily adaptable for other coronaviruses such as Middle East respiratory syndrome coronavirus or a future newly discovered coronavirus. LeTRS was tested on published data sets and novel clinical samples from patients and longitudinal samples from animal models with coronavirus disease 2019. LeTRS identified known leader–TRS junctions and identified putative novel sgmRNAs that were common across different mammalian species. This may be indicative of an evolutionary mechanism where plasticity in transcription generates novel open reading frames, which can then subject to selection pressure. The data indicated multiphasic abundance of sgmRNAs in two different animal models. This recapitulates the relative sgmRNA abundance observed in cells at early points in infection but not at late points. This pattern is reflected in some human nasopharyngeal samples and therefore has implications for transmission models and nucleic acid–based diagnostics. LeTRS provides a quantitative measure of sgmRNA abundance from sequencing data. This can be used to assess the biology of SARS-CoV-2 (or other coronaviruses) in clinical and nonclinical samples, especially to evaluate different variants and medical countermeasures that may influence viral RNA synthesis.

## Importance

When infecting cells, severe acute respiratory syndrome coronavirus 2 (SARS-CoV-2) not only replicates its genome but also makes molecules called subgenomic mRNAs (sgmRNAs) that are used as the template for many of the viral proteins, including the spike glycoprotein. The sgmRNAs can be found only in infected cells, and therefore their presence and ratio in a clinical sample are indicative that viral RNA synthesis has occurred and infected cells are present. The sgmRNAs are targets for diagnostic assays. We have developed a rapid informatics methodology (LeTRS) to identify these unique molecules from multiple types of sequencing data generated in response to the coronavirus disease 2019 (COVID-19) pandemic. We used this pipeline to follow the pattern of sgmRNA abundance in nasopharyngeal samples taken from nonhuman primate models and clinical samples from humans. We identified putative novel sgmRNAs that may point to a potential new evolutionary mechanism in the virus. The data indicated that SARS-CoV-2 RNA synthesis (and by inference infection) may occur in waves, and this has implications for diagnostics and modeling of disease spread.

## Introduction

Various sequencing approaches are used to characterize severe acute respiratory syndrome coronavirus 2 (SARS-CoV-2) RNA synthesis in cell culture [1, 2], *ex vivo* models [3], and clinical samples. This can include nasopharyngeal swabs from patients with coronavirus disease 2019 (COVID-19) [4] to postmortem samples from patients who died of severe disease [5]. Bioinformatic interrogation of these data can provide critical information on the biology of the virus. SARS-CoV-2 genomes are message sense, and the 5′ two-thirds of the genome is translated and proteolytically cleaved into a variety of functional subunits, many of which are involved in the synthesis of viral RNA [6]. The remaining one-third of the genome is expressed through a nested set of subgenomic mRNAs (sgmRNAs). These have common 5′ and 3′ ends with the coronavirus genome, including a leader sequence, and are thus coterminal. Many studies have shown that the sgmRNA located toward the 3′ end of the genome, which encodes the nucleoprotein, generally has a higher abundance than those located immediately after the 1a/b region and the genome itself in infected cells [7, 8]. However, there is not necessarily a precise transcription gradient of the sgmRNAs. The 5′ leader sequence on the sgmRNAs is immediately abutted to a short sequence called a transcriptional regulatory sequence (TRS) that is involved in the control of sgmRNA synthesis [9, 10]. These TRSs are located along the genome and are proximal to the start codons of the open reading frames [11]. In the negative sense, the TRSs are complementary to a short portion of the genomic leader sequence. The TRS is composed of a short core motif that is conserved and flanking sequences [9, 10, 12]. The core motif of the TRS in SARS-CoV-2 is ACGAAC.

The prevailing thought is that synthesis of sgmRNAs involves a discontinuous step during negative strand synthesis [13, 14]. A natural consequence of this is recombination resulting in insertions and deletions (indels) in the viral genome and the formation of defective viral RNAs. Thus, the identification of the leader/sgmRNA complexes by sequencing provides information on the abundance of the sgmRNAs and evidence that transcription has occurred in the tissue being analyzed. In terms of clinical samples, if infected cells are present, then the leader/sgmRNA “fusion” sequence can be identified and inferences made about active viral RNA synthesis from the relative abundance of the sgmRNAs. In the absence of published data from human challenge models, the kinetics of virus infection are unknown, and most studies will begin with detectable viral RNA on presentation of the patient with clinical symptoms. In general, models of infection of humans with SARS-CoV-2 assume an exponential increase in viral RNA synthesis followed by a decrease, as antibody levels increase [15].

To investigate the presence of SARS-CoV-2 sgmRNAs in clinical (and other) samples, a bioinformatics tool (LeTRS) was developed to analyze sequencing data from SARS-CoV-2 infections by identifying the unique leader–TRS gene junction site for each sgmRNA. The utility of this tool was demonstrated on cultured cells infected with SARS-CoV-2, nasopharyngeal samples from humans with COVID-19, and longitudinal analysis of nasopharyngeal samples from two nonhuman primate models infected with SARS-CoV-2. The tool is adaptable for other coronaviruses. The results have implications for virus biology, diagnostics, and disease modeling.

## Results

A tool, LeTRS (named after the leader–TRS fusion site), was developed to detect and quantify defined leader gene junctions of SARS-CoV-2 (and other coronaviruses) from multiple types of sequencing data. This was used to investigate SARS-CoV-2 sgmRNA synthesis in humans and nonhuman primate animal models. LeTRS was developed using the Perl programming language, including a main program for the identification of sgmRNAs and a script for plotting graphs of the results. The tool accepts FASTQ files derived from Illumina paired-end or Oxford Nanopore sequencing (amplicon or direct RNA) or BAM files produced by a splicing alignment method with a SARS-CoV-2 genome (Supplementary Fig. 1). Note that SARS-CoV-2 sgmRNAs are not formed by splicing, but this is the apparent observation from sequencing data because of the discontinuous nature of transcription. By default, LeTRS analyzes SARS-CoV-2 sequence data by using 10 known leader–TRS junctions and an NCBI reference genome (NC_045 512.2) to identify leader-dependent canonical sgmRNAs. However, given the potential heterogeneity in the leader–TRS region and potential novel (leader-dependent noncanonical) sgmRNAs, the user can also provide customized leader–TRS junctions and SARS-CoV-2 variants as a reference. As there is some heterogeneity in the leader–TRS sites, LeTRS was also designed to search for multiple features of sgmRNAs. This included to annotate the leader–TRS junction in a given interval, report on the leader (20 nucleotides at the 3′ end) and TRS sequences, translate the first predicted open reading frame (orf) of the sgmRNA, and find the conserved ACGAAC sequences in the TRS. LeTRS can also be used to identify the sequencing reads with leader-independent fusion sites that has been suggested to probably produce unknown ORFs yielding functional products [2]. The tool was designed to investigate very large data sets that are produced during sequencing of multiple samples.

### Combinations of read alignments with the leader–TRS junction that are considered for identifying leader–TRS junction sites

**Figure 1.**
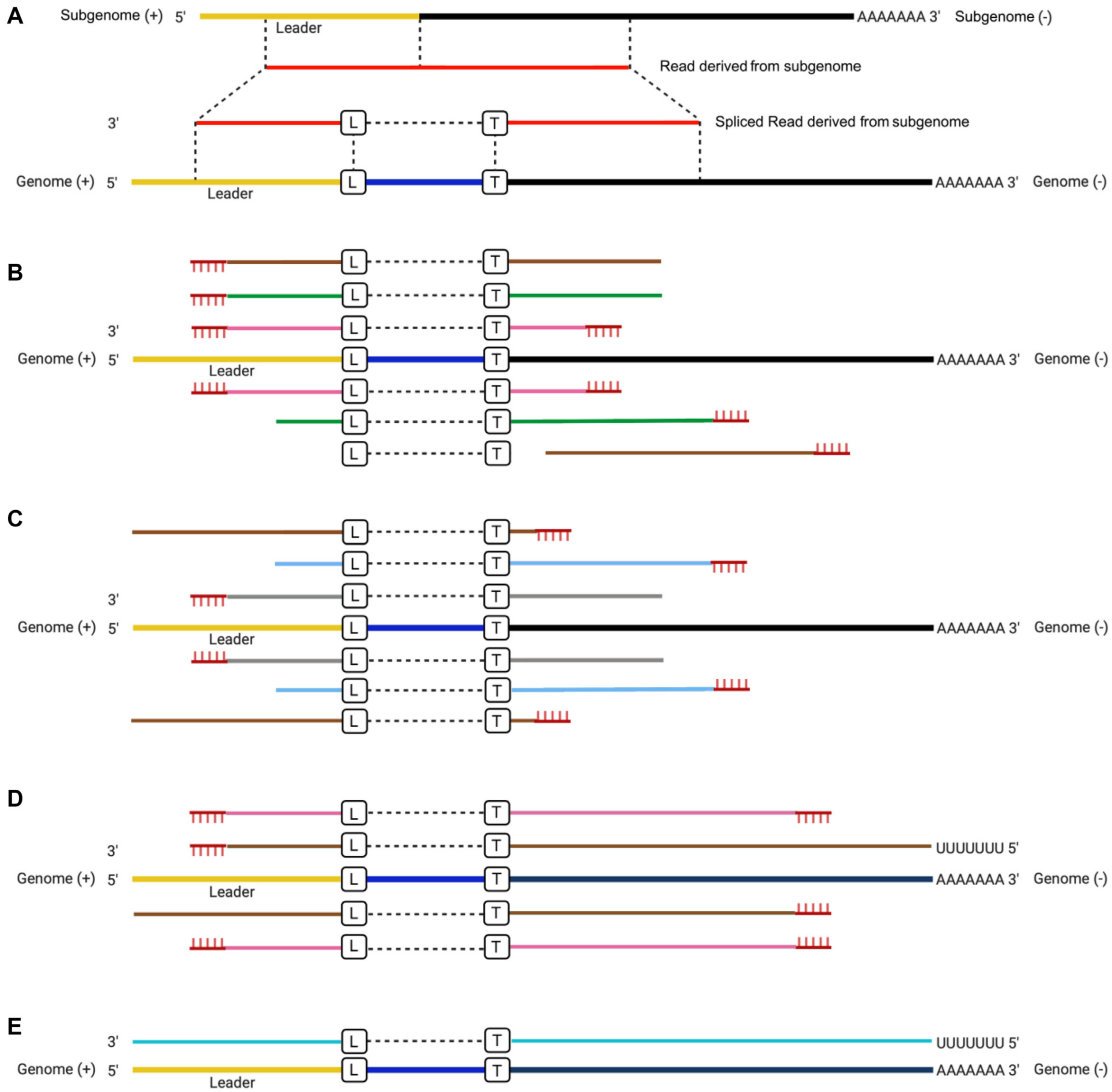
(A) Illustration of reads derived from subgenomic mRNAs (sgmRNAs) mapped onto the severe acute respiratory syndrome coronavirus 2 (SARS-CoV-2) reference genome with a splicing method. We note that splicing does not occur in coronaviruses, but this is the apparent observation of a fusion event between different parts of the genome. (B, C) Illustration of the possible type of reads mapped on the SARS-CoV-2 reference genome for the paired-end Illumina amplicon sequencing, where the lines with the same color implied paired reads. (D) Nanopore amplicon sequencing and (E) Nanopore direct RNA sequencing of the SARS-CoV-2 genome and sgmRNAs. L and B in the boxes indicate the leader–transcriptional regulatory sequence (TRS) breaking sites on the leader side and TRS side, respectively, although we note these are where the apparent fusion site occurs. Yellow indicates the leader region, black is the TRS and gene sequence, and red indicates a sequence read that maps to the SARS-CoV-2 sequence. Blue is a sequence that is present between the leader sequence and the TRS. For (B) and (C), the same color (brown, green, and pink) indicates that same paired read. For (B), the paired read contains both primers. For (C), the gray and light blue color is a paired read but only contains one primer sequence at any end. The vertical hash lines on (B), (C), and (D) indicate the position of a primer.

Various approaches have been used to sequence the SARS-CoV-2 genome, and in most cases, this would also include any sgmRNAs as they are 3′ coterminal and share a common sequence extending from the 3′ end. Methods such as ARTIC [16], MIDNIGHT [16], and RSLA [4] use primer sets to generate overlapping amplicons that span the entire genome and also amplify sgmRNA. Included is a primer to the leader sequence, so that the unique 5′ end of these moieties is sequenced. Primer sets of ARTIC, MIDNIGHT, and RSLA are generally formed of two pools. For the ARTIC method, at the time of the study, only pool 1 included a forward primer located within the leader region (<80 nts) of the SARS-CoV-2 genome [17]. Therefore, LeTRS was designed with a function to analyze reads in the primer pool 1, pool 2, or both pools. Unbiased sequencing can also be used in methodologies to identify SARS-CoV-2 sequence. Data in the GISAID database have been generated by Oxford Nanopore–based (minority) or Illumina-based (majority) approaches. These can give different types of sequencing reads derived from the sgmRNAs that can be mapped back on the reference SARS-CoV-2 genome by splicing alignment (Fig. 1A). For example, several different types of reads can be derived from mapping Illumina-based amplicon sequencing onto the reference viral genome (Fig. 1B and C). During the polymerase chain reaction (PCR) stage, the extension time allows the leader–TRS region on the sgmRNAs to be PCR amplified by the forward primer and the reverse primer before and after the leader–TRS junction in different primer sets, respectively. If the amplicon had a length shorter than the Illumina read length (usually 100–250 nts), both the forward and reverse primers would be detected at the ends of each paired read (Fig. 1B, pink lines). If the amplicon was longer than the Illumina read length, primer sequence would be found only at one end of each paired read (Fig. 1B, green and brown lines), with the possibility of one of the paired reads having a fusion site. The extension stage could also proceed with a single primer using cDNA derived from the sgmRNA as a template. This type of PCR product has a very low amplification efficiency but theoretically could also generate the same Illumina paired-end read with a single primer sequence at one end (Fig. 1C). These paired-end reads could include the full length of the leader sequence but might not reach the 3′ end of the sgmRNA, because of the limitation of Illumina sequencing length and extension time (Fig. 1C). Also, unless there are cryptic TRSs located toward the 3′ end of the genome, all sgmRNAs would be expected to be larger than the Illumina sequencing length.

In contrast, the different types of read alignment in the Nanopore-based amplicon are simpler to assign. The longer reads that tend to be generated by Nanopore sequencing (depending on optimization) enable the capture of full-length sequences of all amplicons. Provided the leader sequence is included as a forward primer, most of the reads spanning the leader–TRS junction would contain the forward and reverse primer sequences at both ends (Fig. 1D, pink lines). If the extension time allowed, single primer PCR amplification could take the Nanopore amplicon sequencing reads to both the 3′ and 5′ ends of the sgmRNAs, and these types of reads would have a primer sequence only at one end (Fig. 1D, brown lines). In the Nanopore direct RNA sequencing (dRNAseq) approach, the full-length sgmRNA could be sequenced and mapped entirely on the leader and TRS–orf regions (Fig. 1E).

### Evaluation of LeTRS on SARS-CoV-2 infection in cell culture

**Figure 2.**
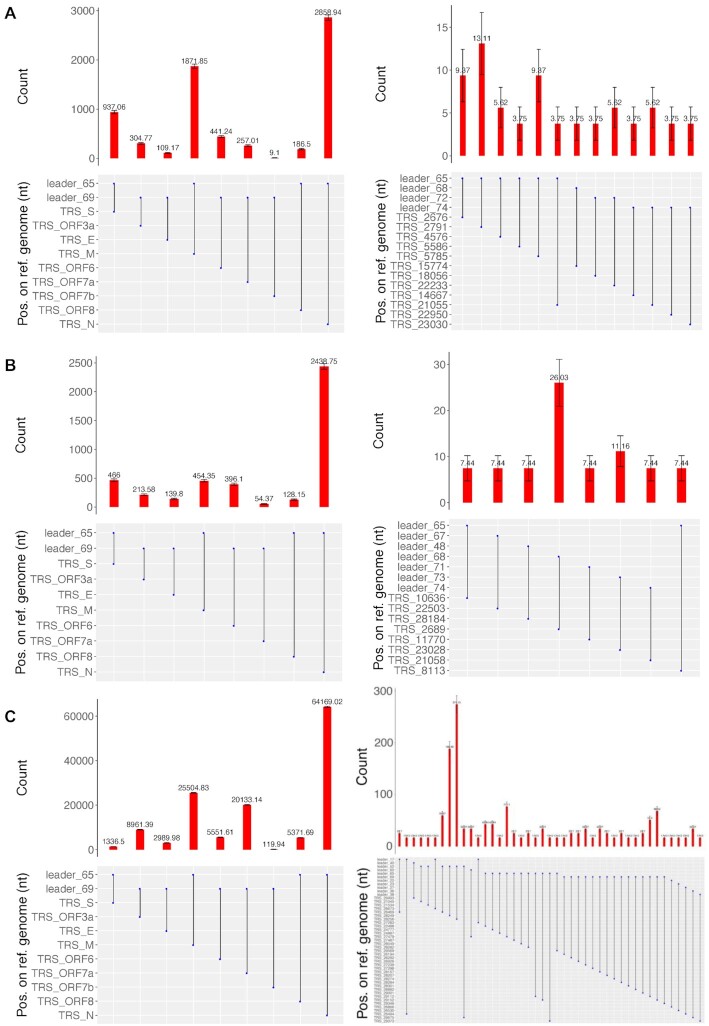
Analysis of reads mapping to the leader–transcriptional regulatory sequence (TRS) gene junctions with at least one primer sequence at either end in sequencing data from hACE2-A549 cells infected with severe acute respiratory syndrome coronavirus 2 and sequenced using (A) an ARTIC-Nanopore approach, (B) an ARTIC-Illumina approach, and (C) a Nanopore direct RNA sequencing approach. The data correspond to that shown in detailed in Supplementary Tables 1, 2, and 3. The standard deviation of a binomial distribution was calculated to generate error bars. The data are presented as a histogram with a normalized count for each subgenomic mRNA (sgmRNA) starting at a particular position in the leader sequence as indicated in the line diagram underneath. For each panel (A, B, and C), the expected sgmRNA pattern is shown on the left and novel sgmRNAs are shown on the right.

In order to assess the ability of LeTRS to identify the leader–TRS junctions from sequencing information, a total RNA sample was prepared at 72 hours postinfection (hpi) from hACE2-A549 cells infected with SARS-CoV-2 (a lineage B isolate). This RNA was sequenced using an amplicon-based approach (ARTIC) with either Nanopore (ARTIC-Nanopore) or Illumina (ARTIC-Illumina), or alternatively by a Nanopore dRNAseq approach [22]. The ARTIC-Nanopore (Fig. 2A, Supplementary Table 1) and ARTIC-Illumina (Fig. 2B, Supplementary Table 2) sequencing data were evaluated with LeTRS by setting the analysis to both primers' pools. For dRNAseq (Fig. 2C, Supplementary Table 3), data were evaluated with LeTRS using the default setting. All the major known leader–TRS gene junctions were identified by these sequencing methods. Analysis demonstrated an expected pattern of abundance of the leader–TRS gene junctions, with the leader–TRS nucleoprotein gene junction being most abundant (Fig. 2A, B, and C; Supplementary Tables 1, 2, and 3). Novel low-abundance leader–TRS gene junctions were also identified (Fig. 2A, B, and C; Supplementary Tables 1, 2, and 3). These known and novel leader–TRS junctions were also known as leader-dependent canonical and noncanonical fusions, respectively [2]. LeTRS also has a function to identify leader-independent long-distance fusion (>5,000 nt) and local joining, yielding a deletion between proximal sites (20–5000 nt distance) in the sequencing reads. The leader-independent fusions (coverage ≥2) are shown in Supplementary Tables 1, 2, and 3. Indel sequencing errors are frequent (defined as <20 nucleotides), especially in Nanopore sequencing data, and therefore it is difficult to find precise fusion (apparent splicing) sites in this case [18]. However, some of the novel leader–TRS junctions (noncanonical fusions) and leader-independent fusions in the test sample were supported by all three sequencing methods (Supplementary Fig. 2) with similar fusion sites. Many local fusions/deletions within the orf3, E, M, orf6, orf7a, orf7b, orf8, and N genes were identified (Supplementary Fig. 2G, H, and I) and confirmed previous findings [2, 19], indicating these are common events. Some of the novel leader–TRS junctions (noncanonical fusions) and leader-independent fusions may be the result of sequencing or reverse transcription errors, especially those with low abundance (Supplementary Tables 1, 2, and 3; Supplementary Fig. 2). The ARTIC-Illumina approach identified fewer novel leader–TRS junctions (noncanonical fusions) and leader-independent fusions than the other two sequencing methodologies, probably due to lower sequencing coverage (Supplementary Tables 1, 2, and 3).

For ARTIC approaches, LeTRS was designed to analyze reads in the primer pool 1, pool 2, or both pools. Only the ARTIC pool 1 included a forward primer that is located within the leader region (<80 nts) of the SARS-CoV-2 genome. The leader–TRS regions of sgmRNAs can be PCR amplified by both forward and reverse primers in ARTIC pool 1 but only reverse primers in ARTIC pool 2. The read counts evaluated by LeTRS in both ARTIC-Nanopore and ARTIC-Illumina were compared in the test data for pools 1 and 2 and found only very few reads/read pairs contained the correct primers (Supplementary Tables 4 and 5), suggesting the primers in ARTIC pool 2 generally do not contribute to sequencing of leader–TRS regions.

### Comparison with other informatic tools that can identify leader–TRS gene junctions

Other tools have been developed to identify sgmRNAs from ARTIC-Illumina and ARTIC-Nanopore sequencing data, such as Periscope (v0.1.0) [20], SARS-CoV-2-leader [21, 22], and SuPER [23]. These tools were compared with LeTRS, as shown in Table 1. LeTRS and Periscope used the FASTQ files as input, while SARS-CoV-2–leader and SuPER required SAM files from a user-generated alignment. Searching fusion site and sequences tag in the sequencing reads are two major methods used. LeTRS and SuPER analyzed the fusion/splicing information in sequence reads achieved by an alignment program and also took account of the conserved ACGAAC sequences in the TRS. Periscope and SARS-CoV-2–leader are based on searching for a short tag sequence in the leader from sequencing reads. However, searching for a short tag sequence in the leader with the high error rate associated with Nanopore data can be challenging. LeTRS and Periscope use primer information to differentiate reads mapping to amplicons to reads mapping from original virus genomes. Besides Periscope, output from dRNAseq is supported by the other available tools. Illumina sequencing reads are usually short (<250 bases), paired, and sequenced from both ends. If both reads in a single pair contain a fusion site, this will be counted twice by the other three tools (Fig. 1B, green and pink). However, if only one of the reads in the pair contains a fusion site, it will be counted once (Fig. 1B, brown). This leads to biased counting. LeTRS takes this into account by treating each read pair as a single event. LeTRS also has a unique function to analyze reads in the primer pool 1, pool 2, or both pools from ARTIC-based sequencing (Table 1). Accuracy, sensitivity, specificity, and the F-measure score were calculated with simulated Illumina and Nanopore sequencing reads. All of these tools performed better for analyzing the simulated Illumina sequencing reads compared to the simulated Nanopore sequencing reads (Table 1). LeTRS showed greater sensitivity and F-measure score than the other tools for processing the simulated Nanopore sequencing reads (Table 1).

**Table 1:** Comparison of other tools with LeTRS

		LeTRS	Periscope	SARS-CoV-2–leader	SuPER
Input files		fastq	fastq	bam/sam	sam
Consideration of amplicon primer information used		yes	yes	no	no
Consideration of paired-end Illumina data		yes	no	no	no
Consideration of amplicon primer pool		yes	no	no	no
Consideration of the ACGAAC box		yes	no	no	yes
Support of amplicon Illumina data		yes	yes	yes	yes
Support of amplicon Nanopore data		yes	yes	yes	yes
Support of Nanopore dRNAseq data		yes	no	yes	yes
Method		Fusion site searching	Sequences tag searching	Sequences tag searching	Fusion site searching
Accuracy	ARTIC-Illumina	1.0000	0.9998	0.9998	0.9996
	ARTIC-Nanopore	0.9985	0.9981	0.9980	0.9979
	Nanopore dRNAseq	0.9982		0.9948	0.9937
Sensitivity	ARTIC-Illumina	0.9997	0.9498	0.9644	0.9230
	ARTIC-Nanopore	0.6294	0.5326	0.5154	0.4843
	Nanopore dRNAseq	0.8448		0.5949	0.4817
Specificity	ARTIC-Illumina	1.0000	1.0000	1.0000	1.0000
	ARTIC-Nanopore	1.0000	1.0000	1.0000	1.0000
	Nanopore dRNAseq	1.0000		1.0000	1.0000
F-measure	ARTIC-Illumina	0.9998	0.9499	0.9655	0.9243
	ARTIC-Nanopore	0.7621	0.6699	0.6611	0.6215
	Nanopore dRNAseq	0.9157		0.7140	0.5934

Accuracy, sensitivity, specificity, and F-measure score were calculated with simulated Illumina and Nanopore sequencing reads for the known subgenomic mRNAs.

To compare the performance to LeTRS, these three tools were evaluated using the hACE2-A549 cell culture sample sequenced by ARTIC-Nanopore, ARTIC-Illumina, and Nanopore dRNAseq. Using the ARTIC-Nanopore sequencing data, all the tools reported a similar number of read counts for the 10 known sgmRNAs (Supplementary Fig. 3A). LeTRS showed fewer counts for the ARTIC-Illumina than the other three tools because of considering read pairs (Supplementary Fig. 3B). Interestingly, Periscope also identified fewer nucleoprotein sgmRNAs with the ARTIC-Illumina sequencing data (Supplementary Fig. 3B). As of writing, Periscope does not yet support Nanopore dRNAseq data; therefore, LeTRS, SARS-CoV-2–leader, and SuPER were compared. LeTRS and SARS-CoV-2–leader generally identified more dRNAseq reads than SuPER, especially for the nucleoprotein sgmRNA (Supplementary Fig. 3C). Finally, the ratio of read counts with the 10 known sgmRNAs (S:orf3:E:M:orf6:orf7a:orf7b:orf8:N:orf10) were compared, and the three tools showed almost an identical ratio when analyzing data from the same sequencing methods (Supplementary Fig. 3D). ARTIC-Nanopore and Nanopore dRNAseq resulted in a higher ratio of read counts with M and orf7a, respectively (Supplementary Fig. 3D). The read counts ratio of sgmRNAs mapping to spike was much lower with dRNAseq approaches (Supplementary Fig. 3D).

### Normalization of read counts for sgmRNA

Normalization of read counts has been widely used for RNAseq in the comparison of gene expression level across samples [24]. The normalization is generally based on the ratio of reads mapped on the gene to the total number of reads in that sample. These tools use this algorithm for the normalization of read counts in searching for sgmRNA [20, 25]. LeTRS also incorporated a method to differentiate the total reads mapped (i) or whether the reads have forward primer only (ii), reverse primer only (iii), both primers (iv), or at least one primer (v) present. This is achieved by (i) the total number of reads mapped on the SARS-CoV-2 genome for the number of reads of the leader–TRS fusion site as the numerator; (ii) the total number of reads with forward primers only for the number of reads of the leader–TRS fusion site, with forward primers only as the numerator; (iii) the total number of reads with reverse primers only for the number of reads of the leader–TRS fusion site, with reverse primers only as the numerator; (iv) the total number of reads with both primers for the number of reads of the leader–TRS fusion site with both as the numerator, and (v) the total number of reads with at least one primer on one side for the number of reads of the leader–TRS fusion site, with at least one primer as the numerator (notes in Supplementary Tables 1, 2, and 3).

**Figure 3.**
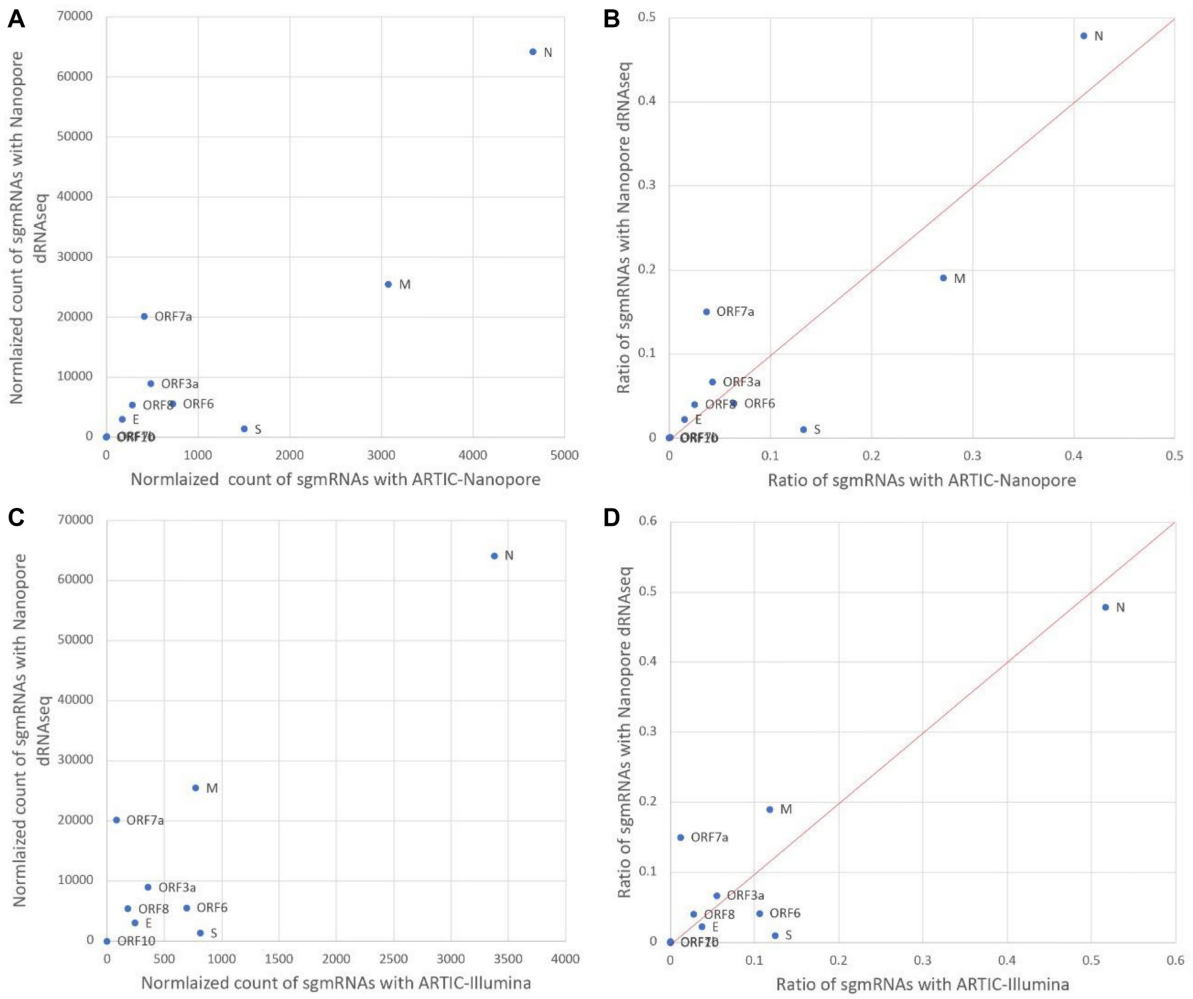
An X-Y/scatterplot using normalized counts of subgenomic mRNAs (sgmRNAs) (with >5 A residues at the 3′ end—indicative of a polyA tail for the direct RNA sequencing [dRNAseq] data). To generate the scatterplots, Nanopore dRNAseq data were plotted against the either the normalized count (at least one primer sequence) of sgmRNAs with (A) ARTIC-Nanopore sequencing data and (C) ARTIC-Illumina sequencing data or provided as ratio (B) and (D), respectively, for S:orf3:E:M:orf6:orf7a:orf7b:orf8:N:orf10 (using data from Supplementary Tables 3, 4, and 5).

Because LeTRS considers the primers, pool 1, pool 2, or both pools, normalization could be observed in ARTIC pool 1 only to minimize the effect from ARTIC pool 2 since primers in ARTIC pool 2 are almost not involved in the sequencing of leader–TRS regions (as described above). For the same RNA derived from the hACE2-A549 cell culture sample sequenced by ARTIC-Nanopore, ARTIC-Illumina, or Nanopore dRNAseq approaches, the normalized counts for the known sgmRNAs were much smaller with pool 1 of PCR-based amplicon methods (ARTIC-Nanopore and ARTIC-Illumina) than the Nanopore dRNAseq approach (Fig. 3A and C for the reads with at least one primer sequence; Supplementary Tables 3, 4, and 5). However, the normalized counts with ARTIC-Nanopore and ARTIC-Illumina showed the same ratio of known sgmRNAs as the Nanopore dRNAseq approach, except for sgmRNA mapping to S and orf7a (Fig. 3B and D for the reads with at least a primer sequence). PCR-based approaches increased the value of the denominator and reduced the normalized count, because a full length of sgmRNA was counted once with the dRNAseq approach compared to many times with the amplicon approaches. ARTIC-Illumina had fewer normalized counts than ARTIC-Nanopore probably due to the sequencing bias of Illumina during PCR [26]. Thus, if the samples were sequenced with the same methodology, they were comparable. With a PCR-based method, a normalized count should be used to show the relative difference between samples.

LeTRS identified many reads with only one primer (one-sided amplification) with the PCR-based amplicon methods (Supplementary Tables 4 and 5). The ratio of reads with either forward and/or reverse primers was compared for each sgmRNA to the overall ratios of reads, with forward primers only or reverse primers only, both primers in all mapped reads of pool 1 and pool 2, and the mapped reads with any fusion sites of pool 1 and pool 2. This indicated that abundant reads were identified with a single pattern and these were similar to reads mapping to sgmRNAs, suggesting a one-sided amplification is associated with amplicon-based approaches (Supplementary Fig. 4).

### Analysis of sequencing data from longitudinal nasopharyngeal samples taken from two nonhuman primate models of COVID-19 indicated multiphasic sgmRNA synthesis and novel sgmRNAs

Part of the difficulty of studying SARS-CoV-2 and the disease COVID-19 is establishing the sequence of events from the start of infection. Most samples from humans are from nasopharyngeal aspirates taken when clinical symptoms develop. This tends to be 5 to 6 days postexposure. In the absence of a human challenge model, animal models can be used to study the kinetics of SARS-CoV-2 [27, 28]. Two separate nonhuman primate (NHP) models, cynomolgus and rhesus macaques, were established for the study of SARS-CoV-2 that mirrored disease in most humans [27]. To study the pattern of sgmRNA synthesis over the course of infection, nasopharyngeal samples were sequentially gathered daily from 1 to 18 days postinfection (dpi) from the two NHP models. RNA was purified from these longitudinal samples as well as the inoculum virus and viral RNA sequenced using ARTIC-Illumina.

**Figure 4.**
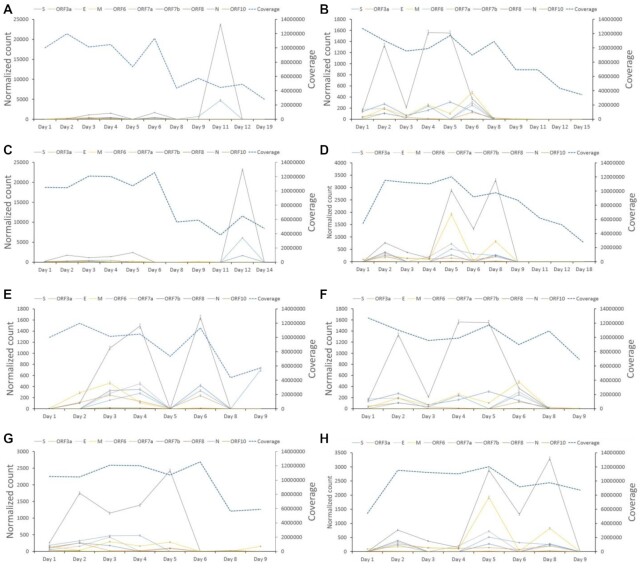
Analysis of the abundance of reads mapping to the leader–transcriptional regulatory sequence (TRS) gene junctions that have at least one primer sequence at either end in longitudinal nasopharyngeal samples taken from two nonhuman primate models infected with severe acute respiratory syndrome coronavirus 2 (SARS-CoV-2). The time postinfection in days is indicated on the x-axis. The normalized count (read count/total number of reads mapped on the reference genome * 1,000,000) of the leader–TRS gene junction abundance is shown on the left-hand y-axis with each unique leader–TRS gene junction color coded. The right-hand y-axis is a measure of the total depth of coverage for SARS-CoV-2 in that sample. Note the two scales are different. SARS-CoV-2 was amplified and sequenced by ARTIC-Illumina. The data are organized into groups of animals for the cynomolgus macaque groups 1 and 2 (A/E and B/F) and rhesus macaque groups 1 and 2 (C/G and D/H). E, F, G, and H zoom in to see the details of A, B, C, and D for days 1 to 9. The data correspond to that shown in Supplementary Table 7. Standard deviation of a binomial distribution was calculated to provide error bars.

As expected, analysis of the sequence data using LeTRS from the inoculum used to infect the NHPs indicated that leader gene junctions could be identified, but these did not follow the pattern of abundance of leader–TRS gene junctions found in infected cells in culture, where the leader TRS nucleoprotein gene junction was most abundant (Supplementary Fig. 5). The inoculum would be expected to contain mostly genomic RNA found in virions. In contrast, analysis of the longitudinal sequencing data from nasopharyngeal aspirates from the NHP model using LeTRS identified leader–TRS gene junctions associated with the major sgmRNAs (Fig. 4, Supplementary Table 7) as well as novel leader–TRS gene junction sites (Supplementary Figs. 6 and 7). Analyzing the abundance of the leader–TRS gene junctions for both model species over the course of infection revealed a phasic nature of sgmRNA synthesis in pool 1 to minimize the effect from ARTIC pool 2 (Fig. 4). The leader–TRS nucleoprotein gene junction was the most abundant, and there was a phasic pattern of potential sgmRNA abundance identified with the ARTIC-Illumina method (Fig. 4). For both species, viral load and hence sgmRNA abundance had decreased by 8 and 9 dpi.

### Analysis of leader–TRS gene junction in human samples revealed expected and aberrant abundances of sgmRNAs

**Figure 5.**
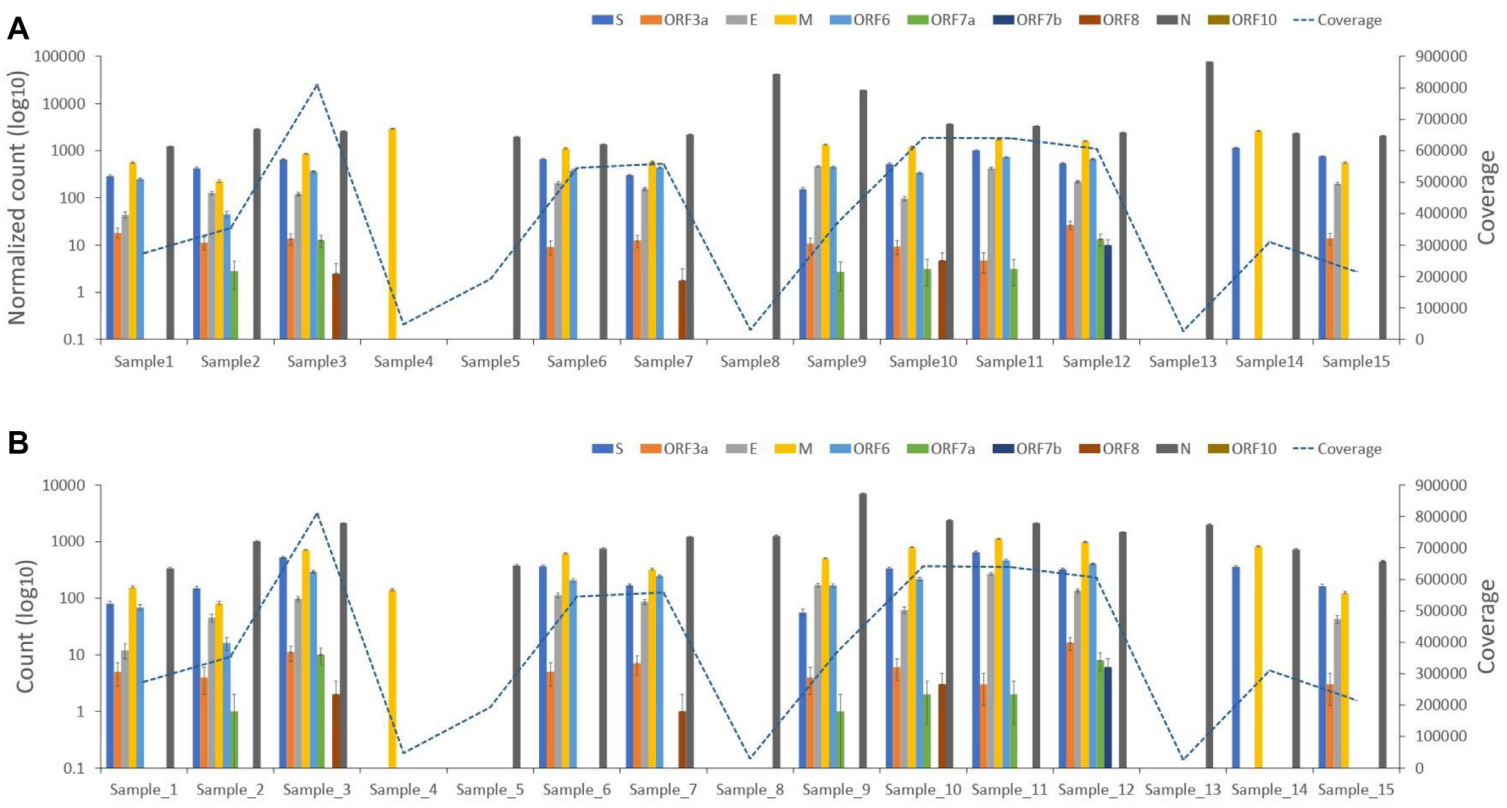
Plots of normalized peak counts (A) and peak counts (B) of leader–transcriptional regulatory sequence gene junctions of reads with at least one primer sequence at either end derived from sequence data from 15 human patients sequenced with the ARTIC-Illumina approach and analyzed by using sequence derived from pool 1 primers. The data correspond to that shown in Supplementary Table 8. Standard deviation of a binomial distribution was calculated to provide error bars.

**Figure 6.**
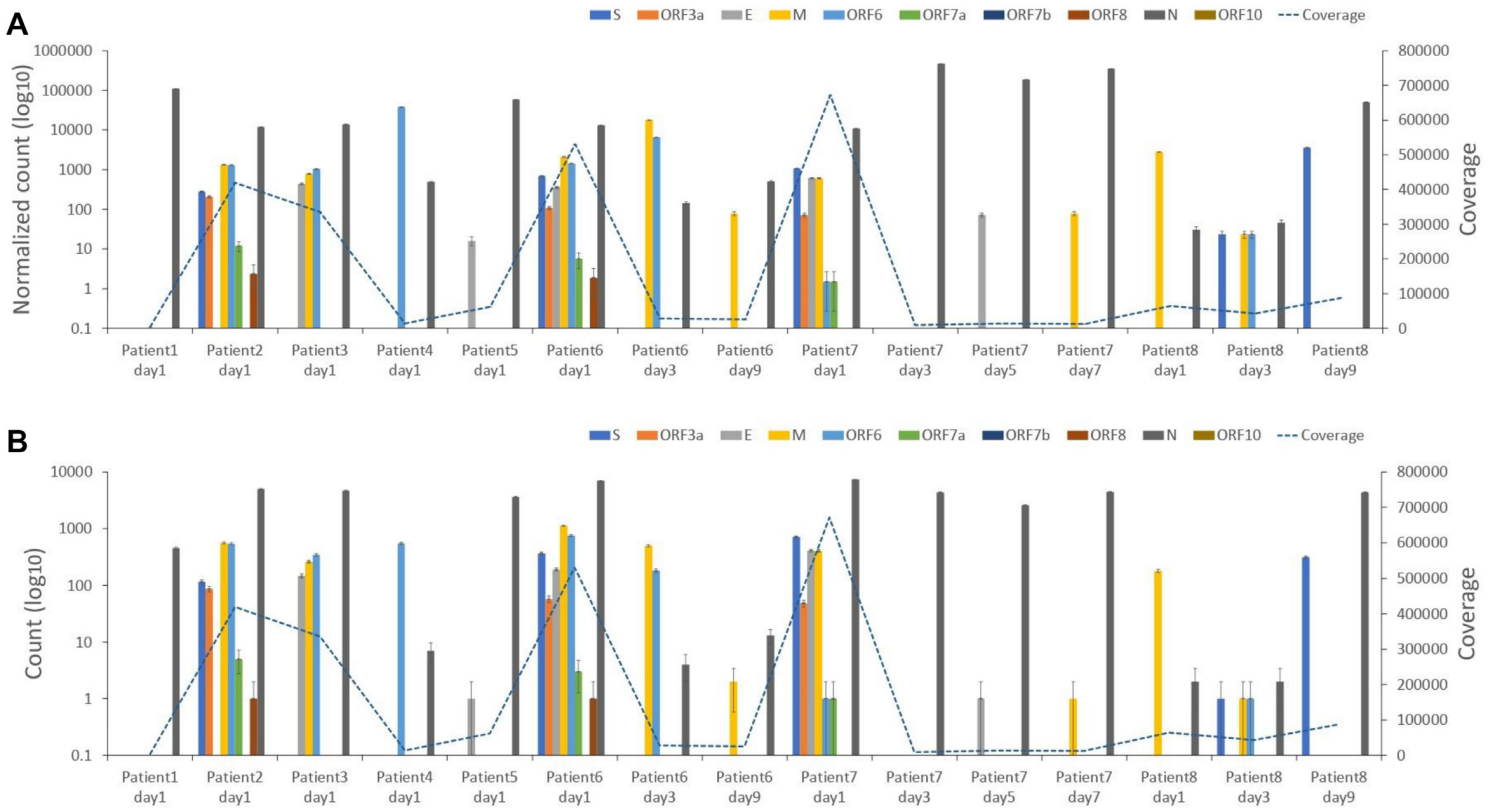
Plots of normalized peak counts (A) and peak counts (B) of leader–transcriptional regulatory sequence gene junctions of reads with at least one primer sequence at either end derived from sequence data from 15 human patients sequenced with the ARTIC-Nanopore approach and analyzed by using sequence derived from pool 1 primers. The data correspond to that shown in Supplementary Table 9. Standard deviation of a binomial distribution was calculated to provide error bars.

To investigate the pattern of leader–TRS gene junction abundance during infection of SARS-CoV-2 in humans, nasopharyngeal swabs from patients with COVID-19 were sequenced by ARTIC-Illumina (using samples from COG-UK) (*n* = 15 patients) (Fig. 5, Supplementary Table 8) or by ARTIC-Nanopore (using samples from ISARIC-4C) (*n* = 15 patients) (Fig. 6, Supplementary Tables 9 and 10). In several samples, leader–TRS gene junctions were identified and followed an expected pattern, with the nucleoprotein gene junction being the most abundant (e.g., sample 1 in Fig. 5A and B, patient 2 on day 1 in Fig. 6A and B). However, in several of the samples, there was very large representation of a single leader–TRS gene junction (e.g., samples 4 and 5 in Fig. 5A and B). These tended to map to the nucleoprotein gene (samples 5, 8, and 13 in Fig. 5A and B). The heterogeneity in abundance of leader–TRS gene junctions was reminiscent of that from the NHP study with a defined and expected pattern near the start of infection but then becoming phasic. The samples gathered under ISARIC-4C were from hospitalized patients and permitted analysis in relation to reported date of symptom onset and sequential sampling. In general, the data indicated that the first sample on admission to hospital contained an abundance of leader–TRS gene junctions, which resembled the pattern seen in infected cells (patient 6 on day 1 and day 9 in Fig. 6A and B). However, with further days postsample (e.g., patient 7 on day 7 in Fig. 6A and B), the leader–TRS nucleoprotein gene junction was the most abundant and far exceeded any other detectable species. The abundance of leader–TRS nucleoprotein gene junction in the patients at a later stage of infection followed that observed in the NHP model (Fig. 4).

### Analysis of sequencing data from a previously published study investigating SARS-CoV-2 RNA in samples from patients

Recent research detected sgmRNAs mapping to E, ORF7a, and N in swabs up to 14 days in one patient and ORF7a and N in another patient up to 17 days after first detection by using a high-throughput amplicon sequencing method known as the Ion AmpliSeq Coronavirus Research Panel on an Ion S5 XL genetic sequencer. The authors concluded these sgmRNAs may be present for a significant time after active infection due to nuclease resistance and protection by cellular membranes [25]. The sequencing data from this study were reanalyzed using LeTRS and confirmed the finding of sgmRNAs in late infection from the two patients (Supplementary Table 11). Apart from nuclease resistance and protection by cellular membranes, a phasic pattern of sgmRNA synthesis may also contribute to the presence of sgmRNAs at later time points.

### Analysis of sgmRNA modification in longitudinal samples in cell culture

N6-methyladenosine (6mA) is a widely observed modification on cellular RNA, and 5-methylcytosine methylation (5mC) has also been reported on viral RNAs [22]. Methylation of SARS-CoV-2 RNA was examined using sequencing data from the Nanopore dRNAseq approach. Total RNA was purified at 6, 12, and 24 hpi from cells infected with SARS-CoV-2. The total RNA was sequenced and reads mapping to sgmRNAs were extracted with LeTRS for 6mA and 5mC examination. Almost all 10 observed sgmRNAs showed the same number of modification sites of 6mA and 5mC at 6, 12, and 24 hpi (Supplementary Table 12). Modification with 5mC was more abundant than 6mA in all 10 known sgmRNAs (Supplementary Table 12). There were differences in abundance of some sgmRNAs, especially the M and N subgenomic mRNAs (Supplementary Table 12). However, there did not appear to be a relationship between number of methylation sites and the abundance of a particular sgmRNA (Supplementary Table 12).

To further evaluate the relationship between time postinfection and modification by methylation, a paired samples one-sided Wilcoxon test was used. This analysis suggested that the 5mC modification fraction at 24 hpi was significantly less than compared to modification at 6 and 12 hpi (*P* < 0.05), except for ORF7b and ORF10 (Supplementary Figs. 8 and 9; Supplementary Table 13). Modification with 6mA at 24 hpi was also significantly less than at 6 hpi but not at 12 hpi (*P* < 0.05) in S, ORF3a, E, M, ORF6, ORF7a, ORF8, and N.

### Common properties/features of novel leader–TRS gene junctions and sgmRNAs

**Figure 7.**
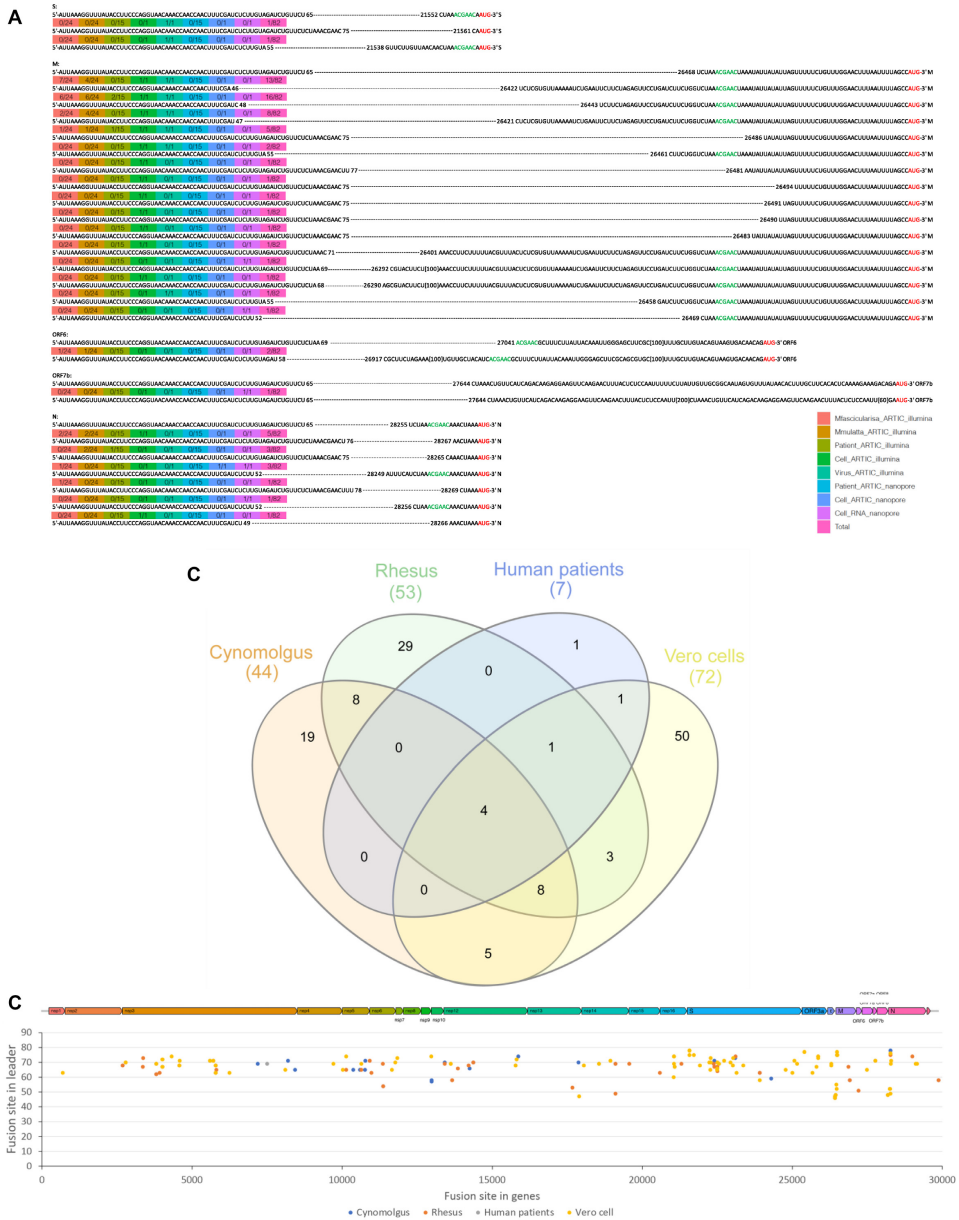
(A) Diagram of novel leader–transcriptional regulatory sequence (TRS) junctions centered on the known gene orf but out of the search interval in the analysis of severe acute respiratory syndrome coronavirus 2 (SARS-CoV-2) RNA from cell culture, nonhuman primate, and human sequencing data. Many novel junctions map to the leader–TRS membrane gene junctions. (B) Venn diagram showing the overlap of novel leader–TRS gene junctions present in SARS-CoV-2–infected cynomolgus and rhesus macaques, human patients, and Vero cells. Data were obtained using the ATRIC-Illumina method (Supplementary Table 15). (C) Virus genome position of the start of the fusion site (y-axis) in the leader sequence plotted against the fusion site present in the gene to show the potential positions of the novel leader–TRS junctions along the SARS-CoV-2 genome (indicated above). The colors present the novel leader–TRS junctions identified in the different experimental condition (cynomolgus and rhesus macaques, human patients, and Vero cells).

The sequencing data from cells infected in culture (Supplementary Table 14), animal models, and clinical samples from humans indicated the presence of novel leader–TRS gene junctions. Their detection generally increased with depth of coverage. Coronavirus replication and transcription is promiscuous, and recombination is a natural result of this, resulting in indels and potential gene rearrangements. Many of these novel leader–TRS junctions were centered on the known gene orf but out of the search interval. These types of leader–TRS gene junctions could be found only with spike, membrane, ORF6, ORF7b, and nucleocapsid orfs, in which the membrane orf was the most common (Fig. 7A). To define what might be genuine novel leader–TRS gene junctions, these were compared across the data in all ARTIC-Illumina data (Fig. 7B, Supplementary Table 15). Five novel leader–TRS gene junctions were identified that were common to all the data, and the majority of these were present immediately 5′ of the membrane orf. The novel leader–TRS gene junctions from LeTRS (Fig. 7C) showed a similar distribution as a previous study, although this study did not detail the precise location [29].

## Discussion

Coronavirus sgmRNAs are only synthesized during infection of cells and therefore their presence in sequence data can be indicative of active viral RNA synthesis. The abundance of the sgmRNAs in infected cells should follow a general pattern where the sgmRNA encoding the nucleoprotein is the most abundant. Identification and quantification of the unique leader–TRS gene junctions for each sgmRNA can be used as a proxy for their abundance.

LeTRS was developed to interrogate sequencing data sets to identify the leader–TRS gene junctions present at the 5′ end of the sgmRNAs. LeTRS was first evaluated and validated on cell culture data from published data sets [2, 30] and from a cell culture experiment as part of this study and then used in an analysis of nasopharyngeal samples from NHP and human clinical samples. The results showed that the positions of the leader–TRS junction sites with peak read counts were the same as the given reference positions. The exception was at the leader–TRS gene junction for orf7b in the Nanopore sequencing. The normalized count results that confirmed the reads spanning the junctions showed that the leader–TRS nucleoprotein gene junction was the most abundant, and orf7b and orf10 were the most infrequent in line with other data [2, 25]. Several low abundant leader–TRS junctions were identified in all of the data sets (Supplementary Fig. 2), with the implication these were from potential lower abundant novel sgmRNAs or represented known sgmRNAs but with different leader–TRS junctions. Likewise, at low frequency, these could represent an aberrant viral transcription, perhaps as a mechanism to generate new orfs for selection, or these could be artifacts of the different sequencing processes (Fig. 2). Traditionally, such sgmRNAs have been first identified in coronaviruses by either northern blot and/or metabolic labeling [8], and sequencing approaches are likely to be more sensitive giving the amplification steps involved. Several other groups have identified novel leader–TRS gene junctions and potential sgmRNAs for other coronaviruses, including avian infectious bronchitis virus [31]. The best way of validating potential novel sgmRNAs would be through matching proteomic data to confirm genuine ORFs [1]. Analysis of several published sequencing data sets identified novel viral RNA molecules that the authors suggested were sgmRNAs containing only the 5′ region of orf1a [32]. Such species are likely to be defective RNAs, which act as templates for replication, rather than sgmRNAs. Interestingly, at later time points postinfection in cell culture, potential novel sgmRNAs were found to be generated nonspecifically [32]. This potentially ties in with a disconnect of leader–TRS gene junctions observed in our study both *in vivo* from the nasopharyngeal samples from latter time points in the NHP models and in humans. This is also shown in published data from SARS-CoV-2 infections in cell culture gathered at later time points compared to earlier time points [2, 30].

Advanced filtering can improve the confidence of the identified leader–TRS junction from sequencing data. Amplicon sequencing provided a unique opportunity to filter the sequencing reads. The reads spanning the junctions with the correct forward primer, reverse primer, or both primer sequences at the ends of reads proved the known/novel sgmRNA existing in tested ARTIC-Illumina and ARTIC-Nanopore amplicon sequencing data (Supplementary Tables 1 and 2). For Illumina sequencing, the same junction on paired reads with at least one primer provided extra evidence for leader–TRS identification. Some reads were identified that did not have primer sequences, and these were likely to be erroneously mapped, from template sgmRNA or low-quality sequence. These were present at very low abundance compared to authentically mapped reads (Supplementary Tables 1 and 2). The Nanopore dRNAseq approach had the potential to generate full-length mRNA sequences. The polyA sequences and leader–TRS junctions in the reads can be good signals to prove the full-length sgmRNA in the test data (Supplementary Table 3). Currently, LeTRS is the only tool to consider paired-end Illumina data and primer pools and therefore is suited for interrogating paired-end Illumina data and providing data from amplicon sequencing information from either primer pool.

In terms of clinical samples (typically nasopharyngeal swabs), the presence of sgmRNAs will generally be due to the presence of infected cells. This has been seen as indicative of active viral RNA synthesis at the time of sampling [5, 33, 34], although these have also been postulated to be present through resistant structures after infection has finished [35]. Analysis of inoculum indicated that leader–TRS gene junctions could be identified (Supplementary Fig. 5) but that these were not in the same ratio as found in cells infected in culture (e.g., Fig. 2A, B, and C). Thus, if the abundance of leader–TRS gene junctions follows an expected pattern of the leader–TRS nucleoprotein gene junction being the most abundant followed by a general gradient in sequence data from nasopharyngeal samples, then this may be indicative of an active infection—and the presence of infected cells in a sample.

In the absence of a human challenge model, NHP models that closely resemble COVID-19 disease in humans can be used to study SARS-CoV-2 infection from a very defined initial exposure. RNA was sequenced from longitudinal nasopharyngeal samples from two NHP models, rhesus and cynomolgus macaques [27]. LeTRS was used to identify the abundance of the leader–TRS gene junctions in this data. The analysis indicated a phasic pattern of sgmRNA synthesis with a large drop-off after 8 or 9 dpi in both NHP models. This phasic pattern may be explained by an initial synchronous infection of respiratory epithelial cells followed by cell death. Released virus then goes on to infect new epithelial cells, with virus infection increasing exponentially in waves but becoming asynchronous. The decline in sgmRNA from 8 or 9 dpi overlaps with IgG seroconversion and humoral immunity in both species [27] and follows similar kinetics to serology profiles measured in patients with COVID-19.

The identification of sgmRNAs in nasopharyngeal samples and their kinetics has implications for nucleic acid–based diagnostics (many of which have three targets, one in the orf1a/b region and two that are shared between the genome and sgmRNAs—the nucleoprotein and the spike genes). The phasic nature of leader–TRS gene junctions in the longitudinal samples, and by implication sgmRNAs, and overt abundance of the leader–TRS nucleoprotein gene junction found in many of the human samples suggest that it may not be possible to precisely identify where in infection an individual is based on the abundance of sgmRNAs. Likewise, assuming equivalency between the targets, if the nucleoprotein target is found to be more abundant than the spike target than the genomic target, then this would suggest infected cells are present in the sample. Decreases in Ct values associated with emerging variants could equally be explained by sloughed cells being present in a nasopharyngeal sample as well as by increases in the amount of virions/viral load. Therefore, we would caution that a decrease in Ct associated with quantitative reverse transcriptase PCR-based assays may not just be reflective of higher viral loads but also may be indicative of more infected cells being present. These possibilities may be resolved by considering the relative ratios of sgmRNAs identified.

## METHODS

### Data input

LeTRS was designed to analyze FASTQ files derived from Illumina paired-end or Nanopore sequencing data derived from a SARS-CoV-2 amplicon protocol, or standard Nanopore SARS-CoV-2 dRNAseq data (Fig. 1). The Illumina/Nanopore FASTQ sequencing data were cleaned to remove adapters and low-quality reads before input. Sequencing data derived from other sequencing modes or platforms can also be analyzed by LeTRS via input of a BAM file produced by a custom splicing alignment method with a SARS-CoV-2 genome (NC_045 512.2) as a reference (Fig. 1). This can also be rapidly adapted for other coronaviruses.

### Library preparations and sequencing

We sequenced the 15 samples from human patients with Nanopore. Total RNA was isolated using a QIAamp Viral RNA Mini Kit (Qiagen, Manchester, UK) by a spin-column procedure according to the manufacturer's instructions. Clinical samples were extracted with Trizol LS as described [4]. All RNA samples were treated with Turbo DNase (Invitrogen, Carlsbad, USA"). SuperScript IV (Invitrogen) was used to generate single-strand cDNA using random primer mix (NEB, Hitchin, UK). ARTIC V3 PCR amplicons from the single-strand cDNA were generated following the Nanopore Protocol of PCR tiling of SARS-CoV-2 virus (Version: PTC_9096_v109_revL_06Feb2020). Amplicons generated by ARTIC PCR were purified and normalized to 200 fmol before DNA end preparation and barcode and adapter ligation. Library was loaded onto a FLO-MIN106 flow cell and sequencing reads were called with Guppy using the high-accuracy calling parameters.

The NHP samples and their inoculum, as well as our laboratory experiments conducted in cells, were sequenced with Illumina (San Diego, CA, USA). The amplicon products for Illumina sequencing were prepared as per the Nanopore sequencing above and then used in Illumina NEBNext Ultra II DNA Library preparation. Following four cycles of amplification, the library was purified using Ampure XP beads and quantified using Qubit and the size distribution assessed using the Fragment analyzer. Finally, the ARTIC library was sequenced on the Illumina NovaSeq 6000 platform (RRID:SCR_016387) following the standard workflow. The generated raw FastQ files (2 × 250 bp) were trimmed to remove Illumina adapter sequences using Cutadapt v1.2.1 (RRID:SCR_011841) [36]. The option “−O 3” was set, so that the 3′ end of any reads that matched the adapter sequence with greater than 3 bp was trimmed off. The reads were further trimmed to remove low-quality bases, using Sickle v1.200 [37], with a minimum window quality score of 20. After trimming, reads shorter than 10 bp were removed.

The LeTRS was also tested with a combined Nanopore-ARTIC v3 amplicon data set of seven published viral cell culture samples (barcode01–barcode07) [30] and a data set from a published direct RNA Nanopore sequencing analysis of Vero cells infected with SARS-CoV-2 or an uninfected negative control [2].

### Sequencing data alignment and basic filtering

LeTRS controlled Hisat2 v2.1.0 (RRID:SCR_015530) [38] to map the paired-end Illumina reads against the SARS-CoV-2 reference genome (NC_045 512.2) with the default setting and Minimap2 v2.1 [18] to align the Nanopore cDNA reads and dRNAseq reads on the viral genome using Minimap2 with “–ax splice” and “–ax splice -uf -k14” parameters, respectively. LeTRS provided 10 known leader–TRS junctions to improve alignment accuracy by using the “–known-splicesite-infile” function in Hisat2 and “–junc-bed” function in Minimap2, but this application could be optionally switched off by users. In order to remove low mapping quality and mis-mapped reads before searching the leader–TRS junction sites, LeTRS used Samtools v1.9 (RRID:SCR_002105) [39] to have basic filtering for the reads in the output Sam/Bam files according to their alignment states as shown (Table 2–basic filtering).

### Searching the leader–TRS motifs

After the mapping and basic filtering step, LeTRS searched aligned reads spanning the leader–TRS junctions in the SARS-CoV-2 reference genome (Supplementary Fig. 1). For the known leader–TRS junctions, LeTRS searched the reads, including the leader–TRS junctions within a given interval around the known leader and TRS junctions sites. The leader break site interval is ±10 nts, and the TRS breaking sites interval is –20 nts to the 1 nt before the first known initiation codon (AUG) in the default setting (the intervals can be changed to custom values to investigate heterogeneity). LeTRS then reported a peak count that was the number of reads carrying the most common leader–TRS junctions within the given leader and TRS breaking site intervals, as well as a cluster count that was the number of all reads carrying leader–TRS junctions within the given leader and TRS breaking site intervals ( Supplementary Tables 1–9 ). LeTRS also searched the junctions out of the given intervals (the genomic position of leader breaking site <80) and reported the number of reads (>10 by default) with novel leader–TRS junctions. These numbers of read counts were also reported by number of reads in 1,000,000 as normalization. The read including the known and novel leader–TRS junctions could be optionally outputted in FastA format. Based on the identified known and novel leader–TRS junctions, LeTRS could report on the leader (20 nucleotides at the 3′ end) and TRS sequences, translate the first predicted orf of the sgmRNA, and find the conserved ACGAAC sequences in the TRS (Supplementary Tables 1–9).

### Advance filtering

Based on the alignment possibilities illustrated in Fig. 2 and discussed, LeTRS further filters the identified reads with known and novel leader–TRS junctions. This step is named advance filtering and can only be applied when the input data are from Illumina paired-end reads, Nanopore cDNA reads, or Nanopore RNA reads (Table 2). If a BAM file is used as input data, the advanced filtering step would be automatically skipped (Table 2). The number of reads, including the known and novel leader–TRS junctions, and the number of reads filtered with corresponding advance filtering criteria were outputted into two tables in tab format (Tables 1–9).

**Table 2:** The criteria of basic and advanced filtering for four different types of input data for LeTRS

	Output filters​	Illumina paired-end amplicon reads	Nanopore amplicon reads	Nanopore dRNAseq reads	Bam
Basic filtering​	Mapping quality (MAPQ) >10​	● ​	● ​	● ​	● ​
	Read only one splicing junction ​	● ​	● ​	● ​	● ​
	Primary alignment only​	● ​	● ​	● ​	● ​
	No supplementary alignment​	● ​	● ​	● ​	● ​
	Read mapped in pair​	● ​	​	​	​
	No read reverse strand​	​	​	● ​	​
Advance filtering​	Read alignment 5' end includes forward primer​	● ​	● ​	​	​
	Read alignment 3' end includes reverse primer​	● ​	● ​	​	​
	Read alignment 5' end includes forward primer and 3' end includes reverse primer​	● ​	● ​	​	​
	Paired read including at least one primer in each has same leader–TRS junction in alignments​	● ​	● ​	​	​
	Read alignment 3' with >1 ployA​	​	● ​	● ​	​
	Read alignment 3' with >5 ployA​	​	● ​	● ​	​

### Leader–TRS junction plotting

LeTRS-plot was developed as an automatic plotting tool that interfaces with the R package ggplot2 v3.3.3 to view the leader–TRS junctions in the tables generated by LeTRS (Figs. 3–5). The plot shows peak count, filtered peak count, normalized peak count, and normalized filtered peak count for known leader–TRS junctions, novel junction counts, filtered novel junction count, normalized novel junction count, and filtered normalized novel junction for novel leader–TRS junctions.

### Simulation of Illumina and Nanopore reads

To assess the performance of LeTRS and other tools, simulated Illumina reads were generated using ART (v2.5.8) [40] and Nanopore reads were generated using NanoSim (v2.6.0, RRID:SCR_018243) [41]. The real reads generated by the ARTIC-Nanopore approach, ARTIC-Illumina approach, and Nanopore dRNAseq approach for the hACE2-A549 cells infected with SARS-CoV-2 were used to create custom Illumina and Nanopore read quality/error profiles with ART and NanoSim. Illumina paired reads (2 × 250 bp) and Nanopore cDNA-1D reads for both ARTIC and sgmRNA amplicons were simulated at 50,000× coverage for each amplicon and 2,000,000 reads in total, respectively. Nanopore dRNAseq reads (2,000,000) of the sgmRNA and viral genome were generated using transcriptome mode.

### RNA modifications

Total RNA extracted from cultured cells at 6, 12, and 24 hours were collected for Oxford Nanopore direct RNA sequence. LeTRS was then run with a parameter of “extractfasta” to extract subgenomic mRNA reads in sequenced samples. The fast5 files that correspond to the extracted subgenomic mRNAs reads were withdrawn using fast5_subset in Oxford Nanopore ont_fast5_api package (v0.3.2, [42]). The re-squiggle algorithm in Tombo analysis pipelines (v1.5.1, [43]) defines a new assignment from raw signals to reference sequence with the “–num-most-common-errors 5” option. The resquiggled raw signals were further processed using “detect_modifications alternative_model” functions in Tombo by setting “–rna and –alternate-bases 5mC” to identify 5mC and “predict_sites” in Nanom6A package (v2021_10_22) [44] with default setting to identify 6mA in the subgenomic mRNAs reads.

## Ethics approval and consent to participate

All experimental work on NHPs was conducted under the authority of a UK Home Office approved project license (PDC57C033) that had been subject to local ethical review at PHE Porton Down by the Animal Welfare and Ethical Review Body (AWERB) and approved as required by the Home Office Animals (Scientific Procedures) Act 1986 and the full ethics and NHP model are described.

## Additional Files

**Supplementary Figure 1**. Bioinformatics pipeline for the identification of leader–TRS junctions in sequencing data from SARS-CoV-2–infected material with LeTRS. This can be rapidly adapted for other coronaviruses such as MERS-CoV and any newly emerged coronavirus. LeTRS can work from Nanopore or Illumina amplicon data or more unbiased approaches such as direct RNA sequencing, metagenomic sequencing, or Illumina sequencing by using a BAM file.

**Supplementary Figure 2**. Novel (leader-dependent noncanonical) fusions (count ≥2) found in the cell culture test sample sequenced by (A) ARTIC-Nanopore, (B) ARTIC-Illumina, and (C) Nanopore dRNAseq approaches; leader-independent long-distance (>5,000 nt) fusions (count ≥2) found in the cell culture test sample sequenced by (D) ARTIC-Nanopore, (E) ARTIC-Illumina, and (F) Nanopore dRNAseq approaches; leader-independent local joining yielding a deletion between proximal site (20–5,000 nt distance) fusions (count ≥2) found in the cell culture test sample sequenced by (G) ARTIC-Nanopore, (H) ARTIC-Illumina, and (I) Nanopore dRNAseq approaches. The data correspond to that shown Supplementary Tables 1, 2, and 3.

**Supplementary Figure 3**. Comparison of different tools and LeTRS to evaluate sequencing data to identify the unique sequencing features of SARS-CoV-2 sgmRNAs. Number of reads were evaluated by LeTRS (all peak count), SARS-COV-2–leader, SuPER, or periscope (High Quality [HQ] count) with the cell culture test sample sequenced by (A) ARTIC-Nanopore, (B) ARTIC-Illumina, and (C) Nanopore dRNAseq approaches. (D) Ratio of sgmRNAs (S:orf3:E:M:orf6:orf7a:orf7b:orf8:N:orf10) identified by LeTRS (all peak count), SARS-COV-2-leader, SuPER, or periscope (HQ count) with the cell culture test sample sequenced by ARTIC-Nanopore, ARTIC-Illumina, and Nanopore dRNAseq approaches. The data are corresponded to that shown in Supplementary Tables 1, 2, and 3.

**Supplementary Figure 4**. Comparison of the ratio of reads in amplicon sequencing approaches based on the ARTIC approach with the forward primer only, reads with reverse primer only, and reads with both primers in sgmRNAs to the overall ratio of reads with the forward primer only, reads with reverse primer only, and reads with both primers in all reads amplified by pool 1 primers, pool 2 primers, and both pools of primers for the cell culture test sample sequenced by (A) ARTIC-Nanopore and (B) ARTIC-Illumina approaches.

**Supplementary Figure 5**. Raw (A, C) and normalized (B, D) canonical (upper) and novel (lower) leader–TRS gene junction count in RNA purified from the inoculum of SARS-CoV-2 used to infect either the cynomolgus or rhesus macaques. The RNA was sequenced by the ARTIC-Illumina method (Supplementary Table 6). Standard deviation of a binomial distribution was calculated to provide error bars.

**Supplementary Figure 6**. Novel leader–TRS gene junctions (count >10) identified in RNA purified from nasopharyngeal swabs taken daily from cynomolgus macaques infected with SARS-CoV-2 (Supplementary Table 7). The number before “-Day” indicates the group of cynomolgus macaques. Standard deviation of a binomial distribution was calculated to provide error bars.

**Supplementary Figure 7**. Novel leader–TRS gene junctions (count >10) identified in RNA purified from nasopharyngeal swabs taken daily from rhesus macaques (Supplementary Table 7). The number before “-Day” indicates the group of cynomolgus macaques. Standard deviation of a binomial distribution was calculated to provide error bars.

**Supplementary Figure 8**. Comparison of the fraction of 6mA modification (right-hand y-axis) of each site in sgmRNA at 6, 12, and 24 hours postinfection using direct RNA sequencing from RNA purified from SARS-CoV-2–infected cells. Only the sites with modification in at least one of the 6 hpi, 12 hpi, and 24 hpi were analyzed.

**Supplementary Figure 9**. Comparison of the fraction of 5mC modification (right-hand y-axis) of each site in sgmRNA at 6, 12, and 24 hours postinfection using direct RNA sequencing from RNA purified from SARS-CoV-2–infected cells. Only the sites with modification in at least one of the 6 hpi, 12 hpi, and 24 hpi were analyzed.

**Supplementary Table 1**. The LeTRS output tables for known sgmRNA, details of known sgmRNA, novel sgmRNA (count ≥2), details of novel sgmRNA, and leader-independent long-distance and local fusions (count ≥2) evaluated in the cell culture test sample sequenced by the ARTIC-Nanopore approach.

**Supplementary Table 2**. The LeTRS output tables for known sgmRNA, details of known sgmRNA, novel sgmRNA (count ≥2), details of novel sgmRNA, and leader-independent long-distance and local fusions (count ≥2) evaluated in the cell culture test sample sequenced by the ARTIC-Illumina approach.

**Supplementary Table 3**. The LeTRS output tables for known sgmRNA, details of known sgmRNA, novel sgmRNA (count ≥2), details of novel sgmRNA, and leader-independent long-distance and local fusions (count ≥2) evaluated in the cell culture test sample sequenced by the Nanopore dRNAseq approach.

**Supplementary Table 4**. The LeTRS output table for known sgmRNA evaluated by primers of pools 1 and 2 in the cell culture test sample sequenced by the ARTIC-Nanopore approach.

**Supplementary Table 5**. The LeTRS output tables for known sgmRNA evaluated by primers of pools 1 and 2 in the cell culture test sample sequenced by the ARTIC-Illumina approach.

**Supplementary Table 6**. The LeTRS output tables for known sgmRNA and details of known sgmRNA with pool 1 primers and novel sgmRNA (count >10) and details of novel sgmRNA with both pools’ primers in the infecting SARS-CoV-2 inoculum source used for the NHP study, sequenced by the ARTIC-Illumina method.

**Supplementary Table 7**. The LeTRS output tables for known sgmRNA and details of known sgmRNA with pool 1 primers and novel sgmRNA (count >10) and details of novel sgmRNA with both pools’ primers in longitudinal nasopharyngeal samples taken from two nonhuman primate models (cynomolgus and rhesus macaques) of SARS-CoV-2 in groups. SARS-CoV-2 was amplified using the ARTIC approach and sequenced by Illumina. The data are organized into groups of animals for the cynomolgus macaque groups 1 and 2 that were with “–1” and “–2” in the Excel sheets.

**Supplementary Table 8**. The LeTRS output tables for known sgmRNA and details of known sgmRNA in pool 1 and novel sgmRNA (count >10) and details of novel sgmRNA with both pools’ primers from 15 human patients sequenced with ARTIC-Illumina.

**Supplementary Table 9**. The LeTRS output tables for known sgmRNA and details of known sgmRNA in pool 1 from 15 human patients sequenced with ARTIC-Nanopore.

**Supplementary Table 10**. The spreadsheet for the 15 human patients sequenced with the ARTIC-Nanopore detailed in Supplementary Table 9.

**Supplementary Table 11**. Reanalysis of reads for known sgmRNAs in the (NCBI accession No. PRJNA636225) [25].

**Supplementary Table 12**. Summary of normalized count, number of modification sites, and average modification fraction in each sgmRNA at 6 hpi, 12 hpi, and 24 hpi.

**Supplementary Table 13**. Evaluation of the difference of modification by the paired samples one-sided Wilcoxon test to calculate *P* value by treating the same nucleotides between any two time points as paired data.

**Supplementary Table 14**. The LeTRS output table for novel sgmRNA (count >10) and details of novel sgmRNA with both primer pools from VeroE6 cells infected in culture with SARS-CoV-2 (SCV2-006) sequenced by ARTIC-Illumina primers. This sample is different from the one in Supplementary Table 2.

**Supplementary Table 15**. Novel leader–TRS junctions centered on the known gene open reading frame but out of the search interval in the analysis of cell culture, nonhuman primate, and human sequencing data.

giac045_GIGA-D-21-00142_Original_Submission

giac045_GIGA-D-21-00142_Revision_1

giac045_GIGA-D-21-00142_Revision_2

giac045_Response_to_Reviewer_Comments_Original_Submission

giac045_Response_to_Reviewer_Comments_Revision_1

giac045_Reviewer_1_Report_Original_Submission Chan Zhou -- 6/17/2021 Reviewed

giac045_Reviewer_1_Report_Revision_1 Chan Zhou -- 12/13/2021 Reviewed

giac045_Reviewer_2_Report_Original_Submission Lachlan Coin -- 6/23/2021 Reviewed

giac045_Reviewer_2_Report_Revision_1 Lachlan Coin -- 1/23/2022 Reviewed

giac045_Supplemental_Files

## Abbreviations

5mC: 5-methylcytosine methylation; 6mA: N6-methyladenosine; COVID-19: cornavirus disease 2019; dpi: days postinfection; dRNAseq: direct RNA sequencing; hpi: hours postinfection; NHP: nonhuman primate; PCR: polymerase chain reaction; SARS-CoV-2: severe acute respiratory syndrome coronavirus 2; sgmRNA: subgenomic mRNA; TRS: transcriptional regulatory sequence.

## Consent for publication

Not applicable

## Data Availability

Illumina and Nanopore test data sets are available under NCBI PRJNA699398. Snapshots of the code are available in the *GigaScience* GigaDB repository [45].

## Availability and requirements

- Project name: LeTRS
- Project home page: [46]
- Operating system(s): Platform independent
- Programming language: Perl
- Other requirements: samtools(> = 1.11), hisat2( = 2.1.0), minimap2( = 2.17), portcullis(> = 1.1.2)
- License: Apache 2.0
- RRID:SCR_022138

## Competing interests

The authors declare that they have no competing interests

## Funding

This work was funded by a US Food and Drug Administration (FDA) Medical Countermeasures Initiative contract (75F40120C00085) awarded to JAH. The article reflects the views of the authors and does not represent the views or policies of the FDA. This work was also supported by the MRC (MR/W005611/1) G2P-UK: A national virology consortium to address phenotypic consequences of SARS-CoV-2 genomic variation (JAH as a co-investigator). JAH was also funded by the Centre of Excellence in Infectious Diseases Research (CEIDR) and the Alder Hey Charity. The nonhuman primate work was funded by the Coalition of Epidemic Preparedness Innovations (CEPI) and the Medical Research Council Project CV220-060, Development of an NHP model of infection and ADE with COVID-19 (SARS-CoV-2) both awarded to MWC. The ISARIC4C sample collection and sequencing in this study was supported by grants from the Medical Research Council (grant MC_PC_19059), the National Institute for Health Research (NIHR; award CO-CIN-01), the Medical Research Council (MRC; grant MC_PC_19059), and the NIHR Health Protection Research Unit (HPRU) in Emerging and Zoonotic Infections at University of Liverpool in partnership with Public Health England (PHE), in collaboration with Liverpool School of Tropical Medicine and the University of Oxford (award 200907), NIHR HPRU in Respiratory Infections at Imperial College London with PHE (award 200927), Wellcome Trust and Department for International Development (DID; 215091/Z/18/Z), the Bill and Melinda Gates Foundation (OPP1209135), Liverpool Experimental Cancer Medicine Centre (grant reference C18616/A25153), and NIHR Biomedical Research Centre at Imperial College London (IS-BRC-1215–20013). PJMO is supported by a NIHR senior investigator award (201385). The views expressed are those of the authors and not necessarily those of the Department of Health and Social Care, DID, NIHR, MRC, Wellcome Trust, or PHE. The funders had no role in the study design; in the collection, analysis, and interpretation of data; in the writing of the report; or in the decision to submit the article for publication.

## Authors' contributions

XD developed the LeTRS software and performed the informatics analysis. XD, AD, and JAH analyzed the data. JS, JT, and MWC coordinated the NHP work and sample processing. RP-R, JPS, HG, TP, and NR were involved in sequencing and informatics analysis of the NHP samples with DAM. AD oversaw sequencing of the human clinical samples with EV and CN for the COG-UK data. RP-R and JAH oversaw sequencing of samples under the auspices of ISARIC-4C with clinical samples collected and managed by JKB, LT, MGS, and PJMO. JAH and MWC initiated and led the study and wrote the manuscript with XD, RP-R, and AD, with other authors involved in editing the final version. Conflict of Interest: None declared.
