# Supplementary material for: Analysis of SARS-CoV-2 known and novel subgenomic mRNAs in cell culture, animal model, and clinical samples using LeTRS, a bioinformatic tool to identify unique sequence identifiers
Source: Gigascience. 2022 May 26;11:giac045. doi: 10.1093/gigascience/giac045 (PMC9154083; doi:10.1093/gigascience/giac045)
Supplement: giac045_GIGA-D-21-00142_Original_Submission [file giac045_giga-d-21-00142_original_submission.pdf]

GigaScience

Identification and quantification of SARS-CoV-2 leader subgenomic mRNA gene junctions in nasopharyngeal samples shows phasic transcription in animal models of COVID-19 and dysregulation at later time points that can also be identified in humans

--Manuscript Draft--

**Manuscript Number:**	GIGA-D-21-00142
**Full Title:**	Identification and quantification of SARS-CoV-2 leader subgenomic mRNA gene junctions in nasopharyngeal samples shows phasic transcription in animal models of COVID-19 and dysregulation at later time points that can also be identified in humans
**Article Type:**	Research
**Funding Information:**	U.S. Food and Drug Administration Medical Countermeasures (75F40120C00085) — Prof. Julian A. Hiscox<br>MRC ((MR/W005611/1) G2P-UK) — Prof. Julian A. Hiscox
**Abstract:**	SARS-CoV-2 has a complex strategy for the transcription of viral subgenomic mRNAs (sgmRNAs),<br>which are targets for nucleic acid diagnostics. Each of these sgRNAs has a unique 5' sequence,<br>the leader-transcriptional regulatory sequence gene junction (leader-TRS-junction), that can be<br>identified using sequencing. High resolution sequencing has been used to investigate the biology<br>of SARS-CoV-2 and the host response in cell culture models and from clinical samples. LeTRS, a<br>bioinformatics tool, was developed to identify leader-TRS-junctions and be used as a proxy to<br>quantify sgmRNAs for understanding virus biology. This was tested on published datasets and<br>clinical samples from patients and longitudinal samples from animal models with COVID-19.<br>LeTRS identified known leader-TRS-junctions and identified novel species that were common<br>across different species. The data indicated multi-phasic abundance of sgmRNAs in two different<br>animal models, with spikes in sgmRNA abundance reflected in human samples, and therefore has<br>implications for transmission models and nucleic acid-based diagnostics.
**Corresponding Author:**	Julian Hiscox<br>University of Liverpool<br>Liverpool, UNITED KINGDOM
**Corresponding Author Secondary Information:**	
**Corresponding Author's Institution:**	University of Liverpool
**Corresponding Author's Secondary Institution:**	
**First Author:**	Xiaofeng Dong
**First Author Secondary Information:**	
**Order of Authors:**	Xiaofeng Dong<br>Rebekah Penrice-Randal<br>Hannah Goldswain<br>Tessa Prince

Powered by Editorial Manager® and ProduXion Manager® from Aries Systems Corporation

	Nadine Randle
	Francisco J Salguero
	Julia Tree
	Ecaterina Vamos
	Charlotte Nelson
	James P. Stewart
	Malcolm G. Semple
	John Kenneth Baillie
	Peter J. Openshaw
	Lance Turtle
	David A. Matthews
	Miles W. Carroll
	Alistair C. Darby
	Julian A. Hiscox
**Order of Authors Secondary Information:**	
**Additional Information:**	
**Question**	**Response**
Are you submitting this manuscript to a special series or article collection?	No
**Experimental design and statistics**<br><br>Full details of the experimental design and statistical methods used should be given in the Methods section, as detailed in our Minimum Standards Reporting Checklist. Information essential to interpreting the data presented should be made available in the figure legends.<br><br>Have you included all the information requested in your manuscript?	Yes
**Resources**<br><br>A description of all resources used, including antibodies, cell lines, animals and software tools, with enough information to allow them to be uniquely identified, should be included in the Methods section. Authors are strongly encouraged to cite Research Resource Identifiers (RRIDs) for antibodies, model organisms and tools, where possible.	Yes

Have you included the information requested as detailed in our Minimum Standards Reporting Checklist?	
**Availability of data and materials**<br><br>All datasets and code on which the conclusions of the paper rely must be either included in your submission or deposited in publicly available repositories (where available and ethically appropriate), referencing such data using a unique identifier in the references and in the "Availability of Data and Materials" section of your manuscript.<br><br>Have you have met the above requirement as detailed in our Minimum Standards Reporting Checklist?	Yes

Powered by Editorial Manager® and ProduXion Manager® from Aries Systems Corporation

Main text of manuscript

Click here to access/download;Manuscript;Manuscript_Text_File.pdf

Identification and quantification of SARS-CoV-2 leader subgenomic mRNA gene junctions in nasopharyngeal samples shows phasic transcription in animal models of COVID-19 and dysregulation at later time points that can also be identified in humans

Xiaofeng Dong[1], Rebekah Penrice-Randal[1], Hannah Goldswain[1], Tessa Prince[1], Nadine Randle[1], Francisco J. Salguero[2], Julia Tree[2], Ecaterina Vamos[1], Charlotte Nelson[1], James P. Stewart[1], Malcolm G. Semple[1,3], J. Kenneth Baillie[4], Peter J. M. Openshaw[5], Lance Turtle[1], David A. Matthews[6], Miles W. Carroll[2], Alistair C. Darby[1] and Julian A. Hiscox[1,3,7].

[1]Institute of Infection, Veterinary and Ecological Sciences, University of Liverpool, UK.

[2]Public Health England, Salisbury, UK.

[3]NIHR Health Protection Research Unit in Emerging and Zoonotic Infections, Liverpool, UK.

[4]The Roslin Institute, University of Edinburgh, UK.

[5]National Heart and Lung Institute, Imperial College London, UK.

[6]University of Bristol, UK.

[7]Infectious Diseases Horizontal Technology Centre (ID HTC), A*STAR, Singapore.

Corresponding author: julian.hiscox@liverpool.ac.uk

Abstract

SARS-CoV-2 has a complex strategy for the transcription of viral subgenomic mRNAs (sgmRNAs), which are targets for nucleic acid diagnostics. Each of these sgRNAs has a unique 5' sequence, the leader-transcriptional regulatory sequence gene junction (leader-TRS-junction), that can be identified using sequencing. High resolution sequencing has been used to investigate the biology of SARS-CoV-2 and the host response in cell culture models and from clinical samples. LeTRS, a bioinformatics tool, was developed to identify leader-TRS-junctions and be used as a proxy to quantify sgmRNAs for understanding virus biology. This was tested on published datasets and clinical samples from patients and longitudinal samples from animal models with COVID-19. LeTRS identified known leader-TRS-junctions and identified novel species that were common across different species. The data indicated multi-phasic abundance of sgmRNAs in two different animal models, with spikes in sgmRNA abundance reflected in human samples, and therefore has implications for transmission models and nucleic acid-based diagnostics.

Importance

When infecting cells SARS-CoV-2 not only replicates its genome but also makes molecules called subgenomic mRNAs (sgmRNAs) that are used to make many of the viral proteins, including the spike glycoprotein. The sgmRNAs can only be found in infected cells, and therefore their presence and ratio in a clinical sample is indicative that viral RNA synthesis has occurred, and infected cells are present. Many of the sgmRNAs are targets for diagnostic assays. We have developed a rapid informatics methodology to identify these unique molecules from sequencing data. We used this to follow their pattern of abundance in nasopharyngeal samples taken from non-human primate models and clinical samples from humans. The data indicates that SARS-CoV-2 RNA synthesis

(and by inference infection) may occur in waves and this has implications for diagnostics and modelling of disease spread.

Introduction

Various sequencing approaches are used to characterise SARS-CoV-2 RNA synthesis in cell culture[1, 2], ex vivo models[3] and clinical samples. This can include nasopharyngeal swabs from patients with COVID-19[4] to post-mortem samples from patients who died of severe disease[5]. Bioinformatic interrogation of this data can provide critical information on the biology of the virus. SARS-CoV-2 genomes are message sense, and the 5' two thirds of the genome is translated and proteolytically cleaved into a variety of functional subunits, many of which are involved in the synthesis of viral RNA[6]. The remaining one third of the genome is expressed through a nested set of subgenomic mRNAs (sgmRNAs). These have common 5' and 3' ends with the coronavirus genome, including a leader sequence. Many studies have shown that the sgmRNA located towards the 3' end of the genome, which encodes the nucleoprotein, generally has a higher abundance than those located immediately after the 1a/b region and the genome itself[7, 8]. However, there is not necessarily a precise transcription gradient of the sgmRNAs. The 5' leader sequence on the sgmRNAs is immediately abutted to a short sequence called a transcriptional regulatory sequence (TRS) that is involved in the control of sgmRNA synthesis[9, 10]. These TRSs are located along the genome and are proximal to the start codons of the open reading frames[11]. In the negative sense the TRSs are complementary to a short portion of the genomic leader sequence. The TRS is composed of a short core motif that is conserved and flanking sequences[9, 10, 12]. For SARS-CoV-2 the core motif is ACGAAC.

The prevailing thought is that synthesis of sgmRNAs involves a discontinuous step during negative strand synthesis[13, 14]. A natural consequence of this is recombination resulting in insertions and

deletions in the viral genome and the formation of defective viral RNAs. Thus, the identification of the leader/sgmRNA complexes by sequencing provides information on the abundance of the sgmRNAs and evidence that transcription has occurred in the tissue being analysed. In terms of clinical samples, if infected cells are present, then leader/sgmRNA 'fusion' sequence can be identified, and inferences made about active viral RNA synthesis from the relative abundance of the sgmRNAs. In the absence of human challenge models, the kinetics of virus infection are unknown, and most studies will begin with detectable viral RNA on presentation of the patient with clinical symptoms. In general, models of infection of humans with SARS-CoV-2 assume an exponential increase in viral RNA synthesis followed by a decrease as antibody levels increase[15].

In order to investigate the presence of SARS-CoV-2 sgmRNAs in clinical (and other) samples, a bioinformatics tool (LeTRS), was developed to analyse sequencing data from SARS-CoV-2 infections by identifying the unique leader-TRS gene junction site for each sgmRNA. The utility of this tool was demonstrated on cells infected in culture, nasopharyngeal samples from human infections and longitudinal analysis of nasopharyngeal samples from two non-human primate models for COVID-19. The results have implications for diagnostics and disease modelling.

Results

A tool, LeTRS (named after the leader-TRS fusion site), was developed to detect and quantify defined leader gene junctions of SARS-CoV-2 (and other coronaviruses) from multiple types of sequencing data. This was used to investigate SARS-CoV-2 sgmRNA synthesis in humans and non-human primate animal models. LeTRS was developed using the Perl programming language, including a main program for the identification of sgmRNAs and a script for plotting graphs of the results. The tool accepts FastQ files derived from Illumina paired-end or Oxford Nanopore amplicon cDNA, Nanopore direct RNA sequencing, or BAM files produced by a splicing alignment method with a SARS-CoV-2 genome (Supplementary Figure 1). (Note that SARS-CoV-2 sgmRNAs are not formed by splicing, but this is the apparent observation from sequencing data as a result of the discontinuous nature of transcription). By default, LeTRS analyses SARS-CoV-2 sequence data by using 10 known leader-TRS junctions and an NCBI reference genome (NC_045512.2). However, given the potential heterogeneity in the leader-TRS region and potential novel sgmRNAs, the user can also provide customize leader-TRS junctions and SARS-CoV-2 variants as a reference. The tool was designed to investigate very large data sets that are produced during sequencing of multiple samples. As there is some heterogeneity in the leader-TRS sites, LeTRS was also designed to search the leader-TRS junction in a given interval, report 20 nucleotides at the 3' end of the leader sequence, the TRS and translated first orf of the sgmRNA, and find the conserved ACGAAC sequences in the TRS.

Combinations of read alignments with the leader-TRS junction that are considered for identifying leader-TRS junction sites

Various approaches have been used to sequence the SARS-CoV-2 genome and in most cases, this would also include any sgmRNAs as they are 3’ co-terminal and share common sequence extending from the 3’ end. Methods such as ARTIC[16] and RSLA[4] use primer sets to generate overlapping amplicons that span the entire genome, and also amplify sgmRNA. Included is a primer to the leader sequence, so that the unique 5’ end of these moieties are also sequenced. Primer sets of ARTIC and RSLA are generally pooled. Unbiased sequencing can also be used in methodologies to identify SARS-CoV-2 sequence. Data in the GISAID database have been generated by Oxford Nanopore (minority) or Illumina (majority) based approaches. These can give different types of sequencing reads derived from the sgmRNAs that can be mapped back on the reference SARS-CoV-2 genome by splicing alignment (Figure 1A). For example, there are a number of different types of reads that can be derived from mapping Illumina-based amplicon sequencing onto the reference viral genome (Figure 1B and 1C). During the PCR stage, the extension time allows the leader-TRS region on the sgmRNAs to be PCR-amplified by the forward primer and the reverse primer before and after leader-TRS junction in different primer sets, respectively. Both of these forward and reverse and primers would be detected at both ends of each paired read (Figure 1B pink lines) if the amplicon had a length shorter than the Illumina read length (usually 100-250 nts). If the amplicon was longer than the Illumina read length, primer sequence would be only found at one end of each paired read (Figure 1B green lines). The extension stage could also proceed with a single primer using cDNA from the sgmRNA as template. This type of PCR has a very low amplification efficiency, but theoretically could also generate the same Illumina paired end reads with just one primer sequence at one end (Figure 1C). These paired end reads could include the full length of the leader sequence but might not reach the 3’

end of the sgmRNA, because of the limitation of Illumina sequencing length and extension time (Figure 1C). Also, unless there are cryptic TRSs, all sgmRNAs would be expected to be larger than the Illumina sequencing length.

In contrast, the different types of read alignment in the Nanopore based cDNA sequencing are simpler to assign. The longer reads that tend to be generated by Nanopore sequencing (depending on optimisation) enable the capture of full-length sequences of all amplicons. Provided the leader sequence is included as a forward primer most of the reads spanning the leader-TRS junction would contain the forward and reverse primer sequences at both ends (Figure 1D pink lines). If the extension time allowed, single primer PCR amplification could take the Nanopore cDNA sequencing reads to both the 3' and 5' ends of the sgmRNAs, and these types of reads would only have a primer sequence at one end (Figure 2D brown lines). In the Nanopore direct RNA sequencing approach, the full length sgmRNA could be sequenced and mapped entirely on the leader and TRS-orf regions (Figure 1E).

Evaluation of LeTRS on SARS-CoV-2 infection in cell culture.

In order to assess the ability of LeTRS to identify the leader-TRS junctions from sequencing information, the tool was first evaluated on sequence data obtained from published SARS-CoV-2 infections in cell culture and our laboratory experiments conducted for this study. First, published data was used from sequencing viral RNA at 72 hrs post-infection in a cell culture model[16]. SARS-CoV-2 was sequenced using Nanopore from an amplicon-based approach (ARTIC)[16] (Figure 2A, Table 1 and 2, Supplementary Tables 1 and 2). All of the major known leader-TRS gene junctions

were identified. Interestingly, the nucleoprotein gene leader-TRS was approximately the same abundance as the membrane leader-TRS, whereas the other leader-TRSs were much lower. Two potential novel leader-TRS gene junctions were identified at positions 21,055 and 28,249 (Figure 2A, Table 2, Supplementary Table 2). The former is within the orf1b region and the latter within orf8. Second, data was analysed from a published experiment of cells infected (Figure 2B, Table 3 and 4, Supplementary Tables 3 and 4) and control sample (Tables 5 and 6) in culture using a direct RNA sequencing approach[2]. Analysis demonstrated a more expected pattern of abundance of the leader-TRS gene junctions (Figure 2B, Table 3 and Supplementary Table 3). The leader-TRS nucleoprotein gene junction was most abundant, and in general, the patten of abundance of the leader-TRS gene junctions for the major structural proteins followed the order of the gene junction along the genome. Novel low abundance leader-TRS gene junctions were also identified. One of these low abundance leader-TRSs gene junctions was also common to those found by the ARTIC amplicon analysis (Figure 2A and B, Table 2 and 4, Supplementary Table 2 and 4). Third, LeTRS was evaluated on sequencing data obtained from VeroE6 cells infected in culture with SARS-CoV-2 (SCV2-006). Here viral RNA, that had been prepared at 24 hrs post-infection, was amplified using the ARTIC approach and sequenced by Illumina (Figure 2C, Table 7 and 8, Supplementary Tables 5 and 6). As would be predicted the major leader-TRS gene junctions were identified, with the nucleoprotein one being the most abundant. Novel potential leader-gene junctions were also identified, including three that were greater in abundance than the other leader-gene junction. Some of the novel leader-TRS gene junctions from these three cell culture data sets shared the same first orf with the known sgmRNAs (Supplementary Tables 2, 4 and 6).

Comparison with other informatic tools that can identify leader TRS gene junctions.

Periscope v0.08a is another tool that was developed to identify sgmRNA from Illumina and Nanopore ARTIC amplicon sequencing data[17]. The tool functions based on searching a 32 nt leader sequence (genomic position: 34-65) and anchoring the known TRS-orf boundaries on the reads for identification of known sgmRNAs. Periscope does not take into consideration the sequences and distance between the leader and TRS-orf boundaries. Periscope can analyse ARTIC amplicon sequencing data, whereas LeTRS can also input a variety of different amplicon and direct RNA sequencing data. Given the very large sequencing datasets being generated as part of the global effort to sequence SARS-CoV-2 the performance of LeTRS was compared to Periscope in terms of computation time. Illumina sequencing data from a nasopharyngeal sample of a human patient with COVID-19 and Nanopore ARTIC amplicon published cell culture data sets were used for comparison. This used the number of reads with at least one primer sequence at either end in the LeTRS and the number of "High Quality" reads (the reads with both 32 nts leader sequences and known TRS-orf boundary) in Periscope. Periscope was run with the default setting[17]. Both LeTRS and Periscope identified a similar number of reads for both data sets (Supplementary Figure 2, Tables 1 and 3 and Supplementary table 7). With 16 CPU cores, the run times for LeTRS was 1m 40.692s and 2m 14.911s for these tested Illumina and Nanopore data sets, respectively, and for Periscope these were 7m 31.183s and 16m 49.448s. We also tested the data from ARTIC Illumina cell culture data, but Periscope had an error.

Analysis of sequencing data from longitudinal nasopharyngeal samples taken from two non-human primate models of COVID-19 indicated multi-phasic sgmRNA synthesis.

Part of the difficulty of studying SARS-CoV-2 and the disease COVID-19 is establishing the sequence of events from the start of infection. Most samples from humans are from nasopharyngeal aspirates taken when clinical symptoms develop. This tends to be 5 to 6 days post-exposure. In the absence of a human challenge model, animal models can be used to study the kinetics of SARS-CoV-2[18, 19]. Two separate non-human primate models, cynomolgus and rhesus macaques, were established for the study of SARS-CoV-2 that mirrored disease in the majority of humans[18]. To study the pattern of sgmRNA synthesis over the course of infection, nasopharyngeal samples were sequentially gathered daily from one day post-infection up to 18 days post-infection from the two NHP models. RNA was purified from these longitudinal samples as well as the inoculum virus and viral RNA sequenced using the ARTIC approach on the Illumina platform.

Analysis of the sequence data from the inoculum used to infect the NHPs indicated that leader gene junctions could be identified (Supplementary Figure 3, Supplementary_Table_8), but these did not follow the pattern of abundance of leader TRS-gene junctions found in infected cells in culture, where the leader TRS-N gene junction was most abundant (Figure 1C). In contrast, analysis of the longitudinal sequencing data from nasopharyngeal aspirates from the non-human primate model identified leader TRS-gene junctions associated with the major sgmRNAs (Figure 3, Supplementary_Table_9) as well as novel leader-TRS gene junction sites (Supplementary Figures 4 and 5). Analysing the abundance of the leader TRS-gene junctions for both model species over the course of infection revealed a phasic nature of sgmRNA synthesis. The leader TRS nucleoprotein gene junction was the most abundant, and there was a similar phasic pattern

of potential sgmRNA synthesis with Illumina ARTIC method (Figure 3). For both species, viral load and hence sgmRNA synthesis had dropped by Day 8 and Day 9.

Analysis of leader TRS-gene junction in human samples revealed expected and aberrant abundances

To investigate the pattern of leader-TRS gene junction abundance during infection of SARS-CoV-2 in humans, nasopharyngeal swabs form patients with COVID-19 were sequenced by the ARTIC approach using either Illumina (as part of COG-UK) (N=15 patients) (Figure 4, Supplementary Table 7) or by Oxford Nanopore (as part of ISARIC-4C) (N=15 patients) (Figure 5, Supplementary Table 10). In a number of samples, leader-TRS gene junctions were identified, and followed an expected pattern, with the nucleoprotein gene junction being the most abundant (e.g., Sample 1 in Figures 4A and B, Patient 2 day1 in Figure 5A and B). However, in several of the samples there was very large representation of single leader-TRS gene junction (e.g., Sample 4 and 5 in Figures 4A and B). These tended to map to the nucleoprotein gene (Sample 5, 8 and 13 Figures 4A and B). The heterogeneity in abundance of leader gene junctions was reminiscent for that from the non-human primate study with a defined and expected pattern near the start of infection but then becoming phasic. The samples gathered under ISARIC-4C were from hospitalised patients and permitted analysis in relation to reported date of symptom onset and sequential sampling. In general, the data indicated that on first sample on admission to hospital contained an abundance of leader-TRS gene junctions which resembled the pattern seen in infected cells (Patient 6 day1 and day9 in Figures 5A and B). However, with further days post-sample, e.g.

(Patient 7 day7 Figures 5A and B), the leader-TRS N gene junction was the most abundant and far exceeded any other detectable species. The abundance of leader-TRS N gene junction in the patients at a later stage of infection followed that observed in the NHP model (Figure 3).

Commonality of novel leader-TRS gene junctions

The sequencing data spanning cell culture infection, animal models and clinical samples from humans indicated the presence of novel leader-TRS gene junctions. Their detection generally increased with depth of coverage. Coronavirus replication and transcription is promiscuous, and recombination is a natural result of this, resulting in insertions, deletions and potential gene rearrangements. Many of these novel leader-TRS junctions were centred around the known gene open reading frame but out of the search interval. These type of leader-TRS gene junctions could be only found with spike, membrane, ORF6, ORF7b and nucleocapsid orfs, in which the membrane orf was the most common (Figure 6A). In order to define what might be genuine novel leader-TRS gene junctions, these were compared across the data in all Illumina ARTIC data (Figure 6B, Supplementary Table 11). This identified 5 novel leader-TRS junctions that were common to all the data, the majority of these being focused on the membrane orf.

Discussion

Coronavirus sgmRNAs are only synthesised during infection of cells and therefore their presence in sequence data can be indicative of active viral RNA synthesis. The abundance of the sgmRNAs in infected cells should follow a general pattern where the sgmRNA encoding the nucleoprotein is the most abundant. Identification and quantification of the unique leader-TRS gene junctions for each sgmRNA can be used as a proxy for their abundance.

LeTRS was developed to interrogate sequencing datasets to identify the leader-gene junctions present at the 5' end of the sgmRNAs. LeTRS was first evaluated and validated on cell culture data from published datasets[2, 16] and from a cell culture experiment as part of this study and then used in an analysis of nasopharyngeal samples from non-human primate and human clinical samples. The results showed that the positions of the leader-TRS junction sites with peak read counts were same as the given reference positions. The exception was at leader-gene junction for orf7b in the Nanopore sequencing. The normalized count results confirmed the reads spanning the junctions showed that the leader-TRS nucleoprotein gene junction was the most abundant, and orf7b and orf10 were the most infrequent in line with other data[2,20]. Several low abundant leader-TRS junctions were identified in all of the datasets with the implication these were either from potential lower abundant novel sgmRNAs, or represented known sgmRNAs, but with different leader-TRS junctions. Likewise, at low frequency these could represent an aberrant viral transcription process or artefacts of the different sequencing processes – although this latter possibility is less likely through the published direct RNA sequencing approach[2] (Figure 2B). Traditionally, such sgmRNAs have been first identified in coronaviruses by either northern blot

and/or metabolic labelling[8]. Several other groups have identified novel leader-TRS gene junctions and potential subgenomic mRNAs for other coronaviruses, including avian infectious bronchitis virus[21]. The best way of validating potential novel sgmRNAs would be through matching proteomic data to confirm genuine open reading frames[1]. Analysis of several published sequencing datasets identified novel viral RNA molecules that the authors suggested were sgmRNAs containing only the 5' region of orf1a[22]. Such species are likely to be defective RNAs, that act as templates for replication. Interestingly, they hypothesize that at later time points post-infection in cell culture potential novel sgRNAs are generated non-specifically[22], which potentially ties in with a disconnect of leader-TRS gene junctions observed in our study both *in vivo* from the nasopharyngeal samples from latter time points in the NHP models and in humans and from the published data from SARS-CoV-2 infections in cell culture gathered at later time points compared to earlier time points[2, 16].

Advanced filtering can improve the confidence of the identified leader-TRS junction in the sequencing reads. Amplicon sequencing provided a unique opportunity to filter the sequencing reads. The reads spanning the junctions with the correct forward primer, reverse primer or both primer sequences at the ends of reads proved the known/novel sgmRNA existing in tested Illumina and Nanopore ARTIC v3 primers amplicon sequencing data (Tables 1 and 3). For Illumina sequencing, the same junction on paired reads with at least a primer provided extra evidence for leader-TRS identification. Some reads were identified that did not have primer sequences and these were likely to be miss-mapped, from template sgmRNA or low-quality sequence. These were present at very low abundance compared to authentic mapped reads (Tables 1, 3 and 5).

No reads with polyA were detected in the Nanopore amplicon sequencing data, this was likely because the limited PCR extension time restricted the primers to reach the 5'end of subgenomic mRNA (Table 1 and Supplementary Table5). The Nanopore direct RNA sequencing had the potential to generate full length mRNA sequences. The polyA sequences and leader-TRS junctions in the reads can be good signals to prove the full length sgmRNA in the test data (Tables 3 and 4). Because the fortuitous sequencing of some host mRNA may lead false positive result, LeTRS was tested against sequence data from uninfected controls cells[2]. No positive reads were found in this control sample (Tables 5 and 6), suggesting the LeTRS could effectively screen out or not recognise any false positives. Crucially, LeTRS used less CPU runtime and provided more detailed information than other tools to investigate this[17], and therefore is suited for the high throughput analysis of large amounts of diverse sequencing data.

In terms of clinical samples (typically nasopharyngeal swabs), the presence of sgmRNAs will be due to the presence infected cells. In general, this has been seen as indicative of active viral RNA synthesis at the time of sampling[5, 23, 24], although these have also been postulated to be present through resistant structures after infection has finished[25]. Analysis of inoculum indicated that leader-TRS gene junctions could be identified (Supplementary Figure 3) but that these were not in the same ratio as found in cells infected in culture (e.g., Figure 2B and 2C). Thus, if the abundance of leader-TRS gene junctions follows an expected pattern of the nucleoprotein gene leader-TRS gene junction being the most abundant followed by a general gradient in sequence data from nasopharyngeal samples, then this may be indicative of an active infection – and the presence of infected cells in a sample.

In the absence of a human challenge model, NHP models that closely resemble COVID-19 disease in humans can be used to study SARS-CoV-2 infection, from a very defined initial exposure. RNA was sequenced from longitudinal nasopharyngeal samples from two NHP models, rhesus and cynomolgus macaques[18]. LeTRS used to identify the abundance of the leader-TRS gene junctions in this data. The analysis indicated a phasic pattern of sgmRNA synthesis with a large drop off after Day 8/9 post-infection in both NHP models. This phasic pattern may be explained by an initial synchronous infection of respiratory epithelial cells and then these cells dying. Released virus then goes on to infect new epithelial cells with virus infection increasing exponentially in waves but becoming asynchronous. The decline in sgmRNA from Day 8/9 overlaps with IgG seroconversion and humoral immunity in both species[18] and follows similar kinetics to serology profiles measured in patients with COVID-19.

The identification of sgmRNAs in nasopharyngeal samples and their kinetics has implications for nucleic acid-based diagnostics (many of which have three targets, one in the orf1a/b region and two which are shared between the genome and sgmRNAs – the nucleoprotein and the spike genes). The phasic nature of leader-TRS gene junctions in the longitudinal samples, and by implication sgmRNAs, and overt abundance of the leader-TRS nucleoprotein gene junction found in many of the human samples, suggests that it may not be possible to precisely identify where in infection an individual is based on the abundance of sgmRNAs. Likewise, assuming equivalency between the targets, if the nucleoprotein target is found to be more abundant than the spike target than the genomic target, then this would suggest infected cells are present in the sample.

Decreases in Ct values associated with emerging variants could equally be explained by sloughed cells being present in a nasopharyngeal sample as well as by increases in the amount of virions/viral load. Therefore, we would caution that a decrease in Ct associated with RT-qPCR based assays may not just be reflective of higher viral loads but also may be indicative of more infected cells being present. These possibilities may be resolved by considering the relative ratios of sgmRNAs identified.

METHODS

Data input

LeTRS was designed to analyse FastQ files derived from Illumina paired-end or Nanopore sequencing data derived from a SARS-CoV-2 amplicon protocol, or standard Nanopore SARS-CoV-2 direct RNA sequencing data (Figure 1). The Illumina/Nanopore FastQ sequencing data were cleaned to remove adapters and low-quality reads before input. Sequencing data derived from other sequencing modes or platforms can also be analysed by LeTRS via input of a BAM file produced by a custom splicing alignment method with a SARS-CoV-2 genome (NC_045512.2) as a reference (Figure 1). This can also be rapidly adapted for other coronaviruses.

Library preparations and sequencing

We sequenced the 15 samples from human patients with nanopore. Total RNA was isolated using a QIAamp Viral RNA Mini Kit (Qiagen, Manchester, UK) by spin-column procedure according to the manufacturer's instructions. Clinical samples were extracted with Trizol LS as described[4]. All RNA samples were treated with Turbo DNase (Invitrogen). SuperScript IV (Invitrogen) was used to generate single-strand cDNA using random primer mix (NEB, Hitchin, UK). ARTIC V3 PCR amplicons from the single-strand cDNA were generated following the Nanopore Protocol of PCR tiling of SARS-CoV-2 virus (Version: PTC_9096_v109_revL_06Feb2020). Amplicons generated by ARTIC PCR were purified and normalised to 200 fmol before DNA end preparation and barcode and adapter ligation. Library was loaded onto a FLO-MIN106 flow cell and sequencing reads were called with Guppy using the high-accuracy calling parameters.

The NHP samples and their inoculum, and our laboratory experiments conducted in cells were sequenced with Illumina. The amplicons products for Illumina sequencing were prepared as per the Nanopore sequencing above and then used in Illumina NEBNext Ultra II DNA Library preparation. Following 4 cycles of amplification the library was purified using Ampure XP beads and quantified using Qubit and the size distribution assessed using the Fragment analyzer. Finally, the ARTIC library was sequenced on the Illumina® NovaSeq 6000 platform (Illumina®, San Diego, USA) following the standard workflow. The generated raw FastQ files (2 x 250 bp) were trimmed to remove Illumina adapter sequences using Cutadapt v1.2.1 [26]. The option "−O 3" was set, so the that 3' end of any reads which matched the adapter sequence with greater than 3 bp was trimmed off. The reads were further trimmed to remove low quality bases, using Sickle v1.200 [27] with a minimum window quality score of 20. After trimming, reads shorter than 10 bp were removed.

The LeTRS was also tested with a combined Nanopore cDNA ARTIC v3 amplicon dataset of 7 published viral cell culture samples (barcode01-barcode07) [16], and a dataset from a published direct RNA Nanopore sequencing analysis Vero cells infected with SARS-CoV-2 or an uninfected negative control [2].

Sequencing data alignment and basic filtering

LeTRS controlled Hisat2 v2.1.0 [28] to map the paired-end Illumina reads against the SARS-CoV-2 reference genome (NC_045512.2) with the default setting, and Minimap2 v2.1 [29] to align the Nanopore cDNA reads and direct RNA-seq reads on the viral genome using Minimap2 with "–ax

splice" and "-ax splice -uf -k14" parameters, respectively. LeTRS provided 10 known leader-TRS junctions to improve alignment accuracy by using "--known-splicesite-infile" function in Hisat2 and "--junc-bed" function in Minimap2, but this application could be optionally switched off by users. In order to remove low mapping quality and mis-mapped reads before searching the leader-TRS junction sites, LeTRS used Samtools v1.9 [30] to have basic filtering for the reads in the output Sam/Bam files according to their alignment states as shown (Table 9 - basic filtering).

Search leader-TRS

After the mapping and basic filtering step, LeTRS searched aligned reads spanning the leader-TRS junctions in the SARS-CoV-2 reference genome (Supplementary Figure 1). For the known leader-TRS junctions, LeTRS searched the reads including the leader-TRS junctions within a given interval around the known leader and TRS junctions sites. The leader break site interval is ±10 nts, and the TRS breaking sites interval is -20 nts to the 1 nt before the first known AUG in the default setting (the intervals can be changed to custom values to investigate heterogeneity). LeTRS then reported a peak count that was the number of reads carrying the most common leader-TRS junctions within the given leader and TRS breaking sites intervals, and a cluster count that was the number of all reads carrying leader-TRS junctions within the given leader and TRS breaking sites intervals (Tables 1-6). LeTRS also searched the junctions out of the given intervals (the genomic position of leader breaking site < 80) and reported the number of reads (>10 by default) with novel leader-TRS junctions. These number of read counts were also reported by number of reads in 1000000 as normalization. The read including the known and novel leader-TRS junctions could be optionally outputted in FastA format. Based on identified known and novel leader-TRS

junctions, LeTRS could report 20 nucleotides towards the 3' end of the leader sequence, the TRS and translated the first orf of sgmRNAs sequence, and find the conserved ACGAAC sequences in the TRS (Table S1-S6).

Advance filtering

Based on the alignment possibilities illustrated in Figure 2 and discussed, LeTRS further filters the identified reads with known and novel leader-TRS junctions. This step is named as advance filtering and can only applied when the input data is from Illumina paired end reads, Nanopore cDNA reads or Nanopore RNA reads (Table 9). If a BAM file is used as input data, the advanced filtering step would be automatically skipped (Table 9). The number of reads including the known and novel leader-TRS junctions, and the number of reads filtered with corresponding advance filtering criteria were outputted into two tables in tab format (Tables 1-6).

Leader-TRS junction plotting

LeTRS-plot was developed as an automatic plotting tool that interfaces with the R package ggplot2 v3.3.3 to view the leader-TRS junctions in the tables generated by LeTRS (Figure 3-5). The plot shows peak count, filtered peak count, normalized peak count and normalized filtered peak count for known leader-TRS junctions, and novel junction counts, filtered novel junction count, normalized novel junction count and filtered normalized novel junction for novel leader-TRS junctions.

References

1. Davidson, A.D. et al. Characterisation of the transcriptome and proteome of SARS-CoV-2 reveals a cell passage induced in-frame deletion of the furin-like cleavage site from the spike glycoprotein. *Genome Med* **12**, 68 (2020).
2. Kim, D. et al. The Architecture of SARS-CoV-2 Transcriptome. *Cell* **181**, 914-921 e910 (2020).
3. Nasir, J.A. et al. A Comparison of Whole Genome Sequencing of SARS-CoV-2 Using Amplicon-Based Sequencing, Random Hexamers, and Bait Capture. *Viruses* **12** (2020).
4. Moore, S.C. et al. Amplicon-Based Detection and Sequencing of SARS-CoV-2 in Nasopharyngeal Swabs from Patients With COVID-19 and Identification of Deletions in the Viral Genome That Encode Proteins Involved in Interferon Antagonism. *Viruses* **12** (2020).
5. Dorward, D.A. et al. Tissue-Specific Immunopathology in Fatal COVID-19. *Am J Respir Crit Care Med* **203**, 192-201 (2021).
6. Graham, R.L., Sparks, J.S., Eckerle, L.D., Sims, A.C. & Denison, M.R. SARS coronavirus replicase proteins in pathogenesis. *Virus Res* **133**, 88-100 (2008).
7. Pyrc, K., Jebbink, M.F., Berkhout, B. & van der Hoek, L. Genome structure and transcriptional regulation of human coronavirus NL63. *Virol J* **1**, 7 (2004).
8. Hiscox, J.A., Cavanagh, D. & Britton, P. Quantification of individual subgenomic mRNA species during replication of the coronavirus transmissible gastroenteritis virus. *Virus Res* **36**, 119-130 (1995).

9. Hiscox, J.A., Mawditt, K.L., Cavanagh, D. & Britton, P. Investigation of the control of coronavirus subgenomic mRNA transcription by using T7-generated negative-sense RNA transcripts. *J Virol* **69**, 6219-6227 (1995).

10. van Marle, G., Luytjes, W., van der Most, R.G., van der Straaten, T. & Spaan, W.J. Regulation of coronavirus mRNA transcription. *J Virol* **69**, 7851-7856 (1995).

11. La Monica, N., Yokomori, K. & Lai, M.M. Coronavirus mRNA synthesis: identification of novel transcription initiation signals which are differentially regulated by different leader sequences. *Virology* **188**, 402-407 (1992).

12. Alonso, S., Izeta, A., Sola, I. & Enjuanes, L. Transcription regulatory sequences and mRNA expression levels in the coronavirus transmissible gastroenteritis virus. *J Virol* **76**, 1293-1308 (2002).

13. Sawicki, S.G., Sawicki, D.L. & Siddell, S.G. A contemporary view of coronavirus transcription. *J Virol* **81**, 20-29 (2007).

14. Jeong, Y.S. & Makino, S. Evidence for coronavirus discontinuous transcription. *J Virol* **68**, 2615-2623 (1994).

15. Cevik, M., Kuppalli, K., Kindrachuk, J. & Peiris, M. Virology, transmission, and pathogenesis of SARS-CoV-2. *BMJ* **371**, m3862 (2020).

16. Tyson, J.R. et al. Improvements to the ARTIC multiplex PCR method for SARS-CoV-2 genome sequencing using nanopore. *bioRxiv* (2020).

17. Parker, M.D. et al. periscope: sub-genomic RNA identification in SARS-CoV-2 Genomic Sequencing Data. *bioRxiv*, 2020.2007.2001.181867 (2020).

18. Salguero, F.J. et al. Comparison of rhesus and cynomolgus macaques as an infection model for COVID-19. *Nat Commun* **12**, 1260 (2021).

19. Ryan, K.A. et al. Dose-dependent response to infection with SARS-CoV-2 in the ferret model and evidence of protective immunity. *Nat Commun* **12**, 81 (2021).

20. Alexandersen, S., Chamings, A. & Bhatta, T.R. SARS-CoV-2 genomic and subgenomic RNAs in diagnostic samples are not an indicator of active replication. *Nature communications* **11**, 1-13 (2020).

21. Keep, S. et al. Multiple novel non-canonically transcribed sub-genomic mRNAs produced by avian coronavirus infectious bronchitis virus. *J Gen Virol* **101**, 1103-1118 (2020).

22. Nomburg, J., Meyerson, M. & DeCaprio, J.A. Pervasive generation of non-canonical subgenomic RNAs by SARS-CoV-2. *Genome Med* **12**, 108 (2020).

23. Corbett, K.S. et al. Evaluation of the mRNA-1273 Vaccine against SARS-CoV-2 in Nonhuman Primates. *N Engl J Med* **383**, 1544-1555 (2020).

24. Yu, J. et al. DNA vaccine protection against SARS-CoV-2 in rhesus macaques. *Science* **369**, 806-811 (2020).

25. Alexandersen, S., Chamings, A. & Bhatta, T.R. SARS-CoV-2 genomic and subgenomic RNAs in diagnostic samples are not an indicator of active replication. *Nat Commun* **11**, 6059 (2020).

26. Martin, M. Cutadapt removes adapter sequences from high-throughput sequencing reads. *EMBnet.journal* **17**, https://doi.org/10.14806/ej.14817.14801.14200 (2011).

27. Joshi, N.A. & Fass, J.N. Sickle: A sliding-window, adaptive, quality-based trimming tool for FastQ files

(Version 1.33). https://github.com/najoshi/sickle (2011).

28. Kim, D., Langmead, B. & Salzberg, S.L. HISAT: a fast spliced aligner with low memory requirements. *Nat Methods* **12**, 357-360 (2015).

29. Li, H. Minimap2: pairwise alignment for nucleotide sequences. *Bioinformatics* **34**, 3094-3100 (2018).

30. Li, H. et al. The Sequence Alignment/Map format and SAMtools. *Bioinformatics* **25**, 2078-2079 (2009).

29 Li, H. Minimap2: pairwise alignment for nucleotide sequences. *Bioinformatics* **34**, 3094-3100, doi:10.1093/bioinformatics/bty191 (2018).

30 Li, H. *et al.* The Sequence Alignment/Map format and SAMtools. *Bioinformatics* **25**, 2078-2079, doi:10.1093/bioinformatics/btp352 (2009).

Ethics approval and consent to participate

All experimental work on NHPs was conducted under the authority of a UK Home Office approved project license (PDC57C033) that had been subject to local ethical review at PHE Porton Down by the Animal Welfare and Ethical Review Body (AWERB) and approved as required by the Home Office Animals (Scientific Procedures) Act 1986 and the full ethics and NHP model are described.

Consent for publication

Not applicable

Availability of data and materials

LeTRS is available at https://github.com/xiaofengdong83/LeTRS.

Illumina and nanopore test data sets are available under NCBI PRJNA699398.

Competing interests

The authors declare that they have no competing interests

Funding

This work was funded by U.S. Food and Drug Administration Medical Countermeasures Initiative contract (75F40120C00085) awarded to JAH. The article reflects the views of the authors and does not represent the views or policies of the FDA. This work was also supported by the MRC (MR/W005611/1) G2P-UK: A national virology consortium to address phenotypic consequences of SARS-CoV-2 genomic variation (co-I JAH). JAH is also funded by the Centre of Excellence in Infectious Diseases Research (CEIDR) and the Alder Hey Charity. The non-human primate work was funded by the Coalition of Epidemic Preparedness Innovations (CEPI) and the Medical Research Council Project CV220-060, Development of an NHP model of infection and ADE with

COVID-19 (SARS-CoV-2) both awarded to MWC. The ISARIC4C sample collection and sequencing in this study was supported by a grants from the Medical Research Council (grant MC_PC_19059), the National Institute for Health Research (NIHR; award CO-CIN-01), the Medical Research Council (MRC; grant MC_PC_19059), and by the NIHR Health Protection Research Unit (HPRU) in Emerging and Zoonotic Infections at University of Liverpool in partnership with Public Health England (PHE), in collaboration with Liverpool School of Tropical Medicine and the University of Oxford (award 200907), NIHR HPRU in Respiratory Infections at Imperial College London with PHE (award 200927), Wellcome Trust and Department for International Development (DID; 215091/Z/18/Z), the Bill and Melinda Gates Foundation (OPP1209135), Liverpool Experimental Cancer Medicine Centre (grant reference C18616/A25153), NIHR Biomedical Research Centre at Imperial College London (IS-BRC-1215-20013), PJMO is supported by a NIHR senior investigator award (201385). The views expressed are those of the authors and not necessarily those of the Department of Health and Social Care, DID, NIHR, MRC, Wellcome Trust, or PHE. The funders had no role in the study design; in the collection, analysis, and interpretation of data; in the writing of the report; or in the decision to submit the article for publication.

Authors' contributions

X.D. developed the LeTRS software and performed the informatics analysis. X.D., A.D. and J.A.H. analysed the data. J.S., J.T. and M.W.C. co-ordinated the NHP work and sample processing. R.P.-R., J.P.S., H.G., T.P. and N.R. were involved in sequencing and informatics analysis of the NHP samples with D.A.M. A.D. oversaw sequencing of the human clinical samples with E.V. and C.N for the COG-UK data. R.P.-R. and J.A.H. oversaw sequencing of samples under the auspices of

ISARIC-4C managed by J.K.B, L.T., M.G.S. and P.J.M.O. J.A.H. and M.W.C. initiated and managed the study and wrote the manuscript with X.D., R.P.-R., A.D. with other authors involved in editing the final version.

Acknowledgments

We would like to thank all members of the Hiscox Laboratory and the Centre for Genome Research for supporting SARS-CoV-2/COVID-19 sequencing research. We would like to acknowledge members of the COG-UK and ISARIC4C consortia for acquisition of the human samples used in this study. **The members of the COG-UK consortia are:** Thomas R. Connor, Nicholas J. Loman, Samuel C. Robson, Tanya Golubchik, M. Estee Torok, William L. Hamilton, David Bonsall, Ali R. Awan, Sally Corden, Ian Goodfellow, Darren L. Smith, Martin D. Curran, Surendra Parmar, James G. Shepherd, Matthew D. Parker, Catherine Moore, Derek J. Fairley, Matthew W. Loose, Joanne Watkins, Matthew Bull, Sam Nicholls, David M. Aanensen, Sharon Glaysher, Matthew Bashton, Nicole Pacchiarini, Anthony P. Underwood, Thushan I. de Silva, Dennis Wang, Monique Andersson, Anoop J. Chauhan, Mariateresa de Cesare, Catherine Ludden, Tabitha W. Mahungu, Rebecca Dewar, Martin P. McHugh, Natasha G. Jesudason, Kathy K. Li, Rajiv N. Shah, Yusri Taha, Kate E. Templeton, Simon Cottrell, Justin O'Grady, Andrew Rambaut, Colin P. Smith, Matthew T.G. Holden, Emma C. Thomson, Samuel Moses, Meera Chand, Chrystala Constantinidou, Alistair C. Darby, Julian A. Hiscox, Steve Paterson, Meera Unnikrishnan, Andrew J. Page, Erik M. Volz, Charlotte J. Houldcroft, Aminu S. Jahun, James P. McKenna, Luke W. Meredith, Andrew Nelson, Sarojini Pandey, Gregory R. Young, Anna Price, Sara Rey, Sunando Roy, Ben Temperton, Matthew Wyles, Stefan Rooke, Sharif Shaaban, Helen Adams, Yann Bourgeois, Katie F. Loveson, Áine O'Toole, Richard Stark, Ewan M. Harrison, David Heyburn, Sharon J.

Peacock, David Buck, Michaela John, Dorota Jamrozy, Joshua Quick, Rahul Batra, Katherine L. Bellis, Beth Blane, Sophia T. Girgis, Angie Green, Anita Justice, Mark Kristiansen, Rachel J. Williams, Radoslaw Poplawski, Garry P. Scarlett, John A. Todd, Christophe Fraser, Judith Breuer, Sergi Castellano, Stephen L. Michell, Dimitris Gramatopoulos, Jonathan Edgeworth, Gemma L. Kay, Ana da Silva Filipe, Aaron R. Jeffries, Sascha Ott, Oliver Pybus, David L. Robertson, David A. Simpson, Chris Williams, Cressida Auckland, John Boyes, Samir Dervisevic, Sian Ellard, Sonia Goncalves, Emma J. Meader, Peter Muir, Husam Osman, Reenesh Prakash, Venkat Sivaprakasam, Ian B. Vipond, Jane A.H. Masoli, Nabil-Fareed Alikhan, Matthew Carlile, Noel Craine, Sam T. Haldenby, Nadine Holmes, Ronan A. Lyons, Christopher Moore, Malorie Perry, Ben Warne, Thomas Williams, Lisa Berry, Andrew Bosworth, Julianne Rose Brown, Sharon Campbell, Anna Casey, Gemma Clark, Jennifer Collins, Alison Cox, Thomas Davis, Gary Eltringham, Cariad Evans, Clive Graham, Fenella Halstead, Kathryn Ann Harris, Christopher Holmes, Stephanie Hutchings, Miren Iturriza-Gomara, Kate Johnson, Katie Jones, Alexander J. Keeley, Bridget A. Knight, Cherian Koshy, Steven Liggett, Hannah Lowe, Anita O. Lucaci, Jessica Lynch, Patrick C McClure, Nathan Moore, Matilde Mori, David G. Partridge, Pinglawathee Madona, Hannah M. Pymont, Paul Anthony Randell, Mohammad Raza, Felicity Ryan, Robert Shaw, Tim J. Sloan, Emma Swindells, Alexander Adams, Hibo Asad, Alec Birchley, Tony Thomas Brooks, Giselda Bucca, Ethan Butcher, Sarah L. Caddy, Laura G. Caller, Yasmin Chaudhry, Jason Coombes, Michelle Cronin, Patricia L. Dyal, Johnathan M. Evans, Laia Fina, Bree Gatica-Wilcox, Iliana Georgana, Lauren Gilbert, Lee Graham, Danielle C. Groves, Grant Hall, Ember Hilvers, Myra Hosmillo, Hannah Jones, Sophie Jones, Fahad A. Khokhar, Sara Kumziene-Summerhayes, George MacIntyre-Cockett, Rocio T. Martinez Nunez, Caoimhe McKerr, Claire McMurray, Richard Myers, Yasmin Nicole Panchbhaya, Malte L. Pinckert, Amy

Plimmer, Joanne Stockton, Sarah Taylor, Alicia Thornton, Amy Trebes, Alexander J. Trotter, Helena Jane Tutill, Charlotte A. Williams, Anna Yakovleva, Wen C. Yew, Mohammad T. Alam, Laura Baxter, Olivia Boyd, Fabricia F. Nascimento, Timothy M. Freeman, Lily Geidelberg, Joseph Hughes, David Jorgensen, Benjamin B. Lindsey, Richard J. Orton, Manon Ragonnet-Cronin, Joel Southgate, Sreenu Vattipally, Igor Starinskij, Joshua B. Singer, Khalil Abudahab, Leonardo de Oliveira Martins, Thanh Le-Viet, Mirko Menegazzo, Ben E.W. Taylor, Corin A. Yeats, Sophie Palmer, Carol M. Churcher, Alisha Davies, Elen De Lacy, Fatima Downing, Sue Edward, Nikki Smith, Frances Bolt, Alex Alderton, Matt Berriman, Ian G. Charles, Nicholas Cortes, Tanya Curran, John Danesh, Sahar Eldirdiri, Ngozi Elumogo, Andrew Hattersley, Alison Holmes, Robin Howe, Rachel Jones, Anita Kenyon, Robert A. Kingsley, Dominic Kwiatkowski, Cordelia Langford, Jenifer Mason, Alison E. Mather, Lizzie Meadows, Sian Morgan, James Price, Trevor I. Robinson, Giri Shankar, John Wain, Mark A. Webber, Declan T. Bradley, Michael R. Chapman, Derrick Crooke, David Eyre, Martyn Guest, Huw Gulliver, Sarah Hoosdally, Christine Kitchen, Ian Merrick, Siddharth Mookerjee, Robert Munn, Timothy Peto, Will Potter, Dheeraj K Sethi, Wendy Smith, Luke B. Snell, Rachael Stanley, Claire Stuart, Elizabeth Wastenge, Erwan Acheson, Safiah Afifi, Elias Allara, Roberto Amato, Adrienn Angyal, Elihu Aranday-Cortes, Cristina Ariani, Jordan Ashworth, Stephen Attwood, Alp Aydin, David J. Baker, Carlos E. Balcazar, Angela Beckett, Robert Beer, Gilberto Betancor, Emma Betteridge, David Bibby, Daniel Bradshaw, Catherine Bresner, Hannah E. Bridgewater, Alice Broos, Rebecca Brown, Paul E. Brown, Kirstyn Brunker, Stephen N. Carmichael, Jeffrey K.J. Cheng, Rachel Colquhoun, Gavin Dabrera, Johnny Debebe, Eleanor Drury, Louis du Plessis, Richard Eccles, Nicholas Ellaby, Audrey Farbos, Ben Farr, Jacqueline Findlay, Chloe L. Fisher, Leysa Marie Forrest, Sarah Francois, Lucy R. Frost, William Fuller, Eileen Gallagher, Michael D. Gallagher,

Matthew Gemmell, Rachel A.J. Gilroy, Scott Goodwin, Luke R. Green, Richard Gregory, Natalie Groves, James W. Harrison, Hassan Hartman, Andrew R. Hesketh, Verity Hill, Jonathan Hubb, Margaret Hughes, David K. Jackson, Ben Jackson, Keith James, Natasha Johnson, Ian Johnston, Jon-Paul Keatley, Moritz Kraemer, Angie Lackenby, Mara Lawniczak, David Lee, Rich Livett, Stephanie Lo, Daniel Mair, Joshua Maksimovic, Nikos Manesis, Robin Manley, Carmen Manso, Angela Marchbank, Inigo Martincorena, Tamyo Mbisa, Kathryn McCluggage, J.T. McCrone, Shahjahan Miah, Michelle L. Michelsen, Mari Morgan, Gaia Nebbia, Charlotte Nelson, Jenna Nichols, Paola Niola, Kyriaki Nomikou, Steve Palmer, Naomi Park, Yasmin A. Parr, Paul J. Parsons, Vineet Patel, Minal Patel, Clare Pearson, Steven Platt, Christoph Puethe, Mike Quail, Jayna Raghwani, Lucille Rainbow, Shavanthi Rajatileka, Mary Ramsay, Paola C. Resende Silva, Steven Rudder, Chris Ruis, Christine M. Sambles, Fei Sang, Ulf Schaefer, Emily Scher, Carol Scott, Lesley Shirley, Adrian W. Signell, John Sillitoe, Christen Smith, Katherine L. Smollett, Karla Spellman, Thomas D. Stanton, David J. Studholme, Grace Taylor-Joyce, Ana P. Tedim, Thomas Thompson, Nicholas M. Thomson, Scott Thurston, Lily Tong, Gerry Tonkin-Hill, Rachel M. Tucker, Edith E. Vamos, Tetyana Vasylyeva, Joanna Warwick-Dugdale, Danni Weldon, Mark Whitehead, David Williams, Kathleen A. Williamson, Harry D. Wilson, Trudy Workman, Muhammad Yasir, Xiaoyu Yu, Alex Zarebski, Evelien M. Adriaenssens, Shazaad S.Y. Ahmad, Adela Alcolea-Medina, John Allan, Patawee Asamaphan, Laura Atkinson, Paul Baker, Jonathan Ball, Edward Barton, Mathew A. Beale, Charlotte Beaver, Andrew Beggs, Andrew Bell, Duncan J Berger, Louise Berry, Claire M. Bewshea, Kelly Bicknell, Paul Bird, Chloe Bishop, Tim Boswell, Cassie Breen, Sarah K. Buddenborg, Shirelle Burton-Fanning, Vicki Chalker, Joseph G. Chappell, Themoula Charalampous, Claire Cormie, Nick Cortes, Lindsay J. Coupland, Angela Cowell, Rose K. Davidson, Joana Dias, Maria Diaz,

Thomas Dibling, Matthew J. Dorman, Nichola Duckworth, Scott Elliott, Sarah Essex, Karlie Fallon, Theresa Feltwell, Vicki M Fleming, Sally Forrest, Luke Foulser, Maria V. Garcia-Casado, Artemis Gavriil, Ryan P. George, Laura Gifford, Harmeet K. Gill, Jane Greenaway, Luke Griffith, Ana Victoria Gutierrez, Antony D. Hale, Tanzina Haque, Katherine L. Harper, Ian Harrison, Judith Heaney, Thomas Helmer, Ellen E. Higginson, Richard Hopes, Hannah C. Howson-Wells, Adam D. Hunter, Robert Impey, Dianne Irish-Tavares, David A. Jackson, Kathryn A. Jackson, Amelia Joseph, Leanne Kane, Sally Kay, Leanne M. Kermack, Manjinder Khakh, Stephen P. Kidd, Anastasia Kolyva, Jack C.D. Lee, Laura Letchford, Nick Levene, Lisa J. Levett, Michelle M. Lister, Allyson Lloyd, Joshua Loh, Louissa R. Macfarlane-Smith, Nicholas W. Machin, Mailis Maes, Samantha McGuigan, Liz McMinn, Lamia Mestek-Boukhibar, Zoltan Molnar, Lynn Monaghan, Catrin Moore, Plamena Naydenova, Alexandra S. Neaverson, Rachel Nelson, Marc O. Niebel, Elaine O'Toole, Debra Padgett, Gaurang Patel, Brendan A.I. Payne, Liam Prestwood, Veena Raviprakash, Nicola Reynolds, Alex Richter, Esther Robinson, Hazel A. Rogers, Aileen Rowan, Garren Scott, Divya Shah, Nicola Sheriff, Graciela Sluga, Emily Souster, Michael Spencer-Chapman, Sushmita Sridhar, Tracey Swingler, Julian Tang, Graham P. Taylor, Theocharis Tsoleridis, Lance Turtle, Sarah Walsh, Michelle Wantoch, Joanne Watts, Sheila Waugh, Sam Weeks, Rebecca Williams, Iona Willingham, Emma L. Wise, Victoria Wright, Sarah Wyllie, Jamie Young, Amy Gaskin, Will Rowe, Igor Siveroni, and Robert Johnson.

The members of the ISARIC4C consortia are: Consortium Lead Investigator: J. Kenneth Baillie; Chief Investigator: Malcolm G. Semple; Co-Lead Investigator: Peter J.M. Openshaw; ISARIC Clinical Coordinator: Gail Carson; Co-Investigators: Beatrice Alex, Benjamin Bach, Wendy S. Barclay, Debby Bogaert, Meera Chand, Graham S. Cooke, Annemarie B. Docherty, Jake Dunning, Ana da Silva Filipe, Tom Fletcher, Christopher A. Green, Ewen M. Harrison, Julian A. Hiscox,

Antonia Ying Wai Ho, Peter W. Horby, Samreen Ijaz, Saye Khoo, Paul Klenerman, Andrew Law, Wei Shen Lim, Alexander J. Mentzer, Laura Merson, Alison M. Meynert, Mahdad Noursadeghi, Shona C. Moore, Massimo Palmarini, William A. Paxton, Georgios Pollakis, Nicholas Price, Andrew Rambaut, David L. Robertson, Clark D. Russell, Vanessa Sancho-Shimizu, Janet T. Scott, Thushan de Silva, Louise Sigfrid, Tom Solomon, Shiranee Sriskandan, David Stuart, Charlotte Summers, Richard S. Tedder, Emma C. Thomson, A.A. Roger Thompson, Ryan S. Thwaites, Lance C.W. Turtle, and Maria Zambon; Project Managers: Hayley Hardwick, Chloe Donohue, Ruth Lyons, Fiona Griffiths, and Wilna Oosthuyzen; Data Analysts: Lisa Norman, Riinu Pius, Tom M. Drake, Cameron J. Fairfield, Stephen Knight, Kenneth A. Mclean, Derek Murphy, and Catherine A. Shaw; Data and Information System Managers: Jo Dalton, James Lee, Daniel Plotkin, Michelle Girvan, Egle Saviciute, Stephanie Roberts, Janet Harrison, Laura Marsh, Marie Connor, Sophie Halpin, Clare Jackson, and Carrol Gamble; Data Integration and Presentation: Gary Leeming, Andrew Law, Murray Wham, Sara Clohisey, Ross Hendry, and James Scott-Brown; Material Management: William Greenhalf, Victoria Shaw, and Sarah McDonald; Patient Engagement: Seán Keating; Outbreak Laboratory Staff and Volunteers: Katie A. Ahmed, Jane A. Armstrong, Milton Ashworth, Innocent G. Asiimwe, Siddharth Bakshi, Samantha L. Barlow, Laura Booth, Benjamin Brennan, Katie Bullock, Benjamin W.A. Catterall, Jordan J. Clark, Emily A. Clarke, Sarah Cole, Louise Cooper, Helen Cox, Christopher Davis, Oslem Dincarslan, Chris Dunn, Philip Dyer, Angela Elliott, Anthony Evans, Lorna Finch, Lewis W.S. Fisher, Terry Foster, Isabel Garcia-Dorival, Willliam Greenhalf, Philip Gunning, Catherine Hartley, Antonia Ho, Rebecca L. Jensen, Christopher B. Jones, Trevor R. Jones, Shadia Khandaker, Katharine King, Robyn T. Kiy, Chrysa Koukorava, Annette Lake, Suzannah Lant, Diane Latawiec, L. Lavelle-Langham, Daniella Lefteri, Lauren Lett, Lucia A. Livoti,

Maria Mancini, Sarah McDonald, Laurence McEvoy, John McLauchlan, Soeren Metelmann, Nahida S. Miah, Joanna Middleton, Joyce Mitchell, Shona C. Moore, Ellen G. Murphy, Rebekah Penrice-Randal, Jack Pilgrim, Tessa Prince, Will Reynolds, P. Matthew Ridley, Debby Sales, Victoria E. Shaw, Rebecca K. Shears, Benjamin Small, Krishanthi S. Subramaniam, Agnieska Szemiel, Aislynn Taggart, Jolanta Tanianis-Hughes, Jordan Thomas, Erwan Trochu, Libby van Tonder, Eve Wilcock, and J. Eunice Zhang; Local Principal Investigators: Kayode Adeniji, Daniel Agranoff, Ken Agwuh, Dhiraj Ail, Ana Alegria, Brian Angus, Abdul Ashish, Dougal Atkinson, Shahedal Bari, Gavin Barlow, Stella Barnass, Nicholas Barrett, Christopher Bassford, David Baxter, Michael Beadsworth, Jolanta Bernatoniene, John Berridge, Nicola Best, Pieter Bothma, David Brealey, Robin Brittain-Long, Naomi Bulteel, Tom Burden, Andrew Burtenshaw, Vikki Caruth, David Chadwick, Duncan Chambler, Nigel Chee, Jenny Child, Srikanth Chukkambotla, Tom Clark, Paul Collini, Catherine Cosgrove, Jason Cupitt, Maria-Teresa Cutino-Moguel, Paul Dark, Chris Dawson, Samir Dervisevic, Phil Donnison, Sam Douthwaite, Ingrid DuRand, Ahilanadan Dushianthan, Tristan Dyer, Cariad Evans, Chi Eziefula, Chrisopher Fegan, Adam Finn, Duncan Fullerton, Sanjeev Garg, Sanjeev Garg, Atul Garg, Effrossyni Gkrania-Klotsas, Jo Godden, Arthur Goldsmith, Clive Graham, Elaine Hardy, Stuart Hartshorn, Daniel Harvey, Peter Havalda, Daniel B. Hawcutt, Maria Hobrok, Luke Hodgson, Anil Hormis, Michael Jacobs, Susan Jain, Paul Jennings, Agilan Kaliappan, Vidya Kasipandian, Stephen Kegg, Michael Kelsey, Jason Kendall, Caroline Kerrison, Ian Kerslake, Oliver Koch, Gouri Koduri, George Koshy, Shondipon Laha, Steven Laird, Susan Larkin, Tamas Leiner, Patrick Lillie, James Limb, Vanessa Linnett, Jeff Little, Michael MacMahon, Emily MacNaughton, Ravish Mankregod, Huw Masson, Elijah Matovu, Katherine McCullough, Ruth McEwen, Manjula Meda, Gary Mills, Jane Minton, Mariyam Mirfenderesky,

Kavya Mohandas, Quen Mok, James Moon, Elinoor Moore, Patrick Morgan, Craig Morris, Katherine Mortimore, Samuel Moses, Mbiye Mpenge, Rohinton Mulla, Michael Murphy, Megan Nagel, Thapas Nagarajan, Mark Nelson, Igor Otahal, Mark Pais, Selva Panchatsharam, Hassan Paraiso, Brij Patel, Natalie Pattison, Justin Pepperell, Mark Peters, Mandeep Phull, Stefania Pintus, Jagtur Singh Pooni, Frank Post, David Price, Rachel Prout, Nikolas Rae, Henrik Reschreiter, Tim Reynolds, Neil Richardson, Mark Roberts, Devender Roberts, Alistair Rose, Guy Rousseau, Brendan Ryan, Taranprit Saluja, Aarti Shah, Prad Shanmuga, Anil Sharma, Anna Shawcross, Jeremy Sizer, Manu Shankar-Hari, Richard Smith, Catherine Snelson, Nick Spittle, Nikki Staines, Tom Stambach, Richard Stewart, Pradeep Subudhi, Tamas Szakmany, Kate Tatham, Jo Thomas, Chris Thompson, Robert Thompson, Ascanio Tridente, Darell Tupper-Carey, Mary Twagira, Andrew Ustianowski, Nick Vallotton, Lisa Vincent-Smith, Shico Visuvanathan, Alan Vuylsteke, Sam Waddy, Rachel Wake, Andrew Walden, Ingeborg Welters, Tony Whitehouse, Paul Whittaker, Ashley Whittington, Meme Wijesinghe, Martin Williams, Lawrence Wilson, Sarah Wilson, Stephen Winchester, Martin Wiselka, Adam Wolverson, Daniel G. Wooton, Andrew Workman, Bryan Yates, and Peter Young.

Table 1. The LeTRS output table for known sgmRNA in the tested Nanopore ARTIC v3 primers amplicon sequencing data. “ref_leader_end” and “peak_leader_end” point to the reference position of the end of leader and the position of the end of leader identified in the most common reads (peak count) on the reference genome, and “ref_TRS_start” and “peak_TRS_start” refer to the reference position of the start of TRS and the position of the start of TRS identified in the most common reads (peak count) on the reference genome.

subgenome	ref_leader_end	peak_leader_end	ref_TRS_start	peak_TRS_start	peak_count	peak_normalized_count	cluster_count	cluster_normalized_count
S	65	65	21552	21552	980(963,343,337,0,0)	467.02(458.92,163.46,160.60,0.00,0.00)	984(967,346,340,0,0)	468.93(460.83,164.89,162.03,0.00,0.00)
ORF3a	69	69	25385	25385	76(70,51,46,0,0)	36.22(33.36,24.30,21.92,0.00,0.00)	79(73,52,47,0,0)	37.65(34.79,24.78,22.40,0.00,0.00)
E	69	69	26237	26237	268(260,41,39,0,0)	127.72(123.90,19.54,18.59,0.00,0.00)	269(261,42,40,0,0)	128.19(124.38,20.02,19.06,0.00,0.00)
M	65	65	26469	26469	15933(15731,2084,2058,0,0)	7592.89(7496.63,993.13,980.74,0.00,0.00)	16358(16153,2151,2122,0,0)	7795.43(7697.73,1025.06,1011.24,0.00,0.00)
ORF6	69	69	27041	27041	1359(1339,1334,1315,0,0)	647.63(638.10,635.72,626.66,0.00,0.00)	1377(1357,1351,1332,0,0)	656.21(646.68,643.82,634.77,0.00,0.00)
ORF7a	69	69	27388	27388	991(960,758,733,0,0)	472.26(457.49,361.23,349.31,0.00,0.00)	999(968,765,740,0,0)	476.07(461.30,364.56,352.65,0.00,0.00)
ORF7b	65	69	27644	27755	31(31,31,31,0,0)	14.77(14.77,14.77,14.77,0.00,0.00)	40(40,40,40,0,0)	19.06(19.06,19.06,19.06,0.00,0.00)
ORF8	65	65	27884	27884	19(14,5,3,0,0)	9.05(6.67,2.38,1.43,0.00,0.00)	20(15,6,4,0,0)	9.53(7.15,2.86,1.91,0.00,0.00)
N	65	65	28256	28256	15079(14916,14608,14451,0,0)	7185.92(7108.24,6961.46,6886.64,0.00,0.00)	15447(15277,14962,14799,0,0)	7361.29(7280.27,7130.16,7052.48,0.00,0.00)
ORF10	65	0	29530	0	0	0	0	0

The numbers in the bracket are (reads with left primers, reads with right primers, reads with both primers, reads with > 1 poly A, reads with > 5 poly A).

Normalized count=(Read count-Total number of read mapped on reference genome)*1000000.

Total number of read mapped on reference genome is 2098410, excluding the mapped reads not primary alignment and supplementary alignment.

Table 2. The LeTRS output table for novel sgmRNA in the tested Nanopore ARTIC v3 primers amplicon sequencing data. “leader_end” and “TRS_start” refer to the position of the end of leader and the position of the start of TRS identified in the reads >10.

subgenome	leader_end	TRS_start	nb_count	normalized_count
1	74	21055	15(13,15,13,0,0)	7.15(6.20,7.15,6.20,0.00,0.00)
2	52	28249	13(13,12,12,0,0)	6.20(6.20,5.72,5.72,0.00,0.00)

The numbers in the bracket are (reads with left primers, reads with right primers, reads with both primers, same junction on paired reads with at least a primer).

Normalized count=(Read count/Total number of read mapped on reference genome)*1000000.

Total number of read mapped on reference genome is 2098410, excluding the mapped reads unpaired, not primary alignment and supplementary alignment.

Table 3. The LeTRS output table for known sgmRNA in the tested Nanopore direct RNA sequencing data. "ref_leader_end" and "peak_leader_end" point to the reference position of the end of leader and the position of the end of leader identified in the most common reads (peak count) on the reference genome, and "ref_TRS_start" and "peak_TRS_start" refer to the reference position of the start of TRS and the position of the start of TRS identified in the most common reads (peak count) on the reference genome.

subgenome	ref_leader_end	peak_leader_end	ref_TRS_start	peak_TRS_start	peak_count	peak_normalized_count	cluster_count	cluster_normalized_count
S	65	65	21552	21552	6788(6174,2523)	11792.99(10726.27,4383.28)	6804(6188,2530)	11820.79(10750.60,4395.44)
ORF3a	69	69	25385	25385	22067(20772,8642)	38337.65(36087.81,15014.00)	22877(21523,8958)	39744.89(37392.55,15563.00)
E	69	69	26237	26237	1628(1549,645)	2828.37(2691.12,1120.58)	1650(1568,653)	2866.59(2724.13,1134.48)
M	65	65	26469	26469	44139(41659,17295)	76683.99(72375.42,30047.12)	44694(42179,17509)	77648.21(73278.83,30418.90)
ORF6	69	69	27041	27041	6469(6155,2412)	11238.79(10693.26,4190.44)	6634(6312,2474)	11525.44(10966.02,4298.15)
ORF7a	69	69	27388	27388	36409(34564,13697)	63254.44(60049.06,23796.20)	36830(34956,13872)	63985.85(60730.10,24100.24)
ORF7b	65	69	27644	27755	271(258,100)	470.82(448.23,173.73)	510(485,194)	886.04(842.60,337.04)
ORF8	65	65	27884	27884	7321(6976,2755)	12718.99(12119.61,4786.34)	7400(7047,2781)	12856.24(12242.96,4831.51)
N	65	65	28256	28256	84729(80939,32275)	147202.20(140617.72,56072.31)	85768(81918,32650)	149007.29(142318.57,56723.81)
ORF10	65	0	29530	0	0	0	0	0

The numbers in the bracket are (reads with > 1 poly A, reads with > 5 poly A).

Normalized count=(Read count/Total number of read mapped on reference genome)*1000000.

Total number of read mapped on reference genome is 575596, excluding mapped reads on reverse strand, not primary alignment and supplementary alignment.

Table 4. The LeTRS output table for novel sgmRNA in the tested Nanopore direct RNA sequencing data. “leader_end” and “TRS_start” refer to the position of the end of leader and the position of the start of TRS identified in the reads >10.

subgenome	leader_end	TRS_start	nb_count	normalized_count
1	52	26469	40(36,13)	69.49(62.54,22.59)
2	52	28249	99(90,36)	172.00(156.36,62.54)
3	52	28256	138(127,50)	239.75(220.64,86.87)
4	60	21045	16(16,6)	27.80(27.80,10.42)
5	65	22273	17(16,5)	29.53(27.80,8.69)
6	65	22488	12(11,5)	20.85(19.11,8.69)
7	65	24777	24(23,11)	41.70(39.96,19.11)
8	65	27479	51(48,18)	88.60(83.39,31.27)
9	69	21053	17(14,6)	29.53(24.32,10.42)
10	69	26292	14(14,4)	24.32(24.32,6.95)
11	69	28284	14(14,7)	24.32(24.32,12.16)
12	69	29112	21(21,6)	36.48(36.48,10.42)
13	69	29152	11(11,4)	19.11(19.11,6.95)

The numbers in the bracket are (reads with > 1 poly A, reads with > 5 poly A).

Normalized count=(Read count/Total number of read mapped on reference genome)*1000000.

Total number of read mapped on reference genome is 575596, excluding mapped reads on reverse strand, not primary alignment and supplementary alignment.

Table 5. The LeTRS output table for known sgmRNA in the negative control of the tested nanopore direct RNA sequencing data. “ref_leader_end” and “peak_leader_end” point to the reference position of the end of leader and the position of the end of leader identified in the most common reads (peak count) on the reference genome, and “ref_TRS_start” and “peak_TRS_start” refer to the reference position of the start of TRS and the position of the start of TRS identified in the most common reads (peak count) on the reference genome.

subgenome	ref_leader_end	peak_leader_end	ref_TRS_start	peak_TRS_start	peak_count	peak_normalized_count	cluster_count	cluster_normalized_count
S	65	0	21552	0	0	0	0	0
ORF3a	69	0	25385	0	0	0	0	0
E	69	0	26237	0	0	0	0	0
M	65	0	26469	0	0	0	0	0
ORF6	69	0	27041	0	0	0	0	0
ORF7a	69	0	27388	0	0	0	0	0
ORF7b	65	0	27644	0	0	0	0	0
ORF8	65	0	27884	0	0	0	0	0
N	65	0	28256	0	0	0	0	0
ORF10	65	0	29530	0	0	0	0	0

The numbers in the bracket are (reads with > 1 poly A, reads with > 5 poly A).

Normalized count=(Read count/Total number of read mapped on reference genome)*1000000.

Total number of read mapped on reference genome is 0, excluding mapped reads on reverse strand, not primary alignment and supplementary alignment.

Table 6. The LeTRS output table for novel sgmRNA in the negative control of the tested nanopore direct RNA sequencing data. “leader_end” and “TRS_start” refer to the position of the end of leader and the position of the start of TRS identified in the reads >10.

subgenome	leader_end	TRS_start	nb_count	normalized_count

The numbers in the bracket are (reads with > 1 poly A, reads with > 5 poly A).

Normalized count=(Read count/Total number of read mapped on reference genome)*1000000.

Total number of read mapped on reference genome is 0, excluding mapped reads on reverse strand, not primary alignment and supplementary alignment.

Table 7. The LeTRS output table for known sgmRNA in the tested Illumina ARTIC v3 primers amplicon sequencing data. "ref_leader_end" and "peak_leader_end" point to the reference position of the end of leader and the position of the end of leader identified in the most common reads (peak count) on the reference genome, and "ref_TRS_start" and "peak_TRS_start" refer to the reference position of the start of TRS and the position of the start of TRS identified in the most common reads (peak count) on the reference genome.

subgenome	ref_leader_end	peak_leader_end	ref_TRS_start	peak_TRS_start	peak_count	peak_normalized_count	cluster_count	cluster_normalized_count
S	65	65	21552	21552	22925(22785,16484,16368,17326)	478.71(475.79,344.21,341.79,361.79)	23312(23169,16709,16591,17418)	486.79(483.80,348.91,346.45,363.71)
ORF3a	69	69	25385	25385	5130(4784,0,0,78)	107.12(99.90,0.00,0.00,1.63)	5362(4995,0,0,82)	111.97(104.30,0.00,0.00,1.71)
E	69	69	26237	26237	5135(5014,10,7,50)	107.23(104.70,0.21,0.15,1.04)	5139(5017,10,7,50)	107.31(104.76,0.21,0.15,1.04)
M	65	64	26469	26468	12768(12177,12175,11599,8190)	266.62(254.27,254.23,242.20,171.02)	31312(30636,30026,29378,21180)	653.84(639.73,626.99,613.46,442.27)
ORF6	69	69	27041	27041	21201(20895,19094,18830,19104)	442.71(436.32,398.71,393.20,398.92)	21512(21206,19383,19119,19302)	449.20(442.81,404.75,399.23,403.06)
ORF7a	69	69	27388	27388	370(202,244,79,142)	7.73(4.22,5.10,1.65,2.97)	372(204,246,81,144)	7.77(4.26,5.14,1.69,3.01)
ORF7b	65	69	27644	27674	8(8,8,8,6)	0.17(0.17,0.17,0.17,0.13)	12(12,12,12,10)	0.25(0.25,0.25,0.25,0.21)
ORF8	65	65	27884	27884	678(675,0,0,4)	14.16(14.10,0.00,0.00,0.08)	692(689,0,0,4)	14.45(14.39,0.00,0.00,0.08)
N	65	64	28256	28255	35983(35839,35568,35443,9840)	751.38(748.37,742.71,740.10,205.47)	73700(73334,72937,72597,19878)	1538.97(1531.33,1523.04,1515.94,415.08)
ORF10	65	0	29530	0	0	0	0	0

The numbers in the bracket are (reads with left primers, reads with right primers, reads with both primers, reads with > 1 poly A, reads with > 5 poly A). Normalized count=(Read count/Total number of read mapped on reference genome)*1000000. Total number of read mapped on the

reference genome is 47889224, excluding the mapped reads not primary alignment and supplementary alignment.

Table 8. The LeTRS output table for novel sgmRNA in the tested Nanopore ARTIC v3 primers amplicon sequencing data. “leader_end” and “TRS_start” refer to the position of the end of leader and the position of the start of TRS identified in the reads >10.

subgenome	leader_end	TRS_start	nb_count	normalized_count
1	67	25800	121(118,117,114,116)	2.53(2.46,2.44,2.38,2.42)
2	68	2689	13(13,13,13,12)	0.27(0.27,0.27,0.27,0.25)
3	68	4580	144(143,107,107,118)	3.01(2.99,2.23,2.23,2.46)
4	68	5789	56(56,54,54,52)	1.17(1.17,1.13,1.13,1.09)
5	68	15777	536(511,0,0,4)	11.19(10.67,0.00,0.00,0.08)
6	68	18051	14(14,0,0,0)	0.29(0.29,0.00,0.00,0.00)
7	48	26443	151(151,149,149,146)	3.15(3.15,3.11,3.11,3.05)
8	68	21926	14(14,0,0,0)	0.29(0.29,0.00,0.00,0.00)
9	68	23020	14(14,1,1,4)	0.29(0.29,0.02,0.02,0.08)
10	68	23365	38(38,36,36,36)	0.79(0.79,0.75,0.75,0.75)
11	48	28184	11(11,0,0,0)	0.23(0.23,0.00,0.00,0.00)
12	68	26290	17(17,0,0,0)	0.35(0.35,0.00,0.00,0.00)
13	69	3816	12(12,0,0,0)	0.25(0.25,0.00,0.00,0.00)
14	69	9713	31(31,0,0,0)	0.65(0.65,0.00,0.00,0.00)
15	69	10640	14(14,4,4,6)	0.29(0.29,0.08,0.08,0.13)
16	69	13619	41(41,36,36,36)	0.86(0.86,0.75,0.75,0.75)
17	69	18554	123(123,108,108,114)	2.57(2.57,2.26,2.26,2.38)
18	49	28266	21(20,21,20,20)	0.44(0.42,0.44,0.42,0.42)
19	69	22559	104(103,0,0,2)	2.17(2.15,0.00,0.00,0.04)

20	69	25164	155(153,140,138,148)	3.24(3.19,2.92,2.88,3.09)
21	70	2794	42(42,40,40,40)	0.88(0.88,0.84,0.84,0.84)
22	70	11779	172(171,155,154,164)	3.59(3.57,3.24,3.22,3.42)
23	70	22231	139(139,137,137,136)	2.90(2.90,2.86,2.86,2.84)
24	70	22277	1241(1237,1227,1223,1218)	25.91(25.83,25.62,25.54,25.43)
25	70	22502	19(18,0,0,0)	0.40(0.38,0.00,0.00,0.00)
26	70	22945	31(29,0,0,2)	0.65(0.61,0.00,0.00,0.04)
27	70	27761	11(11,6,6,6)	0.23(0.23,0.13,0.13,0.13)
28	71	4592	47(47,36,36,40)	0.98(0.98,0.75,0.75,0.84)
29	71	5594	41(40,0,0,0)	0.86(0.84,0.00,0.00,0.00)
30	71	5708	61(5,56,0,0)	1.27(0.10,1.17,0.00,0.00)
31	71	18943	84(83,0,0,2)	1.75(1.73,0.00,0.00,0.04)
32	71	22727	35(32,33,30,30)	0.73(0.67,0.69,0.63,0.63)
33	71	25069	11(11,0,0,0)	0.23(0.23,0.00,0.00,0.00)
34	71	26401	14(14,14,14,14)	0.29(0.29,0.29,0.29,0.29)
35	71	27762	55(55,34,34,34)	1.15(1.15,0.71,0.71,0.71)
36	71	28286	24(24,24,24,24)	0.50(0.50,0.50,0.50,0.50)
37	72	4019	36(36,28,28,32)	0.75(0.75,0.58,0.58,0.67)
38	72	15808	13(13,0,0,0)	0.27(0.27,0.00,0.00,0.00)
39	73	11839	73(73,58,58,62)	1.52(1.52,1.21,1.21,1.29)
40	73	22285	173(173,172,172,172)	3.61(3.61,3.59,3.59,3.59)
41	73	23028	57(57,0,0,2)	1.19(1.19,0.00,0.00,0.04)
42	73	25856	24(24,24,24,24)	0.50(0.50,0.50,0.50,0.50)
43	74	4321	40(29,5,0,4)	0.84(0.61,0.10,0.00,0.08)
44	74	10132	30(28,28,28,28)	0.63(0.58,0.58,0.58,0.58)

45	74	12974	116(116,96,96,102)	2.42(2.42,2.00,2.00,2.13)
46	74	13555	16(16,16,16,16)	0.33(0.33,0.33,0.33,0.33)
47	74	21058	767(759,759,751,744)	16.02(15.85,15.85,15.68,15.54)
48	74	25858	29(29,28,28,28)	0.61(0.61,0.58,0.58,0.58)
49	75	21566	14(14,11,11,10)	0.29(0.29,0.23,0.23,0.21)
50	75	21572	24(24,18,18,20)	0.50(0.50,0.38,0.38,0.42)
51	75	21574	14(14,12,12,10)	0.29(0.29,0.25,0.25,0.21)
52	75	21575	29(29,18,18,18)	0.61(0.61,0.38,0.38,0.38)
53	75	21718	24(24,0,0,0)	0.50(0.50,0.00,0.00,0.00)
54	75	25135	14(9,4,0,8)	0.29(0.19,0.08,0.00,0.17)
55	75	26483	11(11,11,11,10)	0.23(0.23,0.23,0.23,0.21)
56	75	26486	95(86,93,84,88)	1.98(1.80,1.94,1.75,1.84)
57	75	26490	19(19,18,18,18)	0.40(0.40,0.38,0.38,0.38)
58	75	26491	115(115,112,112,110)	2.40(2.40,2.34,2.34,2.30)
59	75	26494	36(36,32,32,34)	0.75(0.75,0.67,0.67,0.71)
60	76	28267	31(30,27,26,18)	0.65(0.63,0.56,0.54,0.38)
61	77	25396	15(6,0,0,0)	0.31(0.13,0.00,0.00,0.00)
62	77	26481	44(44,43,43,12)	0.92(0.92,0.90,0.90,0.25)
63	78	21570	159(155,118,116,92)	3.32(3.24,2.46,2.42,1.92)
64	78	21576	41(41,20,20,28)	0.86(0.86,0.42,0.42,0.58)
65	55	26461	26(26,17,17,16)	0.54(0.54,0.35,0.35,0.33)
66	58	23922	100(14,86,0,0)	2.09(0.29,1.80,0.00,0.00)
67	60	4007	16(16,16,16,14)	0.33(0.33,0.33,0.33,0.29)
68	46	26422	53(53,52,52,50)	1.11(1.11,1.09,1.09,1.04)
69	63	698	35(16,0,0,2)	0.73(0.33,0.00,0.00,0.04)

70	47	17898	39(39,39,39,38)	0.81(0.81,0.81,0.81,0.79)
71	63	5785	144(142,142,140,140)	3.01(2.97,2.97,2.92,2.92)
72	63	6243	11(11,0,0,0)	0.23(0.23,0.00,0.00,0.00)
73	63	23142	23(19,0,0,0)	0.48(0.40,0.00,0.00,0.00)
74	63	25001	19(4,1,0,0)	0.40(0.08,0.02,0.00,0.00)
75	63	25672	11(10,0,0,0)	0.23(0.21,0.00,0.00,0.00)
76	47	26421	65(65,64,64,62)	1.36(1.36,1.34,1.34,1.29)
77	64	25795	19(8,19,8,18)	0.40(0.17,0.40,0.17,0.38)
78	65	7595	15(14,14,14,14)	0.31(0.29,0.29,0.29,0.29)
79	65	8113	82(45,44,7,80)	1.71(0.94,0.92,0.15,1.67)
80	65	10020	32(32,30,30,32)	0.67(0.67,0.63,0.63,0.67)
81	65	11200	12(12,8,8,8)	0.25(0.25,0.17,0.17,0.17)
82	65	11664	70(68,0,0,2)	1.46(1.42,0.00,0.00,0.04)
83	66	22502	22(20,0,0,0)	0.46(0.42,0.00,0.00,0.00)
84	67	3362	20(20,20,20,20)	0.42(0.42,0.42,0.42,0.42)
85	67	4018	75(75,66,66,72)	1.57(1.57,1.38,1.38,1.50)
86	67	21083	32(31,30,29,32)	0.67(0.65,0.63,0.61,0.67)

The numbers in the bracket are (reads with left primers, reads with right primers, reads with both primers, reads with > 1 poly A, reads with > 5 poly A).

Normalized count=(Read count/Total number of read mapped on reference genome)*1000000.

Total number of read mapped on reference genome is 47889224, excluding the mapped reads not primary alignment and supplementary alignment.

Table 9. The criteria of basic and advanced filtering for four different types of input data for LeTRS.

	Output Filters	Illumina paired-end reads	Nanopore cDNA reads	Nanopore RNA reads	Bam
Basic filtering	MAPQ > 10	●	●	●	●
	Read only one splicing junction	●	●	●	●
	Primary alignment only	●	●	●	●
	No supplementary alignment	●	●	●	●
	Read mapped in pair	●			
	No read reverse strand			●	
Advance filtering	Read aligment5' end includes forward primer	●	●		
	Read aligment3' end includes reverse primer	●	●		

Read aligment5' end includes forward primer and 3' end includes reverse primer	●	●	
Paired read including at least one primer in each have same leader-TRS junction in alignments	●	●	
Read aligment3' with > 1ployA		●	●
Read aligment3' with > 5ployA		●	●

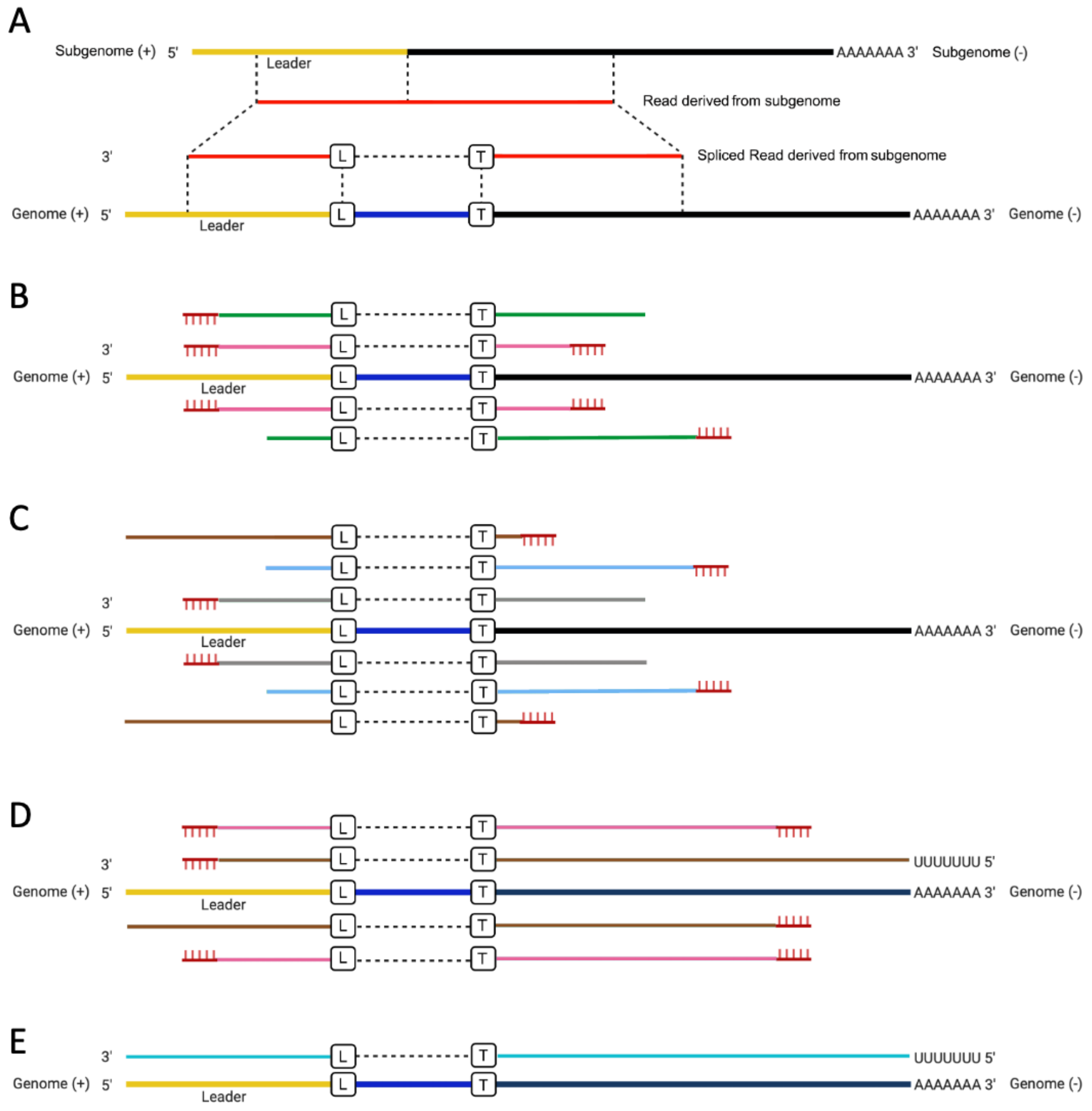
A
Subgenome (+) 5'
Leader
AAAAAAA 3'
Subgenome (-)
Read derived from subgenome
3'
L
T
Spliced Read derived from subgenome
Genome (+) 5'
Leader
AAAAAAA 3'
Genome (-)
B
C
D
UUUUUUU 5'
E

Figure 1. (A) Illustration of reads derived from sgmRNAs mapped onto the SARS-CoV-2 reference genome with a splicing method. Illustration of the possible type of reads mapped on the SARS-CoV-2 reference genome for the (B and C) paired end Illumina cDNA amplicon sequencing, where the lines with same colour implied paired reads, (D) Nanopore cDNA amplicon sequencing and (E) Nanopore direct RNA sequencing of SARS-CoV-2 genome. L and B in the boxes indicate the leader-TRS breaking sites on the leader side and TRS side, respectively.

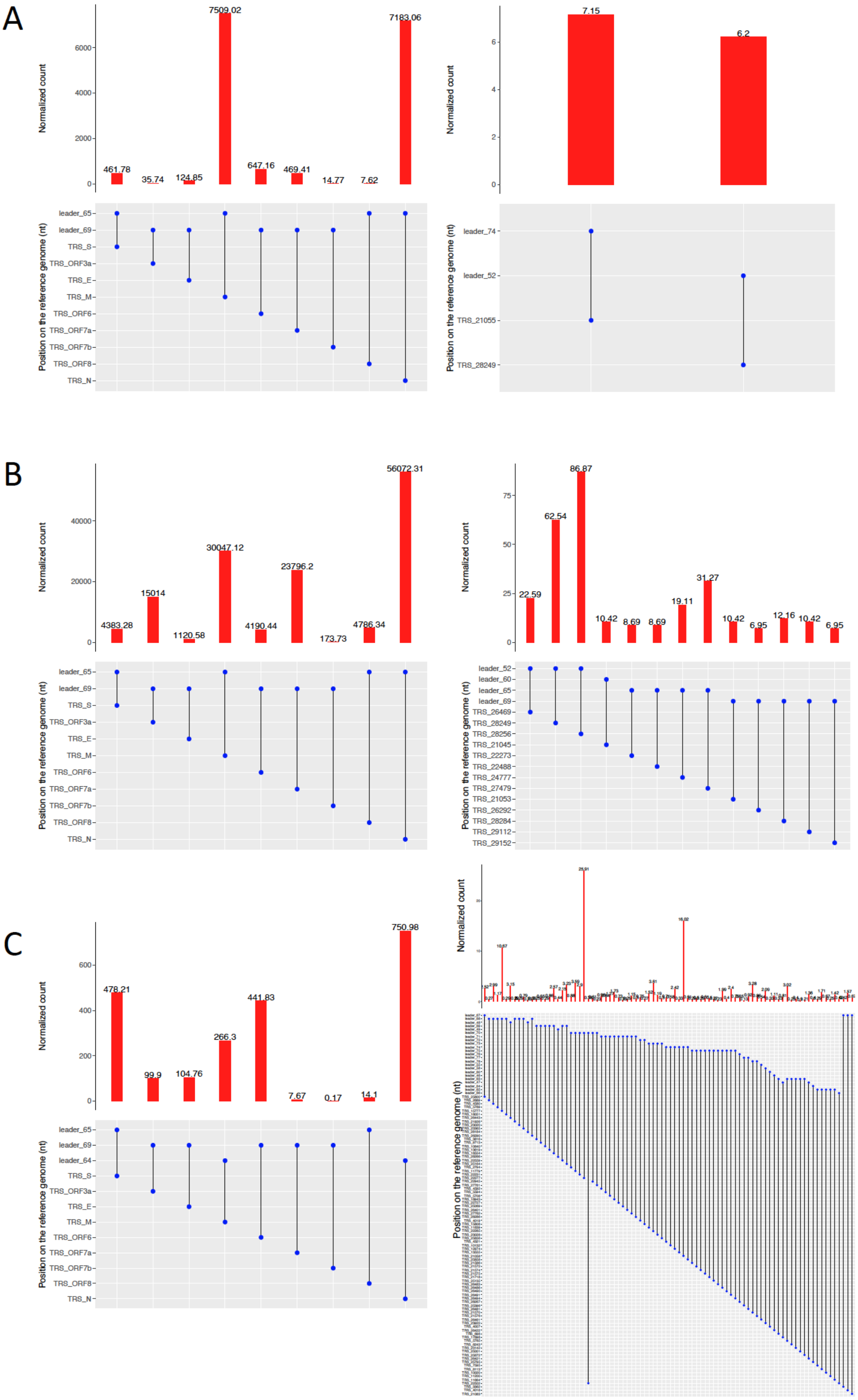
A
Normalized count
0
2000
4000
6000
461.78
35.74
124.85
7509.02
647.16
469.41
14.77
7.62
7183.06
Position on the reference genome (nt)
leader_65
leader_69
TRS_S
TRS_ORF3a
TRS_E
TRS_M
TRS_ORF6
TRS_ORF7a
TRS_ORF7b
TRS_ORF8
TRS_N
Normalized count
0
2
4
6
7.15
6.2
Position on the reference genome (nt)
leader_74
leader_52
TRS_21055
TRS_28249
B
Normalized count
0
20000
40000
4383.28
15014
1120.58
30047.12
4190.44
23796.2
173.73
4786.34
56072.31
Position on the reference genome (nt)
leader_65
leader_69
TRS_S
TRS_ORF3a
TRS_E
TRS_M
TRS_ORF6
TRS_ORF7a
TRS_ORF7b
TRS_ORF8
TRS_N
Normalized count
0
25
50
75
22.59
62.54
86.87
10.42
8.69
8.69
19.11
31.27
10.42
6.95
12.16
10.42
6.95
Position on the reference genome (nt)
leader_52
leader_60
leader_65
leader_69
TRS_26469
TRS_28249
TRS_28256
TRS_21045
TRS_22273
TRS_22488
TRS_24777
TRS_27479
TRS_21053
TRS_26292
TRS_28284
TRS_29112
TRS_29152
C
Normalized count
0
200
400
600
478.21
99.9
104.76
266.3
441.83
7.67
0.17
14.1
750.98
Position on the reference genome (nt)
leader_65
leader_69
leader_64
TRS_S
TRS_ORF3a
TRS_E
TRS_M
TRS_ORF6
TRS_ORF7a
TRS_ORF7b
TRS_ORF8
TRS_N
Normalized count
Position on the reference genome (nt)

Figure 2. Analysis of leader TRS-gene junctions of reads with at least one primer sequence at either end in sequencing data from cell culture from (A) VeroE6 cells infected with SARS-CoV-2 (England/2/2020) and sequenced using an ARTIC Nanopore approach[16] and (B) direct RNA sequencing of Cero CCL81 cells in culture infected with SARS-CoV-2 (National Culture Collection for Pathogens, Korea National Institute of Health, Korea)[2]. (C) Vero E6 cells were also infected with a near clinical isolate of known provenance and sequenced using the ARTIC Illumina approach.

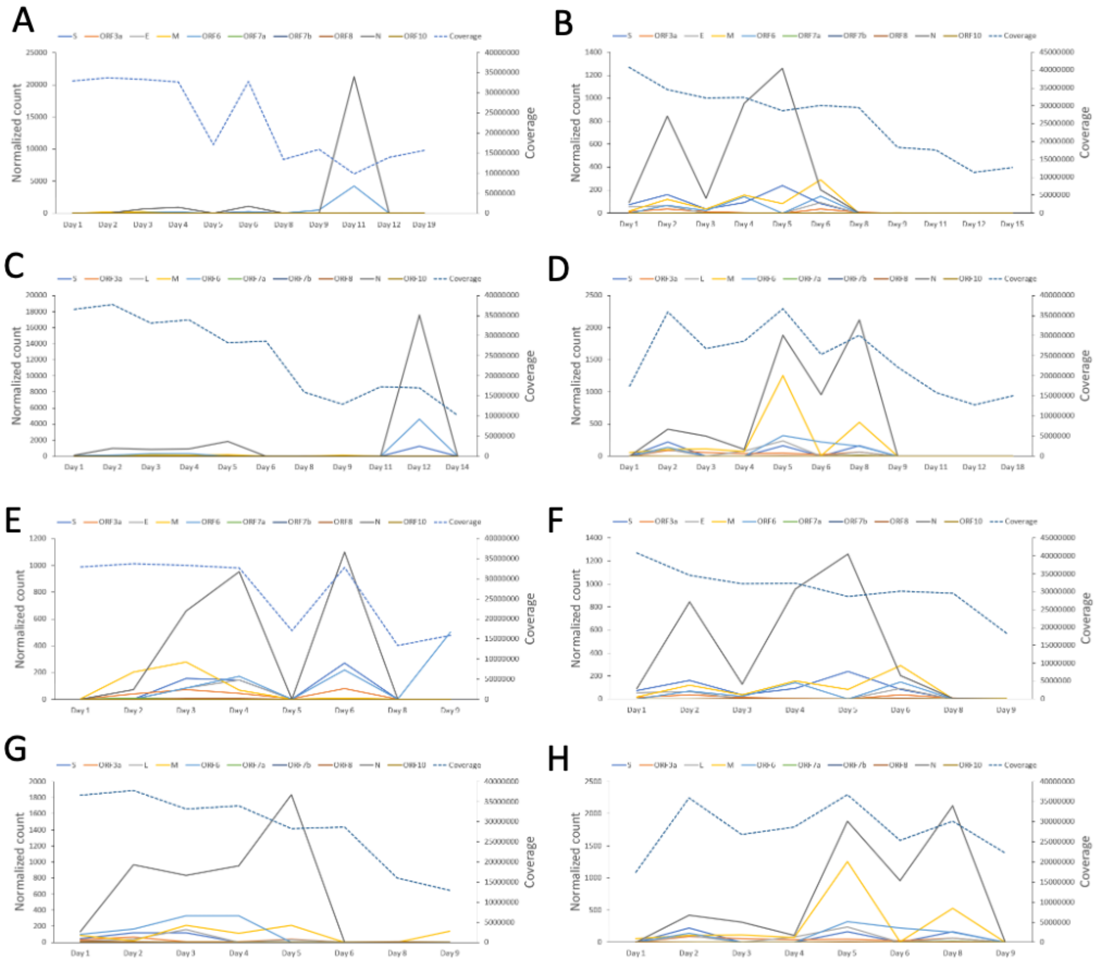

Figure 3. Analysis of leader TRS-gene junction abundance of reads with at least one primer sequence at either end in longitudinal nasopharyngeal samples (daily indicated on the x-axis) taken from two non-human primate models of SARS-CoV-2 in groups. The normalised count (Read count/total number of reads mapped on the reference genome)*1,000,000) of the leader TRS-gene junction abundance is shown on the left-hand Y-axis with each unique junction colour coded. The right-hand Y axis is a measure of the total depth of coverage for SARS-CoV-2 in that sample. Note the two scales are different. SARS-CoV-2 was amplified using the ARTIC approach and sequenced by Illumina. The data is organised into groups of animals for the cynomolgus macaque groups 1 and 2 (A/E and B/F), and rhesus macaque groups 1 and 2 (C/G and D/H). E, F,

G and H zoom in to see the details of A, B, C and D for Day1 to Day9. The data correspond to Supplementary Table 9.

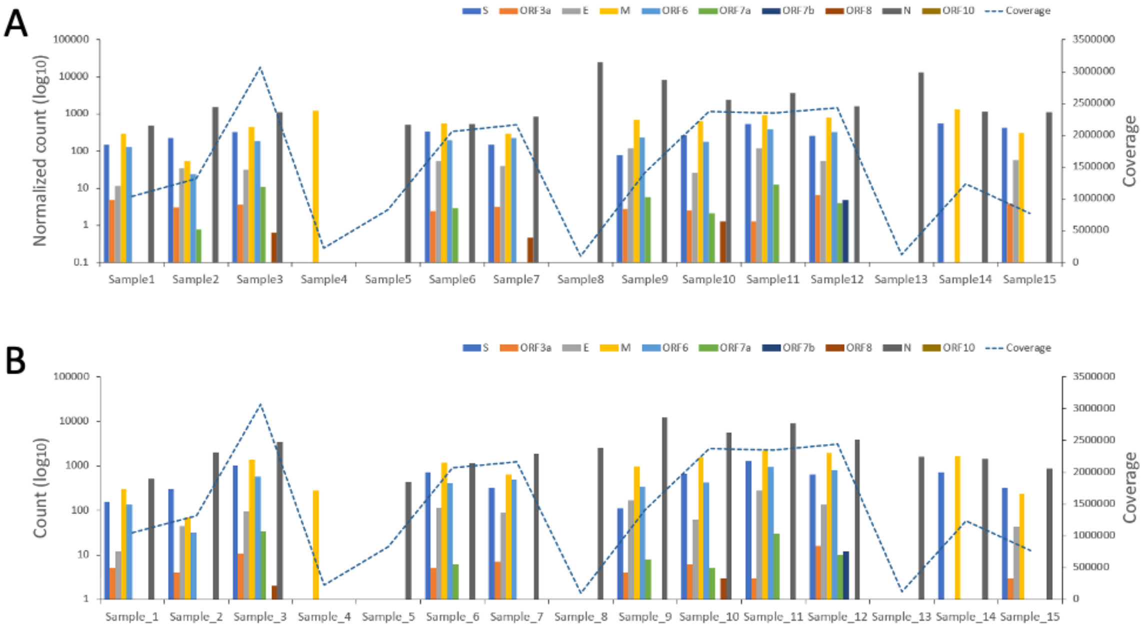

Figure 4. Plots of normalised peak counts (A) and peak counts (B) of leader-TRS gene junctions of reads with at least one primer sequences at either end derived from sequence data from 15 human patients. These were sequenced with the ARTIC pipeline via Illumina. The data correspond to Supplementary Table 7.

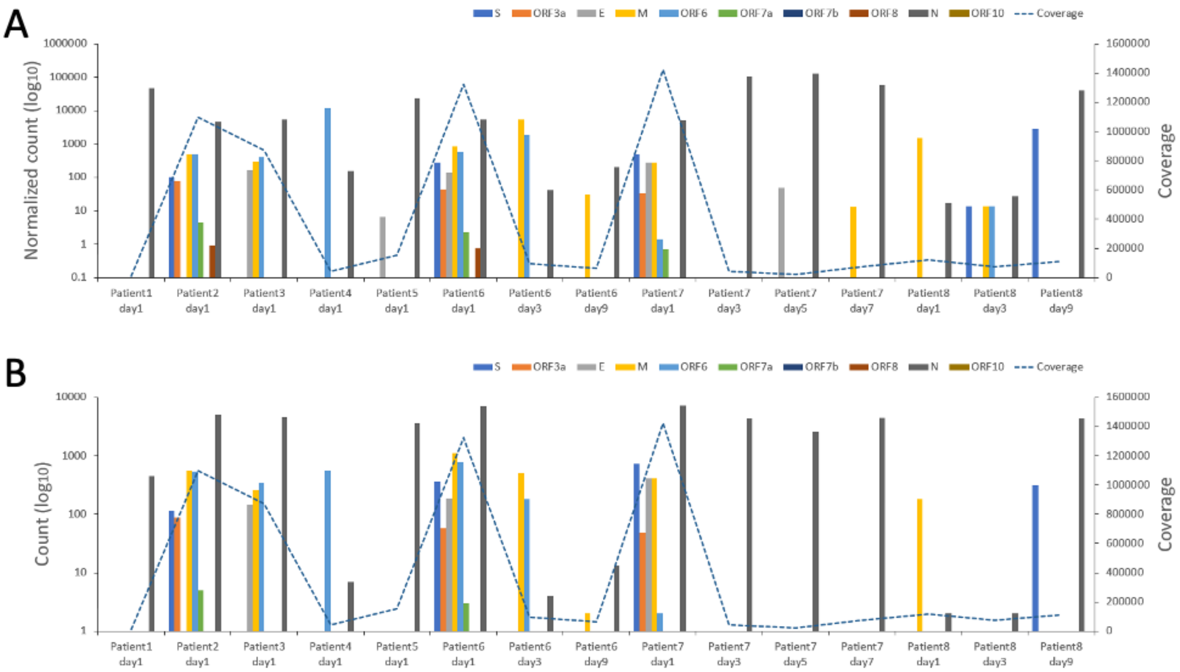

Figure 5. Plots of normalised peak counts (A) and peak counts (B) of leader-TRS gene junctions of reads with at least one primer sequence at either end derived from sequence data from 15 human patients. Some of these samples are longitudinal as indicated by the patient number and day post admission the sample was taken. These were sequenced with the ARTIC pipeline via Nanopore. The data are correspond to Supplementary Table 10.

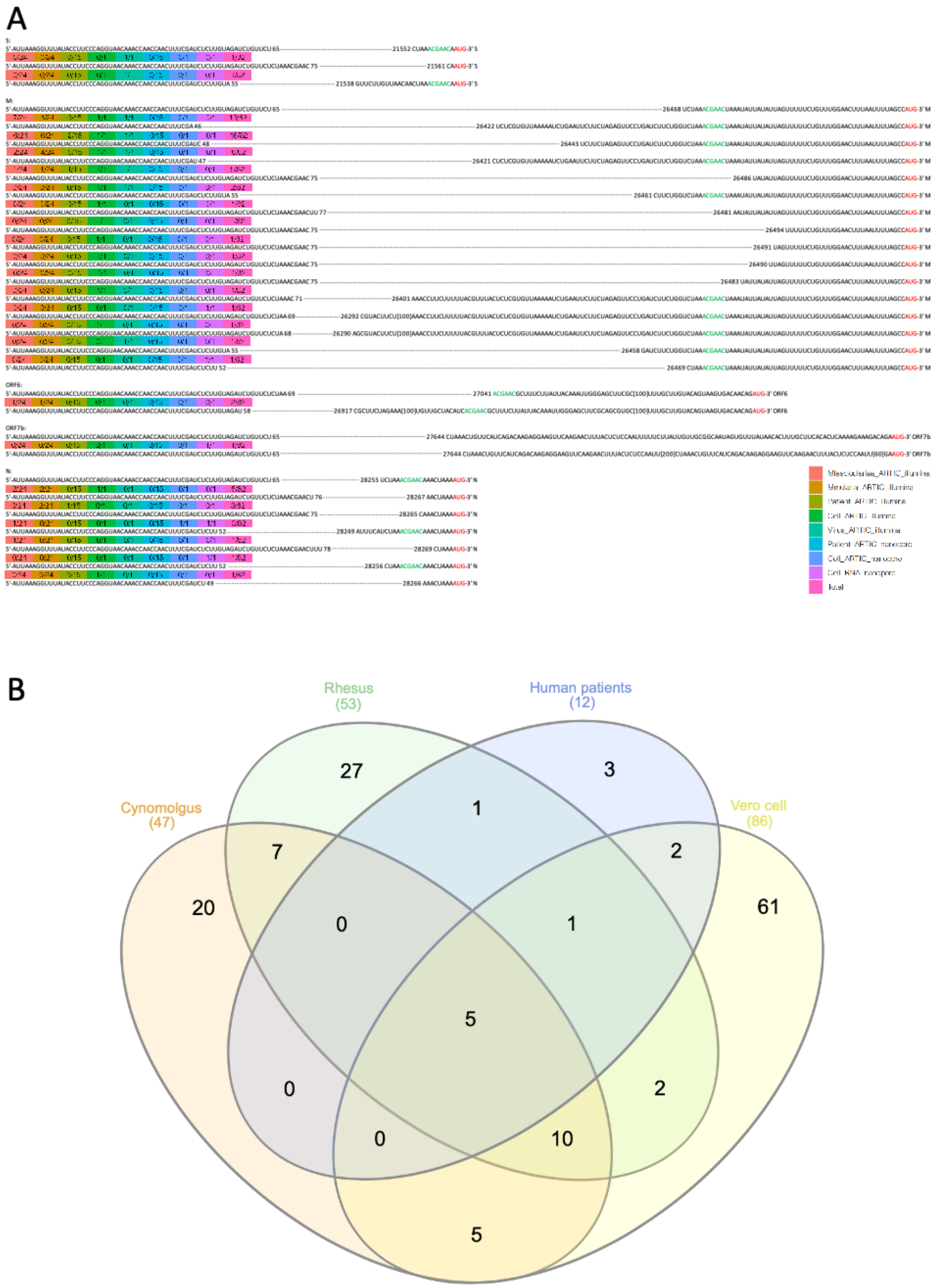

Figure 6. (A). Diagram of novel leader-TRS junctions centred around the known gene orf but out of the search interval in the analysis of cell culture, non-human primate and human sequencing data. Many novel junctions map to the leader-TRS membrane gene junctions. (B). Venn diagram showing the overlap of novel leader-TRS gene junctions among cynomolgus and rhesus macaques,

human patients, and infected Vero cells all sequenced with the ATRIC Illumina method (Supplementary Table 11).

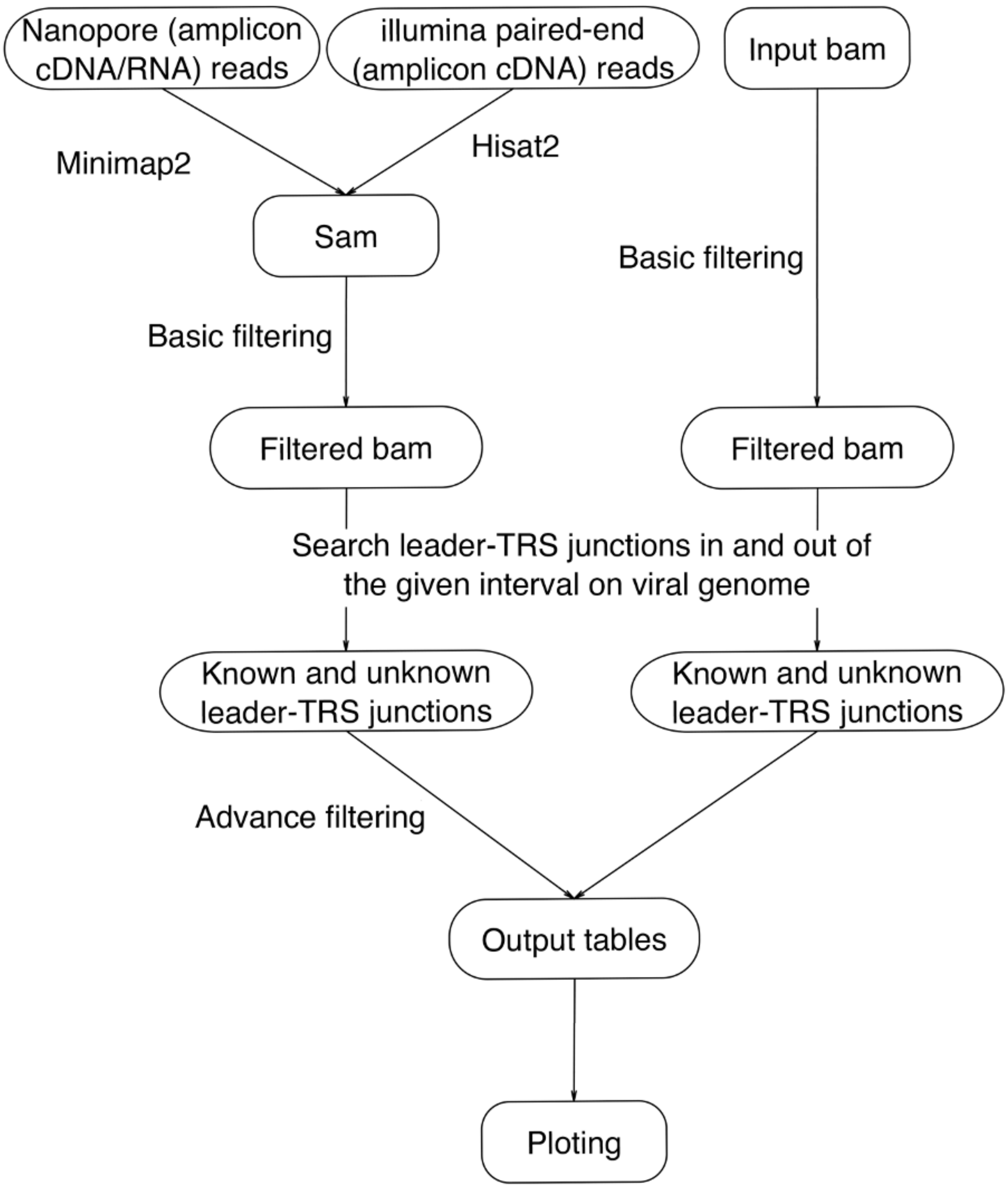

Supplementary Figure 1. Bioinformatics pipeline for the identification of leader-TRS junctions in sequencing data from SARS-CoV-2 infected material with LeTRS. This can be rapidly adapted for other coronaviruses. LeTRS can work from Nanopore or Illumina amplicon data or more unbiased approaches such as metagenomic or Illumina sequencing by using a BAM file.

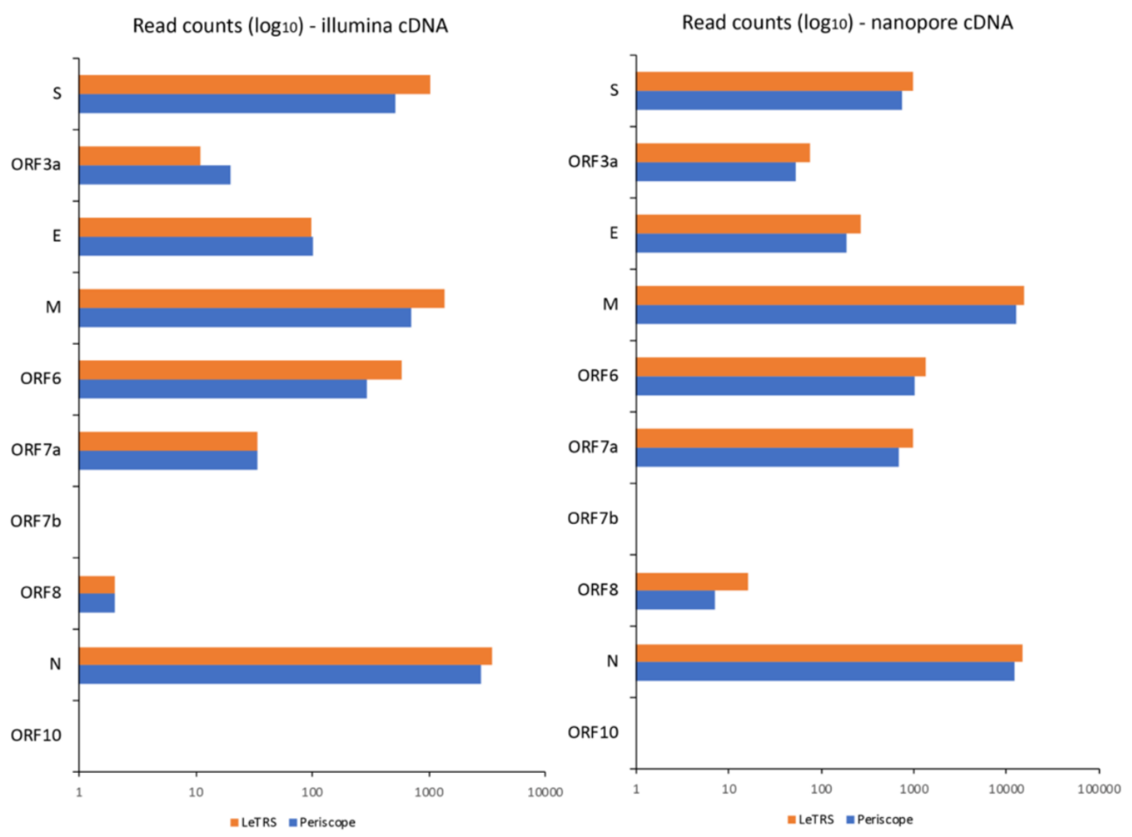

Supplementary Figure 2. Comparison of LeTRS to Periscope with the Illumina (left) and Nanopore (right) ARTIC amplicon sequencing test data sets by using the number of reads with at least one primer sequences at either end in LeTRS and the number of "High Quality" reads (the reads with both 32 nts leader sequences and known TRS-orf boundary) in Periscope.

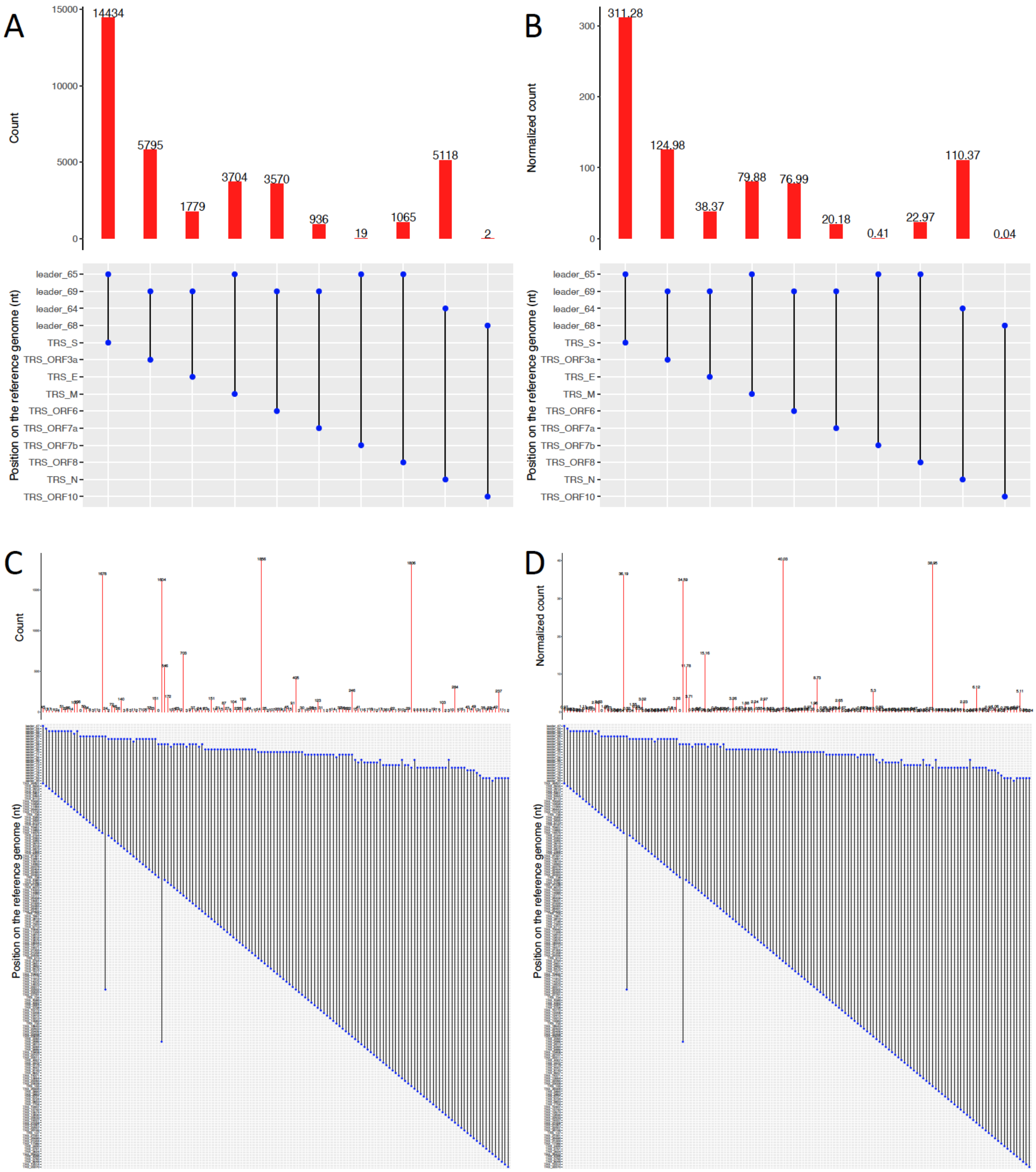

Supplementary Figure 3. Raw (A and C) and normalised (B and D) expected (upper) and novel (lower) leader-TRS gene junctions count in the infecting SARS-CoV-2 inoculum source used for NHP study, sequenced by Illumina ARTIC method (Supplementary Table 8).

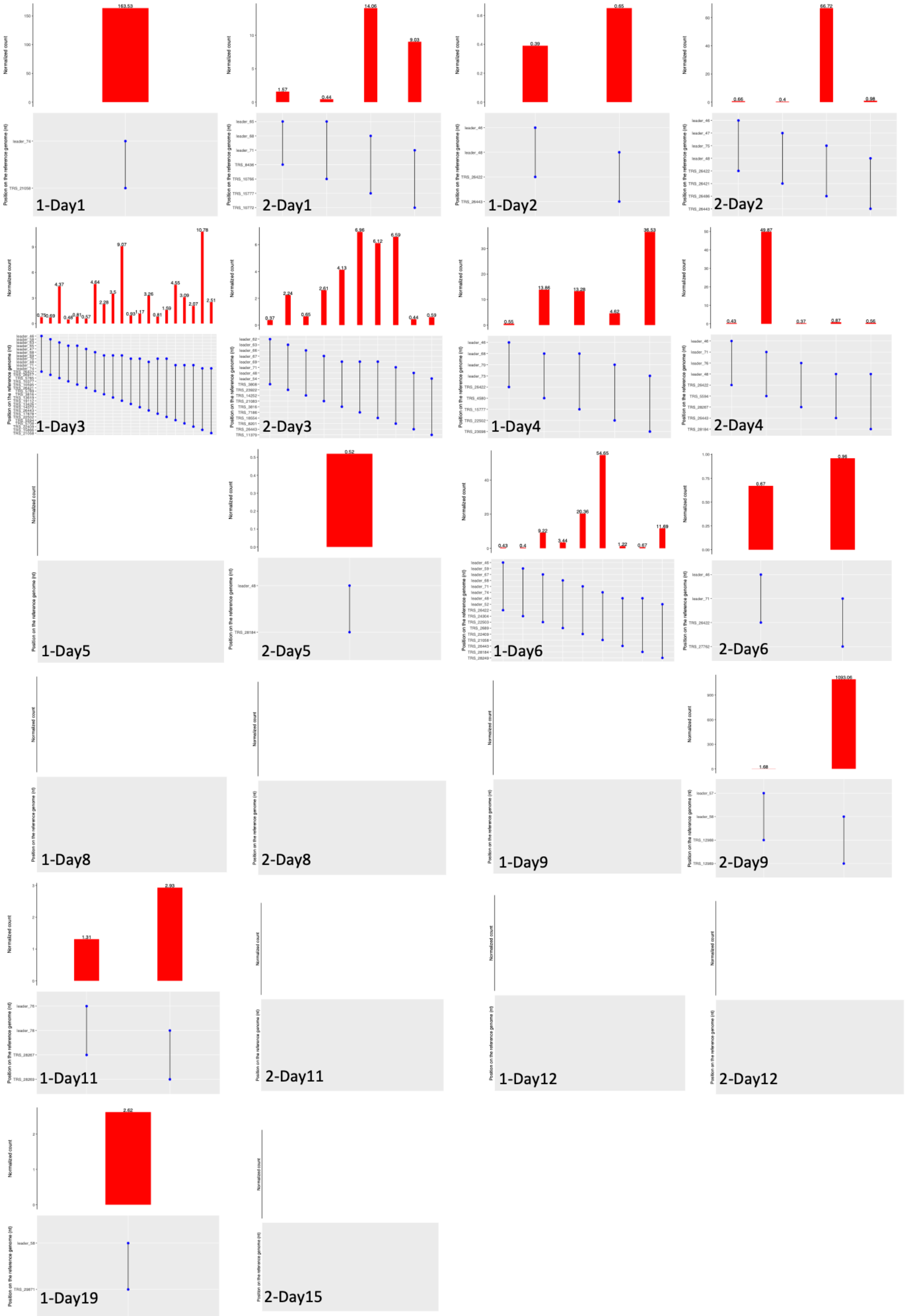
1-Day1
2-Day1
1-Day2
2-Day2
1-Day3
2-Day3
1-Day4
2-Day4
1-Day5
2-Day5
1-Day6
2-Day6
1-Day8
2-Day8
1-Day9
2-Day9
1-Day11
2-Day11
1-Day12
2-Day12
1-Day19
2-Day15

Supplementary Figure 4. Novel leader-TRS gene junctions identified for cynomolgus macaques (Supplementary Table 9). The number before "-Day" indicated the group of cynomolgus macaques.

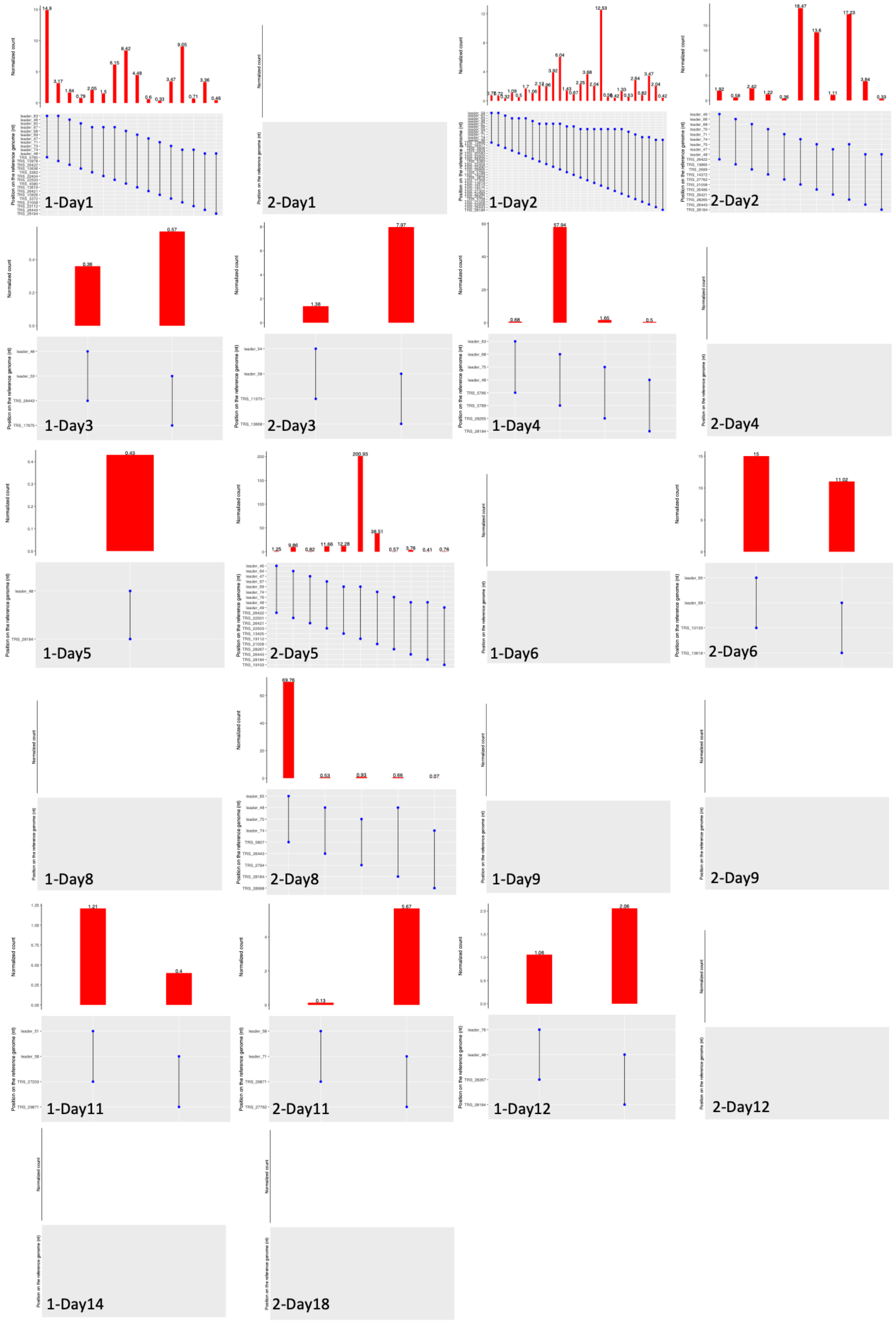
1-Day1
2-Day1
1-Day2
2-Day2
1-Day3
2-Day3
1-Day4
2-Day4
1-Day5
2-Day5
1-Day6
2-Day6
1-Day8
2-Day8
1-Day9
2-Day9
1-Day11
2-Day11
1-Day12
2-Day12
1-Day14
2-Day18

Supplementary Figure 4. Novel leader-TRS gene junctions identified for rhesus macaques (Supplementary Table 9). The number before “-Day” indicated the group of cynomolgus macaques.

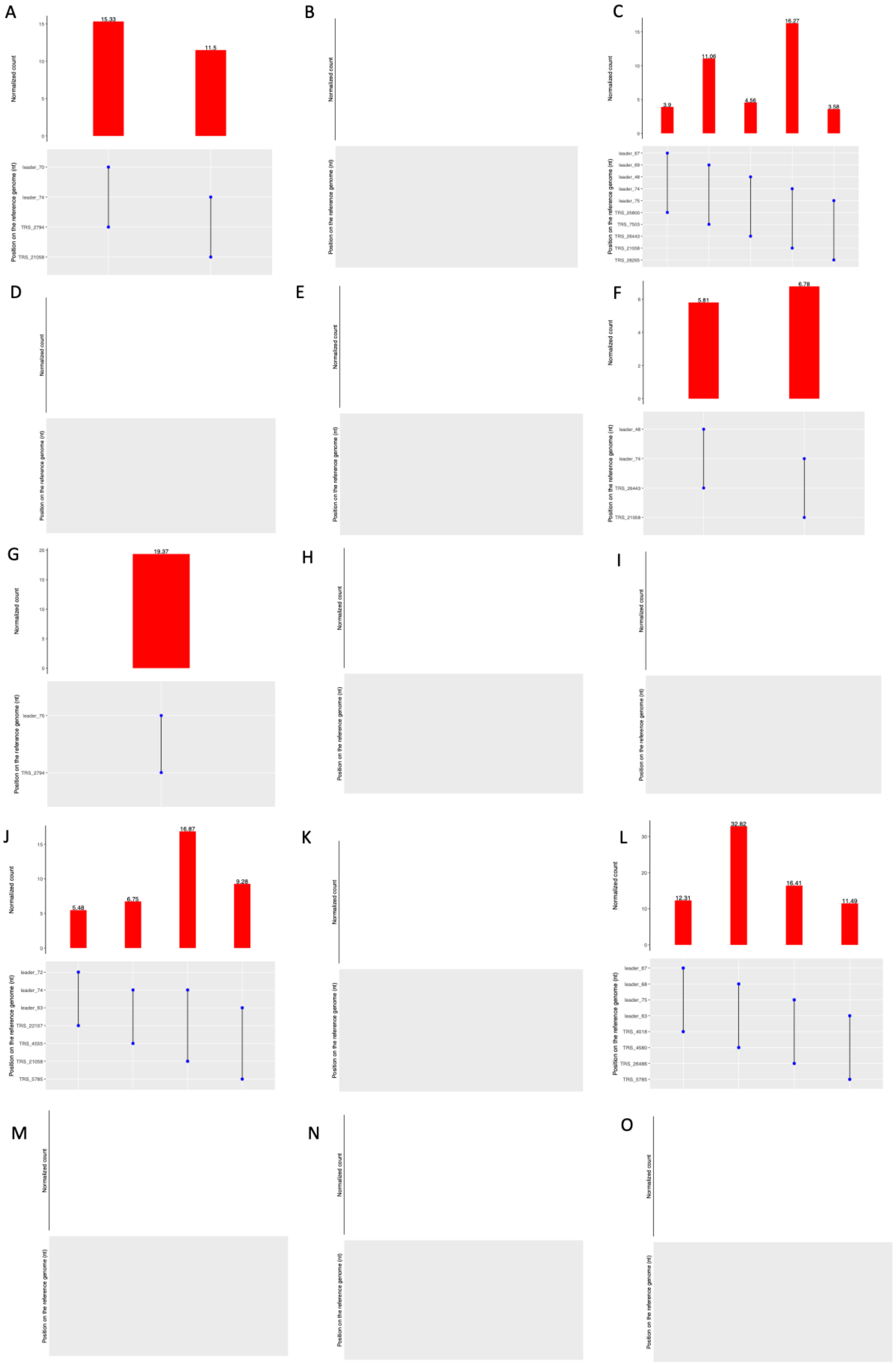
A
15.33
11.5
Normalized count
Position on the reference genome (nt)
leader_70
leader_74
TRS_2794
TRS_21058
B
Normalized count
Position on the reference genome (nt)
C
3.9
11.06
4.56
16.27
3.58
Normalized count
Position on the reference genome (nt)
leader_67
leader_69
leader_48
leader_74
leader_75
TRS_25800
TRS_7503
TRS_26443
TRS_21058
TRS_28265
D
Normalized count
Position on the reference genome (nt)
E
Normalized count
Position on the reference genome (nt)
F
5.81
6.78
Normalized count
Position on the reference genome (nt)
leader_48
leader_74
TRS_26443
TRS_21058
G
19.37
Normalized count
Position on the reference genome (nt)
leader_70
TRS_2794
H
Normalized count
Position on the reference genome (nt)
I
Normalized count
Position on the reference genome (nt)
J
5.48
6.75
16.87
9.28
Normalized count
Position on the reference genome (nt)
leader_72
leader_74
leader_63
TRS_22107
TRS_4555
TRS_21058
TRS_5785
K
Normalized count
Position on the reference genome (nt)
L
12.31
32.82
16.41
11.49
Normalized count
Position on the reference genome (nt)
leader_67
leader_68
leader_75
leader_63
TRS_4018
TRS_4580
TRS_26486
TRS_5785
M
Normalized count
Position on the reference genome (nt)
N
Normalized count
Position on the reference genome (nt)
O
Normalized count
Position on the reference genome (nt)

Supplementary Figure 5. Novel leader-TRS gene junctions identified in nasopharyngeal swabs from human patients sequenced using the ARTIC-Illumina approach (Supplementary Table 7).

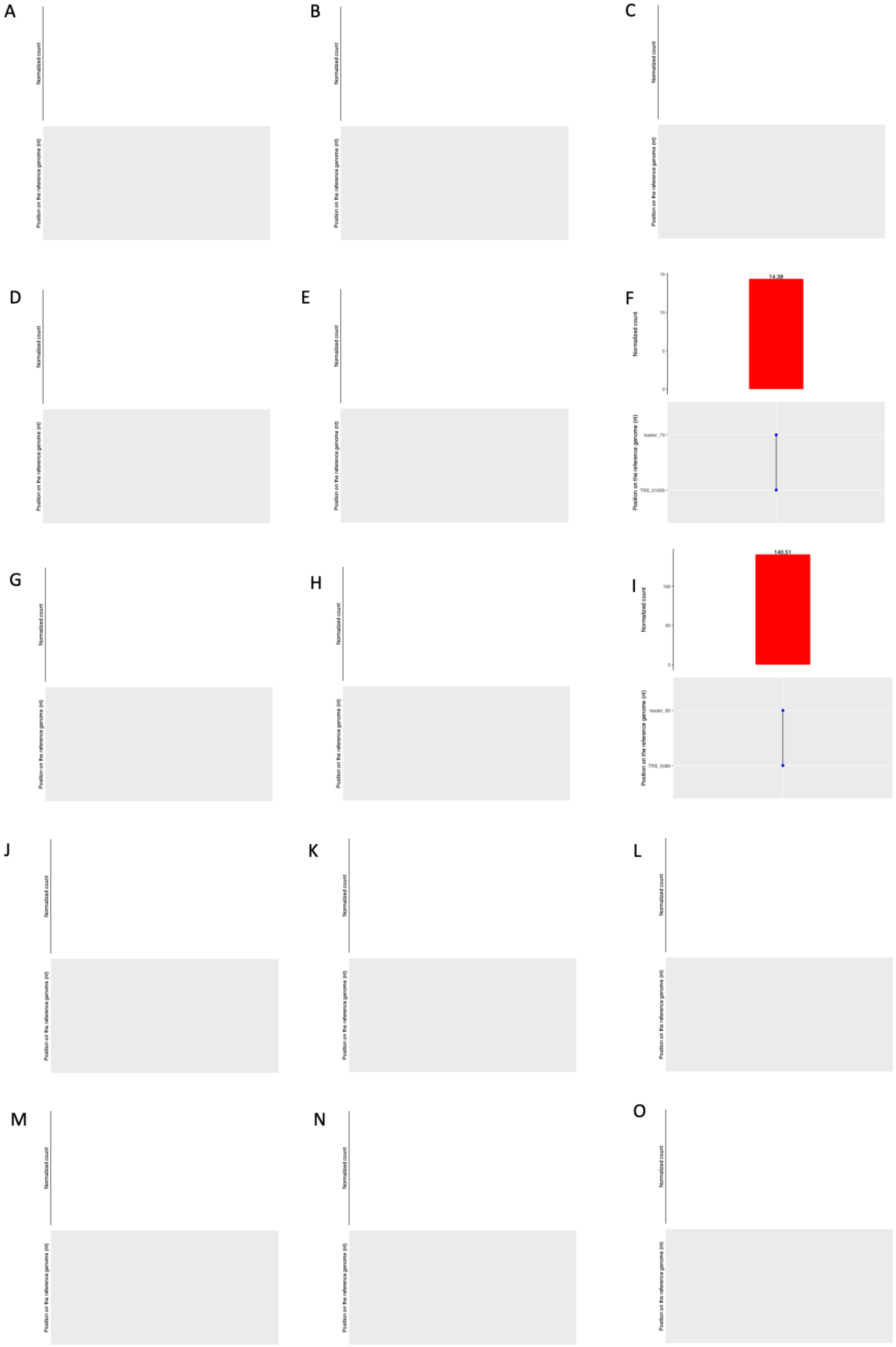
A
Normalized count
Position on the reference genome (nt)
B
Normalized count
Position on the reference genome (nt)
C
Normalized count
Position on the reference genome (nt)
D
Normalized count
Position on the reference genome (nt)
E
Normalized count
Position on the reference genome (nt)
F
14.38
15
10
5
0
Normalized count
Position on the reference genome (nt)
leader_74
TRS_21055
G
Normalized count
Position on the reference genome (nt)
H
Normalized count
Position on the reference genome (nt)
I
140.51
100
50
0
Normalized count
Position on the reference genome (nt)
leader_65
TRS_5080
J
Normalized count
Position on the reference genome (nt)
K
Normalized count
Position on the reference genome (nt)
L
Normalized count
Position on the reference genome (nt)
M
Normalized count
Position on the reference genome (nt)
N
Normalized count
Position on the reference genome (nt)
O
Normalized count
Position on the reference genome (nt)

Supplementary Figure 6. Novel leader-TRS gene junctions identified in nasopharyngeal swabs from human patients sequenced using the ARTIC-Nanopore approach (Supplementary Table 10).

Supplementary data

Table S1. The LeTRS output table for details of known sgmRNA in the tested Nanopore ARTIC v3 primers amplicon sequencing data. "peak_leader" and "peak_TRS_start" point to the leader-TRS junctions in Table 1, "ACGAAC" indicates if there is a ACGAAC sequence in the "TRS_seq" (TRS sequences), "20_leader_seq" refers to the 20 nucleotides before the end of leader, and "AUG_postion" and "first_orf_aa" refer to the first AUG position and translated orf of the sgmRNA.

Table S2. The LeTRS output table for details of novel sgmRNA in the tested Nanopore ARTIC v3 primers amplicon sequencing data. "peak_leader" and "peak_TRS_start" point to the leader-TRS junctions in Table 2, "ACGAAC" indicates if there is a ACGAAC sequences in the "TRS_seq" (TRS sequences), "20_leader_seq" refers to the 20 sequences before the end of the leader, "AUG_postion" and "first_orf_aa" refer to the first AUG position and translated orf of the sgmRNA, and "known_ATG" indicates if the first AUG position is the same as a known sgmRNA.

Table S3. The LeTRS output table for details of known sgmRNA in the tested Nanopore direct RNA sequencing data. "peak_leader" and "peak_TRS_start" point to the leader-TRS junctions in Table 3, "ACGAAC" indicates if there is a ACGAAC sequence in the "TRS_seq" (TRS sequences),

“20_leader_seq” refers to the 20 nucleotides before the end of leader, and “AUG_postion” and “first_orf_aa” refer to the first AUG position and translated orf of the sgmRNA.

Table S4. The LeTRS output table for details of novel sgmRNA in the tested nanopore direct RNA sequencing data. “peak_leader” and “peak_TRS_start” point to the leader-TRS junctions in Table 4, “ACGAAC” indicates if there is a ACGAAC sequences in the “TRS_seq” (TRS sequences), “20_leader_seq” refers to the 20 nucleotides before the end of leader, “AUG_postion” and “first_orf_aa” refer to the first AUG position and translated orf of the sgmRNA, and “known_AUG” indicates if the first AUG position is the same as a known sgmRNA.

Table S5. The LeTRS output table for details of known sgmRNA in the tested Illumina ARTIC v3 primers amplicon sequencing data. “peak_leader” and “peak_TRS_start” point to the leader-TRS junctions in Table 7, “ACGAAC” indicates if there is a ACGAAC sequence in the “TRS_seq” (TRS sequences), “20_leader_seq” refers to the 20 nucleotides before the end of leader, and “ATG_postion” and “first_orf_aa” refer to the first AUG position and translated orf of the sgmRNA.

Table S6. The LeTRS output table for details of novel sgmRNA in the tested Illumina ARTIC v3 primers amplicon sequencing data. “peak_leader” and “peak_TRS_start” point to the leader-TRS junctions in Table 8, “ACGAAC” indicates if there is a ACGAAC sequences in the “TRS_seq” (TRS sequences), “20_leader_seq” refers to the 20 nucleotides before the end of leader,

“AUG_postion” and “first_orf_aa” refer to the first AUG position and translated orf of the sgmRNA, and “known_AUG” indicates if the first AUG position same as a known sgmRNA.

Table S7. Leader-TRS gene junctions of reads with at least one primer sequence derived from sequence data from 15 human patients sequenced with the ARTIC pipeline via Illumina.

Table S8. Leader-TRS gene junction count in the infecting SARS-CoV-2 inoculum source used for the NHP study, sequenced by Illumina ARTIC method.

Table S9. Analysis of leader TRS-gene junction, abundance of reads with at least one primer sequence at either end in longitudinal nasopharyngeal samples taken from two non-human primate models (cynomolgus and rhesus macaques) of SARS-CoV-2 in groups. SARS-CoV-2 was amplified using the ARTIC approach and sequenced by Illumina. The data is organised into groups of animals for the cynomolgus macaque groups 1 and 2 that were with “-1” and “-2” in the excel sheets.

Table S10. leader-TRS gene junctions of reads with at least one primer sequence derived from sequence data from 15 human patients sequenced with the ARTIC pipeline via Nanopore.

Table S11. Novel leader-TRS junctions centred around the known gene open reading frame but out of the search interval in the analysis of cell culture, non-human primate and human sequencing data.

Supplemental tables

Click here to access/download;Table;Supplemental_Material_1.xlsx

subgenome	peak_leader_en(	peak_TRS_start	ACGAAC	ATG_postion
S	65	21552	yes	21563
ORF3a	69	25385	yes	25393
E	69	26237	yes	26245
M	65	26469	yes	26523
ORF6	69	27041	yes	27202
ORF7a	69	27388	yes	27394
ORF7b	69	27755	no	27756
ORF8	65	27884	yes	27894
N	65	28256	yes	28274

20_leader_seq	TRS_seq
ATCTCTTGTAGATCTGTTCT	CTAAACGAACA
CTTGTAGATCTGTTCTCTAA	ACGAACTT
CTTGTAGATCTGTTCTCTAA	ACGAACTT
ATCTCTTGTAGATCTGTTCT	CTAAACGAACTAAATATTATATTAGTTTTTCTGT
CTTGTAGATCTGTTCTCTAA	ACGAACGCTTTCTTATTACAAATTGGGAGCTTC
CTTGTAGATCTGTTCTCTAA	ACGAAC
CTTGTAGATCTGTTCTCTAA	A
ATCTCTTGTAGATCTGTTCT	CTAAACGAAC
ATCTCTTGTAGATCTGTTCT	CTAAACGAACAAACTAAA

first_orf_aa

MFVFLVLLPLVSSQCVNLTTRTQLPPAYTNSFTRGVYYPDKVFRSSVLHSTQDLFLPFFSNVTWFHAIHVS

MDLFMRIFTIGTVTLKQGEIKDATPSDFVRATATIPIQASLPFGWLIVGVALLAVFQSASKIITLKKRWQLA

MYSFVSEETGTLIVNSVLLFLAFVVFLLVTLAILTALRLCAYCCNIVNVSLVKPSFYVYSRVKNLNSSRVPDL

MADSNGTITVEELKKLLEQWNLVIGFLFLTWICLLQFAYANRNRFLYIIKLIFLWLLWPVTLACFVLAAVY

MFHLVDFQVTIAEILLIIMRTFKVSIWNLDYIINLIIKNLSKSLTENKYSQLDEEQPMEID*

MKIILFLALITLATCELYHYQECVRGTTVLLKEPCSSGTYEGNSPFHPLADNKFALTCFSTQFAFACPDGVK

MIELSLIDFYLCFLAFLLFLVLIMLIIFWFSLELQDHNETCHA*

MKFLVFLGIITTVAAFHQECSLQSCTQHQPYVVDDPCPIHFYSKWYIRVGARKSAPLIELCVDEAGSKSPI

MSDNGPQNQRNAPRITFGGPSDSTGSNQNGERSGARSKQRRPQGLPNNTASWFTALTQHGKEDLK

SGTNGTKRFDNPVLPFNDGVYFASTEKSNIIRGWIFGTTLDSKTQSLLIVNNATNVVIKVCEFQFCNDPFLGVY
ALSKGVHFVCNLLLLFVTVYSHLLLVAAGLEAPFLYLYALVYFLQSINFVRIIMRLWLCWKCRSKNPLLYDANYFL
LV*
RINWITGGIAIAMACLVGLMWLSYFIASFRLFARTRSMWSFNPETNILLNVPLHGTILTRPLLESELVIGAVILRG

HVYQLRARSVSPKLFIRQEEVQELYSPIFLIVAAIVFITLCFTLKRKTE*

QYIDIGNYTVSCLPFTINCQEPKLGSLVVRCSFYEDFLEYHDVRVVLDFI*
FPRGQGVPINTNSSPDDQIGYYRRATRRIRGGDGKMKDLSPRWYFYYLGTGPEAGLPYGANKDGIIWVATEG

YHKNNKSWMESEFRVYSSANNCTFEYVSQPFLMDLEGKQGNFKNLREFVFKNIDGYFKIYSKHTPINLVRDLP
CWHTNCYDYCIPYNSVTSSIVITSGDGTTSPISEHDYQIGGYTEKWESGVKDCVVLHSYFTSDYYQLYSTQLSTD

HLRIAGHHLGRCDIKDLPKEITVATSRTLSYYKLGASQRVAGDSGFAAYSRYRIGNYKLNTDHSSSSDNIALLVQ

ALNTPKDHIGTRNPANNAAIVLQLPQGTTLPKGFYAEGSRGGSQASSRSSSRSRNSSRNSTPGSSRGTSPARM

QGFSALEPLVDLPIGINITRFQTLLALHRSYLTPGDSSSGWTAGAAAYYVGYLQPRTFLLKYNENGTITDAVDC

TGVEHVTFFIYNKIVDEPEEHVQIHTIDGSSGVVNPVMEPIYDEPTTTTSVPL*

*

AGNGGDAALALLLLDRLNQLESKMSGKGQQQQGQTVTKKSAAEASKKPRQKRTATKAYNVTQAFGRRGP

ALDPLSETKCTLKSFTVEKGIYQTSNFRVQPTESIVRFPNITNLCPFGEVFNATRFASVYAWNRKRISNCVADYSV

EQTQGNFGDQELIRQGTDYKHWPQIAQFAPSASAFFGMSRIGMEVTPSGTWLTYTGAIKLDDKDPNFKDQV

VLYNSASFSTFKCYGVSPTKLNDLCFTNVYADSFVIRGDEVRQIAPGQTGKIADYNYKLPDDFTGCVIAWNSN[illegible]

[illegible]ILLNKHIDAYKTFPPTEPKKDKKKKADETQALPQRQKKQQTVTLLPAADLDDFSKQLQQSMSSADSTQA*

NLDSKVGGNYNYLYRLFRKSNLKPFERDISTEIYQAGSTPCNGVEGFNCYFPLQSYGFQPTNGVGYQPYRVVVL

_SFELLHAPATVCGPKKSTNLVKNKCVNFNFNGLTGTGVLTESNKKFLPFQQFGRDIADTTDAVRDPQTLEILD

ITPCSFGGVSVITPGTNTSNQVAVLYQDVNCTEVPVAIHADQLTPTWRVYSTGSNVFQTRAGCLIGAEHVNN

SYECDIPIGAGICASYQTQTNSPRRARSVASQSIIAYTMSLGAENSVAYSNNSIAIPTNFTISVTTEILPVSMTKTS

[illegible]VDCTMYICGDSTECSNLLLQYGSFCTQLNRALTGIAVEQDKNTQEVFAQVKQIYKTPPIKDFGGFNFSQILPD[illegible]

PSKPSKRSFIEDLLFNKVTLADAGFIKQYGDCLGDIAARDLICAQKFNGLTVLPPLLTDEMIAQYTSALLAGTITS[illegible]

GWTFGAGAALQIPFAMQMAYRFNGIGVTQNVLYENQKLIANQFNSAIGKIQDSLSSTASALGKLQDVVNQN

AQALNTLVKQLSSNFGAISSVLNDILSRLDKVEAEVQIDRLITGRLQSLQTYVTQQLIRAAEIRASANL

subgenome	peak_leader_	peak_TRS_st	ACGAAC	ATG_postion	known_ATG
1	74	21055	no	21071	-
2	52	28249	yes	28274	N

20_leader_seq	TRS_seq	first_orf_aa
AGATCTGTTCTCTAAACGAA	GACCCTAAGACTAAAA	MLQKKMTLKRVFSLTFVGLYNKS*
CAACCAACTTTCGATCTCTT	ATTTCATCTAAACGAACAAACT[illegible]	MSDNGPQNQRNAPRITFGGPSDS[illegible]

TGSNQNGERSGARSKQRRPQGLPNNTASWFTALTQHGKEDLKFPRGQGVPINTNSSPDDQIGYYRRATRRI[illegible]

RGGDGKMKDLSPRWYFYYLGTGPEAGLPYGANKDGIIWVATEGALNTPKDHIGTRNPANNAAIVLQLPQGT

TLPKGFYAEGSRGGSQASSRSSSRSRNSSRNSTPGSSRGTSPARMAGNGGDAALALLLLDRLNQLESKMSGK

[illegible]GQQQQGQTVTKKSAAEASKKPRQKRTATKAYNVTQAFGRRGPEQTQGNFGDQELIRQGTDYKHWPQIAQ

[illegible]FAPSASAFFGMSRIGMEVTPSGTWLTYTGAIKLDDKDPNFKDQVILLNKHIDAYKTFPPTEPKKDKKKKADET

QALPQRQKKQQTVTLLPAADLDDFSKQLQQSMSSADSTQA*

subgenome	peak_leader_	peak_TRS_sta	ACGAAC	ATG_postion
S	65	21552	yes	21563
ORF3a	69	25385	yes	25393
E	69	26237	yes	26245
M	65	26469	yes	26523
ORF6	69	27041	yes	27202
ORF7a	69	27388	yes	27394
ORF7b	69	27755	no	27756
ORF8	65	27884	yes	27894
N	65	28256	yes	28274

20_leader_seq	TRS_seq	first_orf_aa
ATCTCTTGTAGATCTGTTCT	CTAAACGAACA	MFVFLVLLPLVSSQCVNLTTRTQLPP
CTTGTAGATCTGTTCTCTAA	ACGAACTT	MDLFMRIFTIGTVTLKQGEIKDATPS
CTTGTAGATCTGTTCTCTAA	ACGAACTT	MYSFVSEETGTLIVNSVLLFLAFVVFLL
ATCTCTTGTAGATCTGTTCT	CTAAACGAACTAAATATTATATTAG	MADSNGTITVEELKKLLEQWNLVIGF
CTTGTAGATCTGTTCTCTAA	ACGAACGCTTTCTTATTACAAATTG	MFHLVDFQVTIAEILLIIMRTFKVSIW
CTTGTAGATCTGTTCTCTAA	ACGAAC	MKIILFLALITLATCELYHYQECVRGTT
CTTGTAGATCTGTTCTCTAA	A	MIELSLIDFYLCFLAFLLFLVLIMLIIFW
ATCTCTTGTAGATCTGTTCT	CTAAACGAAC	MKFLVFLGIITTVAAFHQECSLQSCTQ
ATCTCTTGTAGATCTGTTCT	CTAAACGAACAAACTAAA	MSDNGPQNQRNAPRITFGGPSDST

YTNSFTRGVYYPDKVFRSSVLHSTQDLFLPFFSNVTWFHAIHVSGTNGTKRFDNPVLPFNDGVYFASTEK

FVRATATIPIQASLPFGWLIVGVALLAVFQSASKIITLKKRWQLALSKGVHFVCNLLLLFVTVYSHLLLVAA

VTLAILTALRLCAYCCNIVNVSLVKPSFYVYSRVKNLNSSRVPDLLV*

LFLTWICLLQFAYANRNRFLYIIKLIFLWLLWPVTLACFVLAAVYRINWITGGIAIAMACLVGLMWLSYFIA

LDYIINLIIKNLSKSLTENKYSQLDEEQPMEID*

VLLKEPCSSGTYEGNSPFHPLADNKFALTCFSTQFAFACPDGVKHVYQLRARSVSPKLFIRQEEVQELYSPI

SLELQDHNETCHA*

HQPYVVDDPCPIHFYSKWYIRVGARKSAPLIELCVDEAGSKSPIQYIDIGNYTVSCLPFTINCQEPKLGSLV

SNQNGERSGARSKQRRPQGLPNNTASWFTALTQHGKEDLKFPRGQGVPINTNSSPDDQIGYYRRATR

SNIIRGWIFGTTLDSKTQSLLIVNNATNVVIKVCEFQFCNDPFLGVYYHKNNKSWMESEFRVYSSANNCTFEYV
GLEAPFLYLYALVYFLQSINFVRIIMRLWLCWKCRSKNPLLYDANYFLCWHTNCYDYCIPYNSVTSSIVITSGDGTT

SFRLFARTRSMWSFNPETNILLNVPLHGTILTRPLLESELVIGAVILRGHLRIAGHHLGRCDIKDLPKEITVATSRTLS

FLIVAAIVFITLCFTLKRKTE*

RCSFYEDFLEYHDVRVVLDFI*
RIRGGDGKMKDLSPRWYFYYLGTGPEAGLPYGANKDGIIWVATEGALNTPKDHIGTRNPANNAAIVLQLPQG

SQPFLMDLEGKQGNFKNLREFVFKNIDGYFKIYSKHTPINLVRDLPQGFSALEPLVDLPIGINITRFQTLLALHRSY
TSPISEHDYQIGGYTEKWESGVKDCVVLHSYFTSDYYQLYSTQLSTDTGVEHVTFFIYNKIVDEPEEHVQIHTIDGS

SYYKLGASQRVAGDSGFAAYSRYRIGNYKLNTDHSSSSDNIALLVQ*

TTLPKGFYAEGSRGGSQASSRSSSRSRNSSRNSTPGSSRGTSPARMAGNGGDAALALLLLDRLNQLESKMSGK[illegible]

[illegible]LTPGDSSSGWTAGAAAYYVGYLQPRTFLLKYNENGTITDAVDCALDPLSETKCTLKSFTVEKGIYQTSNFRVQP[illegible]
[illegible]SSGVVNPVMEPIYDEPTTTTSVPL*

[illegible]GQQQQGQTVTKKSAAEASKKPRQKRTATKAYNVTQAFGRRGPEQTQGNFGDQELIRQGTDYKHWPQIAQF[illegible]

TESIVRFPNITNLCPFGEVFNATRFASVYAWNRKRISNCVADYSVLYNSASFSTFKCYGVSPTKLNDLCFTNVYA[illegible]

APSASAFFGMSRIGMEVTPSGTWLTYTGAIKLDDKDPNFKDQVILLNKHIDAYKTFPPTEPKKDKKKKADETQA

[illegible]SFVIRGDEVRQIAPGQTGKIADYNYKLPDDFTGCVIAWNSNNLDSKVGGNYNYLYRLFRKSNLKPFERDISTEI[illegible]

[illegible]LPQRQKKQQTVTLLPAADLDDFSKQLQQSMSSADSTQA*

YQAGSTPCNGVEGFNCYFPLQSYGFQPTNGVGYQPYRVVVLSFELLHAPATVCGPKKSTNLVKNKCVNFNFN[illegible]

GLTGTGVLTESNKKFLPFQQFGRDIADTTDAVRDPQTLEILDITPCSFGGVSVITPGTNTSNQVAVLYQDVNCTE

EVPVAIHADQLTPTWRVYSTGSNVFQTRAGCLIGAEHVNNSYECDIPIGAGICASYQTQTNSPRRARSVASQSII[illegible]

AYTMSLGAENSVAYSNNSIAIPTNFTISVTTEILPVSMTKTSVDCTMYICGDSTECSNLLLQYGSFCTQLNRALTG

IAVEQDKNTQEVFAQVKQIYKTPPIKDFGGFNFSQILPDPSKPSKRSFIEDLLFNKVTLADAGFIKQYGDCLGDIA

ARDLICAQKFNGLTVLPPLLTDEMIAQYTSALLAGTITSGWTFGAGAALQIPFAMQMAYRFNGIGVTQNVLYE

NQKLIANQFNSAIGKIQDSLSSTASALGKLQDVVNQNAQALNTLVKQLSSNFGAISSVLNDILSRLDKVEAEVQI

DRLITGRLQSLQTYVTQQLIRAAEIRASANL

subgenome	peak_leader_	peak_TRS_sta	ACGAAC	ATG_postion	known_ATG
1	52	26469	yes	26523	M
2	52	28249	yes	28274	N
3	52	28256	yes	28274	N
4	60	21045	no	21049	-
5	65	22273	no	22359	-
6	65	22488	no	22614	-
7	65	24777	no	24810	-
8	65	27479	no	27756	ORF7b
9	69	21053	no	21071	-
10	69	26292	yes	26523	M
11	69	28284	no	28284	-
12	69	29112	no	29222	-
13	69	29152	no	29222	-

20_leader_seq	TRS_seq	first_orf_aa
CAACCAACTTTCGATCTCTT	CTAAACGAACTAAATA	MADSNGTITVEELKKLLEQWNLVIGFLFLTWICLLQFAYAN
CAACCAACTTTCGATCTCTT	ATTTCATCTAAACGAA	MSDNGPQNQRNAPRITFGGPSDSTGSNQNGERSGARSK
CAACCAACTTTCGATCTCTT	CTAAACGAACAAACTA	MSDNGPQNQRNAPRITFGGPSDSTGSNQNGERSGARSK
TTTCGATCTCTTGTAGATCT	TGAT	MYDPKTKNVTKENDSKEGFFTYICGFIQQKLALGGSVAIKI
ATCTCTTGTAGATCTGTTCT	GTTTCAAACTTTACTT	MWVIFNLGLFY*
ATCTCTTGTAGATCTGTTCT	AAAAAGGAATCTATCA	MLGTGRESATVLLIILSYIIPHHFPLLSVMECLLLN*
ATCTCTTGTAGATCTGTTCT	AAAAGAACTTCACAA	MMEKHTFLVKVSLFQMAHTGL*
ATCTCTTGTAGATCTGTTCT	TACTTTTAAAAGAACC	MIELSLIDFYLCFLAFLLFLVLIMLIIFWFSLELQDHNETCHA*
CTTGTAGATCTGTTCTCTAA	ACGACCCTAAGACTAA	MLQKKMTLKRVFSLTFVGLYNKS*
CTTGTAGATCTGTTCTCTAA	CGTACTTCTTTTTCTTG	MADSNGTITVEELKKLLEQWNLVIGFLFLTWICLLQFAYAN
CTTGTAGATCTGTTCTCTAA	-	MDPKISEMHPALRLVDPQIQLAVTRMENAVGRDQNNV
CTTGTAGATCTGTTCTCTAA	AACAAACCCAAGGAA	MSRIGMEVTPSGTWLTYTGAIKLDDKDPNFKDQVILLNK
CTTGTAGATCTGTTCTCTAA	ACAAGGAACTGATTA	MSRIGMEVTPSGTWLTYTGAIKLDDKDPNFKDQVILLNK

IRNRFLYIIKLIFLWLLWPVTLACFVLAAVYRINWITGGIAIAMACLVGLMWLSYFIASFRLFARTRSMWSFNPE

QRRPQGLPNNTASWFTALTQHGKEDLKFPRGQGVPINTNSSPDDQIGYYRRATRRIRGGDGKMKDLSPRW

QRRPQGLPNNTASWFTALTQHGKEDLKFPRGQGVPINTNSSPDDQIGYYRRATRRIRGGDGKMKDLSPRW

TEHSWNADLYKLMGHFAWWTAFVTNVNASSSEAFLIGCNYLGKPREQIDGYVMHANYIFWRNTNPIQLSSY

IRNRFLYIIKLIFLWLLWPVTLACFVLAAVYRINWITGGIAIAMACLVGLMWLSYFIASFRLFARTRSMWSFNPE

GPKVYPIILRLGSPLSLNMARKTLNSLEDKAFQLTPIAVQMTKLATTEELPDEFVVVTVK*

HIDAYKTFPPTEPKKDKKKKADETQALPQRQKKQQTVTLLPAADLDDFSKQLQQSMSSADSTQA*

HIDAYKTFPPTEPKKDKKKKADETQALPQRQKKQQTVTLLPAADLDDFSKQLQQSMSSADSTQA*

TNILLNVPLHGTILTRPLLESELVIGAVILRGHLRIAGHHLGRCDIKDLPKEITVATSRTLSYYKLGASQRVAGDSG
YFYYLGTGPEAGLPYGANKDGIIWVATEGALNTPKDHIGTRNPANNAAIVLQLPQGTTLPKGFYAEGSRGGS
YFYYLGTGPEAGLPYGANKDGIIWVATEGALNTPKDHIGTRNPANNAAIVLQLPQGTTLPKGFYAEGSRGGS
SLFDMSKFPLKLRGTAVMSLKEGQINDMILSLLSKGRLIIRENNRVVISSDVLVNN*

TNILLNVPLHGTILTRPLLESELVIGAVILRGHLRIAGHHLGRCDIKDLPKEITVATSRTLSYYKLGASQRVAGDSG

FAAYSRYRIGNYKLNTDHSSSSDNIALLVQ*

ASSRSSSRSRNSSRNSTPGSSRGTSPARMAGNGGDAALALLLLDRLNQLESKMSGKGQQQQGQTVTKKSA

ASSRSSSRSRNSSRNSTPGSSRGTSPARMAGNGGDAALALLLLDRLNQLESKMSGKGQQQQGQTVTKKSA

FAAYSRYRIGNYKLNTDHSSSSDNIALLVQ*

AEASKKPRQKRTATKAYNVTQAFGRRGPEQTQGNFGDQELIRQGTDYKHWPQIAQFAPSASAFFGMSRIG[illegible]
AEASKKPRQKRTATKAYNVTQAFGRRGPEQTQGNFGDQELIRQGTDYKHWPQIAQFAPSASAFFGMSRIG[illegible]

MEVTPSGTWLTYTGAIKLDDKDPNFKDQVILLNKHIDAYKTFPPTEPKKDKKKKADETQALPQRQKKQQTVTL
MEVTPSGTWLTYTGAIKLDDKDPNFKDQVILLNKHIDAYKTFPPTEPKKDKKKKADETQALPQRQKKQQTVTL

[illegible]LPAADLDDFSKQLQQSMSSADSTQA*
[illegible]LPAADLDDFSKQLQQSMSSADSTQA*

subgenome	peak_leader_	peak_TRS_st	ACGAAC	ATG_postion	20_leader_seq
S	65	21552	yes	21563	ATCTCTTGTAGATCTGTT
ORF3a	69	25385	yes	25393	CTTGTAGATCTGTTCTCT
E	69	26237	yes	26245	CTTGTAGATCTGTTCTCT
M	64	26468	yes	26523	GATCTCTTGTAGATCTGT
ORF6	69	27041	yes	27202	CTTGTAGATCTGTTCTCT
ORF7a	69	27388	yes	27394	CTTGTAGATCTGTTCTCT
ORF7b	69	27674	no	27756	CTTGTAGATCTGTTCTCT
ORF8	65	27884	yes	27894	ATCTCTTGTAGATCTGTT
N	64	28255	yes	28274	GATCTCTTGTAGATCTGT

TRS_seq	first_orf_aa
CTAAACGAACA	MFVFLVLLPLVSSQCVNLTTRTQLPPAYTNSFTRGVYYPDKVFRSSVLHSTQDLFL
ACGAACTT	MDLFMRIFTIGTVTLKQGEIKDATPSDFVRATATIPIQASLPFGWLIVGVALLAVF
ACGAACTT	MYSFVSEETGTLIVNSVLLFLAFVVFLLVTLAILTALRLCAYCCNIVNVSLVKPSFYV
TCTAAACGAACTAAATATTATATT	MADSNGTITVEELKKLLEQWNLVIGFLFLTWICLLQFAYANRNRFLYIIKLIFLWLL
ACGAACGCTTTCTTATTACAAATT	MFHLVDFQVTIAEILLIIMRTFKVSIWNLDYIINLIIKNLSKSLTENKYSQLDEEQPM
ACGAAC	MKIILFLALITLATCELYHYQECVRGTTVLLKEPCSSGTYEGNSPFHPLADNKFALT
AAGAACTTTACTCTCCAATTTTTC	MIELSLIDFYLCFLAFLLFLVLIMLIIFWFSLELQDHNETCHA*
CTAAACGAAC	MKFLVFLGIITTVAAFHQECSLQSCTQHQPYVVDDPCPIHFYSKWYIRVGARKSA
TCTAAACGAACAAACTAAA	MSDNGPQNQRNAPRITFGGPSDSTGSNQNGERSGARSKQRRPQGLPNNTAS

PFFSNVTWFHAIHVSGTNGTKRFDNPVLPFNDGVYFASTEKSNIIRGWIFGTTLDSKTQSLLIVNNATNVVIKV
QSASKIITLKKRWQLALSKGVHFVCNLLLLFVTVYSHLLLVAAGLEAPFLYLYALVYFLQSINFVRIIMRLWLCWK
YSRVKNLNSSRVPDLLV*
WPVTLACFVLAAVYRINWITGGIAIAMACLVGLMWLSYFIASFRLFARTRSMWSFNPETNILLNVPLHGTILTF
EID*
CFSTQFAFACPDGVKHVYQLRARSVSPKLFIRQEEVQELYSPIFLIVAAIVFITLCFTLKRKTE*

PLIELCVDEAGSKSPIQYIDIGNYTVSCLPFTINCQEPKLGSLVVRCSFYEDFLEYHDVRVVLDFI*
WFTALTQHGKEDLKFPRGQGVPINTNSSPDDQIGYYRRATRRIRGGDGKMKDLSPRWYFYYLGTGPEAGLPY

CEFQFCNDPFLGVYYHKNNKSWMESEFRVYSSANNCTFEYVSQPFLMDLEGKQGNFKNLREFVFKNIDGYF
CRSKNPLLYDANYFLCWHTNCYDYCIPYNSVTSSIVITSGDGTTSPISEHDYQIGGYTEKWESGVKDCVVLHSY

PLLESELVIGAVILRGHLRIAGHHLGRCDIKDLPKEITVATSRTLSYYKLGASQRVAGDSGFAAYSRYRIGNYKLN

GANKDGIIWVATEGALNTPKDHIGTRNPANNAAIVLQLPQGTTLPKGFYAEGSRGGSQASSRSSSRSRNSSR

KIYSKHTPINLVRDLPQGFSALEPLVDLPIGINITRFQTLLALHRSYLTPGDSSSGWTAGAAAYYVGYLQPRTFLL
FTSDYYQLYSTQLSTDTGVEHVTFFIYNKIVDEPEEHVQIHTIDGSSGVVNPVMEPIYDEPTTTTSVPL*

ITDHSSSSDNIALLVQ*

NSTPGSSRGTSPARMAGNGGDAALALLLLDRLNQLESKMSGKGQQQQGQTVTKKSAAEASKKPRQKRTAT[illegible]

[illegible]KYNENGTITDAVDCALDPLSETKCTLKSFTVEKGIYQTSNFRVQPTESIVRFPNITNLCPFGEVFNATRFASVYA[illegible]

[illegible]AYNVTQAFGRRGPEQTQGNFGDQELIRQGTDYKHWPQIAQFAPSASAFFGMSRIGMEVTPSGTWLTYTG[illegible]

WNRKRISNCVADYSVLYNSASFSTFKCYGVSPTKLNDLCFTNVYADSFVIRGDEVRQIAPGQTGKIADYNYKLP

AIKLDDKDPNFKDQVILLNKHIDAYKTFPPTEPKKDKKKKADETQALPQRQKKQQTVTLLPAADLDDFSKQLQ

DDFTGCVIAWNSNNLDSKVGGNYNYLYRLFRKSNLKPFERDISTEIYQAGSTPCNGVEGFNCYFPLQSYGFQ

QSMSSADSTQA*

[illegible]TNGVGYQPYRVVVLSFELLHAPATVCGPKKSTNLVKNKCVNFNFNGLTGTGVLTESNKKFLPFQQFGRDIAD

TTDAVRDPQTLEILDITPCSFGGVSVITPGTNTSNQVAVLYQDVNCTEVPVAIHADQLTPTWRVYSTGSNVFC

QTRAGCLIGAEHVNNSYECDIPIGAGICASYQTQTNSPRRARSVASQSIIAYTMSLGAENSVAYSNNSIAIPTNFT

TISVTTEILPVSMTKTSVDCTMYICGDSTECSNLLLQYGSFCTQLNRALTGIAVEQDKNTQEVFAQVKQIYKTPP

IKDFGGFNFSQILPDPSKPSKRSFIEDLLFNKVTLADAGFIKQYGDCLGDIAARDLICAQKFNGLTVLPPLLTDEM

MIAQYTSALLAGTITSGWTFGAGAALQIPFAMQMAYRFNGIGVTQNVLYENQKLIANQFNSAIGKIQDSLSSTA

ASALGKLQDVVNQNAQALNTLVKQLSSNFGAISSVLNDILSRLDKVEAEVQIDRLITGRLQSLQTYVTQQLIRAA

AEIRASANL

subgenome	peak_leader_	peak_TRS_st	ACGAAC	ATG_postion	known_ATG
1	67	25800	no	25814	-
2	68	2689	no	2745	-
3	68	4580	no	4590	-
4	68	5789	no	5859	-
5	68	15777	no	15812	-
6	68	18051	no	18153	-
7	48	26443	yes	26523	M
8	68	21926	no	21936	-
9	68	23020	no	23046	-
10	68	23365	no	23403	-
11	48	28184	no	28207	-
12	68	26290	yes	26523	M
13	69	3816	no	3842	-
14	69	9713	no	9768	-
15	69	10640	no	10662	-
16	69	13619	no	13625	-
17	69	18554	no	18594	-
18	49	28266	no	28274	N
19	69	22559	no	22614	-
20	69	25164	no	25179	-
21	70	2794	no	2802	-
22	70	11779	no	11849	-
23	70	22231	no	22359	-
24	70	22277	no	22359	-
25	70	22502	no	22614	-
26	70	22945	no	23004	-
27	70	27761	no	27825	-
28	71	4592	no	4607	-
29	71	5594	no	5681	-
30	71	5708	no	5718	-
31	71	18943	no	18956	-
32	71	22727	no	22743	-
33	71	25069	no	25080	-
34	71	26401	yes	26523	M
35	71	27762	no	27825	-
36	71	28286	no	28305	-
37	72	4019	no	4044	-
38	72	15808	no	15812	-
39	73	11839	no	11849	-
40	73	22285	no	22359	-
41	73	23028	no	23046	-
42	73	25856	no	25910	-

43	74	4321	no	4350	-
44	74	10132	no	10152	-
45	74	12974	no	12986	-
46	74	13555	no	13556	-
47	74	21058	no	21071	-
48	74	25858	no	25910	-
49	75	21566	no	21744	-
50	75	21572	no	21744	-
51	75	21574	no	21744	-
52	75	21575	no	21744	-
53	75	21718	no	21744	-
54	75	25135	no	25143	-
55	75	26483	no	26523	M
56	75	26486	no	26523	M
57	75	26490	no	26523	M
58	75	26491	no	26523	M
59	75	26494	no	26523	M
60	76	28267	no	28274	N
61	77	25396	no	25405	-
62	77	26481	no	26523	M
63	78	21570	no	21744	-
64	78	21576	no	21744	-
65	55	26461	yes	26523	M
66	58	23922	no	24051	-
67	60	4007	no	4044	-
68	46	26422	yes	26523	M
69	63	698	no	726	-
70	47	17898	no	17906	-
71	63	5785	no	5859	-
72	63	6243	no	6243	-
73	63	23142	no	23173	-
74	63	25001	no	25050	-
75	63	25672	no	25718	-
76	47	26421	yes	26523	M
77	64	25795	no	25814	-
78	65	7595	no	7665	-
79	65	8113	no	8133	-
80	65	10020	no	10070	-
81	65	11200	no	11219	-
82	65	11664	no	11697	-
83	66	22502	no	22614	-
84	67	3362	no	3375	-
85	67	4018	no	4044	-

86	67	21083	no	21086	-

20_leader_seq	TRS_seq
CTCTTGTAGATCTGTTCTCT	AAACCCATTACTTT
TCTTGTAGATCTGTTCTCTA	AACAAACAATACCTTCACACTCAAAGGCGGT
TCTTGTAGATCTGTTCTCTA	AACGATCTAA
TCTTGTAGATCTGTTCTCTA	AAAGAAACTTTGTATTGCATAGACGGTGCTT
TCTTGTAGATCTGTTCTCTA	AAAGAACTTTAAGTCAGTTCTTTATTATCAAA
TCTTGTAGATCTGTTCTCTA	AACAGGACTCTTTAAAGATTGTAGTAAGGTA
AAACCAACCAACTTTCGATC	TCTTCTAGAGTTCCTGATCTTCTGGTCTAAAC
TCTTGTAGATCTGTTCTCTA	AACGCTACTA
TCTTGTAGATCTGTTCTCTA	TAATTGTTACTTTCCTTTACAATCAT
TCTTGTAGATCTGTTCTCTA	AACAAATACTTCTAACCAGGTTGCTGTTCTTT
AAACCAACCAACTTTCGATC	TCTTGTAGTGCGTTGTTCGTTCT
TCTTGTAGATCTGTTCTCTA	AGCGTACTTCTTTTTCTTGCTTTCGTGGTATT
CTTGTAGATCTGTTCTCTAA	ACAAACTTGTTTCAAGCTTTTTGGAA
CTTGTAGATCTGTTCTCTAA	ACAAAGCATTTCTATTGGTTCTTTAGTAATTA
CTTGTAGATCTGTTCTCTAA	ACGGACACAACTATTACAGTTA
CTTGTAGATCTGTTCTCTAA	ACGAAG
CTTGTAGATCTGTTCTCTAA	ACACACTTAAAAATCTCTCTGACAGAGTCGT
AACCAACCAACTTTCGATCT	AAACTAAA
CTTGTAGATCTGTTCTCTAA	ACAAACTTGTGCCCTTTTGGTGAAGTTTTTAA
CTTGTAGATCTGTTCTCTAA	AAGAACTTGGAAAGT
TTGTAGATCTGTTCTCTAAA	TGAACTTG
TTGTAGATCTGTTCTCTAAA	CAAACTCAACATTAAATTGTTGGGTGTTGGT
TTGTAGATCTGTTCTCTAAA	AGAACCATTGGTAGATTTGCCAATAGGTATT
TTGTAGATCTGTTCTCTAAA	CAAACTTTACTTGCTTTACATAGAAGTTATTT
TTGTAGATCTGTTCTCTAAA	CAAACTTCTAACTTTAGAGTCCAACCAACAG
TTGTAGATCTGTTCTCTAAA	CAAACCTTTTGAGAGAGATATTTCAACTGAA
TTGTAGATCTGTTCTCTAAA	TGAACTTTCATTAATTGACTTCTATTTGTGCT
TGTAGATCTGTTCTCTAAAC	GAAACTCTTGTTACA
TGTAGATCTGTTCTCTAAAC	GAACAATTTAAGAAAGGTGTTCAGATACCTT
TGTAGATCTGTTCTCTAAAC	GAACTTAAGC
TGTAGATCTGTTCTCTAAAC	GAACTGAAGATTA
TGTAGATCTGTTCTCTAAAC	GATCTCTGCTTTACTA
TGTAGATCTGTTCTCTAAAC	CTCTGGCATTA
TGTAGATCTGTTCTCTAAAC	AAACCTTCTTTTTACGTTTACTCTCGTGTTAA
TGTAGATCTGTTCTCTAAAC	GAACTTTCATTAATTGACTTCTATTTGTGCTT
TGTAGATCTGTTCTCTAAAC	GGACCCCAAAATCAGCGAA
GTAGATCTGTTCTCTAAACG	AACTTGTTACTTTATATTGACATTA
GTAGATCTGTTCTCTAAACG	AACA
TAGATCTGTTCTCTAAACGA	ACAGTCTAAA
TAGATCTGTTCTCTAAACGA	ACTTGCTTTACATAGAAGTTATTTGACTCCTG
TAGATCTGTTCTCTAAACGA	ACTTTCCTTTACAATCAT
TAGATCTGTTCTCTAAACGA	ACTATTGTATACCTTACAATAGTGTAACTTCT

AGATCTGTTCTCTAAACGAA	CTTTTACATTCTACCATCTATTATCTCTA
AGATCTGTTCTCTAAACGAA	ACTTAACGGTCTTTGGCTTG
AGATCTGTTCTCTAAACGAA	CTAAATAGAGGT
AGATCTGTTCTCTAAACGAA	A
AGATCTGTTCTCTAAACGAA	CCTAAGACTAAAA
AGATCTGTTCTCTAAACGAA	TATTGTATACCTTACAATAGTGTAACTTCTTC
GATCTGTTCTCTAAACGAAC	TTTGTTTTTCTTGTTTTATTGCCACTAGTCTCT
GATCTGTTCTCTAAACGAAC	TTTCTTGTTTTATTGCCACTAGTCTCTAGTCAG
GATCTGTTCTCTAAACGAAC	TCTTGTTTTATTGCCACTAGTCTCTAGTCAGT
GATCTGTTCTCTAAACGAAC	CTTGTTTTATTGCCACTAGTCTCTAGTCAGTG
GATCTGTTCTCTAAACGAAC	GGACTTGTTCTTACCTTTCTTTTCCA
GATCTGTTCTCTAAACGAAC	GAATTTAA
GATCTGTTCTCTAAACGAAC	TATTATATTAGTTTTTCTGTTTGGAACTTTAAT
GATCTGTTCTCTAAACGAAC	TATATTAGTTTTTCTGTTTGGAACTTTAATTTT
GATCTGTTCTCTAAACGAAC	TTAGTTTTTCTGTTTGGAACTTTAATTTTAGC
GATCTGTTCTCTAAACGAAC	TAGTTTTTCTGTTTGGAACTTTAATTTTAGCC
GATCTGTTCTCTAAACGAAC	TTTTTCTGTTTGGAACTTTAATTTTAGCC
ATCTGTTCTCTAAACGAACT	AACTAAA
TCTGTTCTCTAAACGAACTT	GATTTGTTT
TCTGTTCTCTAAACGAACTT	AATATTATATTAGTTTTTCTGTTTGGAACTTT
CTGTTCTCTAAACGAACTTT	TTTTTCTTGTTTTATTGCCACTAGTCTCTAGTC
CTGTTCTCTAAACGAACTTT	TTGTTTTATTGCCACTAGTCTCTAGTCAGTGT
CCAACTTTCGATCTCTTGTA	CTTCTGGTCTAAACGAACTAAATATTATATTA
ACTTTCGATCTCTTGTAGAT	AAATTTACAAAACACCACCAATTAAAGATTTT
TTTCGATCTCTTGTAGATCT	TTCCTCACAGAAAACTTGTTACTTTATATTGA
ACAAACCAACCAACTTTCGA	TCTCGTGTTAAAAATCTGAATTCTTCTAGAGT
CGATCTCTTGTAGATCTGTT	TTAGGCGACGAGCTTGGCACTGATCCTT
CAAACCAACCAACTTTCGAT	CTCTTGTA
CGATCTCTTGTAGATCTGTT	TTCTAAAGAAACTTTGTATTGCATAGACGGT
CGATCTCTTGTAGATCTGTT	-
CGATCTCTTGTAGATCTGTT	CTAAAAAGTCTACTAATTTGGTTAAAAACAA
CGATCTCTTGTAGATCTGTT	TCATTCAAGGAGGAGTTAGATAAATATTTA
CGATCTCTTGTAGATCTGTT	CTTTTGCTCGTTGCTGCTGGCCTTGAAGCCCC
CAAACCAACCAACTTTCGAT	CTCTCGTGTTAAAAATCTGAATTCTTCTAGAG
GATCTCTTGTAGATCTGTTC	TCCAAAAACCCATTACTTT
ATCTCTTGTAGATCTGTTCT	TTTTGCAAACTACACAATTGGAATTGTGTTAA
ATCTCTTGTAGATCTGTTCT	AGAAGCTGAACTTGCAAAGA
ATCTCTTGTAGATCTGTTCT	CACCACAAACCTCTATCACCTCAGCTGTTTTG
ATCTCTTGTAGATCTGTTCT	CACTGTAGCTTATTTTAAT
ATCTCTTGTAGATCTGTTCT	TCAACCGCTACTTTAGACTGACTCTTGGTGTT
TCTCTTGTAGATCTGTTCTC	CAAACTTCTAACTTTAGAGTCCAACCAACAG
CTCTTGTAGATCTGTTCTCT	AAACTTACTGACA
CTCTTGTAGATCTGTTCTCT	AAACTTGTTACTTTATATTGACATTA

CTCTTGTAGATCTGTTCTCT AAA

first_orf_aa
MMPTIFFAGILIVTTIVYLTIV*
MTL*
MKLLLQCHLAM*
MFSTKKTVTQQP*
MFLCLKQNVGLRLTLLKDLMNFALNIQC*
MC*
MADSNGTITVEELKKLLEQWNLVIGFLFLTWICLLQFAYANRNRFLYIIKLIFLWLLWPVTLACFVLAAVYRINWITGGIA
MLLLKSVNFNFVMIHFWVFITTKTTKVGWKVSSEFILVRIIALLNMSLSLFLWTLKENRVISKILGNLCLRILMVILKYILST
MVSNPLMVLVTNHTE*
MLTAQKSLLLFMQINLLLLGVFILQVLMFFKHVQAV*
MKTF*
MADSNGTITVEELKKLLEQWNLVIGFLFLTWICLLQFAYANRNRFLYIIKLIFLWLLWPVTLACFVLAAVYRINWITGGIA
MKSEKQVEQKIAEIPKEEVKPFITESKPSVEQRKQDDKKIKACVEEVTTTLEETKFLTENLLLYIDINGNLHPDSATLVSDID
MVFPLVLLKKLRCAPFC*
MF*
MTI*
MGTWL*
MSDNGPQNQRNAPRITFGGPSDSTGSNQNGERSGARSKQRRPQGLPNNTASWFTALTQHGKEDLKFPRGQGVPIN
MLGTGRESATVLLIILSYIIPHHFPLLSVMECLLLN*
MSSI*
MKGLIKYLMRSALPIQLNSVQK*
MSDVKCTSVVLLSVLQQLRVESSSKLWAQCVQLHNDILLAKDTTEAFEKMVSLLSVLLSMQGAVDINKLCEEMLDNRA
MWVIFNLGLFY*
MWVIFNLGLFY*
MLGTGRESATVLLIILSYIIPHHFPLLSVMECLLLN*
MVLKVLIVTFLYNHMVSNPLMVLVTNHTE*
MLIIFWFSLELQDHNETCHA*
MPLGYVTHGLNLEEAARYMRSLKVPATVSVSSPDAVTAYNGYLTSSSKTPEEHFIETISLAGSYKDWSYSGQSTQLGIEF
MMSAPPAQYELKHGTFTCASEYTGNYQCGHYKHITSKETLYCIDGALLTKSSEYKGPITDVFYKENSYTTTIKPVTYKLDG
MVHLLVLVSTLVITSVVTINI*
MRLVERFNTWLLKLHY*
MSMQIHL*
MLQL*
MADSNGTITVEELKKLLEQWNLVIGFLFLTWICLLQFAYANRNRFLYIIKLIFLWLLWPVTLACFVLAAVYRINWITGGIA
MLIIFWFSLELQDHNETCHA*
MHPALRLVDPQIQLAVTRMENAVGRDQNNVGPKVYPIILRLGSPLSLNMARKTLNSLEDKAFQLTPIAVQMTKLATTE
MAIFIQILPLLLVTLTSLS*
MFLCLKQNVGLRLTLLKDLMNFALNIQC*
MSDVKCTSVVLLSVLQQLRVESSSKLWAQCVQLHNDILLAKDTTEAFEKMVSLLSVLLSMQGAVDINKLCEEMLDNRA
MWVIFNLGLFY*
MVSNPLMVLVTNHTE*
MAQQVLFLNMTTRLVVILKNGNLE*

MRSKKFLELFLGICEKCLHMQKKHAN*
MT*
MVLGSLAATVRLQAGNATEVPANSTVLSFCAFAVDAAKAYKDYLASGGQPITNCVKMLCTHTGTGQAITVTPEANM
MIK*
MLQKKMTLKRVFSLTFVGLYNKS*
MAQQVLFLNMTTRLVVILKNGNLE*
MLLGSMLYMSLGPMVLRGLITLSYHLMMVFILLPLRSLT*
MLLGSMLYMSLGPMVLRGLITLSYHLMMVFILLPLRSLT*
MLLGSMLYMSLGPMVLRGLITLSYHLMMVFILLPLRSLT*
MLLGSMLYMSLGPMVLRGLITLSYHLMMVFILLPLRSLT*
MLLGSMLYMSLGPMVLRGLITLSYHLMMVFILLPLRSLT*
MNLSSISKNLESMSSI*
MADSNGTITVEELKKLLEQWNLVIGFLFLTWICLLQFAYANRNRFLYIIKLIFLWLLWPVTLACFVLAAVYRINWITGGIA
MADSNGTITVEELKKLLEQWNLVIGFLFLTWICLLQFAYANRNRFLYIIKLIFLWLLWPVTLACFVLAAVYRINWITGGIA
MADSNGTITVEELKKLLEQWNLVIGFLFLTWICLLQFAYANRNRFLYIIKLIFLWLLWPVTLACFVLAAVYRINWITGGIA
MADSNGTITVEELKKLLEQWNLVIGFLFLTWICLLQFAYANRNRFLYIIKLIFLWLLWPVTLACFVLAAVYRINWITGGIA
MADSNGTITVEELKKLLEQWNLVIGFLFLTWICLLQFAYANRNRFLYIIKLIFLWLLWPVTLACFVLAAVYRINWITGGIA
MSDNGPQNQRNAPRITFGGPSDSTGSNQNGERSGARSKQRRPQGLPNNTASWFTALTQHGKEDLKFPRGQGVPIN
MRIFTIGTVTLKQGEIKDATPSDFVRATATIPIQASLPFGWLIVGVALLAVFQSASKIITLKKRWQLALSKGVHFVCNLLLL
MADSNGTITVEELKKLLEQWNLVIGFLFLTWICLLQFAYANRNRFLYIIKLIFLWLLWPVTLACFVLAAVYRINWITGGIA
MLLGSMLYMSLGPMVLRGLITLSYHLMMVFILLPLRSLT*
MLLGSMLYMSLGPMVLRGLITLSYHLMMVFILLPLRSLT*
MADSNGTITVEELKKLLEQWNLVIGFLFLTWICLLQFAYANRNRFLYIIKLIFLWLLWPVTLACFVLAAVYRINWITGGIA
MLASSNNMVIALVILLLETSFVHKSLTALLFCHLCSQMK*
MAIFIQILPLLLVTLTSLS*
MADSNGTITVEELKKLLEQWNLVIGFLFLTWICLLQFAYANRNRFLYIIKLIFLWLLWPVTLACFVLAAVYRINWITGGIA
MKIFKKTGTLNIAVVLPVNSCVSLTEGHTLAMSITTSVALMATLLSALKTF*
M*
MFSTKKTVTQQP*
MLTMQLIKPRINQIPGVYVVFGAQNQLKHQIRLMY*
MCQFQLQWFNRHRCSY*
MLI*
ML*
MADSNGTITVEELKKLLEQWNLVIGFLFLTWICLLQFAYANRNRFLYIIKLIFLWLLWPVTLACFVLAAVYRINWITGGIA
MMPTIFFAGILIVTTIVYLTIV*
MKLRETCHYSLKDQ*
MCP*
MAFPSGKVEGCMVQVTCGTTTLNGLWLDDVVYCPRHVICTSEDMLNPNYEDLLIRKSNHNFLVQAGNVQLRVIGHS
MVYMPASWVMRIMTWLDMVDTSLSGFKLKDCVMYASAVVLLILMTARTVYDDGARRVWTLMNVLTLVYKVYYG
MIT*
MLGTGRESATVLLIILSYIIPHHFPLLSVMECLLLN*
MYTLKMQTLWKKLKR*
MAIFIQILPLLLVTLTSLS*

MTLKRVFSLTFVGLYNKS*

IAMACLVGLMWLSYFIASFRLFARTRSMWSFNPETNILLNVPLHGTILTRPLLESELVIGAVILRGHLRIAGHHL
RLLI*

IAMACLVGLMWLSYFIASFRLFARTRSMWSFNPETNILLNVPLHGTILTRPLLESELVIGAVILRGHLRIAGHHL
ITFLKKDAPYIVGDVVQEGVLTAVVIPTKKAGGTTEMLAKALRKVPTDNYITTYPGQGLNGYTVEEAKTVLKK

ITNSSPDDQIGYYRRATRRIRGGDGKMKDLSPRWYFYYLGTGPEAGLPYGANKDGIIWVATEGALNTPKDHI

TLQAIASEFSSLPSYAAFATAQEAYEQAVANGDSEVVLKKLKKSLNVAKSEFDRDAAMQRKLEKMADQAMT

LKRGDKSVYYTSNPTTFHLDGEVITFDNLKTLLSLREVRTIKVFTTVDNINLHTQVVDMSMTYGQQFGPTYLD
VVCTEIDPKLDNYYKKDNSYFTEQPIDLVPNQPYPNASFDNFKFVCDNIKFADDLNQLTGYKKPASRELKVTF

IAMACLVGLMWLSYFIASFRLFARTRSMWSFNPETNILLNVPLHGTILTRPLLESELVIGAVILRGHLRIAGHHL

ELPDEFVVVTVK*

TLQAIASEFSSLPSYAAFATAQEAYEQAVANGDSEVVLKKLKKSLNVAKSEFDRDAAMQRKLEKMADQAMT

QESFGGASCCLYCRCHIDHPNPKGFCDLKGKYVQIPTTCANDPVGFTLKNTVCTVCGMWKGYGCSCDQLR

IAMACLVGLMWLSYFIASFRLFARTRSMWSFNPETNILLNVPLHGTILTRPLLESELVIGAVILRGHLRIAGHHL
IAMACLVGLMWLSYFIASFRLFARTRSMWSFNPETNILLNVPLHGTILTRPLLESELVIGAVILRGHLRIAGHHL
IAMACLVGLMWLSYFIASFRLFARTRSMWSFNPETNILLNVPLHGTILTRPLLESELVIGAVILRGHLRIAGHHL
IAMACLVGLMWLSYFIASFRLFARTRSMWSFNPETNILLNVPLHGTILTRPLLESELVIGAVILRGHLRIAGHHL
IAMACLVGLMWLSYFIASFRLFARTRSMWSFNPETNILLNVPLHGTILTRPLLESELVIGAVILRGHLRIAGHHL
TNSSPDDQIGYYRRATRRIRGGDGKMKDLSPRWYFYYLGTGPEAGLPYGANKDGIIWVATEGALNTPKDHI
FVTVYSHLLLVAAGLEAPFLYLYALVYFLQSINFVRIIMRLWLCWKCRSKNPLLYDANYFLCWHTNCYDYCIPY
IAMACLVGLMWLSYFIASFRLFARTRSMWSFNPETNILLNVPLHGTILTRPLLESELVIGAVILRGHLRIAGHHL

IAMACLVGLMWLSYFIASFRLFARTRSMWSFNPETNILLNVPLHGTILTRPLLESELVIGAVILRGHLRIAGHHL

IAMACLVGLMWLSYFIASFRLFARTRSMWSFNPETNILLNVPLHGTILTRPLLESELVIGAVILRGHLRIAGHHL

IAMACLVGLMWLSYFIASFRLFARTRSMWSFNPETNILLNVPLHGTILTRPLLESELVIGAVILRGHLRIAGHHL

MQNCVLKLKVDTANPKTPKYKFVRIQPGQTFSVLACYNGSPSGVYQCAMRPNFTIKGSFLNGSCGSVGFNID
ALDQAISMWALIISVTSNYSGVVTTVMFLARGIVFMCVEYCPIFFITGNTLQCIMLVYCFLGYFCTCYFGLFCL

RCDIKDLPKEITVATSRTLSYYKLGASQRVAGDSGFAAYSRYRIGNYKLNTDHSSSSDNIALLVQ*

RCDIKDLPKEITVATSRTLSYYKLGASQRVAGDSGFAAYSRYRIGNYKLNTDHSSSSDNIALLVQ*
KSAFYILPSIISNEKQEILGTVSWNLREMLAHAEETRKLMPVCVETKAIVSTIQRKYKGIKIQEGVVDYGARFYF

TRNPANNAAIVLQLPQGTTLPKGFYAEGSRGGSQASSRSSSRSRNSSRNSTPGSSRGTSPARMAGNGGDAA

QMYKQARSEDKRAKVTSAMQTMLFTMLRKLDNDALNNIINNARDGCVPLNIIPLTTAAKLMVVIPDYNTYK

ADVTKIKPHNSHEGKTFYVLPNDDTLRVEAFEYYHTTDPSFLGRYMSALNHTKKWKYPQVNGLTSIKWADN
PDLNGDVVAIDYKHYTPSFKKGAKLLHKPIVWHVNNATNKATYKPNTWCIRCLWSTKPVETSNSFDVLKSED

RCDIKDLPKEITVATSRTLSYYKLGASQRVAGDSGFAAYSRYRIGNYKLNTDHSSSSDNIALLVQ*

QMYKQARSEDKRAKVTSAMQTMLFTMLRKLDNDALNNIINNARDGCVPLNIIPLTTAAKLMVVIPDYNTYK

EPMLQSADAQSFLNGFAV*

GRCDIKDLPKEITVATSRTLSYYKLGASQRVAGDSGFAAYSRYRIGNYKLNTDHSSSSDNIALLVQ*
GRCDIKDLPKEITVATSRTLSYYKLGASQRVAGDSGFAAYSRYRIGNYKLNTDHSSSSDNIALLVQ*
GRCDIKDLPKEITVATSRTLSYYKLGASQRVAGDSGFAAYSRYRIGNYKLNTDHSSSSDNIALLVQ*
GRCDIKDLPKEITVATSRTLSYYKLGASQRVAGDSGFAAYSRYRIGNYKLNTDHSSSSDNIALLVQ*
GRCDIKDLPKEITVATSRTLSYYKLGASQRVAGDSGFAAYSRYRIGNYKLNTDHSSSSDNIALLVQ*
GTRNPANNAAIVLQLPQGTTLPKGFYAEGSRGGSQASSRSSSRSRNSSRNSTPGSSRGTSPARMAGNGGDAA
NSVTSSIVITSGDGTTSPISEHDYQIGGYTEKWESGVKDCVVLHSYFTSDYYQLYSTQLSTDTGVEHVTFFIYNKI
GRCDIKDLPKEITVATSRTLSYYKLGASQRVAGDSGFAAYSRYRIGNYKLNTDHSSSSDNIALLVQ*

GRCDIKDLPKEITVATSRTLSYYKLGASQRVAGDSGFAAYSRYRIGNYKLNTDHSSSSDNIALLVQ*

GRCDIKDLPKEITVATSRTLSYYKLGASQRVAGDSGFAAYSRYRIGNYKLNTDHSSSSDNIALLVQ*

GRCDIKDLPKEITVATSRTLSYYKLGASQRVAGDSGFAAYSRYRIGNYKLNTDHSSSSDNIALLVQ*

YDCVSFCYMHHMELPTGVHAGTDLEGNFYGPFVDRQTAQAAGTDTTITVNVLAWLYAAVINGDRWFLNR
NRYFRLTLGVYDYLVSTQEFRYMNSQGLLPPKNSIDAFKLNIKLLGVGGKPCIKVATVQSKMSDVKCTSVVLLS

YTSKTTVASLINTLNDLNETLVTMPLGYVTHGLNLEEAARYMRSLKVPATVSVSSPDAVTAYNGYLTSSSKTPE

LALLLLDRLNQLESKMSGKGQQQQGQTVTKKSAAEASKKPRQKRTATKAYNVTQAFGRRGPEQTQGNFGD

NTCDGTTFTYASALWEIQQVVDADSKIVQLSEISMDNSPNLAWPLIVTALRANSAVKLQNNELSPVALRQMS

INCYLATALLTLQQIELKFNPPALQDAYYRARAGEAANFCALILAYCNKTVGELGDVRETMSYLFQHANLDSCK
AQGMDNLACEDLKPVSEEVVENPTIQKDVLECNVKTTEVVGDIILKPANNSLKITEEVGHTDLMAAYVDNSS

NTCDGTTFTYASALWEIQQVVDADSKIVQLSEISMDNSPNLAWPLIVTALRANSAVKLQNNELSPVALRQMS

ALALLLLDRLNQLESKMSGKGQQQQGQTVTKKSAAEASKKPRQKRTATKAYNVTQAFGRRGPEQTQGNFGD
VDEPEEHVQIHTIDGSSGVVNPVMEPIYDEPTTTTSVPL*

FTTTLNDFNLVAMKYNYEPLTQDHVDILGPLSAQTGIAVLDMCASLKELLQNGMNGRTILGSALLEDEFTPFD
SVLQQLRVESSSKLWAQCVQLHNDILLAKDTTEAFEKMVSLLSVLLSMQGAVDINKLCEEMLDNRATLQAIAS

EHFIETISLAGSYKDWSYSGQSTQLGIEFLKRGDKSVYYTSNPTTFHLDGEVITFDNLKTLLSLREVRTIKVFTTVD

QELIRQGTDYKHWPQIAQFAPSASAFFGMSRIGMEVTPSGTWLTYTGAIKLDDKDPNFKDQVILLNKHIDAY

CAAGTTQTACTDDNALAYYNTTKGGRFVLALLSDLQDLKWARFPKSDGTGTIYTELEPPCRFVTDTPKGPKV

RVLNVVCKTCGQQQTTLKGVEAVMYMGTLSYEQFKKGVQIPCTCGKQATKYLVQQESPFVMMSAPPAQY
LTIKKPNELSRVLGLKTLATHGLAAVNSVPWDTIANYAKPFLNKVVSTTTNIVTRCLNRVCTNYMPYFFTLLLQ

CAAGTTQTACTDDNALAYYNTTKGGRFVLALLSDLQDLKWARFPKSDGTGTIYTELEPPCRFVTDTPKGPKV

QELIRQGTDYKHWPQIAQFAPSASAFFGMSRIGMEVTPSGTWLTYTGAIKLDDKDPNFKDQVILLNKHIDAY

VVRQCSGVTFQSAVKRTIKGTHHWLLLTILTSLLVLVQSTQWSLFFFLYENAFLPFAMGIIAMSAFAMMFVKH
EFSSLPSYAAFATAQEAYEQAVANGDSEVVLKKLKKSLNVAKSEFDRDAAMQRKLEKMADQAMTQMYKQA

NINLHTQVVDMSMTYGQQFGPTYLDGADVTKIKPHNSHEGKTFYVLPNDDTLRVEAFEYYHTTDPSFLGRY

KTFPPTEPKKDKKKKADETQALPQRQKKQQTVTLLPAADLDDFSKQLQQSMSSADSTQA*

YLYFIKGLNNLNRGMVLGSLAATVRLQAGNATEVPANSTVLSFCAFAVDAAKAYKDYLASGGQPITNCVKM

LKHGTFTCASEYTGNYQCGHYKHITSKETLYCIDGALLTKSSEYKGPITDVFYKENSYTTTIKPVTYKLDGVVCT
CTFTRSTNSRIKASMPTTIAKNTVKSVGKFCLEASFNYLKSPNFSKLINIIIWFLLLSVCLGSLIYSTAALGVLMSN

YLYFIKGLNNLNRGMVLGSLAATVRLQAGNATEVPANSTVLSFCAFAVDAAKAYKDYLASGGQPITNCVKM

KTFPPTEPKKDKKKKADETQALPQRQKKQQTVTLLPAADLDDFSKQLQQSMSSADSTQA*

KHAFLCLFLLPSLATVAYFNMVYMPASWVMRIMTWLDMVDTSLSGFKLKDCVMYASAVVLLILMTARTVYD
RSEDKRAKVTSAMQTMLFTMLRKLDNDALNNIINNARDGCVPLNIIPLTTAAKLMVVIPDYNTYKNTCDGTT

MSALNHTKKWKYPQVNGLTSIKWADNNCYLATALLTLQQIELKFNPPALQDAYYRARAGEAANFCALILAYCI

LCTHTGTGQAITVTPEANMDQESFGGASCCLYCRCHIDHPNPKGFCDLKGKYVQIPTTCANDPVGFTLKNTV

EIDPKLDNYYKKDNSYFTEQPIDLVPNQPYPNASFDNFKFVCDNIKFADDLNQLTGYKKPASRELKVTFFPDLN
LGMPSYCTGYREGYLNSTNVTIATYCTGSIPCSVCLSGLDSLDTYPSLETIQITISSFKWDLTAFGLVAEWFLAYIL

LCTHTGTGQAITVTPEANMDQESFGGASCCLYCRCHIDHPNPKGFCDLKGKYVQIPTTCANDPVGFTLKNTV

[illegible]DGARRVWTLMNVLTLVYKVYYGNALDQAISMWALIISVTSNYSGVVTTVMFLARGIVFMCVEYCPIFFITG[illegible]
[illegible]FTYASALWEIQQVVDADSKIVQLSEISMDNSPNLAWPLIVTALRANSAVKLQNNELSPVALRQMSCAAGTT[illegible]

NKTVGELGDVRETMSYLFQHANLDSCKRVLNVVCKTCGQQQTTLKGVEAVMYMGTLSYEQFKKGVQIPCTC

CTVCGMWKGYGCSCDQLREPMLQSADAQSFLNGFAV*

GDVVAIDYKHYTPSFKKGAKLLHKPIVWHVNNATNKATYKPNTWCIRCLWSTKPVETSNSFDVLKSEDAQGM
LFTRFFYVLGLAAIMQLFFSYFAVHFISNSWLMWLIINLVQMAPISAMVRMYIFFASFYYVWKSYVHVVDGCN

CTVCGMWKGYGCSCDQLREPMLQSADAQSFLNGFAV*

NTLQCIMLVYCFLGYFCTCYFGLFCLLNRYFRLTLGVYDYLVSTQEFRYMNSQGLLPPKNSIDAFKLNIKLLGVGG
QTACTDDNALAYYNTTKGGRFVLALLSDLQDLKWARFPKSDGTGTIYTELEPPCRFVTDTPKGPKVKYLYFIKGL

GKQATKYLVQQESPFVMMSAPPAQYELKHGTFTCASEYTGNYQCGHYKHITSKETLYCIDGALLTKSSEYKG

DNLACEDLKPVSEEVVENPTIQKDVLECNVKTTEVVGDIILKPANNSLKITEEVGHTDLMAAYVDNSSLTIKKP
SSTCMMCYKRNRATRVECTTIVNGVRRSFYVYANGGKGFCKLHNWNCVNCDTFCAGSTFISDEVARDLSLQ

[illegible]KPCIKVATVQSKMSDVKCTSVVLLSVLQQLRVESSSKLWAQCVQLHNDILLAKDTTEAFEKMVSLLSVLLSM[illegible]
[illegible]LNNLNRGMVLGSLAATVRLQAGNATEVPANSTVLSFCAFAVDAAKAYKDYLASGGQPITNCVKMLCTHTGT[illegible]

PITDVFYKENSYTTTIKPVTYKLDGVVCTEIDPKLDNYYKKDNSYFTEQPIDLVPNQPYPNASFDNFKFVCDNIKF

PNELSRVLGLKTLATHGLAAVNSVPWDTIANYAKPFLNKVVSTTTNIVTRCLNRVCTNYMPYFFTLLLQLCTFTR
QFKRPINPTDQSSYIVDSVTVKNGSIHLYFDKAGQKTYERHSLSHFVNLDNLRANNTKGSLPINVIVFDGKSKCE

QGAVDINKLCEEMLDNRATLQAIASEFSSLPSYAAFATAQEAYEQAVANGDSEVVLKKLKKSLNVAKSEFDRDA
GQAITVTPEANMDQESFGGASCCLYCRCHIDHPNPKGFCDLKGKYVQIPTTCANDPVGFTLKNTVCTVCGMV

ADDLNQLTGYKKPASRELKVTFFPDLNGDVVAIDYKHYTPSFKKGAKLLHKPIVWHVNNATNKATYKPNTW

STNSRIKASMPTTIAKNTVKSVGKFCLEASFNYLKSPNFSKLINIIIWFLLLSVCLGSLIYSTAALGVLMSNLGMP
SSAKSASVYYSQLMCQPILLLDQALVSDVGDSAEVAVKMFDAYVNTFSSTFNVPMEKLKTLVATAEAELAKN

AAMQRKLEKMADQAMTQMYKQARSEDKRAKVTSAMQTMLFTMLRKLDNDALNNIINNARDGCVPLNIIP[illegible]
[illegible]WKGYGCSCDQLREPMLQSADAQSFLNGFAV*

CIRCLWSTKPVETSNSFDVLKSEDAQGMDNLACEDLKPVSEEVVENPTIQKDVLECNVKTTEVVGDIILKPANN

SYCTGYREGYLNSTNVTIATYCTGSIPCSVCLSGLDSLDTYPSLETIQITISSFKWDLTAFGLVAEWFLAYILFTRFF
IVSLDNVLSTFISAARQGFVDSDVETKDVVECLKLSHQSDIEVTGDSCNNYMLTYNKVENMTPRDLGACIDCSA

LTTAAKLMVVIPDYNTYKNTCDGTTFTYASALWEIQQVVDADSKIVQLSEISMDNSPNLAWPLIVTALRANSA

NSLKITEEVGHTDLMAAYVDNSSLTIKKPNELSRVLGLKTLATHGLAAVNSVPWDTIANYAKPFLNKVVSTTTN

YVLGLAAIMQLFFSYFAVHFISNSWLMWLIINLVQMAPISAMVRMYIFFASFYYVWKSYVHVVDGCNSSTCM
ARHINAQVAKSHNIALIWNVKDFMSLSEQLRKQIRSAAKKNNLPFKLTCATTRQVVNVVTTKIALKGGKIVNN

[illegible]VKLQNNELSPVALRQMSCAAGTTQTACTDDNALAYYNTTKGGRFVLALLSDLQDLKWARFPKSDGTGTIYT[illegible]

IVTRCLNRVCTNYMPYFFTLLLQLCTFTRSTNSRIKASMPTTIAKNTVKSVGKFCLEASFN

[illegible]MCYKRNRATRVECTTIVNGVRRSFYVYANGGKGFCKLHNWNCVNCDTFCAGSTFISDEVAR
WLKQLIKVTLVFLFVAAIFYLITPVHVMSKHTDFSSEIIGYKAIDGGVTRDIASTDTCFAN

ELEPPCRFVTDTPKGPKVKYLYFIKGLNNLNRGMVLGSLAATVRLQAGNATEVPANSTVLSFCAFAVDAAKAY

KDYLASGGQPIT

Supplemental tables

Click here to access/download;Table;Supplemental_Material_2.xlsx

subgenome	ref_leader_e	peak_leader_	ref_TRS_start	peak_TRS_sta	peak_count
S	65	65	21552	21552	14498(14323,4484,4373,538
ORF3a	69	69	25385	25385	6021(5795,0,0,1920)
E	69	69	26237	26237	1826(1779,41,41,642)
M	65	65	26469	26469	3728(3690,1602,1588,2042)
ORF6	69	69	27041	27041	3590(3540,2068,2038,3234)
ORF7a	69	69	27388	27388	972(921,54,39,290)
ORF7b	65	65	27644	27644	21(19,0,0,14)
ORF8	65	65	27884	27884	1083(1065,0,0,238)
N	65	64	28256	28255	5167(5084,3468,3434,2220)
ORF10	65	68	29530	29538	2(2,0,0,2)

The numbers in the bracket are (reads with left primers, reads with right primers, reads with both

Normalized count=(Read count/Total number of read mapped on reference genome)*1000000.

Total number of read mapped on reference genome is 46368772, excluding the mapped reads un

“ref_leader_end” and “peak_leader_end” point to the reference position of the end of leader an

peak_normalized_count	cluster_count	cluster_normalized_count
312.67(308.89,96.70,94.31,	18395(18152,6698,656	396.71(391.47,144.45,141.56,119.56)
129.85(124.98,0.00,0.00,41	6334(6095,0,0,1968)	136.60(131.45,0.00,0.00,42.44)
39.38(38.37,0.88,0.88,13.8	1826(1779,41,41,642)	39.38(38.37,0.88,0.88,13.85)
80.40(79.58,34.55,34.25,44	4947(4841,1686,1665,2	106.69(104.40,36.36,35.91,50.51)
77.42(76.34,44.60,43.95,69	3643(3593,2108,2078,3	78.57(77.49,45.46,44.81,70.35)
20.96(19.86,1.16,0.84,6.25)	996(944,55,40,290)	21.48(20.36,1.19,0.86,6.25)
0.45(0.41,0.00,0.00,0.30)	30(26,1,1,18)	0.65(0.56,0.02,0.02,0.39)
23.36(22.97,0.00,0.00,5.13)	1123(1104,0,0,238)	24.22(23.81,0.00,0.00,5.13)
111.43(109.64,74.79,74.06,	8243(8075,5794,5737,2	177.77(174.15,124.95,123.73,53.70)
0.04(0.04,0.00,0.00,0.04)	2(2,0,0,2)	0.04(0.04,0.00,0.00,0.04)

primers, same junction on paired reads with at least a primer).

paired, not primary alignment and supplementary alignment.

d the position of the end of leader identified in the most common reads (peak count) on the

reference genome, and “ref_TRS_start” and “peak_TRS_start” refer to the reference position of

the start of TRS and the position of the start of TRS identified in the most common reads (peak

count) on the reference genome.

subgenome	peak_leader_	peak_TRS_sta	ACGAAC	ATG_postion	20_leader_se	TRS_seq
S	65	21552	yes	21563	ATCTCTTGTA(	CTAAACGAAC
ORF3a	69	25385	yes	25393	CTTGTAGATC	ACGAACTT
E	69	26237	yes	26245	CTTGTAGATC	ACGAACTT
M	65	26469	yes	26523	ATCTCTTGTA(	CTAAACGAAC
ORF6	69	27041	yes	27202	CTTGTAGATC	ACGAACGCTT
ORF7a	69	27388	yes	27394	CTTGTAGATC	ACGAAC
ORF7b	65	27644	no	27756	ATCTCTTGTA(	CTAAACTGTT
ORF8	65	27884	yes	27894	ATCTCTTGTA(	CTAAACGAAC
N	64	28255	yes	28274	GATCTCTTGT.	TCTAAACGAA
ORF10	68	29538	no	29538	TCTTGTAGAT	-

“peak_leader” and “peak_TRS_start” point to the leader-TRS junctions in sheet "known", “ACGA

first_orf_aa

MFVFLVLLPLVSSQCVNLTTRTQLPPAYTNSFTRGVYYPDKVFRSSVLHSTQDLFLPFFSNVTWFHAIHVSGTN

MDLFMRIFTIGTVTLKQGEIKDATPSDFVRATATIPIQASLPFGWLIVGVALLAVFQSASKIITLKKRWQLALSKG

MYSFVSEETGTLIVNSVLLFLAFVVFLLVTLAILTALRLCAYCCNIVNVSLVKPSFYVYSRVKNLNSSRVPDLLV*

MADSNGTITVEELKKLLEQWNLVIGFLFLTWICLLQFAYANRNRFLYIIKLIFLWLLWPVTLACFVLAAVYRINW

MFHLVDFQVTIAEILLIIMRTFKVSIWNLDYIINLIIKNLSKSLTENKYSQLDEEQPMEID*

MKIILFLALITLATCELYHYQECVRGTTVLLKEPCSSGTYEGNSPFHPLADNKFALTCFSTQFAFACPDGVKHVYC

MIELSLIDFYLCFLAFLLFLVLIMLIIFWFSLELQDHNETCHA*

MKFLVFLGIITTVAAFHQECSLQSCTQHQPYVVDDPCPIHFYSKWYIRVGARKSAPLIELCVDEAGSKSPIQYIDI

MSDNGPQNQRNAPRITFGGPSDSTGSNQNGERSGARSKQRRPQGLPNNTASWFTALTQHGKEDLKFPRGC

MQTTQGRWAI*

AC” indicates if there is a ACGAAC sequence in the “TRS_seq” (TRS sequences), “20_leader_seq”

GTKRFDNPVLPFNDGVYFASTEKSNIIRGWIFGTTLDSKTQSLLIVNNATNVVIKVCEFQFCNDPFLGVYYHKNN
VHFVCNLLLLFVTVYSHLLLVAAGLEAPFLYLYALVYFLQSINFVRIIMRLWLCWKCRSKNPLLYDANYFLCWHT

ITGGIAIAMACLVGLMWLSYFIASFRLFARTRSMWSFNPETNILLNVPLHGTILTRPLLESELVIGAVILRGHLRIA

LRARSVSPKLFIRQEEVQELYSPIFLIVAAIVFITLCFTLKRKTE*

GNYTVSCLPFTINCQEPKLGSLVVRCSFYEDFLEYHDVRVVLDFI*
GVPINTNSSPDDQIGYYRRATRRIRGGDGKMKDLSPRWYFYYLGTGPEAGLPYGANKDGIIWVATEGALNTP

refers to the 20 nucleotides before the end of leader, and “AUG_postion” and “first_orf_aa” ref

IKSWMESEFRVYSSANNCTFEYVSQPFLMDLEGKQGNFKNLREFVFKNIDGYFKIYSKHTPINLVRDLPQGFSAI
NCYDYCIPYNSVTSSIVITSGDGTTSPISEHDYQIGGYTEKWESGVKDCVVLHSYFTSDYYQLYSTQLSTDTGVEH

GHHLGRCDIKDLPKEITVATSRTLSYYKLGASQRVAGDSGFAAYSRYRIGNYKLNTDHSSSSDNIALLVQ*

KDHIGTRNPANNAAIVLQLPQGTTLPKGFYAEGSRGGSQASSRSSSRSRNSSRNSTPGSSRGTSPARMAGNG[illegible]

er to the first AUG position and translated orf of the sgmRNA.

[illegible]EPLVDLPIGINITRFQTLLALHRSYLTPGDSSSGWTAGAAAYYVGYLQPRTFLLKYNENGTITDAVDCALDPLSE[illegible]
VTFFIYNKIVDEPEEHVQIHTIDGSSGVVNPVMEPIYDEPTTTTSVPL*

[illegible]GDAALALLLLDRLNQLESKMSGKGQQQQGQTVTKKSAAEASKKPRQKRTATKAYNVTQAFGRRGPEQTQGN

TKCTLKSFTVEKGIYQTSNFRVQPTESIVRFPNITNLCPFGEVFNATRFASVYAWNRKRISNCVADYSVLYNSASF

FGDQELIRQGTDYKHWPQIAQFAPSASAFFGMSRIGMEVTPSGTWLTYTGAIKLDDKDPNFKDQVILLNKHID

STFKCYGVSPTKLNDLCFTNVYADSFVIRGDEVRQIAPGQTGKIADYNYKLPDDFTGCVIAWNSNNLDSKVGG[illegible]

[illegible]AYKTFPPTEPKKDKKKKADETQALPQRQKKQQTVTLLPAADLDDFSKQLQQSMSSADSTQA*

NYNYLYRLFRKSNLKPFERDISTEIYQAGSTPCNGVEGFNCYFPLQSYGFQPTNGVGYQPYRVVVLSFELLHAPA

TVCGPKKSTNLVKNKCVNFNFNGLTGTGVLTESNKKFLPFQQFGRDIADTTDAVRDPQTLEILDITPCSFGGVS[illegible]

VITPGTNTSNQVAVLYQDVNCTEVPVAIHADQLTPTWRVYSTGSNVFQTRAGCLIGAEHVNNSYECDIPIGAG

ICASYQTQTNSPRRARSVASQSIIAYTMSLGAENSVAYSNNSIAIPTNFTISVTTEILPVSMTKTSVDCTMYICGD[illegible]

STECSNLLLQYGSFCTQLNRALTGIAVEQDKNTQEVFAQVKQIYKTPPIKDFGGFNFSQILPDPSKPSKRSFIEDLL

.FNKVTLADAGFIKQYGDCLGDIAARDLICAQKFNGLTVLPPLLTDEMIAQYTSALLAGTITSGWTFGAGAALQI[illegible]

PFAMQMAYRFNGIGVTQNVLYENQKLIANQFNSAIGKIQDSLSSTASALGKLQDVVNQNAQALNTLVKQLSSN

NFGAISSVLNDILSRLDKVEAEVQIDRLITGRLQSLQTYVTQQLIRAAEIRASANL

subgenome	leader_end	TRS_start	nb_count	normalized_count
1	47	26421	45(45,6,6,6)	0.97(0.97,0.13,0.13,0.13)
2	64	6935	15(12,7,6,6)	0.32(0.26,0.15,0.13,0.13)
3	65	3913	17(17,0,0,4)	0.37(0.37,0.00,0.00,0.09)
4	65	4577	15(9,7,5,6)	0.32(0.19,0.15,0.11,0.13)
5	65	5807	12(12,4,4,4)	0.26(0.26,0.09,0.09,0.09)
6	65	6495	34(2,4,2,2)	0.73(0.04,0.09,0.04,0.04)
7	65	8113	52(36,23,8,44)	1.12(0.78,0.50,0.17,0.95)
8	65	10020	22(20,14,12,16)	0.47(0.43,0.30,0.26,0.35)
9	65	10636	39(36,4,4,6)	0.84(0.78,0.09,0.09,0.13)
10	65	11664	14(14,0,0,4)	0.30(0.30,0.00,0.00,0.09)
11	48	26443	100(100,12,12,12)	2.16(2.16,0.26,0.26,0.26)
12	65	13730	110(14,94,0,6)	2.37(0.30,2.03,0.00,0.13)
13	66	736	17(0,0,0,0)	0.37(0.00,0.00,0.00,0.00)
14	66	3360	103(10,45,5,42)	2.22(0.22,0.97,0.11,0.91)
15	66	5635	35(34,0,0,12)	0.75(0.73,0.00,0.00,0.26)
16	66	5773	14(8,3,3,2)	0.30(0.17,0.06,0.06,0.04)
17	66	6181	21(2,1,0,0)	0.45(0.04,0.02,0.00,0.00)
18	66	10857	13(11,3,3,8)	0.28(0.24,0.06,0.06,0.17)
19	66	13865	48(1,1,0,0)	1.04(0.02,0.02,0.00,0.00)
20	66	14679	1679(1,1677,0,0)	36.21(0.02,36.17,0.00,0.00)
21	66	22502	32(24,0,0,4)	0.69(0.52,0.00,0.00,0.09)
22	67	2194	35(2,0,0,0)	0.75(0.04,0.00,0.00,0.00)
23	67	2292	72(72,0,0,10)	1.55(1.55,0.00,0.00,0.22)
24	67	3362	49(46,26,26,30)	1.06(0.99,0.56,0.56,0.65)
25	67	3916	26(1,25,0,0)	0.56(0.02,0.54,0.00,0.00)
26	67	4018	151(139,54,53,78)	3.26(3.00,1.16,1.14,1.68)
27	67	5875	35(3,0,0,0)	0.75(0.06,0.00,0.00,0.00)
28	67	7505	14(8,4,4,6)	0.30(0.17,0.09,0.09,0.13)
29	67	8205	13(2,3,2,2)	0.28(0.04,0.06,0.04,0.04)
30	52	21491	11(9,2,0,0)	0.24(0.19,0.04,0.00,0.00)
31	67	13691	25(2,0,0,0)	0.54(0.04,0.00,0.00,0.00)
32	67	13916	28(5,2,0,4)	0.60(0.11,0.04,0.00,0.09)
33	67	14663	12(10,0,0,6)	0.26(0.22,0.00,0.00,0.13)
34	67	22278	39(5,2,2,2)	0.84(0.11,0.04,0.04,0.04)
35	67	22404	39(23,14,0,8)	0.84(0.50,0.30,0.00,0.17)
36	67	22490	26(23,0,0,6)	0.56(0.50,0.00,0.00,0.13)
37	67	22503	156(151,4,4,42)	3.36(3.26,0.09,0.09,0.91)
38	68	738	11(0,0,0,0)	0.24(0.00,0.00,0.00,0.00)
39	68	2689	1607(1572,1445,1413,[illegible]	34.66(33.90,31.16,30.47,29.85)
40	68	4580	555(542,486,482,516)	11.97(11.69,10.48,10.39,11.13)
41	68	5789	174(170,38,36,70)	3.75(3.67,0.82,0.78,1.51)
42	55	21538	15(9,5,4,4)	0.32(0.19,0.11,0.09,0.09)

43	68	8072	19(15,0,0,2)	0.41(0.32,0.00,0.00,0.04)
44	68	14223	29(23,2,2,10)	0.63(0.50,0.04,0.04,0.22)
45	68	14686	15(1,11,0,0)	0.32(0.02,0.24,0.00,0.00)
46	68	15777	763(703,12,12,258)	16.46(15.16,0.26,0.26,5.56)
47	55	26458	12(2,3,2,2)	0.26(0.04,0.06,0.04,0.04)
48	68	18051	12(9,0,0,2)	0.26(0.19,0.00,0.00,0.04)
49	68	21926	39(36,1,0,4)	0.84(0.78,0.02,0.00,0.09)
50	68	23365	13(13,4,4,12)	0.28(0.28,0.09,0.09,0.26)
51	55	26461	26(24,2,2,2)	0.56(0.52,0.04,0.04,0.04)
52	68	25425	17(1,16,0,10)	0.37(0.02,0.35,0.00,0.22)
53	69	705	33(23,0,0,2)	0.71(0.50,0.00,0.00,0.04)
54	69	1897	17(5,1,1,0)	0.37(0.11,0.02,0.02,0.00)
55	69	3816	158(151,0,0,50)	3.41(3.26,0.00,0.00,1.08)
56	69	7186	16(11,6,3,0)	0.35(0.24,0.13,0.06,0.00)
57	69	7503	34(30,15,14,18)	0.73(0.65,0.32,0.30,0.39)
58	69	9713	17(15,0,0,6)	0.37(0.32,0.00,0.00,0.13)
59	69	10640	95(87,32,32,40)	2.05(1.88,0.69,0.69,0.86)
60	69	11356	30(27,6,6,14)	0.65(0.58,0.13,0.13,0.30)
61	69	12615	38(1,4,0,4)	0.82(0.02,0.09,0.00,0.09)
62	69	13619	105(104,46,46,56)	2.26(2.24,0.99,0.99,1.21)
63	69	13679	25(23,0,0,12)	0.54(0.50,0.00,0.00,0.26)
64	69	18554	25(25,10,10,14)	0.54(0.54,0.22,0.22,0.30)
65	69	19112	138(129,105,96,118)	2.98(2.78,2.26,2.07,2.54)
66	69	19571	31(18,2,2,6)	0.67(0.39,0.04,0.04,0.13)
67	69	21305	25(24,6,6,10)	0.54(0.52,0.13,0.13,0.22)
68	69	21358	42(1,0,0,0)	0.91(0.02,0.00,0.00,0.00)
69	69	22559	14(10,0,0,0)	0.30(0.22,0.00,0.00,0.00)
70	70	913	17(14,12,12,12)	0.37(0.30,0.26,0.26,0.26)
71	70	2794	1860(1838,1666,1648,[illegible]	40.11(39.64,35.93,35.54,37.57)
72	70	2851	27(23,19,17,18)	0.58(0.50,0.41,0.37,0.39)
73	70	3862	11(2,0,0,2)	0.24(0.04,0.00,0.00,0.04)
74	70	6472	240(10,6,6,6)	5.18(0.22,0.13,0.13,0.13)
75	70	7077	15(7,4,4,4)	0.32(0.15,0.09,0.09,0.09)
76	70	10820	20(3,17,0,0)	0.43(0.06,0.37,0.00,0.00)
77	70	11779	20(19,12,12,14)	0.43(0.41,0.26,0.26,0.30)
78	70	11812	14(13,8,8,10)	0.30(0.28,0.17,0.17,0.22)
79	70	14372	47(45,0,0,8)	1.01(0.97,0.00,0.00,0.17)
80	70	14670	17(10,5,0,6)	0.37(0.22,0.11,0.00,0.13)
81	70	22277	97(91,14,14,28)	2.09(1.96,0.30,0.30,0.60)
82	70	22502	459(405,4,4,138)	9.90(8.73,0.09,0.09,2.98)
83	70	22522	112(3,0,0,0)	2.42(0.06,0.00,0.00,0.00)
84	70	27761	33(30,6,6,12)	0.71(0.65,0.13,0.13,0.26)
85	71	734	15(1,0,0,0)	0.32(0.02,0.00,0.00,0.00)

86	71	4586	23(20,20,20,20)	0.50(0.43,0.43,0.43,0.43)
87	71	4592	40(36,36,36,36)	0.86(0.78,0.78,0.78,0.78)
88	71	5594	29(25,0,0,4)	0.63(0.54,0.00,0.00,0.09)
89	71	5708	124(14,109,0,6)	2.67(0.30,2.35,0.00,0.13)
90	71	10772	39(31,0,0,14)	0.84(0.67,0.00,0.00,0.30)
91	71	12208	15(1,0,0,0)	0.32(0.02,0.00,0.00,0.00)
92	71	12412	33(2,2,2,2)	0.71(0.04,0.04,0.04,0.04)
93	71	13313	11(1,4,0,4)	0.24(0.02,0.09,0.00,0.09)
94	71	13583	16(14,8,8,12)	0.35(0.30,0.17,0.17,0.26)
95	59	735	12(1,0,0,0)	0.26(0.02,0.00,0.00,0.00)
96	71	18943	36(32,0,0,8)	0.78(0.69,0.00,0.00,0.17)
97	71	22403	36(1,33,0,0)	0.78(0.02,0.71,0.00,0.00)
98	71	22409	27(23,3,0,4)	0.58(0.50,0.06,0.00,0.09)
99	71	22498	26(22,0,0,4)	0.56(0.47,0.00,0.00,0.09)
100	71	23926	249(1,245,0,0)	5.37(0.02,5.28,0.00,0.00)
101	60	2285	20(15,0,0,0)	0.43(0.32,0.00,0.00,0.00)
102	72	2689	44(10,39,8,8)	0.95(0.22,0.84,0.17,0.17)
103	60	2613	12(11,0,0,2)	0.26(0.24,0.00,0.00,0.04)
104	72	9284	18(6,0,0,4)	0.39(0.13,0.00,0.00,0.09)
105	72	9889	12(7,0,0,2)	0.26(0.15,0.00,0.00,0.04)
106	72	15808	18(16,0,0,6)	0.39(0.35,0.00,0.00,0.13)
107	72	22107	11(6,5,0,4)	0.24(0.13,0.11,0.00,0.09)
108	72	22511	17(2,0,0,0)	0.37(0.04,0.00,0.00,0.00)
109	60	4007	26(17,8,8,8)	0.56(0.37,0.17,0.17,0.17)
110	73	3372	13(13,9,9,8)	0.28(0.28,0.19,0.19,0.17)
111	73	3776	38(6,2,2,4)	0.82(0.13,0.04,0.04,0.09)
112	73	4027	28(12,3,2,4)	0.60(0.26,0.06,0.04,0.09)
113	73	8145	22(22,13,13,18)	0.47(0.47,0.28,0.28,0.39)
114	73	8957	22(1,0,0,0)	0.47(0.02,0.00,0.00,0.00)
115	73	14371	11(3,2,0,0)	0.24(0.06,0.04,0.00,0.00)
116	60	13994	11(10,0,0,2)	0.24(0.22,0.00,0.00,0.04)
117	73	22285	11(2,0,0,0)	0.24(0.04,0.00,0.00,0.00)
118	73	23028	35(29,4,4,8)	0.75(0.63,0.09,0.09,0.17)
119	74	165	1809(6,1800,0,0)	39.01(0.13,38.82,0.00,0.00)
120	60	20306	11(9,0,0,0)	0.24(0.19,0.00,0.00,0.00)
121	74	5890	15(8,6,6,6)	0.32(0.17,0.13,0.13,0.13)
122	74	5893	25(8,0,0,0)	0.54(0.17,0.00,0.00,0.00)
123	74	6721	46(0,9,0,6)	0.99(0.00,0.19,0.00,0.13)
124	74	6724	32(0,5,0,4)	0.69(0.00,0.11,0.00,0.09)
125	74	6853	36(1,1,0,0)	0.78(0.02,0.02,0.00,0.00)
126	74	7704	24(19,0,0,8)	0.52(0.41,0.00,0.00,0.17)
127	74	12407	12(7,4,4,4)	0.26(0.15,0.09,0.09,0.09)
128	74	12742	13(5,0,0,2)	0.28(0.11,0.00,0.00,0.04)

129	74	12974	107(101,62,60,84)	2.31(2.18,1.34,1.29,1.81)
130	74	13630	24(2,2,2,2)	0.52(0.04,0.04,0.04,0.04)
131	60	22933	28(3,0,0,2)	0.60(0.06,0.00,0.00,0.04)
132	74	19227	11(10,6,6,10)	0.24(0.22,0.13,0.13,0.22)
133	74	21058	290(281,129,126,198)	6.25(6.06,2.78,2.72,4.27)
134	74	21091	11(5,2,2,2)	0.24(0.11,0.04,0.04,0.04)
135	74	22507	20(16,4,4,6)	0.43(0.35,0.09,0.09,0.13)
136	74	26199	11(1,0,0,0)	0.24(0.02,0.00,0.00,0.00)
137	75	137	46(43,0,0,0)	0.99(0.93,0.00,0.00,0.00)
138	75	21561	19(14,4,4,2)	0.41(0.30,0.09,0.09,0.04)
139	75	26486	55(49,24,24,30)	1.19(1.06,0.52,0.52,0.65)
140	78	21564	31(9,8,4,0)	0.67(0.19,0.17,0.09,0.00)
141	79	17389	72(1,0,0,0)	1.55(0.02,0.00,0.00,0.00)
142	63	2460	35(35,0,0,2)	0.75(0.75,0.00,0.00,0.04)
143	63	2791	19(19,14,14,16)	0.41(0.41,0.30,0.30,0.35)
144	63	3924	32(32,0,0,10)	0.69(0.69,0.00,0.00,0.22)
145	46	26422	27(27,4,4,4)	0.58(0.58,0.09,0.09,0.09)
146	63	4566	42(42,38,38,40)	0.91(0.91,0.82,0.82,0.86)
147	63	5785	237(234,66,63,112)	5.11(5.05,1.42,1.36,2.42)
148	63	8048	13(11,0,0,2)	0.28(0.24,0.00,0.00,0.04)
149	63	12514	21(1,0,0,0)	0.45(0.02,0.00,0.00,0.00)
150	63	22270	14(2,0,0,0)	0.30(0.04,0.00,0.00,0.00)

The numbers in the bracket are (reads with left primers, reads with right primers, reads with both p
Normalized count=(Read count/Total number of read mapped on reference genome)*1000000.
Total number of read mapped on reference is 46368772, excluding the mapped reads unpaired, not
“leader_end” and “TRS_start” refer to the position of the end of leader and the position of the start

rimers, same junction on paired reads with at least a primer).

: primary alignment and supplementary alignment.
: of TRS identified in the reads >10.

subgenome	leader_end	TRS_start	ACGAAC	ATG_postion	known_ATG	20_leader_se
1	47	26421	yes	26523	M	CAAACCAAC
2	64	6935	no	7025	-	GATCTCTTGT
3	65	3913	no	3948	-	ATCTCTTGTA
4	65	4577	no	4590	-	ATCTCTTGTA
5	65	5807	no	5859	-	ATCTCTTGTA
6	65	6495	no	6551	-	ATCTCTTGTA
7	65	8113	no	8133	-	ATCTCTTGTA
8	65	10020	no	10070	-	ATCTCTTGTA
9	65	10636	no	10662	-	ATCTCTTGTA
10	65	11664	no	11697	-	ATCTCTTGTA
11	48	26443	yes	26523	M	AAACCAACC
12	65	13730	no	13736	-	ATCTCTTGTA
13	66	736	no	785	-	TCTCTTGTAG
14	66	3360	no	3375	-	TCTCTTGTAG
15	66	5635	no	5681	-	TCTCTTGTAG
16	66	5773	no	5859	-	TCTCTTGTAG
17	66	6181	no	6243	-	TCTCTTGTAG
18	66	10857	no	10875	-	TCTCTTGTAG
19	66	13865	no	13898	-	TCTCTTGTAG
20	66	14679	no	14699	-	TCTCTTGTAG
21	66	22502	no	22614	-	TCTCTTGTAG
22	67	2194	no	2456	-	CTCTTGTAGA
23	67	2292	no	2456	-	CTCTTGTAGA
24	67	3362	no	3375	-	CTCTTGTAGA
25	67	3916	no	3948	-	CTCTTGTAGA
26	67	4018	no	4044	-	CTCTTGTAGA
27	67	5875	no	5919	-	CTCTTGTAGA
28	67	7505	no	7531	-	CTCTTGTAGA
29	67	8205	no	8214	-	CTCTTGTAGA
30	52	21491	no	21536	-	CAACCAACTT
31	67	13691	no	13736	-	CTCTTGTAGA
32	67	13916	no	13928	-	CTCTTGTAGA
33	67	14663	no	14699	-	CTCTTGTAGA
34	67	22278	no	22359	-	CTCTTGTAGA
35	67	22404	no	22407	-	CTCTTGTAGA
36	67	22490	no	22614	-	CTCTTGTAGA
37	67	22503	no	22614	-	CTCTTGTAGA
38	68	738	no	785	-	TCTTGTAGAT
39	68	2689	no	2745	-	TCTTGTAGAT
40	68	4580	no	4590	-	TCTTGTAGAT
41	68	5789	no	5859	-	TCTTGTAGAT
42	55	21538	yes	21563	S	CCAACTTTCG

43	68	8072	no	8081	-	TCTTGTAGAT
44	68	14223	no	14258	-	TCTTGTAGAT
45	68	14686	no	14699	-	TCTTGTAGAT
46	68	15777	no	15812	-	TCTTGTAGAT
47	55	26458	yes	26523	M	CCAACTTTCG
48	68	18051	no	18153	-	TCTTGTAGAT
49	68	21926	no	21936	-	TCTTGTAGAT
50	68	23365	no	23403	-	TCTTGTAGAT
51	55	26461	yes	26523	M	CCAACTTTCG
52	68	25425	no	25457	-	TCTTGTAGAT
53	69	705	no	726	-	CTTGTAGATC
54	69	1897	no	1977	-	CTTGTAGATC
55	69	3816	no	3842	-	CTTGTAGATC
56	69	7186	no	7210	-	CTTGTAGATC
57	69	7503	no	7531	-	CTTGTAGATC
58	69	9713	no	9768	-	CTTGTAGATC
59	69	10640	no	10662	-	CTTGTAGATC
60	69	11356	no	11367	-	CTTGTAGATC
61	69	12615	no	12634	-	CTTGTAGATC
62	69	13619	no	13625	-	CTTGTAGATC
63	69	13679	no	13685	-	CTTGTAGATC
64	69	18554	no	18594	-	CTTGTAGATC
65	69	19112	no	19148	-	CTTGTAGATC
66	69	19571	no	19631	-	CTTGTAGATC
67	69	21305	no	21317	-	CTTGTAGATC
68	69	21358	no	21397	-	CTTGTAGATC
69	69	22559	no	22614	-	CTTGTAGATC
70	70	913	no	966	-	TTGTAGATCT
71	70	2794	no	2802	-	TTGTAGATCT
72	70	2851	no	2871	-	TTGTAGATCT
73	70	3862	no	3948	-	TTGTAGATCT
74	70	6472	no	6551	-	TTGTAGATCT
75	70	7077	no	7089	-	TTGTAGATCT
76	70	10820	no	10844	-	TTGTAGATCT
77	70	11779	no	11849	-	TTGTAGATCT
78	70	11812	no	11849	-	TTGTAGATCT
79	70	14372	no	14381	-	TTGTAGATCT
80	70	14670	no	14699	-	TTGTAGATCT
81	70	22277	no	22359	-	TTGTAGATCT
82	70	22502	no	22614	-	TTGTAGATCT
83	70	22522	no	22614	-	TTGTAGATCT
84	70	27761	no	27825	-	TTGTAGATCT
85	71	734	no	785	-	TGTAGATCTG

86	71	4586	no	4590	-	TGTAGATCTG
87	71	4592	no	4607	-	TGTAGATCTG
88	71	5594	no	5681	-	TGTAGATCTG
89	71	5708	no	5718	-	TGTAGATCTG
90	71	10772	no	10791	-	TGTAGATCTG
91	71	12208	no	12219	-	TGTAGATCTG
92	71	12412	no	12417	-	TGTAGATCTG
93	71	13313	no	13338	-	TGTAGATCTG
94	71	13583	no	13625	-	TGTAGATCTG
95	59	735	no	785	-	CTTTCGATCT
96	71	18943	no	18956	-	TGTAGATCTG
97	71	22403	no	22407	-	TGTAGATCTG
98	71	22409	no	22422	-	TGTAGATCTG
99	71	22498	no	22614	-	TGTAGATCTG
100	71	23926	no	24051	-	TGTAGATCTG
101	60	2285	no	2456	-	TTTCGATCTC
102	72	2689	no	2745	-	GTAGATCTGT
103	60	2613	no	2630	-	TTTCGATCTC
104	72	9284	no	9288	-	GTAGATCTGT
105	72	9889	no	9926	-	GTAGATCTGT
106	72	15808	no	15812	-	GTAGATCTGT
107	72	22107	no	22155	-	GTAGATCTGT
108	72	22511	no	22614	-	GTAGATCTGT
109	60	4007	no	4044	-	TTTCGATCTC
110	73	3372	no	3375	-	TAGATCTGTT
111	73	3776	no	3780	-	TAGATCTGTT
112	73	4027	no	4044	-	TAGATCTGTT
113	73	8145	no	8148	-	TAGATCTGTT
114	73	8957	no	9019	-	TAGATCTGTT
115	73	14371	no	14381	-	TAGATCTGTT
116	60	13994	no	14021	-	TTTCGATCTC
117	73	22285	no	22359	-	TAGATCTGTT
118	73	23028	no	23046	-	TAGATCTGTT
119	74	165	no	266	-	AGATCTGTTC
120	60	20306	no	20309	-	TTTCGATCTC
121	74	5890	no	5919	-	AGATCTGTTC
122	74	5893	no	5919	-	AGATCTGTTC
123	74	6721	no	6770	-	AGATCTGTTC
124	74	6724	no	6770	-	AGATCTGTTC
125	74	6853	no	7025	-	AGATCTGTTC
126	74	7704	no	7758	-	AGATCTGTTC
127	74	12407	no	12417	-	AGATCTGTTC
128	74	12742	no	12759	-	AGATCTGTTC

129	74	12974	no	12986	-	AGATCTGTTC
130	74	13630	no	13685	-	AGATCTGTTC
131	60	22933	no	23004	-	TTTCGATCTC
132	74	19227	no	19283	-	AGATCTGTTC
133	74	21058	no	21071	-	AGATCTGTTC
134	74	21091	no	21188	-	AGATCTGTTC
135	74	22507	no	22614	-	AGATCTGTTC
136	74	26199	no	26231	-	AGATCTGTTC
137	75	137	no	266	-	GATCTGTTCT
138	75	21561	no	21563	S	GATCTGTTCT
139	75	26486	no	26523	M	GATCTGTTCT
140	78	21564	no	21744	-	CTGTTCTCTA
141	79	17389	no	17399	-	TGTTCTCTAA
142	63	2460	no	2630	-	CGATCTCTTG
143	63	2791	no	2802	-	CGATCTCTTG
144	63	3924	no	3948	-	CGATCTCTTG
145	46	26422	yes	26523	M	ACAAACCAAC
146	63	4566	no	4590	-	CGATCTCTTG
147	63	5785	no	5859	-	CGATCTCTTG
148	63	8048	no	8081	-	CGATCTCTTG
149	63	12514	no	12519	-	CGATCTCTTG
150	63	22270	no	22359	-	CGATCTCTTG

“peak_leader” and “peak_TRS_start” point to the leader-TRS junctions in sheet "novel", “ACGAAC”

TRS_seq	first_orf_aa
CTCTCGTGTT	MADSNGTITVEELKKLLEQWNLVIGFLFLTWICLLQFAYANRNRFLYIIKLIFLWLLWPVTLACI
TCTAAACTGA	MSNLGMPSYCTGYREGYLNSTNVTIATYCTGSIPCSVCLSGLDSLDTYPSLETIQITISSFKWDL
TGAAAGTAA	MIRKSKLVLKKLQQLWKKLSSSQKTCYFILTLMAIFIQILPLLLVTLTSLS*
CTTAACGATC	MKLLLQCHLAM*
ATAGACGGT	MFSTKKTVTQQP*
TTAAACCAGC	MAAYVDNSSLTIKKPNELSRVLGLKTLATHGLAAVNSVPWDTIANYAKPFLNKVVSTTTNIVT
AGAAGCTGA	MCP*
CACCACAAA	MAFPSGKVEGCMVQVTCGTTTLNGLWLDDVVYCPRHVICTSEDMLNPNYEDLLIRKSNHN
TGGTACGGA	MF*
TCAACCGCTA	MIT*
TCTTCTAGAG	MADSNGTITVEELKKLLEQWNLVIGFLFLTWICLLQFAYANRNRFLYIIKLIFLWLLWPVTLACI
CTAAAC	MTSLSLE*
TCAAGAAAA	MRELNGGAYTRYVDNNFCGPDGYPLECIKDLLARAGKASCTLSEQLDFIDTKRGVYCCREHE
TAAAACTTAC	MYTLKMQTLWKKLKR*
TAAACAAGC	MMSAPPAQYELKHGTFTCASEYTGNYQCGHYKHITSKETLYCIDGALLTKSSEYKGPITDVFYI
TAAACATATA	MFSTKKTVTQQP*
TAAACACTAC	MLTMQLIKPRINQIPGVYVVFGAQNQLKHQIRLMY*
TAAAAGAATT	MV*
TAAAAGAAA	MMIISIKRTGMIL*
TAATTTTAAC	MTLLCLRVSLRKEVLLN*
CAAACTTCTA	MLGTGRESATVLLIILSYIIPHHFPLLSVMECLLLN*
AGACGGTTG	MPLKAPKEIIFLEGETLPTEVLTEEVVLKTGDLQPLEQPTSEAVEAPLVGTPVCINGLMLLEIKD
AGACATTCTT	MPLKAPKEIIFLEGETLPTEVLTEEVVLKTGDLQPLEQPTSEAVEAPLVGTPVCINGLMLLEIKD
AAACTTACTG	MYTLKMQTLWKKLKR*
AAGTAAACC	MIRKSKLVLKKLQQLWKKLSSSQKTCYFILTLMAIFIQILPLLLVTLTSLS*
AAACTTGTTA	MAIFIQILPLLLVTLTSLS*
AAACAGTTA	MVLFVQKLTLSWTIIIRKTILISQSNQLILYQTNHIQTQASIILSLYVIISNLLMI*
AAACGTAAT	MYNYC*
AAACTAAAG	MLLNVLNCHINLT*
GTAGACTTAT	MFLLTTKRTMFVFLVLLPLVSSQCVNLTTRTQLPPAYTNSFTRGVYYPDKVFRSSVLHSTQDLI
AAACAATTTA	MTSLSLE*
AAAAGGACT	MIL*
AAACTGTCA	MTLLCLRVSLRKEVLLN*
AAACTTTACT	MWVIFNLGLFY*
AAA	MEPLQML*
AAAGGAATC	MLGTGRESATVLLIILSYIIPHHFPLLSVMECLLLN*
AAACTTCTAA	MLGTGRESATVLLIILSYIIPHHFPLLSVMECLLLN*
AAGAAAACT	MRELNGGAYTRYVDNNFCGPDGYPLECIKDLLARAGKASCTLSEQLDFIDTKRGVYCCREHE
AACAAACAA	MTL*
AACGATCTA	MKLLLQCHLAM*
AAAGAAACT	MFSTKKTVTQQP*
GTTCTTGTTA	MFVFLVLLPLVSSQCVNLTTRTQLPPAYTNSFTRGVYYPDKVFRSSVLHSTQDLFLPFFSNVTW

AACGTACCA	MEKLKTLVATAEAELAKNVSLDNVLSTFISAARQGFVDSDVETKDVVECLKLSHQSDIEVTGD
AACAAAGCCT	MTSRKRG*
AACAAAGACT	MTLLCLRVSLRKEVLLN*
AAAGAACTTT	MFLCLKQNVGLRLTLLKDLMNFALNIQC*
GATCTTCTGG	MADSNGTITVEELKKLLEQWNLVIGFLFLTWICLLQFAYANRNRFLYIIKLIFLWLLWPVTLACI
AACAGGACTC	MC*
AACGCTACTA	MLLLKSVNFNFVMIHFWVFITTKTTKVGWKVSSEFILVRIIALLNMSLSLFLWTLKENRVISKIL
AACAAATACT	MLTAQKSLLLFMQINLLLLGVFILQVLMFFKHVQAV*
CTTCTGGTCT	MADSNGTITVEELKKLLEQWNLVIGFLFLTWICLLQFAYANRNRFLYIIKLIFLWLLWPVTLACI
AACTGTAACT	MLLLQILFALLQRYRYKPHSLSDGLLLALHFLLFFRALPKS*
ACGAGCTTGC	MKIFKKTGTLNIAVVLPVNSCVSLTEGHTLAMSITTSVALMATLLSALKTF*
ACGATCAATT	MEFHSIH*
ACAAACTTGT	MKSEKQVEQKIAEIPKEEVKPFITESKPSVEQRKQDDKKIKACVEEVTTTLEETKFLTENLLLYID
ACAAATTACC	MGFNCFWLSCRVVFGIYSFH*
ACAAACGTAA	MYNYC*
ACAAAGCATT	MVFPLVLLKKLRCAPFC*
ACGGACACAA	MF*
AAGAACTGTC	MMMVLGECGHL*
ACAATTCACC	MASYCNSFKGQFCCQITE*
ACGAAG	MTI*
ACCAAC	MKKQFIIYLRIVQLLLNMTSLSLE*
ACACACTTAA	MGTWL*
ACAAAGCTTA	MPHILTNSQMVYAYFGIAMSIDILLIPLFVDLTLECYLTLTCLVVMVAVCM*
ACAAACAATT	MWLLML*
GCGAACAAAT	MVMSCMQITYFGGIQIQFSCLPILYLT*
ACAAATCCAA	MSKFPLKLRGTAVMSLKEGQINDMILSLLSKGRLIIRENNRVVISSDVLVNN*
ACAAACTTGT	MLGTGRESATVLLIILSYIIPHHFPLLSVMECLLLN*
CGAACAACTC	MSMKLLGTRNVLKRAMNCRHLLKLNWQRNLTPSMGNVQILYFP*
TGAACTTG	MKGLIKYLMRSALPIQLNSVQK*
TGAACTCGGT	MSSPVLWQMLS*
TGAACAAAAC	MIRKSKLVLKKLQQLWKKLSSSQKTCYFILTLMAIFIQILPLLLVTLTSLS*
CGAAGTTGTA	MAAYVDNSSLTIKKPNELSRVLGLKTLATHGLAAVNSVPWDTIANYAKPFLNKVVSTTTNIVT
TGAACTCTAC	MSLLQPTVLVLYLVVFVLVV*
CAAACTGGAA	MCASLKELLQNGMNGRTILGSALLEDEFTPFDVVRQCSGVTFQSAVKRTIKGTHHWLLLTILT
CAAACTCAAC	MSDVKCTSVVLLSVLQQLRVESSSKLWAQCVQLHNDILLAKDTTEAFEKMVSLLSVLLSMQG
CAAACCTTGT	MSDVKCTSVVLLSVLQQLRVESSSKLWAQCVQLHNDILLAKDTTEAFEKMVSLLSVLLSMQG
CAAACTTTA	MFYSLQCSHLQVLDH*
CAAACCCGGT	MTLLCLRVSLRKEVLLN*
CAAACTTTAC	MWVIFNLGLFY*
CAAACTTCTA	MLGTGRESATVLLIILSYIIPHHFPLLSVMECLLLN*
CCAACCAACA	MLGTGRESATVLLIILSYIIPHHFPLLSVMECLLLN*
TGAACTTTCA	MLIIFWFSLELQDHNETCHA*
TTTCAAGAAA	MRELNGGAYTRYVDNNFCGPDGYPLECIKDLLARAGKASCTLSEQLDFIDTKRGVYCCREHE

CTAA	MKLLLQCHLAM*
GAAACTCTTG	MPLGYVTHGLNLEEAARYMRSLKVPATVSVSSPDAVTAYNGYLTSSSKTPEEHFIETISLAGSY
GAACAATTTA	MMSAPPAQYELKHGTFTCASEYTGNYQCGHYKHITSKETLYCIDGALLTKSSEYKGPITDVFYI
GAACTTAAGC	MVHLLVLVSTLVITSVVTINI*
GAACCTCTAA	MLTY*
GAAGTCTTTG	MWLNLNLTVMQPCNVSWKRWLIKL*
CAACA	MQEMVVFP*
GTACAAATAC	MTLWVLHLKTQSVPSAVCGKVMAVVVINSANPCFSQLMHNRF*
TCCTAAAAAC	MTI*
TTCAAGAAAA	MRELNGGAYTRYVDNNFCGPDGYPLECIKDLLARAGKASCTLSEQLDFIDTKRGVYCCREHE
GAACTGAAGA	MRLVERFNTWLLKLHY*
GAAA	MEPLQML*
GGAACCATTA	ML*
CTATCAAACT	MLGTGRESATVLLIILSYIIPHHFPLLSVMECLLLN*
TTACAAAACA	MLASSNNMVIALVILLLETSFVHKSLTALLFCHLCSQMK*
AGTGTTCAGA	MPLKAPKEIIFLEGETLPTEVLTEEVVLKTGDLQPLEQPTSEAVEAPLVGTPVCINGLMLLEIKD
AACAAACAAT	MTL*
TTTGTATTAA	MLLEIKDTEKYCALAPNMMVTNNTFTLKGGAPTKVTFGDDTVIEVQGYKSVNITFELDERIDI
AACA	MIITDLYQEFSVV*
AGCTCTTTATA	MDTTSYREAACCHLAKALNDFSNSGSDVLYQPPQTSITSAVLQSGFRKMAFPSGKVEGCMV
AACA	MFLCLKQNVGLRLTLLKDLMNFALNIQC*
AACAGGGTAA	MVILKYILSTRLLI*
AACTTTAGAG	MLGTGRESATVLLIILSYIIPHHFPLLSVMECLLLN*
TTCCTCACAG	MAIFIQILPLLLVTLTSLS*
ACA	MYTLKMQTLWKKLKR*
ACAA	MST*
ACTTTATATTC	MAIFIQILPLLLVTLTSLS*
ACA	MSYLLLFQQLGKGLLIQM*
ACACCATCAA	MYNF*
GCAAACTTTA	MFYSLQCSHLQVLDH*
CTTTGTTAAA	MPCEMLVLLVY*
ACTTGCTTTA	MWVIFNLGLFY*
ACTTTCCTTTA	MVSNPLMVLVTNHTE*
GAGTAACTCC	MESLVPGFNEKTHVQLSLPVLQVRDVLVRGFGDSVEEVLSEARQHLKDGTCGLVEVEKGVLF
GCT	MPSNISFMEILVIVS*
AACCATAAAA	MVLFVQKLTLSWTIIIRKTILISQSNQLILYQTNHIQTQASIILSLYVIISNLLMI*
CATAAAACCA	MVLFVQKLTLSWTIIIRKTILISQSNQLILYQTNHIQTQASIILSLYVIISNLLMI*
AACTACTAAC	MPYFFTLLLQLCTFTRSTNSRIKASMPTTIAKNTVKSVGKFCLEASFNYLKSPNFSKLINIIIWFLI
TACTAACATA	MPYFFTLLLQLCTFTRSTNSRIKASMPTTIAKNTVKSVGKFCLEASFNYLKSPNFSKLINIIIWFLI
TACTATAGCA	MSNLGMPSYCTGYREGYLNSTNVTIATYCTGSIPCSVCLSGLDSLDTYPSLETIQITISSFKWDL
CAATAAATCC	MVPSIFTLIKLVKRLMKDILSLILLT*
ATTATCAACA	MQEMVVFP*
ACAAACTGCT	MTMR*

CTAAATAGA	MVLGSLAATVRLQAGNATEVPANSTVLSFCAFAVDAAKAYKDYLASGGQPITNCVKMLCTH
AATTTAATTG	MKKQFIIYLRIVQLLLNMTSLSLE*
GAAGTCTAAT	MVLKVLIVTFLYNHMVSNPLMVLVTNHTE*
CATTGTTTGT	MVAVCM*
CCTAAGACTA	MLQKKMTLKRVFSLTFVGLYNKS*
TCTAAAGAG	MLIFISSWDTSHGGQPLLLM*
TTCTAACTTT	MLGTGRESATVLLIILSYIIPHHFPLLSVMECLLLN*
GACTACTAG	MSTNLCTHSFRKRQVR*
TAACTAATTA	MESLVPGFNEKTHVQLSLPVLQVRDVLVRGFGDSVEEVLSEARQHLKDGTCGLVEVEKGVL
CA	MFVFLVLLPLVSSQCVNLTTRTQLPPAYTNSFTRGVYYPDKVFRSSVLHSTQDLFLPFFSNVTV
TATATTAGTT	MADSNGTITVEELKKLLEQWNLVIGFLFLTWICLLQFAYANRNRFLYIIKLIFLWLLWPVTLAC
TGTTTGTTTT	MLLGSMLYMSLGPMVLRGLITLSYHLMMVFILLPLRSLT*
AGTGTTGTCA	MPDYVLSTMCTLATLLNYLHHAHC*
CTCTAAAAG	MLLEIKDTEKYCALAPNMMVTNNTFTLKGGAPTKVTFGDDTVIEVQGYKSVNITFELDERID
TTTTGAACTT	MKGLIKYLMRSALPIQLNSVQK*
CTTCAGTTGA	MIRKSKLVLKKLQQLWKKLSSSQKTCYFILTLMAIFIQILPLLLVTLTSLS*
TCTCGTGTTA	MADSNGTITVEELKKLLEQWNLVIGFLFLTWICLLQFAYANRNRFLYIIKLIFLWLLWPVTLAC
TTATCAACAC	MKLLLQCHLAM*
TTCTAAAGAA	MFSTKKTVTQQP*
GTTAATACGT	MEKLKTLVATAEAELAKNVSLDNVLSTFISAARQGFVDSDVETKDVVECLKLSHQSDIEVTGD
GTGTG	MVQHLLMHQHCGKSNRL*
TAGGTTTCAA	MWVIFNLGLFY*

indicates if there is a ACGAAC sequences in the “TRS_seq” (TRS sequences), “20_leader_seq” re

FVLAAVYRINWITGGIAIAMACLVGLMWLSYFIASFRLFARTRSMWSFNPETNILLNVPLHGTILTRPLLESELV
.TAFGLVAEWFLAYILFTRFFYVLGLAAIMQLFFSYFAVHFISNSWLMWLIINLVQMAPISAMVRMYIFFASFYY

TRCLNRVCTNYMPYFFTLLLQLCTFTRSTNSRIKASMPTTIAKNTVKSVGKFCLEASFNYLKSPNFSKLINIIIWFLL

FLVQAGNVQLRVIGHSMQNCVLKLKVDTANPKTPKYKFVRIQPGQTFSVLACYNGSPSGVYQCAMRPNFTIK

FVLAAVYRINWITGGIAIAMACLVGLMWLSYFIASFRLFARTRSMWSFNPETNILLNVPLHGTILTRPLLESELV

HEIAWYTERSEKSYELQTPFEIKLAKKFDTFNGECPNFVFPLNSIIKTIQPRVEKKKLDGFMGRIRSVYPVASPNE

KENSYTTTIKPVTYKLDGVVCTEIDPKLDNYYKKDNSYFTEQPIDLVPNQPYPNASFDNFKFVCDNIKFADDLNC

TEKYCALAPNMMVTNNTFTLKGGAPTKVTFGDDTVIEVQGYKSVNITFELDERIDKVLNEKCSAYTVELGTEV
TEKYCALAPNMMVTNNTFTLKGGAPTKVTFGDDTVIEVQGYKSVNITFELDERIDKVLNEKCSAYTVELGTEV

FLPFFSNVTWFHAIHVSGTNGTKRFDNPVLPFNDGVYFASTEKSNIIRGWIFGTTLDSKTQSLLIVNNATNVVIK

HEIAWYTERSEKSYELQTPFEIKLAKKFDTFNGECPNFVFPLNSIIKTIQPRVEKKKLDGFMGRIRSVYPVASPNE

WFHAIHVSGTNGTKRFDNPVLPFNDGVYFASTEKSNIIRGWIFGTTLDSKTQSLLIVNNATNVVIKVCEFQFCN

SCNNYMLTYNKVENMTPRDLGACIDCSARHINAQVAKSHNIALIWNVKDFMSLSEQLRKQIRSAAKKNNLP

FVLAAVYRINWITGGIAIAMACLVGLMWLSYFIASFRLFARTRSMWSFNPETNILLNVPLHGTILTRPLLESELV

GNLCLRILMVILKYILSTRLLI*

FVLAAVYRINWITGGIAIAMACLVGLMWLSYFIASFRLFARTRSMWSFNPETNILLNVPLHGTILTRPLLESELV

INGNLHPDSATLVSDIDITFLKKDAPYIVGDVVQEGVLTAVVIPTKKAGGTTEMLAKALRKVPTDNYITTYPG

RCLNRVCTNYMPYFFTLLLQLCTFTRSTNSRIKASMPTTIAKNTVKSVGKFCLEASFNYLKSPNFSKLINIIIWFL

TSLLVLVQSTQWSLFFFLYENAFLPFAMGIIAMSAFAMMFVKHKHAFLCLFLLPSLATVAYFNMVYMPASWV

AVDINKLCEEMLDNRATLQAIASEFSSLPSYAAFATAQEAYEQAVANGDSEVVLKKLKKSLNVAKSEFDRDA

AVDINKLCEEMLDNRATLQAIASEFSSLPSYAAFATAQEAYEQAVANGDSEVVLKKLKKSLNVAKSEFDRDA

HEIAWYTERSEKSYELQTPFEIKLAKKFDTFNGECPNFVFPLNSIIKTIQPRVEKKKLDGFMGRIRSVYPVASPNE

KDWSYSGQSTQLGIEFLKRGDKSVYYTSNPTTFHLDGEVITFDNLKTLLSLREVRTIKVFTTVDNINLHTQVVDI
KENSYTTTIKPVTYKLDGVVCTEIDPKLDNYYKKDNSYFTEQPIDLVPNQPYPNASFDNFKFVCDNIKFADDLN

HEIAWYTERSEKSYELQTPFEIKLAKKFDTFNGECPNFVFPLNSIIKTIQPRVEKKKLDGFMGRIRSVYPVASPNE

TEKYCALAPNMMVTNNTFTLKGGAPTKVTFGDDTVIEVQGYKSVNITFELDERIDKVLNEKCSAYTVELGTEV

KVLNEKCSAYTVELGTEVNEFACVVADAVIKTLQPVSELLTPLGIDLDEWSMATYYLFDESGEFKLASHMYCSF

QVTCGTTTLNGLWLDDVVYCPRHVICTSEDMLNPNYEDLLIRKSNHNFLVQAGNVQLRVIGHSMQNCVLKL

QLEQPYVFIKRSDARTAPHGHVMVELVAELEGIQYGRSGETLGVLVPHVGEIPVAYRKVLLRKNGNKGAGG

LLSVCLGSLIYSTAALGVLMSNLGMPSYCTGYREGYLNSTNVTIATYCTGSIPCSVCLSGLDSLDTYPSLETIQITIS
LLSVCLGSLIYSTAALGVLMSNLGMPSYCTGYREGYLNSTNVTIATYCTGSIPCSVCLSGLDSLDTYPSLETIQITIS
TAFGLVAEWFLAYILFTRFFYVLGLAAIMQLFFSYFAVHFISNSWLMWLIINLVQMAPISAMVRMYIFFASFYY

ITGTGQAITVTPEANMDQESFGGASCCLYCRCHIDHPNPKGFCDLKGKYVQIPTTCANDPVGFTLKNTVCTVC

PQLEQPYVFIKRSDARTAPHGHVMVELVAELEGIQYGRSGETLGVLVPHVGEIPVAYRKVLLRKNGNKGAGGH
WFHAIHVSGTNGTKRFDNPVLPFNDGVYFASTEKSNIIRGWIFGTTLDSKTQSLLIVNNATNVVIKVCEFQFCN
FVLAAVYRINWITGGIAIAMACLVGLMWLSYFIASFRLFARTRSMWSFNPETNILLNVPLHGTILTRPLLESELV

KVLNEKCSAYTVELGTEVNEFACVVADAVIKTLQPVSELLTPLGIDLDEWSMATYYLFDESGEFKLASHMYCSF

FVLAAVYRINWITGGIAIAMACLVGLMWLSYFIASFRLFARTRSMWSFNPETNILLNVPLHGTILTRPLLESELV

DSCNNYMLTYNKVENMTPRDLGACIDCSARHINAQVAKSHNIALIWNVKDFMSLSEQLRKQIRSAAKKNNLP

efers to the 20 sequences before the end of the leader, “AUG_postion” and “first_orf_aa” refer

IGAVILRGHLRIAGHHLGRCDIKDLPKEITVATSRTLSYYKLGASQRVAGDSGFAAYSRYRIGNYKLNTDHSSSSD
VWKSYVHVVDGCNSSTCMMCYKRNRATRVECTTIVNGVRRSFYVYANGGKGFCKLHNWNCVNCDTFCAG

LSVCLGSLIYSTAALGVLMSNLGMPSYCTGYREGYLNSTNVTIATYCTGSIPCSVCLSGLDSLDTYPSLETIQITIS

GSFLNGSCGSVGFNIDYDCVSFCYMHHMELPTGVHAGTDLEGNFYGPFVDRQTAQAAGTDTTITVNVLAW

IGAVILRGHLRIAGHHLGRCDIKDLPKEITVATSRTLSYYKLGASQRVAGDSGFAAYSRYRIGNYKLNTDHSSSSD

CNQMCLSTLMKCDHCGETSWQTGDFVKATCEFCGTENLTKEGATTCGYLPQNAVVKIYCPACHNSEVGPE

QLTGYKKPASRELKVTFFPDLNGDVVAIDYKHYTPSFKKGAKLLHKPIVWHVNNATNKATYKPNTWCIRCLWS

NEFACVVADAVIKTLQPVSELLTPLGIDLDEWSMATYYLFDESGEFKLASHMYCSFYPPDEDEEEGDCEEEEFE
NEFACVVADAVIKTLQPVSELLTPLGIDLDEWSMATYYLFDESGEFKLASHMYCSFYPPDEDEEEGDCEEEEFE

VCEFQFCNDPFLGVYYHKNNKSWMESEFRVYSSANNCTFEYVSQPFLMDLEGKQGNFKNLREFVFKNIDGY

CNQMCLSTLMKCDHCGETSWQTGDFVKATCEFCGTENLTKEGATTCGYLPQNAVVKIYCPACHNSEVGPE

DPFLGVYYHKNNKSWMESEFRVYSSANNCTFEYVSQPFLMDLEGKQGNFKNLREFVFKNIDGYFKIYSKHTPI

FKLTCATTRQVVNVVTTKIALKGGKIVNNWLKQLIKVTLVFLFVAAIFYLITPVHVMSKHTDFSSEIIGYKAIDGG

IGAVILRGHLRIAGHHLGRCDIKDLPKEITVATSRTLSYYKLGASQRVAGDSGFAAYSRYRIGNYKLNTDHSSSS

IGAVILRGHLRIAGHHLGRCDIKDLPKEITVATSRTLSYYKLGASQRVAGDSGFAAYSRYRIGNYKLNTDHSSSS

GLNGYTVEEAKTVLKKCKSAFYILPSIISNEKQEILGTVSWNLREMLAHAEETRKLMPVCVETKAIVSTIQRKYK

LSVCLGSLIYSTAALGVLMSNLGMPSYCTGYREGYLNSTNVTIATYCTGSIPCSVCLSGLDSLDTYPSLETIQITIS

MRIMTWLDMVDTSLSGFKLKDCVMYASAVVLLILMTARTVYDDGARRVWTLMNVLTLVYKVYYGNALDQ

MQRKLEKMADQAMTQMYKQARSEDKRAKVTSAMQTMLFTMLRKLDNDALNNIINNARDGCVPLNIIPLT

MQRKLEKMADQAMTQMYKQARSEDKRAKVTSAMQTMLFTMLRKLDNDALNNIINNARDGCVPLNIIPLT

CNQMCLSTLMKCDHCGETSWQTGDFVKATCEFCGTENLTKEGATTCGYLPQNAVVKIYCPACHNSEVGPE

MSMTYGQQFGPTYLDGADVTKIKPHNSHEGKTFYVLPNDDTLRVEAFEYYHTTDPSFLGRYMSALNHTKKW
QLTGYKKPASRELKVTFFPDLNGDVVAIDYKHYTPSFKKGAKLLHKPIVWHVNNATNKATYKPNTWCIRCLWS

CNQMCLSTLMKCDHCGETSWQTGDFVKATCEFCGTENLTKEGATTCGYLPQNAVVKIYCPACHNSEVGPEH

NEFACVVADAVIKTLQPVSELLTPLGIDLDEWSMATYYLFDESGEFKLASHMYCSFYPPDEDEEEGDCEEEEFE

YPPDEDEEEGDCEEEEFEPSTQYEYGTEDDYQGKPLEFGATSAALQPEEEQEEDWLDDDSQQTVGQQDGSE

LKVDTANPKTPKYKFVRIQPGQTFSVLACYNGSPSGVYQCAMRPNFTIKGSFLNGSCGSVGFNIDYDCVSFCYN

HSYGADLKSFDLGDELGTDPYEDFQENWNTKHSSGVTRELMRELNGGAYTRYVDNNFCGPDGYPLECIKDLL

SSFKWDLTAFGLVAEWFLAYILFTRFFYVLGLAAIMQLFFSYFAVHFISNSWLMWLIINLVQMAPISAMVRMY
SSFKWDLTAFGLVAEWFLAYILFTRFFYVLGLAAIMQLFFSYFAVHFISNSWLMWLIINLVQMAPISAMVRMY
VWKSYVHVVDGCNSSTCMMCYKRNRATRVECTTIVNGVRRSFYVYANGGKGFCKLHNWNCVNCDTFCAG

CGMWKGYGCSCDQLREPMLQSADAQSFLNGFAV*

HSYGADLKSFDLGDELGTDPYEDFQENWNTKHSSGVTRELMRELNGGAYTRYVDNNFCGPDGYPLECIKDLL
DPFLGVYYHKNNKSWMESEFRVYSSANNCTFEYVSQPFLMDLEGKQGNFKNLREFVFKNIDGYFKIYSKHTPI
IGAVILRGHLRIAGHHLGRCDIKDLPKEITVATSRTLSYYKLGASQRVAGDSGFAAYSRYRIGNYKLNTDHSSSD

YPPDEDEEEGDCEEEEFEPSTQYEYGTEDDYQGKPLEFGATSAALQPEEEQEEDWLDDDSQQTVGQQDGSE

IGAVILRGHLRIAGHHLGRCDIKDLPKEITVATSRTLSYYKLGASQRVAGDSGFAAYSRYRIGNYKLNTDHSSSD

FKLTCATTRQVVNVVTTKIALKGGKIVNNWLKQLIKVTLVFLFVAAIFYLITPVHVMSKHTDFSSEIIGYKAIDGG

to the first AUG position and translated orf of the sgmRNA, and “known_ATG” indicates if the fi

NIALLVQ*
STFISDEVARDLSLQFKRPINPTDQSSYIVDSVTVKNGSIHLYFDKAGQKTYERHSLSHFVNLDNLRANNTKGS

SFKWDLTAFGLVAEWFLAYILFTRFFYVLGLAAIMQLFFSYFAVHFISNSWLMWLIINLVQMAPISAMVRMY

LYAAVINGDRWFLNRFTTTLNDFNLVAMKYNYEPLTQDHVDILGPLSAQTGIAVLDMCASLKELLQNGMN

NIALLVQ*

HSLAEYHNESGLKTILRKGGRTIAFGGCVFSYVGCHNKCAYWVPRASANIGCNHTGVVGEGSEGLNDNLLEIL

TKPVETSNSFDVLKSEDAQGMDNLACEDLKPVSEEVVENPTIQKDVLECNVKTTEVVGDIILKPANNSLKITE

PSTQYEYGTEDDYQGKPLEFGATSAALQPEEEQEEDWLDDDSQQTVGQQDGSEDNQTTTIQTIVEVQPQL
PSTQYEYGTEDDYQGKPLEFGATSAALQPEEEQEEDWLDDDSQQTVGQQDGSEDNQTTTIQTIVEVQPQL

FKIYSKHTPINLVRDLPQGFSALEPLVDLPIGINITRFQTLLALHRSYLTPGDSSSGWTAGAAAYYVGYLQPRTF

HSLAEYHNESGLKTILRKGGRTIAFGGCVFSYVGCHNKCAYWVPRASANIGCNHTGVVGEGSEGLNDNLLEIL

NLVRDLPQGFSALEPLVDLPIGINITRFQTLLALHRSYLTPGDSSSGWTAGAAAYYVGYLQPRTFLLKYNENGT

VTRDIASTDTCFANKHADFDTWFSQRGGSYTNDKACPLIAAVITREVGFVVPGLPGTILRTTNGDFLHFLPRVI

NIALLVQ*

NIALLVQ*

GIKIQEGVVDYGARFYFYTSKTTVASLINTLNDLNETLVTMPLGYVTHGLNLEEAARYMRSLKVPATVSVSSPD

SFKWDLTAFGLVAEWFLAYILFTRFFYVLGLAAIMQLFFSYFAVHFISNSWLMWLIINLVQMAPISAMVRMYI

AISMWALIISVTSNYSGVVTTVMFLARGIVFMCVEYCPIFFITGNTLQCIMLVYCFLGYFCTCYFGLFCLLNRYFR

TAAKLMVVIPDYNTYKNTCDGTTFTYASALWEIQQVVDADSKIVQLSEISMDNSPNLAWPLIVTALRANSAV

TAAKLMVVIPDYNTYKNTCDGTTFTYASALWEIQQVVDADSKIVQLSEISMDNSPNLAWPLIVTALRANSAV

HSLAEYHNESGLKTILRKGGRTIAFGGCVFSYVGCHNKCAYWVPRASANIGCNHTGVVGEGSEGLNDNLLEIL

KYPQVNGLTSIKWADNNCYLATALLTLQQIELKFNPPALQDAYYRARAGEAANFCALILAYCNKTVGELGDVR
STKPVETSNSFDVLKSEDAQGMDNLACEDLKPVSEEVVENPTIQKDVLECNVKTTEVVGDIILKPANNSLKITEE

HSLAEYHNESGLKTILRKGGRTIAFGGCVFSYVGCHNKCAYWVPRASANIGCNHTGVVGEGSEGLNDNLLEIL

EPSTQYEYGTEDDYQGKPLEFGATSAALQPEEEQEEDWLDDDSQQTVGQQDGSEDNQTTTIQTIVEVQPQLE

DNQTTTIQTIVEVQPQLEMELTPVVQTIEVNSFSGYLKLTDNVYIKNADIVEEAKKVKPTVVVNAANVYLKHG

MHHMELPTGVHAGTDLEGNFYGPFVDRQTAQAAGTDTTITVNVLAWLYAAVINGDRWFLNRFTTTLNDFN

ARAGKASCTLSEQLDFIDTKRGVYCCREHEHEIAWYTERSEKSYELQTPFEIKLAKKFDTFNGECPNFVFPLNSII

IFFASFYYVWKSYVHVVDGCNSSTCMMCYKRNRATRVECTTIVNGVRRSFYVYANGGKGFCKLHNWNCVN
IFFASFYYVWKSYVHVVDGCNSSTCMMCYKRNRATRVECTTIVNGVRRSFYVYANGGKGFCKLHNWNCVN
GSTFISDEVARDLSLQFKRPINPTDQSSYIVDSVTVKNGSIHLYFDKAGQKTYERHSLSHFVNLDNLRANNTKGS

ARAGKASCTLSEQLDFIDTKRGVYCCREHEHEIAWYTERSEKSYELQTPFEIKLAKKFDTFNGECPNFVFPLNSII
NLVRDLPQGFSALEPLVDLPIGINITRFQTLLALHRSYLTPGDSSSGWTAGAAAYYVGYLQPRTFLLKYNENGTI
)NIALLVQ*

DNQTTTIQTIVEVQPQLEMELTPVVQTIEVNSFSGYLKLTDNVYIKNADIVEEAKKVKPTVVVNAANVYLKHG(

)NIALLVQ*

VTRDIASTDTCFANKHADFDTWFSQRGGSYTNDKACPLIAAVITREVGFVVPGLPGTILRTTNGDFLHFLPRVI

rst AUG position is the same as a known sgmRNA.

LPINVIVFDGKSKCEESSAKSASVYYSQLMCQPILLLDQALVSDVGDSAEVAVKMFDAYVNTFSSTFNVPMEK

FFASFYYVWKSYVHVVDGCNSSTCMMCYKRNRATRVECTTIVNGVRRSFYVYANGGKGFCKLHNWNCVN

GRTILGSALLEDEFTPFDVVRQCSGVTFQSAVKRTIKGTHHWLLLTILTSLLVLVQSTQWSLFFFLYENAFLPFAM

QKEKVNINIVGDFKLNEEIAIILASFSASTSAFVETVKGLDYKAFKQIVESCGNFKVTKGKAKKGAWNIGEQKSIL

EVGHTDLMAAYVDNSSLTIKKPNELSRVLGLKTLATHGLAAVNSVPWDTIANYAKPFLNKVVSTTTNIVTRCLN

EMELTPVVQTIEVNSFSGYLKLTDNVYIKNADIVEEAKKVKPTVVVNAANVYLKHGGGVAGALNKATNNAM
EMELTPVVQTIEVNSFSGYLKLTDNVYIKNADIVEEAKKVKPTVVVNAANVYLKHGGGVAGALNKATNNAM

LLKYNENGTITDAVDCALDPLSETKCTLKSFTVEKGIYQTSNFRVQPTESIVRFPNITNLCPFGEVFNATRFASVY

QKEKVNINIVGDFKLNEEIAIILASFSASTSAFVETVKGLDYKAFKQIVESCGNFKVTKGKAKKGAWNIGEQKSIL

ITDAVDCALDPLSETKCTLKSFTVEKGIYQTSNFRVQPTESIVRFPNITNLCPFGEVFNATRFASVYAWNRKRISN

FSAVGNICYTPSKLIEYTDFATSACVLAAECTIFKDASGKPVPYCYDTNVLEGSVAYESLRPDTRYVLMDGSIIQF

AVTAYNGYLTSSSKTPEEHFIETISLAGSYKDWSYSGQSTQLGIEFLKRGDKSVYYTSNPTTFHLDGEVITFDNLK

FFASFYYVWKSYVHVVDGCNSSTCMMCYKRNRATRVECTTIVNGVRRSFYVYANGGKGFCKLHNWNCVN

LTLGVYDYLVSTQEFRYMNSQGLLPPKNSIDAFKLNIKLLGVGGKPCIKVATVQSKMSDVKCTSVVLLSVLQQL

KLQNNELSPVALRQMSCAAGTTQTACTDDNALAYYNTTKGGRFVLALLSDLQDLKWARFPKSDGTGTIYTEL

KLQNNELSPVALRQMSCAAGTTQTACTDDNALAYYNTTKGGRFVLALLSDLQDLKWARFPKSDGTGTIYTEL

QKEKVNINIVGDFKLNEEIAIILASFSASTSAFVETVKGLDYKAFKQIVESCGNFKVTKGKAKKGAWNIGEQKSIL

RETMSYLFQHANLDSCKRVLNVVCKTCGQQQTTLKGVEAVMYMGTLSYEQFKKGVQIPCTCGKQATKYLVQ
EVGHTDLMAAYVDNSSLTIKKPNELSRVLGLKTLATHGLAAVNSVPWDTIANYAKPFLNKVVSTTTNIVTRCLN

QKEKVNINIVGDFKLNEEIAIILASFSASTSAFVETVKGLDYKAFKQIVESCGNFKVTKGKAKKGAWNIGEQKSIL

EMELTPVVQTIEVNSFSGYLKLTDNVYIKNADIVEEAKKVKPTVVVNAANVYLKHGGGVAGALNKATNNAM

GGVAGALNKATNNAMQVESDDYIATNGPLKVGGSCVLSGHNLAKHCLHVVGPNVNKGEDIQLLKSAYENFN

LVAMKYNYEPLTQDHVDILGPLSAQTGIAVLDMCASLKELLQNGMNGRTILGSALLEDEFTPFDVVRQCSGV

KTIQPRVEKKKLDGFMGRIRSVYPVASPNECNQMCLSTLMKCDHCGETSWQTGDFVKATCEFCGTENLTKE

CDTFCAGSTFISDEVARDLSLQFKRPINPTDQSSYIVDSVTVKNGSIHLYFDKAGQKTYERHSLSHFVNLDNLRA
CDTFCAGSTFISDEVARDLSLQFKRPINPTDQSSYIVDSVTVKNGSIHLYFDKAGQKTYERHSLSHFVNLDNLRA
LPINVIVFDGKSKCEESSAKSASVYYSQLMCQPILLLDQALVSDVGDSAEVAVKMFDAYVNTFSSTFNVPMEK

KTIQPRVEKKKLDGFMGRIRSVYPVASPNECNQMCLSTLMKCDHCGETSWQTGDFVKATCEFCGTENLTKE[illegible]
ITDAVDCALDPLSETKCTLKSFTVEKGIYQTSNFRVQPTESIVRFPNITNLCPFGEVFNATRFASVYAWNRKRISN

GGVAGALNKATNNAMQVESDDYIATNGPLKVGGSCVLSGHNLAKHCLHVVGPNVNKGEDIQLLKSAYENFN

FSAVGNICYTPSKLIEYTDFATSACVLAAECTIFKDASGKPVPYCYDTNVLEGSVAYESLRPDTRYVLMDGSIIQF

LKTLVATAEAELAKNVSLDNVLSTFISAARQGFVDSDVETKDVVECLKLSHQSDIEVTGDSCNNYMLTYNKVEI

CDTFCAGSTFISDEVARDLSLQFKRPINPTDQSSYIVDSVTVKNGSIHLYFDKAGQKTYERHSLSHFVNLDNLRAI

MGIIAMSAFAMMFVKHKHAFLCLFLLPSLATVAYFNMVYMPASWVMRIMTWLDMVDTSLSGFKLKDCVMY

SPLYAFASEAARVVRSIFSRTLETAQNSVRVLQKAAITILDGISQYSLRLIDAMMFTSDLATNNLVVMAYITGGV

IRVCTNYMPYFFTLLLQLCTFTRSTNSRIKASMPTTIAKNTVKSVGKFCLEASFNYLKSPNFSKLINIIIWFLLLSVC

QVESDDYIATNGPLKVGGSCVLSGHNLAKHCLHVVGPNVNKGEDIQLLKSAYENFNQHEVLLAPLLSAGIFGAI

QVESDDYIATNGPLKVGGSCVLSGHNLAKHCLHVVGPNVNKGEDIQLLKSAYENFNQHEVLLAPLLSAGIFGAI

AWNRKRISNCVADYSVLYNSASFSTFKCYGVSPTKLNDLCFTNVYADSFVIRGDEVRQIAPGQTGKIADYNYKI

SPLYAFASEAARVVRSIFSRTLETAQNSVRVLQKAAITILDGISQYSLRLIDAMMFTSDLATNNLVVMAYITGGV

NCVADYSVLYNSASFSTFKCYGVSPTKLNDLCFTNVYADSFVIRGDEVRQIAPGQTGKIADYNYKLPDDFTGCV

PNTYLEGSVRVVTTFDSEYCRHGTCERSEAGVCVSTSGRWVLNNDYYRSLPGVFCGVDAVNLLTNMFTPLIQ

TLLSLREVRTIKVFTTVDNINLHTQVVDMSMTYGQQFGPTYLDGADVTKIKPHNSHEGKTFYVLPNDDTLRV

DTFCAGSTFISDEVARDLSLQFKRPINPTDQSSYIVDSVTVKNGSIHLYFDKAGQKTYERHSLSHFVNLDNLRA

RVESSSKLWAQCVQLHNDILLAKDTTEAFEKMVSLLSVLLSMQGAVDINKLCEEMLDNRATLQAIASEFSSLP

EPPCRFVTDTPKGPKVKYLYFIKGLNNLNRGMVLGSLAATVRLQAGNATEVPANSTVLSFCAFAVDAAKAYK

EPPCRFVTDTPKGPKVKYLYFIKGLNNLNRGMVLGSLAATVRLQAGNATEVPANSTVLSFCAFAVDAAKAYK

SPLYAFASEAARVVRSIFSRTLETAQNSVRVLQKAAITILDGISQYSLRLIDAMMFTSDLATNNLVVMAYITGG

QESPFVMMSAPPAQYELKHGTFTCASEYTGNYQCGHYKHITSKETLYCIDGALLTKSSEYKGPITDVFYKENSY
IRVCTNYMPYFFTLLLQLCTFTRSTNSRIKASMPTTIAKNTVKSVGKFCLEASFNYLKSPNFSKLINIIIWFLLLSVC

SPLYAFASEAARVVRSIFSRTLETAQNSVRVLQKAAITILDGISQYSLRLIDAMMFTSDLATNNLVVMAYITGG

VESDDYIATNGPLKVGGSCVLSGHNLAKHCLHVVGPNVNKGEDIQLLKSAYENFNQHEVLLAPLLSAGIFGA

NQHEVLLAPLLSAGIFGADPIHSLRVCVDTVRTNVYLAVFDKNLYDKLVSSFLEMKSEKQVEQKIAEIPKEEVKP

TFQSAVKRTIKGTHHWLLLTILTSLLVLVQSTQWSLFFFLYENAFLPFAMGIIAMSAFAMMFVKHKHAFLCLFL

GATTCGYLPQNAVVKIYCPACHNSEVGPEHSLAEYHNESGLKTILRKGGRTIAFGGCVFSYVGCHNKCAYWV

NNTKGSLPINVIVFDGKSKCEESSAKSASVYYSQLMCQPILLLDQALVSDVGDSAEVAVKMFDAYVNTFSSTF
NNTKGSLPINVIVFDGKSKCEESSAKSASVYYSQLMCQPILLLDQALVSDVGDSAEVAVKMFDAYVNTFSSTF
LKTLVATAEAELAKNVSLDNVLSTFISAARQGFVDSDVETKDVVECLKLSHQSDIEVTGDSCNNYMLTYNKVE

GATTCGYLPQNAVVKIYCPACHNSEVGPEHSLAEYHNESGLKTILRKGGRTIAFGGCVFSYVGCHNKCAYWV

NCVADYSVLYNSASFSTFKCYGVSPTKLNDLCFTNVYADSFVIRGDEVRQIAPGQTGKIADYNYKLPDDFTGCV

NQHEVLLAPLLSAGIFGADPIHSLRVCVDTVRTNVYLAVFDKNLYDKLVSSFLEMKSEKQVEQKIAEIPKEEVKP

PNTYLEGSVRVVTTFDSEYCRHGTCERSEAGVCVSTSGRWVLNNDYYRSLPGVFCGVDAVNLLTNMFTPLIQ

NMTPRDLGACIDCSARHINAQVAKSHNIALIWNVKDFMSLSEQLRKQIRSAAKKNNLPFKLTCATTRQVVNV

NNTKGSLPINVIVFDGKSKCEESSAKSASVYYSQLMCQPILLLDQALVSDVGDSAEVAVKMFDAYVNTFSSTF

YASAVVLLILMTARTVYDDGARRVWTLMNVLTLVYKVYYGNALDQAISMWALIISVTSNYSGVVTTVMFLAR

VQLTSQWLTNIFGTVYEKLKPVLDWLEEKFKEGVEFLRDGWEIVKFISTCACEIVGGQIVTCAKEIKESVQTFF

LGSLIYSTAALGVLMSNLGMPSYCTGYREGYLNSTNVTIATYCTGSIPCSVCLSGLDSLDTYPSLETIQITISSFKW

DPIHSLRVCVDTVRTNVYLAVFDKNLYDKLVSSFLEMKSEKQVEQKIAEIPKEEVKPFITESKPSVEQRKQDDKK
DPIHSLRVCVDTVRTNVYLAVFDKNLYDKLVSSFLEMKSEKQVEQKIAEIPKEEVKPFITESKPSVEQRKQDDKK

LPDDFTGCVIAWNSNNLDSKVGGNYNYLYRLFRKSNLKPFERDISTEIYQAGSTPCNGVEGFNCYFPLQSYGF

VQLTSQWLTNIFGTVYEKLKPVLDWLEEKFKEGVEFLRDGWEIVKFISTCACEIVGGQIVTCAKEIKESVQTFF

IAWNSNNLDSKVGGNYNYLYRLFRKSNLKPFERDISTEIYQAGSTPCNGVEGFNCYFPLQSYGFQPTNGVGY

PIGALDISASIVAGGIVAIVVTCLAYYFMRFRRAFGEYSHVVAFNTLLFLMSFTVLCLTPVYSFLPGVYSVIYLYLTI

EAFEYYHTTDPSFLGRYMSALNHTKKWKYPQVNGLTSIKWADNNCYLATALLTLQQIELKFNPPALQDAYYRA

NNTKGSLPINVIVFDGKSKCEESSAKSASVYYSQLMCQPILLLDQALVSDVGDSAEVAVKMFDAYVNTFSSTFN

SYAAFATAQEAYEQAVANGDSEVVLKKLKKSLNVAKSEFDRDAAMQRKLEKMADQAMTQMYKQARSEDK

DYLASGGQPITNCVKMLCTHTGTGQAITVTPEANMDQESFGGASCCLYCRCHIDHPNPKGFCDLKGKYVQIP

DYLASGGQPITNCVKMLCTHTGTGQAITVTPEANMDQESFGGASCCLYCRCHIDHPNPKGFCDLKGKYVQIP

/VQLTSQWLTNIFGTVYEKLKPVLDWLEEKFKEGVEFLRDGWEIVKFISTCACEIVGGQIVTCAKEIKESVQTFFI

TTTIKPVTYKLDGVVCTEIDPKLDNYYKKDNSYFTEQPIDLVPNQPYPNASFDNFKFVCDNIKFADDLNQLTGYI
LGSLIYSTAALGVLMSNLGMPSYCTGYREGYLNSTNVTIATYCTGSIPCSVCLSGLDSLDTYPSLETIQITISSFKW

/VQLTSQWLTNIFGTVYEKLKPVLDWLEEKFKEGVEFLRDGWEIVKFISTCACEIVGGQIVTCAKEIKESVQTFFI

DPIHSLRVCVDTVRTNVYLAVFDKNLYDKLVSSFLEMKSEKQVEQKIAEIPKEEVKPFITESKPSVEQRKQDDKK

FITESKPSVEQRKQDDKKIKACVEEVTTTLEETKFLTENLLLYIDINGNLHPDSATLVSDIDITFLKKDAPYIVGDV\

LPSLATVAYFNMVYMPASWVMRIMTWLDMVDTSLSGFKLKDCVMYASAVVLLILMTARTVYDDGARRVW

PRASANIGCNHTGVVGEGSEGLNDNLLEILQKEKVNINIVGDFKLNEEIAIILASFSASTSAFVETVKGLDYKAFK(

NVPMEKLKTLVATAEAELAKNVSLDNVLSTFISAARQGFVDSDVETKDVVECLKLSHQSDIEVTGDSCNNYML
NVPMEKLKTLVATAEAELAKNVSLDNVLSTFISAARQGFVDSDVETKDVVECLKLSHQSDIEVTGDSCNNYML
NMTPRDLGACIDCSARHINAQVAKSHNIALIWNVKDFMSLSEQLRKQIRSAAKKNNLPFKLTCATTRQVVNV\

RASANIGCNHTGVVGEGSEGLNDNLLEILQKEKVNINIVGDFKLNEEIAIILASFSASTSAFVETVKGLDYKAFK
IAWNSNNLDSKVGGNYNYLYRLFRKSNLKPFERDISTEIYQAGSTPCNGVEGFNCYFPLQSYGFQPTNGVGY

FITESKPSVEQRKQDDKKIKACVEEVTTTLEETKFLTENLLLYIDINGNLHPDSATLVSDIDITFLKKDAPYIVGDV

PIGALDISASIVAGGIVAIVVTCLAYYFMRFRRAFGEYSHVVAFNTLLFLMSFTVLCLTPVYSFLPGVYSVIYLYLT

VTTKIALKGGKIVNNWLKQLIKVTLVFLFVAAIFYLITPVHVMSKHTDFSSEIIGYKAIDGGVTRDIASTDTCFAN

NVPMEKLKTLVATAEAELAKNVSLDNVLSTFISAARQGFVDSDVETKDVVECLKLSHQSDIEVTGDSCNNYML

GIVFMCVEYCPIFFITGNTLQCIMLVYCFLGYFCTCYFGLFCLLNRYFRLTLGVYDYLVSTQEFRYMNSQGLLPP

KLVNKFLALCADSIIIGGAKLKALNLGETFVTHSKGLYRKCVKSREETGLLMPLKAPKEIIFLEGETLPTEVLTEEV

VDLTAFGLVAEWFLAYILFTRFFYVLGLAAIMQLFFSYFAVHFISNSWLMWLIINLVQMAPISAMVRMYIFFAS

IKACVEEVTTTLEETKFLTENLLLYIDINGNLHPDSATLVSDIDITFLKKDAPYIVGDVVQEGVLTAVVIPTKKAG

IKACVEEVTTTLEETKFLTENLLLYIDINGNLHPDSATLVSDIDITFLKKDAPYIVGDVVQEGVLTAVVIPTKKAG

QPTNGVGYQPYRVVVLSFELLHAPATVCGPKKSTNLVKNKCVNFNFNGLTGTGVLTESNKKFLPFQQFGRDIA

KLVNKFLALCADSIIIGGAKLKALNLGETFVTHSKGLYRKCVKSREETGLLMPLKAPKEIIFLEGETLPTEVLTEEV

QPYRVVVLSFELLHAPATVCGPKKSTNLVKNKCVNFNFNGLTGTGVLTESNKKFLPFQQFGRDIADTTDAVRD

FYLTNDVSFLAHIQWMVMFTPLVPFWITIAYIICISTKHFYWFFSNYLKRRVVFNGVSFSTFEEAALCTFLLNKE

RAGEAANFCALILAYCNKTVGELGDVRETMSYLFQHANLDSCKRVLNVVCKTCGQQQTTLKGVEAVMYM

VPMEKLKTLVATAEAELAKNVSLDNVLSTFISAARQGFVDSDVETKDVVECLKLSHQSDIEVTGDSCNNYML

RAKVTSAMQTMLFTMLRKLDNDALNNIINNARDGCVPLNIIPLTTAAKLMVVIPDYNTYKNTCDGTTFTYAS
TTCANDPVGFTLKNTVCTVCGMWKGYGCSCDQLREPMLQSADAQSFLNGFAV*
TTCANDPVGFTLKNTVCTVCGMWKGYGCSCDQLREPMLQSADAQSFLNGFAV*

LVNKFLALCADSIIIGGAKLKALNLGETFVTHSKGLYRKCVKSREETGLLMPLKAPKEIIFLEGETLPTEVLTEEV

KKPASRELKVTFFPDLNGDVVAIDYKHYTPSFKKGAKLLHKPIVWHVNNATNKATYKPNTWCIRCLWSTKPVE
/DLTAFGLVAEWFLAYILFTRFFYVLGLAAIMQLFFSYFAVHFISNSWLMWLIINLVQMAPISAMVRMYIFFASI

KLVNKFLALCADSIIIGGAKLKALNLGETFVTHSKGLYRKCVKSREETGLLMPLKAPKEIIFLEGETLPTEVLTEEVV

IKACVEEVTTTLEETKFLTENLLLYIDINGNLHPDSATLVSDIDITFLKKDAPYIVGDVVQEGVLTAVVIPTKKAGG

/QEGVLTAVVIPTKKAGGTTEMLAKALRKVPTDNYITTYPGQGLNGYTVEEAKTVLKKCKSAFYILPSIISNEKQE

/TLMNVLTLVYKVYYGNALDQAISMWALIISVTSNYSGVVTTVMFLARGIVFMCVEYCPIFFITGNTLQCIMLV

QIVESCGNFKVTKGKAKKGAWNIGEQKSILSPLYAFASEAARVVRSIFSRTLETAQNSVRVLQKAAITILDGISQY

TYNKVENMTPRDLGACIDCSARHINAQVAKSHNIALIWNVKDFMSLSEQLRKQIRSAAKKNNLPFKLTCATTF
TYNKVENMTPRDLGACIDCSARHINAQVAKSHNIALIWNVKDFMSLSEQLRKQIRSAAKKNNLPFKLTCATTF
VTTKIALKGGKIVNNWLKQLIKVTLVFLFVAAIFYLITPVHVMSKHTDFSSEIIGYKAIDGGVTRDIASTDTCFANI

QIVESCGNFKVTKGKAKKGAWNIGEQKSILSPLYAFASEAARVVRSIFSRTLETAQNSVRVLQKAAITILDGISQY
QPYRVVVLSFELLHAPATVCGPKKSTNLVKNKCVNFNFNGLTGTGVLTESNKKFLPFQQFGRDIADTTDAVRD[illegible]

VQEGVLTAVVIPTKKAGGTTEMLAKALRKVPTDNYITTYPGQGLNGYTVEEAKTVLKKCKSAFYILPSIISNEKQ[illegible]

FYLTNDVSFLAHIQWMVMFTPLVPFWITIAYIICISTKHFYWFFSNYLKRRVVFNGVSFSTFEEAALCTFLLNKE[illegible]

KHADFDTWFSQRGGSYTNDKACPLIAAVITREVGFVVPGLPGTILRTTNGDFLHFLPRVFSAVGNICYTPSKLIE

TYNKVENMTPRDLGACIDCSARHINAQVAKSHNIALIWNVKDFMSLSEQLRKQIRSAAKKNNLPFKLTCATTR

KNSIDAFKLNIKLLGVGGKPCIKVATVQSKMSDVKCTSVVLLSVLQQLRVESSSKLWAQCVQLHNDILLAKDTT

VLKTGDLQPLEQPTSEAVEAPLVGTPVCINGLMLLEIKDTEKYCALAPNMMVTNNTFTLKGGAPTKVTFGDDT

FYYVWKSYVHVVDGCNSSTCMMCYKRNRATRVECTTIVNGVRRSFYVYANGGKGFCKLHNWNCVNCDTFC

GTTEMLAKALRKVPTDNYITTYPGQGLNGYTVEEAKTVLKKCKSAFYILPSIISNEKQEILGTVSWNLREMLAHA
GTTEMLAKALRKVPTDNYITTYPGQGLNGYTVEEAKTVLKKCKSAFYILPSIISNEKQEILGTVSWNLREMLAHA

LDTTDAVRDPQTLEILDITPCSFGGVSVITPGTNTSNQVAVLYQDVNCTEVPVAIHADQLTPTWRVYSTGSNVF

VLKTGDLQPLEQPTSEAVEAPLVGTPVCINGLMLLEIKDTEKYCALAPNMMVTNNTFTLKGGAPTKVTFGDDT

PQTLEILDITPCSFGGVSVITPGTNTSNQVAVLYQDVNCTEVPVAIHADQLTPTWRVYSTGSNVFQTRAGCLIG

MYLKLRSDVLLPLTQYNRYLALYNKYKYFSGAMDTTSYREAACCHLAKALNDFSNSGSDVLYQPPQTSITSAVL

GTLSYEQFKKGVQIPCTCGKQATKYLVQQESPFVMMSAPPAQYELKHGTFTCASEYTGNYQCGHYKHITSKET

TYNKVENMTPRDLGACIDCSARHINAQVAKSHNIALIWNVKDFMSLSEQLRKQIRSAAKKNNLPFKLTCATTR

ALWEIQQVVDADSKIVQLSEISMDNSPNLAWPLIVTALRANSAVKLQNNELSPVALRQMSCAAGTTQTACTD

LKTGDLQPLEQPTSEAVEAPLVGTPVCINGLMLLEIKDTEKYCALAPNMMVTNNTFTLKGGAPTKVTFGDDT

TSNSFDVLKSEDAQGMDNLACEDLKPVSEEVVENPTIQKDVLECNVKTTEVVGDIILKPANNSLKITEEVGHT
FYYVWKSYVHVVDGCNSSTCMMCYKRNRATRVECTTIVNGVRRSFYVYANGGKGFCKLHNWNCVNCDTF

LKTGDLQPLEQPTSEAVEAPLVGTPVCINGLMLLEIKDTEKYCALAPNMMVTNNTFTLKGGAPTKVTFGDDT

TTEMLAKALRKVPTDNYITTYPGQGLNGYTVEEAKTVLKKCKSAFYILPSIISNEKQEILGTVSWNLREMLAHA

EILGTVSWNLREMLAHAEETRKLMPVCVETKAIVSTIQRKYKGIKIQEGVVDYGARFYFYTSKTTVASLINTLND

YCFLGYFCTCYFGLFCLLNRYFRLTLGVYDYLVSTQEFRYMNSQGLLPPKNSIDAFKLNIKLLGVGGKPCIKVAT

SLRLIDAMMFTSDLATNNLVVMAYITGGVVQLTSQWLTNIFGTVYEKLKPVLDWLEEKFKEGVEFLRDGWE

QVVNVVTTKIALKGGKIVNNWLKQLIKVTLVFLFVAAIFYLITPVHVMSKHTDFSSEIIGYKAIDGGVTRDIAST
QVVNVVTTKIALKGGKIVNNWLKQLIKVTLVFLFVAAIFYLITPVHVMSKHTDFSSEIIGYKAIDGGVTRDIAST
HADFDTWFSQRGGSYTNDKACPLIAAVITREVGFVVPGLPGTILRTTNGDFLHFLPRVFSAVGNICYTPSKLIE

YSLRLIDAMMFTSDLATNNLVVMAYITGGVVQLTSQWLTNIFGTVYEKLKPVLDWLEEKFKEGVEFLRDGWE
PQTLEILDITPCSFGGVSVITPGTNTSNQVAVLYQDVNCTEVPVAIHADQLTPTWRVYSTGSNVFQTRAGCLIG

EILGTVSWNLREMLAHAEETRKLMPVCVETKAIVSTIQRKYKGIKIQEGVVDYGARFYFYTSKTTVASLINTLND

MYLKLRSDVLLPLTQYNRYLALYNKYKYFSGAMDTTSYREAACCHLAKALNDFSNSGSDVLYQPPQTSITSAVL

YTDFATSACVLAAECTIFKDASGKPVPYCYDTNVLEGSVAYESLRPDTRYVLMDGSIIQFPNTYLEGSVRVVTT

RQVVNVVTTKIALKGGKIVNNWLKQLIKVTLVFLFVAAIFYLITPVHVMSKHTDFSSEIIGYKAIDGGVTRDIAST

EAFEKMVSLLSVLLSMQGAVDINKLCEEMLDNRATLQAIASEFSSLPSYAAFATAQEAYEQAVANGDSEVVLK

VIEVQGYKSVNITFELDERIDKVLNEKCSAYTVELGTEVNEFACVVADAVIKTLQPVSELLTPLGIDLDEWSMAT

CAGSTFISDEVARDLSLQFKRPINPTDQSSYIVDSVTVKNGSIHLYFDKAGQKTYERHSLSHFVNLDNLRANNTK

EETRKLMPVCVETKAIVSTIQRKYKGIKIQEGVVDYGARFYFYTSKTTVASLINTLNDLNETLVTMPLGYVTHGL
EETRKLMPVCVETKAIVSTIQRKYKGIKIQEGVVDYGARFYFYTSKTTVASLINTLNDLNETLVTMPLGYVTHGL

FQTRAGCLIGAEHVNNSYECDIPIGAGICASYQTQTNSPRRARSVASQSIIAYTMSLGAENSVAYSNNSIAIPTN

VIEVQGYKSVNITFELDERIDKVLNEKCSAYTVELGTEVNEFACVVADAVIKTLQPVSELLTPLGIDLDEWSMAT

GAEHVNNSYECDIPIGAGICASYQTQTNSPRRARSVASQSIIAYTMSLGAENSVAYSNNSIAIPTNFTISVTTEILP

QSGFRKMAFPSGKVEGCMVQVTCGTTTLNGLWLDDVVYCPRHVICTSEDMLNPNYEDLLIRKSNHNFLVQ

LYCIDGALLTKSSEYKGPITDVFYKENSYTTTIKPVTYKLDGVVCTEIDPKLDNYYKKDNSYFTEQPIDLVPNQPY

QVVNVVTTKIALKGGKIVNNWLKQLIKVTLVFLFVAAIFYLITPVHVMSKHTDFSSEIIGYKAIDGGVTRDIAST

DNALAYYNTTKGGRFVLALLSDLQDLKWARFPKSDGTGTIYTELEPPCRFVTDTPKGPKVKYLYFIKGLNNLN

VIEVQGYKSVNITFELDERIDKVLNEKCSAYTVELGTEVNEFACVVADAVIKTLQPVSELLTPLGIDLDEWSMA

DLMAAYVDNSSLTIKKPNELSRVLGLKTLATHGLAAVNSVPWDTIANYAKPFLNKVVSTTTNIVTRCLNRVCTN
CAGSTFISDEVARDLSLQFKRPINPTDQSSYIVDSVTVKNGSIHLYFDKAGQKTYERHSLSHFVNLDNLRANNTK

VIEVQGYKSVNITFELDERIDKVLNEKCSAYTVELGTEVNEFACVVADAVIKTLQPVSELLTPLGIDLDEWSMAT

EETRKLMPVCVETKAIVSTIQRKYKGIKIQEGVVDYGARFYFYTSKTTVASLINTLNDLNETLVTMPLGYVTHGL

LNETLVTMPLGYVTHGLNLEEAARYMRSLKVPATVSVSSPDAVTAYNGYLTSSSKTPEEHFIETISLAGSYKDW

VQSKMSDVKCTSVVLLSVLQQLRVESSSKLWAQCVQLHNDILLAKDTTEAFEKMVSLLSVLLSMQGAVDINKL

IVKFISTCACEIVGGQIVTCAKEIKESVQTFFKLVNKFLALCADSIIIGGAKLKALNLGETFVTHSKGLYRKCVKSRE

DTCFANKHADFDTWFSQRGGSYTNDKACPLIAAVITREVGFVVPGLPGTILRTTNGDFLHFLPRVFSAVGNICY
DTCFANKHADFDTWFSQRGGSYTNDKACPLIAAVITREVGFVVPGLPGTILRTTNGDFLHFLPRVFSAVGNICY
YTDFATSACVLAAECTIFKDASGKPVPYCYDTNVLEGSVAYESLRPDTRYVLMDGSIIQFPNTYLEGSVRVVTTF

IVKFISTCACEIVGGQIVTCAKEIKESVQTFFKLVNKFLALCADSIIIGGAKLKALNLGETFVTHSKGLYRKCVKSRE
GAEHVNNSYECDIPIGAGICASYQTQTNSPRRARSVASQSIIAYTMSLGAENSVAYSNNSIAIPTNFTISVTTEILP

LNETLVTMPLGYVTHGLNLEEAARYMRSLKVPATVSVSSPDAVTAYNGYLTSSSKTPEEHFIETISLAGSYKDW

QSGFRKMAFPSGKVEGCMVQVTCGTTTLNGLWLDDVVYCPRHVICTSEDMLNPNYEDLLIRKSNHNFLVQ

FDSEYCRHGTCERSEAGVCVSTSGRWVLNNDYYRSLPGVFCGVDAVNLLTNMFTPLIQPIGALDISASIVAGGI

DTCFANKHADFDTWFSQRGGSYTNDKACPLIAAVITREVGFVVPGLPGTILRTTNGDFLHFLPRVFSAVGNICY

KLKKSLNVAKSEFDRDAAMQRKLEKMADQAMTQMYKQARSEDKRAKVTSAMQTMLFTMLRKLDNDALN

TYYLFDESGEFKLASHMYCSFYPPDEDEEEGDCEEEEFEPSTQYEYGTEDDYQGKPLEFGATSAALQPEEEQEEI

KGSLPINVIVFDGKSKCEESSAKSASVYYSQLMCQPILLLDQALVSDVGDSAEVAVKMFDAYVNTFSSTFNVPM

NLEEAARYMRSLKVPATVSVSSPDAVTAYNGYLTSSSKTPEEHFIETISLAGSYKDWSYSGQSTQLGIEFLKRGD
NLEEAARYMRSLKVPATVSVSSPDAVTAYNGYLTSSSKTPEEHFIETISLAGSYKDWSYSGQSTQLGIEFLKRGD

FTISVTTEILPVSMTKTSVDCTMYICGDSTECSNLLLQYGSFCTQLNRALTGIAVEQDKNTQEVFAQVKQIYKTP

TYYLFDESGEFKLASHMYCSFYPPDEDEEEGDCEEEEFEPSTQYEYGTEDDYQGKPLEFGATSAALQPEEEQEEI

PVSMTKTSVDCTMYICGDSTECSNLLLQYGSFCTQLNRALTGIAVEQDKNTQEVFAQVKQIYKTPPIKDFGGFN

AGNVQLRVIGHSMQNCVLKLKVDTANPKTPKYKFVRIQPGQTFSVLACYNGSPSGVYQCAMRPNFTIKGSFL

PNASFDNFKFVCDNIKFADDLNQLTGYKKPASRELKVTFFPDLNGDVVAIDYKHYTPSFKKGAKLLHKPIVWH

TCFANKHADFDTWFSQRGGSYTNDKACPLIAAVITREVGFVVPGLPGTILRTTNGDFLHFLPRVFSAVGNIC

RGMVLGSLAATVRLQAGNATEVPANSTVLSFCAFAVDAAKAYKDYLASGGQPITNCVKMLCTHTGTGQAIT

TYYLFDESGEFKLASHMYCSFYPPDEDEEEGDCEEEEFEPSTQYEYGTEDDYQGKPLEFGATSAALQPEEEQEE

NYMPYFFTLLLQLCTFTRSTNSRIKASMPTTIAKNTVKSVGKFCLEASFNYLKSPNFSKLINIIIWFLLLSVCLGSLIY
GSLPINVIVFDGKSKCEESSAKSASVYYSQLMCQPILLLDQALVSDVGDSAEVAVKMFDAYVNTFSSTFNVPM

TYYLFDESGEFKLASHMYCSFYPPDEDEEEGDCEEEEFEPSTQYEYGTEDDYQGKPLEFGATSAALQPEEEQEE

NLEEAARYMRSLKVPATVSVSSPDAVTAYNGYLTSSSKTPEEHFIETISLAGSYKDWSYSGQSTQLGIEFLKRGD

SYSGQSTQLGIEFLKRGDKSVYYTSNPTTFHLDGEVITFDNLKTLLSLREVRTIKVFTTVDNINLHTQVVDMSM

CEEMLDNRATLQAIASEFSSLPSYAAFATAQEAYEQAVANGDSEVVLKKLKKSLNVAKSEFDRDAAMQRKLE

ETGLLMPLKAPKEIIFLEGETLPTEVLTEEVVLKTGDLQPLEQPTSEAVEAPLVGTPVCINGLMLLEIKDTEKYCA

YTPSKLIEYTDFATSACVLAAECTIFKDASGKPVPYCYDTNVLEGSVAYESLRPDTRYVLMDGSIIQFPNTYLEGS
YTPSKLIEYTDFATSACVLAAECTIFKDASGKPVPYCYDTNVLEGSVAYESLRPDTRYVLMDGSIIQFPNTYLEGS
FDSEYCRHGTCERSEAGVCVSTSGRWVLNNDYYRSLPGVFCGVDAVNLLTNMFTPLIQPIGALDISASIVAGGI

ETGLLMPLKAPKEIIFLEGETLPTEVLTEEVVLKTGDLQPLEQPTSEAVEAPLVGTPVCINGLMLLEIKDTEKYCA
VSMTKTSVDCTMYICGDSTECSNLLLQYGSFCTQLNRALTGIAVEQDKNTQEVFAQVKQIYKTPPIKDFGGF

SYSGQSTQLGIEFLKRGDKSVYYTSNPTTFHLDGEVITFDNLKTLLSLREVRTIKVFTTVDNINLHTQVVDMSM

AGNVQLRVIGHSMQNCVLKLKVDTANPKTPKYKFVRIQPGQTFSVLACYNGSPSGVYQCAMRPNFTIKGSFL

VAIVVTCLAYYFMRFRRAFGEYSHVVAFNTLLFLMSFTVLCLTPVYSFLPGVYSVIYLYLTFYLTNDVSFLAHIQW

TPSKLIEYTDFATSACVLAAECTIFKDASGKPVPYCYDTNVLEGSVAYESLRPDTRYVLMDGSIIQFPNTYLEGS

NIINNARDGCVPLNIIPLTTAAKLMVVIPDYNTYKNTCDGTTFTYASALWEIQQVVDADSKIVQLSEISMDNS

DWLDDDSQQTVGQQDGSEDNQTTTIQTIVEVQPQLEMELTPVVQTIEVNSFSGYLKLTDNVYIKNADIVEEA

EKLKTLVATAEAELAKNVSLDNVLSTFISAARQGFVDSDVETKDVVECLKLSHQSDIEVTGDSCNNYMLTYNK

KSVYYTSNPTTFHLDGEVITFDNLKTLLSLREVRTIKVFTTVDNINLHTQVVDMSMTYGQQFGPTYLDGADVT

KSVYYTSNPTTFHLDGEVITFDNLKTLLSLREVRTIKVFTTVDNINLHTQVVDMSMTYGQQFGPTYLDGADVT

PIKDFGGFNFSQILPDPSKPSKRSFIEDLLFNKVTLADAGFIKQYGDCLGDIAARDLICAQKFNGLTVLPPLLTDE

DWLDDDSQQTVGQQDGSEDNQTTTIQTIVEVQPQLEMELTPVVQTIEVNSFSGYLKLTDNVYIKNADIVEEA

FSQILPDPSKPSKRSFIEDLLFNKVTLADAGFIKQYGDCLGDIAARDLICAQKFNGLTVLPPLLTDEMIAQYTSA

NGSCGSVGFNIDYDCVSFCYMHHMELPTGVHAGTDLEGNFYGPFVDRQTAQAAGTDTTITVNVLAWLYAA

VNNATNKATYKPNTWCIRCLWSTKPVETSNSFDVLKSEDAQGMDNLACEDLKPVSEEVVENPTIQKDVLECN

TPSKLIEYTDFATSACVLAAECTIFKDASGKPVPYCYDTNVLEGSVAYESLRPDTRYVLMDGSIIQFPNTYLEGS

VTPEANMDQESFGGASCCLYCRCHIDHPNPKGFCDLKGKYVQIPTTCANDPVGFTLKNTVCTVCGMWKGYG

DWLDDDSQQTVGQQDGSEDNQTTTIQTIVEVQPQLEMELTPVVQTIEVNSFSGYLKLTDNVYIKNADIVEEA

STAALGVLMSNLGMPSYCTGYREGYLNSTNVTIATYCTGSIPCSVCLSGLDSLDTYPSLETIQITISSFKWDLTA
EKLKTLVATAEAELAKNVSLDNVLSTFISAARQGFVDSDVETKDVVECLKLSHQSDIEVTGDSCNNYMLTYNK

DWLDDDSQQTVGQQDGSEDNQTTTIQTIVEVQPQLEMELTPVVQTIEVNSFSGYLKLTDNVYIKNADIVEEA

KSVYYTSNPTTFHLDGEVITFDNLKTLLSLREVRTIKVFTTVDNINLHTQVVDMSMTYGQQFGPTYLDGADVT

TYGQQFGPTYLDGADVTKIKPHNSHEGKTFYVLPNDDTLRVEAFEYYHTTDPSFLGRYMSALNHTKKWKYPQ

KMADQAMTQMYKQARSEDKRAKVTSAMQTMLFTMLRKLDNDALNNIINNARDGCVPLNIIPLTTAAKLM

LAPNMMVTNNTFTLKGGAPTKVTFGDDTVIEVQGYKSVNITFELDERIDKVLNEKCSAYTVELGTEVNEFAC

VRVVTTFDSEYCRHGTCERSEAGVCVSTSGRWVLNNDYYRSLPGVFCGVDAVNLLTNMFTPLIQPIGALDISA
VRVVTTFDSEYCRHGTCERSEAGVCVSTSGRWVLNNDYYRSLPGVFCGVDAVNLLTNMFTPLIQPIGALDISA
VAIVVTCLAYYFMRFRRAFGEYSHVVAFNTLLFLMSFTVLCLTPVYSFLPGVYSVIYLYLTFYLTNDVSFLAHIQ

LAPNMMVTNNTFTLKGGAPTKVTFGDDTVIEVQGYKSVNITFELDERIDKVLNEKCSAYTVELGTEVNEFACV
NFSQILPDPSKPSKRSFIEDLLFNKVTLADAGFIKQYGDCLGDIAARDLICAQKFNGLTVLPPLLTDEMIAQYTSAL

TYGQQFGPTYLDGADVTKIKPHNSHEGKTFYVLPNDDTLRVEAFEYYHTTDPSFLGRYMSALNHTKKWKYPQ

NGSCGSVGFNIDYDCVSFCYMHHMELPTGVHAGTDLEGNFYGPFVDRQTAQAAGTDTTITVNVLAWLYAA

MVMFTPLVPFWITIAYIICISTKHFYWFFSNYLKRRVVFNGVSFSTFEEAALCTFLLNKEMYLKLRSDVLLPLTQ

RVVTTFDSEYCRHGTCERSEAGVCVSTSGRWVLNNDYYRSLPGVFCGVDAVNLLTNMFTPLIQPIGALDISA

NLAWPLIVTALRANSAVKLQNNELSPVALRQMSCAAGTTQTACTDDNALAYYNTTKGGRFVLALLSDLQDL

KKVKPTVVVNAAANVYLKHGGGVAGALNKATNNAMQVESDDYIATNGPLKVGGSCVLSGHNLAKHCLHVV

VENMTPRDLGACIDCSARHINAQVAKSHNIALIWNVKDFMSLSEQLRKQIRSAAKKNNLPFKLTCATTRQVV

KIKPHNSHEGKTFYVLPNDDTLRVEAFEYYHTTDPSFLGRYMSALNHTKKWKYPQVNGLTSIKWADNNCYL
KIKPHNSHEGKTFYVLPNDDTLRVEAFEYYHTTDPSFLGRYMSALNHTKKWKYPQVNGLTSIKWADNNCYL

MIAQYTSALLAGTITSGWTFGAGAALQIPFAMQMAYRFNGIGVTQNVLYENQKLIANQFNSAIGKIQDSLSS

KKVKPTVVVNAAANVYLKHGGGVAGALNKATNNAMQVESDDYIATNGPLKVGGSCVLSGHNLAKHCLHVV

LLAGTITSGWTFGAGAALQIPFAMQMAYRFNGIGVTQNVLYENQKLIANQFNSAIGKIQDSLSSTASALGKLQ

VINGDRWFLNRFTTTLNDFNLVAMKYNYEPLTQDHVDILGPLSAQTGIAVLDMCASLKELLQNGMNGRTIL[illegible]

[illegible]NVKTTEVVGDIILKPANNSLKITEEVGHTDLMAAYVDNSSLTIKKPNELSRVLGLKTLATHGLAAVNSVPWDTI[illegible]

[illegible]VRVVTTFDSEYCRHGTCERSEAGVCVSTSGRWVLNNDYYRSLPGVFCGVDAVNLLTNMFTPLIQPIGALDISA

[illegible]GCSCDQLREPMLQSADAQSFLNGFAV*

KKVKPTVVVNAANVYLKHGGGVAGALNKATNNAMQVESDDYIATNGPLKVGGSCVLSGHNLAKHCLHVV[illegible]

FGLVAEWFLAYILFTRFFYVLGLAAIMQLFFSYFAVHFISNSWLMWLIINLVQMAPISAMVRMYIFFASFYYVW
VENMTPRDLGACIDCSARHINAQVAKSHNIALIWNVKDFMSLSEQLRKQIRSAAKKNNLPFKLTCATTRQVVI

KKVKPTVVVNAANVYLKHGGGVAGALNKATNNAMQVESDDYIATNGPLKVGGSCVLSGHNLAKHCLHVVC

TKIKPHNSHEGKTFYVLPNDDTLRVEAFEYYHTTDPSFLGRYMSALNHTKKWKYPQVNGLTSIKWADNNCYLA

VNGLTSIKWADNNCYLATALLTLQQIELKFNPPALQDAYYRARAGEAANFCALILAYCNKTVGELGDVRETMS

VVIPDYNTYKNTCDGTTFTYASALWEIQQVVDADSKIVQLSEISMDNSPNLAWPLIVTALRANSAVKLQNNEL

VVADAVIKTLQPVSELLTPLGIDLDEWSMATYYLFDESGEFKLASHMYCSFYPPDEDEEEGDCEEEEFEPSTQYE

ASIVAGGIVAIVVTCLAYYFMRFRRAFGEYSHVVAFNTLLFLMSFTVLCLTPVYSFLPGVYSVIYLYLTFYLTNDVSI
ASIVAGGIVAIVVTCLAYYFMRFRRAFGEYSHVVAFNTLLFLMSFTVLCLTPVYSFLPGVYSVIYLYLTFYLTNDVSI
VMVMFTPLVPFWITIAYIICISTKHFYWFFSNYLKRRVVFNGVSFSTFEEAALCTFLLNKEMYLKLRSDVLLPLTQ

VADAVIKTLQPVSELLTPLGIDLDEWSMATYYLFDESGEFKLASHMYCSFYPPDEDEEEGDCEEEEFEPSTQYE
LAGTITSGWTFGAGAALQIPFAMQMAYRFNGIGVTQNVLYENQKLIANQFNSAIGKIQDSLSSTASALGKLQ

VNGLTSIKWADNNCYLATALLTLQQIELKFNPPALQDAYYRARAGEAANFCALILAYCNKTVGELGDVRETM

VINGDRWFLNRFTTTLNDFNLVAMKYNYEPLTQDHVDILGPLSAQTGIAVLDMCASLKELLQNGMNGRTIL

YNRYLALYNKYKYFSGAMDTTSYREAACCHLAKALNDFSNSGSDVLYQPPQTSITSAVLQSGFRKMAFPSGK

SIVAGGIVAIVVTCLAYYFMRFRRAFGEYSHVVAFNTLLFLMSFTVLCLTPVYSFLPGVYSVIYLYLTFYLTND

KWARFPKSDGTGTIYTELEPPCRFVTDTPKGPKVKYLYFIKGLNNLNRGMVLGSLAATVRLQAGNATEVPAN

PNVNKGEDIQLLKSAYENFNQHEVLLAPLLSAGIFGADPIHSLRVCVDTVRTNVYLAVFDKNLYDKLVSSFLE

NVVTTKIALKGGKIVNNWLKQLIKVTLVFLFVAAIFYLITPVHVMSKHTDFSSEIIGYKAIDGGVTRDIASTDTCF

TALLTLQQIELKFNPPALQDAYYRARAGEAANFCALILAYCNKTVGELGDVRETMSYLFQHANLDSCKRVLN

TALLTLQQIELKFNPPALQDAYYRARAGEAANFCALILAYCNKTVGELGDVRETMSYLFQHANLDSCKRVLN

TASALGKLQDVVNQNAQALNTLVKQLSSNFGAISSVLNDILSRLDKVEAEVQIDRLITGRLQSLQTYVTQQLIR

PNVNKGEDIQLLKSAYENFNQHEVLLAPLLSAGIFGADPIHSLRVCVDTVRTNVYLAVFDKNLYDKLVSSFLE

DVVNQNAQALNTLVKQLSSNFGAISSVLNDILSRLDKVEAEVQIDRLITGRLQSLQTYVTQQLIRAAEIRASAN

GSALLEDEFTPFDVVRQCSGVTFQSAVKRTIKGTHHWLLLTILTSLLVLVQSTQWSLFFFLYENAFLPFAMGIIAM

ANYAKPFLNKVVSTTTNIVTRCLNRVCTNYMPYFFTLLLQLCTFTRSTNSRIKASMPTTIAKNTVKSVGKFCLEAS

SIVAGGIVAIVVTCLAYYFMRFRRAFGEYSHVVAFNTLLFLMSFTVLCLTPVYSFLPGVYSVIYLYLTFYLTND

GPNVNKGEDIQLLKSAYENFNQHEVLLAPLLSAGIFGADPIHSLRVCVDTVRTNVYLAVFDKNLYDKLVSSFLEM

KSYVHVVDGCNSSTCMMCYKRNRATRVECTTIVNGVRRSFYVYANGGKGFCKLHNWNCVNCDTFCAGST
NVVTTKIALKGGKIVNNWLKQLIKVTLVFLFVAAIFYLITPVHVMSKHTDFSSEIIGYKAIDGGVTRDIASTDTCF

PNVNKGEDIQLLKSAYENFNQHEVLLAPLLSAGIFGADPIHSLRVCVDTVRTNVYLAVFDKNLYDKLVSSFLE

TALLTLQQIELKFNPPALQDAYYRARAGEAANFCALILAYCNKTVGELGDVRETMSYLFQHANLDSCKRVLN

YLFQHANLDSCKRVLNVVCKTCGQQQTTLKGVEAVMYMGTLSYEQFKKGVQIPCTCGKQATKYLVQQESP

SPVALRQMSCAAGTTQTACTDDNALAYYNTTKGGRFVLALLSDLQDLKWARFPKSDGTGTIYTELEPPCRFV

YGTEDDYQGKPLEFGATSAALQPEEEQEEDWLDDDSQQTVGQQDGSEDNQTTTIQTIVEVQPQLEMELTP

FLAHIQWMVMFTPLVPFWITIAYIICISTKHFYWFFSNYLKRRVVFNGVSFSTFEEAALCTFLLNKEMYLK
FLAHIQWMVMFTPLVPFWITIAYIICISTKHFYWFFSNYLKRRVVFNGVSFSTFEEAALCTFLLNKEMYLK
YNRYLALYNKYKYFSGAMDTTSYREAACCHLAKALNDFSNSGSDVLYQPPQTSITSAVLQSGFRKMAFPSGK

YGTEDDYQGKPLEFGATSAALQPEEEQEEDWLDDDSQQTVGQQDGSEDNQTTTIQTIVEVQPQLEMELTP
DVVNQNAQALNTLVKQLSSNFGAISSVLNDILSRLDKVEAEVQIDRLITGRLQSLQTYVTQQLIRAAEIRASAN

YLFQHANLDSCKRVLNVVCKTCGQQQTTLKGVEAVMYMGTLSYEQFKKGVQIPCTCGKQATKYLVQQESP

SALLEDEFTPFDVVRQCSGVTFQSAVKRTIKGTHHWLLLTILTSLLVLVQSTQWSLFFFLYENAFLPFAMGIIA

VE

STVLSFCAFAVDAAKAYKDYLASGGQPIT

MKSEK

AN

VVCKT
VVCKT

A

MKSEK

L

MSAFAMMFV

SFN

MKSEK

[illegible]FISDEVAR
[illegible]AN

[illegible]MKSEK

[illegible]VCKT

[illegible]FVMMSAPPA

[illegible]TDTPKGPKVKYLYFIKGLNNLNRGMVLG

[illegible]VVQTIE

[illegible]VE

VVQTIE
L

FVMMSAPPA

MSAFAMMFV

 Click here to access/download;Table;Supplemental_Material_3.xlsx

	subgenome	ref_leader_e	peak_leader_	ref_TRS_start	peak_TRS_sta	peak_count
Sample_1	S	65	65	21552	21552	156(156,152,
	ORF3a	69	69	25385	25385	5(5,0,0,0)
	E	69	69	26237	26237	12(12,0,0,0)
	M	65	65	26469	26469	305(305,304,
	ORF6	69	69	27041	27041	136(131,135,
	ORF7a	69	0	27388	0	0
	ORF7b	65	0	27644	0	0
	ORF8	65	0	27884	0	0
	N	65	64	28256	28255	509(509,504,
	ORF10	65	0	29530	0	0
Sample_2	S	65	65	21552	21552	298(296,291,
	ORF3a	69	69	25385	25385	4(4,0,0,0)
	E	69	69	26237	26237	45(45,0,0,0)
	M	65	65	26469	26469	71(71,66,66,6
	ORF6	69	69	27041	27041	32(32,30,30,3
	ORF7a	69	69	27388	27388	1(1,0,0,0)
	ORF7b	65	0	27644	0	0
	ORF8	65	0	27884	0	0
	N	65	65	28256	28256	1996(1991,19
	ORF10	65	0	29530	0	0
Sample_3	S	65	65	21552	21552	1022(1007,96
	ORF3a	69	69	25385	25385	13(11,0,0,0)
	E	69	69	26237	26237	97(97,0,0,0)
	M	65	65	26469	26469	1384(1382,12
	ORF6	69	69	27041	27041	583(581,569,
	ORF7a	69	69	27388	27388	34(19,33,18,1
	ORF7b	65	0	27644	0	0
	ORF8	65	65	27884	27884	3(2,0,0,0)
	N	65	65	28256	28256	3462(3452,33
	ORF10	65	0	29530	0	0
Sample_4	S	65	0	21552	0	0
	ORF3a	69	0	25385	0	0
	E	69	0	26237	0	0
	M	65	65	26469	26469	276(276,276,
	ORF6	69	0	27041	0	0
	ORF7a	69	0	27388	0	0
	ORF7b	65	0	27644	0	0
	ORF8	65	0	27884	0	0
	N	65	0	28256	0	0
	ORF10	65	0	29530	0	0
	S	65	0	21552	0	0
	ORF3a	69	0	25385	0	0

	E	69	0	26237	0	0
	M	65	0	26469	0	0
Sample_5	ORF6	69	0	27041	0	0
	ORF7a	69	0	27388	0	0
	ORF7b	65	0	27644	0	0
	ORF8	65	0	27884	0	0
	N	65	64	28256	28255	433(432,431,
	ORF10	65	0	29530	0	0
	S	65	65	21552	21552	715(713,677,
	ORF3a	69	69	25385	25385	5(5,0,0,0)
	E	69	69	26237	26237	115(115,2,2,2
	M	65	65	26469	26469	1178(1176,11
Sample_6	ORF6	69	69	27041	27041	410(409,403,
	ORF7a	69	69	27388	27388	6(0,6,0,4)
	ORF7b	65	0	27644	0	0
	ORF8	65	0	27884	0	0
	N	65	65	28256	28256	1135(1126,11
	ORF10	65	0	29530	0	0
	S	65	65	21552	21552	324(324,313,
	ORF3a	69	69	25385	25385	7(7,0,0,0)
	E	69	69	26237	26237	87(87,2,2,2)
	M	65	65	26469	26469	639(639,629,
Sample_7	ORF6	69	69	27041	27041	486(485,471,
	ORF7a	69	0	27388	0	0
	ORF7b	65	0	27644	0	0
	ORF8	65	65	27884	27884	1(1,0,0,0)
	N	65	65	28256	28256	1847(1843,17
	ORF10	65	0	29530	0	0
	S	65	0	21552	0	0
	ORF3a	69	0	25385	0	0
	E	69	0	26237	0	0
	M	65	0	26469	0	0
Sample_8	ORF6	69	0	27041	0	0
	ORF7a	69	0	27388	0	0
	ORF7b	65	0	27644	0	0
	ORF8	65	0	27884	0	0
	N	65	65	28256	28256	2502(2498,24
	ORF10	65	0	29530	0	0
	S	65	65	21552	21552	112(111,112,
	ORF3a	69	69	25385	25385	4(4,0,0,0)
	E	69	69	26237	26237	171(170,0,0,(
	M	65	65	26469	26469	993(993,983,
Sample_9	ORF6	69	69	27041	27041	334(333,328,

Sample_9	ORF7a	69	69	27388	27388	8(1,7,0,2)
	ORF7b	65	0	27644	0	0
	ORF8	65	0	27884	0	0
	N	65	65	28256	28256	11960(11926
	ORF10	65	0	29530	0	0
	S	65	65	21552	21552	656(655,617,
	ORF3a	69	69	25385	25385	8(6,0,0,0)
	E	69	69	26237	26237	62(62,0,0,0)
	M	65	65	26469	26469	1549(1548,13
Sample_10	ORF6	69	69	27041	27041	429(429,407,
	ORF7a	69	69	27388	27388	5(3,4,2,2)
	ORF7b	65	0	27644	0	0
	ORF8	65	65	27884	27884	3(3,0,0,0)
	N	65	64	28256	28255	5681(5670,55
	ORF10	65	0	29530	0	0
	S	65	65	21552	21552	1283(1274,12
	ORF3a	69	69	25385	25385	3(3,0,0,0)
	E	69	69	26237	26237	274(273,2,2,4
	M	65	65	26469	26469	2213(2212,20
Sample_11	ORF6	69	69	27041	27041	925(924,904,
	ORF7a	69	69	27388	27388	30(3,29,2,2)
	ORF7b	65	0	27644	0	0
	ORF8	65	0	27884	0	0
	N	65	65	28256	28256	8846(8822,86
	ORF10	65	0	29530	0	0
	S	65	65	21552	21552	641(639,593,
	ORF3a	69	69	25385	25385	19(16,0,0,0)
	E	69	69	26237	26237	134(134,2,2,2
	M	65	65	26469	26469	1947(1943,18
Sample_12	ORF6	69	69	27041	27041	796(795,758,
	ORF7a	69	69	27388	27388	10(9,3,2,2)
	ORF7b	65	68	27644	27677	12(12,12,12,1
	ORF8	65	0	27884	0	0
	N	65	64	28256	28255	3861(3860,38
	ORF10	65	0	29530	0	0
	S	65	0	21552	0	0
	ORF3a	69	0	25385	0	0
	E	69	0	26237	0	0
	M	65	0	26469	0	0
Sample_13	ORF6	69	0	27041	0	0
	ORF7a	69	0	27388	0	0
	ORF7b	65	0	27644	0	0
	ORF8	65	0	27884	0	0

	N	65	65	28256	28256	1599(1594,15
	ORF10	65	0	29530	0	0
Sample_14	S	65	65	21552	21552	711(710,703,
	ORF3a	69	69	25385	25385	0(0,0,0,0)
	E	69	0	26237	0	0
	M	65	65	26469	26469	1647(1647,16
	ORF6	69	0	27041	0	0
	ORF7a	69	0	27388	0	0
	ORF7b	65	0	27644	0	0
	ORF8	65	0	27884	0	0
	N	65	65	28256	28256	1445(1440,14
	ORF10	65	0	29530	0	0
Sample_15	S	65	65	21552	21552	325(325,324,
	ORF3a	69	69	25385	25385	3(3,0,0,0)
	E	69	69	26237	26237	43(43,0,0,0)
	M	65	65	26469	26469	242(242,237,
	ORF6	69	0	27041	0	0
	ORF7a	69	0	27388	0	0
	ORF7b	65	0	27644	0	0
	ORF8	65	0	27884	0	0
	N	65	65	28256	28256	870(868,859,
	ORF10	65	0	29530	0	0

The numbers in the bracket are (reads with left primers, reads with right primers, reads with bot
Normalized count=(Read count/Total number of read mapped on reference genome)*1000000.
Total number of read mapped on reference genome is 1043447, 1313236, 3073041, 224581, 83
“ref_leader_end” and “peak_leader_end” point to the reference position of the end of leader a

peak_normal	cluster_count	cluster_normalized_count
149.50(149.5	158(158,154,	151.42(151.42,147.59,147.59,145.67)
4.79(4.79,0.0	5(5,0,0,0)	4.79(4.79,0.00,0.00,0.00)
11.50(11.50,(	12(12,0,0,0)	11.50(11.50,0.00,0.00,0.00)
292.30(292.3	305(305,304,	292.30(292.30,291.34,291.34,270.26)
130.34(125.5	136(131,135,	130.34(125.55,129.38,124.59,124.59)
0	0	0
0	0	0
0	0	0
487.81(487.8	616(616,606,	590.35(590.35,580.77,580.77,578.85)
0	0	0
226.92(225.4	298(296,291,	226.92(225.40,221.59,220.07,223.87)
3.05(3.05,0.0	4(4,0,0,0)	3.05(3.05,0.00,0.00,0.00)
34.27(34.27,(	45(45,0,0,0)	34.27(34.27,0.00,0.00,0.00)
54.06(54.06,5	72(72,67,67,6	54.83(54.83,51.02,51.02,50.26)
24.37(24.37,2	32(32,30,30,3	24.37(24.37,22.84,22.84,24.37)
0.76(0.76,0.0	1(1,0,0,0)	0.76(0.76,0.00,0.00,0.00)
0	0	0
0	0	0
1519.91(1516	2022(2017,19	1539.71(1535.90,1507.73,1503.92,1439.19)
0	0	0
332.57(327.6	1033(1018,97	336.15(331.27,317.60,313.37,318.90)
4.23(3.58,0.0	13(11,0,0,0)	4.23(3.58,0.00,0.00,0.00)
31.56(31.56,(	97(97,0,0,0)	31.56(31.56,0.00,0.00,0.00)
450.37(449.7	1388(1386,12	451.67(451.02,422.71,422.06,433.45)
189.71(189.0	583(581,569,	189.71(189.06,185.16,184.51,182.23)
11.06(6.18,1(	34(19,33,18,1	11.06(6.18,10.74,5.86,5.86)
0	0	0
0.98(0.65,0.0	3(2,0,0,0)	0.98(0.65,0.00,0.00,0.00)
1126.57(1123	5419(5408,53	1763.40(1759.82,1731.84,1728.26,1690.83)
0	0	0
0	0	0
0	0	0
0	0	0
1228.96(1228	276(276,276,	1228.96(1228.96,1228.96,1228.96,1228.96)
0	0	0
0	0	0
0	0	0
0	0	0
0	0	0
0	0	0
0	0	0
0	0	0

0	0	0
0	0	0
0	0	0
0	0	0
0	0	0
0	0	0
519.94(518.7	643(642,636,	772.11(770.91,763.71,762.51,763.71)
0	0	0
346.11(345.1	720(718,681,	348.53(347.56,329.65,328.68,330.13)
2.42(2.42,0.0	5(5,0,0,0)	2.42(2.42,0.00,0.00,0.00)
55.67(55.67,	115(115,2,2,	55.67(55.67,0.97,0.97,0.97)
570.23(569.2	1179(1177,1	570.71(569.74,552.80,551.83,527.63)
198.47(197.9	416(415,409,	201.37(200.89,197.98,197.50,184.91)
2.90(0.00,2.9	6(0,6,0,4)	2.90(0.00,2.90,0.00,1.94)
0	0	0
0	0	0
549.41(545.0	1736(1722,1	840.33(833.56,814.68,808.39,781.28)
0	0	0
149.46(149.4	343(343,332,	158.22(158.22,153.15,153.15,150.38)
3.23(3.23,0.0	7(7,0,0,0)	3.23(3.23,0.00,0.00,0.00)
40.13(40.13,	87(87,2,2,2)	40.13(40.13,0.92,0.92,0.92)
294.77(294.7	639(639,629,	294.77(294.77,290.15,290.15,286.92)
224.19(223.7	486(485,471,	224.19(223.73,217.27,216.81,216.81)
0	0	0
0	0	0
0.46(0.46,0.0	1(1,0,0,0)	0.46(0.46,0.00,0.00,0.00)
852.01(850.1	3044(3037,2	1404.18(1400.95,1371.89,1369.12,1353.44)
0	0	0
0	0	0
0	0	0
0	0	0
0	0	0
0	0	0
0	0	0
0	0	0
0	0	0
25440.79(25	2519(2515,2	25613.65(25572.98,25288.27,25247.60,24220.61)
0	0	0
79.33(78.62,	112(111,112,	79.33(78.62,79.33,78.62,79.33)
2.83(2.83,0.0	4(4,0,0,0)	2.83(2.83,0.00,0.00,0.00)
121.12(120.4	171(170,0,0,	121.12(120.41,0.00,0.00,0.00)
703.35(703.3	993(993,983,	703.35(703.35,696.27,696.27,685.64)
236.58(235.8	334(333,328,	236.58(235.87,232.33,231.62,230.91)

5.67(0.71,4.9	8(1,7,0,2)	5.67(0.71,4.96,0.00,1.42)
0	0	0
0	0	0
8471.38(8447	22623(22583	16024.08(15995.74,15810.88,15782.54,15664.96)
0	0	0
276.60(276.1	656(655,617,	276.60(276.18,260.16,259.74,264.80)
3.37(2.53,0.0	8(6,0,0,0)	3.37(2.53,0.00,0.00,0.00)
26.14(26.14,(	62(62,0,0,0)	26.14(26.14,0.00,0.00,0.00)
653.14(652.7	1551(1550,13	653.98(653.56,585.67,585.25,627.42)
180.89(180.8	443(443,419,	186.79(186.79,176.67,176.67,176.25)
2.11(1.26,1.6	5(3,4,2,2)	2.11(1.26,1.69,0.84,0.84)
0	0	0
1.26(1.26,0.0	3(3,0,0,0)	1.26(1.26,0.00,0.00,0.00)
2395.40(239(	5922(5910,57	2497.02(2491.96,2436.30,2431.24,2459.07)
0	0	0
545.83(542.0	1283(1274,12	545.83(542.00,531.37,528.39,524.14)
1.28(1.28,0.0	3(3,0,0,0)	1.28(1.28,0.00,0.00,0.00)
116.57(116.1	274(273,2,2,4	116.57(116.14,0.85,0.85,1.70)
941.49(941.0	2217(2216,2(	943.19(942.76,882.35,881.93,907.88)
393.53(393.1	925(924,904,	393.53(393.10,384.59,384.17,377.79)
12.76(1.28,12	30(3,29,2,2)	12.76(1.28,12.34,0.85,0.85)
0	0	0
0	0	0
3763.39(3753	8928(8902,87	3798.28(3787.22,3718.72,3707.66,3597.47)
0	0	0
263.01(262.1	641(639,593,	263.01(262.19,243.31,242.49,252.75)
7.80(6.56,0.0	20(17,0,0,0)	8.21(6.98,0.00,0.00,0.00)
54.98(54.98,(	134(134,2,2,2	54.98(54.98,0.82,0.82,0.82)
798.86(797.2	1975(1971,18	810.35(808.71,765.22,763.58,784.50)
326.60(326.1	798(797,760,	327.42(327.01,311.83,311.42,311.83)
4.10(3.69,1.2	10(9,3,2,2)	4.10(3.69,1.23,0.82,0.82)
4.92(4.92,4.9	12(12,12,12,1	4.92(4.92,4.92,4.92,4.92)
0	0	0
1584.19(1583	4697(4695,46	1927.20(1926.38,1900.53,1899.71,1891.51)
0	0	0
0	0	0
0	0	0
0	0	0
0	0	0
0	0	0
0	0	0
0	0	0
0	0	0

13108.17(130	2485(2480,24	20371.36(20330.37,20166.41,20125.43,19838.50)
0	0	0
574.31(573.5	711(710,703,	574.31(573.50,567.85,567.04,562.19)
0.00(0.00,0.0	0(0,0,0,0)	0.00(0.00,0.00,0.00,0.00)
0	0	0
1330.36(1330	1647(1647,16	1330.36(1330.36,1307.74,1307.74,1282.70)
0	0	0
0	0	0
0	0	0
0	0	0
1167.19(1163	1452(1447,14	1172.85(1168.81,1161.54,1157.50,1143.77)
0	0	0
423.71(423.7	325(325,324,	423.71(423.71,422.41,422.41,417.19)
3.91(3.91,0.0	3(3,0,0,0)	3.91(3.91,0.00,0.00,0.00)
56.06(56.06,0	43(43,0,0,0)	56.06(56.06,0.00,0.00,0.00)
315.50(315.5	242(242,237,	315.50(315.50,308.98,308.98,305.07)
0	0	0
0	0	0
0	0	0
0	0	0
1134.24(1131	880(878,869,	1147.28(1144.67,1132.94,1130.33,1079.48)
0	0	0

th primers, same junction on paired reads with at least a primer).

2781, 2065846, 2167817, 98346, 1411813, 2371630, 2350539, 2437210, 121985, 1238012 and 7

nd the position of the end of leader identified in the most common reads (peak count) on the ref

'67034, excluding the mapped reads unpaired, not primary alignment and supplementary alignm
'erence genome, and “ref_TRS_start” and “peak_TRS_start” refer to the reference position of the

ıent.
ɛ start of TRS and the position of the start of TRS identified in the most common reads (peak cou

nt) on the reference genome.

	subgenome	peak_leader_	peak_TRS_sta	ACGAAC	ATG_postion	20_leader_se
Sample_1	S	65	21552	yes	21563	ATCTCTTGTA(
	ORF3a	69	25385	yes	25393	CTTGTAGATC
	E	69	26237	yes	26245	CTTGTAGATC
	M	65	26469	yes	26523	ATCTCTTGTA(
	ORF6	69	27041	yes	27202	CTTGTAGATC
	N	64	28255	yes	28274	GATCTCTTGT.
Sample_2	S	65	21552	yes	21563	ATCTCTTGTA(
	ORF3a	69	25385	yes	25393	CTTGTAGATC
	E	69	26237	yes	26245	CTTGTAGATC
	M	65	26469	yes	26523	ATCTCTTGTA(
	ORF6	69	27041	yes	27202	CTTGTAGATC
	ORF7a	69	27388	yes	27394	CTTGTAGATC
	N	65	28256	yes	28274	ATCTCTTGTA(
Sample_3	S	65	21552	yes	21563	ATCTCTTGTA(
	ORF3a	69	25385	yes	25393	CTTGTAGATC
	E	69	26237	yes	26245	CTTGTAGATC
	M	65	26469	yes	26523	ATCTCTTGTA(
	ORF6	69	27041	yes	27202	CTTGTAGATC
	ORF7a	69	27388	yes	27394	CTTGTAGATC
	ORF8	65	27884	yes	27894	ATCTCTTGTA(
	N	65	28256	yes	28274	ATCTCTTGTA(
Sample_4	M	65	26469	yes	26523	ATCTCTTGTA(
Sample_5	N	64	28255	yes	28274	GATCTCTTGT.
Sample_6	S	65	21552	yes	21563	ATCTCTTGTA(
	ORF3a	69	25385	yes	25393	CTTGTAGATC
	E	69	26237	yes	26245	CTTGTAGATC
	M	65	26469	yes	26523	ATCTCTTGTA(
	ORF6	69	27041	yes	27202	CTTGTAGATC
	ORF7a	69	27388	yes	27394	CTTGTAGATC
	N	65	28256	yes	28274	ATCTCTTGTA(
Sample_7	S	65	21552	yes	21563	ATCTCTTGTA(
	ORF3a	69	25385	yes	25393	CTTGTAGATC
	E	69	26237	yes	26245	CTTGTAGATC
	M	65	26469	yes	26523	ATCTCTTGTA(
	ORF6	69	27041	yes	27202	CTTGTAGATC
	ORF8	65	27884	yes	27894	ATCTCTTGTA(
	N	65	28256	yes	28274	ATCTCTTGTA(
Sample_8	N	65	28256	yes	28274	ATCTCTTGTA(
Sample_9	S	65	21552	yes	21563	ATCTCTTGTA(
	ORF3a	69	25385	yes	25393	CTTGTAGATC
	E	69	26237	yes	26245	CTTGTAGATC
	M	65	26469	yes	26523	ATCTCTTGTA(

	ORF6	69	27041	yes	27202	CTTGTAGATC
	ORF7a	69	27388	yes	27394	CTTGTAGATC
	N	65	28256	yes	28274	ATCTCTTGTA(
Sample_10	S	65	21552	yes	21563	ATCTCTTGTA(
	ORF3a	69	25385	yes	25393	CTTGTAGATC
	E	69	26237	yes	26245	CTTGTAGATC
	M	65	26469	yes	26523	ATCTCTTGTA(
	ORF6	69	27041	yes	27202	CTTGTAGATC
	ORF7a	69	27388	yes	27394	CTTGTAGATC
	ORF8	65	27884	yes	27894	ATCTCTTGTA(
	N	64	28255	yes	28274	GATCTCTTGT.
Sample_11	S	65	21552	yes	21563	ATCTCTTGTA(
	ORF3a	69	25385	yes	25393	CTTGTAGATC
	E	69	26237	yes	26245	CTTGTAGATC
	M	65	26469	yes	26523	ATCTCTTGTA(
	ORF6	69	27041	yes	27202	CTTGTAGATC
	ORF7a	69	27388	yes	27394	CTTGTAGATC
	N	65	28256	yes	28274	ATCTCTTGTA(
Sample_12	S	65	21552	yes	21563	ATCTCTTGTA(
	ORF3a	69	25385	yes	25393	CTTGTAGATC
	E	69	26237	yes	26245	CTTGTAGATC
	M	65	26469	yes	26523	ATCTCTTGTA(
	ORF6	69	27041	yes	27202	CTTGTAGATC
	ORF7a	69	27388	yes	27394	CTTGTAGATC
	ORF7b	68	27677	no	27756	TCTTGTAGAT
	N	64	28255	yes	28274	GATCTCTTGT.
Sample_13	N	65	28256	yes	28274	ATCTCTTGTA(
Sample_14	S	65	21552	yes	21563	ATCTCTTGTA(
	ORF3a	69	25385	yes	25393	CTTGTAGATC
	M	65	26469	yes	26523	ATCTCTTGTA(
	N	65	28256	yes	28274	ATCTCTTGTA(
Sample_15	S	65	21552	yes	21563	ATCTCTTGTA(
	ORF3a	69	25385	yes	25393	CTTGTAGATC
	E	69	26237	yes	26245	CTTGTAGATC
	M	65	26469	yes	26523	ATCTCTTGTA(
	N	65	28256	yes	28274	ATCTCTTGTA(

“peak_leader” and “peak_TRS_start” point to the leader-TRS junctions in sheet "known", “ACGA

TRS_seq	first_orf_aa
CTAAACGAA	MFVFLVLLPLVSSQCVNLTTRTQLPPAYTNSFTRGVYYPDKVFRSSVLHSTQDLFLPFFSNVTW
ACGAACTT	MDLFMRIFTIGTVTLKQGEIKDATPSDFVRATATIPIQASLPFGWLIVGVALLAVFQSASKIITLK
ACGAACTT	MYSFVSEETGTLIVNSVLLFLAFVVFLLVTLAILTALRLCAYCCNIVNVSLVKPSFYVYSRVKNLN
CTAAACGAA	MADSNGTITVEELKKLLEQWNLVIGFLFLTWICLLQFAYANRNRFLYIIKLIFLWLLWPVTLACF
ACGAACGCT	MFHLVDFQVTIAEILLIIMRTFKVSIWNLDYIINLIIKNLSKSLTENKYSQLDEEQPMEID*
TCTAAACGA	MSDNGPQNQRNAPRITFGGPSDSTGSNQNGERSGARSKQRRPQGLPNNTASWFTALTQH
CTAAACGAA	MFVFLVLLPLVSSQCVNLTTRTQLPPAYTNSFTRGVYYPDKVFRSSVLHSTQDLFLPFFSNVTW
ACGAACTT	MDLFMRIFTIGTVTLKQGEIKDATPSDFVRATATIPIQASLPFGWLIVGVALLAVFQSASKIITLK
ACGAACTT	MYSFVSEETGTLIVNSVLLFLAFVVFLLVTLAILTALRLCAYCCNIVNVSLVKPSFYVYSRVKNLN
CTAAACGAA	MADSNGTITVEELKKLLEQWNLVIGFLFLTWICLLQFAYANRNRFLYIIKLIFLWLLWPVTLACF
ACGAACGCT	MFHLVDFQVTIAEILLIIMRTFKVSIWNLDYIINLIIKNLSKSLTENKYSQLDEEQPMEID*
ACGAAC	MKIILFLALITLATCELYHYQECVRGTTVLLKEPCSSGTYEGNSPFHPLADNKFALTCFSTQFAFA
CTAAACGAA	MSDNGPQNQRNAPRITFGGPSDSTGSNQNGERSGARSKQRRPQGLPNNTASWFTALTQH
CTAAACGAA	MFVFLVLLPLVSSQCVNLTTRTQLPPAYTNSFTRGVYYPDKVFRSSVLHSTQDLFLPFFSNVTW
ACGAACTT	MDLFMRIFTIGTVTLKQGEIKDATPSDFVRATATIPIQASLPFGWLIVGVALLAVFQSASKIITLK
ACGAACTT	MYSFVSEETGTLIVNSVLLFLAFVVFLLVTLAILTALRLCAYCCNIVNVSLVKPSFYVYSRVKNLN
CTAAACGAA	MADSNGTITVEELKKLLEQWNLVIGFLFLTWICLLQFAYANRNRFLYIIKLIFLWLLWPVTLACF
ACGAACGCT	MFHLVDFQVTIAEILLIIMRTFKVSIWNLDYIINLIIKNLSKSLTENKYSQLDEEQPMEID*
ACGAAC	MKIILFLALITLATCELYHYQECVRGTTVLLKEPCSSGTYEGNSPFHPLADNKFALTCFSTQFAFA
CTAAACGAA	MKFLVFLGIITTVAAFHQECSLQSCTQHQPYVVDDPCPIHFYSKWYIRVGARKSAPLIELCVDE
CTAAACGAA	MSDNGPQNQRNAPRITFGGPSDSTGSNQNGERSGARSKQRRPQGLPNNTASWFTALTQH
CTAAACGAA	MADSNGTITVEELKKLLEQWNLVIGFLFLTWICLLQFAYANRNRFLYIIKLIFLWLLWPVTLACF
TCTAAACGA	MSDNGPQNQRNAPRITFGGPSDSTGSNQNGERSGARSKQRRPQGLPNNTASWFTALTQH
CTAAACGAA	MFVFLVLLPLVSSQCVNLTTRTQLPPAYTNSFTRGVYYPDKVFRSSVLHSTQDLFLPFFSNVTW
ACGAACTT	MDLFMRIFTIGTVTLKQGEIKDATPSDFVRATATIPIQASLPFGWLIVGVALLAVFQSASKIITLK
ACGAACTT	MYSFVSEETGTLIVNSVLLFLAFVVFLLVTLAILTALRLCAYCCNIVNVSLVKPSFYVYSRVKNLN
CTAAACGAA	MADSNGTITVEELKKLLEQWNLVIGFLFLTWICLLQFAYANRNRFLYIIKLIFLWLLWPVTLACF
ACGAACGCT	MFHLVDFQVTIAEILLIIMRTFKVSIWNLDYIINLIIKNLSKSLTENKYSQLDEEQPMEID*
ACGAAC	MKIILFLALITLATCELYHYQECVRGTTVLLKEPCSSGTYEGNSPFHPLADNKFALTCFSTQFAFA
CTAAACGAA	MSDNGPQNQRNAPRITFGGPSDSTGSNQNGERSGARSKQRRPQGLPNNTASWFTALTQH
CTAAACGAA	MFVFLVLLPLVSSQCVNLTTRTQLPPAYTNSFTRGVYYPDKVFRSSVLHSTQDLFLPFFSNVTW
ACGAACTT	MDLFMRIFTIGTVTLKQGEIKDATPSDFVRATATIPIQASLPFGWLIVGVALLAVFQSASKIITLK
ACGAACTT	MYSFVSEETGTLIVNSVLLFLAFVVFLLVTLAILTALRLCAYCCNIVNVSLVKPSFYVYSRVKNLN
CTAAACGAA	MADSNGTITVEELKKLLEQWNLVIGFLFLTWICLLQFAYANRNRFLYIIKLIFLWLLWPVTLACF
ACGAACGCT	MFHLVDFQVTIAEILLIIMRTFKVSIWNLDYIINLIIKNLSKSLTENKYSQLDEEQPMEID*
CTAAACGAA	MKFLVFLGIITTVAAFHQECSLQSCTQHQPYVVDDPCPIHFYSKWYIRVGARKSAPLIELCVDE
CTAAACGAA	MSDNGPQNQRNAPRITFGGPSDSTGSNQNGERSGARSKQRRPQGLPNNTASWFTALTQH
CTAAACGAA	MSDNGPQNQRNAPRITFGGPSDSTGSNQNGERSGARSKQRRPQGLPNNTASWFTALTQH
CTAAACGAA	MFVFLVLLPLVSSQCVNLTTRTQLPPAYTNSFTRGVYYPDKVFRSSVLHSTQDLFLPFFSNVTW
ACGAACTT	MDLFMRIFTIGTVTLKQGEIKDATPSDFVRATATIPIQASLPFGWLIVGVALLAVFQSASKIITLK
ACGAACTT	MYSFVSEETGTLIVNSVLLFLAFVVFLLVTLAILTALRLCAYCCNIVNVSLVKPSFYVYSRVKNLN
CTAAACGAA	MADSNGTITVEELKKLLEQWNLVIGFLFLTWICLLQFAYANRNRFLYIIKLIFLWLLWPVTLACF

ACGAACGCTT	MFHLVDFQVTIAEILLIIMRTFKVSIWNLDYIINLIIKNLSKSLTENKYSQLDEEQPMEID*
ACGAAC	MKIILFLALITLATCELYHYQECVRGTTVLLKEPCSSGTYEGNSPFHPLADNKFALTCFSTQFAFA
CTAAACGAAC	MSDNGPQNQRNAPRITFGGPSDSTGSNQNGERSGARSKQRRPQGLPNNTASWFTALTQHG
CTAAACGAAC	MFVFLVLLPLVSSQCVNLTTRTQLPPAYTNSFTRGVYYPDKVFRSSVLHSTQDLFLPFFSNVTW
ACGAACTT	MDLFMRIFTIGTVTLKQGEIKDATPSDFVRATATIPIQASLPFGWLIVGVALLAVFQSASKIITLK
ACGAACTT	MYSFVSEETGTLIVNSVLLFLAFVVFLLVTLAILTALRLCAYCCNIVNVSLVKPSFYVYSRVKNLNS
CTAAACGAAC	MADSNGTITVEELKKLLEQWNLVIGFLFLTWICLLQFAYANRNRFLYIIKLIFLWLLWPVTLACF
ACGAACGCTT	MFHLVDFQVTIAEILLIIMRTFKVSIWNLDYIINLIIKNLSKSLTENKYSQLDEEQPMEID*
ACGAAC	MKIILFLALITLATCELYHYQECVRGTTVLLKEPCSSGTYEGNSPFHPLADNKFALTCFSTQFAFA
CTAAACGAAC	MKFLVFLGIITTVAAFHQECSLQSCTQHQPYVVDDPCPIHFYSKWYIRVGARKSAPLIELCVDEA
TCTAAACGAA	MSDNGPQNQRNAPRITFGGPSDSTGSNQNGERSGARSKQRRPQGLPNNTASWFTALTQHG
CTAAACGAAC	MFVFLVLLPLVSSQCVNLTTRTQLPPAYTNSFTRGVYYPDKVFRSSVLHSTQDLFLPFFSNVTW
ACGAACTT	MDLFMRIFTIGTVTLKQGEIKDATPSDFVRATATIPIQASLPFGWLIVGVALLAVFQSASKIITLK
ACGAACTT	MYSFVSEETGTLIVNSVLLFLAFVVFLLVTLAILTALRLCAYCCNIVNVSLVKPSFYVYSRVKNLNS
CTAAACGAAC	MADSNGTITVEELKKLLEQWNLVIGFLFLTWICLLQFAYANRNRFLYIIKLIFLWLLWPVTLACF
ACGAACGCTT	MFHLVDFQVTIAEILLIIMRTFKVSIWNLDYIINLIIKNLSKSLTENKYSQLDEEQPMEID*
ACGAAC	MKIILFLALITLATCELYHYQECVRGTTVLLKEPCSSGTYEGNSPFHPLADNKFALTCFSTQFAFA
CTAAACGAAC	MSDNGPQNQRNAPRITFGGPSDSTGSNQNGERSGARSKQRRPQGLPNNTASWFTALTQHG
CTAAACGAAC	MFVFLVLLPLVSSQCVNLTTRTQLPPAYTNSFTRGVYYPDKVFRSSVLHSTQDLFLPFFSNVTW
ACGAACTT	MDLFMRIFTIGTVTLKQGEIKDATPSDFVRATATIPIQASLPFGWLIVGVALLAVFQSASKIITLK
ACGAACTT	MYSFVSEETGTLIVNSVLLFLAFVVFLLVTLAILTALRLCAYCCNIVNVSLVKPSFYVYSRVKNLNS
CTAAACGAAC	MADSNGTITVEELKKLLEQWNLVIGFLFLTWICLLQFAYANRNRFLYIIKLIFLWLLWPVTLACF
ACGAACGCTT	MFHLVDFQVTIAEILLIIMRTFKVSIWNLDYIINLIIKNLSKSLTENKYSQLDEEQPMEID*
ACGAAC	MKIILFLALITLATCELYHYQECVRGTTVLLKEPCSSGTYEGNSPFHPLADNKFALTCFSTQFAFA
AACTTTACTC	MIELSLIDFYLCFLAFLLFLVLIMLIIFWFSLELQDHNETCHA*
TCTAAACGAA	MSDNGPQNQRNAPRITFGGPSDSTGSNQNGERSGARSKQRRPQGLPNNTASWFTALTQHG
CTAAACGAAC	MSDNGPQNQRNAPRITFGGPSDSTGSNQNGERSGARSKQRRPQGLPNNTASWFTALTQHG
CTAAACGAAC	MFVFLVLLPLVSSQCVNLTTRTQLPPAYTNSFTRGVYYPDKVFRSSVLHSTQDLFLPFFSNVTW
ACGAACTT	MDLFMRIFTIGTVTLKQGEIKDATPSDFVRATATIPIQASLPFGWLIVGVALLAVFQSASKIITLK
CTAAACGAAC	MADSNGTITVEELKKLLEQWNLVIGFLFLTWICLLQFAYANRNRFLYIIKLIFLWLLWPVTLACF
CTAAACGAAC	MSDNGPQNQRNAPRITFGGPSDSTGSNQNGERSGARSKQRRPQGLPNNTASWFTALTQHG
CTAAACGAAC	MFVFLVLLPLVSSQCVNLTTRTQLPPAYTNSFTRGVYYPDKVFRSSVLHSTQDLFLPFFSNVTW
ACGAACTT	MDLFMRIFTIGTVTLKQGEIKDATPSDFVRATATIPIQASLPFGWLIVGVALLAVFQSASKIITLK
ACGAACTT	MYSFVSEETGTLIVNSVLLFLAFVVFLLVTLAILTALRLCAYCCNIVNVSLVKPSFYVYSRVKNLNS
CTAAACGAAC	MADSNGTITVEELKKLLEQWNLVIGFLFLTWICLLQFAYANRNRFLYIIKLIFLWLLWPVTLACF
CTAAACGAAC	MSDNGPQNQRNAPRITFGGPSDSTGSNQNGERSGARSKQRRPQGLPNNTASWFTALTQHG

AC” indicates if there is a ACGAAC sequence in the “TRS_seq” (TRS sequences), “20_leader_seq”

FHAIHVSGTNGTKRFDNPVLPFNDGVYFASTEKSNIIRGWIFGTTLDSKTQSLLIVNNATNVVIKVCEFQFCND
KRWQLALSKGVHFVCNLLLLFVTVYSHLLLVAAGLEAPFLYLYALVYFLQSINFVRIIMRLWLCWKCRSKNPLLY
SRVPDLLV*
VLAAVYRINWITGGIAIAMACLVGLMWLSYFIASFRLFARTRSMWSFNPETNILLNVPLHGTILTRPLLESELVIG

GKEDLKFPRGQGVPINTNSSPDDQIGYYRRATRRIRGGDGKMKDLSPRWYFYYLGTGPEAGLPYGANKDGII
FHAIHVSGTNGTKRFDNPVLPFNDGVYFASTEKSNIIRGWIFGTTLDSKTQSLLIVNNATNVVIKVCEFQFCND
KRWQLALSKGVHFVCNLLLLFVTVYSHLLLVAAGLEAPFLYLYALVYFLQSINFVRIIMRLWLCWKCRSKNPLLY
SRVPDLLV*
VLAAVYRINWITGGIAIAMACLVGLMWLSYFIASFRLFARTRSMWSFNPETNILLNVPLHGTILTRPLLESELVIG

CPDGVKHVYQLRARSVSPKLFIRQEEVQELYSPIFLIVAAIVFITLCFTLKRKTE*
GKEDLKFPRGQGVPINTNSSPDDQIGYYRRATRRIRGGDGKMKDLSPRWYFYYLGTGPEAGLPYGANKDGII
FHAIHVSGTNGTKRFDNPVLPFNDGVYFASTEKSNIIRGWIFGTTLDSKTQSLLIVNNATNVVIKVCEFQFCND
KRWQLALSKGVHFVCNLLLLFVTVYSHLLLVAAGLEAPFLYLYALVYFLQSINFVRIIMRLWLCWKCRSKNPLLY
SRVPDLLV*
VLAAVYRINWITGGIAIAMACLVGLMWLSYFIASFRLFARTRSMWSFNPETNILLNVPLHGTILTRPLLESELVIG

CPDGVKHVYQLRARSVSPKLFIRQEEVQELYSPIFLIVAAIVFITLCFTLKRKTE*
AGSKSPIQYIDIGNYTVSCLPFTINCQEPKLGSLVVRCSFYEDFLEYHDVRVVLDFI*
GKEDLKFPRGQGVPINTNSSPDDQIGYYRRATRRIRGGDGKMKDLSPRWYFYYLGTGPEAGLPYGANKDGII
VLAAVYRINWITGGIAIAMACLVGLMWLSYFIASFRLFARTRSMWSFNPETNILLNVPLHGTILTRPLLESELVIG
GKEDLKFPRGQGVPINTNSSPDDQIGYYRRATRRIRGGDGKMKDLSPRWYFYYLGTGPEAGLPYGANKDGII
FHAIHVSGTNGTKRFDNPVLPFNDGVYFASTEKSNIIRGWIFGTTLDSKTQSLLIVNNATNVVIKVCEFQFCND
KRWQLALSKGVHFVCNLLLLFVTVYSHLLLVAAGLEAPFLYLYALVYFLQSINFVRIIMRLWLCWKCRSKNPLLY
SRVPDLLV*
VLAAVYRINWITGGIAIAMACLVGLMWLSYFIASFRLFARTRSMWSFNPETNILLNVPLHGTILTRPLLESELVIG

CPDGVKHVYQLRARSVSPKLFIRQEEVQELYSPIFLIVAAIVFITLCFTLKRKTE*
GKEDLKFPRGQGVPINTNSSPDDQIGYYRRATRRIRGGDGKMKDLSPRWYFYYLGTGPEAGLPYGANKDGII
FHAIHVSGTNGTKRFDNPVLPFNDGVYFASTEKSNIIRGWIFGTTLDSKTQSLLIVNNATNVVIKVCEFQFCND
KRWQLALSKGVHFVCNLLLLFVTVYSHLLLVAAGLEAPFLYLYALVYFLQSINFVRIIMRLWLCWKCRSKNPLLY
SRVPDLLV*
VLAAVYRINWITGGIAIAMACLVGLMWLSYFIASFRLFARTRSMWSFNPETNILLNVPLHGTILTRPLLESELVIG

AGSKSPIQYIDIGNYTVSCLPFTINCQEPKLGSLVVRCSFYEDFLEYHDVRVVLDFI*
GKEDLKFPRGQGVPINTNSSPDDQIGYYRRATRRIRGGDGKMKDLSPRWYFYYLGTGPEAGLPYGANKDGII
GKEDLKFPRGQGVPINTNSSPDDQIGYYRRATRRIRGGDGKMKDLSPRWYFYYLGTGPEAGLPYGANKDGII
FHAIHVSGTNGTKRFDNPVLPFNDGVYFASTEKSNIIRGWIFGTTLDSKTQSLLIVNNATNVVIKVCEFQFCND
KRWQLALSKGVHFVCNLLLLFVTVYSHLLLVAAGLEAPFLYLYALVYFLQSINFVRIIMRLWLCWKCRSKNPLLY
SRVPDLLV*
VLAAVYRINWITGGIAIAMACLVGLMWLSYFIASFRLFARTRSMWSFNPETNILLNVPLHGTILTRPLLESELVIG

CPDGVKHVYQLRARSVSPKLFIRQEEVQELYSPIFLIVAAIVFITLCFTLKRKTE*
GKEDLKFPRGQGVPINTNSSPDDQIGYYRRATRRIRGGDGKMKDLSPRWYFYYLGTGPEAGLPYGANKDGII
FHAIHVSGTNGTKRFDNPVLPFNDGVYFASTEKSNIIRGWIFGTTLDSKTQSLLIVNNATNVVIKVCEFQFCND
KRWQLALSKGVHFVCNLLLLFVTVYSHLLLVAAGLEAPFLYLYALVYFLQSINFVRIIMRLWLCWKCRSKNPLLY
SRVPDLLV*
VLAAVYRINWITGGIAIAMACLVGLMWLSYFIASFRLFARTRSMWSFNPETNILLNVPLHGTILTRPLLESELVIG

CPDGVKHVYQLRARSVSPKLFIRQEEVQELYSPIFLIVAAIVFITLCFTLKRKTE*
AGSKSPIQYIDIGNYTVSCLPFTINCQEPKLGSLVVRCSFYEDFLEYHDVRVVLDFI*
GKEDLKFPRGQGVPINTNSSPDDQIGYYRRATRRIRGGDGKMKDLSPRWYFYYLGTGPEAGLPYGANKDGII
FHAIHVSGTNGTKRFDNPVLPFNDGVYFASTEKSNIIRGWIFGTTLDSKTQSLLIVNNATNVVIKVCEFQFCND
KRWQLALSKGVHFVCNLLLLFVTVYSHLLLVAAGLEAPFLYLYALVYFLQSINFVRIIMRLWLCWKCRSKNPLLY
SRVPDLLV*
VLAAVYRINWITGGIAIAMACLVGLMWLSYFIASFRLFARTRSMWSFNPETNILLNVPLHGTILTRPLLESELVIG

CPDGVKHVYQLRARSVSPKLFIRQEEVQELYSPIFLIVAAIVFITLCFTLKRKTE*
GKEDLKFPRGQGVPINTNSSPDDQIGYYRRATRRIRGGDGKMKDLSPRWYFYYLGTGPEAGLPYGANKDGII
FHAIHVSGTNGTKRFDNPVLPFNDGVYFASTEKSNIIRGWIFGTTLDSKTQSLLIVNNATNVVIKVCEFQFCND
KRWQLALSKGVHFVCNLLLLFVTVYSHLLLVAAGLEAPFLYLYALVYFLQSINFVRIIMRLWLCWKCRSKNPLLY
SRVPDLLV*
VLAAVYRINWITGGIAIAMACLVGLMWLSYFIASFRLFARTRSMWSFNPETNILLNVPLHGTILTRPLLESELVIG

CPDGVKHVYQLRARSVSPKLFIRQEEVQELYSPIFLIVAAIVFITLCFTLKRKTE*

GKEDLKFPRGQGVPINTNSSPDDQIGYYRRATRRIRGGDGKMKDLSPRWYFYYLGTGPEAGLPYGANKDGII
GKEDLKFPRGQGVPINTNSSPDDQIGYYRRATRRIRGGDGKMKDLSPRWYFYYLGTGPEAGLPYGANKDGII
FHAIHVSGTNGTKRFDNPVLPFNDGVYFASTEKSNIIRGWIFGTTLDSKTQSLLIVNNATNVVIKVCEFQFCND
KRWQLALSKGVHFVCNLLLLFVTVYSHLLLVAAGLEAPFLYLYALVYFLQSINFVRIIMRLWLCWKCRSKNPLLY
VLAAVYRINWITGGIAIAMACLVGLMWLSYFIASFRLFARTRSMWSFNPETNILLNVPLHGTILTRPLLESELVIG
GKEDLKFPRGQGVPINTNSSPDDQIGYYRRATRRIRGGDGKMKDLSPRWYFYYLGTGPEAGLPYGANKDGII
FHAIHVSGTNGTKRFDNPVLPFNDGVYFASTEKSNIIRGWIFGTTLDSKTQSLLIVNNATNVVIKVCEFQFCND
KRWQLALSKGVHFVCNLLLLFVTVYSHLLLVAAGLEAPFLYLYALVYFLQSINFVRIIMRLWLCWKCRSKNPLLY
SRVPDLLV*
VLAAVYRINWITGGIAIAMACLVGLMWLSYFIASFRLFARTRSMWSFNPETNILLNVPLHGTILTRPLLESELVIG
GKEDLKFPRGQGVPINTNSSPDDQIGYYRRATRRIRGGDGKMKDLSPRWYFYYLGTGPEAGLPYGANKDGII
refers to the 20 nucleotides before the end of leader, and “AUG_postion” and “first_orf_aa” ref

PFLGVYYHKNNKSWMESEFRVYSSANNCTFEYVSQPFLMDLEGKQGNFKNLREFVFKNIDGYFKIYSKHTPINL
DANYFLCWHTNCYDYCIPYNSVTSSIVITSGDGTTSPISEHDYQIGGYTEKWESGVKDCVVLHSYFTSDYYQLYST

AVILRGHLRIAGHHLGRCDIKDLPKEITVATSRTLSYYKLGASQRVAGDSGFAAYSRYRIGNYKLNTDHSSSSDNI

VVATEGALNTPKDHIGTRNPANNAAIVLQLPQGTTLPKGFYAEGSRGGSQASSRSSSRSRNSSRNSTPGSSRGT
PFLGVYYHKNNKSWMESEFRVYSSANNCTFEYVSQPFLMDLEGKQGNFKNLREFVFKNIDGYFKIYSKHTPINL
DANYFLCWHTNCYDYCIPYNSVTSSIVITSGDGTTSPISEHDYQIGGYTEKWESGVKDCVVLHSYFTSDYYQLYST

AVILRGHLRIAGHHLGRCDIKDLPKEITVATSRTLSYYKLGASQRVAGDSGFAAYSRYRIGNYKLNTDHSSSSDNI

VVATEGALNTPKDHIGTRNPANNAAIVLQLPQGTTLPKGFYAEGSRGGSQASSRSSSRSRNSSRNSTPGSSRGT
PFLGVYYHKNNKSWMESEFRVYSSANNCTFEYVSQPFLMDLEGKQGNFKNLREFVFKNIDGYFKIYSKHTPINL
DANYFLCWHTNCYDYCIPYNSVTSSIVITSGDGTTSPISEHDYQIGGYTEKWESGVKDCVVLHSYFTSDYYQLYST

AVILRGHLRIAGHHLGRCDIKDLPKEITVATSRTLSYYKLGASQRVAGDSGFAAYSRYRIGNYKLNTDHSSSSDNI

VVATEGALNTPKDHIGTRNPANNAAIVLQLPQGTTLPKGFYAEGSRGGSQASSRSSSRSRNSSRNSTPGSSRGT
AVILRGHLRIAGHHLGRCDIKDLPKEITVATSRTLSYYKLGASQRVAGDSGFAAYSRYRIGNYKLNTDHSSSSDNI
VVATEGALNTPKDHIGTRNPANNAAIVLQLPQGTTLPKGFYAEGSRGGSQASSRSSSRSRNSSRNSTPGSSRGT
PFLGVYYHKNNKSWMESEFRVYSSANNCTFEYVSQPFLMDLEGKQGNFKNLREFVFKNIDGYFKIYSKHTPINL
DANYFLCWHTNCYDYCIPYNSVTSSIVITSGDGTTSPISEHDYQIGGYTEKWESGVKDCVVLHSYFTSDYYQLYST

AVILRGHLRIAGHHLGRCDIKDLPKEITVATSRTLSYYKLGASQRVAGDSGFAAYSRYRIGNYKLNTDHSSSSDNI

VVATEGALNTPKDHIGTRNPANNAAIVLQLPQGTTLPKGFYAEGSRGGSQASSRSSSRSRNSSRNSTPGSSRGT
PFLGVYYHKNNKSWMESEFRVYSSANNCTFEYVSQPFLMDLEGKQGNFKNLREFVFKNIDGYFKIYSKHTPINL
DANYFLCWHTNCYDYCIPYNSVTSSIVITSGDGTTSPISEHDYQIGGYTEKWESGVKDCVVLHSYFTSDYYQLYST

AVILRGHLRIAGHHLGRCDIKDLPKEITVATSRTLSYYKLGASQRVAGDSGFAAYSRYRIGNYKLNTDHSSSSDNI

VVATEGALNTPKDHIGTRNPANNAAIVLQLPQGTTLPKGFYAEGSRGGSQASSRSSSRSRNSSRNSTPGSSRGT
VVATEGALNTPKDHIGTRNPANNAAIVLQLPQGTTLPKGFYAEGSRGGSQASSRSSSRSRNSSRNSTPGSSRGT
PFLGVYYHKNNKSWMESEFRVYSSANNCTFEYVSQPFLMDLEGKQGNFKNLREFVFKNIDGYFKIYSKHTPINL
DANYFLCWHTNCYDYCIPYNSVTSSIVITSGDGTTSPISEHDYQIGGYTEKWESGVKDCVVLHSYFTSDYYQLYST

AVILRGHLRIAGHHLGRCDIKDLPKEITVATSRTLSYYKLGASQRVAGDSGFAAYSRYRIGNYKLNTDHSSSSDNI

VATEGALNTPKDHIGTRNPANNAAIVLQLPQGTTLPKGFYAEGSRGGSQASSRSSSRSRNSSRNSTPGSSRGT
FLGVYYHKNNKSWMESEFRVYSSANNCTFEYVSQPFLMDLEGKQGNFKNLREFVFKNIDGYFKIYSKHTPINL
ANYFLCWHTNCYDYCIPYNSVTSSIVITSGDGTTSPISEHDYQIGGYTEKWESGVKDCVVLHSYFTSDYYQLYS

AVILRGHLRIAGHHLGRCDIKDLPKEITVATSRTLSYYKLGASQRVAGDSGFAAYSRYRIGNYKLNTDHSSSDNI

VATEGALNTPKDHIGTRNPANNAAIVLQLPQGTTLPKGFYAEGSRGGSQASSRSSSRSRNSSRNSTPGSSRGT
FLGVYYHKNNKSWMESEFRVYSSANNCTFEYVSQPFLMDLEGKQGNFKNLREFVFKNIDGYFKIYSKHTPINL
ANYFLCWHTNCYDYCIPYNSVTSSIVITSGDGTTSPISEHDYQIGGYTEKWESGVKDCVVLHSYFTSDYYQLYS

AVILRGHLRIAGHHLGRCDIKDLPKEITVATSRTLSYYKLGASQRVAGDSGFAAYSRYRIGNYKLNTDHSSSDNI

VATEGALNTPKDHIGTRNPANNAAIVLQLPQGTTLPKGFYAEGSRGGSQASSRSSSRSRNSSRNSTPGSSRGT
FLGVYYHKNNKSWMESEFRVYSSANNCTFEYVSQPFLMDLEGKQGNFKNLREFVFKNIDGYFKIYSKHTPINL
ANYFLCWHTNCYDYCIPYNSVTSSIVITSGDGTTSPISEHDYQIGGYTEKWESGVKDCVVLHSYFTSDYYQLYS

AVILRGHLRIAGHHLGRCDIKDLPKEITVATSRTLSYYKLGASQRVAGDSGFAAYSRYRIGNYKLNTDHSSSDNI

VATEGALNTPKDHIGTRNPANNAAIVLQLPQGTTLPKGFYAEGSRGGSQASSRSSSRSRNSSRNSTPGSSRGT
VATEGALNTPKDHIGTRNPANNAAIVLQLPQGTTLPKGFYAEGSRGGSQASSRSSSRSRNSSRNSTPGSSRGT
FLGVYYHKNNKSWMESEFRVYSSANNCTFEYVSQPFLMDLEGKQGNFKNLREFVFKNIDGYFKIYSKHTPINL
ANYFLCWHTNCYDYCIPYNSVTSSIVITSGDGTTSPISEHDYQIGGYTEKWESGVKDCVVLHSYFTSDYYQLYS
AVILRGHLRIAGHHLGRCDIKDLPKEITVATSRTLSYYKLGASQRVAGDSGFAAYSRYRIGNYKLNTDHSSSDNI
VATEGALNTPKDHIGTRNPANNAAIVLQLPQGTTLPKGFYAEGSRGGSQASSRSSSRSRNSSRNSTPGSSRGT
FLGVYYHKNNKSWMESEFRVYSSANNCTFEYVSQPFLMDLEGKQGNFKNLREFVFKNIDGYFKIYSKHTPINL
ANYFLCWHTNCYDYCIPYNSVTSSIVITSGDGTTSPISEHDYQIGGYTEKWESGVKDCVVLHSYFTSDYYQLYS

AVILRGHLRIAGHHLGRCDIKDLPKEITVATSRTLSYYKLGASQRVAGDSGFAAYSRYRIGNYKLNTDHSSSDNI
VATEGALNTPKDHIGTRNPANNAAIVLQLPQGTTLPKGFYAEGSRGGSQASSRSSSRSRNSSRNSTPGSSRGT
er to the first AUG position and translated orf of the sgmRNA.

VRDLPQGFSALEPLVDLPIGINITRFQTLLALHRSYLTPGDSSSGWTAGAAAYYVGYLQPRTFLLKYNENGTITDA
TQLSTDTGVEHVTFFIYNKIVDEPEEHVQIHTIDGSSGVVNPVMEPIYDEPTTTTSVPL*

ALLVQ*

SPARMAGNGGDAALALLLLDRLNQLESKMSGKGQQQQGQTVTKKSAAEASKKPRQKRTATKAYNVTQAFG
VRDLPQGFSALEPLVDLPIGINITRFQTLLALHRSYLTPGDSSSGWTAGAAAYYVGYLQPRTFLLKYNENGTITDA
TQLSTDTGVEHVTFFIYNKIVDEPEEHVQIHTIDGSSGVVNPVMEPIYDEPTTTTSVPL*

ALLVQ*

SPARMAGNGGDAALALLLLDRLNQLESKMSGKGQQQQGQTVTKKSAAEASKKPRQKRTATKAYNVTQAFG
VRDLPQGFSALEPLVDLPIGINITRFQTLLALHRSYLTPGDSSSGWTAGAAAYYVGYLQPRTFLLKYNENGTITDA
TQLSTDTGVEHVTFFIYNKIVDEPEEHVQIHTIDGSSGVVNPVMEPIYDEPTTTTSVPL*

ALLVQ*

SPARMAGNGGDAALALLLLDRLNQLESKMSGKGQQQQGQTVTKKSAAEASKKPRQKRTATKAYNVTQAFG
ALLVQ*
SPARMAGNGGDAALALLLLDRLNQLESKMSGKGQQQQGQTVTKKSAAEASKKPRQKRTATKAYNVTQAFG
VRDLPQGFSALEPLVDLPIGINITRFQTLLALHRSYLTPGDSSSGWTAGAAAYYVGYLQPRTFLLKYNENGTITDA
TQLSTDTGVEHVTFFIYNKIVDEPEEHVQIHTIDGSSGVVNPVMEPIYDEPTTTTSVPL*

ALLVQ*

SPARMAGNGGDAALALLLLDRLNQLESKMSGKGQQQQGQTVTKKSAAEASKKPRQKRTATKAYNVTQAFG
VRDLPQGFSALEPLVDLPIGINITRFQTLLALHRSYLTPGDSSSGWTAGAAAYYVGYLQPRTFLLKYNENGTITDA
TQLSTDTGVEHVTFFIYNKIVDEPEEHVQIHTIDGSSGVVNPVMEPIYDEPTTTTSVPL*

ALLVQ*

SPARMAGNGGDAALALLLLDRLNQLESKMSGKGQQQQGQTVTKKSAAEASKKPRQKRTATKAYNVTQAFG
SPARMAGNGGDAALALLLLDRLNQLESKMSGKGQQQQGQTVTKKSAAEASKKPRQKRTATKAYNVTQAFG
VRDLPQGFSALEPLVDLPIGINITRFQTLLALHRSYLTPGDSSSGWTAGAAAYYVGYLQPRTFLLKYNENGTITDA
TQLSTDTGVEHVTFFIYNKIVDEPEEHVQIHTIDGSSGVVNPVMEPIYDEPTTTTSVPL*

ALLVQ*

SPARMAGNGGDAALALLLLDRLNQLESKMSGKGQQQQGQTVTKKSAAEASKKPRQKRTATKAYNVTQAFG[illegible]
VRDLPQGFSALEPLVDLPIGINITRFQTLLALHRSYLTPGDSSSGWTAGAAAYYVGYLQPRTFLLKYNENGTITDA
TQLSTDTGVEHVTFFIYNKIVDEPEEHVQIHTIDGSSGVVNPVMEPIYDEPTTTTSVPL*

ALLVQ*

SPARMAGNGGDAALALLLLDRLNQLESKMSGKGQQQQGQTVTKKSAAEASKKPRQKRTATKAYNVTQAFG[illegible]
VRDLPQGFSALEPLVDLPIGINITRFQTLLALHRSYLTPGDSSSGWTAGAAAYYVGYLQPRTFLLKYNENGTITDA
TQLSTDTGVEHVTFFIYNKIVDEPEEHVQIHTIDGSSGVVNPVMEPIYDEPTTTTSVPL*

ALLVQ*

SPARMAGNGGDAALALLLLDRLNQLESKMSGKGQQQQGQTVTKKSAAEASKKPRQKRTATKAYNVTQAFG[illegible]
VRDLPQGFSALEPLVDLPIGINITRFQTLLALHRSYLTPGDSSSGWTAGAAAYYVGYLQPRTFLLKYNENGTITDA
TQLSTDTGVEHVTFFIYNKIVDEPEEHVQIHTIDGSSGVVNPVMEPIYDEPTTTTSVPL*

ALLVQ*

SPARMAGNGGDAALALLLLDRLNQLESKMSGKGQQQQGQTVTKKSAAEASKKPRQKRTATKAYNVTQAFG[illegible]
SPARMAGNGGDAALALLLLDRLNQLESKMSGKGQQQQGQTVTKKSAAEASKKPRQKRTATKAYNVTQAFG[illegible]
VRDLPQGFSALEPLVDLPIGINITRFQTLLALHRSYLTPGDSSSGWTAGAAAYYVGYLQPRTFLLKYNENGTITDA
TQLSTDTGVEHVTFFIYNKIVDEPEEHVQIHTIDGSSGVVNPVMEPIYDEPTTTTSVPL*
ALLVQ*
SPARMAGNGGDAALALLLLDRLNQLESKMSGKGQQQQGQTVTKKSAAEASKKPRQKRTATKAYNVTQAFG[illegible]
VRDLPQGFSALEPLVDLPIGINITRFQTLLALHRSYLTPGDSSSGWTAGAAAYYVGYLQPRTFLLKYNENGTITDA
TQLSTDTGVEHVTFFIYNKIVDEPEEHVQIHTIDGSSGVVNPVMEPIYDEPTTTTSVPL*

ALLVQ*
SPARMAGNGGDAALALLLLDRLNQLESKMSGKGQQQQGQTVTKKSAAEASKKPRQKRTATKAYNVTQAFG[illegible]

AVDCALDPLSETKCTLKSFTVEKGIYQTSNFRVQPTESIVRFPNITNLCPFGEVFNATRFASVYAWNRKRISNCVA

RRGPEQTQGNFGDQELIRQGTDYKHWPQIAQFAPSASAFFGMSRIGMEVTPSGTWLTYTGAIKLDDKDPNFK
AVDCALDPLSETKCTLKSFTVEKGIYQTSNFRVQPTESIVRFPNITNLCPFGEVFNATRFASVYAWNRKRISNCVA

RRGPEQTQGNFGDQELIRQGTDYKHWPQIAQFAPSASAFFGMSRIGMEVTPSGTWLTYTGAIKLDDKDPNFK
AVDCALDPLSETKCTLKSFTVEKGIYQTSNFRVQPTESIVRFPNITNLCPFGEVFNATRFASVYAWNRKRISNCVA

RRGPEQTQGNFGDQELIRQGTDYKHWPQIAQFAPSASAFFGMSRIGMEVTPSGTWLTYTGAIKLDDKDPNFK

RRGPEQTQGNFGDQELIRQGTDYKHWPQIAQFAPSASAFFGMSRIGMEVTPSGTWLTYTGAIKLDDKDPNFK
AVDCALDPLSETKCTLKSFTVEKGIYQTSNFRVQPTESIVRFPNITNLCPFGEVFNATRFASVYAWNRKRISNCVA

RRGPEQTQGNFGDQELIRQGTDYKHWPQIAQFAPSASAFFGMSRIGMEVTPSGTWLTYTGAIKLDDKDPNFK
AVDCALDPLSETKCTLKSFTVEKGIYQTSNFRVQPTESIVRFPNITNLCPFGEVFNATRFASVYAWNRKRISNCVA

RRGPEQTQGNFGDQELIRQGTDYKHWPQIAQFAPSASAFFGMSRIGMEVTPSGTWLTYTGAIKLDDKDPNFK
RRGPEQTQGNFGDQELIRQGTDYKHWPQIAQFAPSASAFFGMSRIGMEVTPSGTWLTYTGAIKLDDKDPNFK
AVDCALDPLSETKCTLKSFTVEKGIYQTSNFRVQPTESIVRFPNITNLCPFGEVFNATRFASVYAWNRKRISNCVA

RRGPEQTQGNFGDQELIRQGTDYKHWPQIAQFAPSASAFFGMSRIGMEVTPSGTWLTYTGAIKLDDKDPNFK
AVDCALDPLSETKCTLKSFTVEKGIYQTSNFRVQPTESIVRFPNITNLCPFGEVFNATRFASVYAWNRKRISNCVA

RRGPEQTQGNFGDQELIRQGTDYKHWPQIAQFAPSASAFFGMSRIGMEVTPSGTWLTYTGAIKLDDKDPNFK
AVDCALDPLSETKCTLKSFTVEKGIYQTSNFRVQPTESIVRFPNITNLCPFGEVFNATRFASVYAWNRKRISNCVA

RRGPEQTQGNFGDQELIRQGTDYKHWPQIAQFAPSASAFFGMSRIGMEVTPSGTWLTYTGAIKLDDKDPNFK
AVDCALDPLSETKCTLKSFTVEKGIYQTSNFRVQPTESIVRFPNITNLCPFGEVFNATRFASVYAWNRKRISNCVA

RRGPEQTQGNFGDQELIRQGTDYKHWPQIAQFAPSASAFFGMSRIGMEVTPSGTWLTYTGAIKLDDKDPNFK
RRGPEQTQGNFGDQELIRQGTDYKHWPQIAQFAPSASAFFGMSRIGMEVTPSGTWLTYTGAIKLDDKDPNFK
AVDCALDPLSETKCTLKSFTVEKGIYQTSNFRVQPTESIVRFPNITNLCPFGEVFNATRFASVYAWNRKRISNCVA

RRGPEQTQGNFGDQELIRQGTDYKHWPQIAQFAPSASAFFGMSRIGMEVTPSGTWLTYTGAIKLDDKDPNFK
AVDCALDPLSETKCTLKSFTVEKGIYQTSNFRVQPTESIVRFPNITNLCPFGEVFNATRFASVYAWNRKRISNCVA

RRGPEQTQGNFGDQELIRQGTDYKHWPQIAQFAPSASAFFGMSRIGMEVTPSGTWLTYTGAIKLDDKDPNFK

DYSVLYNSASFSTFKCYGVSPTKLNDLCFTNVYADSFVIRGDEVRQIAPGQTGKIADYNYKLPDDFTGCVIAWN

DQVILLNKHIDAYKTFPPTEPKKDKKKKADETQALPQRQKKQQTVTLLPAADLDDFSKQLQQSMSSADSTQA
DYSVLYNSASFSTFKCYGVSPTKLNDLCFTNVYADSFVIRGDEVRQIAPGQTGKIADYNYKLPDDFTGCVIAWN

DQVILLNKHIDAYKTFPPTEPKKDKKKKADETQALPQRQKKQQTVTLLPAADLDDFSKQLQQSMSSADSTQA
DYSVLYNSASFSTFKCYGVSPTKLNDLCFTNVYADSFVIRGDEVRQIAPGQTGKIADYNYKLPDDFTGCVIAWN

DQVILLNKHIDAYKTFPPTEPKKDKKKKADETQALPQRQKKQQTVTLLPAADLDDFSKQLQQSMSSADSTQA

DQVILLNKHIDAYKTFPPTEPKKDKKKKADETQALPQRQKKQQTVTLLPAADLDDFSKQLQQSMSSADSTQA
DYSVLYNSASFSTFKCYGVSPTKLNDLCFTNVYADSFVIRGDEVRQIAPGQTGKIADYNYKLPDDFTGCVIAWN

DQVILLNKHIDAYKTFPPTEPKKDKKKKADETQALPQRQKKQQTVTLLPAADLDDFSKQLQQSMSSADSTQA
DYSVLYNSASFSTFKCYGVSPTKLNDLCFTNVYADSFVIRGDEVRQIAPGQTGKIADYNYKLPDDFTGCVIAWN

DQVILLNKHIDAYKTFPPTEPKKDKKKKADETQALPQRQKKQQTVTLLPAADLDDFSKQLQQSMSSADSTQA
DQVILLNKHIDAYKTFPPTEPKKDKKKKADETQALPQRQKKQQTVTLLPAADLDDFSKQLQQSMSSADSTQA
DYSVLYNSASFSTFKCYGVSPTKLNDLCFTNVYADSFVIRGDEVRQIAPGQTGKIADYNYKLPDDFTGCVIAWN

DQVILLNKHIDAYKTFPPTEPKKDKKKKADETQALPQRQKKQQTVTLLPAADLDDFSKQLQQSMSSADSTQA
DYSVLYNSASFSTFKCYGVSPTKLNDLCFTNVYADSFVIRGDEVRQIAPGQTGKIADYNYKLPDDFTGCVIAWN

DQVILLNKHIDAYKTFPPTEPKKDKKKKADETQALPQRQKKQQTVTLLPAADLDDFSKQLQQSMSSADSTQA
DYSVLYNSASFSTFKCYGVSPTKLNDLCFTNVYADSFVIRGDEVRQIAPGQTGKIADYNYKLPDDFTGCVIAWN

DQVILLNKHIDAYKTFPPTEPKKDKKKKADETQALPQRQKKQQTVTLLPAADLDDFSKQLQQSMSSADSTQA
DYSVLYNSASFSTFKCYGVSPTKLNDLCFTNVYADSFVIRGDEVRQIAPGQTGKIADYNYKLPDDFTGCVIAWN

DQVILLNKHIDAYKTFPPTEPKKDKKKKADETQALPQRQKKQQTVTLLPAADLDDFSKQLQQSMSSADSTQA
DQVILLNKHIDAYKTFPPTEPKKDKKKKADETQALPQRQKKQQTVTLLPAADLDDFSKQLQQSMSSADSTQA
DYSVLYNSASFSTFKCYGVSPTKLNDLCFTNVYADSFVIRGDEVRQIAPGQTGKIADYNYKLPDDFTGCVIAWN

DQVILLNKHIDAYKTFPPTEPKKDKKKKADETQALPQRQKKQQTVTLLPAADLDDFSKQLQQSMSSADSTQA
DYSVLYNSASFSTFKCYGVSPTKLNDLCFTNVYADSFVIRGDEVRQIAPGQTGKIADYNYKLPDDFTGCVIAWN

DQVILLNKHIDAYKTFPPTEPKKDKKKKADETQALPQRQKKQQTVTLLPAADLDDFSKQLQQSMSSADSTQA

SNNLDSKVGGNYNYLYRLFRKSNLKPFERDISTEIYQAGSTPCNGVEGFNCYFPLQSYGFQPTNGVGYQPYRVV

*

SNNLDSKVGGNYNYLYRLFRKSNLKPFERDISTEIYQAGSTPCNGVEGFNCYFPLQSYGFQPTNGVGYQPYRVV

*

SNNLDSKVGGNYNYLYRLFRKSNLKPFERDISTEIYQAGSTPCNGVEGFNCYFPLQSYGFQPTNGVGYQPYRVV

*

*

SNNLDSKVGGNYNYLYRLFRKSNLKPFERDISTEIYQAGSTPCNGVEGFNCYFPLQSYGFQPTNGVGYQPYRVV

*

SNNLDSKVGGNYNYLYRLFRKSNLKPFERDISTEIYQAGSTPCNGVEGFNCYFPLQSYGFQPTNGVGYQPYRVV

*

*

SNNLDSKVGGNYNYLYRLFRKSNLKPFERDISTEIYQAGSTPCNGVEGFNCYFPLQSYGFQPTNGVGYQPYRVV

*

SNNLDSKVGGNYNYLYRLFRKSNLKPFERDISTEIYQAGSTPCNGVEGFNCYFPLQSYGFQPTNGVGYQPYRVV

*

SNNLDSKVGGNYNYLYRLFRKSNLKPFERDISTEIYQAGSTPCNGVEGFNCYFPLQSYGFQPTNGVGYQPYRVV

*

SNNLDSKVGGNYNYLYRLFRKSNLKPFERDISTEIYQAGSTPCNGVEGFNCYFPLQSYGFQPTNGVGYQPYRVV

*

*

SNNLDSKVGGNYNYLYRLFRKSNLKPFERDISTEIYQAGSTPCNGVEGFNCYFPLQSYGFQPTNGVGYQPYRVV

*

SNNLDSKVGGNYNYLYRLFRKSNLKPFERDISTEIYQAGSTPCNGVEGFNCYFPLQSYGFQPTNGVGYQPYRVV

*

'VLSFELLHAPATVCGPKKSTNLVKNKCVNFNFNGLTGTGVLTESNKKFLPFQQFGRDIADTTDAVRDPQTLEILI

'VLSFELLHAPATVCGPKKSTNLVKNKCVNFNFNGLTGTGVLTESNKKFLPFQQFGRDIADTTDAVRDPQTLEILI

'VLSFELLHAPATVCGPKKSTNLVKNKCVNFNFNGLTGTGVLTESNKKFLPFQQFGRDIADTTDAVRDPQTLEILI

'VLSFELLHAPATVCGPKKSTNLVKNKCVNFNFNGLTGTGVLTESNKKFLPFQQFGRDIADTTDAVRDPQTLEILI

'VLSFELLHAPATVCGPKKSTNLVKNKCVNFNFNGLTGTGVLTESNKKFLPFQQFGRDIADTTDAVRDPQTLEILI

'VLSFELLHAPATVCGPKKSTNLVKNKCVNFNFNGLTGTGVLTESNKKFLPFQQFGRDIADTTDAVRDPQTLEILI

'VLSFELLHAPATVCGPKKSTNLVKNKCVNFNFNGLTGTGVLTESNKKFLPFQQFGRDIADTTDAVRDPQTLEILI

'VLSFELLHAPATVCGPKKSTNLVKNKCVNFNFNGLTGTGVLTESNKKFLPFQQFGRDIADTTDAVRDPQTLEILI

'VLSFELLHAPATVCGPKKSTNLVKNKCVNFNFNGLTGTGVLTESNKKFLPFQQFGRDIADTTDAVRDPQTLEILI

'VLSFELLHAPATVCGPKKSTNLVKNKCVNFNFNGLTGTGVLTESNKKFLPFQQFGRDIADTTDAVRDPQTLEILI

'VLSFELLHAPATVCGPKKSTNLVKNKCVNFNFNGLTGTGVLTESNKKFLPFQQFGRDIADTTDAVRDPQTLEILI

DITPCSFGGVSVITPGTNTSNQVAVLYQDVNCTEVPVAIHADQLTPTWRVYSTGSNVFQTRAGCLIGAEHVNN

DITPCSFGGVSVITPGTNTSNQVAVLYQDVNCTEVPVAIHADQLTPTWRVYSTGSNVFQTRAGCLIGAEHVNN

DITPCSFGGVSVITPGTNTSNQVAVLYQDVNCTEVPVAIHADQLTPTWRVYSTGSNVFQTRAGCLIGAEHVNN

DITPCSFGGVSVITPGTNTSNQVAVLYQDVNCTEVPVAIHADQLTPTWRVYSTGSNVFQTRAGCLIGAEHVNN

DITPCSFGGVSVITPGTNTSNQVAVLYQDVNCTEVPVAIHADQLTPTWRVYSTGSNVFQTRAGCLIGAEHVNN

DITPCSFGGVSVITPGTNTSNQVAVLYQDVNCTEVPVAIHADQLTPTWRVYSTGSNVFQTRAGCLIGAEHVNN

DITPCSFGGVSVITPGTNTSNQVAVLYQDVNCTEVPVAIHADQLTPTWRVYSTGSNVFQTRAGCLIGAEHVNN

DITPCSFGGVSVITPGTNTSNQVAVLYQDVNCTEVPVAIHADQLTPTWRVYSTGSNVFQTRAGCLIGAEHVNN

DITPCSFGGVSVITPGTNTSNQVAVLYQDVNCTEVPVAIHADQLTPTWRVYSTGSNVFQTRAGCLIGAEHVNN

DITPCSFGGVSVITPGTNTSNQVAVLYQDVNCTEVPVAIHADQLTPTWRVYSTGSNVFQTRAGCLIGAEHVNN

DITPCSFGGVSVITPGTNTSNQVAVLYQDVNCTEVPVAIHADQLTPTWRVYSTGSNVFQTRAGCLIGAEHVNN

SYECDIPIGAGICASYQTQTNSPRRARSVASQSIIAYTMSLGAENSVAYSNNSIAIPTNFTISVTTEILPVSMTKTS

SYECDIPIGAGICASYQTQTNSPRRARSVASQSIIAYTMSLGAENSVAYSNNSIAIPTNFTISVTTEILPVSMTKTS

SYECDIPIGAGICASYQTQTNSPRRARSVASQSIIAYTMSLGAENSVAYSNNSIAIPTNFTISVTTEILPVSMTKTS

SYECDIPIGAGICASYQTQTNSPRRARSVASQSIIAYTMSLGAENSVAYSNNSIAIPTNFTISVTTEILPVSMTKTS

SYECDIPIGAGICASYQTQTNSPRRARSVASQSIIAYTMSLGAENSVAYSNNSIAIPTNFTISVTTEILPVSMTKTS

SYECDIPIGAGICASYQTQTNSPRRARSVASQSIIAYTMSLGAENSVAYSNNSIAIPTNFTISVTTEILPVSMTKTS

SYECDIPIGAGICASYQTQTNSPRRARSVASQSIIAYTMSLGAENSVAYSNNSIAIPTNFTISVTTEILPVSMTKTS

SYECDIPIGAGICASYQTQTNSPRRARSVASQSIIAYTMSLGAENSVAYSNNSIAIPTNFTISVTTEILPVSMTKTS

SYECDIPIGAGICASYQTQTNSPRRARSVASQSIIAYTMSLGAENSVAYSNNSIAIPTNFTISVTTEILPVSMTKTS

SYECDIPIGAGICASYQTQTNSPRRARSVASQSIIAYTMSLGAENSVAYSNNSIAIPTNFTISVTTEILPVSMTKTS

SYECDIPIGAGICASYQTQTNSPRRARSVASQSIIAYTMSLGAENSVAYSNNSIAIPTNFTISVTTEILPVSMTKTS

VDCTMYICGDSTECSNLLLQYGSFCTQLNRALTGIAVEQDKNTQEVFAQVKQIYKTPPIKDFGGFNFSQILPDPS

VDCTMYICGDSTECSNLLLQYGSFCTQLNRALTGIAVEQDKNTQEVFAQVKQIYKTPPIKDFGGFNFSQILPDPS

VDCTMYICGDSTECSNLLLQYGSFCTQLNRALTGIAVEQDKNTQEVFAQVKQIYKTPPIKDFGGFNFSQILPDPS

VDCTMYICGDSTECSNLLLQYGSFCTQLNRALTGIAVEQDKNTQEVFAQVKQIYKTPPIKDFGGFNFSQILPDPS

VDCTMYICGDSTECSNLLLQYGSFCTQLNRALTGIAVEQDKNTQEVFAQVKQIYKTPPIKDFGGFNFSQILPDPS

VDCTMYICGDSTECSNLLLQYGSFCTQLNRALTGIAVEQDKNTQEVFAQVKQIYKTPPIKDFGGFNFSQILPDPS

VDCTMYICGDSTECSNLLLQYGSFCTQLNRALTGIAVEQDKNTQEVFAQVKQIYKTPPIKDFGGFNFSQILPDPS

VDCTMYICGDSTECSNLLLQYGSFCTQLNRALTGIAVEQDKNTQEVFAQVKQIYKTPPIKDFGGFNFSQILPDPS

VDCTMYICGDSTECSNLLLQYGSFCTQLNRALTGIAVEQDKNTQEVFAQVKQIYKTPPIKDFGGFNFSQILPDPS

VDCTMYICGDSTECSNLLLQYGSFCTQLNRALTGIAVEQDKNTQEVFAQVKQIYKTPPIKDFGGFNFSQILPDPS

VDCTMYICGDSTECSNLLLQYGSFCTQLNRALTGIAVEQDKNTQEVFAQVKQIYKTPPIKDFGGFNFSQILPDPS

KPSKRSFIEDLLFNKVTLADAGFIKQYGDCLGDIAARDLICAQKFNGLTVLPPLLTDEMIAQYTSALLAGTITSGW

KPSKRSFIEDLLFNKVTLADAGFIKQYGDCLGDIAARDLICAQKFNGLTVLPPLLTDEMIAQYTSALLAGTITSGW

KPSKRSFIEDLLFNKVTLADAGFIKQYGDCLGDIAARDLICAQKFNGLTVLPPLLTDEMIAQYTSALLAGTITSGW

KPSKRSFIEDLLFNKVTLADAGFIKQYGDCLGDIAARDLICAQKFNGLTVLPPLLTDEMIAQYTSALLAGTITSGW

KPSKRSFIEDLLFNKVTLADAGFIKQYGDCLGDIAARDLICAQKFNGLTVLPPLLTDEMIAQYTSALLAGTITSGW

KPSKRSFIEDLLFNKVTLADAGFIKQYGDCLGDIAARDLICAQKFNGLTVLPPLLTDEMIAQYTSALLAGTITSGW

KPSKRSFIEDLLFNKVTLADAGFIKQYGDCLGDIAARDLICAQKFNGLTVLPPLLTDEMIAQYTSALLAGTITSGW

KPSKRSFIEDLLFNKVTLADAGFIKQYGDCLGDIAARDLICAQKFNGLTVLPPLLTDEMIAQYTSALLAGTITSGW

KPSKRSFIEDLLFNKVTLADAGFIKQYGDCLGDIAARDLICAQKFNGLTVLPPLLTDEMIAQYTSALLAGTITSGW

KPSKRSFIEDLLFNKVTLADAGFIKQYGDCLGDIAARDLICAQKFNGLTVLPPLLTDEMIAQYTSALLAGTITSGW

KPSKRSFIEDLLFNKVTLADAGFIKQYGDCLGDIAARDLICAQKFNGLTVLPPLLTDEMIAQYTSALLAGTITSGW

TFGAGAALQIPFAMQMAYRFNGIGVTQNVLYENQKLIANQFNSAIGKIQDSLSSTASALGKLQDVVNQNAQA

TFGAGAALQIPFAMQMAYRFNGIGVTQNVLYENQKLIANQFNSAIGKIQDSLSSTASALGKLQDVVNQNAQA

TFGAGAALQIPFAMQMAYRFNGIGVTQNVLYENQKLIANQFNSAIGKIQDSLSSTASALGKLQDVVNQNAQA

TFGAGAALQIPFAMQMAYRFNGIGVTQNVLYENQKLIANQFNSAIGKIQDSLSSTASALGKLQDVVNQNAQA

TFGAGAALQIPFAMQMAYRFNGIGVTQNVLYENQKLIANQFNSAIGKIQDSLSSTASALGKLQDVVNQNAQA

TFGAGAALQIPFAMQMAYRFNGIGVTQNVLYENQKLIANQFNSAIGKIQDSLSSTASALGKLQDVVNQNAQA

TFGAGAALQIPFAMQMAYRFNGIGVTQNVLYENQKLIANQFNSAIGKIQDSLSSTASALGKLQDVVNQNAQA

TFGAGAALQIPFAMQMAYRFNGIGVTQNVLYENQKLIANQFNSAIGKIQDSLSSTASALGKLQDVVNQNAQA

TFGAGAALQIPFAMQMAYRFNGIGVTQNVLYENQKLIANQFNSAIGKIQDSLSSTASALGKLQDVVNQNAQA

TFGAGAALQIPFAMQMAYRFNGIGVTQNVLYENQKLIANQFNSAIGKIQDSLSSTASALGKLQDVVNQNAQA

TFGAGAALQIPFAMQMAYRFNGIGVTQNVLYENQKLIANQFNSAIGKIQDSLSSTASALGKLQDVVNQNAQA

ALNTLVKQLSSNFGAISSVLNDILSRLDKVEAEVQIDRLITGRLQSLQTYVTQQLIRAAEIRASANL

ALNTLVKQLSSNFGAISSVLNDILSRLDKVEAEVQIDRLITGRLQSLQTYVTQQLIRAAEIRASANL

ALNTLVKQLSSNFGAISSVLNDILSRLDKVEAEVQIDRLITGRLQSLQTYVTQQLIRAAEIRASANL

ALNTLVKQLSSNFGAISSVLNDILSRLDKVEAEVQIDRLITGRLQSLQTYVTQQLIRAAEIRASANL

ALNTLVKQLSSNFGAISSVLNDILSRLDKVEAEVQIDRLITGRLQSLQTYVTQQLIRAAEIRASANL

ALNTLVKQLSSNFGAISSVLNDILSRLDKVEAEVQIDRLITGRLQSLQTYVTQQLIRAAEIRASANL

ALNTLVKQLSSNFGAISSVLNDILSRLDKVEAEVQIDRLITGRLQSLQTYVTQQLIRAAEIRASANL

ALNTLVKQLSSNFGAISSVLNDILSRLDKVEAEVQIDRLITGRLQSLQTYVTQQLIRAAEIRASANL

ALNTLVKQLSSNFGAISSVLNDILSRLDKVEAEVQIDRLITGRLQSLQTYVTQQLIRAAEIRASANL

ALNTLVKQLSSNFGAISSVLNDILSRLDKVEAEVQIDRLITGRLQSLQTYVTQQLIRAAEIRASANL

ALNTLVKQLSSNFGAISSVLNDILSRLDKVEAEVQIDRLITGRLQSLQTYVTQQLIRAAEIRASANL

	subgenome	leader_end	TRS_start	nb_count	normalized_count
Sample_1	1	70	2794	16(16,16,16,1	15.33(15.33,15.33,15.33,13
	2	74	21058	12(12,12,12,1	11.50(11.50,11.50,11.50,9.5
Sample_2	subgenome	leader_end	TRS_start	nb_count	normalized_count
Sample_3	1	67	25800	12(12,10,10,1	3.90(3.90,3.25,3.25,3.90)
	2	69	7503	34(34,34,34,3	11.06(11.06,11.06,11.06,11
	3	48	26443	14(14,14,14,1	4.56(4.56,4.56,4.56,4.56)
	4	74	21058	50(48,50,48,4	16.27(15.62,16.27,15.62,15
	5	75	28265	11(11,11,11,8	3.58(3.58,3.58,3.58,2.60)
Sample_4	subgenome	leader_end	TRS_start	nb_count	normalized_count
Sample_5	subgenome	leader_end	TRS_start	nb_count	normalized_count
Sample_6	1	48	26443	12(12,12,12,1	5.81(5.81,5.81,5.81,5.81)
	2	74	21058	14(14,14,14,1	6.78(6.78,6.78,6.78,5.81)
Sample_8	1	70	2794	42(42,42,42,4	19.37(19.37,19.37,19.37,18
Sample_9	subgenome	leader_end	TRS_start	nb_count	normalized_count
Sample_10	subgenome	leader_end	TRS_start	nb_count	normalized_count
Sample_11	1	72	22107	13(7,6,0,12)	5.48(2.95,2.53,0.00,5.06)
	2	74	4555	16(16,16,16,1	6.75(6.75,6.75,6.75,5.90)
	3	74	21058	40(40,40,40,4	16.87(16.87,16.87,16.87,16
	4	63	5785	22(22,22,22,2	9.28(9.28,9.28,9.28,9.28)
Sample_12	subgenome	leader_end	TRS_start	nb_count	normalized_count
Sample_14	1	67	4018	30(30,30,30,3	12.31(12.31,12.31,12.31,12
	2	68	4580	80(80,74,74,7	32.82(32.82,30.36,30.36,31
	3	75	26486	40(40,40,40,3	16.41(16.41,16.41,16.41,15
	4	63	5785	28(28,26,26,2	11.49(11.49,10.67,10.67,10
Sample_17					
Sample_18					
Sample_19					

The numbers in the bracket are (reads with left primers, reads with right primers, reads with bot

Normalized count=(Read count/Total number of read mapped on reference genome)*1000000.

Total number of read mapped on reference genome is 1043447, 1313236, 3073041, 224581, 83

“leader_end” and “TRS_start” refer to the position of the end of leader and the position of the s

¦.42)
58)

..06)

¦.62)

¦.45)

¦.87)

¦.31)
..18)
¦.59)
¦.67)

th primers, same junction on paired reads with at least a primer).

2781, 2065846, 2167817, 98346, 1411813, 2371630, 2350539, 2437210, 121985, 1238012 and 7
start of TRS identified in the reads >10.

'67034, excluding the mapped reads unpaired, not primary alignment and supplementary alignm

ent.

	subgenome	leader_end	TRS_start	ACGAAC	ATG_postion	known_ATG
Sample_1	1	70	2794	no	2802	-
	2	74	21058	no	21071	-
Sample_2						
Sample_3	1	67	25800	no	25814	-
	2	69	7503	no	7531	-
	3	48	26443	yes	26523	M
	4	74	21058	no	21071	-
	5	75	28265	no	28274	N
Sample_4						
Sample_5						
Sample_6	1	48	26443	yes	26523	M
	2	74	21058	no	21071	-
Sample_8						
Sample_9	1	70	2794	no	2802	-
Sample_10						
Sample_11	1	72	22107	no	22155	-
	2	74	4555	no	4590	-
	3	74	21058	no	21071	-
	4	63	5785	no	5859	-
Sample_12						
Sample_14	1	67	4018	no	4044	-
	2	68	4580	no	4590	-
	3	75	26486	no	26523	M
	4	63	5785	no	5859	-
Sample_17						
Sample_18						
Sample_19						

“peak_leader” and “peak_TRS_start” point to the leader-TRS junctions in sheet "novel", “ACGAA

20_leader_se	TRS_seq	first_orf_aa
TTGTAGATCT	TGAACTTG	MKGLIKYLMRSALPIQLNSVQK*
AGATCTGTTC	CCTAAGACTA	MLQKKMTLKRVFSLTFVGLYNKS*
CTCTTGTAGA	AAACCCATTA	MMPTIFFAGILIVTTIVYLTIV*
CTTGTAGATC	ACAAACGTA/	MYNYC*
AAACCAACCA	TCTTCTAGAG	MADSNGTITVEELKKLLEQWNLVIGFLFLTWICLLQFAYANRNRFLYIIKLIFLW
AGATCTGTTC	CCTAAGACTA	MLQKKMTLKRVFSLTFVGLYNKS*
GATCTGTTCT	CAAACTAAA	MSDNGPQNQRNAPRITFGGPSDSTGSNQNGERSGARSKQRRPQGLPNNT/
AAACCAACCA	TCTTCTAGAG	MADSNGTITVEELKKLLEQWNLVIGFLFLTWICLLQFAYANRNRFLYIIKLIFLW
AGATCTGTTC	CCTAAGACTA	MLQKKMTLKRVFSLTFVGLYNKS*
TTGTAGATCT	TGAACTTG	MKGLIKYLMRSALPIQLNSVQK*
GTAGATCTGT	AACAGGGTA/	MVILKYILSTRLLI*
AGATCTGTTC	TGTAGCGTCA	MKLLLQCHLAM*
AGATCTGTTC	CCTAAGACTA	MLQKKMTLKRVFSLTFVGLYNKS*
CGATCTCTTG	TTCTAAAGAA	MFSTKKTVTQQP*
CTCTTGTAGA	AAACTTGTTA	MAIFIQILPLLLVTLTSLS*
TCTTGTAGAT	AACGATCTAA	MKLLLQCHLAM*
GATCTGTTCT	TATATTAGTT	MADSNGTITVEELKKLLEQWNLVIGFLFLTWICLLQFAYANRNRFLYIIKLIFLW
CGATCTCTTG	TTCTAAAGAA	MFSTKKTVTQQP*

C” indicates if there is a ACGAAC sequences in the “TRS_seq” (TRS sequences), “20_leader_seq”

LLWPVTLACFVLAAVYRINWITGGIAIAMACLVGLMWLSYFIASFRLFARTRSMWSFNPETNILLNVPLHGTIL

ASWFTALTQHGKEDLKFPRGQGVPINTNSSPDDQIGYYRRATRRIRGGDGKMKDLSPRWYFYYLGTGPEAGL

LLWPVTLACFVLAAVYRINWITGGIAIAMACLVGLMWLSYFIASFRLFARTRSMWSFNPETNILLNVPLHGTIL

LLWPVTLACFVLAAVYRINWITGGIAIAMACLVGLMWLSYFIASFRLFARTRSMWSFNPETNILLNVPLHGTIL

refers to the 20 sequences before the end of the leader, “AUG_postion” and “first_orf_aa” refer

TRPLLESELVIGAVILRGHLRIAGHHLGRCDIKDLPKEITVATSRTLSYYKLGASQRVAGDSGFAAYSRYRIGNYKL[illegible]

[illegible]YGANKDGIIWVATEGALNTPKDHIGTRNPANNAAIVLQLPQGTTLPKGFYAEGSRGGSQASSRSSSRSRNSS[illegible]

TRPLLESELVIGAVILRGHLRIAGHHLGRCDIKDLPKEITVATSRTLSYYKLGASQRVAGDSGFAAYSRYRIGNYKL[illegible]

TRPLLESELVIGAVILRGHLRIAGHHLGRCDIKDLPKEITVATSRTLSYYKLGASQRVAGDSGFAAYSRYRIGNYKL[illegible]

[illegible] to the first AUG position and translated orf of the sgmRNA, and “known_ATG” indicates if the fi[illegible]

NTDHSSSSDNIALLVQ*

RNSTPGSSRGTSPARMAGNGGDAALALLLLDRLNQLESKMSGKGQQQQGQTVTKKSAAEASKKPRQKRTATK

NTDHSSSSDNIALLVQ*

NTDHSSSSDNIALLVQ*

rst AUG position is the same as a known sgmRNA.

[illegible]AYNVTQAFGRRGPEQTQGNFGDQELIRQGTDYKHWPQIAQFAPSASAFFGMSRIGMEVTPSGTWLTYTGA

IKLDDKDPNFKDQVILLNKHIDAYKTFPPTEPKKDKKKKADETQALPQRQKKQQTVTLLPAADLDDFSKQLQQS

SMSSADSTQA*

Supplemental tables

Click here to access/download;Table;Supplemental_Material_4.xlsx

	subgenome	ref_leader_e	peak_leader_	ref_TRS_start	peak_TRS_start
	S	65	0	21552	0
	ORF3a	69	0	25385	0
	E	69	0	26237	0
	M	65	65	26469	26469
S1-Day1	ORF6	69	0	27041	0
	ORF7a	69	69	27388	27388
	ORF7b	65	0	27644	0
	ORF8	65	0	27884	0
	N	65	65	28256	28256
	ORF10	65	0	29530	0
	S	65	0	21552	0
	ORF3a	69	69	25385	25385
	E	69	0	26237	0
	M	65	64	26469	26468
S2-Day2	ORF6	69	0	27041	0
	ORF7a	69	69	27388	27388
	ORF7b	65	0	27644	0
	ORF8	65	65	27884	27884
	N	65	64	28256	28255
	ORF10	65	0	29530	0
	S	65	65	21552	21552
	ORF3a	69	69	25385	25385
	E	69	69	26237	26237
	M	65	64	26469	26468
S3-Day3	ORF6	69	69	27041	27041
	ORF7a	69	69	27388	27388
	ORF7b	65	65	27644	27644
	ORF8	65	65	27884	27884
	N	65	64	28256	28255
	ORF10	65	0	29530	0
	S	65	65	21552	21552
	ORF3a	69	69	25385	25385
	E	69	69	26237	26237
	M	65	65	26469	26469
S1-Day4	ORF6	69	69	27041	27041
	ORF7a	69	69	27388	27388
	ORF7b	65	65	27644	27644
	ORF8	65	65	27884	27884
	N	65	64	28256	28255
	ORF10	65	0	29530	0
	S	65	65	21552	21552
	ORF3a	69	69	25385	25385

	E	69	0	26237	0
	M	65	65	26469	26469
S2-Day5	ORF6	69	0	27041	0
	ORF7a	69	0	27388	0
	ORF7b	65	0	27644	0
	ORF8	65	0	27884	0
	N	65	65	28256	28256
	ORF10	65	0	29530	0
	S	65	65	21552	21552
	ORF3a	69	69	25385	25385
	E	69	0	26237	0
	M	65	65	26469	26469
S3-Day6	ORF6	69	69	27041	27041
	ORF7a	69	69	27388	27388
	ORF7b	65	65	27644	27644
	ORF8	65	65	27884	27884
	N	65	63	28256	28254
	ORF10	65	0	29530	0
	S	65	0	21552	0
	ORF3a	69	0	25385	0
	E	69	0	26237	0
	M	65	0	26469	0
S2-Day8	ORF6	69	0	27041	0
	ORF7a	69	69	27388	27388
	ORF7b	65	0	27644	0
	ORF8	65	0	27884	0
	N	65	65	28256	28256
	ORF10	65	0	29530	0
	S	65	0	21552	0
	ORF3a	69	0	25385	0
	E	69	0	26237	0
	M	65	0	26469	0
S3-Day9	ORF6	69	69	27041	27041
	ORF7a	69	0	27388	0
	ORF7b	65	0	27644	0
	ORF8	65	0	27884	0
	N	65	65	28256	28256
	ORF10	65	0	29530	0
	S	65	0	21552	0
	ORF3a	69	0	25385	0
	E	69	0	26237	0
	M	65	65	26469	26469
	ORF6	69	69	27041	27041
S2-Day11					

S2-Day11	ORF7a	69	0	27388	0
	ORF7b	65	65	27644	27644
	ORF8	65	0	27884	0
	N	65	64	28256	28255
	ORF10	65	0	29530	0
S3-Day12	S	65	0	21552	0
	ORF3a	69	0	25385	0
	E	69	0	26237	0
	M	65	65	26469	26469
	ORF6	69	0	27041	0
	ORF7a	69	0	27388	0
	ORF7b	65	0	27644	0
	ORF8	65	0	27884	0
	N	65	65	28256	28256
	ORF10	65	0	29530	0
S3-Day19	S	65	0	21552	0
	ORF3a	69	69	25385	25385
	E	69	0	26237	0
	M	65	0	26469	0
	ORF6	69	0	27041	0
	ORF7a	69	0	27388	0
	ORF7b	65	0	27644	0
	ORF8	65	0	27884	0
	N	65	0	28256	0
	ORF10	65	0	29530	0

The numbers in the bracket are (reads with left primers, reads with right primers, read
Normalized count=(Read count/Total number of read mapped on reference genome)*
Total number of read mapped on reference genome is 32903734, 33694953, 3340048
“ref_leader_end” and “peak_leader_end” point to the reference position of the end o

peak_count	peak_normalized_count	cluster_count
0	0	0
0	0	0
0	0	0
196(195,194,193,190)	5.96(5.93,5.90,5.87,5.77)	204(203,202,201,196)
0	0	0
1(1,0,0,0)	0.03(0.03,0.00,0.00,0.00)	1(1,0,0,0)
0	0	0
0	0	0
20(8,20,8,8)	0.61(0.24,0.61,0.24,0.24)	22(10,22,10,10)
0	0	0
0	0	0
1382(1368,0,0,30)	41.02(40.60,0.00,0.00,0.89)	1388(1374,0,0,30)
0	0	0
6874(6868,6701,6697,6852)	204.01(203.83,198.87,198.75,203.[illegible]	8414(8402,8188,8179,8342)
0	0	0
259(13,256,10,32)	7.69(0.39,7.60,0.30,0.95)	259(13,256,10,32)
0	0	0
12(12,0,0,0)	0.36(0.36,0.00,0.00,0.00)	12(12,0,0,0)
2463(2457,2436,2430,2438)	73.10(72.92,72.30,72.12,72.36)	3884(3874,3838,3828,3818)
0	0	0
5259(5236,3453,3442,3664)	157.45(156.76,103.38,103.05,109.[illegible]	5347(5323,3498,3486,3674)
2548(2490,0,0,42)	76.29(74.55,0.00,0.00,1.26)	2681(2620,0,0,46)
2824(2797,4,4,32)	84.55(83.74,0.12,0.12,0.96)	2825(2798,4,4,32)
9355(9343,8993,8984,9280)	280.09(279.73,269.25,268.98,277.[illegible]	10837(10820,10377,10367,10[illegible]
2893(2876,2560,2550,2612)	86.62(86.11,76.65,76.35,78.20)	2976(2959,2635,2625,2672)
61(50,15,4,4)	1.83(1.50,0.45,0.12,0.12)	62(51,15,4,4)
2(2,0,0,0)	0.06(0.06,0.00,0.00,0.00)	2(2,0,0,0)
183(183,0,0,4)	5.48(5.48,0.00,0.00,0.12)	183(183,0,0,4)
22082(22010,21900,21853,218[illegible]	661.13(658.97,655.68,654.27,654.[illegible]	23693(23612,23459,23404,232[illegible]
0	0	0
4751(4736,3166,3155,2084)	145.35(144.89,96.86,96.52,63.76)	6150(6127,4390,4373,2128)
1431(1414,0,0,26)	43.78(43.26,0.00,0.00,0.80)	1435(1418,0,0,26)
4766(4753,0,0,26)	145.81(145.41,0.00,0.00,0.80)	4768(4754,0,0,26)
2267(2264,2170,2170,2194)	69.36(69.26,66.39,66.39,67.12)	2347(2344,2247,2247,2262)
5600(5576,5275,5252,5312)	171.32(170.59,161.38,160.68,162.[illegible]	5638(5614,5311,5288,5334)
26(25,3,2,4)	0.80(0.76,0.09,0.06,0.12)	26(25,3,2,4)
2(2,0,0,2)	0.06(0.06,0.00,0.00,0.06)	2(2,0,0,2)
140(135,0,0,0)	4.28(4.13,0.00,0.00,0.00)	140(135,0,0,0)
31082(31046,30667,30631,307[illegible]	950.90(949.80,938.21,937.11,940.[illegible]	32535(32488,32095,32048,31[illegible]
0	0	0
1(1,0,0,0)	0.06(0.06,0.00,0.00,0.00)	1(1,0,0,0)
94(91,0,0,0)	5.51(5.34,0.00,0.00,0.00)	95(92,0,0,0)

0	0	0
2(2,2,2,2)	0.12(0.12,0.12,0.12,0.12)	2(2,2,2,2)
0	0	0
0	0	0
0	0	0
0	0	0
12(2,12,2,2)	0.70(0.12,0.70,0.12,0.12)	16(4,16,4,6)
0	0	0
8903(8893,7853,7843,8088)	270.90(270.60,238.95,238.65,246.[illegible]	8960(8950,7896,7886,8092)
2718(2687,0,0,42)	82.70(81.76,0.00,0.00,1.28)	2747(2695,0,0,42)
0	0	0
340(340,328,328,324)	10.35(10.35,9.98,9.98,9.86)	428(427,411,410,398)
7227(7210,6907,6890,6968)	219.90(219.39,210.17,209.65,212.[illegible]	7257(7240,6937,6920,6978)
43(38,12,7,18)	1.31(1.16,0.37,0.21,0.55)	43(38,12,7,18)
1(1,0,0,0)	0.03(0.03,0.00,0.00,0.00)	1(1,0,0,0)
41(41,0,0,0)	1.25(1.25,0.00,0.00,0.00)	41(41,0,0,0)
36127(36101,35933,35908,348[illegible]	1099.28(1098.49,1093.37,1092.61,[illegible]	64772(64692,64000,63922,617[illegible]
0	0	0
0	0	0
0	0	0
0	0	0
0	0	0
0	0	0
10(9,1,0,2)	0.74(0.67,0.07,0.00,0.15)	10(9,1,0,2)
0	0	0
0	0	0
4(2,4,2,2)	0.30(0.15,0.30,0.15,0.15)	4(2,4,2,2)
0	0	0
0	0	0
0	0	0
0	0	0
0	0	0
8006(7996,7805,7795,7796)	501.90(501.27,489.30,488.67,488.[illegible]	8025(8015,7824,7814,7796)
0	0	0
0	0	0
0	0	0
4(2,4,2,2)	0.25(0.13,0.25,0.13,0.13)	4(2,4,2,2)
0	0	0
0	0	0
0	0	0
0	0	0
2(2,2,2,2)	0.20(0.20,0.20,0.20,0.20)	2(2,2,2,2)
41847(41781,41584,41518,410[illegible]	4230.09(4223.42,4203.50,4196.83,[illegible]	41993(41927,41730,41664,41[illegible]

0	0	0
1(1,0,0,0)	0.10(0.10,0.00,0.00,0.00)	1(1,0,0,0)
0	0	0
210234(210002,209510,20928[illegible]	21251.43(21227.97,21178.24,2115[illegible]	215169(214896,214384,21411[illegible]
0	0	0
0	0	0
0	0	0
0	0	0
1(1,0,0,0)	0.07(0.07,0.00,0.00,0.00)	1(1,0,0,0)
0	0	0
0	0	0
0	0	0
0	0	0
2(2,2,2,2)	0.14(0.14,0.14,0.14,0.14)	2(2,2,2,2)
0	0	0
0	0	0
1(0,0,0,0)	0.06(0.00,0.00,0.00,0.00)	1(0,0,0,0)
0	0	0
0	0	0
0	0	0
0	0	0
0	0	0
0	0	0
0	0	0
0	0	0

ls with both primers, same junction on paired reads with at least a primer).

[illegible]1000000.

5, 32686824, 17052923, 32864351, 13447115, 15951471, 9892701, 13862861, 12570298 and 156[illegible]

f leader and the position of the end of leader identified in the most common reads (peak count) or

cluster_normalized_count
0
0
0
6.20(6.17,6.14,6.11,5.96)
0
0.03(0.03,0.00,0.00,0.00)
0
0
0.67(0.30,0.67,0.30,0.30)
0
0
41.19(40.78,0.00,0.00,0.89)
0
249.71(249.35,243.00,242.74,247.57)
0
7.69(0.39,7.60,0.30,0.95)
0
0.36(0.36,0.00,0.00,0.00)
115.27(114.97,113.90,113.61,113.31)
0
160.09(159.37,104.73,104.37,110.00)
80.27(78.44,0.00,0.00,1.38)
84.58(83.77,0.12,0.12,0.96)
324.46(323.95,310.68,310.38,318.44)
89.10(88.59,78.89,78.59,80.00)
1.86(1.53,0.45,0.12,0.12)
0.06(0.06,0.00,0.00,0.00)
5.48(5.48,0.00,0.00,0.12)
709.36(706.94,702.36,700.71,695.68)
0
188.15(187.45,134.30,133.78,65.10)
43.90(43.38,0.00,0.00,0.80)
145.87(145.44,0.00,0.00,0.80)
71.80(71.71,68.74,68.74,69.20)
172.49(171.75,162.48,161.78,163.19)
0.80(0.76,0.09,0.06,0.12)
0.06(0.06,0.00,0.00,0.06)
4.28(4.13,0.00,0.00,0.00)
995.36(993.92,981.89,980.46,976.17)
0
0.06(0.06,0.00,0.00,0.00)
5.57(5.39,0.00,0.00,0.00)

0
0.12(0.12,0.12,0.12,0.12)
0
0
0
0
0.94(0.23,0.94,0.23,0.35)
0
272.64(272.33,240.26,239.96,246.22)
83.59(82.00,0.00,0.00,1.28)
0
13.02(12.99,12.51,12.48,12.11)
220.82(220.30,211.08,210.56,212.33)
1.31(1.16,0.37,0.21,0.55)
0.03(0.03,0.00,0.00,0.00)
1.25(1.25,0.00,0.00,0.00)
1970.89(1968.46,1947.40,1945.03,1880.15)
0
0
0
0
0
0
0.74(0.67,0.07,0.00,0.15)
0
0
0.30(0.15,0.30,0.15,0.15)
0
0
0
0
0
503.09(502.46,490.49,489.86,488.73)
0
0
0
0.25(0.13,0.25,0.13,0.13)
0
0
0
0
0.20(0.20,0.20,0.20,0.20)
4244.85(4238.18,4218.26,4211.59,4149.73)

0
0.10(0.10,0.00,0.00,0.00)
0
21750.28(21722.68,21670.93,21644.14,21514.85)
0
0
0
0
0.07(0.07,0.00,0.00,0.00)
0
0
0
0
0.14(0.14,0.14,0.14,0.14)
0
0
0.06(0.00,0.00,0.00,0.00)
0
0
0
0
0
0
0
0

53045, excluding the mapped reads unpaired, not primary alignment and supplementary alignment.
the reference genome, and “ref_TRS_start” and “peak_TRS_start” refer to the reference position of t

:he start of TRS and the position of the start of TRS identified in the most common reads (peak cc

ɔunt) on the reference genome.

	subgenome	ref_leader_e	peak_leader_	ref_TRS_start	peak_TRS_sta	peak_count
	S	65	65	21552	21552	2928(2926,21
	ORF3a	69	69	25385	25385	578(573,0,0,1
	E	69	69	26237	26237	2276(2273,2,
	M	65	65	26469	26469	607(607,555,
S1-Day1	ORF6	69	0	27041	0	0
	ORF7a	69	0	27388	0	0
	ORF7b	65	0	27644	0	0
	ORF8	65	0	27884	0	0
	N	65	64	28256	28255	3772(3770,37
	ORF10	65	0	29530	0	0
	S	65	65	21552	21552	5529(5523,45
	ORF3a	69	69	25385	25385	1176(1168,0,
	E	69	69	26237	26237	2138(2133,4,
	M	65	64	26469	26468	4176(4172,39
S2-Day2	ORF6	69	69	27041	27041	2267(2265,21
	ORF7a	69	69	27388	27388	12(9,5,2,6)
	ORF7b	65	0	27644	0	0
	ORF8	65	0	27884	0	0
	N	65	64	28256	28255	29251(29214
	ORF10	65	0	29530	0	0
	S	65	65	21552	21552	1191(1189,90
	ORF3a	69	69	25385	25385	326(321,0,0,1
	E	69	0	26237	0	0
	M	65	65	26469	26469	1170(1170,10
S3-Day3	ORF6	69	69	27041	27041	647(647,592,
	ORF7a	69	69	27388	27388	7(6,1,0,0)
	ORF7b	65	0	27644	0	0
	ORF8	65	65	27884	27884	15(15,0,0,0)
	N	65	64	28256	28255	4046(4042,40
	ORF10	65	0	29530	0	0
	S	65	65	21552	21552	2958(2951,25
	ORF3a	69	69	25385	25385	100(98,0,0,2)
	E	69	0	26237	0	0
	M	65	65	26469	26469	5063(5061,49
S1-Day4	ORF6	69	69	27041	27041	4627(4620,44
	ORF7a	69	69	27388	27388	9(8,1,0,0)
	ORF7b	65	65	27644	27644	1(1,0,0,0)
	ORF8	65	65	27884	27884	8(8,0,0,0)
	N	65	64	28256	28255	30869(30831
	ORF10	65	0	29530	0	0
	S	65	65	21552	21552	6764(6763,61
	ORF3a	69	0	25385	0	0

	E	69	0	26237	0	0
	M	65	65	26469	26469	2376(2376,2[illegible]
S2-Day5	ORF6	69	0	27041	0	0
	ORF7a	69	0	27388	0	0
	ORF7b	65	65	27644	27644	2(2,0,0,2)
	ORF8	65	0	27884	0	0
	N	65	68	28256	28263	36092(36037[illegible]
	ORF10	65	0	29530	0	0
	S	65	65	21552	21552	2400(2399,2[illegible]
	ORF3a	69	69	25385	25385	1122(1108,0,[illegible]
	E	69	69	26237	26237	2721(2716,6,[illegible]
	M	65	64	26469	26468	8639(8632,8[illegible]
S3-Day6	ORF6	69	69	27041	27041	4390(4378,4[illegible]
	ORF7a	69	69	27388	27388	29(17,12,0,1[illegible]
	ORF7b	65	0	27644	0	0
	ORF8	65	65	27884	27884	29(29,0,0,0)
	N	65	65	28256	28256	6048(6022,5[illegible]
	ORF10	65	0	29530	0	0
	S	65	0	21552	0	0
	ORF3a	69	69	25385	25385	286(283,0,0,[illegible]
	E	69	0	26237	0	0
	M	65	0	26469	0	0
S2-Day8	ORF6	69	0	27041	0	0
	ORF7a	69	69	27388	27388	12(10,2,0,8)
	ORF7b	65	0	27644	0	0
	ORF8	65	0	27884	0	0
	N	65	65	28256	28256	68(7,68,7,26)
	ORF10	65	0	29530	0	0
	S	65	0	21552	0	0
	ORF3a	69	0	25385	0	0
	E	69	0	26237	0	0
	M	65	65	26469	26469	92(91,90,89,[illegible]
S3-Day9	ORF6	69	0	27041	0	0
	ORF7a	69	0	27388	0	0
	ORF7b	65	0	27644	0	0
	ORF8	65	0	27884	0	0
	N	65	65	28256	28256	5(4,5,4,4)
	ORF10	65	0	29530	0	0
	S	65	0	21552	0	0
	ORF3a	69	69	25385	25385	1(0,0,0,0)
	E	69	0	26237	0	0
	M	65	65	26469	26469	2(2,2,2,2)
	ORF6	69	0	27041	0	0
S2-Day11						

S2-Day11	ORF7a	69	0	27388	0	0
	ORF7b	65	0	27644	0	0
	ORF8	65	0	27884	0	0
	N	65	0	28256	0	0
	ORF10	65	0	29530	0	0
	S	65	0	21552	0	0
	ORF3a	69	0	25385	0	0
	E	69	0	26237	0	0
	M	65	0	26469	0	0
S3-Day12	ORF6	69	0	27041	0	0
	ORF7a	69	0	27388	0	0
	ORF7b	65	0	27644	0	0
	ORF8	65	0	27884	0	0
	N	65	65	28256	28256	1(0,1,0,0)
	ORF10	65	0	29530	0	0
	S	65	0	21552	0	0
	ORF3a	69	69	25385	25385	1(0,0,0,0)
	E	69	0	26237	0	0
	M	65	0	26469	0	0
S3-Day15	ORF6	69	69	27041	27041	2(2,2,2,2)
	ORF7a	69	69	27388	27388	1(0,1,0,0)
	ORF7b	65	0	27644	0	0
	ORF8	65	0	27884	0	0
	N	65	65	28256	28256	2(2,2,2,0)
	ORF10	65	0	29530	0	0

The numbers in the bracket are (reads with left primers, reads with right primers, reads with bot
Normalized count=(Read count/Total number of read mapped on reference genome)*1000000.
Total number of read mapped on reference genome is 40751196, 34608693, 32168622, 322809
“ref_leader_end” and “peak_leader_end” point to the reference position of the end of leader ar

peak_normal	cluster_count	cluster_normalized_count
71.85(71.80,5	2948(2946,22	72.34(72.29,54.23,54.18,59.34)
14.18(14.06,(	581(576,0,0,1	14.26(14.13,0.00,0.00,0.29)
55.85(55.78,(	2276(2273,2,	55.85(55.78,0.05,0.05,0.25)
14.90(14.90,1	618(618,565,	15.17(15.17,13.86,13.86,14.48)
0	0	0
0	0	0
0	0	0
0	0	0
92.56(92.51,9	4254(4250,41	104.39(104.29,102.43,102.33,103.16)
0	0	0
159.76(159.5	5568(5562,45	160.88(160.71,132.08,131.93,140.25)
33.98(33.75,(	1180(1172,0,	34.10(33.86,0.00,0.00,1.10)
61.78(61.63,(	2138(2133,4,	61.78(61.63,0.12,0.12,0.69)
120.66(120.5	7998(7987,75	231.10(230.78,219.42,219.28,226.13)
65.50(65.45,6	2278(2276,21	65.82(65.76,62.44,62.44,62.18)
0.35(0.26,0.1	12(9,5,2,6)	0.35(0.26,0.14,0.06,0.17)
0	0	0
0	0	0
845.19(844.1	31113(31065	898.99(897.61,884.63,883.25,887.18)
0	0	0
37.02(36.96,2	1199(1197,9(	37.27(37.21,28.10,28.04,30.15)
10.13(9.98,0.	327(322,0,0,1	10.17(10.01,0.00,0.00,0.31)
0	0	0
36.37(36.37,3	1180(1180,1(	36.68(36.68,34.04,34.04,36.00)
20.11(20.11,1	652(652,596,	20.27(20.27,18.53,18.53,18.65)
0.22(0.19,0.0	7(6,1,0,0)	0.22(0.19,0.03,0.00,0.00)
0	0	0
0.47(0.47,0.0	15(15,0,0,0)	0.47(0.47,0.00,0.00,0.00)
125.77(125.6	4226(4215,41	131.37(131.03,129.82,129.51,129.38)
0	0	0
91.63(91.42,7	2976(2969,25	92.19(91.97,79.99,79.80,82.77)
3.10(3.04,0.0	100(98,0,0,2)	3.10(3.04,0.00,0.00,0.06)
0	0	0
156.84(156.7	5129(5127,49	158.89(158.82,153.90,153.90,154.95)
143.34(143.1	4655(4648,44	144.20(143.99,137.85,137.64,139.65)
0.28(0.25,0.0	9(8,1,0,0)	0.28(0.25,0.03,0.00,0.00)
0.03(0.03,0.0	1(1,0,0,0)	0.03(0.03,0.00,0.00,0.00)
0.25(0.25,0.0	8(8,0,0,0)	0.25(0.25,0.00,0.00,0.00)
956.26(955.0	31777(31713	984.39(982.41,963.97,962.02,971.60)
0	0	0
236.54(236.5	6806(6805,62	238.01(237.98,217.66,217.62,213.53)
0	0	0

0	0	0
83.09(83.09,8	2516(2515,24	87.99(87.95,85.71,85.68,84.91)
0	0	0
0	0	0
0.07(0.07,0.0	2(2,0,0,2)	0.07(0.07,0.00,0.00,0.07)
0	0	0
1262.17(126(	45257(45180	1582.68(1579.99,1568.03,1565.37,1531.59)
0	0	0
79.85(79.82,6	2419(2418,2(	80.48(80.45,68.84,68.80,70.07)
37.33(36.86,(	1123(1109,0,	37.36(36.90,0.00,0.00,0.60)
90.53(90.36,(	2721(2716,6,	90.53(90.36,0.20,0.20,0.47)
287.43(287.1	8851(8844,8(	294.48(294.25,287.96,287.79,292.18)
146.06(145.6	4411(4399,41	146.76(146.36,138.44,138.07,138.67)
0.96(0.57,0.4	29(17,12,0,14	0.96(0.57,0.40,0.00,0.47)
0	0	0
0.96(0.96,0.0	29(29,0,0,0)	0.96(0.96,0.00,0.00,0.00)
201.22(200.3	7381(7353,72	245.57(244.64,240.41,239.52,180.46)
0	0	0
0	0	0
9.70(9.60,0.0	288(285,0,0,2	9.77(9.67,0.00,0.00,0.07)
0	0	0
0	0	0
0	0	0
0.41(0.34,0.0	12(10,2,0,8)	0.41(0.34,0.07,0.00,0.27)
0	0	0
0	0	0
2.31(0.24,2.3	72(8,72,8,26)	2.44(0.27,2.44,0.27,0.88)
0	0	0
0	0	0
0	0	0
0	0	0
4.99(4.94,4.8	109(108,107,	5.92(5.86,5.81,5.75,5.64)
0	0	0
0	0	0
0	0	0
0	0	0
0.27(0.22,0.2	5(4,5,4,4)	0.27(0.22,0.27,0.22,0.22)
0	0	0
0	0	0
0.06(0.00,0.0	1(0,0,0,0)	0.06(0.00,0.00,0.00,0.00)
0	0	0
0.11(0.11,0.1	2(2,2,2,2)	0.11(0.11,0.11,0.11,0.11)
0	0	0

0	0	0
0	0	0
0	0	0
0	0	0
0	0	0
0	0	0
0	0	0
0	0	0
0	0	0
0	0	0
0	0	0
0	0	0
0	0	0
0.09(0.00,0.0	1(0,1,0,0)	0.09(0.00,0.09,0.00,0.00)
0	0	0
0	0	0
0.08(0.00,0.0	1(0,0,0,0)	0.08(0.00,0.00,0.00,0.00)
0	0	0
0	0	0
0.16(0.16,0.1	2(2,2,2,2)	0.16(0.16,0.16,0.16,0.16)
0.08(0.00,0.0	1(0,1,0,0)	0.08(0.00,0.08,0.00,0.00)
0	0	0
0	0	0
0.16(0.16,0.1	2(2,2,2,0)	0.16(0.16,0.16,0.16,0.00)
0	0	0

:h primers, same junction on paired reads with at least a primer).

32, 28595139, 30056513, 29481101, 18427015, 17677974, 11329365, 10742292 and 127866
ıd the position of the end of leader identified in the most common reads (peak count) on the

;5, excluding the mapped reads unpaired, not primary alignment and supplementary alignment.
reference genome, and “ref_TRS_start” and “peak_TRS_start” refer to the reference position of

the start of TRS and the position of the start of TRS identified in the most common reads (peak c

ount) on the reference genome.

	subgenome	peak_leader_	peak_TRS_st	ACGAAC	ATG_postion
	M	65	26469	yes	26523
S1-Day1	ORF7a	69	27388	yes	27394
	N	65	28256	yes	28274
	ORF3a	69	25385	yes	25393
	M	64	26468	yes	26523
S2-Day2	ORF7a	69	27388	yes	27394
	ORF8	65	27884	yes	27894
	N	64	28255	yes	28274
	S	65	21552	yes	21563
	ORF3a	69	25385	yes	25393
	E	69	26237	yes	26245
	M	64	26468	yes	26523
S3-Day3	ORF6	69	27041	yes	27202
	ORF7a	69	27388	yes	27394
	ORF7b	65	27644	no	27756
	ORF8	65	27884	yes	27894
	N	64	28255	yes	28274
	S	65	21552	yes	21563
	ORF3a	69	25385	yes	25393
	E	69	26237	yes	26245
	M	65	26469	yes	26523
S1-Day4	ORF6	69	27041	yes	27202
	ORF7a	69	27388	yes	27394
	ORF7b	65	27644	no	27756
	ORF8	65	27884	yes	27894
	N	64	28255	yes	28274
	S	65	21552	yes	21563
S2-Day5	ORF3a	69	25385	yes	25393
	M	65	26469	yes	26523
	N	65	28256	yes	28274
	S	65	21552	yes	21563
	ORF3a	69	25385	yes	25393
	M	65	26469	yes	26523
S3-Day6	ORF6	69	27041	yes	27202
	ORF7a	69	27388	yes	27394
	ORF7b	65	27644	no	27756
	ORF8	65	27884	yes	27894
	N	63	28254	yes	28274
S2-Day8	ORF7a	69	27388	yes	27394
	N	65	28256	yes	28274
S3-Day9	ORF6	69	27041	yes	27202
	N	65	28256	yes	28274

S2-Day11	M	65	26469	yes	26523
	ORF6	69	27041	yes	27202
	ORF7b	65	27644	no	27756
	N	64	28255	yes	28274
S3-Day12	M	65	26469	yes	26523
	N	65	28256	yes	28274
S3-Day19	ORF3a	69	25385	yes	25393

“peak_leader” and “peak_TRS_start” point to the leader-TRS junctions in sheet "known", “ACGAA

20_leader_seq	TRS_seq	first_orf_aa
ATCTCTTGTAGATCTGTTCT	CTAAACGAA	MADSNGTITVEELKKLLEQWNLVIGFLFLTW
CTTGTAGATCTGTTCTCTAA	ACGAAC	MKIILFLALITLATCELYHYQECVRGTTVLLKE
ATCTCTTGTAGATCTGTTCT	CTAAACGAA	MSDNGPQNQRNAPRITFGGPSDSTGSNQN
CTTGTAGATCTGTTCTCTAA	ACGAACTT	MDLFMRIFTIGTVTLKQGEIKDATPSDFVRA
GATCTCTTGTAGATCTGTTC	TCTAAACGA	MADSNGTITVEELKKLLEQWNLVIGFLFLTW
CTTGTAGATCTGTTCTCTAA	ACGAAC	MKIILFLALITLATCELYHYQECVRGTTVLLKE
ATCTCTTGTAGATCTGTTCT	CTAAACGAA	MKFLVFLGIITTVAAFHQECSLQSCTQHQPY
GATCTCTTGTAGATCTGTTC	TCTAAACGA	MSDNGPQNQRNAPRITFGGPSDSTGSNQN
ATCTCTTGTAGATCTGTTCT	CTAAACGAA	MFVFLVLLPLVSSQCVNLTTRTQLPPAYTNS
CTTGTAGATCTGTTCTCTAA	ACGAACTT	MDLFMRIFTIGTVTLKQGEIKDATPSDFVRA
CTTGTAGATCTGTTCTCTAA	ACGAACTT	MYSFVSEETGTLIVNSVLLFLAFVVFLLVTLAI
GATCTCTTGTAGATCTGTTC	TCTAAACGA	MADSNGTITVEELKKLLEQWNLVIGFLFLTW
CTTGTAGATCTGTTCTCTAA	ACGAACGCT	MFHLVDFQVTIAEILLIIMRTFKVSIWNLDYII
CTTGTAGATCTGTTCTCTAA	ACGAAC	MKIILFLALITLATCELYHYQECVRGTTVLLKE
ATCTCTTGTAGATCTGTTCT	CTAAACTGTT	MIELSLIDFYLCFLAFLLFLVLIMLIIFWFSLELQ
ATCTCTTGTAGATCTGTTCT	CTAAACGAA	MKFLVFLGIITTVAAFHQECSLQSCTQHQPY
GATCTCTTGTAGATCTGTTC	TCTAAACGA	MSDNGPQNQRNAPRITFGGPSDSTGSNQN
ATCTCTTGTAGATCTGTTCT	CTAAACGAA	MFVFLVLLPLVSSQCVNLTTRTQLPPAYTNS
CTTGTAGATCTGTTCTCTAA	ACGAACTT	MDLFMRIFTIGTVTLKQGEIKDATPSDFVRA
CTTGTAGATCTGTTCTCTAA	ACGAACTT	MYSFVSEETGTLIVNSVLLFLAFVVFLLVTLAI
ATCTCTTGTAGATCTGTTCT	CTAAACGAA	MADSNGTITVEELKKLLEQWNLVIGFLFLTW
CTTGTAGATCTGTTCTCTAA	ACGAACGCT	MFHLVDFQVTIAEILLIIMRTFKVSIWNLDYII
CTTGTAGATCTGTTCTCTAA	ACGAAC	MKIILFLALITLATCELYHYQECVRGTTVLLKE
ATCTCTTGTAGATCTGTTCT	CTAAACTGTT	MIELSLIDFYLCFLAFLLFLVLIMLIIFWFSLELQ
ATCTCTTGTAGATCTGTTCT	CTAAACGAA	MKFLVFLGIITTVAAFHQECSLQSCTQHQPY
GATCTCTTGTAGATCTGTTC	TCTAAACGA	MSDNGPQNQRNAPRITFGGPSDSTGSNQN
ATCTCTTGTAGATCTGTTCT	CTAAACGAA	MFVFLVLLPLVSSQCVNLTTRTQLPPAYTNS
CTTGTAGATCTGTTCTCTAA	ACGAACTT	MDLFMRIFTIGTVTLKQGEIKDATPSDFVRA
ATCTCTTGTAGATCTGTTCT	CTAAACGAA	MADSNGTITVEELKKLLEQWNLVIGFLFLTW
ATCTCTTGTAGATCTGTTCT	CTAAACGAA	MSDNGPQNQRNAPRITFGGPSDSTGSNQN
ATCTCTTGTAGATCTGTTCT	CTAAACGAA	MFVFLVLLPLVSSQCVNLTTRTQLPPAYTNS
CTTGTAGATCTGTTCTCTAA	ACGAACTT	MDLFMRIFTIGTVTLKQGEIKDATPSDFVRA
ATCTCTTGTAGATCTGTTCT	CTAAACGAA	MADSNGTITVEELKKLLEQWNLVIGFLFLTW
CTTGTAGATCTGTTCTCTAA	ACGAACGCT	MFHLVDFQVTIAEILLIIMRTFKVSIWNLDYII
CTTGTAGATCTGTTCTCTAA	ACGAAC	MKIILFLALITLATCELYHYQECVRGTTVLLKE
ATCTCTTGTAGATCTGTTCT	CTAAACTGTT	MIELSLIDFYLCFLAFLLFLVLIMLIIFWFSLELQ
ATCTCTTGTAGATCTGTTCT	CTAAACGAA	MKFLVFLGIITTVAAFHQECSLQSCTQHQPY
CGATCTCTTGTAGATCTGTT	ATCTAAACG	MSDNGPQNQRNAPRITFGGPSDSTGSNQN
CTTGTAGATCTGTTCTCTAA	ACGAAC	MKIILFLALITLATCELYHYQECVRGTTVLLKE
ATCTCTTGTAGATCTGTTCT	CTAAACGAA	MSDNGPQNQRNAPRITFGGPSDSTGSNQN
CTTGTAGATCTGTTCTCTAA	ACGAACGCT	MFHLVDFQVTIAEILLIIMRTFKVSIWNLDYII
ATCTCTTGTAGATCTGTTCT	CTAAACGAA	MSDNGPQNQRNAPRITFGGPSDSTGSNQN

ATCTCTTGTAGATCTGTTCT	CTAAACGAA(	MADSNGTITVEELKKLLEQWNLVIGFLFLTW
CTTGTAGATCTGTTCTCTAA	ACGAACGCTI	MFHLVDFQVTIAEILLIIMRTFKVSIWNLDYIII
ATCTCTTGTAGATCTGTTCT	CTAAACTGTT	MIELSLIDFYLCFLAFLLFLVLIMLIIFWFSLELQ
GATCTCTTGTAGATCTGTTC	TCTAAACGAA	MSDNGPQNQRNAPRITFGGPSDSTGSNQN
ATCTCTTGTAGATCTGTTCT	CTAAACGAA(	MADSNGTITVEELKKLLEQWNLVIGFLFLTW
ATCTCTTGTAGATCTGTTCT	CTAAACGAA(	MSDNGPQNQRNAPRITFGGPSDSTGSNQN
CTTGTAGATCTGTTCTCTAA	ACGAACTT	MDLFMRIFTIGTVTLKQGEIKDATPSDFVRAT

AC" indicates if there is a ACGAAC sequence in the "TRS_seq" (TRS sequences), "20_leader_sec

ICLLQFAYANRNRFLYIIKLIFLWLLWPVTLACFVLAAVYRINWITGGIAIAMACLVGLMWLSYFIASFRLFARTF
PCSSGTYEGNSPFHPLADNKFALTCFSTQFAFACPDGVKHVYQLRARSVSPKLFIRQEEVQELYSPIFLIVAAIVF
IGERSGARSKQRRPQGLPNNTASWFTALTQHGKEDLKFPRGQGVPINTNSSPDDQIGYYRRATRRIRGGDGK
TATIPIQASLPFGWLIVGVALLAVFQSASKIITLKKRWQLALSKGVHFVCNLLLLFVTVYSHLLLVAAGLEAPFLYL
ICLLQFAYANRNRFLYIIKLIFLWLLWPVTLACFVLAAVYRINWITGGIAIAMACLVGLMWLSYFIASFRLFARTF
PCSSGTYEGNSPFHPLADNKFALTCFSTQFAFACPDGVKHVYQLRARSVSPKLFIRQEEVQELYSPIFLIVAAIVF
VVDDPCPIHFYSKWYIRVGARKSAPLIELCVDEAGSKSPIQYIDIGNYTVSCLPFTINCQEPKLGSLVVRCSFYEDF
IGERSGARSKQRRPQGLPNNTASWFTALTQHGKEDLKFPRGQGVPINTNSSPDDQIGYYRRATRRIRGGDGK
FTRGVYYPDKVFRSSVLHSTQDLFLPFFSNVTWFHAIHVSGTNGTKRFDNPVLPFNDGVYFASTEKSNIIRGWI
TATIPIQASLPFGWLIVGVALLAVFQSASKIITLKKRWQLALSKGVHFVCNLLLLFVTVYSHLLLVAAGLEAPFLYL
LTALRLCAYCCNIVNVSLVKPSFYVYSRVKNLNSSRVPDLLV*
ICLLQFAYANRNRFLYIIKLIFLWLLWPVTLACFVLAAVYRINWITGGIAIAMACLVGLMWLSYFIASFRLFARTF
NLIIKNLSKSLTENKYSQLDEEQPMEID*
PCSSGTYEGNSPFHPLADNKFALTCFSTQFAFACPDGVKHVYQLRARSVSPKLFIRQEEVQELYSPIFLIVAAIVF
DHNETCHA*
VVDDPCPIHFYSKWYIRVGARKSAPLIELCVDEAGSKSPIQYIDIGNYTVSCLPFTINCQEPKLGSLVVRCSFYEDF
IGERSGARSKQRRPQGLPNNTASWFTALTQHGKEDLKFPRGQGVPINTNSSPDDQIGYYRRATRRIRGGDGK
FTRGVYYPDKVFRSSVLHSTQDLFLPFFSNVTWFHAIHVSGTNGTKRFDNPVLPFNDGVYFASTEKSNIIRGWI
TATIPIQASLPFGWLIVGVALLAVFQSASKIITLKKRWQLALSKGVHFVCNLLLLFVTVYSHLLLVAAGLEAPFLYL
LTALRLCAYCCNIVNVSLVKPSFYVYSRVKNLNSSRVPDLLV*
ICLLQFAYANRNRFLYIIKLIFLWLLWPVTLACFVLAAVYRINWITGGIAIAMACLVGLMWLSYFIASFRLFARTF
NLIIKNLSKSLTENKYSQLDEEQPMEID*
PCSSGTYEGNSPFHPLADNKFALTCFSTQFAFACPDGVKHVYQLRARSVSPKLFIRQEEVQELYSPIFLIVAAIVF
DHNETCHA*
VVDDPCPIHFYSKWYIRVGARKSAPLIELCVDEAGSKSPIQYIDIGNYTVSCLPFTINCQEPKLGSLVVRCSFYEDF
IGERSGARSKQRRPQGLPNNTASWFTALTQHGKEDLKFPRGQGVPINTNSSPDDQIGYYRRATRRIRGGDGK
FTRGVYYPDKVFRSSVLHSTQDLFLPFFSNVTWFHAIHVSGTNGTKRFDNPVLPFNDGVYFASTEKSNIIRGWI
TATIPIQASLPFGWLIVGVALLAVFQSASKIITLKKRWQLALSKGVHFVCNLLLLFVTVYSHLLLVAAGLEAPFLYL
ICLLQFAYANRNRFLYIIKLIFLWLLWPVTLACFVLAAVYRINWITGGIAIAMACLVGLMWLSYFIASFRLFARTF
IGERSGARSKQRRPQGLPNNTASWFTALTQHGKEDLKFPRGQGVPINTNSSPDDQIGYYRRATRRIRGGDGK
FTRGVYYPDKVFRSSVLHSTQDLFLPFFSNVTWFHAIHVSGTNGTKRFDNPVLPFNDGVYFASTEKSNIIRGWI
TATIPIQASLPFGWLIVGVALLAVFQSASKIITLKKRWQLALSKGVHFVCNLLLLFVTVYSHLLLVAAGLEAPFLYL
ICLLQFAYANRNRFLYIIKLIFLWLLWPVTLACFVLAAVYRINWITGGIAIAMACLVGLMWLSYFIASFRLFARTF
NLIIKNLSKSLTENKYSQLDEEQPMEID*
PCSSGTYEGNSPFHPLADNKFALTCFSTQFAFACPDGVKHVYQLRARSVSPKLFIRQEEVQELYSPIFLIVAAIVF
DHNETCHA*
VVDDPCPIHFYSKWYIRVGARKSAPLIELCVDEAGSKSPIQYIDIGNYTVSCLPFTINCQEPKLGSLVVRCSFYEDF
IGERSGARSKQRRPQGLPNNTASWFTALTQHGKEDLKFPRGQGVPINTNSSPDDQIGYYRRATRRIRGGDGK
PCSSGTYEGNSPFHPLADNKFALTCFSTQFAFACPDGVKHVYQLRARSVSPKLFIRQEEVQELYSPIFLIVAAIVF
IGERSGARSKQRRPQGLPNNTASWFTALTQHGKEDLKFPRGQGVPINTNSSPDDQIGYYRRATRRIRGGDGK
NLIIKNLSKSLTENKYSQLDEEQPMEID*
IGERSGARSKQRRPQGLPNNTASWFTALTQHGKEDLKFPRGQGVPINTNSSPDDQIGYYRRATRRIRGGDGK

ICLLQFAYANRNRFLYIIKLIFLWLLWPVTLACFVLAAVYRINWITGGIAIAMACLVGLMWLSYFIASFRLFARTF

NLIIKNLSKSLTENKYSQLDEEQPMEID*

DHNETCHA*

IGERSGARSKQRRPQGLPNNTASWFTALTQHGKEDLKFPRGQGVPINTNSSPDDQIGYYRRATRRIRGGDGK

ICLLQFAYANRNRFLYIIKLIFLWLLWPVTLACFVLAAVYRINWITGGIAIAMACLVGLMWLSYFIASFRLFARTF

IGERSGARSKQRRPQGLPNNTASWFTALTQHGKEDLKFPRGQGVPINTNSSPDDQIGYYRRATRRIRGGDGK

TATIPIQASLPFGWLIVGVALLAVFQSASKIITLKKRWQLALSKGVHFVCNLLLLFVTVYSHLLLVAAGLEAPFLYL

q" refers to the 20 nucleotides before the end of leader, and "AUG_postion" and "first_orf_aa"

RSMWSFNPETNILLNVPLHGTILTRPLLESELVIGAVILRGHLRIAGHHLGRCDIKDLPKEITVATSRTLSYYKLGAS
ITLCFTLKRKTE*
MKDLSPRWYFYYLGTGPEAGLPYGANKDGIIWVATEGALNTPKDHIGTRNPANNAAIVLQLPQGTTLPKGFY
YALVYFLQSINFVRIIMRLWLCWKCRSKNPLLYDANYFLCWHTNCYDYCIPYNSVTSSIVITSGDGTTSPISEHD
RSMWSFNPETNILLNVPLHGTILTRPLLESELVIGAVILRGHLRIAGHHLGRCDIKDLPKEITVATSRTLSYYKLGAS
ITLCFTLKRKTE*
FLEYHDVRVVLDFI*
MKDLSPRWYFYYLGTGPEAGLPYGANKDGIIWVATEGALNTPKDHIGTRNPANNAAIVLQLPQGTTLPKGFY
FGTTLDSKTQSLLIVNNATNVVIKVCEFQFCNDPFLGVYYHKNNKSWMESEFRVYSSANNCTFEYVSQPFLMD
YALVYFLQSINFVRIIMRLWLCWKCRSKNPLLYDANYFLCWHTNCYDYCIPYNSVTSSIVITSGDGTTSPISEHD

RSMWSFNPETNILLNVPLHGTILTRPLLESELVIGAVILRGHLRIAGHHLGRCDIKDLPKEITVATSRTLSYYKLGAS

ITLCFTLKRKTE*

FLEYHDVRVVLDFI*
MKDLSPRWYFYYLGTGPEAGLPYGANKDGIIWVATEGALNTPKDHIGTRNPANNAAIVLQLPQGTTLPKGFY
FGTTLDSKTQSLLIVNNATNVVIKVCEFQFCNDPFLGVYYHKNNKSWMESEFRVYSSANNCTFEYVSQPFLMD
YALVYFLQSINFVRIIMRLWLCWKCRSKNPLLYDANYFLCWHTNCYDYCIPYNSVTSSIVITSGDGTTSPISEHD

RSMWSFNPETNILLNVPLHGTILTRPLLESELVIGAVILRGHLRIAGHHLGRCDIKDLPKEITVATSRTLSYYKLGAS

ITLCFTLKRKTE*

FLEYHDVRVVLDFI*
MKDLSPRWYFYYLGTGPEAGLPYGANKDGIIWVATEGALNTPKDHIGTRNPANNAAIVLQLPQGTTLPKGFY
FGTTLDSKTQSLLIVNNATNVVIKVCEFQFCNDPFLGVYYHKNNKSWMESEFRVYSSANNCTFEYVSQPFLMD
YALVYFLQSINFVRIIMRLWLCWKCRSKNPLLYDANYFLCWHTNCYDYCIPYNSVTSSIVITSGDGTTSPISEHD
RSMWSFNPETNILLNVPLHGTILTRPLLESELVIGAVILRGHLRIAGHHLGRCDIKDLPKEITVATSRTLSYYKLGAS
MKDLSPRWYFYYLGTGPEAGLPYGANKDGIIWVATEGALNTPKDHIGTRNPANNAAIVLQLPQGTTLPKGFY
FGTTLDSKTQSLLIVNNATNVVIKVCEFQFCNDPFLGVYYHKNNKSWMESEFRVYSSANNCTFEYVSQPFLMD
YALVYFLQSINFVRIIMRLWLCWKCRSKNPLLYDANYFLCWHTNCYDYCIPYNSVTSSIVITSGDGTTSPISEHD
RSMWSFNPETNILLNVPLHGTILTRPLLESELVIGAVILRGHLRIAGHHLGRCDIKDLPKEITVATSRTLSYYKLGAS

ITLCFTLKRKTE*

FLEYHDVRVVLDFI*
MKDLSPRWYFYYLGTGPEAGLPYGANKDGIIWVATEGALNTPKDHIGTRNPANNAAIVLQLPQGTTLPKGFY
ITLCFTLKRKTE*
MKDLSPRWYFYYLGTGPEAGLPYGANKDGIIWVATEGALNTPKDHIGTRNPANNAAIVLQLPQGTTLPKGFY

MKDLSPRWYFYYLGTGPEAGLPYGANKDGIIWVATEGALNTPKDHIGTRNPANNAAIVLQLPQGTTLPKGFY

SMWSFNPETNILLNVPLHGTILTRPLLESELVIGAVILRGHLRIAGHHLGRCDIKDLPKEITVATSRTLSYYKLGA

MKDLSPRWYFYYLGTGPEAGLPYGANKDGIIWVATEGALNTPKDHIGTRNPANNAAIVLQLPQGTTLPKGF
SMWSFNPETNILLNVPLHGTILTRPLLESELVIGAVILRGHLRIAGHHLGRCDIKDLPKEITVATSRTLSYYKLGA
MKDLSPRWYFYYLGTGPEAGLPYGANKDGIIWVATEGALNTPKDHIGTRNPANNAAIVLQLPQGTTLPKGF
YALVYFLQSINFVRIIMRLWLCWKCRSKNPLLYDANYFLCWHTNCYDYCIPYNSVTSSIVITSGDGTTSPISEHD
refer to the first AUG position and translated orf of the sgmRNA.

SQRVAGDSGFAAYSRYRIGNYKLNTDHSSSDNIALLVQ*

YAEGSRGGSQASSRSSSRSRNSSRNSTPGSSRGTSPARMAGNGGDAALALLLLDRLNQLESKMSGKGQQQQ
YQIGGYTEKWESGVKDCVVLHSYFTSDYYQLYSTQLSTDTGVEHVTFFIYNKIVDEPEEHVQIHTIDGSSGVVN
SQRVAGDSGFAAYSRYRIGNYKLNTDHSSSDNIALLVQ*

YAEGSRGGSQASSRSSSRSRNSSRNSTPGSSRGTSPARMAGNGGDAALALLLLDRLNQLESKMSGKGQQQQ
DLEGKQGNFKNLREFVFKNIDGYFKIYSKHTPINLVRDLPQGFSALEPLVDLPIGINITRFQTLLALHRSYLTPGDS
YQIGGYTEKWESGVKDCVVLHSYFTSDYYQLYSTQLSTDTGVEHVTFFIYNKIVDEPEEHVQIHTIDGSSGVVN

SQRVAGDSGFAAYSRYRIGNYKLNTDHSSSDNIALLVQ*

YAEGSRGGSQASSRSSSRSRNSSRNSTPGSSRGTSPARMAGNGGDAALALLLLDRLNQLESKMSGKGQQQQ
DLEGKQGNFKNLREFVFKNIDGYFKIYSKHTPINLVRDLPQGFSALEPLVDLPIGINITRFQTLLALHRSYLTPGDS
YQIGGYTEKWESGVKDCVVLHSYFTSDYYQLYSTQLSTDTGVEHVTFFIYNKIVDEPEEHVQIHTIDGSSGVVN

SQRVAGDSGFAAYSRYRIGNYKLNTDHSSSDNIALLVQ*

YAEGSRGGSQASSRSSSRSRNSSRNSTPGSSRGTSPARMAGNGGDAALALLLLDRLNQLESKMSGKGQQQQ
DLEGKQGNFKNLREFVFKNIDGYFKIYSKHTPINLVRDLPQGFSALEPLVDLPIGINITRFQTLLALHRSYLTPGDS
YQIGGYTEKWESGVKDCVVLHSYFTSDYYQLYSTQLSTDTGVEHVTFFIYNKIVDEPEEHVQIHTIDGSSGVVN
SQRVAGDSGFAAYSRYRIGNYKLNTDHSSSDNIALLVQ*
YAEGSRGGSQASSRSSSRSRNSSRNSTPGSSRGTSPARMAGNGGDAALALLLLDRLNQLESKMSGKGQQQQ
DLEGKQGNFKNLREFVFKNIDGYFKIYSKHTPINLVRDLPQGFSALEPLVDLPIGINITRFQTLLALHRSYLTPGDS
YQIGGYTEKWESGVKDCVVLHSYFTSDYYQLYSTQLSTDTGVEHVTFFIYNKIVDEPEEHVQIHTIDGSSGVVN
SQRVAGDSGFAAYSRYRIGNYKLNTDHSSSDNIALLVQ*

YAEGSRGGSQASSRSSSRSRNSSRNSTPGSSRGTSPARMAGNGGDAALALLLLDRLNQLESKMSGKGQQQQ

YAEGSRGGSQASSRSSSRSRNSSRNSTPGSSRGTSPARMAGNGGDAALALLLLDRLNQLESKMSGKGQQQQ

YAEGSRGGSQASSRSSSRSRNSSRNSTPGSSRGTSPARMAGNGGDAALALLLLDRLNQLESKMSGKGQQQQ

SQRVAGDSGFAAYSRYRIGNYKLNTDHSSSSDNIALLVQ*

YAEGSRGGSQASSRSSSRSRNSSRNSTPGSSRGTSPARMAGNGGDAALALLLLDRLNQLESKMSGKGQQQQ
SQRVAGDSGFAAYSRYRIGNYKLNTDHSSSSDNIALLVQ*
YAEGSRGGSQASSRSSSRSRNSSRNSTPGSSRGTSPARMAGNGGDAALALLLLDRLNQLESKMSGKGQQQQ
YQIGGYTEKWESGVKDCVVLHSYFTSDYYQLYSTQLSTDTGVEHVTFFIYNKIVDEPEEHVQIHTIDGSSGVVN

GQTVTKKSAAEASKKPRQKRTATKAYNVTQAFGRRGPEQTQGNFGDQELIRQGTDYKHWPQIAQFAPSAS
PVMEPIYDEPTTTTSVPL*

GQTVTKKSAAEASKKPRQKRTATKAYNVTQAFGRRGPEQTQGNFGDQELIRQGTDYKHWPQIAQFAPSAS
SSGWTAGAAAYYVGYLQPRTFLLKYNENGTITDAVDCALDPLSETKCTLKSFTVEKGIYQTSNFRVQPTESIVR
PVMEPIYDEPTTTTSVPL*

GQTVTKKSAAEASKKPRQKRTATKAYNVTQAFGRRGPEQTQGNFGDQELIRQGTDYKHWPQIAQFAPSAS
SSGWTAGAAAYYVGYLQPRTFLLKYNENGTITDAVDCALDPLSETKCTLKSFTVEKGIYQTSNFRVQPTESIVR
PVMEPIYDEPTTTTSVPL*

GQTVTKKSAAEASKKPRQKRTATKAYNVTQAFGRRGPEQTQGNFGDQELIRQGTDYKHWPQIAQFAPSAS
SSGWTAGAAAYYVGYLQPRTFLLKYNENGTITDAVDCALDPLSETKCTLKSFTVEKGIYQTSNFRVQPTESIVR
PVMEPIYDEPTTTTSVPL*

GQTVTKKSAAEASKKPRQKRTATKAYNVTQAFGRRGPEQTQGNFGDQELIRQGTDYKHWPQIAQFAPSAS
SSGWTAGAAAYYVGYLQPRTFLLKYNENGTITDAVDCALDPLSETKCTLKSFTVEKGIYQTSNFRVQPTESIVR
PVMEPIYDEPTTTTSVPL*

GQTVTKKSAAEASKKPRQKRTATKAYNVTQAFGRRGPEQTQGNFGDQELIRQGTDYKHWPQIAQFAPSAS

GQTVTKKSAAEASKKPRQKRTATKAYNVTQAFGRRGPEQTQGNFGDQELIRQGTDYKHWPQIAQFAPSAS

GQTVTKKSAAEASKKPRQKRTATKAYNVTQAFGRRGPEQTQGNFGDQELIRQGTDYKHWPQIAQFAPSAS

GQTVTKKSAAEASKKPRQKRTATKAYNVTQAFGRRGPEQTQGNFGDQELIRQGTDYKHWPQIAQFAPSAS[illegible]

GQTVTKKSAAEASKKPRQKRTATKAYNVTQAFGRRGPEQTQGNFGDQELIRQGTDYKHWPQIAQFAPSAS[illegible]
PVMEPIYDEPTTTTSVPL*

AFFGMSRIGMEVTPSGTWLTYTGAIKLDDKDPNFKDQVILLNKHIDAYKTFPPTEPKKDKKKKADETQALPQR

AFFGMSRIGMEVTPSGTWLTYTGAIKLDDKDPNFKDQVILLNKHIDAYKTFPPTEPKKDKKKKADETQALPQR
FPNITNLCPFGEVFNATRFASVYAWNRKRISNCVADYSVLYNSASFSTFKCYGVSPTKLNDLCFTNVYADSFVII

AFFGMSRIGMEVTPSGTWLTYTGAIKLDDKDPNFKDQVILLNKHIDAYKTFPPTEPKKDKKKKADETQALPQR
FPNITNLCPFGEVFNATRFASVYAWNRKRISNCVADYSVLYNSASFSTFKCYGVSPTKLNDLCFTNVYADSFVII

AFFGMSRIGMEVTPSGTWLTYTGAIKLDDKDPNFKDQVILLNKHIDAYKTFPPTEPKKDKKKKADETQALPQR
FPNITNLCPFGEVFNATRFASVYAWNRKRISNCVADYSVLYNSASFSTFKCYGVSPTKLNDLCFTNVYADSFVII

AFFGMSRIGMEVTPSGTWLTYTGAIKLDDKDPNFKDQVILLNKHIDAYKTFPPTEPKKDKKKKADETQALPQR
FPNITNLCPFGEVFNATRFASVYAWNRKRISNCVADYSVLYNSASFSTFKCYGVSPTKLNDLCFTNVYADSFVII

AFFGMSRIGMEVTPSGTWLTYTGAIKLDDKDPNFKDQVILLNKHIDAYKTFPPTEPKKDKKKKADETQALPQR

AFFGMSRIGMEVTPSGTWLTYTGAIKLDDKDPNFKDQVILLNKHIDAYKTFPPTEPKKDKKKKADETQALPQR

AFFGMSRIGMEVTPSGTWLTYTGAIKLDDKDPNFKDQVILLNKHIDAYKTFPPTEPKKDKKKKADETQALPQR

AFFGMSRIGMEVTPSGTWLTYTGAIKLDDKDPNFKDQVILLNKHIDAYKTFPPTEPKKDKKKKADETQALPQR

AFFGMSRIGMEVTPSGTWLTYTGAIKLDDKDPNFKDQVILLNKHIDAYKTFPPTEPKKDKKKKADETQALPQR

QKKQQTVTLLPAADLDDFSKQLQQSMSSADSTQA*

QKKQQTVTLLPAADLDDFSKQLQQSMSSADSTQA*
RGDEVRQIAPGQTGKIADYNYKLPDDFTGCVIAWNSNNLDSKVGGNYNYLYRLFRKSNLKPFERDISTEIYQA[illegible]

QKKQQTVTLLPAADLDDFSKQLQQSMSSADSTQA*
RGDEVRQIAPGQTGKIADYNYKLPDDFTGCVIAWNSNNLDSKVGGNYNYLYRLFRKSNLKPFERDISTEIYQA[illegible]

QKKQQTVTLLPAADLDDFSKQLQQSMSSADSTQA*
RGDEVRQIAPGQTGKIADYNYKLPDDFTGCVIAWNSNNLDSKVGGNYNYLYRLFRKSNLKPFERDISTEIYQA[illegible]

QKKQQTVTLLPAADLDDFSKQLQQSMSSADSTQA*
RGDEVRQIAPGQTGKIADYNYKLPDDFTGCVIAWNSNNLDSKVGGNYNYLYRLFRKSNLKPFERDISTEIYQA[illegible]

QKKQQTVTLLPAADLDDFSKQLQQSMSSADSTQA*

QKKQQTVTLLPAADLDDFSKQLQQSMSSADSTQA*

QKKQQTVTLLPAADLDDFSKQLQQSMSSADSTQA*

QKKQQTVTLLPAADLDDFSKQLQQSMSSADSTQA*

QKKQQTVTLLPAADLDDFSKQLQQSMSSADSTQA*

GSTPCNGVEGFNCYFPLQSYGFQPTNGVGYQPYRVVVLSFELLHAPATVCGPKKSTNLVKNKCVNFNFNGLT

GSTPCNGVEGFNCYFPLQSYGFQPTNGVGYQPYRVVVLSFELLHAPATVCGPKKSTNLVKNKCVNFNFNGLT

GSTPCNGVEGFNCYFPLQSYGFQPTNGVGYQPYRVVVLSFELLHAPATVCGPKKSTNLVKNKCVNFNFNGLT

GSTPCNGVEGFNCYFPLQSYGFQPTNGVGYQPYRVVVLSFELLHAPATVCGPKKSTNLVKNKCVNFNFNGLT

GTGVLTESNKKFLPFQQFGRDIADTTDAVRDPQTLEILDITPCSFGGVSVITPGTNTSNQVAVLYQDVNCTEVP

GTGVLTESNKKFLPFQQFGRDIADTTDAVRDPQTLEILDITPCSFGGVSVITPGTNTSNQVAVLYQDVNCTEVP

GTGVLTESNKKFLPFQQFGRDIADTTDAVRDPQTLEILDITPCSFGGVSVITPGTNTSNQVAVLYQDVNCTEVP

GTGVLTESNKKFLPFQQFGRDIADTTDAVRDPQTLEILDITPCSFGGVSVITPGTNTSNQVAVLYQDVNCTEVP

VAIHADQLTPTWRVYSTGSNVFQTRAGCLIGAEHVNNSYECDIPIGAGICASYQTQTNSPRRARSVASQSIIA

VAIHADQLTPTWRVYSTGSNVFQTRAGCLIGAEHVNNSYECDIPIGAGICASYQTQTNSPRRARSVASQSIIA

VAIHADQLTPTWRVYSTGSNVFQTRAGCLIGAEHVNNSYECDIPIGAGICASYQTQTNSPRRARSVASQSIIA

VAIHADQLTPTWRVYSTGSNVFQTRAGCLIGAEHVNNSYECDIPIGAGICASYQTQTNSPRRARSVASQSIIA

YTMSLGAENSVAYSNNSIAIPTNFTISVTTEILPVSMTKTSVDCTMYICGDSTECSNLLLQYGSFCTQLNRALTGI

YTMSLGAENSVAYSNNSIAIPTNFTISVTTEILPVSMTKTSVDCTMYICGDSTECSNLLLQYGSFCTQLNRALTGI

YTMSLGAENSVAYSNNSIAIPTNFTISVTTEILPVSMTKTSVDCTMYICGDSTECSNLLLQYGSFCTQLNRALTGI

YTMSLGAENSVAYSNNSIAIPTNFTISVTTEILPVSMTKTSVDCTMYICGDSTECSNLLLQYGSFCTQLNRALTGI

AVEQDKNTQEVFAQVKQIYKTPPIKDFGGFNFSQILPDPSKPSKRSFIEDLLFNKVTLADAGFIKQYGDCLGDIA

AVEQDKNTQEVFAQVKQIYKTPPIKDFGGFNFSQILPDPSKPSKRSFIEDLLFNKVTLADAGFIKQYGDCLGDIA

AVEQDKNTQEVFAQVKQIYKTPPIKDFGGFNFSQILPDPSKPSKRSFIEDLLFNKVTLADAGFIKQYGDCLGDIA

AVEQDKNTQEVFAQVKQIYKTPPIKDFGGFNFSQILPDPSKPSKRSFIEDLLFNKVTLADAGFIKQYGDCLGDIA

AARDLICAQKFNGLTVLPPLLTDEMIAQYTSALLAGTITSGWTFGAGAALQIPFAMQMAYRFNGIGVTQNVLY

AARDLICAQKFNGLTVLPPLLTDEMIAQYTSALLAGTITSGWTFGAGAALQIPFAMQMAYRFNGIGVTQNVLY

AARDLICAQKFNGLTVLPPLLTDEMIAQYTSALLAGTITSGWTFGAGAALQIPFAMQMAYRFNGIGVTQNVLY

AARDLICAQKFNGLTVLPPLLTDEMIAQYTSALLAGTITSGWTFGAGAALQIPFAMQMAYRFNGIGVTQNVLY

ENQKLIANQFNSAIGKIQDSLSSTASALGKLQDVVNQNAQALNTLVKQLSSNFGAISSVLNDILSRLDKVEAEV

ENQKLIANQFNSAIGKIQDSLSSTASALGKLQDVVNQNAQALNTLVKQLSSNFGAISSVLNDILSRLDKVEAEV

ENQKLIANQFNSAIGKIQDSLSSTASALGKLQDVVNQNAQALNTLVKQLSSNFGAISSVLNDILSRLDKVEAEV

ENQKLIANQFNSAIGKIQDSLSSTASALGKLQDVVNQNAQALNTLVKQLSSNFGAISSVLNDILSRLDKVEAEV

/QIDRLITGRLQSLQTYVTQQLIRAAEIRASANL

/QIDRLITGRLQSLQTYVTQQLIRAAEIRASANL

/QIDRLITGRLQSLQTYVTQQLIRAAEIRASANL

/QIDRLITGRLQSLQTYVTQQLIRAAEIRASANL

	subgenome	peak_leader_	peak_TRS_sta	ACGAAC	ATG_postion	20_leader_se
S1-Day1	S	65	21552	yes	21563	ATCTCTTGTA
	ORF3a	69	25385	yes	25393	CTTGTAGATC
	E	69	26237	yes	26245	CTTGTAGATC
	M	65	26469	yes	26523	ATCTCTTGTA
	N	64	28255	yes	28274	GATCTCTTGT
S2-Day2	S	65	21552	yes	21563	ATCTCTTGTA
	ORF3a	69	25385	yes	25393	CTTGTAGATC
	E	69	26237	yes	26245	CTTGTAGATC
	M	64	26468	yes	26523	GATCTCTTGT
	ORF6	69	27041	yes	27202	CTTGTAGATC
	ORF7a	69	27388	yes	27394	CTTGTAGATC
	N	64	28255	yes	28274	GATCTCTTGT
S3-Day3	S	65	21552	yes	21563	ATCTCTTGTA
	ORF3a	69	25385	yes	25393	CTTGTAGATC
	M	65	26469	yes	26523	ATCTCTTGTA
	ORF6	69	27041	yes	27202	CTTGTAGATC
	ORF7a	69	27388	yes	27394	CTTGTAGATC
	ORF8	65	27884	yes	27894	ATCTCTTGTA
	N	64	28255	yes	28274	GATCTCTTGT
S1-Day4	S	65	21552	yes	21563	ATCTCTTGTA
	ORF3a	69	25385	yes	25393	CTTGTAGATC
	M	65	26469	yes	26523	ATCTCTTGTA
	ORF6	69	27041	yes	27202	CTTGTAGATC
	ORF7a	69	27388	yes	27394	CTTGTAGATC
	ORF7b	65	27644	no	27756	ATCTCTTGTA
	ORF8	65	27884	yes	27894	ATCTCTTGTA
	N	64	28255	yes	28274	GATCTCTTGT
S2-Day5	S	65	21552	yes	21563	ATCTCTTGTA
	M	65	26469	yes	26523	ATCTCTTGTA
	ORF7b	65	27644	no	27756	ATCTCTTGTA
	N	68	28263	no	28274	TCTTGTAGAT
S3-Day6	S	65	21552	yes	21563	ATCTCTTGTA
	ORF3a	69	25385	yes	25393	CTTGTAGATC
	E	69	26237	yes	26245	CTTGTAGATC
	M	64	26468	yes	26523	GATCTCTTGT
	ORF6	69	27041	yes	27202	CTTGTAGATC
	ORF7a	69	27388	yes	27394	CTTGTAGATC
	ORF8	65	27884	yes	27894	ATCTCTTGTA
	N	65	28256	yes	28274	ATCTCTTGTA
S2-Day8	ORF3a	69	25385	yes	25393	CTTGTAGATC
	ORF7a	69	27388	yes	27394	CTTGTAGATC
	N	65	28256	yes	28274	ATCTCTTGTA

S3-Day9	M	65	26469	yes	26523	ATCTCTTGTA
	N	65	28256	yes	28274	ATCTCTTGTA
S2-Day11	ORF3a	69	25385	yes	25393	CTTGTAGATC
	M	65	26469	yes	26523	ATCTCTTGTA
S3-Day12	N	65	28256	yes	28274	ATCTCTTGTA
S3-Day15	ORF3a	69	25385	yes	25393	CTTGTAGATC
	ORF6	69	27041	yes	27202	CTTGTAGATC
	ORF7a	69	27388	yes	27394	CTTGTAGATC
	N	65	28256	yes	28274	ATCTCTTGTA

“peak_leader” and “peak_TRS_start” point to the leader-TRS junctions in sheet "known", “ACG

TRS_seq	first_orf_aa
CTAAACGAA	MFVFLVLLPLVSSQCVNLTTRTQLPPAYTNSFTRGVYYPDKVFRSSVLHSTQDLFLPFFSNVT
ACGAACTT	MDLFMRIFTIGTVTLKQGEIKDATPSDFVRATATIPIQASLPFGWLIVGVALLAVFQSASKIITL
ACGAACTT	MYSFVSEETGTLIVNSVLLFLAFVVFLLVTLAILTALRLCAYCCNIVNVSLVKPSFYVYSRVKNLN
CTAAACGAA	MADSNGTITVEELKKLLEQWNLVIGFLFLTWICLLQFAYANRNRFLYIIKLIFLWLLWPVTLAC
TCTAAACGA	MSDNGPQNQRNAPRITFGGPSDSTGSNQNGERSGARSKQRRPQGLPNNTASWFTALTQ
CTAAACGAA	MFVFLVLLPLVSSQCVNLTTRTQLPPAYTNSFTRGVYYPDKVFRSSVLHSTQDLFLPFFSNVT
ACGAACTT	MDLFMRIFTIGTVTLKQGEIKDATPSDFVRATATIPIQASLPFGWLIVGVALLAVFQSASKIITL
ACGAACTT	MYSFVSEETGTLIVNSVLLFLAFVVFLLVTLAILTALRLCAYCCNIVNVSLVKPSFYVYSRVKNLN
TCTAAACGA	MADSNGTITVEELKKLLEQWNLVIGFLFLTWICLLQFAYANRNRFLYIIKLIFLWLLWPVTLAC
ACGAACGCT	MFHLVDFQVTIAEILLIIMRTFKVSIWNLDYIINLIIKNLSKSLTENKYSQLDEEQPMEID*
ACGAAC	MKIILFLALITLATCELYHYQECVRGTTVLLKEPCSSGTYEGNSPFHPLADNKFALTCFSTQFAF
TCTAAACGA	MSDNGPQNQRNAPRITFGGPSDSTGSNQNGERSGARSKQRRPQGLPNNTASWFTALTQ
CTAAACGAA	MFVFLVLLPLVSSQCVNLTTRTQLPPAYTNSFTRGVYYPDKVFRSSVLHSTQDLFLPFFSNVT
ACGAACTT	MDLFMRIFTIGTVTLKQGEIKDATPSDFVRATATIPIQASLPFGWLIVGVALLAVFQSASKIITL
CTAAACGAA	MADSNGTITVEELKKLLEQWNLVIGFLFLTWICLLQFAYANRNRFLYIIKLIFLWLLWPVTLAC
ACGAACGCT	MFHLVDFQVTIAEILLIIMRTFKVSIWNLDYIINLIIKNLSKSLTENKYSQLDEEQPMEID*
ACGAAC	MKIILFLALITLATCELYHYQECVRGTTVLLKEPCSSGTYEGNSPFHPLADNKFALTCFSTQFAF
CTAAACGAA	MKFLVFLGIITTVAAFHQECSLQSCTQHQPYVVDDPCPIHFYSKWYIRVGARKSAPLIELCVD
TCTAAACGA	MSDNGPQNQRNAPRITFGGPSDSTGSNQNGERSGARSKQRRPQGLPNNTASWFTALTQ
CTAAACGAA	MFVFLVLLPLVSSQCVNLTTRTQLPPAYTNSFTRGVYYPDKVFRSSVLHSTQDLFLPFFSNVT
ACGAACTT	MDLFMRIFTIGTVTLKQGEIKDATPSDFVRATATIPIQASLPFGWLIVGVALLAVFQSASKIITL
CTAAACGAA	MADSNGTITVEELKKLLEQWNLVIGFLFLTWICLLQFAYANRNRFLYIIKLIFLWLLWPVTLAC
ACGAACGCT	MFHLVDFQVTIAEILLIIMRTFKVSIWNLDYIINLIIKNLSKSLTENKYSQLDEEQPMEID*
ACGAAC	MKIILFLALITLATCELYHYQECVRGTTVLLKEPCSSGTYEGNSPFHPLADNKFALTCFSTQFAF
CTAAACTGTT	MIELSLIDFYLCFLAFLLFLVLIMLIIFWFSLELQDHNETCHA*
CTAAACGAA	MKFLVFLGIITTVAAFHQECSLQSCTQHQPYVVDDPCPIHFYSKWYIRVGARKSAPLIELCVD
TCTAAACGA	MSDNGPQNQRNAPRITFGGPSDSTGSNQNGERSGARSKQRRPQGLPNNTASWFTALTQ
CTAAACGAA	MFVFLVLLPLVSSQCVNLTTRTQLPPAYTNSFTRGVYYPDKVFRSSVLHSTQDLFLPFFSNVT
CTAAACGAA	MADSNGTITVEELKKLLEQWNLVIGFLFLTWICLLQFAYANRNRFLYIIKLIFLWLLWPVTLAC
CTAAACTGTT	MIELSLIDFYLCFLAFLLFLVLIMLIIFWFSLELQDHNETCHA*
AACAAACTA	MSDNGPQNQRNAPRITFGGPSDSTGSNQNGERSGARSKQRRPQGLPNNTASWFTALTQ
CTAAACGAA	MFVFLVLLPLVSSQCVNLTTRTQLPPAYTNSFTRGVYYPDKVFRSSVLHSTQDLFLPFFSNVT
ACGAACTT	MDLFMRIFTIGTVTLKQGEIKDATPSDFVRATATIPIQASLPFGWLIVGVALLAVFQSASKIITL
ACGAACTT	MYSFVSEETGTLIVNSVLLFLAFVVFLLVTLAILTALRLCAYCCNIVNVSLVKPSFYVYSRVKNLN
TCTAAACGA	MADSNGTITVEELKKLLEQWNLVIGFLFLTWICLLQFAYANRNRFLYIIKLIFLWLLWPVTLAC
ACGAACGCT	MFHLVDFQVTIAEILLIIMRTFKVSIWNLDYIINLIIKNLSKSLTENKYSQLDEEQPMEID*
ACGAAC	MKIILFLALITLATCELYHYQECVRGTTVLLKEPCSSGTYEGNSPFHPLADNKFALTCFSTQFAF
CTAAACGAA	MKFLVFLGIITTVAAFHQECSLQSCTQHQPYVVDDPCPIHFYSKWYIRVGARKSAPLIELCVD
CTAAACGAA	MSDNGPQNQRNAPRITFGGPSDSTGSNQNGERSGARSKQRRPQGLPNNTASWFTALTQ
ACGAACTT	MDLFMRIFTIGTVTLKQGEIKDATPSDFVRATATIPIQASLPFGWLIVGVALLAVFQSASKIITL
ACGAAC	MKIILFLALITLATCELYHYQECVRGTTVLLKEPCSSGTYEGNSPFHPLADNKFALTCFSTQFAF
CTAAACGAA	MSDNGPQNQRNAPRITFGGPSDSTGSNQNGERSGARSKQRRPQGLPNNTASWFTALTQ

CTAAACGAA(	MADSNGTITVEELKKLLEQWNLVIGFLFLTWICLLQFAYANRNRFLYIIKLIFLWLLWPVTLACI
CTAAACGAA(	MSDNGPQNQRNAPRITFGGPSDSTGSNQNGERSGARSKQRRPQGLPNNTASWFTALTQH
ACGAACTT	MDLFMRIFTIGTVTLKQGEIKDATPSDFVRATATIPIQASLPFGWLIVGVALLAVFQSASKIITL
CTAAACGAA(	MADSNGTITVEELKKLLEQWNLVIGFLFLTWICLLQFAYANRNRFLYIIKLIFLWLLWPVTLACI
CTAAACGAA(	MSDNGPQNQRNAPRITFGGPSDSTGSNQNGERSGARSKQRRPQGLPNNTASWFTALTQH
ACGAACTT	MDLFMRIFTIGTVTLKQGEIKDATPSDFVRATATIPIQASLPFGWLIVGVALLAVFQSASKIITL
ACGAACGCTI	MFHLVDFQVTIAEILLIIMRTFKVSIWNLDYIINLIIKNLSKSLTENKYSQLDEEQPMEID*
ACGAAC	MKIILFLALITLATCELYHYQECVRGTTVLLKEPCSSGTYEGNSPFHPLADNKFALTCFSTQFAF/
CTAAACGAA(	MSDNGPQNQRNAPRITFGGPSDSTGSNQNGERSGARSKQRRPQGLPNNTASWFTALTQH

AAC” indicates if there is a ACGAAC sequence in the “TRS_seq” (TRS sequences), “20_leader_se(

VFHAIHVSGTNGTKRFDNPVLPFNDGVYFASTEKSNIIRGWIFGTTLDSKTQSLLIVNNATNVVIKVCEFQFCN
KKRWQLALSKGVHFVCNLLLLFVTVYSHLLLVAAGLEAPFLYLYALVYFLQSINFVRIIMRLWLCWKCRSKNPLL
ISSRVPDLLV*
FVLAAVYRINWITGGIAIAMACLVGLMWLSYFIASFRLFARTRSMWSFNPETNILLNVPLHGTILTRPLLESELV
IGKEDLKFPRGQGVPINTNSSPDDQIGYYRRATRRIRGGDGKMKDLSPRWYFYYLGTGPEAGLPYGANKDGII
VFHAIHVSGTNGTKRFDNPVLPFNDGVYFASTEKSNIIRGWIFGTTLDSKTQSLLIVNNATNVVIKVCEFQFCN
KKRWQLALSKGVHFVCNLLLLFVTVYSHLLLVAAGLEAPFLYLYALVYFLQSINFVRIIMRLWLCWKCRSKNPLL
ISSRVPDLLV*
FVLAAVYRINWITGGIAIAMACLVGLMWLSYFIASFRLFARTRSMWSFNPETNILLNVPLHGTILTRPLLESELV

ACPDGVKHVYQLRARSVSPKLFIRQEEVQELYSPIFLIVAAIVFITLCFTLKRKTE*
IGKEDLKFPRGQGVPINTNSSPDDQIGYYRRATRRIRGGDGKMKDLSPRWYFYYLGTGPEAGLPYGANKDGII
VFHAIHVSGTNGTKRFDNPVLPFNDGVYFASTEKSNIIRGWIFGTTLDSKTQSLLIVNNATNVVIKVCEFQFCN
KKRWQLALSKGVHFVCNLLLLFVTVYSHLLLVAAGLEAPFLYLYALVYFLQSINFVRIIMRLWLCWKCRSKNPLL
FVLAAVYRINWITGGIAIAMACLVGLMWLSYFIASFRLFARTRSMWSFNPETNILLNVPLHGTILTRPLLESELV

ACPDGVKHVYQLRARSVSPKLFIRQEEVQELYSPIFLIVAAIVFITLCFTLKRKTE*
EAGSKSPIQYIDIGNYTVSCLPFTINCQEPKLGSLVVRCSFYEDFLEYHDVRVVLDFI*
IGKEDLKFPRGQGVPINTNSSPDDQIGYYRRATRRIRGGDGKMKDLSPRWYFYYLGTGPEAGLPYGANKDGII
VFHAIHVSGTNGTKRFDNPVLPFNDGVYFASTEKSNIIRGWIFGTTLDSKTQSLLIVNNATNVVIKVCEFQFCN
KKRWQLALSKGVHFVCNLLLLFVTVYSHLLLVAAGLEAPFLYLYALVYFLQSINFVRIIMRLWLCWKCRSKNPLL
FVLAAVYRINWITGGIAIAMACLVGLMWLSYFIASFRLFARTRSMWSFNPETNILLNVPLHGTILTRPLLESELV

ACPDGVKHVYQLRARSVSPKLFIRQEEVQELYSPIFLIVAAIVFITLCFTLKRKTE*

EAGSKSPIQYIDIGNYTVSCLPFTINCQEPKLGSLVVRCSFYEDFLEYHDVRVVLDFI*
IGKEDLKFPRGQGVPINTNSSPDDQIGYYRRATRRIRGGDGKMKDLSPRWYFYYLGTGPEAGLPYGANKDGII
VFHAIHVSGTNGTKRFDNPVLPFNDGVYFASTEKSNIIRGWIFGTTLDSKTQSLLIVNNATNVVIKVCEFQFCN
FVLAAVYRINWITGGIAIAMACLVGLMWLSYFIASFRLFARTRSMWSFNPETNILLNVPLHGTILTRPLLESELV

IGKEDLKFPRGQGVPINTNSSPDDQIGYYRRATRRIRGGDGKMKDLSPRWYFYYLGTGPEAGLPYGANKDGII
VFHAIHVSGTNGTKRFDNPVLPFNDGVYFASTEKSNIIRGWIFGTTLDSKTQSLLIVNNATNVVIKVCEFQFCN
KKRWQLALSKGVHFVCNLLLLFVTVYSHLLLVAAGLEAPFLYLYALVYFLQSINFVRIIMRLWLCWKCRSKNPLL
ISSRVPDLLV*
FVLAAVYRINWITGGIAIAMACLVGLMWLSYFIASFRLFARTRSMWSFNPETNILLNVPLHGTILTRPLLESELV

ACPDGVKHVYQLRARSVSPKLFIRQEEVQELYSPIFLIVAAIVFITLCFTLKRKTE*
EAGSKSPIQYIDIGNYTVSCLPFTINCQEPKLGSLVVRCSFYEDFLEYHDVRVVLDFI*
IGKEDLKFPRGQGVPINTNSSPDDQIGYYRRATRRIRGGDGKMKDLSPRWYFYYLGTGPEAGLPYGANKDGII
KKRWQLALSKGVHFVCNLLLLFVTVYSHLLLVAAGLEAPFLYLYALVYFLQSINFVRIIMRLWLCWKCRSKNPLL
ACPDGVKHVYQLRARSVSPKLFIRQEEVQELYSPIFLIVAAIVFITLCFTLKRKTE*
IGKEDLKFPRGQGVPINTNSSPDDQIGYYRRATRRIRGGDGKMKDLSPRWYFYYLGTGPEAGLPYGANKDGII

FVLAAVYRINWITGGIAIAMACLVGLMWLSYFIASFRLFARTRSMWSFNPETNILLNVPLHGTILTRPLLESELV
HGKEDLKFPRGQGVPINTNSSPDDQIGYYRRATRRIRGGDGKMKDLSPRWYFYYLGTGPEAGLPYGANKDGII
KKRWQLALSKGVHFVCNLLLLFVTVYSHLLLVAAGLEAPFLYLYALVYFLQSINFVRIIMRLWLCWKCRSKNPLL
FVLAAVYRINWITGGIAIAMACLVGLMWLSYFIASFRLFARTRSMWSFNPETNILLNVPLHGTILTRPLLESELV
HGKEDLKFPRGQGVPINTNSSPDDQIGYYRRATRRIRGGDGKMKDLSPRWYFYYLGTGPEAGLPYGANKDGII
KKRWQLALSKGVHFVCNLLLLFVTVYSHLLLVAAGLEAPFLYLYALVYFLQSINFVRIIMRLWLCWKCRSKNPLL

ACPDGVKHVYQLRARSVSPKLFIRQEEVQELYSPIFLIVAAIVFITLCFTLKRKTE*
HGKEDLKFPRGQGVPINTNSSPDDQIGYYRRATRRIRGGDGKMKDLSPRWYFYYLGTGPEAGLPYGANKDGII
q” refers to the 20 nucleotides before the end of leader, and “AUG_postion” and “first_orf_aa”

DPFLGVYYHKNNKSWMESEFRVYSSANNCTFEYVSQPFLMDLEGKQGNFKNLREFVFKNIDGYFKIYSKHTPI
YDANYFLCWHTNCYDYCIPYNSVTSSIVITSGDGTTSPISEHDYQIGGYTEKWESGVKDCVVLHSYFTSDYYQL

IGAVILRGHLRIAGHHLGRCDIKDLPKEITVATSRTLSYYKLGASQRVAGDSGFAAYSRYRIGNYKLNTDHSSSD
IWVATEGALNTPKDHIGTRNPANNAAIVLQLPQGTTLPKGFYAEGSRGGSQASSRSSSRSRNSSRNSTPGSSR
DPFLGVYYHKNNKSWMESEFRVYSSANNCTFEYVSQPFLMDLEGKQGNFKNLREFVFKNIDGYFKIYSKHTPI
YDANYFLCWHTNCYDYCIPYNSVTSSIVITSGDGTTSPISEHDYQIGGYTEKWESGVKDCVVLHSYFTSDYYQL

IGAVILRGHLRIAGHHLGRCDIKDLPKEITVATSRTLSYYKLGASQRVAGDSGFAAYSRYRIGNYKLNTDHSSSD

IWVATEGALNTPKDHIGTRNPANNAAIVLQLPQGTTLPKGFYAEGSRGGSQASSRSSSRSRNSSRNSTPGSSR
DPFLGVYYHKNNKSWMESEFRVYSSANNCTFEYVSQPFLMDLEGKQGNFKNLREFVFKNIDGYFKIYSKHTPI
YDANYFLCWHTNCYDYCIPYNSVTSSIVITSGDGTTSPISEHDYQIGGYTEKWESGVKDCVVLHSYFTSDYYQL
IGAVILRGHLRIAGHHLGRCDIKDLPKEITVATSRTLSYYKLGASQRVAGDSGFAAYSRYRIGNYKLNTDHSSSD

IWVATEGALNTPKDHIGTRNPANNAAIVLQLPQGTTLPKGFYAEGSRGGSQASSRSSSRSRNSSRNSTPGSSR
DPFLGVYYHKNNKSWMESEFRVYSSANNCTFEYVSQPFLMDLEGKQGNFKNLREFVFKNIDGYFKIYSKHTPI
YDANYFLCWHTNCYDYCIPYNSVTSSIVITSGDGTTSPISEHDYQIGGYTEKWESGVKDCVVLHSYFTSDYYQL
IGAVILRGHLRIAGHHLGRCDIKDLPKEITVATSRTLSYYKLGASQRVAGDSGFAAYSRYRIGNYKLNTDHSSSD

IWVATEGALNTPKDHIGTRNPANNAAIVLQLPQGTTLPKGFYAEGSRGGSQASSRSSSRSRNSSRNSTPGSSR
DPFLGVYYHKNNKSWMESEFRVYSSANNCTFEYVSQPFLMDLEGKQGNFKNLREFVFKNIDGYFKIYSKHTPI
IGAVILRGHLRIAGHHLGRCDIKDLPKEITVATSRTLSYYKLGASQRVAGDSGFAAYSRYRIGNYKLNTDHSSSD

IWVATEGALNTPKDHIGTRNPANNAAIVLQLPQGTTLPKGFYAEGSRGGSQASSRSSSRSRNSSRNSTPGSSR
DPFLGVYYHKNNKSWMESEFRVYSSANNCTFEYVSQPFLMDLEGKQGNFKNLREFVFKNIDGYFKIYSKHTPI
YDANYFLCWHTNCYDYCIPYNSVTSSIVITSGDGTTSPISEHDYQIGGYTEKWESGVKDCVVLHSYFTSDYYQL

IGAVILRGHLRIAGHHLGRCDIKDLPKEITVATSRTLSYYKLGASQRVAGDSGFAAYSRYRIGNYKLNTDHSSSD

IWVATEGALNTPKDHIGTRNPANNAAIVLQLPQGTTLPKGFYAEGSRGGSQASSRSSSRSRNSSRNSTPGSSR
YDANYFLCWHTNCYDYCIPYNSVTSSIVITSGDGTTSPISEHDYQIGGYTEKWESGVKDCVVLHSYFTSDYYQL

IWVATEGALNTPKDHIGTRNPANNAAIVLQLPQGTTLPKGFYAEGSRGGSQASSRSSSRSRNSSRNSTPGSSR

IGAVILRGHLRIAGHHLGRCDIKDLPKEITVATSRTLSYYKLGASQRVAGDSGFAAYSRYRIGNYKLNTDHSSSSD
WVATEGALNTPKDHIGTRNPANNAAIVLQLPQGTTLPKGFYAEGSRGGSQASSRSSSRSRNSSRNSTPGSSR
YDANYFLCWHTNCYDYCIPYNSVTSSIVITSGDGTTSPISEHDYQIGGYTEKWESGVKDCVVLHSYFTSDYYQL
IGAVILRGHLRIAGHHLGRCDIKDLPKEITVATSRTLSYYKLGASQRVAGDSGFAAYSRYRIGNYKLNTDHSSSSD
WVATEGALNTPKDHIGTRNPANNAAIVLQLPQGTTLPKGFYAEGSRGGSQASSRSSSRSRNSSRNSTPGSSR
YDANYFLCWHTNCYDYCIPYNSVTSSIVITSGDGTTSPISEHDYQIGGYTEKWESGVKDCVVLHSYFTSDYYQL

WVATEGALNTPKDHIGTRNPANNAAIVLQLPQGTTLPKGFYAEGSRGGSQASSRSSSRSRNSSRNSTPGSSR
refer to the first AUG position and translated orf of the sgmRNA.

NLVRDLPQGFSALEPLVDLPIGINITRFQTLLALHRSYLTPGDSSSGWTAGAAAYYVGYLQPRTFLLKYNENGT
YSTQLSTDTGVEHVTFFIYNKIVDEPEEHVQIHTIDGSSGVVNPVMEPIYDEPTTTTSVPL*

NIALLVQ*
GTSPARMAGNGGDAALALLLLDRLNQLESKMSGKGQQQQGQTVTKKSAAEASKKPRQKRTATKAYNVTQ
NLVRDLPQGFSALEPLVDLPIGINITRFQTLLALHRSYLTPGDSSSGWTAGAAAYYVGYLQPRTFLLKYNENGT
YSTQLSTDTGVEHVTFFIYNKIVDEPEEHVQIHTIDGSSGVVNPVMEPIYDEPTTTTSVPL*

NIALLVQ*

GTSPARMAGNGGDAALALLLLDRLNQLESKMSGKGQQQQGQTVTKKSAAEASKKPRQKRTATKAYNVTQ
NLVRDLPQGFSALEPLVDLPIGINITRFQTLLALHRSYLTPGDSSSGWTAGAAAYYVGYLQPRTFLLKYNENGT
YSTQLSTDTGVEHVTFFIYNKIVDEPEEHVQIHTIDGSSGVVNPVMEPIYDEPTTTTSVPL*
NIALLVQ*

GTSPARMAGNGGDAALALLLLDRLNQLESKMSGKGQQQQGQTVTKKSAAEASKKPRQKRTATKAYNVTQ
NLVRDLPQGFSALEPLVDLPIGINITRFQTLLALHRSYLTPGDSSSGWTAGAAAYYVGYLQPRTFLLKYNENGT
YSTQLSTDTGVEHVTFFIYNKIVDEPEEHVQIHTIDGSSGVVNPVMEPIYDEPTTTTSVPL*
NIALLVQ*

GTSPARMAGNGGDAALALLLLDRLNQLESKMSGKGQQQQGQTVTKKSAAEASKKPRQKRTATKAYNVTQ
NLVRDLPQGFSALEPLVDLPIGINITRFQTLLALHRSYLTPGDSSSGWTAGAAAYYVGYLQPRTFLLKYNENGT
NIALLVQ*

GTSPARMAGNGGDAALALLLLDRLNQLESKMSGKGQQQQGQTVTKKSAAEASKKPRQKRTATKAYNVTQ
NLVRDLPQGFSALEPLVDLPIGINITRFQTLLALHRSYLTPGDSSSGWTAGAAAYYVGYLQPRTFLLKYNENGT
YSTQLSTDTGVEHVTFFIYNKIVDEPEEHVQIHTIDGSSGVVNPVMEPIYDEPTTTTSVPL*

NIALLVQ*

GTSPARMAGNGGDAALALLLLDRLNQLESKMSGKGQQQQGQTVTKKSAAEASKKPRQKRTATKAYNVTQ
YSTQLSTDTGVEHVTFFIYNKIVDEPEEHVQIHTIDGSSGVVNPVMEPIYDEPTTTTSVPL*

GTSPARMAGNGGDAALALLLLDRLNQLESKMSGKGQQQQGQTVTKKSAAEASKKPRQKRTATKAYNVTQ

[illegible]NIALLVQ*
GTSPARMAGNGGDAALALLLLDRLNQLESKMSGKGQQQQGQTVTKKSAAEASKKPRQKRTATKAYNVTQ[illegible]
YSTQLSTDTGVEHVTFFIYNKIVDEPEEHVQIHTIDGSSGVVNPVMEPIYDEPTTTTSVPL*
[illegible]NIALLVQ*
GTSPARMAGNGGDAALALLLLDRLNQLESKMSGKGQQQQGQTVTKKSAAEASKKPRQKRTATKAYNVTQ[illegible]
YSTQLSTDTGVEHVTFFIYNKIVDEPEEHVQIHTIDGSSGVVNPVMEPIYDEPTTTTSVPL*

GTSPARMAGNGGDAALALLLLDRLNQLESKMSGKGQQQQGQTVTKKSAAEASKKPRQKRTATKAYNVTQ[illegible]

ITDAVDCALDPLSETKCTLKSFTVEKGIYQTSNFRVQPTESIVRFPNITNLCPFGEVFNATRFASVYAWNRKRISN

AFGRRGPEQTQGNFGDQELIRQGTDYKHWPQIAQFAPSASAFFGMSRIGMEVTPSGTWLTYTGAIKLDDKD
ITDAVDCALDPLSETKCTLKSFTVEKGIYQTSNFRVQPTESIVRFPNITNLCPFGEVFNATRFASVYAWNRKRISN

AFGRRGPEQTQGNFGDQELIRQGTDYKHWPQIAQFAPSASAFFGMSRIGMEVTPSGTWLTYTGAIKLDDKD
ITDAVDCALDPLSETKCTLKSFTVEKGIYQTSNFRVQPTESIVRFPNITNLCPFGEVFNATRFASVYAWNRKRISN

AFGRRGPEQTQGNFGDQELIRQGTDYKHWPQIAQFAPSASAFFGMSRIGMEVTPSGTWLTYTGAIKLDDKD
ITDAVDCALDPLSETKCTLKSFTVEKGIYQTSNFRVQPTESIVRFPNITNLCPFGEVFNATRFASVYAWNRKRISN

AFGRRGPEQTQGNFGDQELIRQGTDYKHWPQIAQFAPSASAFFGMSRIGMEVTPSGTWLTYTGAIKLDDKD
ITDAVDCALDPLSETKCTLKSFTVEKGIYQTSNFRVQPTESIVRFPNITNLCPFGEVFNATRFASVYAWNRKRISN

AFGRRGPEQTQGNFGDQELIRQGTDYKHWPQIAQFAPSASAFFGMSRIGMEVTPSGTWLTYTGAIKLDDKD
ITDAVDCALDPLSETKCTLKSFTVEKGIYQTSNFRVQPTESIVRFPNITNLCPFGEVFNATRFASVYAWNRKRISN

AFGRRGPEQTQGNFGDQELIRQGTDYKHWPQIAQFAPSASAFFGMSRIGMEVTPSGTWLTYTGAIKLDDKD

AFGRRGPEQTQGNFGDQELIRQGTDYKHWPQIAQFAPSASAFFGMSRIGMEVTPSGTWLTYTGAIKLDDKD

AFGRRGPEQTQGNFGDQELIRQGTDYKHWPQIAQFAPSASAFFGMSRIGMEVTPSGTWLTYTGAIKLDDKD

AFGRRGPEQTQGNFGDQELIRQGTDYKHWPQIAQFAPSASAFFGMSRIGMEVTPSGTWLTYTGAIKLDDKD

AFGRRGPEQTQGNFGDQELIRQGTDYKHWPQIAQFAPSASAFFGMSRIGMEVTPSGTWLTYTGAIKLDDKD

NCVADYSVLYNSASFSTFKCYGVSPTKLNDLCFTNVYADSFVIRGDEVRQIAPGQTGKIADYNYKLPDDFTGCV

PNFKDQVILLNKHIDAYKTFPPTEPKKDKKKKADETQALPQRQKKQQTVTLLPAADLDDFSKQLQQSMSSAD
NCVADYSVLYNSASFSTFKCYGVSPTKLNDLCFTNVYADSFVIRGDEVRQIAPGQTGKIADYNYKLPDDFTGCV

PNFKDQVILLNKHIDAYKTFPPTEPKKDKKKKADETQALPQRQKKQQTVTLLPAADLDDFSKQLQQSMSSAD
NCVADYSVLYNSASFSTFKCYGVSPTKLNDLCFTNVYADSFVIRGDEVRQIAPGQTGKIADYNYKLPDDFTGCV

PNFKDQVILLNKHIDAYKTFPPTEPKKDKKKKADETQALPQRQKKQQTVTLLPAADLDDFSKQLQQSMSSAD
NCVADYSVLYNSASFSTFKCYGVSPTKLNDLCFTNVYADSFVIRGDEVRQIAPGQTGKIADYNYKLPDDFTGCV

PNFKDQVILLNKHIDAYKTFPPTEPKKDKKKKADETQALPQRQKKQQTVTLLPAADLDDFSKQLQQSMSSAD
NCVADYSVLYNSASFSTFKCYGVSPTKLNDLCFTNVYADSFVIRGDEVRQIAPGQTGKIADYNYKLPDDFTGCV

PNFKDQVILLNKHIDAYKTFPPTEPKKDKKKKADETQALPQRQKKQQTVTLLPAADLDDFSKQLQQSMSSAD
NCVADYSVLYNSASFSTFKCYGVSPTKLNDLCFTNVYADSFVIRGDEVRQIAPGQTGKIADYNYKLPDDFTGCV

PNFKDQVILLNKHIDAYKTFPPTEPKKDKKKKADETQALPQRQKKQQTVTLLPAADLDDFSKQLQQSMSSAD

PNFKDQVILLNKHIDAYKTFPPTEPKKDKKKKADETQALPQRQKKQQTVTLLPAADLDDFSKQLQQSMSSAD

PNFKDQVILLNKHIDAYKTFPPTEPKKDKKKKADETQALPQRQKKQQTVTLLPAADLDDFSKQLQQSMSSAD

PNFKDQVILLNKHIDAYKTFPPTEPKKDKKKKADETQALPQRQKKQQTVTLLPAADLDDFSKQLQQSMSSAD

PNFKDQVILLNKHIDAYKTFPPTEPKKDKKKKADETQALPQRQKKQQTVTLLPAADLDDFSKQLQQSMSSAD

IAWNSNNLDSKVGGNYNYLYRLFRKSNLKPFERDISTEIYQAGSTPCNGVEGFNCYFPLQSYGFQPTNGVGYC

STQA*
IAWNSNNLDSKVGGNYNYLYRLFRKSNLKPFERDISTEIYQAGSTPCNGVEGFNCYFPLQSYGFQPTNGVGYC

STQA*
IAWNSNNLDSKVGGNYNYLYRLFRKSNLKPFERDISTEIYQAGSTPCNGVEGFNCYFPLQSYGFQPTNGVGYC

STQA*
IAWNSNNLDSKVGGNYNYLYRLFRKSNLKPFERDISTEIYQAGSTPCNGVEGFNCYFPLQSYGFQPTNGVGYC

STQA*
IAWNSNNLDSKVGGNYNYLYRLFRKSNLKPFERDISTEIYQAGSTPCNGVEGFNCYFPLQSYGFQPTNGVGYC

STQA*
IAWNSNNLDSKVGGNYNYLYRLFRKSNLKPFERDISTEIYQAGSTPCNGVEGFNCYFPLQSYGFQPTNGVGYC

STQA*

STQA*

STQA*

STQA*

STQA*

QPYRVVVLSFELLHAPATVCGPKKSTNLVKNKCVNFNFNGLTGTGVLTESNKKFLPFQQFGRDIADTTDAVRD

QPYRVVVLSFELLHAPATVCGPKKSTNLVKNKCVNFNFNGLTGTGVLTESNKKFLPFQQFGRDIADTTDAVRD

QPYRVVVLSFELLHAPATVCGPKKSTNLVKNKCVNFNFNGLTGTGVLTESNKKFLPFQQFGRDIADTTDAVRD

QPYRVVVLSFELLHAPATVCGPKKSTNLVKNKCVNFNFNGLTGTGVLTESNKKFLPFQQFGRDIADTTDAVRD

QPYRVVVLSFELLHAPATVCGPKKSTNLVKNKCVNFNFNGLTGTGVLTESNKKFLPFQQFGRDIADTTDAVRD

QPYRVVVLSFELLHAPATVCGPKKSTNLVKNKCVNFNFNGLTGTGVLTESNKKFLPFQQFGRDIADTTDAVRD

PQTLEILDITPCSFGGVSVITPGTNTSNQVAVLYQDVNCTEVPVAIHADQLTPTWRVYSTGSNVFQTRAGCLIG

PQTLEILDITPCSFGGVSVITPGTNTSNQVAVLYQDVNCTEVPVAIHADQLTPTWRVYSTGSNVFQTRAGCLIG

PQTLEILDITPCSFGGVSVITPGTNTSNQVAVLYQDVNCTEVPVAIHADQLTPTWRVYSTGSNVFQTRAGCLIG

PQTLEILDITPCSFGGVSVITPGTNTSNQVAVLYQDVNCTEVPVAIHADQLTPTWRVYSTGSNVFQTRAGCLIG

PQTLEILDITPCSFGGVSVITPGTNTSNQVAVLYQDVNCTEVPVAIHADQLTPTWRVYSTGSNVFQTRAGCLIG

PQTLEILDITPCSFGGVSVITPGTNTSNQVAVLYQDVNCTEVPVAIHADQLTPTWRVYSTGSNVFQTRAGCLIG

GAEHVNNSYECDIPIGAGICASYQTQTNSPRRARSVASQSIIAYTMSLGAENSVAYSNNSIAIPTNFTISVTTEILP

GAEHVNNSYECDIPIGAGICASYQTQTNSPRRARSVASQSIIAYTMSLGAENSVAYSNNSIAIPTNFTISVTTEILP

GAEHVNNSYECDIPIGAGICASYQTQTNSPRRARSVASQSIIAYTMSLGAENSVAYSNNSIAIPTNFTISVTTEILP

GAEHVNNSYECDIPIGAGICASYQTQTNSPRRARSVASQSIIAYTMSLGAENSVAYSNNSIAIPTNFTISVTTEILP

GAEHVNNSYECDIPIGAGICASYQTQTNSPRRARSVASQSIIAYTMSLGAENSVAYSNNSIAIPTNFTISVTTEILP

GAEHVNNSYECDIPIGAGICASYQTQTNSPRRARSVASQSIIAYTMSLGAENSVAYSNNSIAIPTNFTISVTTEILP

VSMTKTSVDCTMYICGDSTECSNLLLQYGSFCTQLNRALTGIAVEQDKNTQEVFAQVKQIYKTPPIKDFGGF

VSMTKTSVDCTMYICGDSTECSNLLLQYGSFCTQLNRALTGIAVEQDKNTQEVFAQVKQIYKTPPIKDFGGF

VSMTKTSVDCTMYICGDSTECSNLLLQYGSFCTQLNRALTGIAVEQDKNTQEVFAQVKQIYKTPPIKDFGGF

VSMTKTSVDCTMYICGDSTECSNLLLQYGSFCTQLNRALTGIAVEQDKNTQEVFAQVKQIYKTPPIKDFGGF

VSMTKTSVDCTMYICGDSTECSNLLLQYGSFCTQLNRALTGIAVEQDKNTQEVFAQVKQIYKTPPIKDFGGF

VSMTKTSVDCTMYICGDSTECSNLLLQYGSFCTQLNRALTGIAVEQDKNTQEVFAQVKQIYKTPPIKDFGGF

NFSQILPDPSKPSKRSFIEDLLFNKVTLADAGFIKQYGDCLGDIAARDLICAQKFNGLTVLPPLLTDEMIAQYTSAL

NFSQILPDPSKPSKRSFIEDLLFNKVTLADAGFIKQYGDCLGDIAARDLICAQKFNGLTVLPPLLTDEMIAQYTSAL

NFSQILPDPSKPSKRSFIEDLLFNKVTLADAGFIKQYGDCLGDIAARDLICAQKFNGLTVLPPLLTDEMIAQYTSAL

NFSQILPDPSKPSKRSFIEDLLFNKVTLADAGFIKQYGDCLGDIAARDLICAQKFNGLTVLPPLLTDEMIAQYTSAL

NFSQILPDPSKPSKRSFIEDLLFNKVTLADAGFIKQYGDCLGDIAARDLICAQKFNGLTVLPPLLTDEMIAQYTSAL

NFSQILPDPSKPSKRSFIEDLLFNKVTLADAGFIKQYGDCLGDIAARDLICAQKFNGLTVLPPLLTDEMIAQYTSAL

LAGTITSGWTFGAGAALQIPFAMQMAYRFNGIGVTQNVLYENQKLIANQFNSAIGKIQDSLSSTASALGKLQ

LAGTITSGWTFGAGAALQIPFAMQMAYRFNGIGVTQNVLYENQKLIANQFNSAIGKIQDSLSSTASALGKLQ

LAGTITSGWTFGAGAALQIPFAMQMAYRFNGIGVTQNVLYENQKLIANQFNSAIGKIQDSLSSTASALGKLQ

LAGTITSGWTFGAGAALQIPFAMQMAYRFNGIGVTQNVLYENQKLIANQFNSAIGKIQDSLSSTASALGKLQ

LAGTITSGWTFGAGAALQIPFAMQMAYRFNGIGVTQNVLYENQKLIANQFNSAIGKIQDSLSSTASALGKLQ

LAGTITSGWTFGAGAALQIPFAMQMAYRFNGIGVTQNVLYENQKLIANQFNSAIGKIQDSLSSTASALGKLQ

DVVNQNAQALNTLVKQLSSNFGAISSVLNDILSRLDKVEAEVQIDRLITGRLQSLQTYVTQQLIRAAEIRASAN

DVVNQNAQALNTLVKQLSSNFGAISSVLNDILSRLDKVEAEVQIDRLITGRLQSLQTYVTQQLIRAAEIRASAN

DVVNQNAQALNTLVKQLSSNFGAISSVLNDILSRLDKVEAEVQIDRLITGRLQSLQTYVTQQLIRAAEIRASAN

DVVNQNAQALNTLVKQLSSNFGAISSVLNDILSRLDKVEAEVQIDRLITGRLQSLQTYVTQQLIRAAEIRASAN

DVVNQNAQALNTLVKQLSSNFGAISSVLNDILSRLDKVEAEVQIDRLITGRLQSLQTYVTQQLIRAAEIRASAN

DVVNQNAQALNTLVKQLSSNFGAISSVLNDILSRLDKVEAEVQIDRLITGRLQSLQTYVTQQLIRAAEIRASAN

L

L

L

L

L

L

	subgenome	leader_end	TRS_start	nb_count
S1-Day1	1	74	21058	5381(5374,5368,5361,5332)
S2-Day2	1	46	26422	13(13,13,13,12)
	2	48	26443	22(22,22,22,22)
S3-Day3	1	46	26422	25(25,25,25,24)
	2	58	26917	24(23,0,0,0)
	3	63	5785	146(146,140,140,136)
	4	65	10377	16(16,0,0,0)
	5	65	10595	27(27,12,12,12)
	6	47	26421	19(19,19,19,18)
	7	68	5789	155(155,146,146,146)
	8	69	3816	76(76,0,0,6)
	9	69	13619	117(117,112,112,110)
	10	69	19112	303(299,300,296,290)
	11	70	13426	35(31,0,0,4)
	12	70	14372	39(38,1,0,2)
	13	48	26443	109(109,109,109,104)
	14	70	17878	27(27,24,24,24)
	15	70	22502	55(53,0,0,6)
	16	71	5594	152(152,0,0,0)
	17	71	5708	103(56,47,0,4)
	18	71	22409	69(69,0,0,0)
	19	74	15866	360(360,0,0,4)
	20	74	21058	84(84,82,82,82)
S1-Day4	1	46	26422	18(18,18,18,18)
	2	68	4580	453(450,439,436,440)
	3	68	15777	434(433,5,4,6)
	4	70	22502	151(151,0,0,4)
	5	73	23098	1199(1194,2,2,16)
S2-Day5				
S3-Day6	1	46	26422	14(14,14,14,14)
	2	59	24304	13(13,0,0,0)
	3	67	22503	304(303,1,1,6)
	4	68	2689	113(94,112,93,84)
	5	71	22409	669(669,0,0,6)
	6	74	21058	1796(1793,1790,1787,1770)
	7	48	26443	40(40,40,40,40)
	8	48	28184	22(22,0,0,0)
	9	52	28249	384(293,382,291,2)
S2-Day8	1	58	29871	17(0,0,0,0)
S3-Day9				
S2-Day11	1	76	28267	13(12,12,11,0)
	2	78	28269	29(29,29,29,0)

S3-Day12				
S3-Day19	1	58	29871	124(41,0,0,22)

The numbers in the bracket are (reads with left primers, reads with right primers, rea

Normalized count=(Read count/Total number of read mapped on reference genome

Total number of read mapped on reference genome is 32903734, 33694953, 33400[illegible]

“leader_end” and “TRS_start” refer to the position of the end of leader and the posi[illegible]

normalized_count
163.54(163.32,163.14,162.93,162.05)
0.39(0.39,0.39,0.39,0.36)
0.65(0.65,0.65,0.65,0.65)
0.75(0.75,0.75,0.75,0.72)
0.72(0.69,0.00,0.00,0.00)
4.37(4.37,4.19,4.19,4.07)
0.48(0.48,0.00,0.00,0.00)
0.81(0.81,0.36,0.36,0.36)
0.57(0.57,0.57,0.57,0.54)
4.64(4.64,4.37,4.37,4.37)
2.28(2.28,0.00,0.00,0.18)
3.50(3.50,3.35,3.35,3.29)
9.07(8.95,8.98,8.86,8.68)
1.05(0.93,0.00,0.00,0.12)
1.17(1.14,0.03,0.00,0.06)
3.26(3.26,3.26,3.26,3.11)
0.81(0.81,0.72,0.72,0.72)
1.65(1.59,0.00,0.00,0.18)
4.55(4.55,0.00,0.00,0.00)
3.08(1.68,1.41,0.00,0.12)
2.07(2.07,0.00,0.00,0.00)
10.78(10.78,0.00,0.00,0.12)
2.51(2.51,2.46,2.46,2.46)
0.55(0.55,0.55,0.55,0.55)
13.86(13.77,13.43,13.34,13.46)
13.28(13.25,0.15,0.12,0.18)
4.62(4.62,0.00,0.00,0.12)
36.68(36.53,0.06,0.06,0.49)

0.43(0.43,0.43,0.43,0.43)
0.40(0.40,0.00,0.00,0.00)
9.25(9.22,0.03,0.03,0.18)
3.44(2.86,3.41,2.83,2.56)
20.36(20.36,0.00,0.00,0.18)
54.65(54.56,54.47,54.38,53.86)
1.22(1.22,1.22,1.22,1.22)
0.67(0.67,0.00,0.00,0.00)
11.68(8.92,11.62,8.85,0.06)
1.26(0.00,0.00,0.00,0.00)

1.31(1.21,1.21,1.11,0.00)
2.93(2.93,2.93,2.93,0.00)

7.92(2.62,0.00,0.00,1.41)

ads with both primers, same junction on paired reads with at least a primer).

e)*1000000.

485, 32686824, 17052923, 32864351, 13447115, 15951471, 9892701, 13862861, 12570298 and 15

tion of the start of TRS identified in the reads >10.

653045, excluding the mapped reads unpaired, not primary alignment and supplementary align

ment.

	subgenome	leader_end	TRS_start	nb_count	normalized_count
S1-Day1	1	65	8436	64(64,8,8,56)	1.57(1.57,0.20,0.20,1.37)
	2	65	10766	18(18,0,0,0)	0.44(0.44,0.00,0.00,0.00)
	3	68	15777	573(573,0,0,8	14.06(14.06,0.00,0.00,0.20)
	4	71	10772	368(368,0,0,4	9.03(9.03,0.00,0.00,0.10)
S2-Day2	1	46	26422	23(23,23,23,2	0.66(0.66,0.66,0.66,0.64)
	2	47	26421	14(14,14,14,1	0.40(0.40,0.40,0.40,0.40)
	3	75	26486	2311(2307,21	66.78(66.66,62.67,62.61,64
	4	48	26443	34(34,34,34,3	0.98(0.98,0.98,0.98,0.98)
S3-Day3	1	62	3808	12(12,0,0,0)	0.37(0.37,0.00,0.00,0.00)
	2	63	23922	72(64,8,0,0)	2.24(1.99,0.25,0.00,0.00)
	3	66	14252	21(21,20,20,1	0.65(0.65,0.62,0.62,0.56)
	4	67	21083	84(84,84,84,8	2.61(2.61,2.61,2.61,2.61)
	5	69	3816	133(133,0,0,6	4.13(4.13,0.00,0.00,0.19)
	6	69	7186	224(224,4,4,2	6.96(6.96,0.12,0.12,0.06)
	7	69	18554	197(197,182,	6.12(6.12,5.66,5.66,6.03)
	8	71	8201	212(212,206,	6.59(6.59,6.40,6.40,6.40)
	9	48	26443	14(14,14,14,1	0.44(0.44,0.44,0.44,0.44)
	10	54	11370	28(19,0,0,0)	0.87(0.59,0.00,0.00,0.00)
S1-Day4	1	46	26422	14(14,14,14,1	0.43(0.43,0.43,0.43,0.43)
	2	71	5594	1610(1610,2,	49.87(49.87,0.06,0.06,0.50)
	3	76	28267	12(12,11,11,4	0.37(0.37,0.34,0.34,0.12)
	4	48	26443	28(28,28,28,2	0.87(0.87,0.87,0.87,0.87)
	5	48	28184	18(18,0,0,0)	0.56(0.56,0.00,0.00,0.00)
S2-Day5	1	48	28184	15(15,0,0,0)	0.52(0.52,0.00,0.00,0.00)
S3-Day6	1	46	26422	20(20,20,20,2	0.67(0.67,0.67,0.67,0.67)
	2	71	27762	29(29,10,10,1	0.96(0.96,0.33,0.33,0.40)
S2-Day8					
S3-Day9	1	57	12988	31(29,30,28,1	1.68(1.57,1.63,1.52,0.76)
	2	58	12989	20151(19897	1093.56(1079.77,1041.35,1
S2-Day11					
S3-Day12					
S3-Day15					

The numbers in the bracket are (reads with left primers, reads with right primers, reads with bot
Normalized count=(Read count/Total number of read mapped on reference genome)*1000000.
Total number of read mapped on reference genome is 40751196, 34608693, 32168622, 322809
“leader_end” and “TRS_start” refer to the position of the end of leader and the position of the s

)

.43)

)

028.06,1045.20)

h primers, same junction on paired reads with at least a primer).

32, 28595139, 30056513, 29481101, 18427015, 17677974, 11329365, 10742292 and 12786655,
tart of TRS identified in the reads >10.

excluding the mapped reads unpaired, not primary alignment and supplementary alignment.

	subgenome	leader_end	TRS_start	ACGAAC	ATG_postion
S1-Day1	1	74	21058	no	21071
S2-Day2	1	46	26422	yes	26523
	2	48	26443	yes	26523
S3-Day3	1	46	26422	yes	26523
	2	58	26917	yes	27202
	3	63	5785	no	5859
	4	65	10377	no	10410
	5	65	10595	no	10599
	6	47	26421	yes	26523
	7	68	5789	no	5859
	8	69	3816	no	3842
	9	69	13619	no	13625
	10	69	19112	no	19148
	11	70	13426	no	13433
	12	70	14372	no	14381
	13	48	26443	yes	26523
	14	70	17878	no	17906
	15	70	22502	no	22614
	16	71	5594	no	5681
	17	71	5708	no	5718
	18	71	22409	no	22422
	19	74	15866	no	15869
	20	74	21058	no	21071
S1-Day4	1	46	26422	yes	26523
	2	68	4580	no	4590
	3	68	15777	no	15812
	4	70	22502	no	22614
	5	73	23098	no	23118
S2-Day5					
S3-Day6	1	46	26422	yes	26523
	2	59	24304	no	24312
	3	67	22503	no	22614
	4	68	2689	no	2745
	5	71	22409	no	22422
	6	74	21058	no	21071
	7	48	26443	yes	26523
	8	48	28184	no	28207
	9	52	28249	yes	28274
S2-Day8	1	58	29871	-	-
S3-Day9					
S2-Day11	1	76	28267	no	28274
	2	78	28269	no	28274

S3-Day12					
S3-Day19	1	58	29871	-	-

“peak_leader” and “peak_TRS_start” point to the leader-TRS junctions in sheet "novel", “ACGAA[illegible]

known_ATG	20_leader_se	TRS_seq	first_orf_aa
-	AGATCTGTTC	CCTAAGACTA	MLQKKMTLKRVFSLTFVGLYNKS*
M	ACAAACCAA	TCTCGTGTTA	MADSNGTITVEELKKLLEQWNLVIGFLFLTW
M	AAACCAACCA	TCTTCTAGAG	MADSNGTITVEELKKLLEQWNLVIGFLFLTW
M	ACAAACCAA	TCTCGTGTTA	MADSNGTITVEELKKLLEQWNLVIGFLFLTW
ORF6	ACTTTCGATC	CGCTTCTAGA	MFHLVDFQVTIAEILLIIMRTFKVSIWNLDYIII
-	CGATCTCTTG	TTCTAAAGAA	MFSTKKTVTQQP*
-	ATCTCTTGTA	CAGGACAGA	MVHHLVFTNVL*
-	ATCTCTTGTA	TTTT	MDLLLTGKQHKQLVRTQLLQLMF*
M	CAAACCAAC	CTCTCGTGTT	MADSNGTITVEELKKLLEQWNLVIGFLFLTW
-	TCTTGTAGAT	AAAGAAACTT	MFSTKKTVTQQP*
-	CTTGTAGATC	ACAAACTTGT	MKSEKQVEQKIAEIPKEEVKPFITESKPSVEQ
-	CTTGTAGATC	ACGAAG	MTI*
-	CTTGTAGATC	ACAAAGCTTA	MPHILTNSQMVYAYFGIAMSIDILLIPLFVDL
-	TTGTAGATCT	CGAACCC	MLQSADAQSFLNGFAV*
-	TTGTAGATCT	CAAACTTTA	MFYSLQCSHLQVLDH*
M	AAACCAACCA	TCTTCTAGAG	MADSNGTITVEELKKLLEQWNLVIGFLFLTW
-	TTGTAGATCT	CAAACCACTG	M*
-	TTGTAGATCT	CAAACTTCTA	MLGTGRESATVLLIILSYIIPHHFPLLSVMECLL
-	TGTAGATCTG	GAACAATTTA	MMSAPPAQYELKHGTFTCASEYTGNYQCG
-	TGTAGATCTG	GAACTTAAG	MVHLLVLVSTLVITSVVTINI*
-	TGTAGATCTG	GGAACCATTA	ML*
-	AGATCTGTTC	CTC	MNFALNIQC*
-	AGATCTGTTC	CCTAAGACTA	MLQKKMTLKRVFSLTFVGLYNKS*
M	ACAAACCAA	TCTCGTGTTA	MADSNGTITVEELKKLLEQWNLVIGFLFLTW
-	TCTTGTAGAT	AACGATCTAA	MKLLLQCHLAM*
-	TCTTGTAGAT	AAAGAACTTT	MFLCLKQNVGLRLTLLKDLMNFALNIQC*
-	TTGTAGATCT	CAAACTTCTA	MLGTGRESATVLLIILSYIIPHHFPLLSVMECLL
-	TAGATCTGTT	ACTTTCTTTT	MHQQLFVDLKSLLIWLKTNVSISTSMV*
M	ACAAACCAA	TCTCGTGTTA	MADSNGTITVEELKKLLEQWNLVIGFLFLTW
-	CTTTCGATCT	TGTTCTCT	MRTKN*
-	CTCTTGTAGA	AAACTTCTAA	MLGTGRESATVLLIILSYIIPHHFPLLSVMECLL
-	TCTTGTAGAT	AACAAACAAT	MTL*
-	TGTAGATCTG	GGAACCATTA	ML*
-	AGATCTGTTC	CCTAAGACTA	MLQKKMTLKRVFSLTFVGLYNKS*
M	AAACCAACCA	TCTTCTAGAG	MADSNGTITVEELKKLLEQWNLVIGFLFLTW
-	AAACCAACCA	TCTTGTAGTG	MKTF*
N	CAACCAACTT	ATTTCATCTA	MSDNGPQNQRNAPRITFGGPSDSTGSNQN
-	-	-	-
N	ATCTGTTCTC	AACTAAA	MSDNGPQNQRNAPRITFGGPSDSTGSNQN
N	CTGTTCTCTA	CTAAA	MSDNGPQNQRNAPRITFGGPSDSTGSNQN

- - - -

:” indicates if there is a ACGAAC sequences in the “TRS_seq” (TRS sequences), “20_leader_seq” r

ICLLQFAYANRNRFLYIIKLIFLWLLWPVTLACFVLAAVYRINWITGGIAIAMACLVGLMWLSYFIASFRLFARTF
ICLLQFAYANRNRFLYIIKLIFLWLLWPVTLACFVLAAVYRINWITGGIAIAMACLVGLMWLSYFIASFRLFARTF
ICLLQFAYANRNRFLYIIKLIFLWLLWPVTLACFVLAAVYRINWITGGIAIAMACLVGLMWLSYFIASFRLFARTF
NLIIKNLSKSLTENKYSQLDEEQPMEID*

ICLLQFAYANRNRFLYIIKLIFLWLLWPVTLACFVLAAVYRINWITGGIAIAMACLVGLMWLSYFIASFRLFARTF

RKQDDKKIKACVEEVTTTLEETKFLTENLLLYIDINGNLHPDSATLVSDIDITFLKKDAPYIVGDVVQEGVLTAVVI

TLECYLTLTLTCLVVMVAVCM*

ICLLQFAYANRNRFLYIIKLIFLWLLWPVTLACFVLAAVYRINWITGGIAIAMACLVGLMWLSYFIASFRLFARTF

LN*
HYKHITSKETLYCIDGALLTKSSEYKGPITDVFYKENSYTTTIKPVTYKLDGVVCTEIDPKLDNYYKKDNSYFTEQPI

ICLLQFAYANRNRFLYIIKLIFLWLLWPVTLACFVLAAVYRINWITGGIAIAMACLVGLMWLSYFIASFRLFARTF

LN*

ICLLQFAYANRNRFLYIIKLIFLWLLWPVTLACFVLAAVYRINWITGGIAIAMACLVGLMWLSYFIASFRLFARTF

LN*

ICLLQFAYANRNRFLYIIKLIFLWLLWPVTLACFVLAAVYRINWITGGIAIAMACLVGLMWLSYFIASFRLFARTF

IGERSGARSKQRRPQGLPNNTASWFTALTQHGKEDLKFPRGQGVPINTNSSPDDQIGYYRRATRRIRGGDGK

IGERSGARSKQRRPQGLPNNTASWFTALTQHGKEDLKFPRGQGVPINTNSSPDDQIGYYRRATRRIRGGDGK
IGERSGARSKQRRPQGLPNNTASWFTALTQHGKEDLKFPRGQGVPINTNSSPDDQIGYYRRATRRIRGGDGK

efers to the 20 sequences before the end of the leader, “AUG_postion” and “first_orf_aa” refer

RSMWSFNPETNILLNVPLHGTILTRPLLESELVIGAVILRGHLRIAGHHLGRCDIKDLPKEITVATSRTLSYYKLGAS
RSMWSFNPETNILLNVPLHGTILTRPLLESELVIGAVILRGHLRIAGHHLGRCDIKDLPKEITVATSRTLSYYKLGAS
RSMWSFNPETNILLNVPLHGTILTRPLLESELVIGAVILRGHLRIAGHHLGRCDIKDLPKEITVATSRTLSYYKLGAS

RSMWSFNPETNILLNVPLHGTILTRPLLESELVIGAVILRGHLRIAGHHLGRCDIKDLPKEITVATSRTLSYYKLGAS

PTKKAGGTTEMLAKALRKVPTDNYITTYPGQGLNGYTVEEAKTVLKKCKSAFYILPSIISNEKQEILGTVSWNL

RSMWSFNPETNILLNVPLHGTILTRPLLESELVIGAVILRGHLRIAGHHLGRCDIKDLPKEITVATSRTLSYYKLGAS

IDLVPNQPYPNASFDNFKFVCDNIKFADDLNQLTGYKKPASRELKVTFFPDLNGDVVAIDYKHYTPSFKKGAKL

RSMWSFNPETNILLNVPLHGTILTRPLLESELVIGAVILRGHLRIAGHHLGRCDIKDLPKEITVATSRTLSYYKLGAS

RSMWSFNPETNILLNVPLHGTILTRPLLESELVIGAVILRGHLRIAGHHLGRCDIKDLPKEITVATSRTLSYYKLGAS

RSMWSFNPETNILLNVPLHGTILTRPLLESELVIGAVILRGHLRIAGHHLGRCDIKDLPKEITVATSRTLSYYKLGAS

MKDLSPRWYFYYLGTGPEAGLPYGANKDGIIWVATEGALNTPKDHIGTRNPANNAAIVLQLPQGTTLPKGFY

MKDLSPRWYFYYLGTGPEAGLPYGANKDGIIWVATEGALNTPKDHIGTRNPANNAAIVLQLPQGTTLPKGFY
MKDLSPRWYFYYLGTGPEAGLPYGANKDGIIWVATEGALNTPKDHIGTRNPANNAAIVLQLPQGTTLPKGFY

to the first AUG position and translated orf of the sgmRNA, and “known_ATG” indicates if the f

SQRVAGDSGFAAYSRYRIGNYKLNTDHSSSSDNIALLVQ*
SQRVAGDSGFAAYSRYRIGNYKLNTDHSSSSDNIALLVQ*
SQRVAGDSGFAAYSRYRIGNYKLNTDHSSSSDNIALLVQ*

SQRVAGDSGFAAYSRYRIGNYKLNTDHSSSSDNIALLVQ*

REMLAHAEETRKLMPVCVETKAIVSTIQRKYKGIKIQEGVVDYGARFYFYTSKTTVASLINTLNDLNETLVTMPL

SQRVAGDSGFAAYSRYRIGNYKLNTDHSSSSDNIALLVQ*

LHKPIVWHVNNATNKATYKPNTWCIRCLWSTKPVETSNSFDVLKSEDAQGMDNLACEDLKPVSEEVVENPT

SQRVAGDSGFAAYSRYRIGNYKLNTDHSSSSDNIALLVQ*

SQRVAGDSGFAAYSRYRIGNYKLNTDHSSSSDNIALLVQ*

SQRVAGDSGFAAYSRYRIGNYKLNTDHSSSSDNIALLVQ*

YAEGSRGGSQASSRSSSRSRNSSRNSTPGSSRGTSPARMAGNGGDAALALLLLDRLNQLESKMSGKGQQQQ

YAEGSRGGSQASSRSSSRSRNSSRNSTPGSSRGTSPARMAGNGGDAALALLLLDRLNQLESKMSGKGQQQQ
YAEGSRGGSQASSRSSSRSRNSSRNSTPGSSRGTSPARMAGNGGDAALALLLLDRLNQLESKMSGKGQQQQ

irst AUG position is the same as a known sgmRNA.

GYVTHGLNLEEAARYMRSLKVPATVSVSSPDAVTAYNGYLTSSSKTPEEHFIETISLAGSYKDWSYSGQSTQLG

TIQKDVLECNVKTTEVVGDIILKPANNSLKITEEVGHTDLMAAYVDNSSLTIKKPNELSRVLGLKTLATHGLAAV

GQTVTKKSAAEASKKPRQKRTATKAYNVTQAFGRRGPEQTQGNFGDQELIRQGTDYKHWPQIAQFAPSASA

GQTVTKKSAAEASKKPRQKRTATKAYNVTQAFGRRGPEQTQGNFGDQELIRQGTDYKHWPQIAQFAPSASA
GQTVTKKSAAEASKKPRQKRTATKAYNVTQAFGRRGPEQTQGNFGDQELIRQGTDYKHWPQIAQFAPSASA

IEFLKRGDKSVYYTSNPTTFHLDGEVITFDNLKTLLSLREVRTIKVFTTVDNINLHTQVVDMSMTYGQQFGPTY

NSVPWDTIANYAKPFLNKVVSTTTNIVTRCLNRVCTNYMPYFFTLLLQLCTFTRSTNSRIKASMPTTIAKNTVKS

AFFGMSRIGMEVTPSGTWLTYTGAIKLDDKDPNFKDQVILLNKHIDAYKTFPPTEPKKDKKKKADETQALPQR

AFFGMSRIGMEVTPSGTWLTYTGAIKLDDKDPNFKDQVILLNKHIDAYKTFPPTEPKKDKKKKADETQALPQR

AFFGMSRIGMEVTPSGTWLTYTGAIKLDDKDPNFKDQVILLNKHIDAYKTFPPTEPKKDKKKKADETQALPQR

LDGADVTKIKPHNSHEGKTFYVLPNDDTLRVEAFEYYHTTDPSFLGRYMSALNHTKKWKYPQVNGLTSIKWA

SVGKFCLEASFNYLKSPNFSKLINIIIWFLLLSVCLGSLIYSTAALGVLMSNLGMPSYCTGYREGYLNSTNVTIATY[illegible]

QKKQQTVTLLPAADLDDFSKQLQQSMSSADSTQA*

QKKQQTVTLLPAADLDDFSKQLQQSMSSADSTQA*
QKKQQTVTLLPAADLDDFSKQLQQSMSSADSTQA*

DNNCYLATALLTLQQIELKFNPPALQDAYYRARAGEAANFCALILAYCNKTVGELGDVRETMSYLFQHANLD

TGSIPCSVCLSGLDSLDTYPSLETIQITISSFKWDLTAFGLVAEWFLAYILFTRFFYVLGLAAIMQLFFSYFAVHFI

SCKRVLNVVCKTCGQQQTTLKGVEAVMYMGTLSYEQFKKGVQIPCTCGKQATKYLVQQESPFVMMSAPPA[illegible]

SNSWLMWLIINLVQMAPISAMVRMYIFFASFYYVWKSYVHVVDGCNSSTCMMCYKRNRATRVECTTIVNG

QYELKHGTFTCASEYTGNYQCGHYKHITSKETLYCIDGALLTKSSEYKGPITDVFYKENSYTTTIKPVTYKLDGVV[illegible]

VRRSFYVYANGGKGFCKLHNWNCVNCDTFCAGSTFISDEVARDLSLQFKRPINPTDQSSYIVDSVTVKNGSIH

CTEIDPKLDNYYKKDNSYFTEQPIDLVPNQPYPNASFDNFKFVCDNIKFADDLNQLTGYKKPASRELKVTFFPD

LYFDKAGQKTYERHSLSHFVNLDNLRANNTKGSLPINVIVFDGKSKCEESSAKSASVYYSQLMCQPILLLDQAL[illegible]

LNGDVVAIDYKHYTPSFKKGAKLLHKPIVWHVNNATNKATYKPNTWCIRCLWSTKPVETSNSFDVLKSEDAQ

VSDVGDSAEVAVKMFDAYVNTFSSTFNVPMEKLKTLVATAEAELAKNVSLDNVLSTFISAARQGFVDSDVETI

[illegible]GMDNLACEDLKPVSEEVVENPTIQKDVLECNVKTTEVVGDIILKPANNSLKITEEVGHTDLMAAYVDNSSLTI[illegible]

[illegible]DVVECLKLSHQSDIEVTGDSCNNYMLTYNKVENMTPRDLGACIDCSARHINAQVAKSHNIALIWNVKDFM[illegible]

[illegible]KPNELSRVLGLKTLATHGLAAVNSVPWDTIANYAKPFLNKVVSTTTNIVTRCLNRVCTNYMPYFFTLLLQLCT[illegible]

[illegible]LSEQLRKQIRSAAKKNNLPFKLTCATTRQVVNVVTTKIALKGGKIVNNWLKQLIKVTLVFLFVAAIFYLITPVHV

FTRSTNSRIKASMPTTIAKNTVKSVGKFCLEASFN

MSKHTDFSSEIIGYKAIDGGVTRDIASTDTCFAN

	subgenome	leader_end	TRS_start	ACGAAC	ATG_postion	known_ATG
S1-Day1	1	65	8436	no	8494	-
	2	65	10766	no	10770	-
	3	68	15777	no	15812	-
	4	71	10772	no	10791	-
S2-Day2	1	46	26422	yes	26523	M
	2	47	26421	yes	26523	M
	3	75	26486	no	26523	M
	4	48	26443	yes	26523	M
S3-Day3	1	62	3808	no	3813	-
	2	63	23922	no	24051	-
	3	66	14252	no	14258	-
	4	67	21083	no	21086	-
	5	69	3816	no	3842	-
	6	69	7186	no	7210	-
	7	69	18554	no	18594	-
	8	71	8201	no	8214	-
	9	48	26443	yes	26523	M
	10	54	11370	no	11370	-
S1-Day4	1	46	26422	yes	26523	M
	2	71	5594	no	5681	-
	3	76	28267	no	28274	N
	4	48	26443	yes	26523	M
	5	48	28184	no	28207	-
S2-Day5	1	48	28184	no	28207	-
S3-Day6	1	46	26422	yes	26523	M
	2	71	27762	no	27825	-
S2-Day8						
S3-Day9	1	57	12988	no	13032	-
	2	58	12989	no	13032	-
S2-Day11						
S3-Day12						
S3-Day15						

“peak_leader” and “peak_TRS_start” point to the leader-TRS junctions in sheet "novel", “ACGAA

20_leader_se	TRS_seq	first_orf_aa
ATCTCTTGTA	AACTACGAAA	MCNY*
ATCTCTTGTA	AATT	MNL*
TCTTGTAGAT	AAAGAACTTT	MFLCLKQNVGLRLTLLKDLMNFALNIQC*
TGTAGATCTG	GAACCTCTAA	MLTY*
ACAAACCAAC	TCTCGTGTTA	MADSNGTITVEELKKLLEQWNLVIGFLFLTWICLLQFAYANRNRFLYIIKLIFLW
CAAACCAACC	CTCTCGTGTT	MADSNGTITVEELKKLLEQWNLVIGFLFLTWICLLQFAYANRNRFLYIIKLIFLW
GATCTGTTCT	TATATTAGTT	MADSNGTITVEELKKLLEQWNLVIGFLFLTWICLLQFAYANRNRFLYIIKLIFLW
AAACCAACCA	TCTTCTAGAG	MADSNGTITVEELKKLLEQWNLVIGFLFLTWICLLQFAYANRNRFLYIIKLIFLW
TCGATCTCTT	TCTCT	MTNLFQAFWK*
CGATCTCTTG	AAATTTACAA	MLASSNNMVIALVILLLETSFVHKSLTALLFCHLCSQMK*
TCTCTTGTAG	TAAAAT	MTSRKRG*
CTCTTGTAGA	AAA	MTLKRVFSLTFVGLYNKS*
CTTGTAGATC	ACAAACTTGT	MKSEKQVEQKIAEIPKEEVKPFITESKPSVEQRKQDDKKIKACVEEVTTTLEETI
CTTGTAGATC	ACAAATTACC	MGFNCFWLSCRVVFGIYSFH*
CTTGTAGATC	ACACACTTAA	MGTWL*
TGTAGATCTG	GTAGAAACTA	MLLNVLNCHINLT*
AAACCAACCA	TCTTCTAGAG	MADSNGTITVEELKKLLEQWNLVIGFLFLTWICLLQFAYANRNRFLYIIKLIFLW
ACCAACTTTC	-	MMVLGECGHL*
ACAAACCAAC	TCTCGTGTTA	MADSNGTITVEELKKLLEQWNLVIGFLFLTWICLLQFAYANRNRFLYIIKLIFLW
TGTAGATCTG	GAACAATTTA	MMSAPPAQYELKHGTFTCASEYTGNYQCGHYKHITSKETLYCIDGALLTKSSE
ATCTGTTCTC	AACTAAA	MSDNGPQNQRNAPRITFGGPSDSTGSNQNGERSGARSKQRRPQGLPNNTA
AAACCAACCA	TCTTCTAGAG	MADSNGTITVEELKKLLEQWNLVIGFLFLTWICLLQFAYANRNRFLYIIKLIFLW
AAACCAACCA	TCTTGTAGTG	MKTF*
AAACCAACCA	TCTTGTAGTG	MKTF*
ACAAACCAAC	TCTCGTGTTA	MADSNGTITVEELKKLLEQWNLVIGFLFLTWICLLQFAYANRNRFLYIIKLIFLW
TGTAGATCTG	GAACTTTCAT	MLIIFWFSLELQDHNETCHA*
AACTTTCGAT	GGTACTTGGT	MQQKCLPIQLYYLSVLLL*
ACTTTCGATC	GTACTTGGTA	MQQKCLPIQLYYLSVLLL*

C" indicates if there is a ACGAAC sequences in the "TRS_seq" (TRS sequences), "20_leader_seq"

LLWPVTLACFVLAAVYRINWITGGIAIAMACLVGLMWLSYFIASFRLFARTRSMWSFNPETNILLNVPLHGTIL
LLWPVTLACFVLAAVYRINWITGGIAIAMACLVGLMWLSYFIASFRLFARTRSMWSFNPETNILLNVPLHGTIL
LLWPVTLACFVLAAVYRINWITGGIAIAMACLVGLMWLSYFIASFRLFARTRSMWSFNPETNILLNVPLHGTIL
LLWPVTLACFVLAAVYRINWITGGIAIAMACLVGLMWLSYFIASFRLFARTRSMWSFNPETNILLNVPLHGTIL

FLTENLLLYIDINGNLHPDSATLVSDIDITFLKKDAPYIVGDVVQEGVLTAVVIPTKKAGGTTEMLAKALRKVPT

LLWPVTLACFVLAAVYRINWITGGIAIAMACLVGLMWLSYFIASFRLFARTRSMWSFNPETNILLNVPLHGTIL

LLWPVTLACFVLAAVYRINWITGGIAIAMACLVGLMWLSYFIASFRLFARTRSMWSFNPETNILLNVPLHGTIL
YKGPITDVFYKENSYTTTIKPVTYKLDGVVCTEIDPKLDNYYKKDNSYFTEQPIDLVPNQPYPNASFDNFKFVCD
SWFTALTQHGKEDLKFPRGQGVPINTNSSPDDQIGYYRRATRRIRGGDGKMKDLSPRWYFYYLGTGPEAGL
LLWPVTLACFVLAAVYRINWITGGIAIAMACLVGLMWLSYFIASFRLFARTRSMWSFNPETNILLNVPLHGTIL

LLWPVTLACFVLAAVYRINWITGGIAIAMACLVGLMWLSYFIASFRLFARTRSMWSFNPETNILLNVPLHGTIL

refers to the 20 sequences before the end of the leader, “AUG_postion” and “first_orf_aa” refer

TRPLLESELVIGAVILRGHLRIAGHHLGRCDIKDLPKEITVATSRTLSYYKLGASQRVAGDSGFAAYSRYRIGNYKL
TRPLLESELVIGAVILRGHLRIAGHHLGRCDIKDLPKEITVATSRTLSYYKLGASQRVAGDSGFAAYSRYRIGNYKL
TRPLLESELVIGAVILRGHLRIAGHHLGRCDIKDLPKEITVATSRTLSYYKLGASQRVAGDSGFAAYSRYRIGNYKL
TRPLLESELVIGAVILRGHLRIAGHHLGRCDIKDLPKEITVATSRTLSYYKLGASQRVAGDSGFAAYSRYRIGNYKL

NYITTYPGQGLNGYTVEEAKTVLKKCKSAFYILPSIISNEKQEILGTVSWNLREMLAHAEETRKLMPVCVETKAI

TRPLLESELVIGAVILRGHLRIAGHHLGRCDIKDLPKEITVATSRTLSYYKLGASQRVAGDSGFAAYSRYRIGNYKL

TRPLLESELVIGAVILRGHLRIAGHHLGRCDIKDLPKEITVATSRTLSYYKLGASQRVAGDSGFAAYSRYRIGNYKL
NIKFADDLNQLTGYKKPASRELKVTFFPDLNGDVVAIDYKHYTPSFKKGAKLLHKPIVWHVNNATNKATYKPNT
YGANKDGIIWVATEGALNTPKDHIGTRNPANNAAIVLQLPQGTTLPKGFYAEGSRGGSQASSRSSSRSRNSS
TRPLLESELVIGAVILRGHLRIAGHHLGRCDIKDLPKEITVATSRTLSYYKLGASQRVAGDSGFAAYSRYRIGNYKL

TRPLLESELVIGAVILRGHLRIAGHHLGRCDIKDLPKEITVATSRTLSYYKLGASQRVAGDSGFAAYSRYRIGNYKL

to the first AUG position and translated orf of the sgmRNA, and “known_ATG” indicates if the fi

NTDHSSSSDNIALLVQ*
NTDHSSSSDNIALLVQ*
NTDHSSSSDNIALLVQ*
NTDHSSSSDNIALLVQ*

VSTIQRKYKGIKIQEGVVDYGARFYFYTSKTTVASLINTLNDLNETLVTMPLGYVTHGLNLEEAARYMRSLKVPA

NTDHSSSSDNIALLVQ*

NTDHSSSSDNIALLVQ*
WCIRCLWSTKPVETSNSFDVLKSEDAQGMDNLACEDLKPVSEEVVENPTIQKDVLECNVKTTEVVGDIILKPA
NSTPGSSRGTSPARMAGNGGDAALALLLLDRLNQLESKMSGKGQQQQGQTVTKKSAAEASKKPRQKRTAT
NTDHSSSSDNIALLVQ*

NTDHSSSSDNIALLVQ*

rst AUG position is the same as a known sgmRNA.

TVSVSSPDAVTAYNGYLTSSSKTPEEHFIETISLAGSYKDWSYSGQSTQLGIEFLKRGDKSVYYTSNPTTFHLDGEV

NNSLKITEEVGHTDLMAAYVDNSSLTIKKPNELSRVLGLKTLATHGLAAVNSVPWDTIANYAKPFLNKVVSTTTN
KAYNVTQAFGRRGPEQTQGNFGDQELIRQGTDYKHWPQIAQFAPSASAFFGMSRIGMEVTPSGTWLTYTGA

VITFDNLKTLLSLREVRTIKVFTTVDNINLHTQVVDMSMTYGQQFGPTYLDGADVTKIKPHNSHEGKTFYVLPN[illegible]

NIVTRCLNRVCTNYMPYFFTLLLQLCTFTRSTNSRIKASMPTTIAKNTVKSVGKFCLEASFNYLKSPNFSKLINIIIW[illegible]
AIKLDDKDPNFKDQVILLNKHIDAYKTFPPTEPKKDKKKKADETQALPQRQKKQQTVTLLPAADLDDFSKQLQQS[illegible]

[illegible]DTLRVEAFEYYHTTDPSFLGRYMSALNHTKKWKYPQVNGLTSIKWADNNCYLATALLTLQQIELKFNPPALQ[illegible]

FLLLSVCLGSLIYSTAALGVLMSNLGMPSYCTGYREGYLNSTNVTIATYCTGSIPCSVCLSGLDSLDTYPSLETIQIT[illegible]
[illegible]MSSADSTQA*

DAYYRARAGEAANFCALILAYCNKTVGELGDVRETMSYLFQHANLDSCKRVLNVVCKTCGQQQTTLKGVEAVM

ISSFKWDLTAFGLVAEWFLAYILFTRFFYVLGLAAIMQLFFSYFAVHFISNSWLMWLIINLVQMAPISAMVRMY

MYMGTLSYEQFKKGVQIPCTCGKQATKYLVQQESPFVMMSAPPAQYELKHGTFTCASEYTGNYQCGHYKHIT

IFFASFYYVWKSYVHVVDGCNSSTCMMCYKRNRATRVECTTIVNGVRRSFYVYANGGKGFCKLHNWNCVNC

SKETLYCIDGALLTKSSEYKGPITDVFYKENSYTTTIKPVTYKLDGVVCTEIDPKLDNYYKKDNSYFTEQPIDLVPN[illegible]

DTFCAGSTFISDEVARDLSLQFKRPINPTDQSSYIVDSVTVKNGSIHLYFDKAGQKTYERHSLSHFVNLDNLRAN[illegible]

QPYPNASFDNFKFVCDNIKFADDLNQLTGYKKPASRELKVTFFPDLNGDVVAIDYKHYTPSFKKGAKLLHKPIVW

NTKGSLPINVIVFDGKSKCEESSAKSASVYYSQLMCQPILLLDQALVSDVGDSAEVAVKMFDAYVNTFSSTFNV[illegible]

HVNNATNKATYKPNTWCIRCLWSTKPVETSNSFDVLKSEDAQGMDNLACEDLKPVSEEVVENPTIQKDVLE

MEKLKTLVATAEAELAKNVSLDNVLSTFISAARQGFVDSDVETKDVVECLKLSHQSDIEVTGDSCNNYMLTYN

CNVKTTEVVGDIILKPANNSLKITEEVGHTDLMAAYVDNSSLTIKKPNELSRVLGLKTLATHGLAAVNSVPWDTIA

KVENMTPRDLGACIDCSARHINAQVAKSHNIALIWNVKDFMSLSEQLRKQIRSAAKKNNLPFKLTCATTRQVV

ANYAKPFLNKVVSTTTNIVTRCLNRVCTNYMPYFFTLLLQLCTFTRSTNSRIKASMPTTIAKNTVKSVGKFCLEAS

NVVTTKIALKGGKIVNNWLKQLIKVTLVFLFVAAIFYLITPVHVMSKHTDFSSEIIGYKAIDGGVTRDIASTDTCFA

FN

ꓽN

	subgenome	ref_leader_end	peak_leader_end	ref_TRS_start
S1-Day1	S	65	65	21552
	ORF3a	69	69	25385
	E	69	69	26237
	M	65	65	26469
	ORF6	69	69	27041
	ORF7a	69	69	27388
	ORF7b	65	0	27644
	ORF8	65	65	27884
	N	65	64	28256
	ORF10	65	0	29530
S2-Day2	S	65	65	21552
	ORF3a	69	69	25385
	E	69	69	26237
	M	65	65	26469
	ORF6	69	69	27041
	ORF7a	69	69	27388
	ORF7b	65	0	27644
	ORF8	65	65	27884
	N	65	64	28256
	ORF10	65	0	29530
S3-Day3	S	65	65	21552
	ORF3a	69	69	25385
	E	69	69	26237
	M	65	65	26469
	ORF6	69	69	27041
	ORF7a	69	69	27388
	ORF7b	65	0	27644
	ORF8	65	0	27884
	N	65	64	28256
	ORF10	65	0	29530
S1-Day4	S	65	65	21552
	ORF3a	69	69	25385
	E	69	0	26237
	M	65	64	26469
	ORF6	69	69	27041
	ORF7a	69	69	27388
	ORF7b	65	0	27644
	ORF8	65	65	27884
	N	65	64	28256
	ORF10	65	0	29530
	S	65	65	21552
	ORF3a	69	69	25385

	E	69	69	26237
	M	65	64	26469
S2-Day5	ORF6	69	0	27041
	ORF7a	69	69	27388
	ORF7b	65	65	27644
	ORF8	65	65	27884
	N	65	64	28256
	ORF10	65	0	29530
	S	65	0	21552
	ORF3a	69	0	25385
	E	69	69	26237
	M	65	0	26469
S3-Day6	ORF6	69	69	27041
	ORF7a	69	69	27388
	ORF7b	65	0	27644
	ORF8	65	0	27884
	N	65	65	28256
	ORF10	65	0	29530
	S	65	0	21552
	ORF3a	69	0	25385
	E	69	0	26237
	M	65	0	26469
S2-Day8	ORF6	69	69	27041
	ORF7a	69	0	27388
	ORF7b	65	0	27644
	ORF8	65	65	27884
	N	65	65	28256
	ORF10	65	65	29530
	S	65	0	21552
	ORF3a	69	0	25385
	E	69	0	26237
	M	65	65	26469
S3-Day9	ORF6	69	0	27041
	ORF7a	69	0	27388
	ORF7b	65	0	27644
	ORF8	65	0	27884
	N	65	65	28256
	ORF10	65	0	29530
	S	65	0	21552
	ORF3a	69	69	25385
	E	69	69	26237
	M	65	65	26469
S2-Day11	ORF6	69	69	27041

S2-Day11	ORF7a	69	0	27388
	ORF7b	65	0	27644
	ORF8	65	0	27884
	N	65	65	28256
	ORF10	65	0	29530
S3-Day12	S	65	65	21552
	ORF3a	69	69	25385
	E	69	0	26237
	M	65	0	26469
	ORF6	69	69	27041
	ORF7a	69	0	27388
	ORF7b	65	0	27644
	ORF8	65	0	27884
	N	65	64	28256
	ORF10	65	0	29530
S3-Day14	S	65	0	21552
	ORF3a	69	0	25385
	E	69	0	26237
	M	65	65	26469
	ORF6	69	0	27041
	ORF7a	69	0	27388
	ORF7b	65	0	27644
	ORF8	65	0	27884
	N	65	65	28256
	ORF10	65	0	29530

The numbers in the bracket are (reads with left primers, reads with right primers, reads with both
Normalized count=(Read count/Total number of read mapped on reference genome)*1000000.
Total number of read mapped on reference genome is 36593897, 37733596, 33222295, 3394950
“ref_leader_end” and “peak_leader_end” point to the reference position of the end of leader an

peak_TRS_start	peak_count	peak_normalized_cour	cluster_count
21552	1697(1696,1320,1319,1	46.37(46.35,36.07,36.0	1706(1705,1327,1326
25385	1339(1279,0,0,18)	36.59(34.95,0.00,0.00,(	1343(1283,0,0,18)
26237	512(506,0,0,6)	13.99(13.83,0.00,0.00,(	515(508,0,0,6)
26469	3076(3073,2952,2949,2	84.06(83.98,80.67,80.5	3181(3178,3041,3038
27041	3670(3662,3298,3291,3	100.29(100.07,90.12,8	3704(3696,3332,3325
27388	42(42,2,2,8)	1.15(1.15,0.05,0.05,0.2	42(42,2,2,8)
0	0	0	0
27884	405(405,0,0,4)	11.07(11.07,0.00,0.00,(	406(406,0,0,4)
28255	4861(4853,4796,4788,4	132.84(132.62,131.06,1	6447(6435,6362,6350
0	0	0	0
21552	4522(4515,3287,3282,3	119.84(119.65,87.11,8	4552(4545,3297,3292
25385	2661(2598,0,0,24)	70.52(68.85,0.00,0.00,(	2745(2681,0,0,24)
26237	1630(1612,3,3,18)	43.20(42.72,0.08,0.08,(	1632(1614,3,3,18)
26469	679(679,637,637,648)	17.99(17.99,16.88,16.8	931(929,873,872,864)
27041	6163(6144,5492,5473,5	163.33(162.83,145.55,1	6229(6209,5550,5531
27388	56(53,4,1,0)	1.48(1.40,0.11,0.03,0.0	56(53,4,1,0)
0	0	0	0
27884	200(200,0,0,0)	5.30(5.30,0.00,0.00,0.0	200(200,0,0,0)
28255	36388(36328,35987,359	964.34(962.75,953.71,9	37914(37830,37462,3
0	0	0	0
21552	3906(3899,3358,3351,3	117.57(117.36,101.08,1	3930(3923,3377,3370
25385	212(212,0,0,4)	6.38(6.38,0.00,0.00,0.1	212(212,0,0,4)
26237	5208(5203,2,2,36)	156.76(156.61,0.06,0.0	5208(5203,2,2,36)
26469	7037(7027,6807,6798,6	211.82(211.51,204.89,2	7085(7074,6852,6842
27041	11089(11078,10731,107	333.78(333.45,323.01,3	11119(11108,10761,1
27388	2(2,0,0,0)	0.06(0.06,0.00,0.00,0.0	2(2,0,0,0)
0	0	0	0
0	0	0	0
28255	27632(27607,27286,272	831.73(830.98,821.32,8	28304(28266,27942,2
0	0	0	0
21552	0(0,0,0,0)	0.00(0.00,0.00,0.00,0.0	0(0,0,0,0)
25385	298(296,0,0,6)	8.78(8.72,0.00,0.00,0.1	298(296,0,0,6)
0	0	0	0
26468	3815(3815,3714,3714,3	112.37(112.37,109.40,1	6375(6371,6193,6189
27041	11235(11220,10744,107	330.93(330.49,316.47,3	11285(11270,10792,1
27388	8(8,0,0,0)	0.24(0.24,0.00,0.00,0.0	8(8,0,0,0)
0	0	0	0
27884	116(116,0,0,4)	3.42(3.42,0.00,0.00,0.1	116(116,0,0,4)
28255	32243(32207,31567,315	949.73(948.67,929.82,9	37100(37053,36346,3
0	0	0	0
21552	2(2,2,2,2)	0.07(0.07,0.07,0.07,0.0	2(2,2,2,2)
25385	1086(1077,0,0,20)	38.48(38.16,0.00,0.00,(	1089(1080,0,0,20)

26237	862(860,0,0,2)	30.55(30.47,0.00,0.00,(	862(860,0,0,2)
26468	5952(5952,5840,5840,5	210.91(210.91,206.95,:	8897(8897,8702,8702
0	0	0	0
27388	5(4,1,0,2)	0.18(0.14,0.04,0.00,0.0	5(4,1,0,2)
27644	1(1,0,0,0)	0.04(0.04,0.00,0.00,0.0	1(1,0,0,0)
27884	1(1,0,0,0)	0.04(0.04,0.00,0.00,0.0	1(1,0,0,0)
28255	51775(51734,51281,512	1834.69(1833.24,1817.	55251(55188,54723,5
0	0	0	0
0	0	0	0
0	0	0	0
26237	1(0,0,0,0)	0.03(0.00,0.00,0.00,0.0	1(0,0,0,0)
0	0	0	0
27041	2(2,2,2,2)	0.07(0.07,0.07,0.07,0.0	2(2,2,2,2)
27388	4(4,0,0,2)	0.14(0.14,0.00,0.00,0.0	4(4,0,0,2)
0	0	0	0
0	0	0	0
28256	9(4,9,4,4)	0.31(0.14,0.31,0.14,0.1	9(4,9,4,4)
0	0	0	0
0	0	0	0
0	0	0	0
0	0	0	0
0	0	0	0
27041	2(2,2,2,2)	0.12(0.12,0.12,0.12,0.1	2(2,2,2,2)
0	0	0	0
0	0	0	0
27884	117(111,0,0,0)	7.29(6.92,0.00,0.00,0.0	117(111,0,0,0)
28256	2(0,2,0,0)	0.12(0.00,0.12,0.00,0.0	2(0,2,0,0)
29530	1(0,0,0,0)	0.06(0.00,0.00,0.00,0.0	1(0,0,0,0)
0	0	0	0
0	0	0	0
0	0	0	0
26469	1789(1787,1780,1778,1	138.15(137.99,137.45,:	1798(1796,1789,1787
0	0	0	0
0	0	0	0
0	0	0	0
0	0	0	0
28256	6(2,6,2,2)	0.46(0.15,0.46,0.15,0.1	6(2,6,2,2)
0	0	0	0
0	0	0	0
25385	2(0,0,0,0)	0.12(0.00,0.00,0.00,0.0	2(0,0,0,0)
26237	1(1,0,0,0)	0.06(0.06,0.00,0.00,0.0	1(1,0,0,0)
26469	4(4,4,4,4)	0.23(0.23,0.23,0.23,0.2	4(4,4,4,4)
27041	2(2,2,2,0)	0.12(0.12,0.12,0.12,0.0	2(2,2,2,0)

0	0	0	0	
0	0	0	0	
0	0	0	0	
28256	16(16,16,16,16)	0.92(0.92,0.92,0.92,0.9	16(16,16,16,16)	
0	0	0	0	
21552	21437(21418,21323,213	1258.82(1257.70,1252.	21544(21524,21420,2	
25385	1(0,0,0,0)	0.06(0.00,0.00,0.00,0.0	1(0,0,0,0)	
0	0	0	0	
0	0	0	0	
27041	78468(78373,77971,778	4607.78(4602.20,4578.	78719(78623,78221,7	
0	0	0	0	
0	0	0	0	
0	0	0	0	
28255	299498(299228,298519,	17587.04(17571.19,175	305621(305289,3045(	
0	0	0	0	
0	0	0	0	
0	0	0	0	
0	0	0	0	
26469	4(4,4,4,2)	0.39(0.39,0.39,0.39,0.2	4(4,4,4,2)	
0	0	0	0	
0	0	0	0	
0	0	0	0	
0	0	0	0	
28256	2(2,2,2,2)	0.20(0.20,0.20,0.20,0.2	2(2,2,2,2)	
0	0	0	0	

primers, same junction on paired reads with at least a primer).

3, 28220017, 28624584, 16048939, 12949851, 17312079, 17029467, 20512435 and 1014[illegible]
d the position of the end of leader identified in the most common reads (peak count) on th

cluster_normalized_count
46.62(46.59,36.26,36.24,37.82)
36.70(35.06,0.00,0.00,0.49)
14.07(13.88,0.00,0.00,0.16)
86.93(86.85,83.10,83.02,83.68)
101.22(101.00,91.05,90.86,94.22)
1.15(1.15,0.05,0.05,0.22)
0
11.09(11.09,0.00,0.00,0.11)
176.18(175.85,173.85,173.53,140.24)
0
120.64(120.45,87.38,87.24,94.82)
72.75(71.05,0.00,0.00,0.64)
43.25(42.77,0.08,0.08,0.48)
24.67(24.62,23.14,23.11,22.90)
165.08(164.55,147.08,146.58,150.26)
1.48(1.40,0.11,0.03,0.00)
0
5.30(5.30,0.00,0.00,0.00)
1004.78(1002.55,992.80,990.74,986.81)
0
118.29(118.08,101.65,101.44,104.33)
6.38(6.38,0.00,0.00,0.12)
156.76(156.61,0.06,0.06,1.08)
213.26(212.93,206.25,205.95,207.99)
334.68(334.35,323.91,323.58,322.43)
0.06(0.06,0.00,0.00,0.00)
0
0
851.96(850.81,841.06,839.95,840.52)
0
0.00(0.00,0.00,0.00,0.00)
8.78(8.72,0.00,0.00,0.18)
0
187.78(187.66,182.42,182.30,185.75)
332.41(331.96,317.88,317.44,321.18)
0.24(0.24,0.00,0.00,0.00)
0
3.42(3.42,0.00,0.00,0.12)
1092.80(1091.42,1070.59,1069.21,1022.17)
0
0.07(0.07,0.07,0.07,0.07)
38.59(38.27,0.00,0.00,0.71)

30.55(30.47,0.00,0.00,0.07)
315.27(315.27,308.36,308.36,312.26)
0
0.18(0.14,0.04,0.00,0.07)
0.04(0.04,0.00,0.00,0.00)
0.04(0.04,0.00,0.00,0.00)
1957.87(1955.63,1939.16,1937.03,1932.60)
0
0
0
0.03(0.00,0.00,0.00,0.00)
0
0.07(0.07,0.07,0.07,0.07)
0.14(0.14,0.00,0.00,0.07)
0
0
0.31(0.14,0.31,0.14,0.14)
0
0
0
0
0
0.12(0.12,0.12,0.12,0.12)
0
0
7.29(6.92,0.00,0.00,0.00)
0.12(0.00,0.12,0.00,0.00)
0.06(0.00,0.00,0.00,0.00)
0
0
0
138.84(138.69,138.15,137.99,136.06)
0
0
0
0
0.46(0.15,0.46,0.15,0.15)
0
0
0.12(0.00,0.00,0.00,0.00)
0.06(0.06,0.00,0.00,0.00)
0.23(0.23,0.23,0.23,0.23)
0.12(0.12,0.12,0.12,0.00)

0
0
0
0.92(0.92,0.92,0.92,0.92)
0
1265.10(1263.93,1257.82,1256.65,1219.18)
0.06(0.00,0.00,0.00,0.00)
0
0
4622.52(4616.88,4593.27,4587.69,4547.18)
0
0
0
17946.60(17927.10,17884.71,17865.97,17724.57)
0
0
0
0
0.39(0.39,0.39,0.39,0.20)
0
0
0
0
0.20(0.20,0.20,0.20,0.20)
0

3083, excluding the mapped reads unpaired, not primary alignment and supplementary alignn
ne reference genome, and “ref_TRS_start” and “peak_TRS_start” refer to the reference positi

nent.
on of the start of TRS and the position of the start of TRS identified in the most common reads (

peak count) on the reference genome.

	subgenome	ref_leader_e	peak_leader_	ref_TRS_start	peak_TRS_sta
S1-Day1	S	65	0	21552	0
	ORF3a	69	69	25385	25385
	E	69	0	26237	0
	M	65	65	26469	26469
	ORF6	69	0	27041	0
	ORF7a	69	0	27388	0
	ORF7b	65	0	27644	0
	ORF8	65	65	27884	27884
	N	65	65	28256	28256
	ORF10	65	0	29530	0
S2-Day2	S	65	65	21552	21552
	ORF3a	69	69	25385	25385
	E	69	69	26237	26237
	M	65	65	26469	26469
	ORF6	69	69	27041	27041
	ORF7a	69	69	27388	27388
	ORF7b	65	65	27644	27644
	ORF8	65	65	27884	27884
	N	65	64	28256	28255
	ORF10	65	0	29530	0
S3-Day3	S	65	0	21552	0
	ORF3a	69	69	25385	25385
	E	69	0	26237	0
	M	65	65	26469	26469
	ORF6	69	0	27041	0
	ORF7a	69	69	27388	27388
	ORF7b	65	0	27644	0
	ORF8	65	65	27884	27884
	N	65	64	28256	28255
	ORF10	65	0	29530	0
S1-Day4	S	65	0	21552	0
	ORF3a	69	69	25385	25385
	E	69	69	26237	26237
	M	65	65	26469	26469
	ORF6	69	0	27041	0
	ORF7a	69	69	27388	27388
	ORF7b	65	0	27644	0
	ORF8	65	0	27884	0
	N	65	64	28256	28255
	ORF10	65	0	29530	0
	S	65	65	21552	21552
	ORF3a	69	69	25385	25385

	E	69	69	26237	26237
	M	65	64	26469	26468
S2-Day5	ORF6	69	69	27041	27041
	ORF7a	69	69	27388	27388
	ORF7b	65	65	27644	27644
	ORF8	65	65	27884	27884
	N	65	64	28256	28255
	ORF10	65	0	29530	0
	S	65	0	21552	0
	ORF3a	69	69	25385	25385
	E	69	69	26237	26237
	M	65	65	26469	26469
S3-Day6	ORF6	69	69	27041	27041
	ORF7a	69	69	27388	27388
	ORF7b	65	0	27644	0
	ORF8	65	0	27884	0
	N	65	64	28256	28255
	ORF10	65	0	29530	0
	S	65	65	21552	21552
	ORF3a	69	69	25385	25385
	E	69	69	26237	26237
	M	65	64	26469	26468
S2-Day8	ORF6	69	69	27041	27041
	ORF7a	69	69	27388	27388
	ORF7b	65	0	27644	0
	ORF8	65	65	27884	27884
	N	65	64	28256	28255
	ORF10	65	0	29530	0
	S	65	0	21552	0
	ORF3a	69	69	25385	25385
	E	69	0	26237	0
	M	65	0	26469	0
S3-Day9	ORF6	69	0	27041	0
	ORF7a	69	0	27388	0
	ORF7b	65	0	27644	0
	ORF8	65	0	27884	0
	N	65	65	28256	28256
	ORF10	65	0	29530	0
	S	65	0	21552	0
	ORF3a	69	0	25385	0
	E	69	0	26237	0
	M	65	0	26469	0
S2-Day11	ORF6	69	0	27041	0

S2-Day11	ORF7a	69	0	27388	0
	ORF7b	65	0	27644	0
	ORF8	65	0	27884	0
	N	65	65	28256	28256
	ORF10	65	0	29530	0
S3-Day12	S	65	0	21552	0
	ORF3a	69	0	25385	0
	E	69	0	26237	0
	M	65	0	26469	0
	ORF6	69	0	27041	0
	ORF7a	69	0	27388	0
	ORF7b	65	0	27644	0
	ORF8	65	0	27884	0
	N	65	65	28256	28256
	ORF10	65	0	29530	0
S3-Day18	S	65	0	21552	0
	ORF3a	69	0	25385	0
	E	69	0	26237	0
	M	65	0	26469	0
	ORF6	69	69	27041	27041
	ORF7a	69	0	27388	0
	ORF7b	65	0	27644	0
	ORF8	65	0	27884	0
	N	65	65	28256	28256
	ORF10	65	0	29530	0

The numbers in the bracket are (reads with left primers, reads with right primers, reads wi
Normalized count=(Read count/Total number of read mapped on reference genome)*100(
Total number of read mapped on reference genome is17428684, 35949645, 26863662, 28
“ref_leader_end” and “peak_leader_end” point to the reference position of the end of lea

peak_count	peak_normal	cluster_count	cluster_normalized_count
0	0	0	0
56(56,0,0,2)	3.21(3.21,0.0	56(56,0,0,2)	3.21(3.21,0.00,0.00,0.11)
0	0	0	0
1027(1026,10	58.93(58.87,5	1078(1077,10	61.85(61.79,60.88,60.82,59.90)
0	0	0	0
0	0	0	0
0	0	0	0
86(85,0,0,0)	4.93(4.88,0.0	86(85,0,0,0)	4.93(4.88,0.00,0.00,0.00)
19(14,19,14,1	1.09(0.80,1.0	19(14,19,14,1	1.09(0.80,1.09,0.80,0.80)
0	0	0	0
8162(8096,66	227.04(225.2	8232(8166,67	228.99(227.15,187.18,186.29,197.72)
3285(3192,0,	91.38(88.79,0	3322(3216,0,	92.41(89.46,0.00,0.00,2.67)
4011(3981,3,	111.57(110.7	4011(3981,3,	111.57(110.74,0.08,0.08,1.39)
3865(3864,37	107.51(107.4	3999(3992,38	111.24(111.04,106.45,106.32,107.59)
5071(5054,47	141.06(140.5	5116(5099,47	142.31(141.84,132.94,132.55,133.30)
42(35,18,12,1	1.17(0.97,0.5	43(36,18,12,1	1.20(1.00,0.50,0.33,0.39)
2(2,0,0,0)	0.06(0.06,0.0	2(2,0,0,0)	0.06(0.06,0.00,0.00,0.00)
105(105,0,0,0	2.92(2.92,0.0	106(105,0,0,0	2.95(2.92,0.00,0.00,0.00)
15018(14971	417.75(416.4	23046(22968	641.06(638.89,630.24,628.13,484.34)
0	0	0	0
0	0	0	0
1449(1443,0,	53.94(53.72,0	1459(1452,0,	54.31(54.05,0.00,0.00,0.22)
0	0	0	0
3151(3147,31	117.30(117.1	3151(3147,31	117.30(117.15,115.92,115.81,115.17)
0	0	0	0
26(26,4,4,18)	0.97(0.97,0.1	26(26,4,4,18)	0.97(0.97,0.15,0.15,0.67)
0	0	0	0
3(3,0,0,0)	0.11(0.11,0.0	3(3,0,0,0)	0.11(0.11,0.00,0.00,0.00)
8355(8346,82	311.01(310.6	12284(12268	457.27(456.68,453.55,452.99,444.99)
0	0	0	0
0	0	0	0
1273(1266,0,	44.32(44.07,0	1275(1268,0,	44.39(44.14,0.00,0.00,0.84)
2274(2273,0,	79.16(79.13,0	2274(2273,0,	79.16(79.13,0.00,0.00,0.70)
2032(2030,20	70.74(70.67,6	2123(2121,20	73.91(73.84,73.07,73.00,71.71)
0	0	0	0
11(11,0,0,6)	0.38(0.38,0.0	11(11,0,0,6)	0.38(0.38,0.00,0.00,0.21)
0	0	0	0
0	0	0	0
3165(3161,31	110.18(110.0	3375(3370,33	117.49(117.32,115.58,115.40,115.71)
0	0	0	0
6105(6088,51	166.30(165.8	6142(6125,51	167.31(166.84,139.90,139.50,146.22)
1744(1696,0,	47.51(46.20,0	1748(1700,0,	47.62(46.31,0.00,0.00,0.93)

8721(8670,1	237.56(236.1	8724(8673,1	237.64(236.25,0.33,0.33,1.31)
46115(46098	1256.17(125	48146(48125	1311.49(1310.92,1273.96,1273.49,1300.65)
11818(11791	321.92(321.1	11881(11854	323.64(322.90,305.66,305.01,304.54)
26(26,0,0,8)	0.71(0.71,0.0	26(26,0,0,8)	0.71(0.71,0.00,0.00,0.22)
2(2,0,0,0)	0.05(0.05,0.0	2(2,0,0,0)	0.05(0.05,0.00,0.00,0.00)
182(182,0,0,	4.96(4.96,0.0	182(182,0,0,	4.96(4.96,0.00,0.00,0.16)
69092(69015	1882.06(187	72672(72565	1979.58(1976.66,1953.37,1950.60,1949.29)
0	0	0	0
0	0	0	0
699(694,0,0,	27.60(27.40,	699(694,0,0,	27.60(27.40,0.00,0.00,0.39)
3(0,0,0,0)	0.12(0.00,0.0	3(0,0,0,0)	0.12(0.00,0.00,0.00,0.00)
224(224,217,	8.85(8.85,8.5	247(247,238,	9.75(9.75,9.40,9.40,9.32)
5721(5707,5	225.90(225.3	5755(5741,5	227.25(226.69,218.36,217.85,219.07)
13(13,0,0,2)	0.51(0.51,0.0	13(13,0,0,2)	0.51(0.51,0.00,0.00,0.08)
0	0	0	0
0	0	0	0
24203(24191	955.70(955.2	25172(25151	993.96(993.13,978.40,977.61,980.77)
0	0	0	0
4842(4837,4	160.54(160.3	4874(4869,4	161.60(161.43,135.61,135.51,136.40)
2078(2045,0,	68.90(67.80,	2099(2063,0,	69.59(68.40,0.00,0.00,2.32)
1978(1976,0,	65.58(65.51,	1978(1976,0,	65.58(65.51,0.00,0.00,0.13)
16006(15989	530.68(530.1	17328(17307	574.51(573.82,558.40,557.74,569.21)
4757(4726,4	157.72(156.6	4784(4753,4	158.61(157.59,149.70,148.70,149.86)
551(49,506,7	18.27(1.62,1	559(54,514,1	18.53(1.79,17.04,0.40,0.80)
0	0	0	0
239(238,0,0,	7.92(7.89,0.0	245(244,0,0,	8.12(8.09,0.00,0.00,0.00)
64064(63940	2124.06(211	65600(65458	2174.99(2170.28,2142.66,2138.12,2147.86)
0	0	0	0
0	0	0	0
2(0,0,0,0)	0.09(0.00,0.0	2(0,0,0,0)	0.09(0.00,0.00,0.00,0.00)
0	0	0	0
0	0	0	0
0	0	0	0
0	0	0	0
0	0	0	0
0	0	0	0
7(2,7,2,2)	0.32(0.09,0.3	7(2,7,2,2)	0.32(0.09,0.32,0.09,0.09)
0	0	0	0
0	0	0	0
0	0	0	0
0	0	0	0
0	0	0	0
0	0	0	0

0	0	0	0
0	0	0	0
0	0	0	0
2(2,2,2,2)	0.13(0.13,0.1	2(2,2,2,2)	0.13(0.13,0.13,0.13,0.13)
0	0	0	0
0	0	0	0
0	0	0	0
0	0	0	0
0	0	0	0
0	0	0	0
0	0	0	0
0	0	0	0
0	0	0	0
18(2,18,2,2)	1.41(0.16,1.4	18(2,18,2,2)	1.41(0.16,1.41,0.16,0.16)
0	0	0	0
0	0	0	0
0	0	0	0
0	0	0	0
0	0	0	0
2(2,2,2,2)	0.13(0.13,0.1	2(2,2,2,2)	0.13(0.13,0.13,0.13,0.13)
0	0	0	0
0	0	0	0
0	0	0	0
2(2,2,2,2)	0.13(0.13,0.1	2(2,2,2,2)	0.13(0.13,0.13,0.13,0.13)
0	0	0	0

th both primers, same junction on paired reads with at least a primer).

)000.

725840, 36710848, 25324959, 30161120, 22185877, 15869342, 12758257, 18881995 and 1509[illegible]

der and the position of the end of leader identified in the most common reads (peak count) on t[illegible]

3231, excluding the mapped reads unpaired, not primary alignment and supplementary alignmen
he reference genome, and “ref_TRS_start” and “peak_TRS_start” refer to the reference position

ıt.
of the start of TRS and the position of the start of TRS identified in the most common reads (pea

k count) on the reference genome.

	subgenome	peak_leader_	peak_TRS_sta	ACGAAC	ATG_postion
S1-Day1	S	65	21552	yes	21563
	ORF3a	69	25385	yes	25393
	E	69	26237	yes	26245
	M	65	26469	yes	26523
	ORF6	69	27041	yes	27202
	ORF7a	69	27388	yes	27394
	ORF8	65	27884	yes	27894
	N	64	28255	yes	28274
S2-Day2	S	65	21552	yes	21563
	ORF3a	69	25385	yes	25393
	E	69	26237	yes	26245
	M	65	26469	yes	26523
	ORF6	69	27041	yes	27202
	ORF7a	69	27388	yes	27394
	ORF8	65	27884	yes	27894
	N	64	28255	yes	28274
S3-Day3	S	65	21552	yes	21563
	ORF3a	69	25385	yes	25393
	E	69	26237	yes	26245
	M	65	26469	yes	26523
	ORF6	69	27041	yes	27202
	ORF7a	69	27388	yes	27394
	N	64	28255	yes	28274
S1-Day4	S	65	21552	yes	21563
	ORF3a	69	25385	yes	25393
	M	64	26468	yes	26523
	ORF6	69	27041	yes	27202
	ORF7a	69	27388	yes	27394
	ORF8	65	27884	yes	27894
	N	64	28255	yes	28274
S2-Day5	S	65	21552	yes	21563
	ORF3a	69	25385	yes	25393
	E	69	26237	yes	26245
	M	64	26468	yes	26523
	ORF7a	69	27388	yes	27394
	ORF7b	65	27644	no	27756
	ORF8	65	27884	yes	27894
	N	64	28255	yes	28274
S3-Day6	E	69	26237	yes	26245
	ORF6	69	27041	yes	27202
	ORF7a	69	27388	yes	27394
	N	65	28256	yes	28274

	ORF6	69	27041	yes	27202
S2-Day8	ORF8	65	27884	yes	27894
	N	65	28256	yes	28274
	ORF10	65	29530	no	29538
S3-Day9	M	65	26469	yes	26523
	N	65	28256	yes	28274
S2-Day11	ORF3a	69	25385	yes	25393
	E	69	26237	yes	26245
	M	65	26469	yes	26523
	ORF6	69	27041	yes	27202
	N	65	28256	yes	28274
S3-Day12	S	65	21552	yes	21563
	ORF3a	69	25385	yes	25393
	ORF6	69	27041	yes	27202
	N	64	28255	yes	28274
S3-Day14	M	65	26469	yes	26523
	N	65	28256	yes	28274

“peak_leader” and “peak_TRS_start” point to the leader-TRS junctions in sheet "kr

The numbers in the bracket are (reads with left primers, reads with right primers, r
Normalized count=(Read count/Total number of read mapped on reference genom
Total number of read mapped on reference genome is 36593897, 37733596, 3322

20_leader_seq	TRS_seq	first_orf_aa
ATCTCTTGTAGATCTGTTCT	CTAAACGAACA	MFVFLVLLPLVSSQCVNLTTRTQLPPAYTNSF
CTTGTAGATCTGTTCTCTAA	ACGAACTT	MDLFMRIFTIGTVTLKQGEIKDATPSDFVRAT
CTTGTAGATCTGTTCTCTAA	ACGAACTT	MYSFVSEETGTLIVNSVLLFLAFVVFLLVTLAIL
ATCTCTTGTAGATCTGTTCT	CTAAACGAACTAAAT	MADSNGTITVEELKKLLEQWNLVIGFLFLTW
CTTGTAGATCTGTTCTCTAA	ACGAACGCTTTCTTA	MFHLVDFQVTIAEILLIIMRTFKVSIWNLDYIII
CTTGTAGATCTGTTCTCTAA	ACGAAC	MKIILFLALITLATCELYHYQECVRGTTVLLKEF
ATCTCTTGTAGATCTGTTCT	CTAAACGAAC	MKFLVFLGIITTVAAFHQECSLQSCTQHQPY\
GATCTCTTGTAGATCTGTTC	TCTAAACGAACAAAC	MSDNGPQNQRNAPRITFGGPSDSTGSNQN
ATCTCTTGTAGATCTGTTCT	CTAAACGAACA	MFVFLVLLPLVSSQCVNLTTRTQLPPAYTNSF
CTTGTAGATCTGTTCTCTAA	ACGAACTT	MDLFMRIFTIGTVTLKQGEIKDATPSDFVRAT
CTTGTAGATCTGTTCTCTAA	ACGAACTT	MYSFVSEETGTLIVNSVLLFLAFVVFLLVTLAIL
ATCTCTTGTAGATCTGTTCT	CTAAACGAACTAAAT	MADSNGTITVEELKKLLEQWNLVIGFLFLTW
CTTGTAGATCTGTTCTCTAA	ACGAACGCTTTCTTA	MFHLVDFQVTIAEILLIIMRTFKVSIWNLDYIII
CTTGTAGATCTGTTCTCTAA	ACGAAC	MKIILFLALITLATCELYHYQECVRGTTVLLKEF
ATCTCTTGTAGATCTGTTCT	CTAAACGAAC	MKFLVFLGIITTVAAFHQECSLQSCTQHQPY\
GATCTCTTGTAGATCTGTTC	TCTAAACGAACAAAC	MSDNGPQNQRNAPRITFGGPSDSTGSNQN
ATCTCTTGTAGATCTGTTCT	CTAAACGAACA	MFVFLVLLPLVSSQCVNLTTRTQLPPAYTNSF
CTTGTAGATCTGTTCTCTAA	ACGAACTT	MDLFMRIFTIGTVTLKQGEIKDATPSDFVRAT
CTTGTAGATCTGTTCTCTAA	ACGAACTT	MYSFVSEETGTLIVNSVLLFLAFVVFLLVTLAIL
ATCTCTTGTAGATCTGTTCT	CTAAACGAACTAAAT	MADSNGTITVEELKKLLEQWNLVIGFLFLTW
CTTGTAGATCTGTTCTCTAA	ACGAACGCTTTCTTA	MFHLVDFQVTIAEILLIIMRTFKVSIWNLDYIII
CTTGTAGATCTGTTCTCTAA	ACGAAC	MKIILFLALITLATCELYHYQECVRGTTVLLKEF
GATCTCTTGTAGATCTGTTC	TCTAAACGAACAAAC	MSDNGPQNQRNAPRITFGGPSDSTGSNQN
ATCTCTTGTAGATCTGTTCT	CTAAACGAACA	MFVFLVLLPLVSSQCVNLTTRTQLPPAYTNSF
CTTGTAGATCTGTTCTCTAA	ACGAACTT	MDLFMRIFTIGTVTLKQGEIKDATPSDFVRAT
GATCTCTTGTAGATCTGTTC	TCTAAACGAACTAAA	MADSNGTITVEELKKLLEQWNLVIGFLFLTW
CTTGTAGATCTGTTCTCTAA	ACGAACGCTTTCTTA	MFHLVDFQVTIAEILLIIMRTFKVSIWNLDYIII
CTTGTAGATCTGTTCTCTAA	ACGAAC	MKIILFLALITLATCELYHYQECVRGTTVLLKEF
ATCTCTTGTAGATCTGTTCT	CTAAACGAAC	MKFLVFLGIITTVAAFHQECSLQSCTQHQPY\
GATCTCTTGTAGATCTGTTC	TCTAAACGAACAAAC	MSDNGPQNQRNAPRITFGGPSDSTGSNQN
ATCTCTTGTAGATCTGTTCT	CTAAACGAACA	MFVFLVLLPLVSSQCVNLTTRTQLPPAYTNSF
CTTGTAGATCTGTTCTCTAA	ACGAACTT	MDLFMRIFTIGTVTLKQGEIKDATPSDFVRAT
CTTGTAGATCTGTTCTCTAA	ACGAACTT	MYSFVSEETGTLIVNSVLLFLAFVVFLLVTLAIL
GATCTCTTGTAGATCTGTTC	TCTAAACGAACTAAA	MADSNGTITVEELKKLLEQWNLVIGFLFLTW
CTTGTAGATCTGTTCTCTAA	ACGAAC	MKIILFLALITLATCELYHYQECVRGTTVLLKEF
ATCTCTTGTAGATCTGTTCT	CTAAACTGTTCATCA	MIELSLIDFYLCFLAFLLFLVLIMLIIFWFSLELQ
ATCTCTTGTAGATCTGTTCT	CTAAACGAAC	MKFLVFLGIITTVAAFHQECSLQSCTQHQPY\
GATCTCTTGTAGATCTGTTC	TCTAAACGAACAAAC	MSDNGPQNQRNAPRITFGGPSDSTGSNQN
CTTGTAGATCTGTTCTCTAA	ACGAACTT	MYSFVSEETGTLIVNSVLLFLAFVVFLLVTLAIL
CTTGTAGATCTGTTCTCTAA	ACGAACGCTTTCTTA	MFHLVDFQVTIAEILLIIMRTFKVSIWNLDYIII
CTTGTAGATCTGTTCTCTAA	ACGAAC	MKIILFLALITLATCELYHYQECVRGTTVLLKEF
ATCTCTTGTAGATCTGTTCT	CTAAACGAACAAACT	MSDNGPQNQRNAPRITFGGPSDSTGSNQN

CTTGTAGATCTGTTCTCTAA	ACGAACGCTTTCTTA	MFHLVDFQVTIAEILLIIMRTFKVSIWNLDYIII
ATCTCTTGTAGATCTGTTCT	CTAAACGAAC	MKFLVFLGIITTVAAFHQECSLQSCTQHQPYV
ATCTCTTGTAGATCTGTTCT	CTAAACGAACAAACT	MSDNGPQNQRNAPRITFGGPSDSTGSNQN
ATCTCTTGTAGATCTGTTCT	CTAAACTC	MQTTQGRWAI*
ATCTCTTGTAGATCTGTTCT	CTAAACGAACTAAAT	MADSNGTITVEELKKLLEQWNLVIGFLFLTW
ATCTCTTGTAGATCTGTTCT	CTAAACGAACAAACT	MSDNGPQNQRNAPRITFGGPSDSTGSNQN
CTTGTAGATCTGTTCTCTAA	ACGAACTT	MDLFMRIFTIGTVTLKQGEIKDATPSDFVRAT
CTTGTAGATCTGTTCTCTAA	ACGAACTT	MYSFVSEETGTLIVNSVLLFLAFVVFLLVTLAIL
ATCTCTTGTAGATCTGTTCT	CTAAACGAACTAAAT	MADSNGTITVEELKKLLEQWNLVIGFLFLTW
CTTGTAGATCTGTTCTCTAA	ACGAACGCTTTCTTA	MFHLVDFQVTIAEILLIIMRTFKVSIWNLDYIII
ATCTCTTGTAGATCTGTTCT	CTAAACGAACAAACT	MSDNGPQNQRNAPRITFGGPSDSTGSNQN
ATCTCTTGTAGATCTGTTCT	CTAAACGAACA	MFVFLVLLPLVSSQCVNLTTRTQLPPAYTNSF
CTTGTAGATCTGTTCTCTAA	ACGAACTT	MDLFMRIFTIGTVTLKQGEIKDATPSDFVRAT
CTTGTAGATCTGTTCTCTAA	ACGAACGCTTTCTTA	MFHLVDFQVTIAEILLIIMRTFKVSIWNLDYIII
GATCTCTTGTAGATCTGTTC	TCTAAACGAACAAAC	MSDNGPQNQRNAPRITFGGPSDSTGSNQN
ATCTCTTGTAGATCTGTTCT	CTAAACGAACTAAAT	MADSNGTITVEELKKLLEQWNLVIGFLFLTW
ATCTCTTGTAGATCTGTTCT	CTAAACGAACAAACT	MSDNGPQNQRNAPRITFGGPSDSTGSNQN

nown", “ACGAAC” indicates if there is a ACGAAC sequence in the “TRS_seq” (TRS sequences),

reads with both primers, same junction on paired reads with at least a primer).
ne)*1000000.
2295, 33949503, 28220017, 28624584, 16048939, 12949851, 17312079, 17029467, 2051243[illegible]

FTRGVYYPDKVFRSSVLHSTQDLFLPFFSNVTWFHAIHVSGTNGTKRFDNPVLPFNDGVYFASTEKSNIIRGWI
TATIPIQASLPFGWLIVGVALLAVFQSASKIITLKKRWQLALSKGVHFVCNLLLLFVTVYSHLLLVAAGLEAPFLYL
LTALRLCAYCCNIVNVSLVKPSFYVYSRVKNLNSSRVPDLLV*
TICLLQFAYANRNRFLYIIKLIFLWLLWPVTLACFVLAAVYRINWITGGIAIAMACLVGLMWLSYFIASFRLFARTF
NLIIKNLSKSLTENKYSQLDEEQPMEID*
PCSSGTYEGNSPFHPLADNKFALTCFSTQFAFACPDGVKHVYQLRARSVSPKLFIRQEEVQELYSPIFLIVAAIVF
VVDDPCPIHFYSKWYIRVGARKSAPLIELCVDEAGSKSPIQYIDIGNYTVSCLPFTINCQEPKLGSLVVRCSFYEDF
IGERSGARSKQRRPQGLPNNTASWFTALTQHGKEDLKFPRGQGVPINTNSSPDDQIGYYRRATRRIRGGDGK
FTRGVYYPDKVFRSSVLHSTQDLFLPFFSNVTWFHAIHVSGTNGTKRFDNPVLPFNDGVYFASTEKSNIIRGWI
TATIPIQASLPFGWLIVGVALLAVFQSASKIITLKKRWQLALSKGVHFVCNLLLLFVTVYSHLLLVAAGLEAPFLYL
LTALRLCAYCCNIVNVSLVKPSFYVYSRVKNLNSSRVPDLLV*
TICLLQFAYANRNRFLYIIKLIFLWLLWPVTLACFVLAAVYRINWITGGIAIAMACLVGLMWLSYFIASFRLFARTF
NLIIKNLSKSLTENKYSQLDEEQPMEID*
PCSSGTYEGNSPFHPLADNKFALTCFSTQFAFACPDGVKHVYQLRARSVSPKLFIRQEEVQELYSPIFLIVAAIVF
VVDDPCPIHFYSKWYIRVGARKSAPLIELCVDEAGSKSPIQYIDIGNYTVSCLPFTINCQEPKLGSLVVRCSFYEDF
IGERSGARSKQRRPQGLPNNTASWFTALTQHGKEDLKFPRGQGVPINTNSSPDDQIGYYRRATRRIRGGDGK
FTRGVYYPDKVFRSSVLHSTQDLFLPFFSNVTWFHAIHVSGTNGTKRFDNPVLPFNDGVYFASTEKSNIIRGWI
TATIPIQASLPFGWLIVGVALLAVFQSASKIITLKKRWQLALSKGVHFVCNLLLLFVTVYSHLLLVAAGLEAPFLYL
LTALRLCAYCCNIVNVSLVKPSFYVYSRVKNLNSSRVPDLLV*
TICLLQFAYANRNRFLYIIKLIFLWLLWPVTLACFVLAAVYRINWITGGIAIAMACLVGLMWLSYFIASFRLFARTF
NLIIKNLSKSLTENKYSQLDEEQPMEID*
PCSSGTYEGNSPFHPLADNKFALTCFSTQFAFACPDGVKHVYQLRARSVSPKLFIRQEEVQELYSPIFLIVAAIVF
IGERSGARSKQRRPQGLPNNTASWFTALTQHGKEDLKFPRGQGVPINTNSSPDDQIGYYRRATRRIRGGDGK
FTRGVYYPDKVFRSSVLHSTQDLFLPFFSNVTWFHAIHVSGTNGTKRFDNPVLPFNDGVYFASTEKSNIIRGWI
TATIPIQASLPFGWLIVGVALLAVFQSASKIITLKKRWQLALSKGVHFVCNLLLLFVTVYSHLLLVAAGLEAPFLYL
TICLLQFAYANRNRFLYIIKLIFLWLLWPVTLACFVLAAVYRINWITGGIAIAMACLVGLMWLSYFIASFRLFARTF
NLIIKNLSKSLTENKYSQLDEEQPMEID*
PCSSGTYEGNSPFHPLADNKFALTCFSTQFAFACPDGVKHVYQLRARSVSPKLFIRQEEVQELYSPIFLIVAAIVF
VVDDPCPIHFYSKWYIRVGARKSAPLIELCVDEAGSKSPIQYIDIGNYTVSCLPFTINCQEPKLGSLVVRCSFYEDF
IGERSGARSKQRRPQGLPNNTASWFTALTQHGKEDLKFPRGQGVPINTNSSPDDQIGYYRRATRRIRGGDGK
FTRGVYYPDKVFRSSVLHSTQDLFLPFFSNVTWFHAIHVSGTNGTKRFDNPVLPFNDGVYFASTEKSNIIRGWI
TATIPIQASLPFGWLIVGVALLAVFQSASKIITLKKRWQLALSKGVHFVCNLLLLFVTVYSHLLLVAAGLEAPFLYL
LTALRLCAYCCNIVNVSLVKPSFYVYSRVKNLNSSRVPDLLV*
TICLLQFAYANRNRFLYIIKLIFLWLLWPVTLACFVLAAVYRINWITGGIAIAMACLVGLMWLSYFIASFRLFARTF
PCSSGTYEGNSPFHPLADNKFALTCFSTQFAFACPDGVKHVYQLRARSVSPKLFIRQEEVQELYSPIFLIVAAIVF
QDHNETCHA*
VVDDPCPIHFYSKWYIRVGARKSAPLIELCVDEAGSKSPIQYIDIGNYTVSCLPFTINCQEPKLGSLVVRCSFYEDF
IGERSGARSKQRRPQGLPNNTASWFTALTQHGKEDLKFPRGQGVPINTNSSPDDQIGYYRRATRRIRGGDGK
LTALRLCAYCCNIVNVSLVKPSFYVYSRVKNLNSSRVPDLLV*
NLIIKNLSKSLTENKYSQLDEEQPMEID*
PCSSGTYEGNSPFHPLADNKFALTCFSTQFAFACPDGVKHVYQLRARSVSPKLFIRQEEVQELYSPIFLIVAAIVF
IGERSGARSKQRRPQGLPNNTASWFTALTQHGKEDLKFPRGQGVPINTNSSPDDQIGYYRRATRRIRGGDGK

NLIIKNLSKSLTENKYSQLDEEQPMEID*
VVDDPCPIHFYSKWYIRVGARKSAPLIELCVDEAGSKSPIQYIDIGNYTVSCLPFTINCQEPKLGSLVVRCSFYEDF
IGERSGARSKQRRPQGLPNNTASWFTALTQHGKEDLKFPRGQGVPINTNSSPDDQIGYYRRATRRIRGGDGK

TICLLQFAYANRNRFLYIIKLIFLWLLWPVTLACFVLAAVYRINWITGGIAIAMACLVGLMWLSYFIASFRLFARTF
IGERSGARSKQRRPQGLPNNTASWFTALTQHGKEDLKFPRGQGVPINTNSSPDDQIGYYRRATRRIRGGDGK
TATIPIQASLPFGWLIVGVALLAVFQSASKIITLKKRWQLALSKGVHFVCNLLLLFVTVYSHLLLVAAGLEAPFLYL
LTALRLCAYCCNIVNVSLVKPSFYVYSRVKNLNSSRVPDLLV*
TICLLQFAYANRNRFLYIIKLIFLWLLWPVTLACFVLAAVYRINWITGGIAIAMACLVGLMWLSYFIASFRLFARTF
NLIIKNLSKSLTENKYSQLDEEQPMEID*
IGERSGARSKQRRPQGLPNNTASWFTALTQHGKEDLKFPRGQGVPINTNSSPDDQIGYYRRATRRIRGGDGK
FTRGVYYPDKVFRSSVLHSTQDLFLPFFSNVTWFHAIHVSGTNGTKRFDNPVLPFNDGVYFASTEKSNIIRGWI
TATIPIQASLPFGWLIVGVALLAVFQSASKIITLKKRWQLALSKGVHFVCNLLLLFVTVYSHLLLVAAGLEAPFLYL
NLIIKNLSKSLTENKYSQLDEEQPMEID*
IGERSGARSKQRRPQGLPNNTASWFTALTQHGKEDLKFPRGQGVPINTNSSPDDQIGYYRRATRRIRGGDGK
TICLLQFAYANRNRFLYIIKLIFLWLLWPVTLACFVLAAVYRINWITGGIAIAMACLVGLMWLSYFIASFRLFARTF
IGERSGARSKQRRPQGLPNNTASWFTALTQHGKEDLKFPRGQGVPINTNSSPDDQIGYYRRATRRIRGGDGK
“20_leader_seq” refers to the 20 nucleotides before the end of leader, and “AUG_postion” and

5 and 10143083, excluding the mapped reads unpaired, not primary alignment and supplement

FGTTLDSKTQSLLIVNNATNVVIKVCEFQFCNDPFLGVYYHKNNKSWMESEFRVYSSANNCTFEYVSQPFLM
YALVYFLQSINFVRIIMRLWLCWKCRSKNPLLYDANYFLCWHTNCYDYCIPYNSVTSSIVITSGDGTTSPISEHD

RSMWSFNPETNILLNVPLHGTILTRPLLESELVIGAVILRGHLRIAGHHLGRCDIKDLPKEITVATSRTLSYYKLGA

ITLCFTLKRKTE*
FLEYHDVRVVLDFI*
MKDLSPRWYFYYLGTGPEAGLPYGANKDGIIWVATEGALNTPKDHIGTRNPANNAAIVLQLPQGTTLPKGF
FGTTLDSKTQSLLIVNNATNVVIKVCEFQFCNDPFLGVYYHKNNKSWMESEFRVYSSANNCTFEYVSQPFLM
YALVYFLQSINFVRIIMRLWLCWKCRSKNPLLYDANYFLCWHTNCYDYCIPYNSVTSSIVITSGDGTTSPISEHD

RSMWSFNPETNILLNVPLHGTILTRPLLESELVIGAVILRGHLRIAGHHLGRCDIKDLPKEITVATSRTLSYYKLGA

ITLCFTLKRKTE*
FLEYHDVRVVLDFI*
MKDLSPRWYFYYLGTGPEAGLPYGANKDGIIWVATEGALNTPKDHIGTRNPANNAAIVLQLPQGTTLPKGF
FGTTLDSKTQSLLIVNNATNVVIKVCEFQFCNDPFLGVYYHKNNKSWMESEFRVYSSANNCTFEYVSQPFLM
YALVYFLQSINFVRIIMRLWLCWKCRSKNPLLYDANYFLCWHTNCYDYCIPYNSVTSSIVITSGDGTTSPISEHD

RSMWSFNPETNILLNVPLHGTILTRPLLESELVIGAVILRGHLRIAGHHLGRCDIKDLPKEITVATSRTLSYYKLGA

ITLCFTLKRKTE*
MKDLSPRWYFYYLGTGPEAGLPYGANKDGIIWVATEGALNTPKDHIGTRNPANNAAIVLQLPQGTTLPKGF
FGTTLDSKTQSLLIVNNATNVVIKVCEFQFCNDPFLGVYYHKNNKSWMESEFRVYSSANNCTFEYVSQPFLM
YALVYFLQSINFVRIIMRLWLCWKCRSKNPLLYDANYFLCWHTNCYDYCIPYNSVTSSIVITSGDGTTSPISEHD
RSMWSFNPETNILLNVPLHGTILTRPLLESELVIGAVILRGHLRIAGHHLGRCDIKDLPKEITVATSRTLSYYKLGA

ITLCFTLKRKTE*
FLEYHDVRVVLDFI*
MKDLSPRWYFYYLGTGPEAGLPYGANKDGIIWVATEGALNTPKDHIGTRNPANNAAIVLQLPQGTTLPKGF
FGTTLDSKTQSLLIVNNATNVVIKVCEFQFCNDPFLGVYYHKNNKSWMESEFRVYSSANNCTFEYVSQPFLM
YALVYFLQSINFVRIIMRLWLCWKCRSKNPLLYDANYFLCWHTNCYDYCIPYNSVTSSIVITSGDGTTSPISEHD

RSMWSFNPETNILLNVPLHGTILTRPLLESELVIGAVILRGHLRIAGHHLGRCDIKDLPKEITVATSRTLSYYKLGA
ITLCFTLKRKTE*

FLEYHDVRVVLDFI*
MKDLSPRWYFYYLGTGPEAGLPYGANKDGIIWVATEGALNTPKDHIGTRNPANNAAIVLQLPQGTTLPKGF

ITLCFTLKRKTE*
MKDLSPRWYFYYLGTGPEAGLPYGANKDGIIWVATEGALNTPKDHIGTRNPANNAAIVLQLPQGTTLPKGF

FLEYHDVRVVLDFI*
IMKDLSPRWYFYYLGTGPEAGLPYGANKDGIIWVATEGALNTPKDHIGTRNPANNAAIVLQLPQGTTLPKGFY

RSMWSFNPETNILLNVPLHGTILTRPLLESELVIGAVILRGHLRIAGHHLGRCDIKDLPKEITVATSRTLSYYKLGAS
IMKDLSPRWYFYYLGTGPEAGLPYGANKDGIIWVATEGALNTPKDHIGTRNPANNAAIVLQLPQGTTLPKGFY
LYALVYFLQSINFVRIIMRLWLCWKCRSKNPLLYDANYFLCWHTNCYDYCIPYNSVTSSIVITSGDGTTSPISEHD

RSMWSFNPETNILLNVPLHGTILTRPLLESELVIGAVILRGHLRIAGHHLGRCDIKDLPKEITVATSRTLSYYKLGAS

IMKDLSPRWYFYYLGTGPEAGLPYGANKDGIIWVATEGALNTPKDHIGTRNPANNAAIVLQLPQGTTLPKGFY
FGTTLDSKTQSLLIVNNATNVVIKVCEFQFCNDPFLGVYYHKNNKSWMESEFRVYSSANNCTFEYVSQPFLMD
LYALVYFLQSINFVRIIMRLWLCWKCRSKNPLLYDANYFLCWHTNCYDYCIPYNSVTSSIVITSGDGTTSPISEHD

IMKDLSPRWYFYYLGTGPEAGLPYGANKDGIIWVATEGALNTPKDHIGTRNPANNAAIVLQLPQGTTLPKGFY
RSMWSFNPETNILLNVPLHGTILTRPLLESELVIGAVILRGHLRIAGHHLGRCDIKDLPKEITVATSRTLSYYKLGAS
IMKDLSPRWYFYYLGTGPEAGLPYGANKDGIIWVATEGALNTPKDHIGTRNPANNAAIVLQLPQGTTLPKGFY
"first_orf_aa" refer to the first AUG position and translated orf of the sgmRNA.

ary alignment.

[illegible]LEGKQGNFKNLREFVFKNIDGYFKIYSKHTPINLVRDLPQGFSALEPLVDLPIGINITRFQTLLALHRSYLTPGDS
YQIGGYTEKWESGVKDCVVLHSYFTSDYYQLYSTQLSTDTGVEHVTFFIYNKIVDEPEEHVQIHTIDGSSGVVN

SQRVAGDSGFAAYSRYRIGNYKLNTDHSSSSDNIALLVQ*

YAEGSRGGSQASSRSSSRSRNSSRNSTPGSSRGTSPARMAGNGGDAALALLLLDRLNQLESKMSGKGQQQQ
[illegible]LEGKQGNFKNLREFVFKNIDGYFKIYSKHTPINLVRDLPQGFSALEPLVDLPIGINITRFQTLLALHRSYLTPGDS
YQIGGYTEKWESGVKDCVVLHSYFTSDYYQLYSTQLSTDTGVEHVTFFIYNKIVDEPEEHVQIHTIDGSSGVVN

SQRVAGDSGFAAYSRYRIGNYKLNTDHSSSSDNIALLVQ*

YAEGSRGGSQASSRSSSRSRNSSRNSTPGSSRGTSPARMAGNGGDAALALLLLDRLNQLESKMSGKGQQQQ
[illegible]LEGKQGNFKNLREFVFKNIDGYFKIYSKHTPINLVRDLPQGFSALEPLVDLPIGINITRFQTLLALHRSYLTPGDS
YQIGGYTEKWESGVKDCVVLHSYFTSDYYQLYSTQLSTDTGVEHVTFFIYNKIVDEPEEHVQIHTIDGSSGVVN

SQRVAGDSGFAAYSRYRIGNYKLNTDHSSSSDNIALLVQ*

YAEGSRGGSQASSRSSSRSRNSSRNSTPGSSRGTSPARMAGNGGDAALALLLLDRLNQLESKMSGKGQQQQ
[illegible]LEGKQGNFKNLREFVFKNIDGYFKIYSKHTPINLVRDLPQGFSALEPLVDLPIGINITRFQTLLALHRSYLTPGDS
YQIGGYTEKWESGVKDCVVLHSYFTSDYYQLYSTQLSTDTGVEHVTFFIYNKIVDEPEEHVQIHTIDGSSGVVN
SQRVAGDSGFAAYSRYRIGNYKLNTDHSSSSDNIALLVQ*

YAEGSRGGSQASSRSSSRSRNSSRNSTPGSSRGTSPARMAGNGGDAALALLLLDRLNQLESKMSGKGQQQQ
[illegible]LEGKQGNFKNLREFVFKNIDGYFKIYSKHTPINLVRDLPQGFSALEPLVDLPIGINITRFQTLLALHRSYLTPGDS
YQIGGYTEKWESGVKDCVVLHSYFTSDYYQLYSTQLSTDTGVEHVTFFIYNKIVDEPEEHVQIHTIDGSSGVVN

SQRVAGDSGFAAYSRYRIGNYKLNTDHSSSSDNIALLVQ*

YAEGSRGGSQASSRSSSRSRNSSRNSTPGSSRGTSPARMAGNGGDAALALLLLDRLNQLESKMSGKGQQQQ

YAEGSRGGSQASSRSSSRSRNSSRNSTPGSSRGTSPARMAGNGGDAALALLLLDRLNQLESKMSGKGQQQQ

YAEGSRGGSQASSRSSSRSRNSSRNSTPGSSRGTSPARMAGNGGDAALALLLLDRLNQLESKMSGKGQQQQ

SQRVAGDSGFAAYSRYRIGNYKLNTDHSSSSDNIALLVQ*

YAEGSRGGSQASSRSSSRSRNSSRNSTPGSSRGTSPARMAGNGGDAALALLLLDRLNQLESKMSGKGQQQQ

YQIGGYTEKWESGVKDCVVLHSYFTSDYYQLYSTQLSTDTGVEHVTFFIYNKIVDEPEEHVQIHTIDGSSGVVN

SQRVAGDSGFAAYSRYRIGNYKLNTDHSSSSDNIALLVQ*

YAEGSRGGSQASSRSSSRSRNSSRNSTPGSSRGTSPARMAGNGGDAALALLLLDRLNQLESKMSGKGQQQQ

[illegible]LEGKQGNFKNLREFVFKNIDGYFKIYSKHTPINLVRDLPQGFSALEPLVDLPIGINITRFQTLLALHRSYLTPGDS

YQIGGYTEKWESGVKDCVVLHSYFTSDYYQLYSTQLSTDTGVEHVTFFIYNKIVDEPEEHVQIHTIDGSSGVVN

YAEGSRGGSQASSRSSSRSRNSSRNSTPGSSRGTSPARMAGNGGDAALALLLLDRLNQLESKMSGKGQQQQ

SQRVAGDSGFAAYSRYRIGNYKLNTDHSSSSDNIALLVQ*

YAEGSRGGSQASSRSSSRSRNSSRNSTPGSSRGTSPARMAGNGGDAALALLLLDRLNQLESKMSGKGQQQQ

SSGWTAGAAAYYVGYLQPRTFLLKYNENGTITDAVDCALDPLSETKCTLKSFTVEKGIYQTSNFRVQPTESIVR
PVMEPIYDEPTTTTSVPL*

GQTVTKKSAAEASKKPRQKRTATKAYNVTQAFGRRGPEQTQGNFGDQELIRQGTDYKHWPQIAQFAPSAS
SSGWTAGAAAYYVGYLQPRTFLLKYNENGTITDAVDCALDPLSETKCTLKSFTVEKGIYQTSNFRVQPTESIVR
PVMEPIYDEPTTTTSVPL*

GQTVTKKSAAEASKKPRQKRTATKAYNVTQAFGRRGPEQTQGNFGDQELIRQGTDYKHWPQIAQFAPSAS
SSGWTAGAAAYYVGYLQPRTFLLKYNENGTITDAVDCALDPLSETKCTLKSFTVEKGIYQTSNFRVQPTESIVR
PVMEPIYDEPTTTTSVPL*

GQTVTKKSAAEASKKPRQKRTATKAYNVTQAFGRRGPEQTQGNFGDQELIRQGTDYKHWPQIAQFAPSAS
SSGWTAGAAAYYVGYLQPRTFLLKYNENGTITDAVDCALDPLSETKCTLKSFTVEKGIYQTSNFRVQPTESIVR
PVMEPIYDEPTTTTSVPL*

GQTVTKKSAAEASKKPRQKRTATKAYNVTQAFGRRGPEQTQGNFGDQELIRQGTDYKHWPQIAQFAPSAS
SSGWTAGAAAYYVGYLQPRTFLLKYNENGTITDAVDCALDPLSETKCTLKSFTVEKGIYQTSNFRVQPTESIVR
PVMEPIYDEPTTTTSVPL*

GQTVTKKSAAEASKKPRQKRTATKAYNVTQAFGRRGPEQTQGNFGDQELIRQGTDYKHWPQIAQFAPSAS

GQTVTKKSAAEASKKPRQKRTATKAYNVTQAFGRRGPEQTQGNFGDQELIRQGTDYKHWPQIAQFAPSAS

GQTVTKKSAAEASKKPRQKRTATKAYNVTQAFGRRGPEQTQGNFGDQELIRQGTDYKHWPQIAQFAPSASA

GQTVTKKSAAEASKKPRQKRTATKAYNVTQAFGRRGPEQTQGNFGDQELIRQGTDYKHWPQIAQFAPSASA
PVMEPIYDEPTTTTSVPL*

GQTVTKKSAAEASKKPRQKRTATKAYNVTQAFGRRGPEQTQGNFGDQELIRQGTDYKHWPQIAQFAPSASA
SSSGWTAGAAAYYVGYLQPRTFLLKYNENGTITDAVDCALDPLSETKCTLKSFTVEKGIYQTSNFRVQPTESIVR
PVMEPIYDEPTTTTSVPL*

GQTVTKKSAAEASKKPRQKRTATKAYNVTQAFGRRGPEQTQGNFGDQELIRQGTDYKHWPQIAQFAPSASA

GQTVTKKSAAEASKKPRQKRTATKAYNVTQAFGRRGPEQTQGNFGDQELIRQGTDYKHWPQIAQFAPSASA

FPNITNLCPFGEVFNATRFASVYAWNRKRISNCVADYSVLYNSASFSTFKCYGVSPTKLNDLCFTNVYADSFVII

AFFGMSRIGMEVTPSGTWLTYTGAIKLDDKDPNFKDQVILLNKHIDAYKTFPPTEPKKDKKKKADETQALPQR
FPNITNLCPFGEVFNATRFASVYAWNRKRISNCVADYSVLYNSASFSTFKCYGVSPTKLNDLCFTNVYADSFVII

AFFGMSRIGMEVTPSGTWLTYTGAIKLDDKDPNFKDQVILLNKHIDAYKTFPPTEPKKDKKKKADETQALPQR
FPNITNLCPFGEVFNATRFASVYAWNRKRISNCVADYSVLYNSASFSTFKCYGVSPTKLNDLCFTNVYADSFVII

AFFGMSRIGMEVTPSGTWLTYTGAIKLDDKDPNFKDQVILLNKHIDAYKTFPPTEPKKDKKKKADETQALPQR
FPNITNLCPFGEVFNATRFASVYAWNRKRISNCVADYSVLYNSASFSTFKCYGVSPTKLNDLCFTNVYADSFVII

AFFGMSRIGMEVTPSGTWLTYTGAIKLDDKDPNFKDQVILLNKHIDAYKTFPPTEPKKDKKKKADETQALPQR
FPNITNLCPFGEVFNATRFASVYAWNRKRISNCVADYSVLYNSASFSTFKCYGVSPTKLNDLCFTNVYADSFVII

AFFGMSRIGMEVTPSGTWLTYTGAIKLDDKDPNFKDQVILLNKHIDAYKTFPPTEPKKDKKKKADETQALPQR

AFFGMSRIGMEVTPSGTWLTYTGAIKLDDKDPNFKDQVILLNKHIDAYKTFPPTEPKKDKKKKADETQALPQR

AFFGMSRIGMEVTPSGTWLTYTGAIKLDDKDPNFKDQVILLNKHIDAYKTFPPTEPKKDKKKKADETQALPQR

AFFGMSRIGMEVTPSGTWLTYTGAIKLDDKDPNFKDQVILLNKHIDAYKTFPPTEPKKDKKKKADETQALPQR

AFFGMSRIGMEVTPSGTWLTYTGAIKLDDKDPNFKDQVILLNKHIDAYKTFPPTEPKKDKKKKADETQALPQR
FPNITNLCPFGEVFNATRFASVYAWNRKRISNCVADYSVLYNSASFSTFKCYGVSPTKLNDLCFTNVYADSFVII

AFFGMSRIGMEVTPSGTWLTYTGAIKLDDKDPNFKDQVILLNKHIDAYKTFPPTEPKKDKKKKADETQALPQR

AFFGMSRIGMEVTPSGTWLTYTGAIKLDDKDPNFKDQVILLNKHIDAYKTFPPTEPKKDKKKKADETQALPQR

RGDEVRQIAPGQTGKIADYNYKLPDDFTGCVIAWNSNNLDSKVGGNYNYLYRLFRKSNLKPFERDISTEIYQA[illegible]

QKKQQTVTLLPAADLDDFSKQLQQSMSSADSTQA*
RGDEVRQIAPGQTGKIADYNYKLPDDFTGCVIAWNSNNLDSKVGGNYNYLYRLFRKSNLKPFERDISTEIYQA[illegible]

QKKQQTVTLLPAADLDDFSKQLQQSMSSADSTQA*
RGDEVRQIAPGQTGKIADYNYKLPDDFTGCVIAWNSNNLDSKVGGNYNYLYRLFRKSNLKPFERDISTEIYQA[illegible]

QKKQQTVTLLPAADLDDFSKQLQQSMSSADSTQA*
RGDEVRQIAPGQTGKIADYNYKLPDDFTGCVIAWNSNNLDSKVGGNYNYLYRLFRKSNLKPFERDISTEIYQA[illegible]

QKKQQTVTLLPAADLDDFSKQLQQSMSSADSTQA*
RGDEVRQIAPGQTGKIADYNYKLPDDFTGCVIAWNSNNLDSKVGGNYNYLYRLFRKSNLKPFERDISTEIYQA[illegible]

QKKQQTVTLLPAADLDDFSKQLQQSMSSADSTQA*

QKKQQTVTLLPAADLDDFSKQLQQSMSSADSTQA*

QKKQQTVTLLPAADLDDFSKQLQQSMSSADSTQA*

QKKQQTVTLLPAADLDDFSKQLQQSMSSADSTQA*

QKKQQTVTLLPAADLDDFSKQLQQSMSSADSTQA*
RGDEVRQIAPGQTGKIADYNYKLPDDFTGCVIAWNSNNLDSKVGGNYNYLYRLFRKSNLKPFERDISTEIYQA[illegible]

QKKQQTVTLLPAADLDDFSKQLQQSMSSADSTQA*

QKKQQTVTLLPAADLDDFSKQLQQSMSSADSTQA*

GSTPCNGVEGFNCYFPLQSYGFQPTNGVGYQPYRVVVLSFELLHAPATVCGPKKSTNLVKNKCVNFNFNGLT[illegible]

GSTPCNGVEGFNCYFPLQSYGFQPTNGVGYQPYRVVVLSFELLHAPATVCGPKKSTNLVKNKCVNFNFNGLT[illegible]

GSTPCNGVEGFNCYFPLQSYGFQPTNGVGYQPYRVVVLSFELLHAPATVCGPKKSTNLVKNKCVNFNFNGLT[illegible]

GSTPCNGVEGFNCYFPLQSYGFQPTNGVGYQPYRVVVLSFELLHAPATVCGPKKSTNLVKNKCVNFNFNGLT[illegible]

GSTPCNGVEGFNCYFPLQSYGFQPTNGVGYQPYRVVVLSFELLHAPATVCGPKKSTNLVKNKCVNFNFNGLT[illegible]

GSTPCNGVEGFNCYFPLQSYGFQPTNGVGYQPYRVVVLSFELLHAPATVCGPKKSTNLVKNKCVNFNFNGLT[illegible]

GTGVLTESNKKFLPFQQFGRDIADTTDAVRDPQTLEILDITPCSFGGVSVITPGTNTSNQVAVLYQDVNCTEVP

GTGVLTESNKKFLPFQQFGRDIADTTDAVRDPQTLEILDITPCSFGGVSVITPGTNTSNQVAVLYQDVNCTEVP

GTGVLTESNKKFLPFQQFGRDIADTTDAVRDPQTLEILDITPCSFGGVSVITPGTNTSNQVAVLYQDVNCTEVP

GTGVLTESNKKFLPFQQFGRDIADTTDAVRDPQTLEILDITPCSFGGVSVITPGTNTSNQVAVLYQDVNCTEVP

GTGVLTESNKKFLPFQQFGRDIADTTDAVRDPQTLEILDITPCSFGGVSVITPGTNTSNQVAVLYQDVNCTEVP

GTGVLTESNKKFLPFQQFGRDIADTTDAVRDPQTLEILDITPCSFGGVSVITPGTNTSNQVAVLYQDVNCTEVP

PVAIHADQLTPTWRVYSTGSNVFQTRAGCLIGAEHVNNSYECDIPIGAGICASYQTQTNSPRRARSVASQSIIAY

PVAIHADQLTPTWRVYSTGSNVFQTRAGCLIGAEHVNNSYECDIPIGAGICASYQTQTNSPRRARSVASQSIIAY

PVAIHADQLTPTWRVYSTGSNVFQTRAGCLIGAEHVNNSYECDIPIGAGICASYQTQTNSPRRARSVASQSIIAY

PVAIHADQLTPTWRVYSTGSNVFQTRAGCLIGAEHVNNSYECDIPIGAGICASYQTQTNSPRRARSVASQSIIAY

PVAIHADQLTPTWRVYSTGSNVFQTRAGCLIGAEHVNNSYECDIPIGAGICASYQTQTNSPRRARSVASQSIIAY

[illegible]VAIHADQLTPTWRVYSTGSNVFQTRAGCLIGAEHVNNSYECDIPIGAGICASYQTQTNSPRRARSVASQSIIA[illegible]

YTMSLGAENSVAYSNNSIAIPTNFTISVTTEILPVSMTKTSVDCTMYICGDSTECSNLLLQYGSFCTQLNRALTGI

YTMSLGAENSVAYSNNSIAIPTNFTISVTTEILPVSMTKTSVDCTMYICGDSTECSNLLLQYGSFCTQLNRALTGI

YTMSLGAENSVAYSNNSIAIPTNFTISVTTEILPVSMTKTSVDCTMYICGDSTECSNLLLQYGSFCTQLNRALTGI

YTMSLGAENSVAYSNNSIAIPTNFTISVTTEILPVSMTKTSVDCTMYICGDSTECSNLLLQYGSFCTQLNRALTGI

YTMSLGAENSVAYSNNSIAIPTNFTISVTTEILPVSMTKTSVDCTMYICGDSTECSNLLLQYGSFCTQLNRALTGI

YTMSLGAENSVAYSNNSIAIPTNFTISVTTEILPVSMTKTSVDCTMYICGDSTECSNLLLQYGSFCTQLNRALTGI

AVEQDKNTQEVFAQVKQIYKTPPIKDFGGFNFSQILPDPSKPSKRSFIEDLLFNKVTLADAGFIKQYGDCLGDIA

AVEQDKNTQEVFAQVKQIYKTPPIKDFGGFNFSQILPDPSKPSKRSFIEDLLFNKVTLADAGFIKQYGDCLGDIA

AVEQDKNTQEVFAQVKQIYKTPPIKDFGGFNFSQILPDPSKPSKRSFIEDLLFNKVTLADAGFIKQYGDCLGDIA

AVEQDKNTQEVFAQVKQIYKTPPIKDFGGFNFSQILPDPSKPSKRSFIEDLLFNKVTLADAGFIKQYGDCLGDIA

AVEQDKNTQEVFAQVKQIYKTPPIKDFGGFNFSQILPDPSKPSKRSFIEDLLFNKVTLADAGFIKQYGDCLGDIA

[illegible]AVEQDKNTQEVFAQVKQIYKTPPIKDFGGFNFSQILPDPSKPSKRSFIEDLLFNKVTLADAGFIKQYGDCLGDI[illegible]

AARDLICAQKFNGLTVLPPLLTDEMIAQYTSALLAGTITSGWTFGAGAALQIPFAMQMAYRFNGIGVTQNVLY

AARDLICAQKFNGLTVLPPLLTDEMIAQYTSALLAGTITSGWTFGAGAALQIPFAMQMAYRFNGIGVTQNVLY

AARDLICAQKFNGLTVLPPLLTDEMIAQYTSALLAGTITSGWTFGAGAALQIPFAMQMAYRFNGIGVTQNVLY

AARDLICAQKFNGLTVLPPLLTDEMIAQYTSALLAGTITSGWTFGAGAALQIPFAMQMAYRFNGIGVTQNVLY

AARDLICAQKFNGLTVLPPLLTDEMIAQYTSALLAGTITSGWTFGAGAALQIPFAMQMAYRFNGIGVTQNVLY

AARDLICAQKFNGLTVLPPLLTDEMIAQYTSALLAGTITSGWTFGAGAALQIPFAMQMAYRFNGIGVTQNVLY

ENQKLIANQFNSAIGKIQDSLSSTASALGKLQDVVNQNAQALNTLVKQLSSNFGAISSVLNDILSRLDKVEAE

ENQKLIANQFNSAIGKIQDSLSSTASALGKLQDVVNQNAQALNTLVKQLSSNFGAISSVLNDILSRLDKVEAE

ENQKLIANQFNSAIGKIQDSLSSTASALGKLQDVVNQNAQALNTLVKQLSSNFGAISSVLNDILSRLDKVEAE

ENQKLIANQFNSAIGKIQDSLSSTASALGKLQDVVNQNAQALNTLVKQLSSNFGAISSVLNDILSRLDKVEAE

ENQKLIANQFNSAIGKIQDSLSSTASALGKLQDVVNQNAQALNTLVKQLSSNFGAISSVLNDILSRLDKVEAE

[illegible]ENQKLIANQFNSAIGKIQDSLSSTASALGKLQDVVNQNAQALNTLVKQLSSNFGAISSVLNDILSRLDKVEAE[illegible]

[illegible]QIDRLITGRLQSLQTYVTQQLIRAAEIRASANL

[illegible]QIDRLITGRLQSLQTYVTQQLIRAAEIRASANL

[illegible]QIDRLITGRLQSLQTYVTQQLIRAAEIRASANL

[illegible]QIDRLITGRLQSLQTYVTQQLIRAAEIRASANL

[illegible]QIDRLITGRLQSLQTYVTQQLIRAAEIRASANL

/QIDRLITGRLQSLQTYVTQQLIRAAEIRASANL

	subgenome	peak_leader_	peak_TRS_st	ACGAAC	ATG_postion	20_leader_se
S1-Day1	ORF3a	69	25385	yes	25393	CTTGTAGATC
	M	65	26469	yes	26523	ATCTCTTGTA
	ORF8	65	27884	yes	27894	ATCTCTTGTA
	N	65	28256	yes	28274	ATCTCTTGTA
S2-Day2	S	65	21552	yes	21563	ATCTCTTGTA
	ORF3a	69	25385	yes	25393	CTTGTAGATC
	E	69	26237	yes	26245	CTTGTAGATC
	M	65	26469	yes	26523	ATCTCTTGTA
	ORF6	69	27041	yes	27202	CTTGTAGATC
	ORF7a	69	27388	yes	27394	CTTGTAGATC
	ORF7b	65	27644	no	27756	ATCTCTTGTA
	ORF8	65	27884	yes	27894	ATCTCTTGTA
	N	64	28255	yes	28274	GATCTCTTGT
S3-Day3	ORF3a	69	25385	yes	25393	CTTGTAGATC
	M	65	26469	yes	26523	ATCTCTTGTA
	ORF7a	69	27388	yes	27394	CTTGTAGATC
	ORF8	65	27884	yes	27894	ATCTCTTGTA
	N	64	28255	yes	28274	GATCTCTTGT
S1-Day4	ORF3a	69	25385	yes	25393	CTTGTAGATC
	E	69	26237	yes	26245	CTTGTAGATC
	M	65	26469	yes	26523	ATCTCTTGTA
	ORF7a	69	27388	yes	27394	CTTGTAGATC
	N	64	28255	yes	28274	GATCTCTTGT
S2-Day5	S	65	21552	yes	21563	ATCTCTTGTA
	ORF3a	69	25385	yes	25393	CTTGTAGATC
	E	69	26237	yes	26245	CTTGTAGATC
	M	64	26468	yes	26523	GATCTCTTGT
	ORF6	69	27041	yes	27202	CTTGTAGATC
	ORF7a	69	27388	yes	27394	CTTGTAGATC
	ORF7b	65	27644	no	27756	ATCTCTTGTA
	ORF8	65	27884	yes	27894	ATCTCTTGTA
	N	64	28255	yes	28274	GATCTCTTGT
S3-Day6	ORF3a	69	25385	yes	25393	CTTGTAGATC
	E	69	26237	yes	26245	CTTGTAGATC
	M	65	26469	yes	26523	ATCTCTTGTA
	ORF6	69	27041	yes	27202	CTTGTAGATC
	ORF7a	69	27388	yes	27394	CTTGTAGATC
	N	64	28255	yes	28274	GATCTCTTGT
S2-Day8	S	65	21552	yes	21563	ATCTCTTGTA
	ORF3a	69	25385	yes	25393	CTTGTAGATC
	E	69	26237	yes	26245	CTTGTAGATC
	M	64	26468	yes	26523	GATCTCTTGT

S2-Day8	ORF6	69	27041	yes	27202	CTTGTAGATC
	ORF7a	69	27388	yes	27394	CTTGTAGATC
	ORF8	65	27884	yes	27894	ATCTCTTGTA
	N	64	28255	yes	28274	GATCTCTTGT
S3-Day9	ORF3a	69	25385	yes	25393	CTTGTAGATC
	N	65	28256	yes	28274	ATCTCTTGTA
S2-Day11	N	65	28256	yes	28274	ATCTCTTGTA
S3-Day12	N	65	28256	yes	28274	ATCTCTTGTA
S3-Day14	ORF6	69	27041	yes	27202	CTTGTAGATC
	N	65	28256	yes	28274	ATCTCTTGTA

“peak_leader” and “peak_TRS_start” point to the leader-TRS junctions in sheet "known", “ACG

TRS_seq	first_orf_aa
ACGAACTT	MDLFMRIFTIGTVTLKQGEIKDATPSDFVRATATIPIQASLPFGWLIVGVALLAVFQSASKIITL
CTAAACGAA	MADSNGTITVEELKKLLEQWNLVIGFLFLTWICLLQFAYANRNRFLYIIKLIFLWLLWPVTLAC
CTAAACGAA	MKFLVVFLGIITTVAAFHQECSLQSCTQHQPYVVDDPCPIHFYSKWYIRVGARKSAPLIELCVD
CTAAACGAA	MSDNGPQNQRNAPRITFGGPSDSTGSNQNGERSGARSKQRRPQGLPNNTASWFTALTQ
CTAAACGAA	MFVFLVLLPLVSSQCVNLTTRTQLPPAYTNSFTRGVYYPDKVFRSSVLHSTQDLFLPFFSNVT
ACGAACTT	MDLFMRIFTIGTVTLKQGEIKDATPSDFVRATATIPIQASLPFGWLIVGVALLAVFQSASKIITL
ACGAACTT	MYSFVSEETGTLIVNSVLLFLAFVVFLLVTLAILTALRLCAYCCNIVNVSLVKPSFYVYSRVKNLN
CTAAACGAA	MADSNGTITVEELKKLLEQWNLVIGFLFLTWICLLQFAYANRNRFLYIIKLIFLWLLWPVTLAC
ACGAACGCT	MFHLVDFQVTIAEILLIIMRTFKVSIWNLDYIINLIIKNLSKSLTENKYSQLDEEQPMEID*
ACGAAC	MKIILFLALITLATCELYHYQECVRGTTVLLKEPCSSGTYEGNSPFHPLADNKFALTCFSTQFAF
CTAAACTGTT	MIELSLIDFYLCFLAFLLFLVLIMLIIFWFSLELQDHNETCHA*
CTAAACGAA	MKFLVVFLGIITTVAAFHQECSLQSCTQHQPYVVDDPCPIHFYSKWYIRVGARKSAPLIELCVD
TCTAAACGA	MSDNGPQNQRNAPRITFGGPSDSTGSNQNGERSGARSKQRRPQGLPNNTASWFTALTQ
ACGAACTT	MDLFMRIFTIGTVTLKQGEIKDATPSDFVRATATIPIQASLPFGWLIVGVALLAVFQSASKIITL
CTAAACGAA	MADSNGTITVEELKKLLEQWNLVIGFLFLTWICLLQFAYANRNRFLYIIKLIFLWLLWPVTLAC
ACGAAC	MKIILFLALITLATCELYHYQECVRGTTVLLKEPCSSGTYEGNSPFHPLADNKFALTCFSTQFAF
CTAAACGAA	MKFLVVFLGIITTVAAFHQECSLQSCTQHQPYVVDDPCPIHFYSKWYIRVGARKSAPLIELCVD
TCTAAACGA	MSDNGPQNQRNAPRITFGGPSDSTGSNQNGERSGARSKQRRPQGLPNNTASWFTALTQ
ACGAACTT	MDLFMRIFTIGTVTLKQGEIKDATPSDFVRATATIPIQASLPFGWLIVGVALLAVFQSASKIITL
ACGAACTT	MYSFVSEETGTLIVNSVLLFLAFVVFLLVTLAILTALRLCAYCCNIVNVSLVKPSFYVYSRVKNLN
CTAAACGAA	MADSNGTITVEELKKLLEQWNLVIGFLFLTWICLLQFAYANRNRFLYIIKLIFLWLLWPVTLAC
ACGAAC	MKIILFLALITLATCELYHYQECVRGTTVLLKEPCSSGTYEGNSPFHPLADNKFALTCFSTQFAF
TCTAAACGA	MSDNGPQNQRNAPRITFGGPSDSTGSNQNGERSGARSKQRRPQGLPNNTASWFTALTQ
CTAAACGAA	MFVFLVLLPLVSSQCVNLTTRTQLPPAYTNSFTRGVYYPDKVFRSSVLHSTQDLFLPFFSNVT
ACGAACTT	MDLFMRIFTIGTVTLKQGEIKDATPSDFVRATATIPIQASLPFGWLIVGVALLAVFQSASKIITL
ACGAACTT	MYSFVSEETGTLIVNSVLLFLAFVVFLLVTLAILTALRLCAYCCNIVNVSLVKPSFYVYSRVKNLN
TCTAAACGA	MADSNGTITVEELKKLLEQWNLVIGFLFLTWICLLQFAYANRNRFLYIIKLIFLWLLWPVTLAC
ACGAACGCT	MFHLVDFQVTIAEILLIIMRTFKVSIWNLDYIINLIIKNLSKSLTENKYSQLDEEQPMEID*
ACGAAC	MKIILFLALITLATCELYHYQECVRGTTVLLKEPCSSGTYEGNSPFHPLADNKFALTCFSTQFAF
CTAAACTGTT	MIELSLIDFYLCFLAFLLFLVLIMLIIFWFSLELQDHNETCHA*
CTAAACGAA	MKFLVVFLGIITTVAAFHQECSLQSCTQHQPYVVDDPCPIHFYSKWYIRVGARKSAPLIELCVD
TCTAAACGA	MSDNGPQNQRNAPRITFGGPSDSTGSNQNGERSGARSKQRRPQGLPNNTASWFTALTQ
ACGAACTT	MDLFMRIFTIGTVTLKQGEIKDATPSDFVRATATIPIQASLPFGWLIVGVALLAVFQSASKIITL
ACGAACTT	MYSFVSEETGTLIVNSVLLFLAFVVFLLVTLAILTALRLCAYCCNIVNVSLVKPSFYVYSRVKNLN
CTAAACGAA	MADSNGTITVEELKKLLEQWNLVIGFLFLTWICLLQFAYANRNRFLYIIKLIFLWLLWPVTLAC
ACGAACGCT	MFHLVDFQVTIAEILLIIMRTFKVSIWNLDYIINLIIKNLSKSLTENKYSQLDEEQPMEID*
ACGAAC	MKIILFLALITLATCELYHYQECVRGTTVLLKEPCSSGTYEGNSPFHPLADNKFALTCFSTQFAF
TCTAAACGA	MSDNGPQNQRNAPRITFGGPSDSTGSNQNGERSGARSKQRRPQGLPNNTASWFTALTQ
CTAAACGAA	MFVFLVLLPLVSSQCVNLTTRTQLPPAYTNSFTRGVYYPDKVFRSSVLHSTQDLFLPFFSNVT
ACGAACTT	MDLFMRIFTIGTVTLKQGEIKDATPSDFVRATATIPIQASLPFGWLIVGVALLAVFQSASKIITL
ACGAACTT	MYSFVSEETGTLIVNSVLLFLAFVVFLLVTLAILTALRLCAYCCNIVNVSLVKPSFYVYSRVKNLN
TCTAAACGA	MADSNGTITVEELKKLLEQWNLVIGFLFLTWICLLQFAYANRNRFLYIIKLIFLWLLWPVTLAC

ACGAACGCT[illegible]	MFHLVDFQVTIAEILLIIMRTFKVSIWNLDYIINLIIKNLSKSLTENKYSQLDEEQPMEID*
ACGAAC	MKIILFLALITLATCELYHYQECVRGTTVLLKEPCSSGTYEGNSPFHPLADNKFALTCFSTQFAF[illegible]
CTAAACGAA[illegible]	MKFLVFLGIITTVAAFHQECSLQSCTQHQPYVVDDPCPIHFYSKWYIRVGARKSAPLIELCVD[illegible]
TCTAAACGA[illegible]	MSDNGPQNQRNAPRITFGGPSDSTGSNQNGERSGARSKQRRPQGLPNNTASWFTALTQ[illegible]
ACGAACTT	MDLFMRIFTIGTVTLKQGEIKDATPSDFVRATATIPIQASLPFGWLIVGVALLAVFQSASKIITL[illegible]
CTAAACGAA[illegible]	MSDNGPQNQRNAPRITFGGPSDSTGSNQNGERSGARSKQRRPQGLPNNTASWFTALTQ[illegible]
CTAAACGAA[illegible]	MSDNGPQNQRNAPRITFGGPSDSTGSNQNGERSGARSKQRRPQGLPNNTASWFTALTQ[illegible]
CTAAACGAA[illegible]	MSDNGPQNQRNAPRITFGGPSDSTGSNQNGERSGARSKQRRPQGLPNNTASWFTALTQ[illegible]
ACGAACGCT[illegible]	MFHLVDFQVTIAEILLIIMRTFKVSIWNLDYIINLIIKNLSKSLTENKYSQLDEEQPMEID*
CTAAACGAA[illegible]	MSDNGPQNQRNAPRITFGGPSDSTGSNQNGERSGARSKQRRPQGLPNNTASWFTALTQ[illegible]

[illegible]AAC” indicates if there is a ACGAAC sequence in the “TRS_seq” (TRS sequences), “20_leader_se[illegible]

KKRWQLALSKGVHFVCNLLLLFVTVYSHLLLVAAGLEAPFLYLYALVYFLQSINFVRIIMRLWLCWKCRSKNPLL

FVLAAVYRINWITGGIAIAMACLVGLMWLSYFIASFRLFARTRSMWSFNPETNILLNVPLHGTILTRPLLESELV

EAGSKSPIQYIDIGNYTVSCLPFTINCQEPKLGSLVVRCSFYEDFLEYHDVRVVLDFI*

HGKEDLKFPRGQGVPINTNSSPDDQIGYYRRATRRIRGGDGKMKDLSPRWYFYYLGTGPEAGLPYGANKDGII

WFHAIHVSGTNGTKRFDNPVLPFNDGVYFASTEKSNIIRGWIFGTTLDSKTQSLLIVNNATNVVIKVCEFQFCN

KKRWQLALSKGVHFVCNLLLLFVTVYSHLLLVAAGLEAPFLYLYALVYFLQSINFVRIIMRLWLCWKCRSKNPLL

ISSRVPDLLV*

FVLAAVYRINWITGGIAIAMACLVGLMWLSYFIASFRLFARTRSMWSFNPETNILLNVPLHGTILTRPLLESELV

ACPDGVKHVYQLRARSVSPKLFIRQEEVQELYSPIFLIVAAIVFITLCFTLKRKTE*

EAGSKSPIQYIDIGNYTVSCLPFTINCQEPKLGSLVVRCSFYEDFLEYHDVRVVLDFI*

HGKEDLKFPRGQGVPINTNSSPDDQIGYYRRATRRIRGGDGKMKDLSPRWYFYYLGTGPEAGLPYGANKDGII

KKRWQLALSKGVHFVCNLLLLFVTVYSHLLLVAAGLEAPFLYLYALVYFLQSINFVRIIMRLWLCWKCRSKNPLL

FVLAAVYRINWITGGIAIAMACLVGLMWLSYFIASFRLFARTRSMWSFNPETNILLNVPLHGTILTRPLLESELV

ACPDGVKHVYQLRARSVSPKLFIRQEEVQELYSPIFLIVAAIVFITLCFTLKRKTE*

EAGSKSPIQYIDIGNYTVSCLPFTINCQEPKLGSLVVRCSFYEDFLEYHDVRVVLDFI*

HGKEDLKFPRGQGVPINTNSSPDDQIGYYRRATRRIRGGDGKMKDLSPRWYFYYLGTGPEAGLPYGANKDGII

KKRWQLALSKGVHFVCNLLLLFVTVYSHLLLVAAGLEAPFLYLYALVYFLQSINFVRIIMRLWLCWKCRSKNPLL

ISSRVPDLLV*

FVLAAVYRINWITGGIAIAMACLVGLMWLSYFIASFRLFARTRSMWSFNPETNILLNVPLHGTILTRPLLESELV

ACPDGVKHVYQLRARSVSPKLFIRQEEVQELYSPIFLIVAAIVFITLCFTLKRKTE*

HGKEDLKFPRGQGVPINTNSSPDDQIGYYRRATRRIRGGDGKMKDLSPRWYFYYLGTGPEAGLPYGANKDGII

WFHAIHVSGTNGTKRFDNPVLPFNDGVYFASTEKSNIIRGWIFGTTLDSKTQSLLIVNNATNVVIKVCEFQFCN

KKRWQLALSKGVHFVCNLLLLFVTVYSHLLLVAAGLEAPFLYLYALVYFLQSINFVRIIMRLWLCWKCRSKNPLL

ISSRVPDLLV*

FVLAAVYRINWITGGIAIAMACLVGLMWLSYFIASFRLFARTRSMWSFNPETNILLNVPLHGTILTRPLLESELV

ACPDGVKHVYQLRARSVSPKLFIRQEEVQELYSPIFLIVAAIVFITLCFTLKRKTE*

EAGSKSPIQYIDIGNYTVSCLPFTINCQEPKLGSLVVRCSFYEDFLEYHDVRVVLDFI*

HGKEDLKFPRGQGVPINTNSSPDDQIGYYRRATRRIRGGDGKMKDLSPRWYFYYLGTGPEAGLPYGANKDGII

KKRWQLALSKGVHFVCNLLLLFVTVYSHLLLVAAGLEAPFLYLYALVYFLQSINFVRIIMRLWLCWKCRSKNPLL

ISSRVPDLLV*

FVLAAVYRINWITGGIAIAMACLVGLMWLSYFIASFRLFARTRSMWSFNPETNILLNVPLHGTILTRPLLESELV

ACPDGVKHVYQLRARSVSPKLFIRQEEVQELYSPIFLIVAAIVFITLCFTLKRKTE*

HGKEDLKFPRGQGVPINTNSSPDDQIGYYRRATRRIRGGDGKMKDLSPRWYFYYLGTGPEAGLPYGANKDGII

WFHAIHVSGTNGTKRFDNPVLPFNDGVYFASTEKSNIIRGWIFGTTLDSKTQSLLIVNNATNVVIKVCEFQFCN

KKRWQLALSKGVHFVCNLLLLFVTVYSHLLLVAAGLEAPFLYLYALVYFLQSINFVRIIMRLWLCWKCRSKNPLL

ISSRVPDLLV*

FVLAAVYRINWITGGIAIAMACLVGLMWLSYFIASFRLFARTRSMWSFNPETNILLNVPLHGTILTRPLLESELV

ACPDGVKHVYQLRARSVSPKLFIRQEEVQELYSPIFLIVAAIVFITLCFTLKRKTE*
EAGSKSPIQYIDIGNYTVSCLPFTINCQEPKLGSLVVRCSFYEDFLEYHDVRVVLDFI*
HGKEDLKFPRGQGVPINTNSSPDDQIGYYRRATRRIRGGDGKMKDLSPRWYFYYLGTGPEAGLPYGANKDGII
KKRWQLALSKGVHFVCNLLLLFVTVYSHLLLVAAGLEAPFLYLYALVYFLQSINFVRIIMRLWLCWKCRSKNPLL
HGKEDLKFPRGQGVPINTNSSPDDQIGYYRRATRRIRGGDGKMKDLSPRWYFYYLGTGPEAGLPYGANKDGII
HGKEDLKFPRGQGVPINTNSSPDDQIGYYRRATRRIRGGDGKMKDLSPRWYFYYLGTGPEAGLPYGANKDGII
HGKEDLKFPRGQGVPINTNSSPDDQIGYYRRATRRIRGGDGKMKDLSPRWYFYYLGTGPEAGLPYGANKDGII

HGKEDLKFPRGQGVPINTNSSPDDQIGYYRRATRRIRGGDGKMKDLSPRWYFYYLGTGPEAGLPYGANKDGII
q” refers to the 20 nucleotides before the end of leader, and “AUG_postion” and “first_orf_aa”

YDANYFLCWHTNCYDYCIPYNSVTSSIVITSGDGTTSPISEHDYQIGGYTEKWESGVKDCVVLHSYFTSDYYQL
IGAVILRGHLRIAGHHLGRCDIKDLPKEITVATSRTLSYYKLGASQRVAGDSGFAAYSRYRIGNYKLNTDHSSSD

IWVATEGALNTPKDHIGTRNPANNAAIVLQLPQGTTLPKGFYAEGSRGGSQASSRSSSRSRNSSRNSTPGSSR
DPFLGVYYHKNNKSWMESEFRVYSSANNCTFEYVSQPFLMDLEGKQGNFKNLREFVFKNIDGYFKIYSKHTPI
YDANYFLCWHTNCYDYCIPYNSVTSSIVITSGDGTTSPISEHDYQIGGYTEKWESGVKDCVVLHSYFTSDYYQL

IGAVILRGHLRIAGHHLGRCDIKDLPKEITVATSRTLSYYKLGASQRVAGDSGFAAYSRYRIGNYKLNTDHSSSD

IWVATEGALNTPKDHIGTRNPANNAAIVLQLPQGTTLPKGFYAEGSRGGSQASSRSSSRSRNSSRNSTPGSSR
YDANYFLCWHTNCYDYCIPYNSVTSSIVITSGDGTTSPISEHDYQIGGYTEKWESGVKDCVVLHSYFTSDYYQL
IGAVILRGHLRIAGHHLGRCDIKDLPKEITVATSRTLSYYKLGASQRVAGDSGFAAYSRYRIGNYKLNTDHSSSD

IWVATEGALNTPKDHIGTRNPANNAAIVLQLPQGTTLPKGFYAEGSRGGSQASSRSSSRSRNSSRNSTPGSSR
YDANYFLCWHTNCYDYCIPYNSVTSSIVITSGDGTTSPISEHDYQIGGYTEKWESGVKDCVVLHSYFTSDYYQL

IGAVILRGHLRIAGHHLGRCDIKDLPKEITVATSRTLSYYKLGASQRVAGDSGFAAYSRYRIGNYKLNTDHSSSD

IWVATEGALNTPKDHIGTRNPANNAAIVLQLPQGTTLPKGFYAEGSRGGSQASSRSSSRSRNSSRNSTPGSSR
DPFLGVYYHKNNKSWMESEFRVYSSANNCTFEYVSQPFLMDLEGKQGNFKNLREFVFKNIDGYFKIYSKHTPI
YDANYFLCWHTNCYDYCIPYNSVTSSIVITSGDGTTSPISEHDYQIGGYTEKWESGVKDCVVLHSYFTSDYYQL

IGAVILRGHLRIAGHHLGRCDIKDLPKEITVATSRTLSYYKLGASQRVAGDSGFAAYSRYRIGNYKLNTDHSSSD

IWVATEGALNTPKDHIGTRNPANNAAIVLQLPQGTTLPKGFYAEGSRGGSQASSRSSSRSRNSSRNSTPGSSR
YDANYFLCWHTNCYDYCIPYNSVTSSIVITSGDGTTSPISEHDYQIGGYTEKWESGVKDCVVLHSYFTSDYYQL

IGAVILRGHLRIAGHHLGRCDIKDLPKEITVATSRTLSYYKLGASQRVAGDSGFAAYSRYRIGNYKLNTDHSSSD

IWVATEGALNTPKDHIGTRNPANNAAIVLQLPQGTTLPKGFYAEGSRGGSQASSRSSSRSRNSSRNSTPGSSR
DPFLGVYYHKNNKSWMESEFRVYSSANNCTFEYVSQPFLMDLEGKQGNFKNLREFVFKNIDGYFKIYSKHTPI
YDANYFLCWHTNCYDYCIPYNSVTSSIVITSGDGTTSPISEHDYQIGGYTEKWESGVKDCVVLHSYFTSDYYQL

IGAVILRGHLRIAGHHLGRCDIKDLPKEITVATSRTLSYYKLGASQRVAGDSGFAAYSRYRIGNYKLNTDHSSSD

IWVATEGALNTPKDHIGTRNPANNAAIVLQLPQGTTLPKGFYAEGSRGGSQASSRSSSRSRNSSRNSTPGSSR
YDANYFLCWHTNCYDYCIPYNSVTSSIVITSGDGTTSPISEHDYQIGGYTEKWESGVKDCVVLHSYFTSDYYQL
IWVATEGALNTPKDHIGTRNPANNAAIVLQLPQGTTLPKGFYAEGSRGGSQASSRSSSRSRNSSRNSTPGSSR
IWVATEGALNTPKDHIGTRNPANNAAIVLQLPQGTTLPKGFYAEGSRGGSQASSRSSSRSRNSSRNSTPGSSR
IWVATEGALNTPKDHIGTRNPANNAAIVLQLPQGTTLPKGFYAEGSRGGSQASSRSSSRSRNSSRNSTPGSSR

IWVATEGALNTPKDHIGTRNPANNAAIVLQLPQGTTLPKGFYAEGSRGGSQASSRSSSRSRNSSRNSTPGSSR
refer to the first AUG position and translated orf of the sgmRNA.

YSTQLSTDTGVEHVTFFIYNKIVDEPEEHVQIHTIDGSSGVVNPVMEPIYDEPTTTTSVPL*
NIALLVQ*

GTSPARMAGNGGDAALALLLLDRLNQLESKMSGKGQQQQGQTVTKKSAAEASKKPRQKRTATKAYNVTQ
NLVRDLPQGFSALEPLVDLPIGINITRFQTLLALHRSYLTPGDSSSGWTAGAAAYYVGYLQPRTFLLKYNENGT
YSTQLSTDTGVEHVTFFIYNKIVDEPEEHVQIHTIDGSSGVVNPVMEPIYDEPTTTTSVPL*

NIALLVQ*

GTSPARMAGNGGDAALALLLLDRLNQLESKMSGKGQQQQGQTVTKKSAAEASKKPRQKRTATKAYNVTQ
YSTQLSTDTGVEHVTFFIYNKIVDEPEEHVQIHTIDGSSGVVNPVMEPIYDEPTTTTSVPL*
NIALLVQ*

GTSPARMAGNGGDAALALLLLDRLNQLESKMSGKGQQQQGQTVTKKSAAEASKKPRQKRTATKAYNVTQ
YSTQLSTDTGVEHVTFFIYNKIVDEPEEHVQIHTIDGSSGVVNPVMEPIYDEPTTTTSVPL*

NIALLVQ*

GTSPARMAGNGGDAALALLLLDRLNQLESKMSGKGQQQQGQTVTKKSAAEASKKPRQKRTATKAYNVTQ
NLVRDLPQGFSALEPLVDLPIGINITRFQTLLALHRSYLTPGDSSSGWTAGAAAYYVGYLQPRTFLLKYNENGT
YSTQLSTDTGVEHVTFFIYNKIVDEPEEHVQIHTIDGSSGVVNPVMEPIYDEPTTTTSVPL*

NIALLVQ*

GTSPARMAGNGGDAALALLLLDRLNQLESKMSGKGQQQQGQTVTKKSAAEASKKPRQKRTATKAYNVTQ
YSTQLSTDTGVEHVTFFIYNKIVDEPEEHVQIHTIDGSSGVVNPVMEPIYDEPTTTTSVPL*

NIALLVQ*

GTSPARMAGNGGDAALALLLLDRLNQLESKMSGKGQQQQGQTVTKKSAAEASKKPRQKRTATKAYNVTQ
NLVRDLPQGFSALEPLVDLPIGINITRFQTLLALHRSYLTPGDSSSGWTAGAAAYYVGYLQPRTFLLKYNENGT
YSTQLSTDTGVEHVTFFIYNKIVDEPEEHVQIHTIDGSSGVVNPVMEPIYDEPTTTTSVPL*

NIALLVQ*

GTSPARMAGNGGDAALALLLLDRLNQLESKMSGKGQQQQGQTVTKKSAAEASKKPRQKRTATKAYNVTQ
YSTQLSTDTGVEHVTFFIYNKIVDEPEEHVQIHTIDGSSGVVNPVMEPIYDEPTTTTSVPL*
GTSPARMAGNGGDAALALLLLDRLNQLESKMSGKGQQQQGQTVTKKSAAEASKKPRQKRTATKAYNVTQ
GTSPARMAGNGGDAALALLLLDRLNQLESKMSGKGQQQQGQTVTKKSAAEASKKPRQKRTATKAYNVTQ
GTSPARMAGNGGDAALALLLLDRLNQLESKMSGKGQQQQGQTVTKKSAAEASKKPRQKRTATKAYNVTQ

GTSPARMAGNGGDAALALLLLDRLNQLESKMSGKGQQQQGQTVTKKSAAEASKKPRQKRTATKAYNVTQ

AFGRRGPEQTQGNFGDQELIRQGTDYKHWPQIAQFAPSASAFFGMSRIGMEVTPSGTWLTYTGAIKLDDKD
ITDAVDCALDPLSETKCTLKSFTVEKGIYQTSNFRVQPTESIVRFPNITNLCPFGEVFNATRFASVYAWNRKRISN

AFGRRGPEQTQGNFGDQELIRQGTDYKHWPQIAQFAPSASAFFGMSRIGMEVTPSGTWLTYTGAIKLDDKD

AFGRRGPEQTQGNFGDQELIRQGTDYKHWPQIAQFAPSASAFFGMSRIGMEVTPSGTWLTYTGAIKLDDKD

AFGRRGPEQTQGNFGDQELIRQGTDYKHWPQIAQFAPSASAFFGMSRIGMEVTPSGTWLTYTGAIKLDDKD
ITDAVDCALDPLSETKCTLKSFTVEKGIYQTSNFRVQPTESIVRFPNITNLCPFGEVFNATRFASVYAWNRKRISN

AFGRRGPEQTQGNFGDQELIRQGTDYKHWPQIAQFAPSASAFFGMSRIGMEVTPSGTWLTYTGAIKLDDKD

AFGRRGPEQTQGNFGDQELIRQGTDYKHWPQIAQFAPSASAFFGMSRIGMEVTPSGTWLTYTGAIKLDDKD
ITDAVDCALDPLSETKCTLKSFTVEKGIYQTSNFRVQPTESIVRFPNITNLCPFGEVFNATRFASVYAWNRKRISN

AFGRRGPEQTQGNFGDQELIRQGTDYKHWPQIAQFAPSASAFFGMSRIGMEVTPSGTWLTYTGAIKLDDKD

AFGRRGPEQTQGNFGDQELIRQGTDYKHWPQIAQFAPSASAFFGMSRIGMEVTPSGTWLTYTGAIKLDDKD
AFGRRGPEQTQGNFGDQELIRQGTDYKHWPQIAQFAPSASAFFGMSRIGMEVTPSGTWLTYTGAIKLDDKD
AFGRRGPEQTQGNFGDQELIRQGTDYKHWPQIAQFAPSASAFFGMSRIGMEVTPSGTWLTYTGAIKLDDKD

AFGRRGPEQTQGNFGDQELIRQGTDYKHWPQIAQFAPSASAFFGMSRIGMEVTPSGTWLTYTGAIKLDDKD

PNFKDQVILLNKHIDAYKTFPPTEPKKDKKKKADETQALPQRQKKQQTVTLLPAADLDDFSKQLQQSMSSAD
NCVADYSVLYNSASFSTFKCYGVSPTKLNDLCFTNVYADSFVIRGDEVRQIAPGQTGKIADYNYKLPDDFTGCV

PNFKDQVILLNKHIDAYKTFPPTEPKKDKKKKADETQALPQRQKKQQTVTLLPAADLDDFSKQLQQSMSSAD

PNFKDQVILLNKHIDAYKTFPPTEPKKDKKKKADETQALPQRQKKQQTVTLLPAADLDDFSKQLQQSMSSAD

PNFKDQVILLNKHIDAYKTFPPTEPKKDKKKKADETQALPQRQKKQQTVTLLPAADLDDFSKQLQQSMSSAD
NCVADYSVLYNSASFSTFKCYGVSPTKLNDLCFTNVYADSFVIRGDEVRQIAPGQTGKIADYNYKLPDDFTGCV

PNFKDQVILLNKHIDAYKTFPPTEPKKDKKKKADETQALPQRQKKQQTVTLLPAADLDDFSKQLQQSMSSAD

PNFKDQVILLNKHIDAYKTFPPTEPKKDKKKKADETQALPQRQKKQQTVTLLPAADLDDFSKQLQQSMSSAD
NCVADYSVLYNSASFSTFKCYGVSPTKLNDLCFTNVYADSFVIRGDEVRQIAPGQTGKIADYNYKLPDDFTGCV

PNFKDQVILLNKHIDAYKTFPPTEPKKDKKKKADETQALPQRQKKQQTVTLLPAADLDDFSKQLQQSMSSAD

PNFKDQVILLNKHIDAYKTFPPTEPKKDKKKKADETQALPQRQKKQQTVTLLPAADLDDFSKQLQQSMSSAD
PNFKDQVILLNKHIDAYKTFPPTEPKKDKKKKADETQALPQRQKKQQTVTLLPAADLDDFSKQLQQSMSSAD
PNFKDQVILLNKHIDAYKTFPPTEPKKDKKKKADETQALPQRQKKQQTVTLLPAADLDDFSKQLQQSMSSAD

PNFKDQVILLNKHIDAYKTFPPTEPKKDKKKKADETQALPQRQKKQQTVTLLPAADLDDFSKQLQQSMSSAD

STQA*
IAWNSNNLDSKVGGNYNYLYRLFRKSNLKPFERDISTEIYQAGSTPCNGVEGFNCYFPLQSYGFQPTNGVGYC

STQA*

STQA*

STQA*
IAWNSNNLDSKVGGNYNYLYRLFRKSNLKPFERDISTEIYQAGSTPCNGVEGFNCYFPLQSYGFQPTNGVGYC

STQA*

STQA*
IAWNSNNLDSKVGGNYNYLYRLFRKSNLKPFERDISTEIYQAGSTPCNGVEGFNCYFPLQSYGFQPTNGVGYC

STQA*

STQA*
STQA*
STQA*

STQA*

QPYRVVVLSFELLHAPATVCGPKKSTNLVKNKCVNFNFNGLTGTGVLTESNKKFLPFQQFGRDIADTTDAVRD[illegible]

QPYRVVVLSFELLHAPATVCGPKKSTNLVKNKCVNFNFNGLTGTGVLTESNKKFLPFQQFGRDIADTTDAVRD[illegible]

QPYRVVVLSFELLHAPATVCGPKKSTNLVKNKCVNFNFNGLTGTGVLTESNKKFLPFQQFGRDIADTTDAVRD[illegible]

PQTLEILDITPCSFGGVSVITPGTNTSNQVAVLYQDVNCTEVPVAIHADQLTPTWRVYSTGSNVFQTRAGCLI[illegible]

PQTLEILDITPCSFGGVSVITPGTNTSNQVAVLYQDVNCTEVPVAIHADQLTPTWRVYSTGSNVFQTRAGCLI[illegible]

PQTLEILDITPCSFGGVSVITPGTNTSNQVAVLYQDVNCTEVPVAIHADQLTPTWRVYSTGSNVFQTRAGCLI[illegible]

GAEHVNNSYECDIPIGAGICASYQTQTNSPRRARSVASQSIIAYTMSLGAENSVAYSNNSIAIPTNFTISVTTEILP

GAEHVNNSYECDIPIGAGICASYQTQTNSPRRARSVASQSIIAYTMSLGAENSVAYSNNSIAIPTNFTISVTTEILP

GAEHVNNSYECDIPIGAGICASYQTQTNSPRRARSVASQSIIAYTMSLGAENSVAYSNNSIAIPTNFTISVTTEILP

VSMTKTSVDCTMYICGDSTECSNLLLQYGSFCTQLNRALTGIAVEQDKNTQEVFAQVKQIYKTPPIKDFGGFN

VSMTKTSVDCTMYICGDSTECSNLLLQYGSFCTQLNRALTGIAVEQDKNTQEVFAQVKQIYKTPPIKDFGGFN

VSMTKTSVDCTMYICGDSTECSNLLLQYGSFCTQLNRALTGIAVEQDKNTQEVFAQVKQIYKTPPIKDFGGFN

NFSQILPDPSKPSKRSFIEDLLFNKVTLADAGFIKQYGDCLGDIAARDLICAQKFNGLTVLPPLLTDEMIAQYTSAI

NFSQILPDPSKPSKRSFIEDLLFNKVTLADAGFIKQYGDCLGDIAARDLICAQKFNGLTVLPPLLTDEMIAQYTSAI

NFSQILPDPSKPSKRSFIEDLLFNKVTLADAGFIKQYGDCLGDIAARDLICAQKFNGLTVLPPLLTDEMIAQYTSAI

LAGTITSGWTFGAGAALQIPFAMQMAYRFNGIGVTQNVLYENQKLIANQFNSAIGKIQDSLSSTASALGKLQ

LAGTITSGWTFGAGAALQIPFAMQMAYRFNGIGVTQNVLYENQKLIANQFNSAIGKIQDSLSSTASALGKLQ

LAGTITSGWTFGAGAALQIPFAMQMAYRFNGIGVTQNVLYENQKLIANQFNSAIGKIQDSLSSTASALGKLQ

DVVNQNAQALNTLVKQLSSNFGAISSVLNDILSRLDKVEAEVQIDRLITGRLQSLQTYVTQQLIRAAEIRASAN

DVVNQNAQALNTLVKQLSSNFGAISSVLNDILSRLDKVEAEVQIDRLITGRLQSLQTYVTQQLIRAAEIRASAN

DVVNQNAQALNTLVKQLSSNFGAISSVLNDILSRLDKVEAEVQIDRLITGRLQSLQTYVTQQLIRAAEIRASAN

L

L

L

	subgenome	leader_end	TRS_start	nb_count
S1-Day1	1	63	5785	545(544,514,513,522)
	2	63	10978	116(116,0,0,4)
	3	46	26422	60(60,59,59,54)
	4	65	10636	29(29,24,24,24)
	5	67	3362	75(75,72,72,72)
	6	67	22404	55(55,0,0,0)
	7	67	22503	227(225,1,1,4)
	8	68	4580	308(308,276,276,288)
	9	69	13619	164(164,154,154,154)
	10	47	26421	22(22,22,22,22)
	11	71	10926	12(12,0,0,2)
	12	73	3372	127(127,124,124,126)
	13	74	21058	331(330,325,324,324)
	14	74	23112	26(26,0,0,2)
	15	48	26443	123(123,122,122,122)
	16	48	28184	17(17,0,0,0)
S2-Day2	1	58	13666	29(29,0,0,0)
	2	58	26917	27(27,0,0,0)
	3	62	3808	12(12,0,0,0)
	4	63	3924	41(41,0,0,0)
	5	63	20583	19(19,0,0,0)
	6	63	23922	64(50,14,0,0)
	7	46	26422	40(40,39,39,38)
	8	67	3362	80(80,78,78,76)
	9	67	22404	74(74,0,0,2)
	10	67	22490	148(148,0,0,4)
	11	67	26866	234(228,0,0,8)
	12	68	5789	54(54,54,54,54)
	13	68	14223	34(33,24,24,22)
	14	69	3816	85(85,0,0,2)
	15	69	11356	139(139,68,68,70)
	16	69	13619	77(76,72,71,74)
	17	69	19112	473(468,468,463,460)
	18	69	19571	22(21,0,0,0)
	19	69	21305	16(16,0,0,0)
	20	69	22382	50(50,0,0,2)
	21	47	26421	20(20,19,19,18)
	22	71	5708	107(64,43,0,4)
	23	74	21058	31(31,30,30,30)
	24	74	25858	131(131,122,122,124)
	25	48	26443	77(77,76,76,74)
	26	48	28184	16(16,0,0,0)

S3-Day3	1	48	26443	12(12,12,12,12)
	2	53	17675	21(19,0,0,0)
S1-Day4	1	63	5785	23(23,23,23,22)
	2	68	5789	1967(1964,1954,1951,19[illegible]
	3	75	28265	56(50,56,50,48)
	4	48	28184	17(17,0,0,0)
S2-Day5	1	48	28184	12(12,0,0,0)
S3-Day6				
S2-Day8				
S3-Day9				
S2-Day11	1	51	27203	26(21,0,0,2)
	2	58	29871	56(7,0,0,0)
S3-Day12	1	76	28267	18(17,17,16,0)
	2	48	28184	35(35,0,0,0)
S3-Day14				

The numbers in the bracket are (reads with left primers, reads with right primer[illegible]
Normalized count=(Read count/Total number of read mapped on reference gen[illegible]
Total number of read mapped on reference genome is 36593897, 37733596, 33[illegible]
“leader_end” and “TRS_start” refer to the position of the end of leader and the [illegible]

normalized_count
14.89(14.87,14.05,14.02,14.26)
3.17(3.17,0.00,0.00,0.11)
1.64(1.64,1.61,1.61,1.48)
0.79(0.79,0.66,0.66,0.66)
2.05(2.05,1.97,1.97,1.97)
1.50(1.50,0.00,0.00,0.00)
6.20(6.15,0.03,0.03,0.11)
8.42(8.42,7.54,7.54,7.87)
4.48(4.48,4.21,4.21,4.21)
0.60(0.60,0.60,0.60,0.60)
0.33(0.33,0.00,0.00,0.05)
3.47(3.47,3.39,3.39,3.44)
9.05(9.02,8.88,8.85,8.85)
0.71(0.71,0.00,0.00,0.05)
3.36(3.36,3.33,3.33,3.33)
0.46(0.46,0.00,0.00,0.00)
0.77(0.77,0.00,0.00,0.00)
0.72(0.72,0.00,0.00,0.00)
0.32(0.32,0.00,0.00,0.00)
1.09(1.09,0.00,0.00,0.00)
0.50(0.50,0.00,0.00,0.00)
1.70(1.33,0.37,0.00,0.00)
1.06(1.06,1.03,1.03,1.01)
2.12(2.12,2.07,2.07,2.01)
1.96(1.96,0.00,0.00,0.05)
3.92(3.92,0.00,0.00,0.11)
6.20(6.04,0.00,0.00,0.21)
1.43(1.43,1.43,1.43,1.43)
0.90(0.87,0.64,0.64,0.58)
2.25(2.25,0.00,0.00,0.05)
3.68(3.68,1.80,1.80,1.86)
2.04(2.01,1.91,1.88,1.96)
12.54(12.40,12.40,12.27,12.19)
0.58(0.56,0.00,0.00,0.00)
0.42(0.42,0.00,0.00,0.00)
1.33(1.33,0.00,0.00,0.05)
0.53(0.53,0.50,0.50,0.48)
2.84(1.70,1.14,0.00,0.11)
0.82(0.82,0.80,0.80,0.80)
3.47(3.47,3.23,3.23,3.29)
2.04(2.04,2.01,2.01,1.96)
0.42(0.42,0.00,0.00,0.00)

0.36(0.36,0.36,0.36,0.36)
0.63(0.57,0.00,0.00,0.00)
0.68(0.68,0.68,0.68,0.65)
57.94(57.85,57.56,57.47,56.73)
1.65(1.47,1.65,1.47,1.41)
0.50(0.50,0.00,0.00,0.00)
0.43(0.43,0.00,0.00,0.00)

1.50(1.21,0.00,0.00,0.12)
3.23(0.40,0.00,0.00,0.00)
1.06(1.00,1.00,0.94,0.00)
2.06(2.06,0.00,0.00,0.00)

s, reads with both primers, same junction on paired reads with at least a primer).
ome)*1000000.
222295, 33949503, 28220017, 28624584, 16048939, 12949851, 17312079, 17029467, 20512435 and 1
position of the start of TRS identified in the reads >10.

10143083, excluding the mapped reads unpaired, not primary alignment and supplementary alig

gnment.

	subgenome	leader_end	TRS_start	nb_count	normalized_count
S1-Day1					
	1	46	26422	69(69,69,69,6	1.92(1.92,1.92,1.92,1.78)
	2	66	13865	21(21,0,0,0)	0.58(0.58,0.00,0.00,0.00)
	3	68	2689	87(74,83,70,6	2.42(2.06,2.31,1.95,1.84)
	4	70	14372	44(44,0,0,0)	1.22(1.22,0.00,0.00,0.00)
	5	71	27762	13(13,10,10,1	0.36(0.36,0.28,0.28,0.28)
S2-Day2	6	74	21058	664(659,660,	18.47(18.33,18.36,18.22,18
	7	75	26486	489(489,482,	13.60(13.60,13.41,13.41,13
	8	47	26421	40(40,37,37,3	1.11(1.11,1.03,1.03,1.06)
	9	75	28265	619(616,610,	17.22(17.14,16.97,16.88,16
	10	48	26443	138(138,137,	3.84(3.84,3.81,3.81,3.73)
	11	48	28184	12(12,0,0,0)	0.33(0.33,0.00,0.00,0.00)
S3-Day3	1	54	11373	41(37,0,0,0)	1.53(1.38,0.00,0.00,0.00)
	2	58	13668	214(214,0,0,(	7.97(7.97,0.00,0.00,0.00)
S1-Day4					
	1	46	26422	46(46,46,46,4	1.25(1.25,1.25,1.25,1.25)
	2	64	22501	363(362,2,2,6	9.89(9.86,0.05,0.05,0.16)
	3	47	26421	30(30,30,30,3	0.82(0.82,0.82,0.82,0.82)
	4	67	22503	428(428,0,0,2	11.66(11.66,0.00,0.00,0.05)
	5	69	13425	486(449,2,0,3	13.24(12.23,0.05,0.00,0.82)
S2-Day5	6	69	19112	7376(7374,73	200.92(200.87,200.16,200.1
	7	74	21058	1414(1413,14	38.52(38.49,38.24,38.22,37
	8	76	28267	21(21,19,19,8	0.57(0.57,0.52,0.52,0.22)
	9	48	26443	138(138,138,	3.76(3.76,3.76,3.76,3.70)
	10	48	28184	15(15,0,0,0)	0.41(0.41,0.00,0.00,0.00)
	11	49	19103	28(28,28,28,2	0.76(0.76,0.76,0.76,0.76)
S3-Day6	1	65	10133	380(380,379,	15.00(15.00,14.97,14.97,14
	2	69	13619	279(279,268,	11.02(11.02,10.58,10.58,10
	1	65	5807	2104(2104,20	69.76(69.76,69.53,69.53,68
	2	48	26443	16(16,16,16,1	0.53(0.53,0.53,0.53,0.53)
S2-Day8	3	70	2794	28(28,26,26,2	0.93(0.93,0.86,0.86,0.93)
	4	48	28184	20(20,0,0,2)	0.66(0.66,0.00,0.00,0.07)
	5	74	28998	28(2,0,0,0)	0.93(0.07,0.00,0.00,0.00)
S3-Day9					
S2-Day11	1	58	29871	38(2,0,0,0)	2.39(0.13,0.00,0.00,0.00)
	2	71	27762	90(90,90,90,8	5.67(5.67,5.67,5.67,5.42)
S3-Day12					
S3-Day18					

The numbers in the bracket are (reads with left primers, reads with right primers, reads with bot
Normalized count=(Read count/Total number of read mapped on reference genome)*1000000.
Total number of read mapped on reference genome is17428684, 35949645, 26863662, 2872584
“leader_end” and “TRS_start” refer to the position of the end of leader and the position of the s

:.25)
:.35)

;.47)

)
)
10,193.95)
'.97)

:.53)
).19)
:.57)

th primers, same junction on paired reads with at least a primer).

10, 36710848, 25324959, 30161120, 22185877, 15869342, 12758257, 18881995 and 15093231,
start of TRS identified in the reads >10.

excluding the mapped reads unpaired, not primary alignment and supplementary alignment.

	subgenome	leader_end	TRS_start	ACGAAC	ATG_postion	known_ATG
S1-Day1	1	63	5785	no	5859	-
	2	63	10978	no	11062	-
	3	46	26422	yes	26523	M
	4	65	10636	no	10662	-
	5	67	3362	no	3375	-
	6	67	22404	no	22407	-
	7	67	22503	no	22614	-
	8	68	4580	no	4590	-
	9	69	13619	no	13625	-
	10	47	26421	yes	26523	M
	11	71	10926	no	10938	-
	12	73	3372	no	3375	-
	13	74	21058	no	21071	-
	14	74	23112	no	23118	-
	15	48	26443	yes	26523	M
	16	48	28184	no	28207	-
S2-Day2	1	58	13666	no	13685	-
	2	58	26917	yes	27202	ORF6
	3	62	3808	no	3813	-
	4	63	3924	no	3948	-
	5	63	20583	no	20608	-
	6	63	23922	no	24051	-
	7	46	26422	yes	26523	M
	8	67	3362	no	3375	-
	9	67	22404	no	22407	-
	10	67	22490	no	22614	-
	11	67	26866	no	26896	-
	12	68	5789	no	5859	-
	13	68	14223	no	14258	-
	14	69	3816	no	3842	-
	15	69	11356	no	11367	-
	16	69	13619	no	13625	-
	17	69	19112	no	19148	-
	18	69	19571	no	19631	-
	19	69	21305	no	21317	-
	20	69	22382	no	22401	-
	21	47	26421	yes	26523	M
	22	71	5708	no	5718	-
	23	74	21058	no	21071	-
	24	74	25858	no	25910	-
	25	48	26443	yes	26523	M
	26	48	28184	no	28207	-

S3-Day3	1	48	26443	yes	26523	M
	2	53	17675	no	17681	-
S1-Day4	1	63	5785	no	5859	-
	2	68	5789	no	5859	-
	3	75	28265	no	28274	N
	4	48	28184	no	28207	-
S2-Day5	1	48	28184	no	28207	-
S3-Day6						
S2-Day8						
S3-Day9						
S2-Day11	1	51	27203	no	27256	-
	2	58	29871	no	29868	-
S3-Day12	1	76	28267	no	28274	N
	2	48	28184	no	28207	-
S3-Day14						

“peak_leader” and “peak_TRS_start” point to the leader-TRS junctions in sheet "novel", “ACGA

20_leader_se	TRS_seq	first_orf_aa
CGATCTCTTG	TTCTAAAGAA	MFSTKKTVTQQP*
CGATCTCTTG	AGTGAAAAG.	MVFVLFFV*
ACAAACCAA(	TCTCGTGTTA	MADSNGTITVEELKKLLEQWNLVIGFLFLTWICLLQFAYANRNRFLYIIKLIFLV
ATCTCTTGTA	TGGTACGGA(	MF*
CTCTTGTAGA	AAACTTACTG	MYTLKMQTLWKKLKR*
CTCTTGTAGA	AAA	MEPLQML*
CTCTTGTAGA	AAACTTCTAA	MLGTGRESATVLLIILSYIIPHHFPLLSVMECLLLN*
TCTTGTAGAT	AACGATCTAA	MKLLLQCHLAM*
CTTGTAGATC	ACGAAG	MTI*
CAAACCAAC(	CTCTCGTGTT.	MADSNGTITVEELKKLLEQWNLVIGFLFLTWICLLQFAYANRNRFLYIIKLIFLV
TGTAGATCTG	TTACACCTTT	MLLDNAQVLLSKVQ*
TAGATCTGTT	ACA	MYTLKMQTLWKKLKR*
AGATCTGTTC	CCTAAGACTA	MLQKKMTLKRVFSLTFVGLYNKS*
AGATCTGTTC	TTCTAC	MHQQLFVDLKSLLIWLKTNVSISTSMV*
AAACCAACCA	TCTTCTAGAG	MADSNGTITVEELKKLLEQWNLVIGFLFLTWICLLQFAYANRNRFLYIIKLIFLV
AAACCAACCA	TCTTGTAGTG	MKTF*
ACTTTCGATC	ACTTTCTCTA.	MKKQFIIYLRIVQLLLNMTSLSLE*
ACTTTCGATC	CGCTTCTAGA	MFHLVDFQVTIAEILLIIMRTFKVSIWNLDYIINLIIKNLSKSLTENKYSQLDEEC
TCGATCTCTT	TCTCT	MTNLFQAFWK*
CGATCTCTTG	CTTCAGTTGA	MIRKSKLVLKKLQQLWKKLSSSQKTCYFILTLMAIFIQILPLLLVTLTSLS*
CGATCTCTTG	TATTGACTAT	MLWCKDGHVETFYPKLQSSQAWQPGVAMPNLYKMQRMLLEKCDLQNY(
CGATCTCTTG	AAATTTACAA	MLASSNNMVIALVILLLETSFVHKSLTALLFCHLCSQMK*
ACAAACCAA(	TCTCGTGTTA	MADSNGTITVEELKKLLEQWNLVIGFLFLTWICLLQFAYANRNRFLYIIKLIFLV
CTCTTGTAGA	AAACTTACTG	MYTLKMQTLWKKLKR*
CTCTTGTAGA	AAA	MEPLQML*
CTCTTGTAGA	AAAGGAATCT	MLGTGRESATVLLIILSYIIPHHFPLLSVMECLLLN*
CTCTTGTAGA	AAACTAACAT	MALF*
TCTTGTAGAT	AAAGAAACTT	MFSTKKTVTQQP*
TCTTGTAGAT	AACAAAGCCT	MTSRKRG*
CTTGTAGATC	ACAAACTTGT	MKSEKQVEQKIAEIPKEEVKPFITESKPSVEQRKQDDKKIKACVEEVTTTLEET
CTTGTAGATC	AAGAACTGT(	MMMVLGECGHL*
CTTGTAGATC	ACGAAG	MTI*
CTTGTAGATC	ACAAAGCTTA	MPHILTNSQMVYAYFGIAMSIDILLIPLFVDLTLECYLTLTCLVVMVAVCM*
CTTGTAGATC	ACAAACAATT	MWLLML*
CTTGTAGATC	GCGAACAAAT	MVMSCMQITYFGGIQIQFSCLPILYLT*
CTTGTAGATC	ACTTTTCTAT	MKMEPLQML*
CAAACCAAC(	CTCTCGTGTT.	MADSNGTITVEELKKLLEQWNLVIGFLFLTWICLLQFAYANRNRFLYIIKLIFLV
TGTAGATCTG	GAACTTAAG(	MVHLLVLVSTLVITSVVTINI*
AGATCTGTTC	CCTAAGACTA	MLQKKMTLKRVFSLTFVGLYNKS*
AGATCTGTTC	TATTGTATAC	MAQQVLFLNMTTRLVVILKNGNLE*
AAACCAACCA	TCTTCTAGAG	MADSNGTITVEELKKLLEQWNLVIGFLFLTWICLLQFAYANRNRFLYIIKLIFLV
AAACCAACCA	TCTTGTAGTG	MKTF*

AAACCAACCA	TCTTCTAGAG	MADSNGTITVEELKKLLEQWNLVIGFLFLTWICLLQFAYANRNRFLYIIKLIFLV
AACCAACTTT	TCACGC	MMFHLQLTGHK*
CGATCTCTTG	TTCTAAAGAA	MFSTKKTVTQQP*
TCTTGTAGAT	AAAGAAACTT	MFSTKKTVTQQP*
GATCTGTTCT	CAAACTAAA	MSDNGPQNQRNAPRITFGGPSDSTGSNQNGERSGARSKQRRPQGLPNNT
AAACCAACCA	TCTTGTAGTG	MKTF*
AAACCAACCA	TCTTGTAGTG	MKTF*

CCAACCAACT	TGTTTCATCTC	MRTFKVSIWNLDYIINLIIKNLSKSLTENKYSQLDEEQPMEID*
ACTTTCGATC	-	
ATCTGTTCTCT	AACTAAA	MSDNGPQNQRNAPRITFGGPSDSTGSNQNGERSGARSKQRRPQGLPNNT
AAACCAACCA	TCTTGTAGTG	MKTF*

AC” indicates if there is a ACGAAC sequences in the “TRS_seq” (TRS sequences), “20_leader_sec

WLLWPVTLACFVLAAVYRINWITGGIAIAMACLVGLMWLSYFIASFRLFARTRSMWSFNPETNILLNVPLHGT

WLLWPVTLACFVLAAVYRINWITGGIAIAMACLVGLMWLSYFIASFRLFARTRSMWSFNPETNILLNVPLHGT

WLLWPVTLACFVLAAVYRINWITGGIAIAMACLVGLMWLSYFIASFRLFARTRSMWSFNPETNILLNVPLHGT

QPMEID*

GDSATLPKGIMMNVAKYTQLCQYLNTLTLAVPYNMRVIHFGAGSDKGVAPGTAVLRQWLPTGTLLVDSDLN

WLLWPVTLACFVLAAVYRINWITGGIAIAMACLVGLMWLSYFIASFRLFARTRSMWSFNPETNILLNVPLHGT

KFLTENLLLYIDINGNLHPDSATLVSDIDITFLKKDAPYIVGDVVQEGVLTAVVIPTKKAGGTTEMLAKALRKVP

WLLWPVTLACFVLAAVYRINWITGGIAIAMACLVGLMWLSYFIASFRLFARTRSMWSFNPETNILLNVPLHGT

WLLWPVTLACFVLAAVYRINWITGGIAIAMACLVGLMWLSYFIASFRLFARTRSMWSFNPETNILLNVPLHGT

WLLWPVTLACFVLAAVYRINWITGGIAIAMACLVGLMWLSYFIASFRLFARTRSMWSFNPETNILLNVPLHGT

ASWFTALTQHGKEDLKFPRGQGVPINTNSSPDDQIGYYRRATRRIRGGDGKMKDLSPRWYFYYLGTGPEAG

ASWFTALTQHGKEDLKFPRGQGVPINTNSSPDDQIGYYRRATRRIRGGDGKMKDLSPRWYFYYLGTGPEAG

q” refers to the 20 sequences before the end of the leader, “AUG_postion” and “first_orf_aa” re

ILTRPLLESELVIGAVILRGHLRIAGHHLGRCDIKDLPKEITVATSRTLSYYKLGASQRVAGDSGFAAYSRYRIGNY

ILTRPLLESELVIGAVILRGHLRIAGHHLGRCDIKDLPKEITVATSRTLSYYKLGASQRVAGDSGFAAYSRYRIGNY

ILTRPLLESELVIGAVILRGHLRIAGHHLGRCDIKDLPKEITVATSRTLSYYKLGASQRVAGDSGFAAYSRYRIGNY

DFVSDADSTLIGDCATVHTANKWDLIISDMYDPKTKNVTKENDSKEGFFTYICGFIQQKLALGGSVAIKITEHSV

ILTRPLLESELVIGAVILRGHLRIAGHHLGRCDIKDLPKEITVATSRTLSYYKLGASQRVAGDSGFAAYSRYRIGNY

TDNYITTYPGQGLNGYTVEEAKTVLKKCKSAFYILPSIISNEKQEILGTVSWNLREMLAHAEETRKLMPVCVETK

ILTRPLLESELVIGAVILRGHLRIAGHHLGRCDIKDLPKEITVATSRTLSYYKLGASQRVAGDSGFAAYSRYRIGNY

ILTRPLLESELVIGAVILRGHLRIAGHHLGRCDIKDLPKEITVATSRTLSYYKLGASQRVAGDSGFAAYSRYRIGNY

ILTRPLLESELVIGAVILRGHLRIAGHHLGRCDIKDLPKEITVATSRTLSYYKLGASQRVAGDSGFAAYSRYRIGNY

LPYGANKDGIIWVATEGALNTPKDHIGTRNPANNAAIVLQLPQGTTLPKGFYAEGSRGGSQASSRSSSRSRN

LPYGANKDGIIWVATEGALNTPKDHIGTRNPANNAAIVLQLPQGTTLPKGFYAEGSRGGSQASSRSSSRSRN

efer to the first AUG position and translated orf of the sgmRNA, and “known_ATG” indicates if th

KLNTDHSSSSDNIALLVQ*

KLNTDHSSSSDNIALLVQ*

KLNTDHSSSSDNIALLVQ*

WNADLYKLMGHFAWWTAFVTNVNASSSEAFLIGCNYLGKPREQIDGYVMHANYIFWRNTNPIQLSSYSLFD

KLNTDHSSSSDNIALLVQ*

AIVSTIQRKYKGIKIQEGVVDYGARFYFYTSKTTVASLINTLNDLNETLVTMPLGYVTHGLNLEEAARYMRSLK

KLNTDHSSSSDNIALLVQ*

KLNTDHSSSSDNIALLVQ*

KLNTDHSSSSDNIALLVQ*

SSRNSTPGSSRGTSPARMAGNGGDAALALLLLDRLNQLESKMSGKGQQQQGQTVTKKSAAEASKKPRQKRT

SSRNSTPGSSRGTSPARMAGNGGDAALALLLLDRLNQLESKMSGKGQQQQGQTVTKKSAAEASKKPRQKRT

e first AUG position is the same as a known sgmRNA.

[illegible]MSKFPLKLRGTAVMSLKEGQINDMILSLLSKGRLIIRENNRVVISSDVLVNN*

[illegible]PATVSVSSPDAVTAYNGYLTSSSKTPEEHFIETISLAGSYKDWSYSGQSTQLGIEFLKRGDKSVYYTSNPTTFHL

TATKAYNVTQAFGRRGPEQTQGNFGDQELIRQGTDYKHWPQIAQFAPSASAFFGMSRIGMEVTPSGTWLT

TATKAYNVTQAFGRRGPEQTQGNFGDQELIRQGTDYKHWPQIAQFAPSASAFFGMSRIGMEVTPSGTWLT

DGEVITFDNLKTLLSLREVRTIKVFTTVDNINLHTQVVDMSMTYGQQFGPTYLDGADVTKIKPHNSHEGKTFY

YTGAIKLDDKDPNFKDQVILLNKHIDAYKTFPPTEPKKDKKKKADETQALPQRQKKQQTVTLLPAADLDDFSK[illegible]

YTGAIKLDDKDPNFKDQVILLNKHIDAYKTFPPTEPKKDKKKKADETQALPQRQKKQQTVTLLPAADLDDFSK[illegible]

VLPNDDTLRVEAFEYYHTTDPSFLGRYMSALNHTKKWKYPQVNGLTSIKWADNNCYLATALLTLQQIELKFN

QLQQSMSSADSTQA*

QLQQSMSSADSTQA*

PPALQDAYYRARAGEAANFCALILAYCNKTVGELGDVRETMSYLFQHANLDSCKRVLNVVCKTCGQQQTTLK

[illegible]GVEAVMYMGTLSYEQFKKGVQIPCTCGKQATKYLVQQESPFVMMSAPPAQYELKHGTFTCASEYTGNYQC

GHYKHITSKETLYCIDGALLTKSSEYKGPITDVFYKENSYTTTIKPVTYKLDGVVCTEIDPKLDNYYKKDNSYFTEC

QPIDLVPNQPYPNASFDNFKFVCDNIKFADDLNQLTGYKKPASRELKVTFFPDLNGDVVAIDYKHYTPSFKKGA

[illegible]KLLHKPIVWHVNNATNKATYKPNTWCIRCLWSTKPVETSNSFDVLKSEDAQGMDNLACEDLKPVSEEVVEN[illegible]

IPTIQKDVLECNVKTTEVVGDIILKPANNSLKITEEVGHTDLMAAYVDNSSLTIKKPNELSRVLGLKTLATHGLAA

VNSVPWDTIANYAKPFLNKVVSTTTNIVTRCLNRVCTNYMPYFFTLLLQLCTFTRSTNSRIKASMPTTIAKNT

'KSVGKFCLEASFN

	subgenome	leader_end	TRS_start	ACGAAC	ATG_postion	known_ATG
S1-Day1						
	1	46	26422	yes	26523	M
	2	66	13865	no	13898	-
	3	68	2689	no	2745	-
	4	70	14372	no	14381	-
	5	71	27762	no	27825	-
S2-Day2	6	74	21058	no	21071	-
	7	75	26486	no	26523	M
	8	47	26421	yes	26523	M
	9	75	28265	no	28274	N
	10	48	26443	yes	26523	M
	11	48	28184	no	28207	-
S3-Day3	1	54	11373	no	11373	-
	2	58	13668	no	13685	-
S1-Day4						
	1	46	26422	yes	26523	M
	2	64	22501	no	22614	-
	3	47	26421	yes	26523	M
	4	67	22503	no	22614	-
	5	69	13425	no	13433	-
S2-Day5	6	69	19112	no	19148	-
	7	74	21058	no	21071	-
	8	76	28267	no	28274	N
	9	48	26443	yes	26523	M
	10	48	28184	no	28207	-
	11	49	19103	no	19148	-
S3-Day6	1	65	10133	no	10152	-
	2	69	13619	no	13625	-
	1	65	5807	no	5859	-
	2	48	26443	yes	26523	M
S2-Day8	3	70	2794	no	2802	-
	4	48	28184	no	28207	-
	5	74	28998	no	29079	-
S3-Day9						
S2-Day11	1	58	29871	no	29868	-
	2	71	27762	no	27825	-
S3-Day12						
S3-Day18						

“peak_leader” and “peak_TRS_start” point to the leader-TRS junctions in sheet "novel", “ACGAA

20_leader_se	TRS_seq	first_orf_aa
ACAAACCAAC	TCTCGTGTTA	MADSNGTITVEELKKLLEQWNLVIGFLFLTWICLLQFAYANRNRFLYIIKLIFLW
TCTCTTGTAG	TAAAAGAAAT	MMIISIKRTGMIL*
TCTTGTAGAT	AACAAACAAT	MTL*
TTGTAGATCT	CAAACTTTA	MFYSLQCSHLQVLDH*
TGTAGATCTG	GAACTTTCAT	MLIIFWFSLELQDHNETCHA*
AGATCTGTTC	CCTAAGACTA	MLQKKMTLKRVFSLTFVGLYNKS*
GATCTGTTCT	TATATTAGTT	MADSNGTITVEELKKLLEQWNLVIGFLFLTWICLLQFAYANRNRFLYIIKLIFLW
CAAACCAACC	CTCTCGTGTT	MADSNGTITVEELKKLLEQWNLVIGFLFLTWICLLQFAYANRNRFLYIIKLIFLW
GATCTGTTCT	CAAACTAAA	MSDNGPQNQRNAPRITFGGPSDSTGSNQNGERSGARSKQRRPQGLPNNTA
AAACCAACCA	TCTTCTAGAG	MADSNGTITVEELKKLLEQWNLVIGFLFLTWICLLQFAYANRNRFLYIIKLIFLW
AAACCAACCA	TCTTGTAGTG	MKTF*
ACCAACTTTC	-	MVLGECGHL*
ACTTTCGATC	TTTCTCTAACT	MKKQFIIYLRIVQLLLNMTSLSLE*
ACAAACCAAC	TCTCGTGTTA	MADSNGTITVEELKKLLEQWNLVIGFLFLTWICLLQFAYANRNRFLYIIKLIFLW
GATCTCTTGT	TCAAACTTCT	MLGTGRESATVLLIILSYIIPHHFPLLSVMECLLLN*
CAAACCAACC	CTCTCGTGTT	MADSNGTITVEELKKLLEQWNLVIGFLFLTWICLLQFAYANRNRFLYIIKLIFLW
CTCTTGTAGA	AAACTTCTAA	MLGTGRESATVLLIILSYIIPHHFPLLSVMECLLLN*
CTTGTAGATC	GCGAACCC	MLQSADAQSFLNGFAV*
CTTGTAGATC	ACAAAGCTTA	MPHILTNSQMVYAYFGIAMSIDILLIPLFVDLTLECYLTLTCLVVMVAVCM*
AGATCTGTTC	CCTAAGACTA	MLQKKMTLKRVFSLTFVGLYNKS*
ATCTGTTCTC	AACTAAA	MSDNGPQNQRNAPRITFGGPSDSTGSNQNGERSGARSKQRRPQGLPNNTA
AAACCAACCA	TCTTCTAGAG	MADSNGTITVEELKKLLEQWNLVIGFLFLTWICLLQFAYANRNRFLYIIKLIFLW
AAACCAACCA	TCTTGTAGTG	MKTF*
AACCAACCAA	CTTGTAGTGA	MPHILTNSQMVYAYFGIAMSIDILLIPLFVDLTLECYLTLTCLVVMVAVCM*
ATCTCTTGTA	CTTAACGGTC	MT*
CTTGTAGATC	ACGAAG	MTI*
ATCTCTTGTA	ATAGACGGTC	MFSTKKTVTQQP*
AAACCAACCA	TCTTCTAGAG	MADSNGTITVEELKKLLEQWNLVIGFLFLTWICLLQFAYANRNRFLYIIKLIFLW
TTGTAGATCT	TGAACTTG	MKGLIKYLMRSALPIQLNSVQK*
AAACCAACCA	TCTTGTAGTG	MKTF*
AGATCTGTTC	AAGGCCAAAC	M*
ACTTTCGATC	-	
TGTAGATCTG	GAACTTTCAT	MLIIFWFSLELQDHNETCHA*

C” indicates if there is a ACGAAC sequences in the “TRS_seq” (TRS sequences), “20_leader_seq”

/LLWPVTLACFVLAAVYRINWITGGIAIAMACLVGLMWLSYFIASFRLFARTRSMWSFNPETNILLNVPLHGTIL

/LLWPVTLACFVLAAVYRINWITGGIAIAMACLVGLMWLSYFIASFRLFARTRSMWSFNPETNILLNVPLHGTIL
/LLWPVTLACFVLAAVYRINWITGGIAIAMACLVGLMWLSYFIASFRLFARTRSMWSFNPETNILLNVPLHGTIL
ASWFTALTQHGKEDLKFPRGQGVPINTNSSPDDQIGYYRRATRRIRGGDGKMKDLSPRWYFYYLGTGPEAGL
/LLWPVTLACFVLAAVYRINWITGGIAIAMACLVGLMWLSYFIASFRLFARTRSMWSFNPETNILLNVPLHGTIL

/LLWPVTLACFVLAAVYRINWITGGIAIAMACLVGLMWLSYFIASFRLFARTRSMWSFNPETNILLNVPLHGTIL

/LLWPVTLACFVLAAVYRINWITGGIAIAMACLVGLMWLSYFIASFRLFARTRSMWSFNPETNILLNVPLHGTIL

ASWFTALTQHGKEDLKFPRGQGVPINTNSSPDDQIGYYRRATRRIRGGDGKMKDLSPRWYFYYLGTGPEAGL
/LLWPVTLACFVLAAVYRINWITGGIAIAMACLVGLMWLSYFIASFRLFARTRSMWSFNPETNILLNVPLHGTIL

/LLWPVTLACFVLAAVYRINWITGGIAIAMACLVGLMWLSYFIASFRLFARTRSMWSFNPETNILLNVPLHGTIL

refers to the 20 sequences before the end of the leader, “AUG_postion” and “first_orf_aa” refer

TRPLLESELVIGAVILRGHLRIAGHHLGRCDIKDLPKEITVATSRTLSYYKLGASQRVAGDSGFAAYSRYRIGNYKL

TRPLLESELVIGAVILRGHLRIAGHHLGRCDIKDLPKEITVATSRTLSYYKLGASQRVAGDSGFAAYSRYRIGNYKL
TRPLLESELVIGAVILRGHLRIAGHHLGRCDIKDLPKEITVATSRTLSYYKLGASQRVAGDSGFAAYSRYRIGNYKL
YGANKDGIIWVATEGALNTPKDHIGTRNPANNAAIVLQLPQGTTLPKGFYAEGSRGGSQASSRSSSRSRNSS
TRPLLESELVIGAVILRGHLRIAGHHLGRCDIKDLPKEITVATSRTLSYYKLGASQRVAGDSGFAAYSRYRIGNYKL

TRPLLESELVIGAVILRGHLRIAGHHLGRCDIKDLPKEITVATSRTLSYYKLGASQRVAGDSGFAAYSRYRIGNYKL

TRPLLESELVIGAVILRGHLRIAGHHLGRCDIKDLPKEITVATSRTLSYYKLGASQRVAGDSGFAAYSRYRIGNYKL

YGANKDGIIWVATEGALNTPKDHIGTRNPANNAAIVLQLPQGTTLPKGFYAEGSRGGSQASSRSSSRSRNSS
TRPLLESELVIGAVILRGHLRIAGHHLGRCDIKDLPKEITVATSRTLSYYKLGASQRVAGDSGFAAYSRYRIGNYKL

TRPLLESELVIGAVILRGHLRIAGHHLGRCDIKDLPKEITVATSRTLSYYKLGASQRVAGDSGFAAYSRYRIGNYKL

to the first AUG position and translated orf of the sgmRNA, and “known_ATG” indicates if the fi

NTDHSSSSDNIALLVQ*

NTDHSSSSDNIALLVQ*
NTDHSSSSDNIALLVQ*
RNSTPGSSRGTSPARMAGNGGDAALALLLLDRLNQLESKMSGKGQQQQGQTVTKKSAAEASKKPRQKRTATK
NTDHSSSSDNIALLVQ*

NTDHSSSSDNIALLVQ*

NTDHSSSSDNIALLVQ*

RNSTPGSSRGTSPARMAGNGGDAALALLLLDRLNQLESKMSGKGQQQQGQTVTKKSAAEASKKPRQKRTATK
NTDHSSSSDNIALLVQ*

NTDHSSSSDNIALLVQ*

rst AUG position is the same as a known sgmRNA.

KAYNVTQAFGRRGPEQTQGNFGDQELIRQGTDYKHWPQIAQFAPSASAFFGMSRIGMEVTPSGTWLTYTGA

KAYNVTQAFGRRGPEQTQGNFGDQELIRQGTDYKHWPQIAQFAPSASAFFGMSRIGMEVTPSGTWLTYTGA

IKLDDKDPNFKDQVILLNKHIDAYKTFPPTEPKKDKKKKADETQALPQRQKKQQTVTLLPAADLDDFSKQLQQ

IKLDDKDPNFKDQVILLNKHIDAYKTFPPTEPKKDKKKKADETQALPQRQKKQQTVTLLPAADLDDFSKQLQQ

SMSSADSTQA*

SMSSADSTQA*

Supplemental tables

Click here to access/download;Table;Supplemental_Material_5.xlsx

	subgenome	ref_leader_er	peak_leader_	ref_TRS_start	peak_TRS_sta	peak_count
	S	65	0	21552	0	0
	ORF3a	69	0	25385	0	0
	E	69	0	26237	0	0
	M	65	0	26469	0	0
Patient1-day1	ORF6	69	0	27041	0	0
	ORF7a	69	0	27388	0	0
	ORF7b	65	0	27644	0	0
	ORF8	65	0	27884	0	0
	N	65	65	28256	28256	453(447,423,
	ORF10	65	0	29530	0	0
	S	65	65	21552	21552	116(113,60,5
	ORF3a	69	69	25385	25385	87(86,12,11,(
	E	69	0	26237	0	0
	M	65	65	26469	26469	565(557,93,9
Patient2-day1	ORF6	69	69	27041	27041	545(527,528,
	ORF7a	69	69	27388	27388	5(5,1,1,0,0)
	ORF7b	65	0	27644	0	0
	ORF8	65	65	27884	27884	1(1,0,0,0,0)
	N	65	65	28256	28256	5039(4895,45
	ORF10	65	0	29530	0	0
	S	65	0	21552	0	0
	ORF3a	69	0	25385	0	0
	E	69	69	26237	26237	152(143,34,3
	M	65	65	26469	26469	266(262,41,4
Patient3-day1	ORF6	69	69	27041	27041	347(339,325,
	ORF7a	69	0	27388	0	0
	ORF7b	65	0	27644	0	0
	ORF8	65	0	27884	0	0
	N	65	65	28256	28256	4666(4535,43
	ORF10	65	0	29530	0	0
	S	65	0	21552	0	0
	ORF3a	69	0	25385	0	0
	E	69	0	26237	0	0
	M	65	0	26469	0	0
Patient4-day1	ORF6	69	69	27041	27041	550(532,531,
	ORF7a	69	0	27388	0	0
	ORF7b	65	0	27644	0	0
	ORF8	65	0	27884	0	0
	N	65	65	28256	28256	7(7,6,6,0,0)
	ORF10	65	0	29530	0	0
	S	65	0	21552	0	0
	ORF3a	69	0	25385	0	0

	E	69	69	26237	26237	1(1,1,1,0,0)
	M	65	0	26469	0	0
Patient5-day1	ORF6	69	0	27041	0	0
	ORF7a	69	0	27388	0	0
	ORF7b	65	0	27644	0	0
	ORF8	65	0	27884	0	0
	N	65	65	28256	28256	3635(3513,34
	ORF10	65	0	29530	0	0
	S	65	65	21552	21552	383(353,175,
	ORF3a	69	69	25385	25385	59(57,3,3,0,0
	E	69	69	26237	26237	210(188,33,3
	M	65	65	26469	26469	1195(1107,21
Patient6-day1	ORF6	69	69	27041	27041	770(708,728,
	ORF7a	69	69	27388	27388	3(3,0,0,0,0)
	ORF7b	65	0	27644	0	0
	ORF8	65	65	27884	27884	1(1,1,1,0,0)
	N	65	65	28256	28256	7004(6449,65
	ORF10	65	0	29530	0	0
	S	65	0	21552	0	0
	ORF3a	69	0	25385	0	0
	E	69	0	26237	0	0
	M	65	65	26469	26469	510(498,109,
Patient6-day3	ORF6	69	69	27041	27041	182(178,165,
	ORF7a	69	0	27388	0	0
	ORF7b	65	0	27644	0	0
	ORF8	65	0	27884	0	0
	N	65	65	28256	28256	4(4,4,4,0,0)
	ORF10	65	0	29530	0	0
	S	65	0	21552	0	0
	ORF3a	69	0	25385	0	0
	E	69	0	26237	0	0
	M	65	65	26469	26469	2(2,0,0,0,0)
Patient6-day9	ORF6	69	0	27041	0	0
	ORF7a	69	0	27388	0	0
	ORF7b	65	0	27644	0	0
	ORF8	65	0	27884	0	0
	N	65	65	28256	28256	13(13,11,11,(
	ORF10	65	0	29530	0	0
	S	65	65	21552	21552	737(710,333,
	ORF3a	69	65	25385	25381	50(48,3,3,0,0
	E	69	69	26237	26237	425(407,82,8
	M	65	65	26469	26469	421(402,74,7
Patient7-day1	ORF6	69	69	27041	27041	2(1,1,0,0,0)

Patient7-day1	ORF7a	69	69	27388	27388	1(1,0,0,0,0)
	ORF7b	65	0	27644	0	0
	ORF8	65	0	27884	0	0
	N	65	65	28256	28256	7360(7093,68
	ORF10	65	0	29530	0	0
	S	65	0	21552	0	0
	ORF3a	69	0	25385	0	0
	E	69	0	26237	0	0
	M	65	0	26469	0	0
Patient7-day3	ORF6	69	69	27041	27041	0(0,0,0,0,0)
	ORF7a	69	0	27388	0	0
	ORF7b	65	0	27644	0	0
	ORF8	65	0	27884	0	0
	N	65	65	28256	28256	4395(4275,41
	ORF10	65	0	29530	0	0
	S	65	0	21552	0	0
	ORF3a	69	0	25385	0	0
	E	69	69	26237	26237	1(1,0,0,0,0)
	M	65	0	26469	0	0
Patient7-day5	ORF6	69	0	27041	0	0
	ORF7a	69	0	27388	0	0
	ORF7b	65	0	27644	0	0
	ORF8	65	0	27884	0	0
	N	65	65	28256	28256	2612(2531,24
	ORF10	65	0	29530	0	0
	S	65	0	21552	0	0
	ORF3a	69	0	25385	0	0
	E	69	0	26237	0	0
	M	65	65	26469	26469	2(1,0,0,0,0)
Patient7-day7	ORF6	69	0	27041	0	0
	ORF7a	69	0	27388	0	0
	ORF7b	65	0	27644	0	0
	ORF8	65	0	27884	0	0
	N	65	65	28256	28256	4468(4337,42
	ORF10	65	0	29530	0	0
	S	65	0	21552	0	0
	ORF3a	69	0	25385	0	0
	E	69	0	26237	0	0
	M	65	65	26469	26469	181(179,25,2
Patient8-day1	ORF6	69	0	27041	0	0
	ORF7a	69	0	27388	0	0
	ORF7b	65	0	27644	0	0
	ORF8	65	0	27884	0	0

	N	65	65	28256	28256	2(2,2,2,0,0)
	ORF10	65	0	29530	0	0
Patient8-day3	S	65	65	21552	21552	1(1,0,0,0,0)
	ORF3a	69	0	25385	0	0
	E	69	0	26237	0	0
	M	65	65	26469	26469	1(1,0,0,0,0)
	ORF6	69	69	27041	27041	1(0,1,0,0,0)
	ORF7a	69	0	27388	0	0
	ORF7b	65	0	27644	0	0
	ORF8	65	0	27884	0	0
	N	65	65	28256	28256	2(2,2,2,0,0)
	ORF10	65	0	29530	0	0
Patient8-day9	S	65	65	21552	21552	322(312,169,
	ORF3a	69	0	25385	0	0
	E	69	0	26237	0	0
	M	65	0	26469	0	0
	ORF6	69	0	27041	0	0
	ORF7a	69	0	27388	0	0
	ORF7b	65	0	27644	0	0
	ORF8	65	0	27884	0	0
	N	65	65	28256	28256	4373(4213,41
	ORF10	65	0	29530	0	0

The numbers in the bracket are (reads with left primers, reads with right primers, reads with both pri
Normalized count=(Read count/Total number of read mapped on reference genome)*1000000.
Total number of read mapped on reference genome is 9639, 1095598, 872890, 44932, 153792, 1321
“ref_leader_end” and “peak_leader_end” point to the reference position of the end of leader and th

peak_normal	cluster_count	cluster_normalized_count
0	0	0
0	0	0
0	0	0
0	0	0
0	0	0
0	0	0
0	0	0
0	0	0
46996.58(463	460(454,429,	47722.79(47100.32,44506.69,43884.22,0.00,0.00)
0	0	0
105.88(103.1	116(113,60,5	105.88(103.14,54.76,52.94,0.00,0.00)
79.41(78.50,1	118(117,14,1	107.70(106.79,12.78,11.87,0.00,0.00)
0	0	0
515.70(508.4	581(572,98,9	530.30(522.09,89.45,87.62,0.00,0.00)
497.45(481.0	553(535,535,	504.75(488.32,488.32,471.89,0.00,0.00)
4.56(4.56,0.9	5(5,1,1,0,0)	4.56(4.56,0.91,0.91,0.00,0.00)
0	0	0
0.91(0.91,0.0	1(1,0,0,0,0)	0.91(0.91,0.00,0.00,0.00,0.00)
4599.31(4467	5167(5021,47	4716.15(4582.89,4299.93,4180.37,0.00,0.00)
0	0	0
0	0	0
0	0	0
174.13(163.8	154(145,35,3	176.43(166.11,40.10,36.66,0.00,0.00)
304.73(300.1	268(264,41,4	307.03(302.44,46.97,45.82,0.00,0.00)
397.53(388.3	352(344,329,	403.26(394.09,376.91,367.74,0.00,0.00)
0	0	0
0	0	0
0	0	0
5345.46(5195	4772(4639,44	5466.90(5314.53,5072.80,4934.18,0.00,0.00)
0	0	0
0	0	0
0	0	0
0	0	0
0	0	0
12240.72(118	556(538,537,	12374.25(11973.65,11951.39,11573.04,0.00,0.00)
0	0	0
0	0	0
0	0	0
155.79(155.7	8(8,7,7,0,0)	178.05(178.05,155.79,155.79,0.00,0.00)
0	0	0
0	0	0
0	0	0

6.50(6.50,6.5	1(1,1,1,0,0)	6.50(6.50,6.50,6.50,0.00,0.00)
0	0	0
0	0	0
0	0	0
0	0	0
0	0	0
23635.82(228	3722(3600,35	24201.52(23408.24,22966.08,22218.32,0.00,0.00)
0	0	0
289.84(267.1	384(354,175,	290.59(267.89,132.43,121.84,0.00,0.00)
44.65(43.14,2	62(60,3,3,0,0	46.92(45.41,2.27,2.27,0.00,0.00)
158.92(142.2	212(190,34,3	160.43(143.78,25.73,24.97,0.00,0.00)
904.32(837.7	1226(1136,22	927.78(859.68,167.24,154.38,0.00,0.00)
582.70(535.7	785(719,741,	594.05(544.11,560.76,512.32,0.00,0.00)
2.27(2.27,0.0	3(3,0,0,0,0)	2.27(2.27,0.00,0.00,0.00,0.00)
0	0	0
0.76(0.76,0.7	1(1,1,1,0,0)	0.76(0.76,0.76,0.76,0.00,0.00)
5300.32(488(	7174(6615,66	5428.97(5005.94,5040.00,4648.00,0.00,0.00)
0	0	0
0	0	0
0	0	0
0	0	0
5354.61(5228	526(514,113,	5522.60(5396.61,1186.41,1154.92,0.00,0.00)
1910.86(1868	184(180,167,	1931.86(1889.86,1753.37,1711.38,0.00,0.00)
0	0	0
0	0	0
0	0	0
42.00(42.00,4	4(4,4,4,0,0)	42.00(42.00,42.00,42.00,0.00,0.00)
0	0	0
0	0	0
0	0	0
0	0	0
30.55(30.55,(	2(2,0,0,0,0)	30.55(30.55,0.00,0.00,0.00,0.00)
0	0	0
0	0	0
0	0	0
0	0	0
198.60(198.6	13(13,11,11,(	198.60(198.60,168.04,168.04,0.00,0.00)
0	0	0
517.78(498.8	740(712,335,	519.88(500.21,235.35,228.33,0.00,0.00)
35.13(33.72,2	64(62,4,4,0,0	44.96(43.56,2.81,2.81,0.00,0.00)
298.58(285.9	434(416,84,8	304.90(292.26,59.01,58.31,0.00,0.00)
295.77(282.4	431(412,76,7	302.80(289.45,53.39,51.29,0.00,0.00)
1.41(0.70,0.7	2(1,1,0,0,0)	1.41(0.70,0.70,0.00,0.00,0.00)

0.70(0.70,0.0	2(2,1,1,0,0)	1.41(1.41,0.70,0.70,0.00,0.00)
0	0	0
0	0	0
5170.74(498[illegible]	7574(7294,70[illegible]	5321.08(5124.37,4955.05,4768.18,0.00,0.00)
0	0	0
0	0	0
0	0	0
0	0	0
0	0	0
0.00(0.00,0.0	0(0,0,0,0,0)	0.00(0.00,0.00,0.00,0.00,0.00)
0	0	0
0	0	0
0	0	0
102418.90(9[illegible]	4500(4374,4[illegible]	104865.77(101929.53,98457.31,95777.40,0.00,0.00)
0	0	0
0	0	0
0	0	0
49.89(49.89,[illegible]	1(1,0,0,0,0)	49.89(49.89,0.00,0.00,0.00,0.00)
0	0	0
0	0	0
0	0	0
0	0	0
0	0	0
130300.31(1[illegible]	2685(2603,2[illegible]	133941.93(129851.34,127357.08,123466.03,0.00,0.00)
0	0	0
0	0	0
0	0	0
0	0	0
26.36(13.18,[illegible]	2(1,0,0,0,0)	26.36(13.18,0.00,0.00,0.00,0.00)
0	0	0
0	0	0
0	0	0
0	0	0
58878.57(57[illegible]	4579(4445,4[illegible]	60341.31(58575.48,57270.87,55597.29,0.00,0.00)
0	0	0
0	0	0
0	0	0
0	0	0
1514.25(149[illegible]	186(184,27,2	1556.08(1539.35,225.88,217.52,0.00,0.00)
0	0	0
0	0	0
0	0	0
0	0	0

16.73(16.73,1	2(2,2,2,0,0)	16.73(16.73,16.73,16.73,0.00,0.00)
0	0	0
13.76(13.76,(	1(1,0,0,0,0)	13.76(13.76,0.00,0.00,0.00,0.00)
0	0	0
0	0	0
13.76(13.76,(	1(1,0,0,0,0)	13.76(13.76,0.00,0.00,0.00,0.00)
13.76(0.00,13	1(0,1,0,0,0)	13.76(0.00,13.76,0.00,0.00,0.00)
0	0	0
0	0	0
0	0	0
27.52(27.52,2	2(2,2,2,0,0)	27.52(27.52,27.52,27.52,0.00,0.00)
0	0	0
2928.47(2837	322(312,169,	2928.47(2837.52,1536.99,1518.80,0.00,0.00)
0	0	0
0	0	0
0	0	0
0	0	0
0	0	0
0	0	0
0	0	0
39770.82(383	4472(4309,42	40671.18(39188.76,38952.30,37533.54,0.00,0.00)
0	0	0

mers, same junction on paired reads with at least a primer).

429, 95245, 65459, 1423395, 42912, 20046, 75885, 119531, 72674 and 109955 excluding the ma

e position of the end of leader identified in the most common reads (peak count) on the referen

ıpped reads unpaired, not primary alignment and supplementary alignment.
ce genome, and “ref_TRS_start” and “peak_TRS_start” refer to the reference position of the star

t of TRS and the position of the start of TRS identified in the most common reads (peak count) or

n the reference genome.

	subgenome	peak_leader_	peak_TRS_sta	ACGAAC	ATG_postion	20_leader_se
Patient1-day1	N	65	28256	yes	28274	ATCTCTTGTA(
Patient2-day1	S	65	21552	yes	21563	ATCTCTTGTA(
	ORF3a	69	25385	yes	25393	CTTGTAGATC
	M	65	26469	yes	26523	ATCTCTTGTA(
	ORF6	69	27041	yes	27202	CTTGTAGATC
	ORF7a	69	27388	yes	27394	CTTGTAGATC
	ORF8	65	27884	yes	27894	ATCTCTTGTA(
	N	65	28256	yes	28274	ATCTCTTGTA(
Patient3-day1	E	69	26237	yes	26245	CTTGTAGATC
	M	65	26469	yes	26523	ATCTCTTGTA(
	ORF6	69	27041	yes	27202	CTTGTAGATC
	N	65	28256	yes	28274	ATCTCTTGTA(
Patient4-day1	ORF6	69	27041	yes	27202	CTTGTAGATC
	N	65	28256	yes	28274	ATCTCTTGTA(
Patient5-day1	E	69	26237	yes	26245	CTTGTAGATC
	N	65	28256	yes	28274	ATCTCTTGTA(
Patient6-day1	S	65	21552	yes	21563	ATCTCTTGTA(
	ORF3a	69	25385	yes	25393	CTTGTAGATC
	E	69	26237	yes	26245	CTTGTAGATC
	M	65	26469	yes	26523	ATCTCTTGTA(
	ORF6	69	27041	yes	27202	CTTGTAGATC
	ORF7a	69	27388	yes	27394	CTTGTAGATC
	ORF8	65	27884	yes	27894	ATCTCTTGTA(
	N	65	28256	yes	28274	ATCTCTTGTA(
Patient6-day3	M	65	26469	yes	26523	ATCTCTTGTA(
	ORF6	69	27041	yes	27202	CTTGTAGATC
	N	65	28256	yes	28274	ATCTCTTGTA(
Patient6-day9	M	65	26469	yes	26523	ATCTCTTGTA(
	N	65	28256	yes	28274	ATCTCTTGTA(
Patient7-day1	S	65	21552	yes	21563	ATCTCTTGTA(
	ORF3a	65	25381	yes	25393	ATCTCTTGTA(
	E	69	26237	yes	26245	CTTGTAGATC
	M	65	26469	yes	26523	ATCTCTTGTA(
	ORF6	69	27041	yes	27202	CTTGTAGATC
	ORF7a	69	27388	yes	27394	CTTGTAGATC
	N	65	28256	yes	28274	ATCTCTTGTA(
Patient7-day3	ORF6	69	27041	yes	27202	CTTGTAGATC
	N	65	28256	yes	28274	ATCTCTTGTA(
Patient7-day5	E	69	26237	yes	26245	CTTGTAGATC
	N	65	28256	yes	28274	ATCTCTTGTA(
Patient7-day7	M	65	26469	yes	26523	ATCTCTTGTA(
	N	65	28256	yes	28274	ATCTCTTGTA(

Patient8-day1	M	65	26469	yes	26523	ATCTCTTGTA(
	N	65	28256	yes	28274	ATCTCTTGTA(
	S	65	21552	yes	21563	ATCTCTTGTA(
Patient8-day3	M	65	26469	yes	26523	ATCTCTTGTA(
	ORF6	69	27041	yes	27202	CTTGTAGATC
	N	65	28256	yes	28274	ATCTCTTGTA(
Patient8-day9	S	65	21552	yes	21563	ATCTCTTGTA(
	N	65	28256	yes	28274	ATCTCTTGTA(

“peak_leader” and “peak_TRS_start” point to the leader-TRS junctions in sheet "known", “ACGAAC”

TRS_seq	first_orf_aa
CTAAACGAA	MSDNGPQNQRNAPRITFGGPSDSTGSNQNGERSGARSKQRRPQGLPNNTASWFTALTQH
CTAAACGAA	MFVFLVLLPLVSSQCVNLTTRTQLPPAYTNSFTRGVYYPDKVFRSSVLHSTQDLFLPFFSNVTW
ACGAACTT	MDLFMRIFTIGTVTLKQGEIKDATPSDFVRATATIPIQASLPFGWLIVGVALLAVFQSASKIITLK
CTAAACGAA	MADSNGTITVEELKKLLEQWNLVIGFLFLTWICLLQFAYANRNRFLYIIKLIFLWLLWPVTLACF
ACGAACGCT	MFHLVDFQVTIAEILLIIMRTFKVSIWNLDYIINLIIKNLSKSLTENKYSQLDEEQPMEID*
ACGAAC	MKIILFLALITLATCELYHYQECVRGTTVLLKEPCSSGTYEGNSPFHPLADNKFALTCFSTQFAFA
CTAAACGAA	MKFLVFLGIITTVAAFHQECSLQSCTQHQPYVVDDPCPIHFYSKWYIRVGARKSAPLIELCVDE
CTAAACGAA	MSDNGPQNQRNAPRITFGGPSDSTGSNQNGERSGARSKQRRPQGLPNNTASWFTALTQH
ACGAACTT	MYSFVSEETGTLIVNSVLLFLAFVVFLLVTLAILTALRLCAYCCNIVNVSLVKPSFYVYSRVKNLN
CTAAACGAA	MADSNGTITVEELKKLLEQWNLVIGFLFLTWICLLQFAYANRNRFLYIIKLIFLWLLWPVTLACF
ACGAACGCT	MFHLVDFQVTIAEILLIIMRTFKVSIWNLDYIINLIIKNLSKSLTENKYSQLDEEQPMEID*
CTAAACGAA	MSDNGPQNQRNAPRITFGGPSDSTGSNQNGERSGARSKQRRPQGLPNNTASWFTALTQH
ACGAACGCT	MFHLVDFQVTIAEILLIIMRTFKVSIWNLDYIINLIIKNLSKSLTENKYSQLDEEQPMEID*
CTAAACGAA	MSDNGPQNQRNAPRITFGGPSDSTGSNQNGERSGARSKQRRPQGLPNNTASWFTALTQH
ACGAACTT	MYSFVSEETGTLIVNSVLLFLAFVVFLLVTLAILTALRLCAYCCNIVNVSLVKPSFYVYSRVKNLN
CTAAACGAA	MSDNGPQNQRNAPRITFGGPSDSTGSNQNGERSGARSKQRRPQGLPNNTASWFTALTQH
CTAAACGAA	MFVFLVLLPLVSSQCVNLTTRTQLPPAYTNSFTRGVYYPDKVFRSSVLHSTQDLFLPFFSNVTW
ACGAACTT	MDLFMRIFTIGTVTLKQGEIKDATPSDFVRATATIPIQASLPFGWLIVGVALLAVFQSASKIITLK
ACGAACTT	MYSFVSEETGTLIVNSVLLFLAFVVFLLVTLAILTALRLCAYCCNIVNVSLVKPSFYVYSRVKNLN
CTAAACGAA	MADSNGTITVEELKKLLEQWNLVIGFLFLTWICLLQFAYANRNRFLYIIKLIFLWLLWPVTLACF
ACGAACGCT	MFHLVDFQVTIAEILLIIMRTFKVSIWNLDYIINLIIKNLSKSLTENKYSQLDEEQPMEID*
ACGAAC	MKIILFLALITLATCELYHYQECVRGTTVLLKEPCSSGTYEGNSPFHPLADNKFALTCFSTQFAFA
CTAAACGAA	MKFLVFLGIITTVAAFHQECSLQSCTQHQPYVVDDPCPIHFYSKWYIRVGARKSAPLIELCVDE
CTAAACGAA	MSDNGPQNQRNAPRITFGGPSDSTGSNQNGERSGARSKQRRPQGLPNNTASWFTALTQH
CTAAACGAA	MADSNGTITVEELKKLLEQWNLVIGFLFLTWICLLQFAYANRNRFLYIIKLIFLWLLWPVTLACF
ACGAACGCT	MFHLVDFQVTIAEILLIIMRTFKVSIWNLDYIINLIIKNLSKSLTENKYSQLDEEQPMEID*
CTAAACGAA	MSDNGPQNQRNAPRITFGGPSDSTGSNQNGERSGARSKQRRPQGLPNNTASWFTALTQH
CTAAACGAA	MADSNGTITVEELKKLLEQWNLVIGFLFLTWICLLQFAYANRNRFLYIIKLIFLWLLWPVTLACF
CTAAACGAA	MSDNGPQNQRNAPRITFGGPSDSTGSNQNGERSGARSKQRRPQGLPNNTASWFTALTQH
CTAAACGAA	MFVFLVLLPLVSSQCVNLTTRTQLPPAYTNSFTRGVYYPDKVFRSSVLHSTQDLFLPFFSNVTW
ATAAACGAA	MDLFMRIFTIGTVTLKQGEIKDATPSDFVRATATIPIQASLPFGWLIVGVALLAVFQSASKIITLK
ACGAACTT	MYSFVSEETGTLIVNSVLLFLAFVVFLLVTLAILTALRLCAYCCNIVNVSLVKPSFYVYSRVKNLN
CTAAACGAA	MADSNGTITVEELKKLLEQWNLVIGFLFLTWICLLQFAYANRNRFLYIIKLIFLWLLWPVTLACF
ACGAACGCT	MFHLVDFQVTIAEILLIIMRTFKVSIWNLDYIINLIIKNLSKSLTENKYSQLDEEQPMEID*
ACGAAC	MKIILFLALITLATCELYHYQECVRGTTVLLKEPCSSGTYEGNSPFHPLADNKFALTCFSTQFAFA
CTAAACGAA	MSDNGPQNQRNAPRITFGGPSDSTGSNQNGERSGARSKQRRPQGLPNNTASWFTALTQH
ACGAACGCT	MFHLVDFQVTIAEILLIIMRTFKVSIWNLDYIINLIIKNLSKSLTENKYSQLDEEQPMEID*
CTAAACGAA	MSDNGPQNQRNAPRITFGGPSDSTGSNQNGERSGARSKQRRPQGLPNNTASWFTALTQH
ACGAACTT	MYSFVSEETGTLIVNSVLLFLAFVVFLLVTLAILTALRLCAYCCNIVNVSLVKPSFYVYSRVKNLN
CTAAACGAA	MSDNGPQNQRNAPRITFGGPSDSTGSNQNGERSGARSKQRRPQGLPNNTASWFTALTQH
CTAAACGAA	MADSNGTITVEELKKLLEQWNLVIGFLFLTWICLLQFAYANRNRFLYIIKLIFLWLLWPVTLACF
CTAAACGAA	MSDNGPQNQRNAPRITFGGPSDSTGSNQNGERSGARSKQRRPQGLPNNTASWFTALTQH

CTAAACGAA	MADSNGTITVEELKKLLEQWNLVIGFLFLTWICLLQFAYANRNRFLYIIKLIFLWLLWPVTLACF
CTAAACGAA	MSDNGPQNQRNAPRITFGGPSDSTGSNQNGERSGARSKQRRPQGLPNNTASWFTALTQH
CTAAACGAA	MFVFLVLLPLVSSQCVNLTTRTQLPPAYTNSFTRGVYYPDKVFRSSVLHSTQDLFLPFFSNVTW
CTAAACGAA	MADSNGTITVEELKKLLEQWNLVIGFLFLTWICLLQFAYANRNRFLYIIKLIFLWLLWPVTLACF
ACGAACGCT	MFHLVDFQVTIAEILLIIMRTFKVSIWNLDYIINLIIKNLSKSLTENKYSQLDEEQPMEID*
CTAAACGAA	MSDNGPQNQRNAPRITFGGPSDSTGSNQNGERSGARSKQRRPQGLPNNTASWFTALTQH
CTAAACGAA	MFVFLVLLPLVSSQCVNLTTRTQLPPAYTNSFTRGVYYPDKVFRSSVLHSTQDLFLPFFSNVTW
CTAAACGAA	MSDNGPQNQRNAPRITFGGPSDSTGSNQNGERSGARSKQRRPQGLPNNTASWFTALTQH

indicates if there is a ACGAAC sequence in the “TRS_seq” (TRS sequences), “20_leader_seq” ref

GKEDLKFPRGQGVPINTNSSPDDQIGYYRRATRRIRGGDGKMKDLSPRWYFYYLGTGPEAGLPYGANKDGIIW
FHAIHVSGTNGTKRFDNPVLPFNDGVYFASTEKSNIIRGWIFGTTLDSKTQSLLIVNNATNVVIKVCEFQFCND
KRWQLALSKGVHFVCNLLLLFVTVYSHLLLVAAGLEAPFLYLYALVYFLQSINFVRIIMRLWLCWKCRSKNPLLY
VLAAVYRINWITGGIAIAMACLVGLMWLSYFIASFRLFARTRSMWSFNPETNILLNVPLHGTILTRPLLESELVIG

CPDGVKHVYQLRARSVSPKLFIRQEEVQELYSPIFLIVAAIVFITLCFTLKRKTE*
AGSKSPIQYIDIGNYTVSCLPFTINCQEPKLGSLVVRCSFYEDFLEYHDVRVVLDFI*
GKEDLKFPRGQGVPINTNSSPDDQIGYYRRATRRIRGGDGKMKDLSPRWYFYYLGTGPEAGLPYGANKDGIIW
SRVPDLLV*
VLAAVYRINWITGGIAIAMACLVGLMWLSYFIASFRLFARTRSMWSFNPETNILLNVPLHGTILTRPLLESELVIG

GKEDLKFPRGQGVPINTNSSPDDQIGYYRRATRRIRGGDGKMKDLSPRWYFYYLGTGPEAGLPYGANKDGIIW

GKEDLKFPRGQGVPINTNSSPDDQIGYYRRATRRIRGGDGKMKDLSPRWYFYYLGTGPEAGLPYGANKDGIIW
SRVPDLLV*
GKEDLKFPRGQGVPINTNSSPDDQIGYYRRATRRIRGGDGKMKDLSPRWYFYYLGTGPEAGLPYGANKDGIIW
FHAIHVSGTNGTKRFDNPVLPFNDGVYFASTEKSNIIRGWIFGTTLDSKTQSLLIVNNATNVVIKVCEFQFCND
KRWQLALSKGVHFVCNLLLLFVTVYSHLLLVAAGLEAPFLYLYALVYFLQSINFVRIIMRLWLCWKCRSKNPLLY
SRVPDLLV*
VLAAVYRINWITGGIAIAMACLVGLMWLSYFIASFRLFARTRSMWSFNPETNILLNVPLHGTILTRPLLESELVIG

CPDGVKHVYQLRARSVSPKLFIRQEEVQELYSPIFLIVAAIVFITLCFTLKRKTE*
AGSKSPIQYIDIGNYTVSCLPFTINCQEPKLGSLVVRCSFYEDFLEYHDVRVVLDFI*
GKEDLKFPRGQGVPINTNSSPDDQIGYYRRATRRIRGGDGKMKDLSPRWYFYYLGTGPEAGLPYGANKDGIIW
VLAAVYRINWITGGIAIAMACLVGLMWLSYFIASFRLFARTRSMWSFNPETNILLNVPLHGTILTRPLLESELVIG

GKEDLKFPRGQGVPINTNSSPDDQIGYYRRATRRIRGGDGKMKDLSPRWYFYYLGTGPEAGLPYGANKDGIIW
VLAAVYRINWITGGIAIAMACLVGLMWLSYFIASFRLFARTRSMWSFNPETNILLNVPLHGTILTRPLLESELVIG
GKEDLKFPRGQGVPINTNSSPDDQIGYYRRATRRIRGGDGKMKDLSPRWYFYYLGTGPEAGLPYGANKDGIIW
FHAIHVSGTNGTKRFDNPVLPFNDGVYFASTEKSNIIRGWIFGTTLDSKTQSLLIVNNATNVVIKVCEFQFCND
KRWQLALSKGVHFVCNLLLLFVTVYSHLLLVAAGLEAPFLYLYALVYFLQSINFVRIIMRLWLCWKCRSKNPLLY
SRVPDLLV*
VLAAVYRINWITGGIAIAMACLVGLMWLSYFIASFRLFARTRSMWSFNPETNILLNVPLHGTILTRPLLESELVIG

CPDGVKHVYQLRARSVSPKLFIRQEEVQELYSPIFLIVAAIVFITLCFTLKRKTE*
GKEDLKFPRGQGVPINTNSSPDDQIGYYRRATRRIRGGDGKMKDLSPRWYFYYLGTGPEAGLPYGANKDGIIW

GKEDLKFPRGQGVPINTNSSPDDQIGYYRRATRRIRGGDGKMKDLSPRWYFYYLGTGPEAGLPYGANKDGIIW
SRVPDLLV*
GKEDLKFPRGQGVPINTNSSPDDQIGYYRRATRRIRGGDGKMKDLSPRWYFYYLGTGPEAGLPYGANKDGIIW
VLAAVYRINWITGGIAIAMACLVGLMWLSYFIASFRLFARTRSMWSFNPETNILLNVPLHGTILTRPLLESELVIG
GKEDLKFPRGQGVPINTNSSPDDQIGYYRRATRRIRGGDGKMKDLSPRWYFYYLGTGPEAGLPYGANKDGIIW

VLAAVYRINWITGGIAIAMACLVGLMWLSYFIASFRLFARTRSMWSFNPETNILLNVPLHGTILTRPLLESELVIG
GKEDLKFPRGQGVPINTNSSPDDQIGYYRRATRRIRGGDGKMKDLSPRWYFYYLGTGPEAGLPYGANKDGIIW
VFHAIHVSGTNGTKRFDNPVLPFNDGVYFASTEKSNIIRGWIFGTTLDSKTQSLLIVNNATNVVIKVCEFQFCNDP
VLAAVYRINWITGGIAIAMACLVGLMWLSYFIASFRLFARTRSMWSFNPETNILLNVPLHGTILTRPLLESELVIG

GKEDLKFPRGQGVPINTNSSPDDQIGYYRRATRRIRGGDGKMKDLSPRWYFYYLGTGPEAGLPYGANKDGIIW
VFHAIHVSGTNGTKRFDNPVLPFNDGVYFASTEKSNIIRGWIFGTTLDSKTQSLLIVNNATNVVIKVCEFQFCNDP
GKEDLKFPRGQGVPINTNSSPDDQIGYYRRATRRIRGGDGKMKDLSPRWYFYYLGTGPEAGLPYGANKDGIIW
ers to the 20 nucleotides before the end of leader, and “AUG_postion” and “first_orf_aa” refer to

VATEGALNTPKDHIGTRNPANNAAIVLQLPQGTTLPKGFYAEGSRGGSQASSRSSSRSRNSSRNSTPGSSRGT
FLGVYYHKNNKSWMESEFRVYSSANNCTFEYVSQPFLMDLEGKQGNFKNLREFVFKNIDGYFKIYSKHTPINL
ANYFLCWHTNCYDYCIPYNSVTSSIVITSGDGTTSPISEHDYQIGGYTEKWESGVKDCVVLHSYFTSDYYQLYST
AVILRGHLRIAGHHLGRCDIKDLPKEITVATSRTLSYYKLGASQRVAGDSGFAAYSRYRIGNYKLNTDHSSSSDNI

VATEGALNTPKDHIGTRNPANNAAIVLQLPQGTTLPKGFYAEGSRGGSQASSRSSSRSRNSSRNSTPGSSRGT

AVILRGHLRIAGHHLGRCDIKDLPKEITVATSRTLSYYKLGASQRVAGDSGFAAYSRYRIGNYKLNTDHSSSSDNI

VATEGALNTPKDHIGTRNPANNAAIVLQLPQGTTLPKGFYAEGSRGGSQASSRSSSRSRNSSRNSTPGSSRGT

VATEGALNTPKDHIGTRNPANNAAIVLQLPQGTTLPKGFYAEGSRGGSQASSRSSSRSRNSSRNSTPGSSRGT

VATEGALNTPKDHIGTRNPANNAAIVLQLPQGTTLPKGFYAEGSRGGSQASSRSSSRSRNSSRNSTPGSSRGT
FLGVYYHKNNKSWMESEFRVYSSANNCTFEYVSQPFLMDLEGKQGNFKNLREFVFKNIDGYFKIYSKHTPINL
ANYFLCWHTNCYDYCIPYNSVTSSIVITSGDGTTSPISEHDYQIGGYTEKWESGVKDCVVLHSYFTSDYYQLYST

AVILRGHLRIAGHHLGRCDIKDLPKEITVATSRTLSYYKLGASQRVAGDSGFAAYSRYRIGNYKLNTDHSSSSDNI

VATEGALNTPKDHIGTRNPANNAAIVLQLPQGTTLPKGFYAEGSRGGSQASSRSSSRSRNSSRNSTPGSSRGT
AVILRGHLRIAGHHLGRCDIKDLPKEITVATSRTLSYYKLGASQRVAGDSGFAAYSRYRIGNYKLNTDHSSSSDNI

VATEGALNTPKDHIGTRNPANNAAIVLQLPQGTTLPKGFYAEGSRGGSQASSRSSSRSRNSSRNSTPGSSRGT
AVILRGHLRIAGHHLGRCDIKDLPKEITVATSRTLSYYKLGASQRVAGDSGFAAYSRYRIGNYKLNTDHSSSSDNI
VATEGALNTPKDHIGTRNPANNAAIVLQLPQGTTLPKGFYAEGSRGGSQASSRSSSRSRNSSRNSTPGSSRGT
FLGVYYHKNNKSWMESEFRVYSSANNCTFEYVSQPFLMDLEGKQGNFKNLREFVFKNIDGYFKIYSKHTPINL
ANYFLCWHTNCYDYCIPYNSVTSSIVITSGDGTTSPISEHDYQIGGYTEKWESGVKDCVVLHSYFTSDYYQLYST

AVILRGHLRIAGHHLGRCDIKDLPKEITVATSRTLSYYKLGASQRVAGDSGFAAYSRYRIGNYKLNTDHSSSSDNI

VATEGALNTPKDHIGTRNPANNAAIVLQLPQGTTLPKGFYAEGSRGGSQASSRSSSRSRNSSRNSTPGSSRGT

VATEGALNTPKDHIGTRNPANNAAIVLQLPQGTTLPKGFYAEGSRGGSQASSRSSSRSRNSSRNSTPGSSRGT

VATEGALNTPKDHIGTRNPANNAAIVLQLPQGTTLPKGFYAEGSRGGSQASSRSSSRSRNSSRNSTPGSSRGT
AVILRGHLRIAGHHLGRCDIKDLPKEITVATSRTLSYYKLGASQRVAGDSGFAAYSRYRIGNYKLNTDHSSSSDNI
VATEGALNTPKDHIGTRNPANNAAIVLQLPQGTTLPKGFYAEGSRGGSQASSRSSSRSRNSSRNSTPGSSRGT

AVILRGHLRIAGHHLGRCDIKDLPKEITVATSRTLSYYKLGASQRVAGDSGFAAYSRYRIGNYKLNTDHSSSSDNI
/VATEGALNTPKDHIGTRNPANNAAIVLQLPQGTTLPKGFYAEGSRGGSQASSRSSSRSRNSSRNSTPGSSRGT
PFLGVYYHKNNKSWMESEFRVYSSANNCTFEYVSQPFLMDLEGKQGNFKNLREFVFKNIDGYFKIYSKHTPINL
AVILRGHLRIAGHHLGRCDIKDLPKEITVATSRTLSYYKLGASQRVAGDSGFAAYSRYRIGNYKLNTDHSSSSDNI

/VATEGALNTPKDHIGTRNPANNAAIVLQLPQGTTLPKGFYAEGSRGGSQASSRSSSRSRNSSRNSTPGSSRGT
PFLGVYYHKNNKSWMESEFRVYSSANNCTFEYVSQPFLMDLEGKQGNFKNLREFVFKNIDGYFKIYSKHTPINL
/VATEGALNTPKDHIGTRNPANNAAIVLQLPQGTTLPKGFYAEGSRGGSQASSRSSSRSRNSSRNSTPGSSRGT
the first AUG position and translated orf of the sgmRNA.

SPARMAGNGGDAALALLLLDRLNQLESKMSGKGQQQQGQTVTKKSAAEASKKPRQKRTATKAYNVTQAFG[illegible]
VRDLPQGFSALEPLVDLPIGINITRFQTLLALHRSYLTPGDSSSGWTAGAAAYYVGYLQPRTFLLKYNENGTITDA
TQLSTDTGVEHVTFFIYNKIVDEPEEHVQIHTIDGSSGVVNPVMEPIYDEPTTTTSVPL*
ALLVQ*

SPARMAGNGGDAALALLLLDRLNQLESKMSGKGQQQQGQTVTKKSAAEASKKPRQKRTATKAYNVTQAFG[illegible]

ALLVQ*

SPARMAGNGGDAALALLLLDRLNQLESKMSGKGQQQQGQTVTKKSAAEASKKPRQKRTATKAYNVTQAFG[illegible]

SPARMAGNGGDAALALLLLDRLNQLESKMSGKGQQQQGQTVTKKSAAEASKKPRQKRTATKAYNVTQAFG[illegible]

SPARMAGNGGDAALALLLLDRLNQLESKMSGKGQQQQGQTVTKKSAAEASKKPRQKRTATKAYNVTQAFG[illegible]
VRDLPQGFSALEPLVDLPIGINITRFQTLLALHRSYLTPGDSSSGWTAGAAAYYVGYLQPRTFLLKYNENGTITDA
TQLSTDTGVEHVTFFIYNKIVDEPEEHVQIHTIDGSSGVVNPVMEPIYDEPTTTTSVPL*

ALLVQ*

SPARMAGNGGDAALALLLLDRLNQLESKMSGKGQQQQGQTVTKKSAAEASKKPRQKRTATKAYNVTQAFG[illegible]
ALLVQ*

SPARMAGNGGDAALALLLLDRLNQLESKMSGKGQQQQGQTVTKKSAAEASKKPRQKRTATKAYNVTQAFG[illegible]
ALLVQ*
SPARMAGNGGDAALALLLLDRLNQLESKMSGKGQQQQGQTVTKKSAAEASKKPRQKRTATKAYNVTQAFG[illegible]
VRDLPQGFSALEPLVDLPIGINITRFQTLLALHRSYLTPGDSSSGWTAGAAAYYVGYLQPRTFLLKYNENGTITDA
TQLSTDTGVEHVTFFIYNKIVDEPEEHVQIHTIDGSSGVVNPVMEPIYDEPTTTTSVPL*

ALLVQ*

SPARMAGNGGDAALALLLLDRLNQLESKMSGKGQQQQGQTVTKKSAAEASKKPRQKRTATKAYNVTQAFG[illegible]

SPARMAGNGGDAALALLLLDRLNQLESKMSGKGQQQQGQTVTKKSAAEASKKPRQKRTATKAYNVTQAFG[illegible]

SPARMAGNGGDAALALLLLDRLNQLESKMSGKGQQQQGQTVTKKSAAEASKKPRQKRTATKAYNVTQAFG[illegible]
ALLVQ*
SPARMAGNGGDAALALLLLDRLNQLESKMSGKGQQQQGQTVTKKSAAEASKKPRQKRTATKAYNVTQAFG[illegible]

ALLVQ*

SPARMAGNGGDAALALLLLDRLNQLESKMSGKGQQQQGQTVTKKSAAEASKKPRQKRTATKAYNVTQAFG

VRDLPQGFSALEPLVDLPIGINITRFQTLLALHRSYLTPGDSSSGWTAGAAAYYVGYLQPRTFLLKYNENGTITDA

ALLVQ*

SPARMAGNGGDAALALLLLDRLNQLESKMSGKGQQQQGQTVTKKSAAEASKKPRQKRTATKAYNVTQAFG

VRDLPQGFSALEPLVDLPIGINITRFQTLLALHRSYLTPGDSSSGWTAGAAAYYVGYLQPRTFLLKYNENGTITDA

SPARMAGNGGDAALALLLLDRLNQLESKMSGKGQQQQGQTVTKKSAAEASKKPRQKRTATKAYNVTQAFG

RRGPEQTQGNFGDQELIRQGTDYKHWPQIAQFAPSASAFFGMSRIGMEVTPSGTWLTYTGAIKLDDKDPNFK
AVDCALDPLSETKCTLKSFTVEKGIYQTSNFRVQPTESIVRFPNITNLCPFGEVFNATRFASVYAWNRKRISNCVA

RRGPEQTQGNFGDQELIRQGTDYKHWPQIAQFAPSASAFFGMSRIGMEVTPSGTWLTYTGAIKLDDKDPNFK

RRGPEQTQGNFGDQELIRQGTDYKHWPQIAQFAPSASAFFGMSRIGMEVTPSGTWLTYTGAIKLDDKDPNFK

RRGPEQTQGNFGDQELIRQGTDYKHWPQIAQFAPSASAFFGMSRIGMEVTPSGTWLTYTGAIKLDDKDPNFK

RRGPEQTQGNFGDQELIRQGTDYKHWPQIAQFAPSASAFFGMSRIGMEVTPSGTWLTYTGAIKLDDKDPNFK
AVDCALDPLSETKCTLKSFTVEKGIYQTSNFRVQPTESIVRFPNITNLCPFGEVFNATRFASVYAWNRKRISNCVA

RRGPEQTQGNFGDQELIRQGTDYKHWPQIAQFAPSASAFFGMSRIGMEVTPSGTWLTYTGAIKLDDKDPNFK

RRGPEQTQGNFGDQELIRQGTDYKHWPQIAQFAPSASAFFGMSRIGMEVTPSGTWLTYTGAIKLDDKDPNFK

RRGPEQTQGNFGDQELIRQGTDYKHWPQIAQFAPSASAFFGMSRIGMEVTPSGTWLTYTGAIKLDDKDPNFK
AVDCALDPLSETKCTLKSFTVEKGIYQTSNFRVQPTESIVRFPNITNLCPFGEVFNATRFASVYAWNRKRISNCVA

RRGPEQTQGNFGDQELIRQGTDYKHWPQIAQFAPSASAFFGMSRIGMEVTPSGTWLTYTGAIKLDDKDPNFK

RRGPEQTQGNFGDQELIRQGTDYKHWPQIAQFAPSASAFFGMSRIGMEVTPSGTWLTYTGAIKLDDKDPNFK

RRGPEQTQGNFGDQELIRQGTDYKHWPQIAQFAPSASAFFGMSRIGMEVTPSGTWLTYTGAIKLDDKDPNFK

RRGPEQTQGNFGDQELIRQGTDYKHWPQIAQFAPSASAFFGMSRIGMEVTPSGTWLTYTGAIKLDDKDPNFK

RRGPEQTQGNFGDQELIRQGTDYKHWPQIAQFAPSASAFFGMSRIGMEVTPSGTWLTYTGAIKLDDKDPNFK
AVDCALDPLSETKCTLKSFTVEKGIYQTSNFRVQPTESIVRFPNITNLCPFGEVFNATRFASVYAWNRKRISNCVA

RRGPEQTQGNFGDQELIRQGTDYKHWPQIAQFAPSASAFFGMSRIGMEVTPSGTWLTYTGAIKLDDKDPNFK
AVDCALDPLSETKCTLKSFTVEKGIYQTSNFRVQPTESIVRFPNITNLCPFGEVFNATRFASVYAWNRKRISNCVA
RRGPEQTQGNFGDQELIRQGTDYKHWPQIAQFAPSASAFFGMSRIGMEVTPSGTWLTYTGAIKLDDKDPNFK

DQVILLNKHIDAYKTFPPTEPKKDKKKKADETQALPQRQKKQQTVTLLPAADLDDFSKQLQQSMSSADSTQA
DYSVLYNSASFSTFKCYGVSPTKLNDLCFTNVYADSFVIRGDEVRQIAPGQTGKIADYNYKLPDDFTGCVIAWN

DQVILLNKHIDAYKTFPPTEPKKDKKKKADETQALPQRQKKQQTVTLLPAADLDDFSKQLQQSMSSADSTQA

DQVILLNKHIDAYKTFPPTEPKKDKKKKADETQALPQRQKKQQTVTLLPAADLDDFSKQLQQSMSSADSTQA

DQVILLNKHIDAYKTFPPTEPKKDKKKKADETQALPQRQKKQQTVTLLPAADLDDFSKQLQQSMSSADSTQA

DQVILLNKHIDAYKTFPPTEPKKDKKKKADETQALPQRQKKQQTVTLLPAADLDDFSKQLQQSMSSADSTQA
DYSVLYNSASFSTFKCYGVSPTKLNDLCFTNVYADSFVIRGDEVRQIAPGQTGKIADYNYKLPDDFTGCVIAWN

DQVILLNKHIDAYKTFPPTEPKKDKKKKADETQALPQRQKKQQTVTLLPAADLDDFSKQLQQSMSSADSTQA

DQVILLNKHIDAYKTFPPTEPKKDKKKKADETQALPQRQKKQQTVTLLPAADLDDFSKQLQQSMSSADSTQA

DQVILLNKHIDAYKTFPPTEPKKDKKKKADETQALPQRQKKQQTVTLLPAADLDDFSKQLQQSMSSADSTQA
DYSVLYNSASFSTFKCYGVSPTKLNDLCFTNVYADSFVIRGDEVRQIAPGQTGKIADYNYKLPDDFTGCVIAWN

DQVILLNKHIDAYKTFPPTEPKKDKKKKADETQALPQRQKKQQTVTLLPAADLDDFSKQLQQSMSSADSTQA

DQVILLNKHIDAYKTFPPTEPKKDKKKKADETQALPQRQKKQQTVTLLPAADLDDFSKQLQQSMSSADSTQA

DQVILLNKHIDAYKTFPPTEPKKDKKKKADETQALPQRQKKQQTVTLLPAADLDDFSKQLQQSMSSADSTQA

DQVILLNKHIDAYKTFPPTEPKKDKKKKADETQALPQRQKKQQTVTLLPAADLDDFSKQLQQSMSSADSTQA

DQVILLNKHIDAYKTFPPTEPKKDKKKKADETQALPQRQKKQQTVTLLPAADLDDFSKQLQQSMSSADSTQA
DYSVLYNSASFSTFKCYGVSPTKLNDLCFTNVYADSFVIRGDEVRQIAPGQTGKIADYNYKLPDDFTGCVIAWN

DQVILLNKHIDAYKTFPPTEPKKDKKKKADETQALPQRQKKQQTVTLLPAADLDDFSKQLQQSMSSADSTQA
DYSVLYNSASFSTFKCYGVSPTKLNDLCFTNVYADSFVIRGDEVRQIAPGQTGKIADYNYKLPDDFTGCVIAWN
DQVILLNKHIDAYKTFPPTEPKKDKKKKADETQALPQRQKKQQTVTLLPAADLDDFSKQLQQSMSSADSTQA

*

SNNLDSKVGGNYNYLYRLFRKSNLKPFERDISTEIYQAGSTPCNGVEGFNCYFPLQSYGFQPTNGVGYQPYRVV

*

*

*

*

SNNLDSKVGGNYNYLYRLFRKSNLKPFERDISTEIYQAGSTPCNGVEGFNCYFPLQSYGFQPTNGVGYQPYRVV

*

*

*

SNNLDSKVGGNYNYLYRLFRKSNLKPFERDISTEIYQAGSTPCNGVEGFNCYFPLQSYGFQPTNGVGYQPYRVV

*

*

*

*

*

SNNLDSKVGGNYNYLYRLFRKSNLKPFERDISTEIYQAGSTPCNGVEGFNCYFPLQSYGFQPTNGVGYQPYRVV

*

SNNLDSKVGGNYNYLYRLFRKSNLKPFERDISTEIYQAGSTPCNGVEGFNCYFPLQSYGFQPTNGVGYQPYRVV

*

'VLSFELLHAPATVCGPKKSTNLVKNKCVNFNFNGLTGTGVLTESNKKFLPFQQFGRDIADTTDAVRDPQTLEILI

'VLSFELLHAPATVCGPKKSTNLVKNKCVNFNFNGLTGTGVLTESNKKFLPFQQFGRDIADTTDAVRDPQTLEILI

'VLSFELLHAPATVCGPKKSTNLVKNKCVNFNFNGLTGTGVLTESNKKFLPFQQFGRDIADTTDAVRDPQTLEILI

'VLSFELLHAPATVCGPKKSTNLVKNKCVNFNFNGLTGTGVLTESNKKFLPFQQFGRDIADTTDAVRDPQTLEILI

'VLSFELLHAPATVCGPKKSTNLVKNKCVNFNFNGLTGTGVLTESNKKFLPFQQFGRDIADTTDAVRDPQTLEILI

DITPCSFGGVSVITPGTNTSNQVAVLYQDVNCTEVPVAIHADQLTPTWRVYSTGSNVFQTRAGCLIGAEHVNN

DITPCSFGGVSVITPGTNTSNQVAVLYQDVNCTEVPVAIHADQLTPTWRVYSTGSNVFQTRAGCLIGAEHVNN

DITPCSFGGVSVITPGTNTSNQVAVLYQDVNCTEVPVAIHADQLTPTWRVYSTGSNVFQTRAGCLIGAEHVNN

DITPCSFGGVSVITPGTNTSNQVAVLYQDVNCTEVPVAIHADQLTPTWRVYSTGSNVFQTRAGCLIGAEHVNN

DITPCSFGGVSVITPGTNTSNQVAVLYQDVNCTEVPVAIHADQLTPTWRVYSTGSNVFQTRAGCLIGAEHVNN

SYECDIPIGAGICASYQTQTNSPRRARSVASQSIIAYTMSLGAENSVAYSNNSIAIPTNFTISVTTEILPVSMTKTS

SYECDIPIGAGICASYQTQTNSPRRARSVASQSIIAYTMSLGAENSVAYSNNSIAIPTNFTISVTTEILPVSMTKTS

SYECDIPIGAGICASYQTQTNSPRRARSVASQSIIAYTMSLGAENSVAYSNNSIAIPTNFTISVTTEILPVSMTKTS

SYECDIPIGAGICASYQTQTNSPRRARSVASQSIIAYTMSLGAENSVAYSNNSIAIPTNFTISVTTEILPVSMTKTS

SYECDIPIGAGICASYQTQTNSPRRARSVASQSIIAYTMSLGAENSVAYSNNSIAIPTNFTISVTTEILPVSMTKTS

VDCTMYICGDSTECSNLLLQYGSFCTQLNRALTGIAVEQDKNTQEVFAQVKQIYKTPPIKDFGGFNFSQILPDPS

VDCTMYICGDSTECSNLLLQYGSFCTQLNRALTGIAVEQDKNTQEVFAQVKQIYKTPPIKDFGGFNFSQILPDPS

VDCTMYICGDSTECSNLLLQYGSFCTQLNRALTGIAVEQDKNTQEVFAQVKQIYKTPPIKDFGGFNFSQILPDPS

VDCTMYICGDSTECSNLLLQYGSFCTQLNRALTGIAVEQDKNTQEVFAQVKQIYKTPPIKDFGGFNFSQILPDPS

VDCTMYICGDSTECSNLLLQYGSFCTQLNRALTGIAVEQDKNTQEVFAQVKQIYKTPPIKDFGGFNFSQILPDPS

KPSKRSFIEDLLFNKVTLADAGFIKQYGDCLGDIAARDLICAQKFNGLTVLPPLLTDEMIAQYTSALLAGTITSGW

KPSKRSFIEDLLFNKVTLADAGFIKQYGDCLGDIAARDLICAQKFNGLTVLPPLLTDEMIAQYTSALLAGTITSGW

KPSKRSFIEDLLFNKVTLADAGFIKQYGDCLGDIAARDLICAQKFNGLTVLPPLLTDEMIAQYTSALLAGTITSGW

KPSKRSFIEDLLFNKVTLADAGFIKQYGDCLGDIAARDLICAQKFNGLTVLPPLLTDEMIAQYTSALLAGTITSGW

KPSKRSFIEDLLFNKVTLADAGFIKQYGDCLGDIAARDLICAQKFNGLTVLPPLLTDEMIAQYTSALLAGTITSGW

TFGAGAALQIPFAMQMAYRFNGIGVTQNVLYENQKLIANQFNSAIGKIQDSLSSTASALGKLQDVVNQNAQ

TFGAGAALQIPFAMQMAYRFNGIGVTQNVLYENQKLIANQFNSAIGKIQDSLSSTASALGKLQDVVNQNAQ

TFGAGAALQIPFAMQMAYRFNGIGVTQNVLYENQKLIANQFNSAIGKIQDSLSSTASALGKLQDVVNQNAQ

TFGAGAALQIPFAMQMAYRFNGIGVTQNVLYENQKLIANQFNSAIGKIQDSLSSTASALGKLQDVVNQNAQ

TFGAGAALQIPFAMQMAYRFNGIGVTQNVLYENQKLIANQFNSAIGKIQDSLSSTASALGKLQDVVNQNAQ

ALNTLVKQLSSNFGAISSVLNDILSRLDKVEAEVQIDRLITGRLQSLQTYVTQQLIRAAEIRASANL

ALNTLVKQLSSNFGAISSVLNDILSRLDKVEAEVQIDRLITGRLQSLQTYVTQQLIRAAEIRASANL

ALNTLVKQLSSNFGAISSVLNDILSRLDKVEAEVQIDRLITGRLQSLQTYVTQQLIRAAEIRASANL

ALNTLVKQLSSNFGAISSVLNDILSRLDKVEAEVQIDRLITGRLQSLQTYVTQQLIRAAEIRASANL

ALNTLVKQLSSNFGAISSVLNDILSRLDKVEAEVQIDRLITGRLQSLQTYVTQQLIRAAEIRASANL

	subgenome	leader_end	TRS_start	nb_count	normalized_count
Patient1-day1					
Patient2-day1					
Patient3-day1					
Patient4-day1					
Patient5-day1					
Patient6-day1	1	74	21055	19(19,18,18,(	14.38(14.38,13.62,13.62,0.(
Patient6-day3					
Patient6-day9					
Patient7-day1	1	65	5080	200(192,180,	140.51(134.89,126.46,120.[illegible]
Patient7-day3					
Patient7-day5					
Patient7-day7					
Patient8-day1					
Patient8-day3					
Patient8-day9					

The numbers in the bracket are (reads with left primers, reads with right primers, reads with both prim
Normalized count=(Read count/Total number of read mapped on reference genome)*1000000.
Total number of read mapped on reference genome is 9639, 1095598, 872890, 44932, 153792, 13214[illegible]
“leader_end” and “TRS_start” refer to the position of the end of leader and the position of the start of

)0,0.00)

84,0.00,0.00)

ners, same junction on paired reads with at least a primer).

29, 95245, 65459, 1423395, 42912, 20046, 75885, 119531, 72674 and 109955 excluding the map
TRS identified in the reads >10.

ꞌped reads unpaired, not primary alignment and supplementary alignment.

	subgenome	leader_end	TRS_start	ACGAAC	ATG_postion	known_ATG
Patient1-day1						
Patient2-day1						
Patient3-day1						
Patient4-day1						
Patient5-day1						
Patient6-day1	1	74	21055	no	21071	-
Patient6-day3						
Patient6-day9						
Patient7-day1	1	65	5080	no	5103	-
Patient7-day3						
Patient7-day5						
Patient7-day7						
Patient8-day1						
Patient8-day3						
Patient8-day9						

“peak_leader” and “peak_TRS_start” point to the leader-TRS junctions in sheet "novel", “ACGAAC”

20_leader_se	TRS_seq	first_orf_aa
AGATCTGTTC	GACCCTAAG/	MLQKKMTLKRVFSLTFVGLYNKS*
ATCTCTTGTA	TAAAATAAA/	MKVKHFMFYLMMTLYVLRLLSTTTQLILVFWVGTCQH*

' indicates if there is a ACGAAC sequences in the “TRS_seq” (TRS sequences), “20_leader_seq” re

fers to the 20 sequences before the end of the leader, “AUG_postion” and “first_orf_aa” refer tc

› the first AUG position and translated orf of the sgmRNA, and “known_ATG” indicates if the first

AUG position is the same as a known sgmRNA.

Supplemental tables

Click here to access/download;Table;Supplemental_Material_6.xlsx

subgenome	peak_leader_	peak_TRS_sta	ACGAAC	ATG_postion	known_ATG
Shared by all	48	26443	yes	26523	M
	74	21058	no	21071	-
	75	26486	no	26523	M
	63	5785	no	5859	-
	68	4580	no	4590	-
Shared by monkeys	58	26917	yes	27202	ORF6
	69	19112	no	19148	-
	70	14372	no	14381	-
	62	3808	no	3813	-
	63	23922	no	24051	-
	67	22503	no	22614	-
	58	29871	-	-	-

20_leader_seq	TRS_seq	first_orf_aa
AAACCAACCAACTTTCGATC	TCTTCTAGAGTTCCTGATCT	MADSNGTITVEELKKLLEQWNLVIGFLFLTW
AGATCTGTTCTCTAAACGAA	CCTAAGACTAAAA	MLQKKMTLKRVFSLTFVGLYNKS*
GATCTGTTCTCTAAACGAAC	TATATTAGTTTTTCTGTTTG	MADSNGTITVEELKKLLEQWNLVIGFLFLTW
CGATCTCTTGTAGATCTGTT	TTCTAAAGAAACTTTGTATT	MFSTKKTVTQQP*
TCTTGTAGATCTGTTCTCTA	AACGATCTAA	MKLLLQCHLAM*
ACTTTCGATCTCTTGTAGAT	CGCTTCTAGAAAGTGAACT	MFHLVDFQVTIAEILLIIMRTFKVSIWNLDYIII
CTTGTAGATCTGTTCTCTAA	ACAAAGCTTATAAAATAGA	MPHILTNSQMVYAYFGIAMSIDILLIPLFVDL
TTGTAGATCTGTTCTCTAAA	CAAACTTTA	MFYSLQCSHLQVLDH*
TCGATCTCTTGTAGATCTGT	TCTCT	MTNLFQAFWK*
CGATCTCTTGTAGATCTGTT	AAATTTACAAAACACCACC	MLASSNNMVIALVILLLETSFVHKSLTALLFCH
CTCTTGTAGATCTGTTCTCT	AAACTTCTAACTTTAGAGT	MLGTGRESATVLLIILSYIIPHHFPLLSVMECLL
-	-	-

ICLLQFAYANRNRFLYIIKLIFLWLLWPVTLACFVLAAVYRINWITGGIAIAMACLVGLMWLSYFIASFRLFART

ICLLQFAYANRNRFLYIIKLIFLWLLWPVTLACFVLAAVYRINWITGGIAIAMACLVGLMWLSYFIASFRLFART

NLIIKNLSKSLTENKYSQLDEEQPMEID*
TLECYLTLTCLVVMVAVCM*

HLCSQMK*
LN*

RSMWSFNPETNILLNVPLHGTILTRPLLESELVIGAVILRGHLRIAGHHLGRCDIKDLPKEITVATSRTLSYYKLGAS

RSMWSFNPETNILLNVPLHGTILTRPLLESELVIGAVILRGHLRIAGHHLGRCDIKDLPKEITVATSRTLSYYKLGAS

SQRVAGDSGFAAYSRYRIGNYKLNTDHSSSSDNIALLVQ*

SQRVAGDSGFAAYSRYRIGNYKLNTDHSSSSDNIALLVQ*

Click here to access/download;Figure;All_figures_high_resolution.pdf

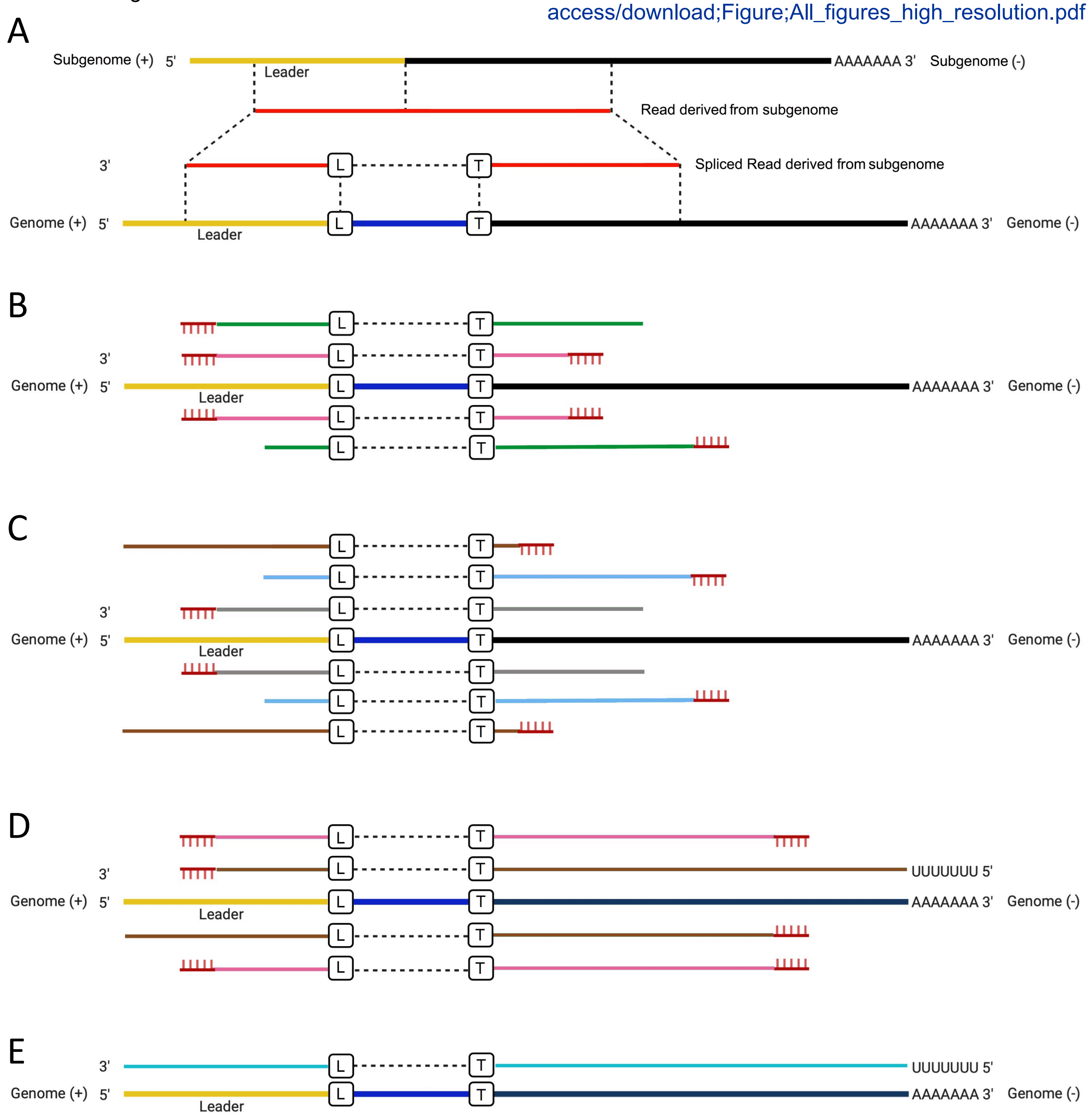

Figure 1

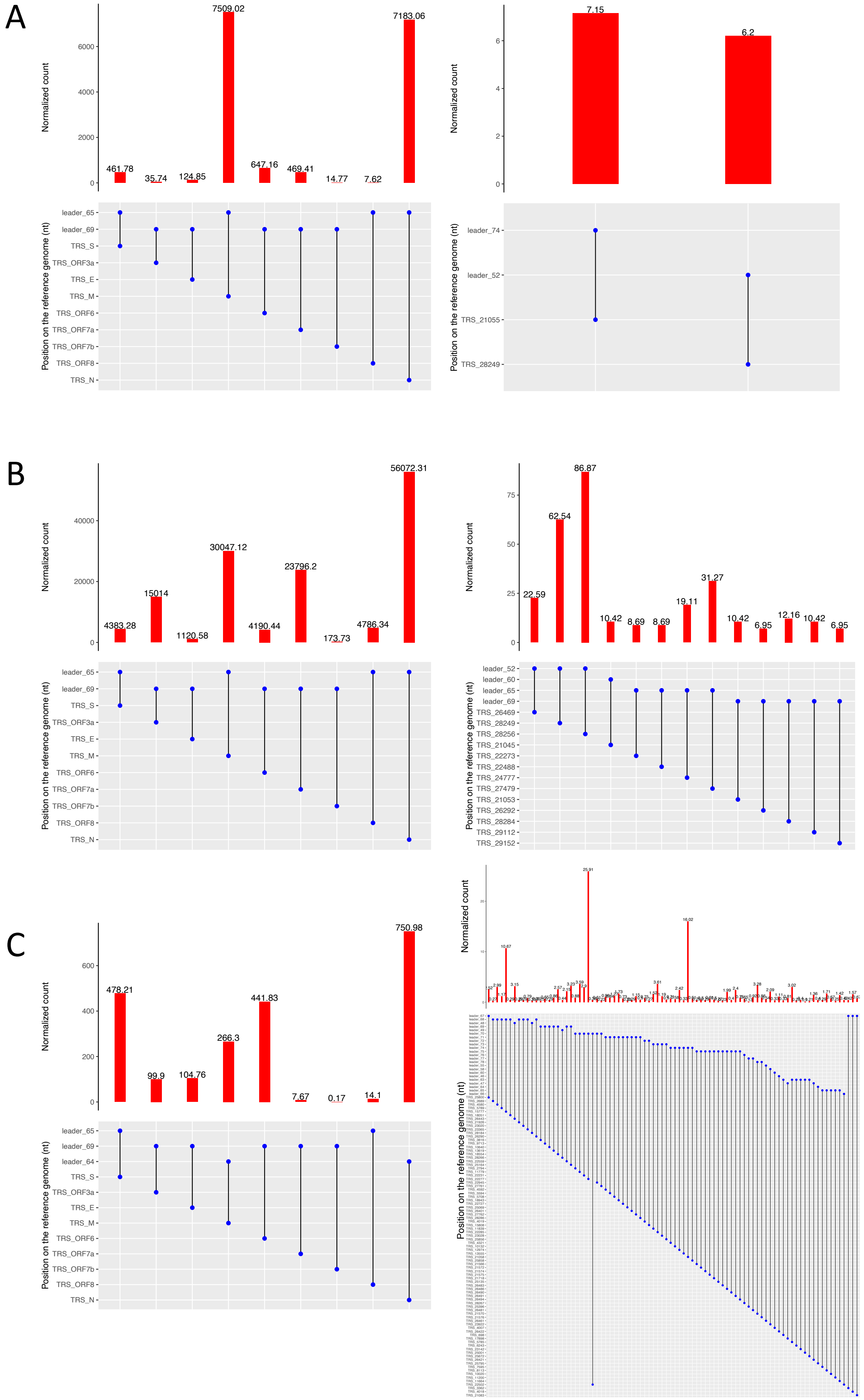
A
Normalized count
461.78
35.74
124.85
7509.02
647.16
469.41
14.77
7.62
7183.06
Position on the reference genome (nt)
leader_65
leader_69
TRS_S
TRS_ORF3a
TRS_E
TRS_M
TRS_ORF6
TRS_ORF7a
TRS_ORF7b
TRS_ORF8
TRS_N
7.15
6.2
leader_74
leader_52
TRS_21055
TRS_28249
B
4383.28
15014
1120.58
30047.12
4190.44
23796.2
173.73
4786.34
56072.31
22.59
62.54
86.87
10.42
8.69
8.69
19.11
31.27
10.42
6.95
12.16
10.42
6.95
leader_52
leader_60
leader_65
leader_69
TRS_26469
TRS_28249
TRS_28256
TRS_21045
TRS_22273
TRS_22488
TRS_24777
TRS_27479
TRS_21053
TRS_26292
TRS_28284
TRS_29112
TRS_29152
C
478.21
99.9
104.76
266.3
441.83
7.67
0.17
14.1
750.98
leader_65
leader_69
leader_64
TRS_S
TRS_ORF3a
TRS_E
TRS_M
TRS_ORF6
TRS_ORF7a
TRS_ORF7b
TRS_ORF8
TRS_N
25.91
16.02
10.67

Figure 2

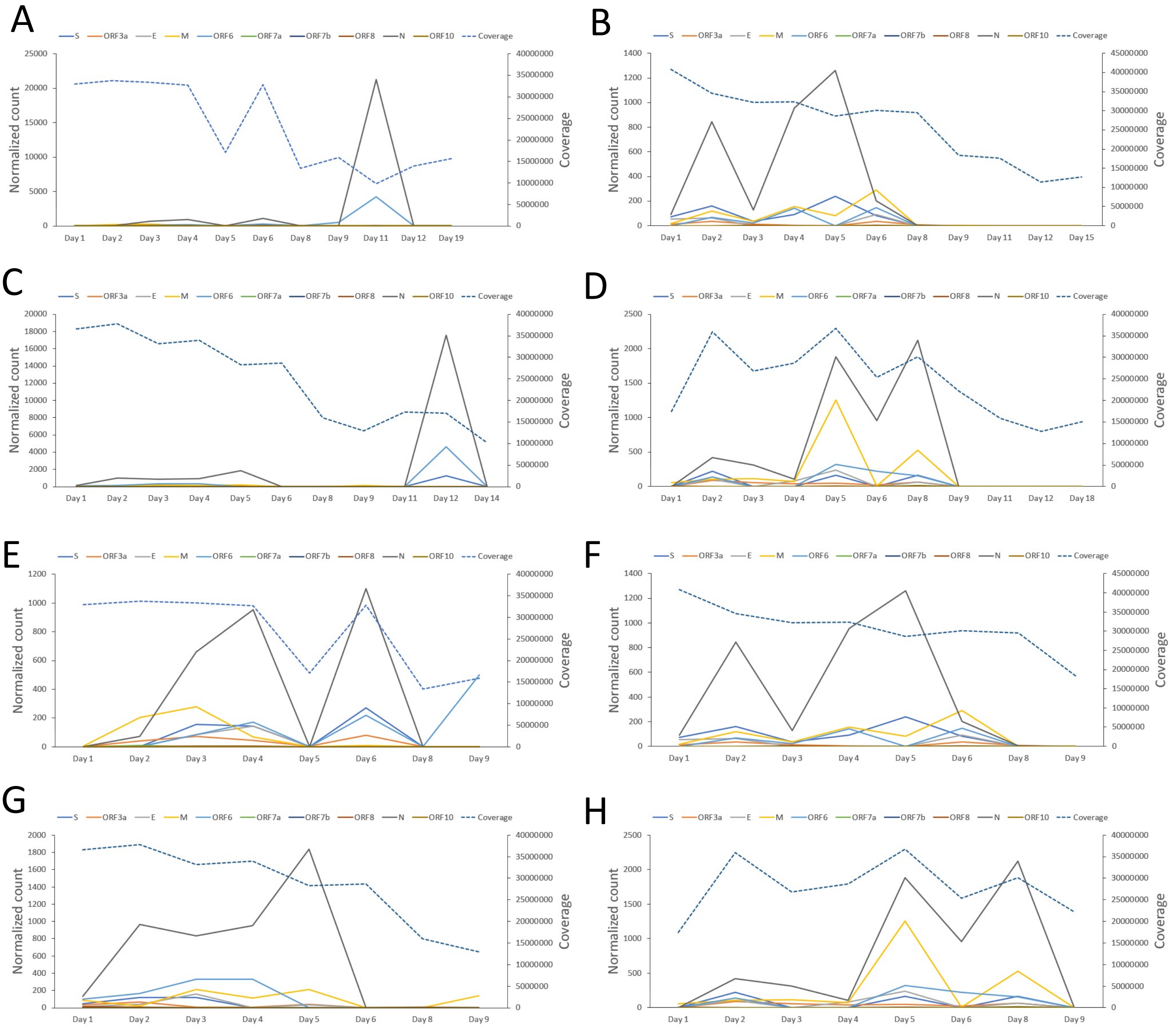
A
S ORF3a E M ORF6 ORF7a ORF7b ORF8 N ORF10 Coverage
Normalized count
Coverage
0 5000 10000 15000 20000 25000
0 5000000 10000000 15000000 20000000 25000000 30000000 35000000 40000000
Day 1 Day 2 Day 3 Day 4 Day 5 Day 6 Day 8 Day 9 Day 11 Day 12 Day 19
B
S ORF3a E M ORF6 ORF7a ORF7b ORF8 N ORF10 Coverage
Normalized count
Coverage
0 200 400 600 800 1000 1200 1400
0 5000000 10000000 15000000 20000000 25000000 30000000 35000000 40000000 45000000
Day 1 Day 2 Day 3 Day 4 Day 5 Day 6 Day 8 Day 9 Day 11 Day 12 Day 15
C
S ORF3a E M ORF6 ORF7a ORF7b ORF8 N ORF10 Coverage
Normalized count
Coverage
0 2000 4000 6000 8000 10000 12000 14000 16000 18000 20000
0 5000000 10000000 15000000 20000000 25000000 30000000 35000000 40000000
Day 1 Day 2 Day 3 Day 4 Day 5 Day 6 Day 8 Day 9 Day 11 Day 12 Day 14
D
S ORF3a E M ORF6 ORF7a ORF7b ORF8 N ORF10 Coverage
Normalized count
Coverage
0 500 1000 1500 2000 2500
0 5000000 10000000 15000000 20000000 25000000 30000000 35000000 40000000
Day 1 Day 2 Day 3 Day 4 Day 5 Day 6 Day 8 Day 9 Day 11 Day 12 Day 18
E
S ORF3a E M ORF6 ORF7a ORF7b ORF8 N ORF10 Coverage
Normalized count
Coverage
0 200 400 600 800 1000 1200
0 5000000 10000000 15000000 20000000 25000000 30000000 35000000 40000000
Day 1 Day 2 Day 3 Day 4 Day 5 Day 6 Day 8 Day 9
F
S ORF3a E M ORF6 ORF7a ORF7b ORF8 N ORF10 Coverage
Normalized count
Coverage
0 200 400 600 800 1000 1200 1400
0 5000000 10000000 15000000 20000000 25000000 30000000 35000000 40000000 45000000
Day 1 Day 2 Day 3 Day 4 Day 5 Day 6 Day 8 Day 9
G
S ORF3a E M ORF6 ORF7a ORF7b ORF8 N ORF10 Coverage
Normalized count
Coverage
0 200 400 600 800 1000 1200 1400 1600 1800 2000
0 5000000 10000000 15000000 20000000 25000000 30000000 35000000 40000000
Day 1 Day 2 Day 3 Day 4 Day 5 Day 6 Day 8 Day 9
H
S ORF3a E M ORF6 ORF7a ORF7b ORF8 N ORF10 Coverage
Normalized count
Coverage
0 500 1000 1500 2000 2500
0 5000000 10000000 15000000 20000000 25000000 30000000 35000000 40000000
Day 1 Day 2 Day 3 Day 4 Day 5 Day 6 Day 8 Day 9

Figure 3

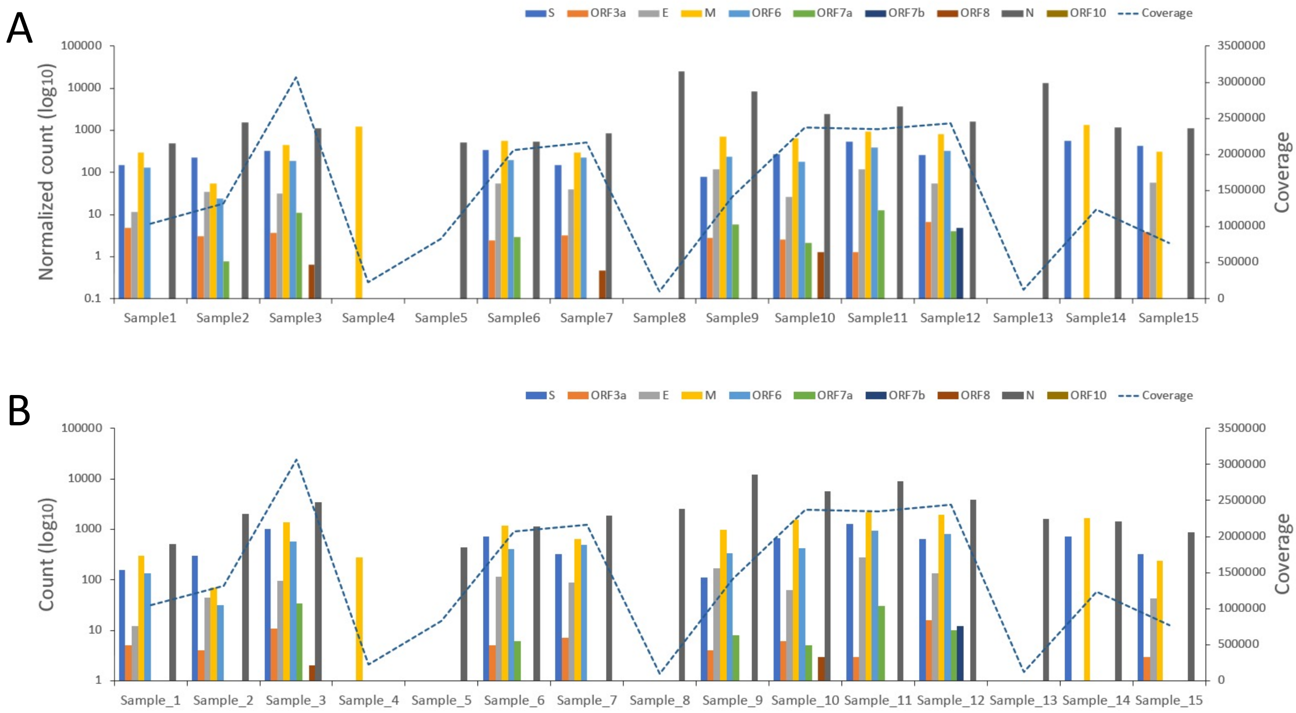
A
S
ORF3a
E
M
ORF6
ORF7a
ORF7b
ORF8
N
ORF10
Coverage
Normalized count (log10)
Coverage
Sample1
Sample2
Sample3
Sample4
Sample5
Sample6
Sample7
Sample8
Sample9
Sample10
Sample11
Sample12
Sample13
Sample14
Sample15
B
Count (log10)
Sample_1
Sample_2
Sample_3
Sample_4
Sample_5
Sample_6
Sample_7
Sample_8
Sample_9
Sample_10
Sample_11
Sample_12
Sample_13
Sample_14
Sample_15

Figure 4

A

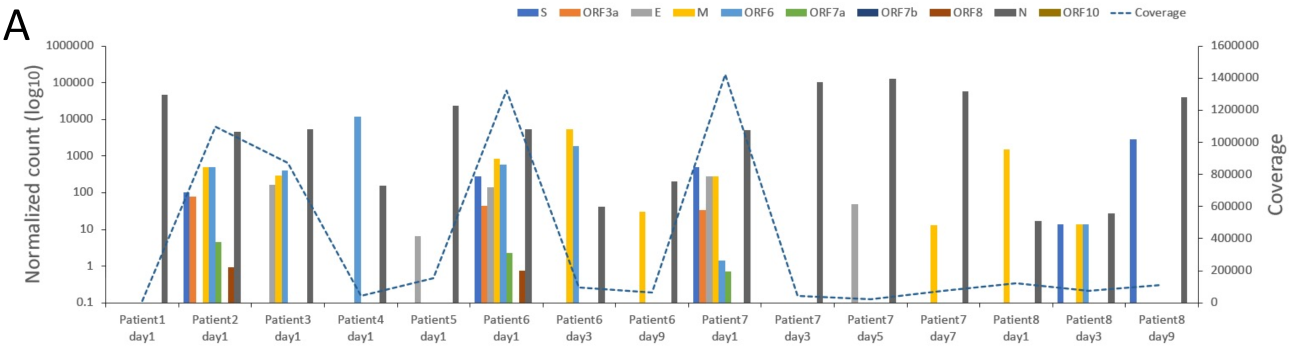
S
ORF3a
E
M
ORF6
ORF7a
ORF7b
ORF8
N
ORF10
Coverage
Normalized count (log10)
Coverage
1000000
100000
10000
1000
100
10
1
0.1
1600000
1400000
1200000
1000000
800000
600000
400000
200000
0
Patient1 day1
Patient2 day1
Patient3 day1
Patient4 day1
Patient5 day1
Patient6 day1
Patient6 day3
Patient6 day9
Patient7 day1
Patient7 day3
Patient7 day5
Patient7 day7
Patient8 day1
Patient8 day3
Patient8 day9

B

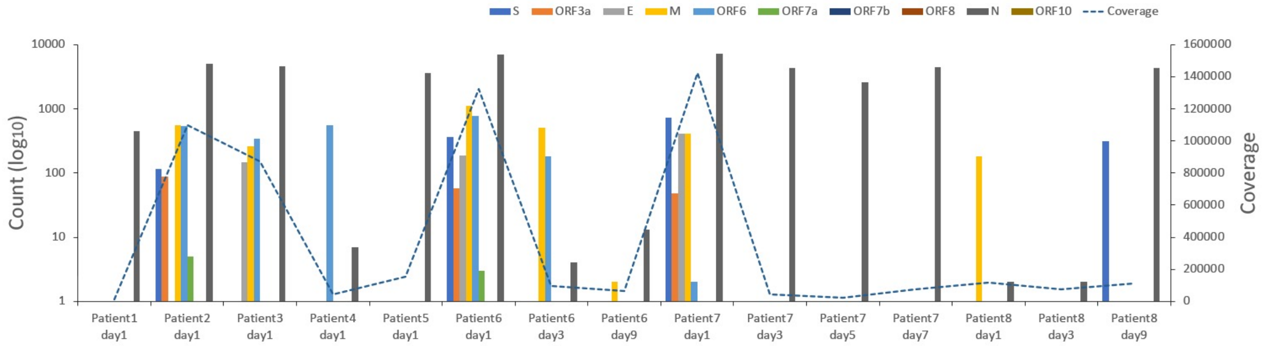
S
ORF3a
E
M
ORF6
ORF7a
ORF7b
ORF8
N
ORF10
Coverage
Count (log10)
Coverage
10000
1000
100
10
1
1600000
1400000
1200000
1000000
800000
600000
400000
200000
0
Patient1 day1
Patient2 day1
Patient3 day1
Patient4 day1
Patient5 day1
Patient6 day1
Patient6 day3
Patient6 day9
Patient7 day1
Patient7 day3
Patient7 day5
Patient7 day7
Patient8 day1
Patient8 day3
Patient8 day9

Figure 5

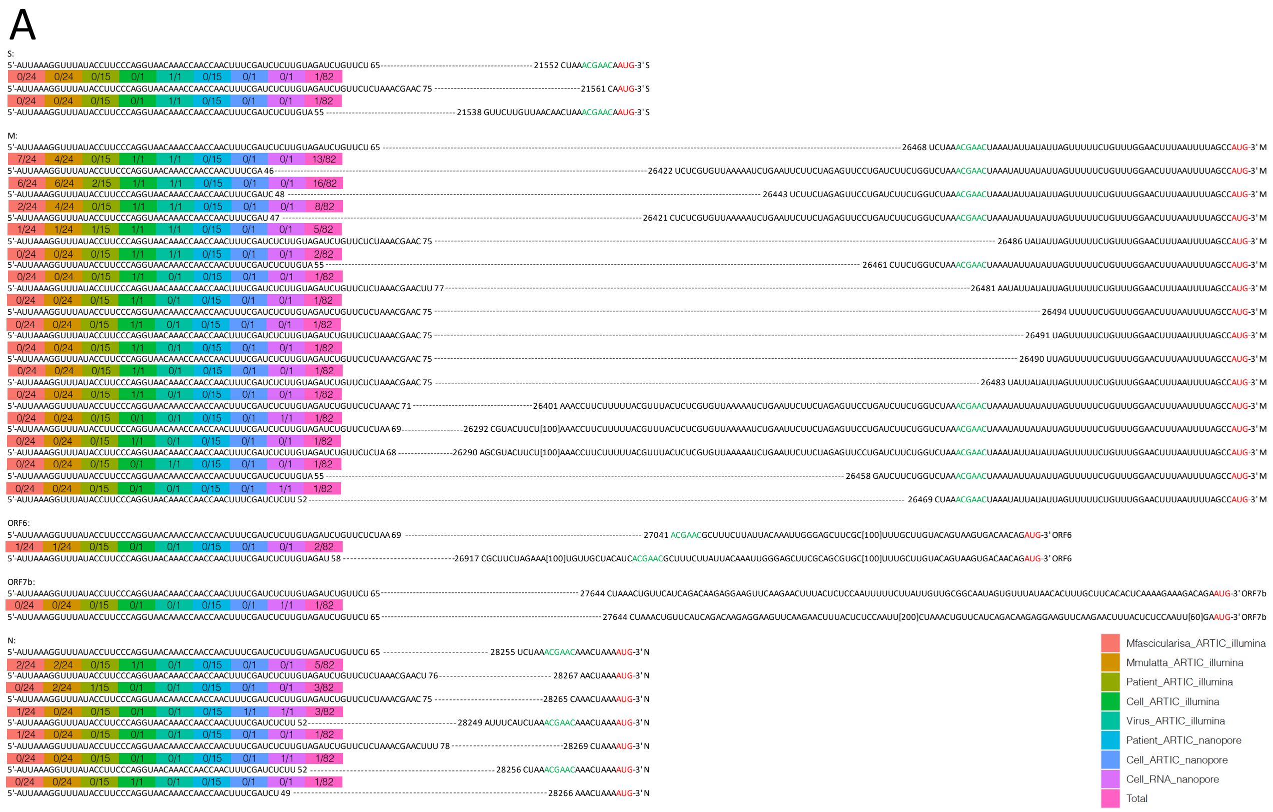
A
S:
M:
ORF6:
ORF7b:
N:
Mfascicularisa_ARTIC_illumina
Mmulatta_ARTIC_illumina
Patient_ARTIC_illumina
Cell_ARTIC_illumina
Virus_ARTIC_illumina
Patient_ARTIC_nanopore
Cell_ARTIC_nanopore
Cell_RNA_nanopore
Total

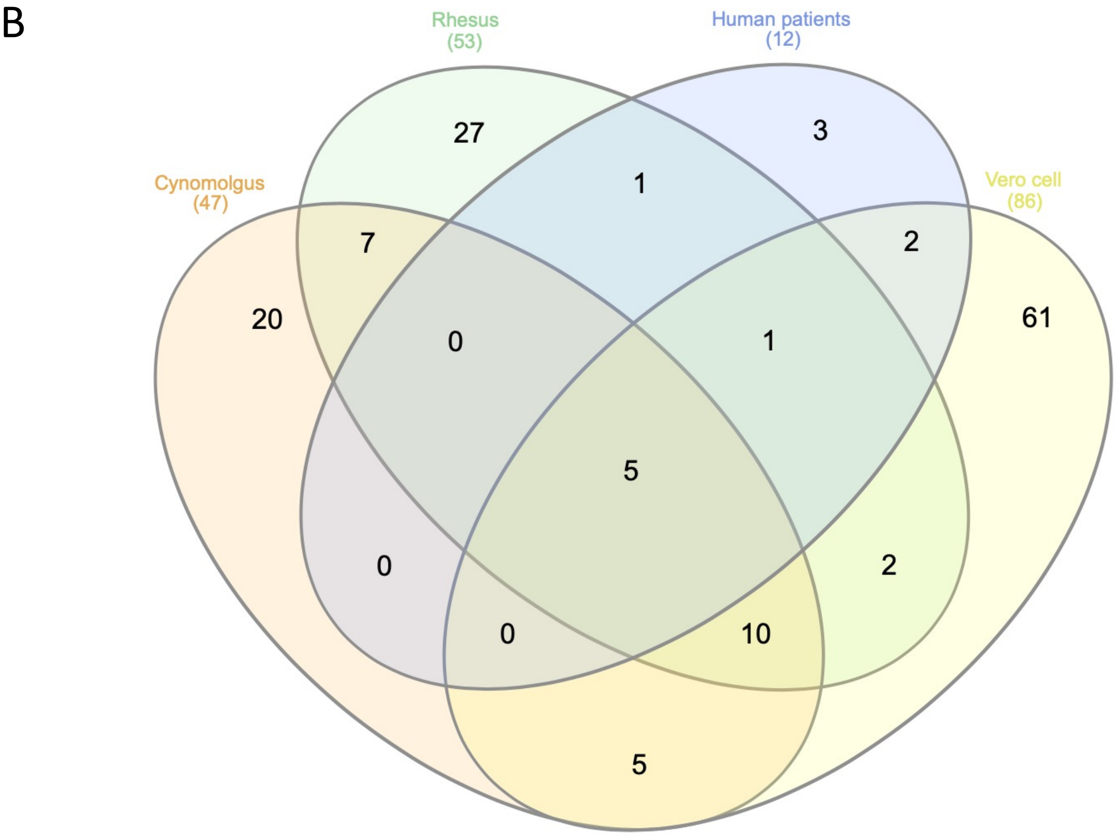
B
Rhesus (53)
Human patients (12)
Cynomolgus (47)
Vero cell (86)
27
3
1
7
2
20
0
1
61
5
0
2
0
10
5

Figure 6

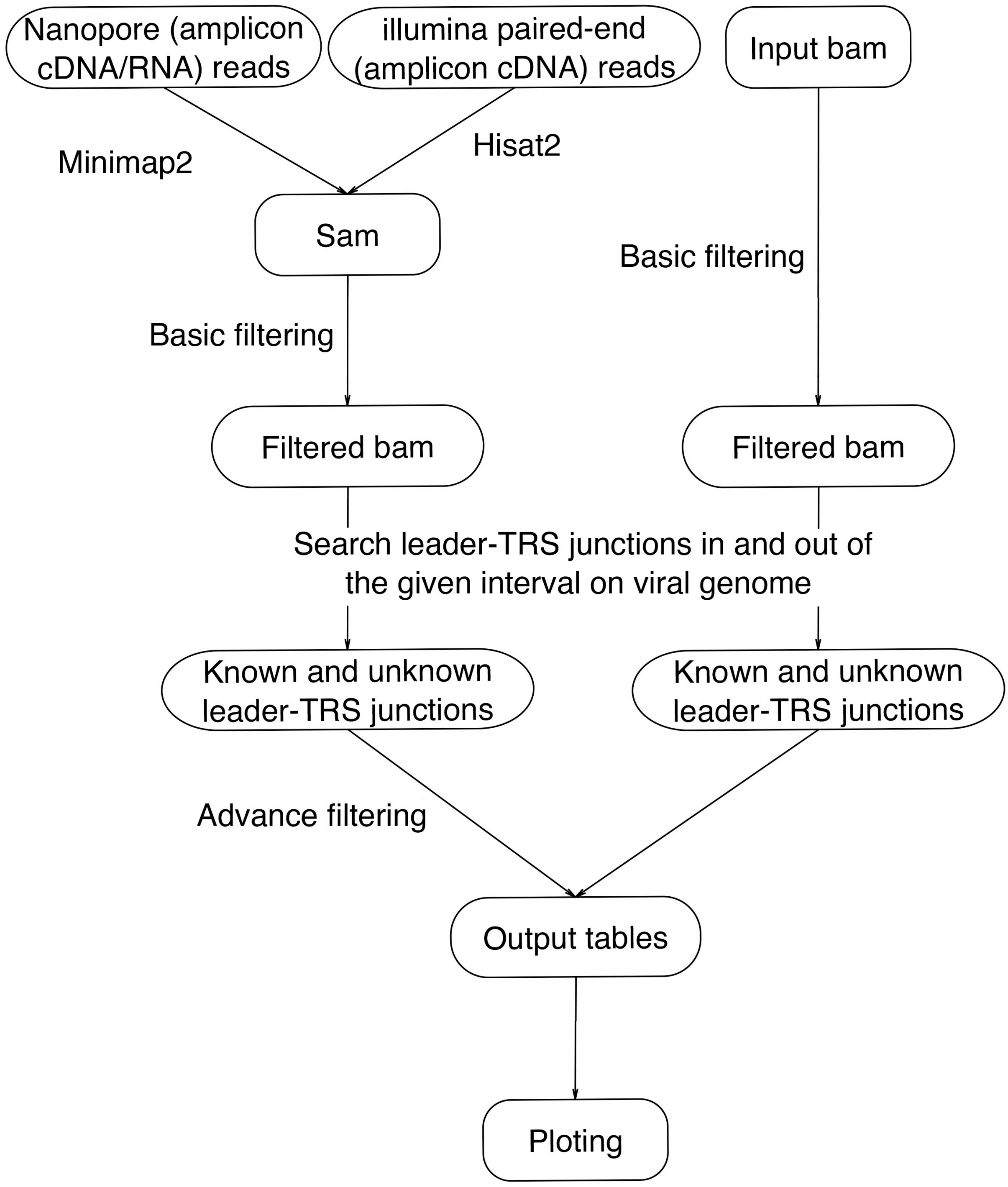

Supplementary Figure 1

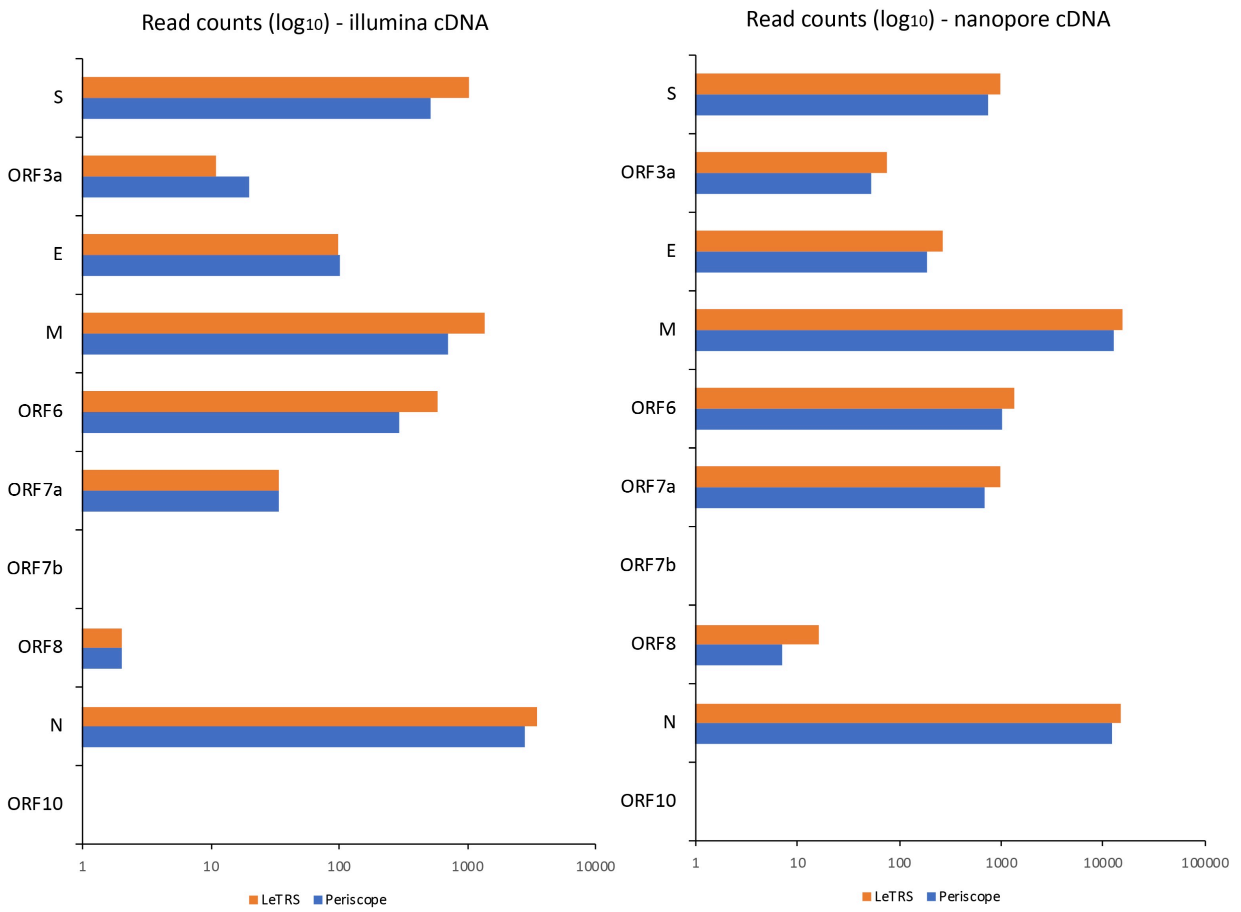

Supplementary Figure 2

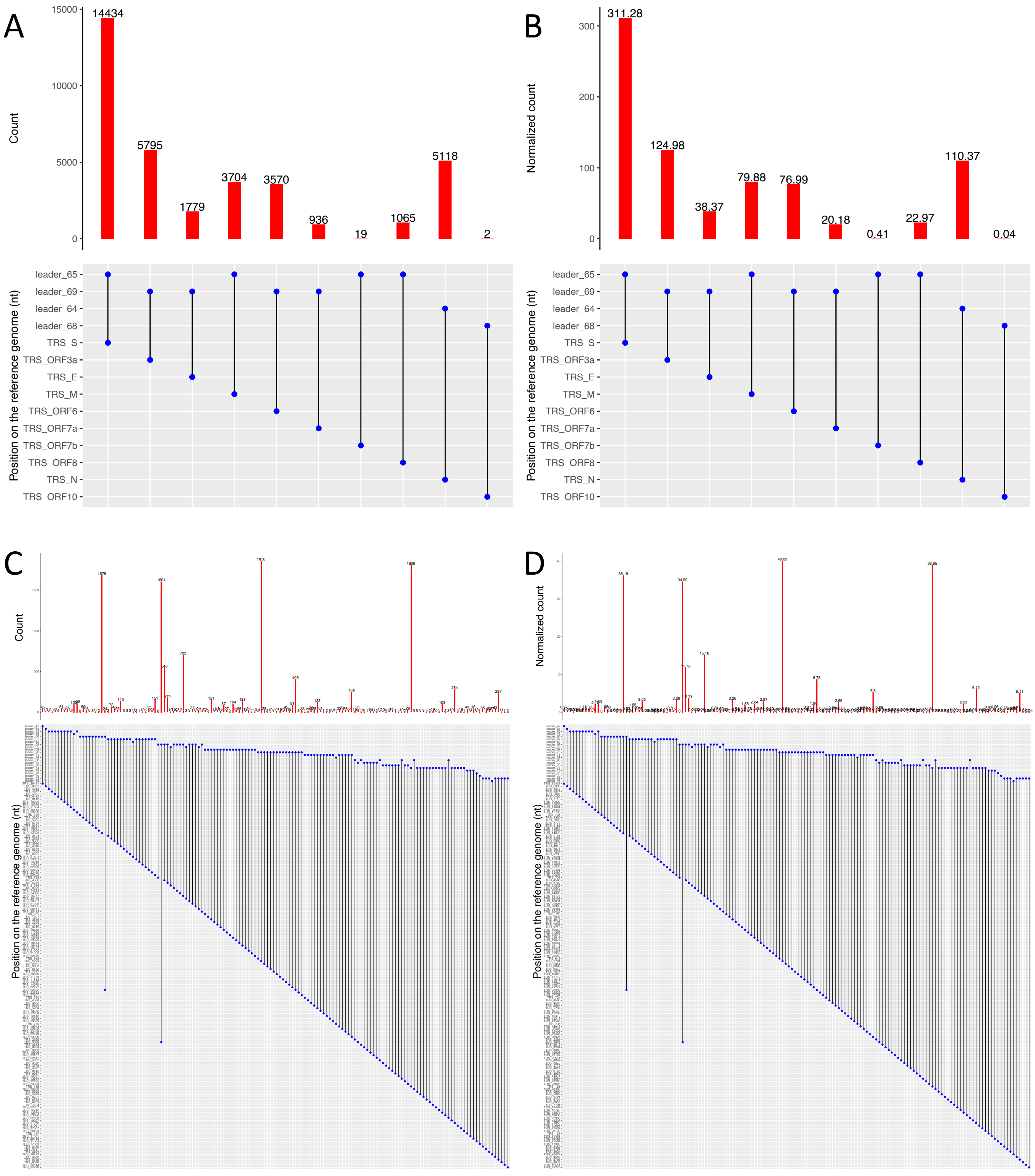

Supplementary Figure 3

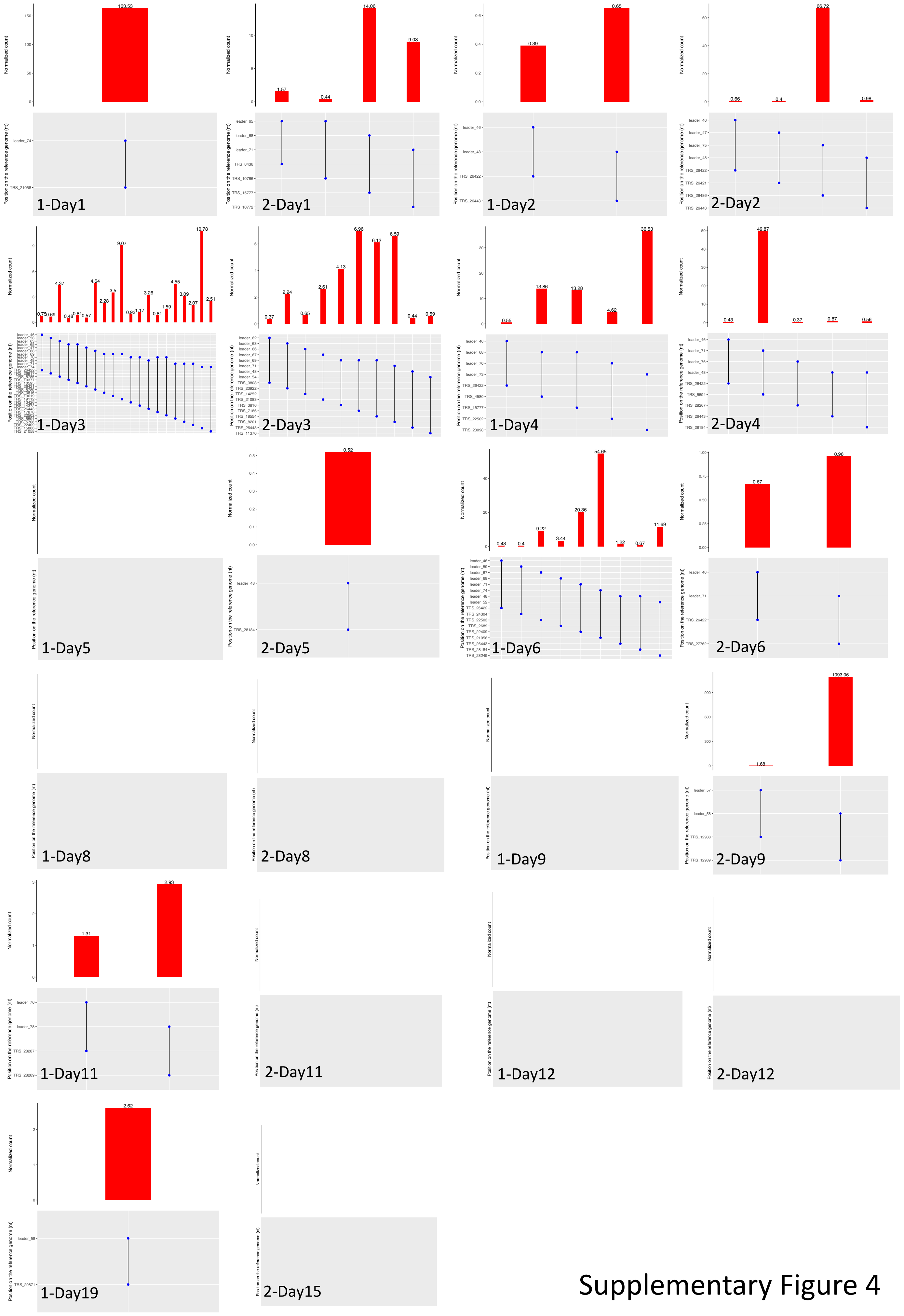

Supplementary Figure 4

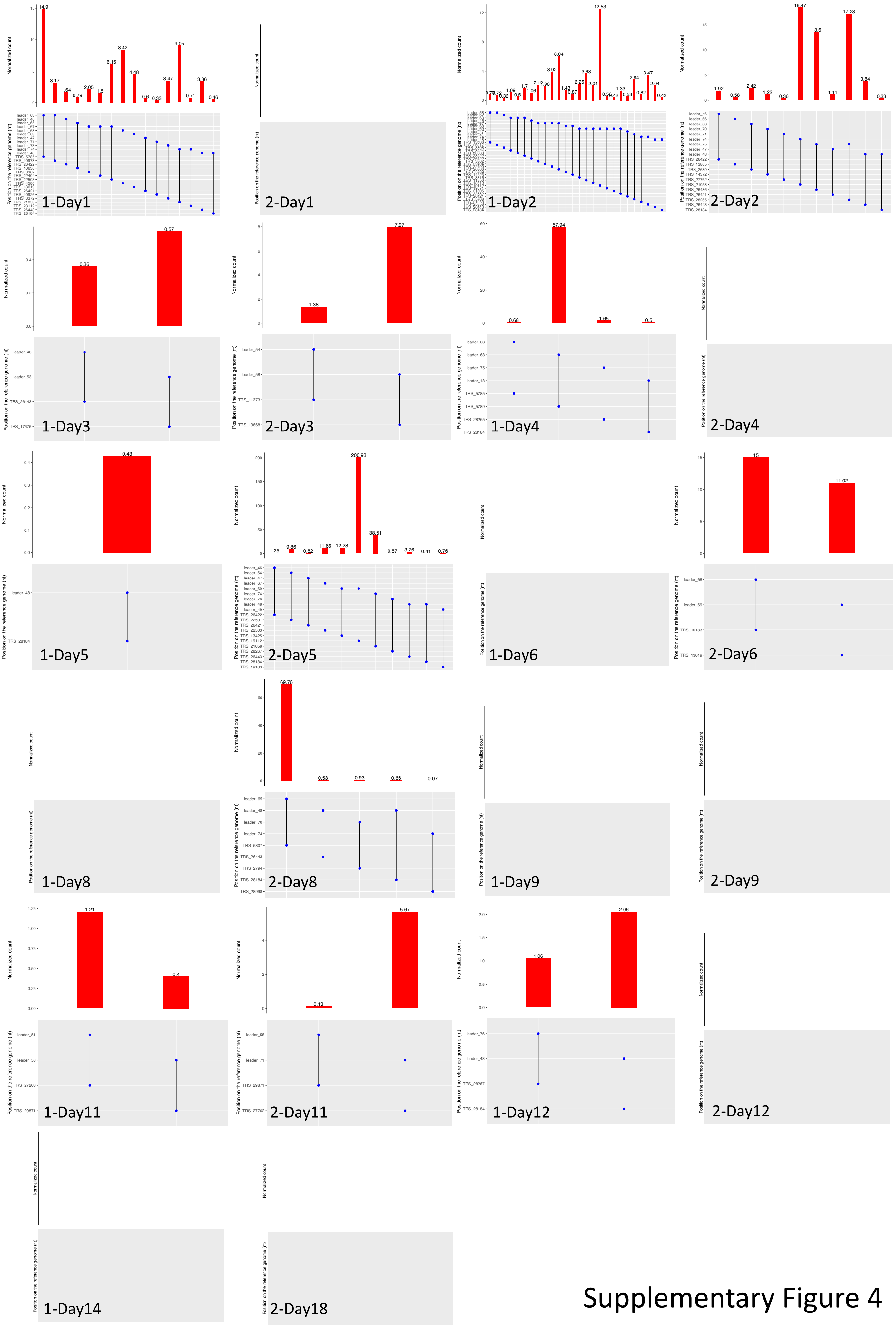

Supplementary Figure 4

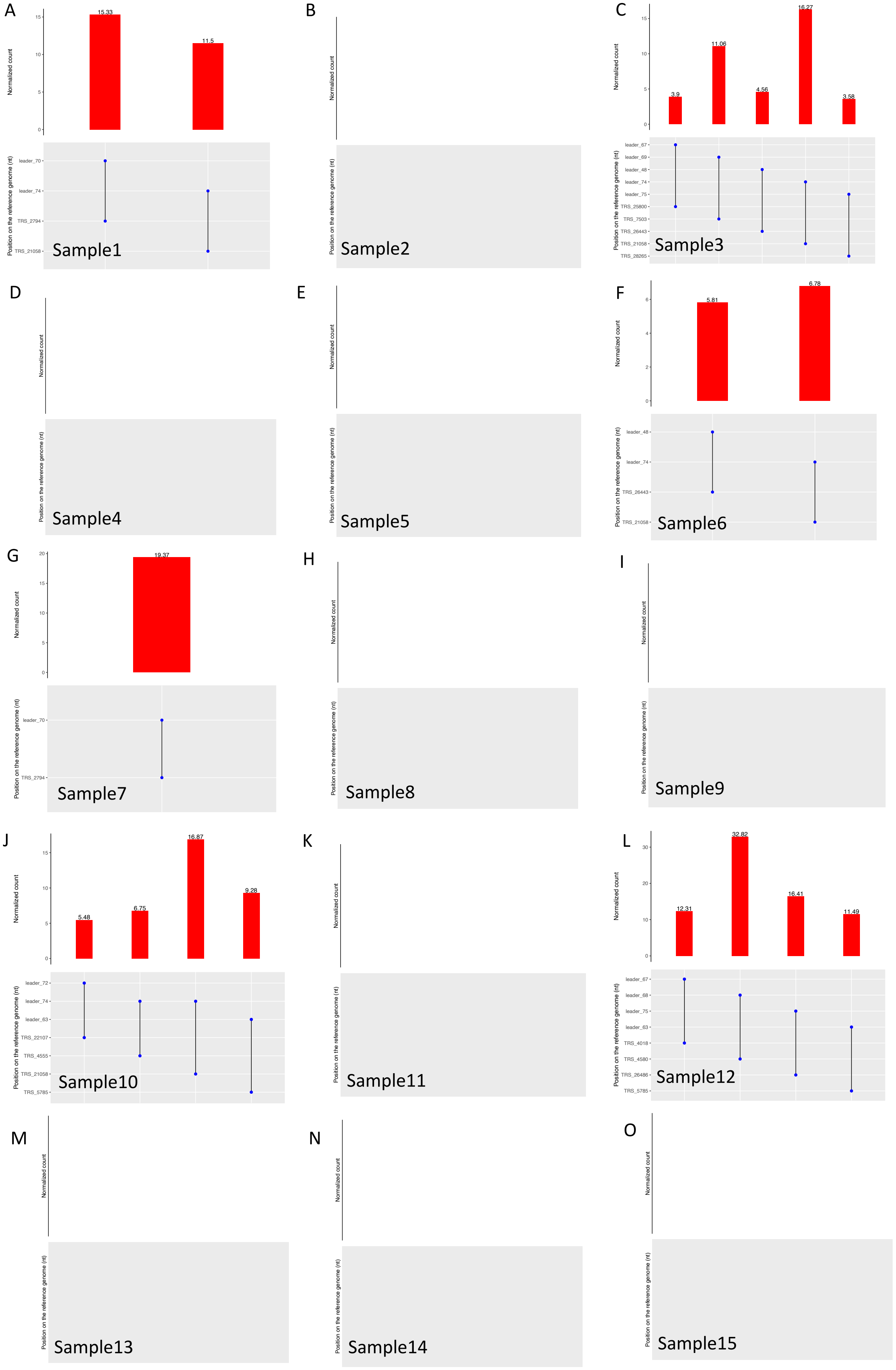

Supplementary Figure 5

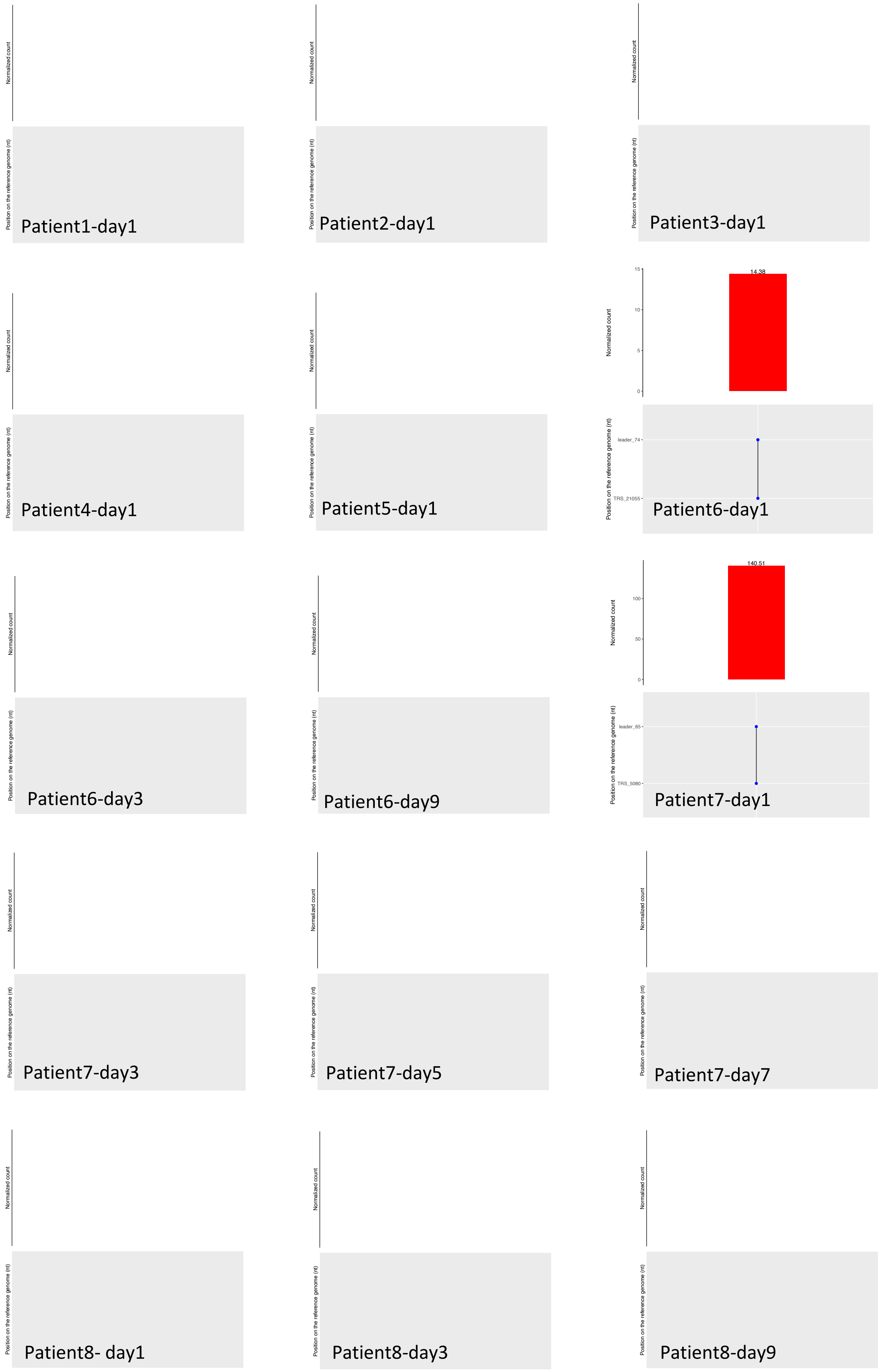

Supplementary Figure 6

Cover letter
Click here to access/download;Personal Cover;Author cover letter.pdf

Prof. Julian A. Hiscox

Chair in Infection and Global Health

Deputy Executive Dean

The University of Liverpool
Institute of Infection, Veterinary and Ecological Sciences
Liverpool Science Park IC2
146 Brownlow Hill
Liverpool
L3 5RF

Tel: +44 (0)7812238359.
Email: julian.hiscox@liverpool.ac.uk
Assistant: jhudson@liverpool.ac.uk

11 May 2021

Dear GigaScience,

Please find our submission 'Identification and quantification of SARS-CoV-2 leader subgenomic mRNA gene junctions in nasopharyngeal samples shows phasic transcription in animal models of COVID-19 and dysregulation at later time points that can also be identified in humans' for consideration for publication in your journal. We have developed a bioinformatics tool, LeTRS, that can interrogate any type of sequencing data and identify the unique features of SARS-CoV-2 subgenomic mRNAs (sgmRNAs). This is important because coronavirus sgmRNAs are only produced during infection. What tends to be forgotten is that their presence in a clinical sample can be indicate of active virus RNA synthesis and the presence of infected cells and not just the presence of virions. Also, the sgmRNAs are targets for the majority of nucleic acid diagnostics used in COVID-19. Therefore, their ratios can dramatically alter the interpretation of these results and the subsequent use of Ct values in building models of SARS-CoV-2 transmission.

The tool is computationally optimized and allows rapid analysis of very large datasets and presents a big step change as global sequencing databases can be used to track sgmRNAs and how these may change with the emergence of variants with different pathogenic properties. We think the tool can be used by many different research groups when analyzing their own local or global datasets. The tool can be very simply adapted to study sequencing data from other coronaviruses such as MERS-coronavirus that is continuously emerging in the Middle East.

Our manuscript isn't just about an informatics tool, we use it to study the pattern of sgmRNA synthesis over the natural course of infection in two non-human primate models that we recently have established to study COVID-19. Human challenge models are just starting, and unlikely to recapitulate disease, due to the infectious doses used. Thus, we provide unique insight that SARS-CoV-2 infection is phasic and that the pattern of sgmRNA abundance becomes dysregulated towards the end of infection. Analysis of similar longitudinal samples from humans also suggests the same pattern – again indicating that the interpretation of diagnostic Cts can be influenced by the time of infection and the presence of infected cells in the sample.

Yours sincerely,

Prof. Julian A. Hiscox.
