# Supplementary material for: Analysis of SARS-CoV-2 known and novel subgenomic mRNAs in cell culture, animal model, and clinical samples using LeTRS, a bioinformatic tool to identify unique sequence identifiers
Source: Gigascience. 2022 May 26;11:giac045. doi: 10.1093/gigascience/giac045 (PMC9154083; doi:10.1093/gigascience/giac045)
Supplement: giac045_GIGA-D-21-00142_Revision_2 [file giac045_giga-d-21-00142_revision_2.pdf]

GigaScience

Analysis of SARS-CoV-2 known and novel subgenomic mRNAs in cell culture, animal model and clinical samples using LeTRS, a bioinformatic tool to identify unique sequence identifiers.

--Manuscript Draft--

**Manuscript Number:**	GIGA-D-21-00142R2
**Full Title:**	Analysis of SARS-CoV-2 known and novel subgenomic mRNAs in cell culture, animal model and clinical samples using LeTRS, a bioinformatic tool to identify unique sequence identifiers.
**Article Type:**	Research
**Funding Information:**	U.S. Food and Drug Administration Medical Countermeasures (75F40120C00085) — Prof. Julian A. Hiscox<br>MRC ((MR/W005611/1) G2P-UK) — Prof. Julian A. Hiscox
**Abstract:**	SARS-CoV-2 has a complex strategy for the transcription of viral subgenomic mRNAs (sgmRNAs), which are targets for nucleic acid diagnostics. Each of these sgmRNAs has a unique 5’ sequence, the leader-transcriptional regulatory sequence gene junction (leader-TRS-junction), that can be identified using sequencing. High resolution sequencing has been used to investigate the biology of SARS-CoV-2 and the host response in cell culture and animal models and from clinical samples. LeTRS, a bioinformatics tool, was developed to identify leader-TRS-junctions and be used as a proxy to quantify sgmRNAs for understanding virus biology. LeTRS is readily adaptable for other coronaviruses such as Middle East respiratory syndrome coronavirus (MERS-CoV) or a future newly discovered coronavirus. LeTRS was tested on published datasets and novel clinical samples from patients and longitudinal samples from animal models with COVID-19. LeTRS identified known leader-TRS-junctions and identified putative novel sgmRNAs that were common across different mammalian species. This may be indicative of an evolutionary mechanism where plasticity in transcription generates novel open reading frames, that can then subject to selection pressure. The data indicated multi-phasic abundance of sgmRNAs in two different animal models. This recapitulates the relative sgmRNA abundance observed in cells at early points in infection, but not at late points. This pattern is reflected in some human nasopharyngeal samples, and therefore has implications for transmission models and nucleic acid-based diagnostics. LeTRS provides a quantitative measure of sgmRNA abundance from sequencing data. This can be used to assess the biology of SARS-CoV-2 (or other coronaviruses) in clinical and non-clinical samples, especially to evaluate different variants and medical countermeasures that may influence viral RNA synthesis.
**Corresponding Author:**	Julian Hiscox<br>University of Liverpool<br>Liverpool, UNITED KINGDOM
**Corresponding Author Secondary Information:**	
**Corresponding Author's Institution:**	University of Liverpool
**Corresponding Author's Secondary Institution:**	
**First Author:**	Xiaofeng Dong
**First Author Secondary Information:**	
**Order of Authors:**	Xiaofeng Dong
	Rebekah Penrice-Randal
	Hannah Goldswain
	Tessa Prince

Powered by Editorial Manager® and ProduXion Manager® from Aries Systems Corporation

	Nadine Randle
	Donavan-Banfield I'ah
	Francisco J Salguero
	Julia Tree
	Ecaterina Vamos
	Charlotte Nelson
	Jordan Clark
	Yan Ryan
	James P. Stewart
	Malcolm G. Semple
	John Kenneth Baillie
	Peter J. Openshaw
	Lance Turtle
	David A. Matthews
	Miles W. Carroll
	Alistair C. Darby
	Julian A. Hiscox
**Order of Authors Secondary Information:**	
**Response to Reviewers:**	Reviewer reports:<br>Reviewer #1: Comments: It is an important study. Except for a few minor points, the authors have addressed most of the reviewers' suggestions. This manuscript will be considered for acceptance after addressing the following minor suggestions:<br><br>1.The authors have compared the algorithm design, input, and output, and the counts of predicted sgmRNA across four tools. However, it would be nice if the authors could compare these tools' performances regarding prediction accuracy, F-measure, sensitivity, and specific scores. These will let the readers and potential users have a better sense of choosing a different tool for different purposes.<br><br>[We have added the prediction accuracy, F-measure, sensitivity, and specific scores, calculated based on simulated Illumina and Nanopore reads, in the Table 1.]<br><br>2.It is unclear what the red line means in Supplemental Figure 8-9.<br><br>[The red lines in Supplemental Figure 8 and 9 are for the normalized count of sgmRNA identified by LeTRS. We have moved this to Supplementary Table 12.]<br><br>3.On page 18, lines 364-370. The analysis and significance that the authors stated in that paragraph do not show the apparent trends in Supplemental Figure 9. Would the authors update the figure types to reflect the results of their statistical tests?<br><br>[We have updated the boxplots in Supplemental Figures 8 and 9. We used a paired samples one-sided Wilcoxon test that takes account the difference at each modification site of two compared sgmRNAs in different time points. A large amount of modification sites with differences resulted a low p-value even the trends in boxplots are not very large.]<br><br>4.On page 18, line 370. The author mentioned that "The abundance of most sgmRNAs decreased with time, and both of these factors could account for the frequency of methylation." Based on the context, it seems that the conclusion could not be derived. Because the methylation frequency is a ratio, then it may not correlate with the abundance of the sgmRNAs.

Powered by Editorial Manager® and ProduXion Manager® from Aries Systems Corporation

	[We have removed this sentence to reflect the reviewer's content.]<br><br>Reviewer #2: Happy with revisions, no further comments
**Additional Information:**	
**Question**	**Response**
Are you submitting this manuscript to a special series or article collection?	No
**Experimental design and statistics**<br><br>Full details of the experimental design and statistical methods used should be given in the Methods section, as detailed in our Minimum Standards Reporting Checklist. Information essential to interpreting the data presented should be made available in the figure legends.<br><br>Have you included all the information requested in your manuscript?	Yes
**Resources**<br><br>A description of all resources used, including antibodies, cell lines, animals and software tools, with enough information to allow them to be uniquely identified, should be included in the Methods section. Authors are strongly encouraged to cite Research Resource Identifiers (RRIDs) for antibodies, model organisms and tools, where possible.<br><br>Have you included the information requested as detailed in our Minimum Standards Reporting Checklist?	Yes
**Availability of data and materials**<br><br>All datasets and code on which the conclusions of the paper rely must be either included in your submission or deposited in publicly available repositories (where available and ethically appropriate), referencing such data using	Yes

a unique identifier in the references and in the “Availability of Data and Materials” section of your manuscript.<br><br>Have you have met the above requirement as detailed in our Minimum Standards Reporting Checklist?	

Powered by Editorial Manager® and ProduXion Manager® from Aries Systems Corporation

Manuscript

Click here to access/download;Manuscript;Leader_TRS_paper_revision_3rd_x

Analysis of SARS-CoV-2 known and novel subgenomic mRNAs in cell culture, animal model and clinical samples using LeTRS, a bioinformatic tool to identify unique sequence identifiers.

Xiaofeng Dong[1], Rebekah Penrice-Randal[1], Hannah Goldswain[1], Tessa Prince[1], Nadine Randle[1], I'ah Donovan-Banfield[1,2], Francisco J. Salguero[3], Julia Tree[3], Ecaterina Vamos[1], Charlotte Nelson[1], Jordan Clark[1], Yan Ryan[1], James P. Stewart[1], Malcolm G. Semple[1,2], J. Kenneth Baillie[4], Peter J. M. Openshaw[5], Lance Turtle[1,2], David A. Matthews[6], Miles W. Carroll[2,3], Alistair C. Darby[1] and Julian A. Hiscox[1,2,7].

[1]Institute of Infection, Veterinary and Ecological Sciences, University of Liverpool, UK.

[2]NIHR Health Protection Research Unit in Emerging and Zoonotic Infections, Liverpool, UK.

[3]UK-Health Security Agency, Salisbury, UK.

[4]The Roslin Institute, University of Edinburgh, UK.

[5]National Heart and Lung Institute, Imperial College London, UK.

[6]University of Bristol, UK.

[7]Infectious Diseases Horizontal Technology Centre (ID HTC), A*STAR, Singapore.

Corresponding author: julian.hiscox@liverpool.ac.uk

ORCID iDs:

Julian A Hiscox [0000-0002-6582-0275]; Rebekah Penrice-Randal [0000-0002-0653-2097]; Hannah Goldswain [0000-0003-4194-8714]; Tessa Prince [0000-0002-8796-2629]; Nadine Randle [0000-0002-3775-9585]; Francisco J Salguero [0000-0002-5315-3882]; Julia Tree [0000-

0002-1720-6764]; James P Stewart [0000-0002-8928-2037]; Malcolm G Semple [0000-0001-9700-0418]; John Kenneth Baillie [0000-0001-5258-793X]; Peter J Openshaw [0000-0002-7220-2555]; Lance Turtle [0000-0002-0778-1693]; David A Matthews [0000-0003-4611-8795]; Miles W Carroll [0000-0002-7026-7187]; Alistair C Darby [0000-0002-3786-6209]

Abstract

SARS-CoV-2 has a complex strategy for the transcription of viral subgenomic mRNAs (sgmRNAs), which are targets for nucleic acid diagnostics. Each of these sgmRNAs has a unique 5’ sequence, the leader-transcriptional regulatory sequence gene junction (leader-TRS-junction), that can be identified using sequencing. High resolution sequencing has been used to investigate the biology of SARS-CoV-2 and the host response in cell culture and animal models and from clinical samples. LeTRS, a bioinformatics tool, was developed to identify leader-TRS-junctions and be used as a proxy to quantify sgmRNAs for understanding virus biology. LeTRS is readily adaptable for other coronaviruses such as Middle East respiratory syndrome coronavirus (MERS-CoV) or a future newly discovered coronavirus. LeTRS was tested on published datasets and novel clinical samples from patients and longitudinal samples from animal models with COVID-19. LeTRS identified known leader-TRS-junctions and identified putative novel sgmRNAs that were common across different mammalian species. This may be indicative of an evolutionary mechanism where plasticity in transcription generates novel open reading frames, that can then subject to selection pressure. The data indicated multi-phasic abundance of sgmRNAs in two different animal models. This recapitulates the relative sgmRNA abundance observed in cells at early points in infection, but not at late points. This pattern is reflected in some human nasopharyngeal samples, and therefore has implications for transmission models and nucleic acid-based diagnostics. LeTRS provides a quantitative measure of sgmRNA abundance from sequencing data. This can be used to assess the biology of SARS-CoV-2 (or other coronaviruses) in clinical and non-clinical samples, especially to evaluate different variants and medical countermeasures that may influence viral RNA synthesis.

Importance

When infecting cells, SARS-CoV-2 not only replicates its genome but also makes molecules called subgenomic mRNAs (sgmRNAs) that are used as the template for many of the viral proteins, including the spike glycoprotein. The sgmRNAs can only be found in infected cells, and therefore their presence and ratio in a clinical sample is indicative that viral RNA synthesis has occurred, and infected cells are present. The sgmRNAs are targets for diagnostic assays. We have developed a rapid informatics methodology (LeTRS) to identify these unique molecules from multiple types of sequencing data generated in response to the COVID-19 pandemic. We used this pipeline to follow the pattern of sgmRNA abundance in nasopharyngeal samples taken from non-human primate models and clinical samples from humans. We identified putative novel sgmRNAs that may point to a potential new evolutionary mechanism in the virus. The data indicated that SARS-CoV-2 RNA synthesis (and by inference infection) may occur in waves, and this has implications for diagnostics and modelling of disease spread.

Introduction

Various sequencing approaches are used to characterise SARS-CoV-2 RNA synthesis in cell culture [1, 2], ex vivo models [3] and clinical samples. This can include nasopharyngeal swabs from patients with COVID-19 [4] to post-mortem samples from patients who died of severe disease [5]. Bioinformatic interrogation of this data can provide critical information on the biology of the virus. SARS-CoV-2 genomes are message sense, and the 5’ two thirds of the genome is translated and proteolytically cleaved into a variety of functional subunits, many of which are involved in the synthesis of viral RNA [6]. The remaining one third of the genome is expressed through a nested set of subgenomic mRNAs (sgmRNAs). These have common 5’ and 3’ ends with the coronavirus genome, including a leader sequence, and are thus co-terminal. Many studies have shown that the sgmRNA located towards the 3’ end of the genome, which encodes the nucleoprotein, generally has a higher abundance than those located immediately after the 1a/b region and the genome itself in infected cells [7, 8]. However, there is not necessarily a precise transcription gradient of the sgmRNAs. The 5’ leader sequence on the sgmRNAs is immediately abutted to a short sequence called a transcriptional regulatory sequence (TRS) that is involved in the control of sgmRNA synthesis [9, 10]. These TRSs are located along the genome and are proximal to the start codons of the open reading frames [11]. In the negative sense the TRSs are complementary to a short portion of the genomic leader sequence. The TRS is composed of a short core motif that is conserved and flanking sequences [9, 10, 12]. The core motif of the TRS in SARS-CoV-2 is ACGAAC.

The prevailing thought is that synthesis of sgmRNAs involves a discontinuous step during negative strand synthesis [13, 14]. A natural consequence of this is recombination resulting in insertions and deletions (indels) in the viral genome and the formation of defective viral RNAs. Thus, the identification of the leader/sgmRNA complexes by sequencing provides information on the abundance of the sgmRNAs and evidence that transcription has occurred in the tissue being analysed. In terms of clinical samples, if infected cells are present, then leader/sgmRNA 'fusion' sequence can be identified, and inferences made about active viral RNA synthesis from the relative abundance of the sgmRNAs. In the absence of published data from human challenge models, the kinetics of virus infection are unknown, and most studies will begin with detectable viral RNA on presentation of the patient with clinical symptoms. In general, models of infection of humans with SARS-CoV-2 assume an exponential increase in viral RNA synthesis followed by a decrease, as antibody levels increase [15].

To investigate the presence of SARS-CoV-2 sgmRNAs in clinical (and other) samples, a bioinformatics tool (LeTRS), was developed to analyse sequencing data from SARS-CoV-2 infections by identifying the unique leader-TRS gene junction site for each sgmRNA. The utility of this tool was demonstrated on cultured cells infected with SARS-CoV-2, nasopharyngeal samples from humans with COVID-19 and longitudinal analysis of nasopharyngeal samples from two non-human primate models infected with SARS-CoV-2. The tool is adaptable for other coronaviruses. The results have implications for virus biology, diagnostics and disease modelling.

Results

A tool, LeTRS (named after the leader-TRS fusion site), was developed to detect and quantify defined leader gene junctions of SARS-CoV-2 (and other coronaviruses) from multiple types of sequencing data. This was used to investigate SARS-CoV-2 sgmRNA synthesis in humans and non-human primate animal models. LeTRS was developed using the Perl programming language, including a main program for the identification of sgmRNAs and a script for plotting graphs of the results. The tool accepts FASTQ files derived from Illumina paired-end or Oxford Nanopore sequencing (amplicon or direct RNA), or BAM files produced by a splicing alignment method with a SARS-CoV-2 genome (Supplementary Figure 1). Note that SARS-CoV-2 sgmRNAs are not formed by splicing, but this is the apparent observation from sequencing data because of the discontinuous nature of transcription. By default, LeTRS analyses SARS-CoV-2 sequence data by using 10 known leader-TRS junctions and an NCBI reference genome (NC_045512.2) to identify leader dependent canonical sgmRNAs. However, given the potential heterogeneity in the leader-TRS region and potential novel (leader dependent noncanonical) sgmRNAs the user can also provide customised leader-TRS junctions and SARS-CoV-2 variants as a reference. As there is some heterogeneity in the leader-TRS sites, LeTRS was also designed to search for multiple features of sgmRNAs. This included the leader-TRS junction in a given interval, report on the 20 nucleotides at the 3’ end of the leader sequence, the TRS, translate the first predicted orf of the sgmRNA, and find the conserved ACGAAC sequences in the TRS. LeTRS can also be used to identify the sequencing reads with leader independent fusion sites that has been suggested to probably produce unknown ORFs yielding functional products [2]. The tool was designed to investigate very large data sets that are produced during sequencing of multiple samples.

Combinations of read alignments with the leader-TRS junction that are considered for identifying leader-TRS junction sites

Various approaches have been used to sequence the SARS-CoV-2 genome and in most cases, this would also include any sgmRNAs as they are 3’ co-terminal and share common sequence extending from the 3’ end. Methods such as ARTIC[16], MIDNIGHT[17] and RSLA[4] use primer sets to generate overlapping amplicons that span the entire genome, and also amplify sgmRNA. Included is a primer to the leader sequence, so that the unique 5’ end of these moieties are sequenced. Primer sets of ARTIC, MIDNIGHT and RSLA are generally formed of 2 pools. For the ARTIC method, at the time of the study, only the pool 1 included a forward primer located within the leader region (< 80 nts) of the SARS-CoV-2 genome (https://github.com/artic-network/artic-ncov2019/blob/master/primer_schemes/nCoV-2019/V3/nCoV-2019.primer.bed). Therefore, LeTRS was designed with a function to analyse reads in the primer pool 1, pool 2 or both pools. Unbiased sequencing can also be used in methodologies to identify SARS-CoV-2 sequence. Data in the GISAID database have been generated by Oxford Nanopore (minority) or Illumina (majority) based approaches. These can give different types of sequencing reads derived from the sgmRNAs that can be mapped back on the reference SARS-CoV-2 genome by splicing alignment (Figure 1A). For example, there are several different types of reads that can be derived from mapping Illumina-based amplicon sequencing onto the reference viral genome (Figure 1B and 1C). During the PCR stage, the extension time allows the leader-TRS region on the sgmRNAs to be PCR-amplified by the forward primer and the reverse primer before and after leader-TRS junction in different primer sets, respectively. If the amplicon had a length shorter than the

Illumina read length (usually 100-250 nts), both the forward and reverse primers would be detected at the ends of each paired read (Figure 1B pink lines). If the amplicon was longer than the Illumina read length, primer sequence would be only found at one end of each paired read (Figure 1B green and brown lines), with the possibility of one of the paired reads having a fusion site. The extension stage could also proceed with a single primer using cDNA derived from the sgmRNA as a template. This type of PCR product has a very low amplification efficiency, but theoretically could also generate the same Illumina paired-end read with a single primer sequence at one end (Figure 1C). These paired-end reads could include the full length of the leader sequence but might not reach the 3' end of the sgmRNA, because of the limitation of Illumina sequencing length and extension time (Figure 1C). Also, unless there are cryptic TRSs located towards the 3' end of the genome, all sgmRNAs would be expected to be larger than the Illumina sequencing length.

In contrast, the different types of read alignment in the Nanopore based amplicon are simpler to assign. The longer reads that tend to be generated by Nanopore sequencing (depending on optimisation) enable the capture of full-length sequences of all amplicons. Provided the leader sequence is included as a forward primer most of the reads spanning the leader-TRS junction would contain the forward and reverse primer sequences at both ends (Figure 1D pink lines). If the extension time allowed, single primer PCR amplification could take the Nanopore amplicon sequencing reads to both the 3' and 5' ends of the sgmRNAs, and these types of reads would only have a primer sequence at one end (Figure 1D brown lines). In the Nanopore direct RNA

sequencing (dRNAseq) approach, the full-length sgmRNA could be sequenced and mapped entirely on the leader and TRS-orf regions (Figure 1E).

Evaluation of LeTRS on SARS-CoV-2 infection in cell culture.

In order to assess the ability of LeTRS to identify the leader-TRS junctions from sequencing information, a total RNA sample was prepared at 72 hours post-infection (hpi) from hACE2-A549 cells infected with SARS-CoV-2 (a lineage B isolate). This RNA was sequenced using an amplicon-based approach (ARTIC) with either Nanopore (ARTIC-Nanopore) or Illumina (ARTIC-Illumina), or alternatively by a Nanopore dRNAseq approach [18]. The ARTIC-Nanopore (Figure 2A, Supplementary Table 1) and ARTIC-Illumina (Figure 2B, Supplementary Table 2) sequencing data were evaluated with LeTRS by setting the analysis to both primers pools. For dRNAseq (Figure 2C, Supplementary Table 3), data was evaluated with LeTRS using the default setting. All the major known leader-TRS gene junctions were identified by these sequencing methods. Analysis demonstrated an expected pattern of abundance of the leader-TRS gene junctions with the leader-TRS nucleoprotein gene junction being most abundant (Figure 2A, B and C; Supplementary Tables 1, 2 and 3). Novel low abundance leader-TRS gene junctions were also identified (Figure 2A, B and C; Supplementary Tables 1, 2 and 3). These known and novel leader-TRS junctions were also known as leader dependent canonical and noncanonical fusions, respectively [2]. LeTRS also has a function to identify leader independent long-distance fusion (>5,000 nt) and local joining yielding a deletion between proximal sites (20-5,000 nt distance) in the sequencing reads. The leader independent fusions (coverage >= 2) are shown in Supplementary Tables 1, 2 and 3. Indel sequencing errors are frequent (defined as less than 20 nucleotides), especially in Nanopore

sequencing data, and therefore it is difficult to find precise fusion (apparent splicing) sites in this case [19]. However, some of the novel leader-TRS junctions (noncanonical fusions) and leader independent fusions in the test sample were supported by all three sequencing methods (Supplementary Figure 2) with similar fusion sites. Many local fusions/deletions within the orf3, E, M, orf6, orf7a, orf7b, orf8 and N genes were identified (Supplementary Figure 2 G, H and I) confirmed previous findings [2, 20], and indicates these are common events. Some of the novel leader-TRS junctions (noncanonical fusions) and leader independent fusions may be the result of sequencing or reverse transcription errors, especially those with low abundance (Supplementary Tables 1, 2 and 3; Supplementary Figure 2). The ARTIC-Illumina approach identified fewer novel leader-TRS junctions (noncanonical fusions) and leader independent fusions than the other two sequencing methodologies, probably due to lower sequencing coverage (Supplementary Tables 1, 2 and 3).

For ARTIC approaches, LeTRS was designed to analyse reads in the primers pool 1, pool 2 or both pools. Only the ARTIC pool 1 included a forward primer that is located within the leader region (< 80 nts) of the SARS-CoV-2 genome. The leader-TRS regions of sgmRNAs can be PCR-amplified by both forward and reverse primers in ARTIC pool 1, but only reverse primers in ARTIC pool 2. The read counts evaluated by LeTRS in both ARTIC-Nanopore and ARTIC-Illumina were compared in the test data for pool 1 and 2, and found only very few reads/read pairs contained the correct primers (Supplementary Table 4 and 5), suggesting the primers in ARTIC pool 2 generally do not contribute to sequencing of leader-TRS regions.

Comparison with other informatic tools that can identify leader TRS gene junctions.

Other tools have been developed to identify sgmRNAs from ARTIC-Illumina and ARTIC-Nanopore sequencing data, such as Periscope (v0.1.0) [21], SARS-CoV-2-leader (https://github.com/hyeshik/sars-cov-2-transcriptome) [18] and SuPER (https://github.com/ncbi/SuPER) [22]. These tools were compared with LeTRS as shown in Table 1. LeTRS and Periscope used the FASTQ files as input, while SARS-CoV-2-leader and SuPER required SAM files from a user generated alignment. Searching fusion site and sequences tag in the sequencing reads are two major methods used. LeTRS and SuPER analysed the fusion/splicing information in sequence reads achieved by an alignment program and also take account of the conserved ACGAAC sequences in the TRS. Periscope and SARS-CoV-2-leader are based on searching for a short tag sequence in the leader from sequencing reads. However, searching for a short tag sequence in the leader with the high error rate associated with Nanopore data can be challenging. LeTRS and Periscope use primer information to differentiate reads mapping to amplicons to reads mapping from original virus genomes. Besides Periscope, output from dRNAseq is supported by the other available tools. Illumina sequencing reads are usually short (< 250 bases), paired and sequenced from both ends. If both reads in a single pair contain a fusion site this will be counted twice by the other three tools (Figure 1B green and pink). However, if only one of the reads in the pair contains a fusion site it will be counted once (Figure 1B brown). This leads to biased counting. LeTRS takes this into account by treating each read pair as a single event. LeTRS also has a unique function to analyse reads in the primers pool 1, pool 2 or both pools from ARTIC based sequencing (Table 1). Accuracy, sensitivity, specificity and the F-measure score were calculated with simulated Illumina and Nanopore sequencing reads. All of these tools

performed better for analysing the simulated Illumina sequencing reads compared to the simulated Nanopore sequencing reads (Table 1). LeTRS showed greater sensitivity and F-measure score than the other tools for processing the simulated Nanopore sequencing reads (Table 1).

To compare the performance to LeTRS, these three tools were evaluated using the hACE2-A549 cell culture sample sequenced by ARTIC-Nanopore, ARTIC-Illumina and Nanopore dRNAseq. Using the ARTIC-Nanopore sequencing data, all the tools reported a similar number of read counts for the 10 known sgmRNAs (Supplementary Figure 3A). LeTRS showed fewer counts for the ARTIC-Illumina than the other three tools because of considering read pairs (Supplementary Figure 3B). Interestingly, Periscope also identified fewer nucleoprotein sgmRNAs with the ARTIC-Illumina sequencing data (Supplementary Figure 3B). As of writing, Periscope does not yet support Nanopore dRNAseq data, therefore LeTRS, SARS-CoV-2-leader and SuPER were compared. LeTRS and SARS-CoV-2-leader generally identified more dRNAseq reads than SuPER, especially for the nucleoprotein sgmRNA (Supplementary Figure 3C). Finally, the ratio of read counts with the 10 known sgmRNA (S:orf3:E:M:orf6:orf7a:orf7b:orf8:N:orf10) were compared, and the three tools showed almost an identical ratio when analysing data from the same sequencing methods (Supplementary Figure 3D). ARTIC-Nanopore and Nanopore dRNAseq resulted in a higher ratio of read counts with M and orf7a respectively (Supplementary Figure 3D). The read counts ratio of sgmRNAs mapping to spike was much lower with dRNAseq approaches (Supplementary Figure 3D).

Normalisation of read counts for sgmRNA

Normalisation of read counts has been widely used for RNAseq in the comparison of gene expression level across samples [23]. The normalisation is generally based on the ratio of reads mapped on the gene to the total number of reads in that sample. These tools use this algorithm for the normalisation of read counts in searching for sgmRNA [21, 24]. LeTRS also incorporated a method to differentiate the total reads mapped (i) or whether the reads have forward primer only (ii), reverse primer only (iii), both primers (iv) or at least one primer (v) present. This is achieved by (i) the total number of reads mapped on the SARS-CoV-2 genome for the number of reads of leader-TRS fusion site as the numerator; (ii) the total number of reads with forward primers only for the number of reads of leader-TRS fusion site with forward primers only as the numerator; (iii) the total number of reads with reverse primers only for the number of reads of leader-TRS fusion site with reverse primers only as the numerator; (iv) the total number of reads with both primers for the number of reads of leader-TRS fusion site with both as the numerator and (v) the total number of reads with at least one primer on one side for the number of reads of leader-TRS fusion site with at least one primer on as the numerator (notes in Supplementary Tables 1, 2 and 3).

Because LeTRS considers the primers; pool 1, pool 2 or both pools, normalisation could be observed in ARTIC pool 1 only to minimise the effect from ARTIC pool 2 since primers in ARTIC pool 2 are almost not involved the sequencing of leader-TRS regions (as described above). For the same RNA derived from the hACE2-A549 cell culture sample sequenced by ARTIC-Nanopore, ARTIC-Illumina or Nanopore dRNAseq approaches, the normalised counts for the known sgmRNAs were much smaller with the pool 1 of PCR based amplicon methods (ARTIC-Nanopore

and ARTIC-Illumina) than the Nanopore dRNAseq approach (Figure 3A and C for the reads with at least one primer sequence; Supplementary Tables 3, 4 and 5). However, the normalised counts with ARTIC-Nanopore and ARTIC-Illumina showed the same ratio of known sgmRNA as the Nanopore dRNAseq approach, except for sgmRNAs mapping to S and orf7a (Figure 3B and D for the reads with at least a primer sequence). PCR based approaches increases the value of the denominator and reduced the normalised count, because a full length of sgmRNA was counted once with the dRNAseq approach compared to many times with the amplicon approaches. ARTIC-Illumina had fewer normalised counts than ARTIC-Nanopore probably due to the sequencing bias of Illumina during PCR [25]. Thus, if the samples were sequenced with the same methodology they were comparable. With a PCR based method a normalised count should be used to show the relative difference between samples.

LeTRS identified many reads with only one primer (one-sided amplification) with the PCR based amplicon methods (Supplementary Tables 4 and 5). The ratio of reads with either forward and/or reverse primers were compared for each sgmRNA to the overall ratios of reads, with forward primers only or reverse primers only, both primers in all mapped reads of pool 1 and pool 2 and the mapped reads with any fusion sites of pool 1 and pool 2. This indicated that abundant reads were identified with a single pattern and these were similar to reads mapping to sgmRNAs, suggesting a one sided amplification is associated with amplicon-based approaches (Supplementary Figure 4).

Analysis of sequencing data from longitudinal nasopharyngeal samples taken from two non-human primate models of COVID-19 indicated multi-phasic sgmRNA synthesis and novel sgmRNAs.

Part of the difficulty of studying SARS-CoV-2 and the disease COVID-19 is establishing the sequence of events from the start of infection. Most samples from humans are from nasopharyngeal aspirates taken when clinical symptoms develop. This tends to be 5 to 6 days post-exposure. In the absence of a human challenge model, animal models can be used to study the kinetics of SARS-CoV-2[26, 27]. Two separate non-human primate (NHP) models, cynomolgus and rhesus macaques, were established for the study of SARS-CoV-2 that mirrored disease in most humans[26]. To study the pattern of sgmRNA synthesis over the course of infection, nasopharyngeal samples were sequentially gathered daily from 1 dpi up to 18 dpi from the two NHP models. RNA was purified from these longitudinal samples as well as the inoculum virus and viral RNA sequenced using ARTIC-Illumina.

As expected, analysis of the sequence data using LeTRS from the inoculum used to infect the NHPs indicated that leader gene junctions could be identified, but these did not follow the pattern of abundance of leader TRS-gene junctions found in infected cells in culture, where the leader TRS nucleoprotein gene junction was most abundant (Supplementary Figure 5). The inoculum would be expected to contain mostly genomic RNA found in virions. In contrast, analysis of the longitudinal sequencing data from nasopharyngeal aspirates from the NHP model using LeTRS identified leader TRS-gene junctions associated with the major sgmRNAs (Figure 4, Supplementary Table 7) as well as novel leader-TRS gene junction sites (Supplementary Figures

6 and 7). Analysing the abundance of the leader-TRS-gene junctions for both model species over the course of infection revealed a phasic nature of sgmRNA synthesis in pool 1 to minimise the effect from ARTIC pool 2 (Figure 4). The leader-TRS nucleoprotein gene junction was the most abundant, and there was a phasic pattern of potential sgmRNA abundance identified with the ARTIC-Illumina method (Figure 4). For both species, viral load and hence sgmRNA abundance had decreased by 8 and 9 dpi.

Analysis of leader-TRS-gene junction in human samples revealed expected and aberrant abundances of sgmRNAs

To investigate the pattern of leader-TRS-gene junction abundance during infection of SARS-CoV-2 in humans, nasopharyngeal swabs from patients with COVID-19 were sequenced by ARTIC-Illumina (using samples from COG-UK) (N=15 patients) (Figure 5, Supplementary Table 8) or by ARTIC-Nanopore (using samples from ISARIC-4C) (N=15 patients) (Figure 6, Supplementary Tables 9 and 10). In several samples, leader-TRS-gene junctions were identified and followed an expected pattern, with the nucleoprotein gene junction being the most abundant (e.g., Sample 1 in Figures 5A and B, Patient 2 day1 in Figure 6A and B). However, in several of the samples there was very large representation of single leader-TRS-gene junction (e.g., Sample 4 and 5 in Figures 5A and B). These tended to map to the nucleoprotein gene (Sample 5, 8 and 13 Figures 5A and B). The heterogeneity in abundance of leader-TRS-gene junctions was reminiscent of that from the NHP study with a defined and expected pattern near the start of infection but then becoming phasic. The samples gathered under ISARIC-4C were from hospitalised patients and permitted analysis in relation to reported date of symptom onset and sequential sampling. In general, the

data indicated that the first sample on admission to hospital contained an abundance of leader-TRS-gene junctions which resembled the pattern seen in infected cells (Patient 6 day 1 and day 9 in Figures 6A and B). However, with further days post-sample, e.g. (Patient 7 day 7 Figures 6A and B), the leader-TRS nucleoprotein gene junction was the most abundant and far exceeded any other detectable species. The abundance of leader-TRS nucleoprotein gene junction in the patients at a later stage of infection followed that observed in the NHP model (Figure 4).

Analysis of sequencing data from a previously published study investigating SARS-CoV-2 RNA in samples from patients

Recent research detected sgmRNAs mapping to E, ORF7a and N in swabs up to 14 days in one patient and ORF7a and N in another patient up to 17 days after first detection by using a high-throughput amplicon sequencing method known as Ion AmpliSeq Coronavirus Research Panel on an Ion S5 XL genetic sequencer. The authors concluded these sgmRNAs may be present for a significant time after active infection due to nuclease resistance and protection by cellular membranes [24]. The sequencing data from this study was reanalysed using LeTRS, and confirmed the finding of sgmRNAs in late infection from the two patients (Supplementary Table 11). Apart from nuclease resistance and protection by cellular membranes, a phasic pattern of sgmRNA synthesis may also contribute to the presence of sgmRNAs at later time points.

Analysis of sgmRNA modification in longitudinal samples in cell culture.

N6-methyladenosine (6mA) is a widely observed modification on cellular RNA, and 5-methylcytosine methylation (5mC) has also been reported on viral RNAs [18]. Methylation of

SARS_CoV-2 RNA was examined using sequencing data from the Nanopore direct RNA seq approach. Total RNA was purified at 6, 12 and 24 hpi from cells infected with SARS-CoV-2. The total RNA was sequenced and reads mapping to sgmRNAs were extracted with LeTRS for 6mA and 5mC examination. Almost all 10 observed sgmRNAs showed the same number of modification sites of 6mA and 5mC at 6, 12 and 24 hpi (Supplementary Table 12). Modification with 5mC was more abundant than 6mA in all 10 known sgmRNAs (Supplementary Table 12). There were differences in abundance of some sgmRNAs especially the M and N subgenomic mRNAs (Supplementary Table 12). However, there did not appear to be a relationship between number of methylation sites and the abundance of a particular sgmRNA (Supplementary Table 12).

To further evaluate the relationship between time post-infection and modification by methylation, a paired samples one-sided Wilcoxon test was used. This analysis suggested that the 5mC modification fraction at 24 hpi was significantly less than compared to modification at 6 and 12 hpi (p-value < 0.05), except for ORF7b and ORF10 (Supplementary Figures 8 and 9; Supplementary Table 13). Modification with 6mA at 24 hpi was also significantly less than at 6 hpi, but not at 12 hpi (p-value < 0.05) in S, ORF3a, E, M, ORF6, ORF7a, ORF8 and N.

Common properties/features of novel leader-TRS gene junctions and sgmRNAs

The sequencing data from cells infected in culture (Supplementary Table 14), animal models and clinical samples from humans indicated the presence of novel leader-TRS gene junctions. Their detection generally increased with depth of coverage. Coronavirus replication and transcription is promiscuous, and recombination is a natural result of this, resulting in indels and potential

gene rearrangements. Many of these novel leader-TRS junctions were centred around the known gene orf but out of the search interval. These types of leader-TRS-gene junctions could be only found with spike, membrane, ORF6, ORF7b and nucleocapsid orfs, in which the membrane orf was the most common (Figure 7A). To define what might be genuine novel leader-TRS-gene junctions, these were compared across the data in all ARTIC-Illumina data (Figure 7B, Supplementary Table 15). Five novel leader-TRS-gene junctions were identified that were common to all the data, and the majority of these were present immediately 5’ of the membrane orf). The novel leader-TRS-gene junctions from LeTRS (Figure 7C) showed a similar distribution as a previous study, although this study did not detail the precise location [28].

Discussion

Coronavirus sgmRNAs are only synthesised during infection of cells and therefore their presence in sequence data can be indicative of active viral RNA synthesis. The abundance of the sgmRNAs in infected cells should follow a general pattern where the sgmRNA encoding the nucleoprotein is the most abundant. Identification and quantification of the unique leader-TRS-gene junctions for each sgmRNA can be used as a proxy for their abundance.

LeTRS was developed to interrogate sequencing datasets to identify the leader-TRS-gene junctions present at the 5' end of the sgmRNAs. LeTRS was first evaluated and validated on cell culture data from published datasets [2, 16] and from a cell culture experiment as part of this study and then used in an analysis of nasopharyngeal samples from NHP and human clinical samples. The results showed that the positions of the leader-TRS junction sites with peak read counts were the same as the given reference positions. The exception was at the leader-TRS-gene junction for orf7b in the Nanopore sequencing. The normalised count results confirmed the reads spanning the junctions showed that the leader-TRS nucleoprotein gene junction was the most abundant, and orf7b and orf10 were the most infrequent in line with other data [2, 24]. Several low abundant leader-TRS junctions were identified in all of the datasets (Supplementary Figure 2) with the implication these were either from potential lower abundant novel sgmRNAs or represented known sgmRNAs, but with different leader-TRS junctions. Likewise, at low frequency these could represent an aberrant viral transcription, perhaps as a mechanism to generate new orfs for selection or these could be artefacts of the different sequencing processes (Figure 2). Traditionally, such sgmRNAs have been first identified in coronaviruses by either

northern blot and/or metabolic labelling [8] and sequencing approaches are likely to be more sensitive giving the amplification steps involved. Several other groups have identified novel leader-TRS-gene junctions and potential sgmRNAs for other coronaviruses, including avian infectious bronchitis virus [29]. The best way of validating potential novel sgmRNAs would be through matching proteomic data to confirm genuine ORFs [1]. Analysis of several published sequencing datasets identified novel viral RNA molecules that the authors suggested were sgmRNAs containing only the 5' region of orf1a [30]. Such species are likely to be defective RNAs, that act as templates for replication, rather than sgmRNAs. Interestingly, at later time points post-infection in cell culture, potential novel sgmRNAs were found to be generated non-specifically [30]. This potentially ties in with a disconnect of leader-TRS-gene junctions observed in our study both *in vivo* from the nasopharyngeal samples from latter time points in the NHP models and in humans. This is also shown in published data from SARS-CoV-2 infections in cell culture gathered at later time points compared to earlier time points [2, 16].

Advanced filtering can improve the confidence of the identified leader-TRS junction from sequencing data. Amplicon sequencing provided a unique opportunity to filter the sequencing reads. The reads spanning the junctions with the correct forward primer, reverse primer or both primer sequences at the ends of reads proved the known/novel sgmRNA existing in tested ARTIC-Illumina and ARTIC-Nanopore amplicon sequencing data (Supplementary Tables 1 and 2). For Illumina sequencing, the same junction on paired reads with at least one primer provided extra evidence for leader-TRS identification. Some reads were identified that did not have primer sequences and these were likely to be erroneously mapped, from template sgmRNA or low-

quality sequence. These were present at very low abundance compared to authentically mapped reads (Supplementary Tables 1 and 2). The Nanopore dRNAseq approach had the potential to generate full-length mRNA sequences. The polyA sequences and leader-TRS junctions in the reads can be good signals to prove the full-length sgmRNA in the test data (Supplementary Table 3). Currently, LeTRS is the only tool to consider paired-end Illumina data and primer pools, and therefore is suited for interrogating paired-end Illumina data and providing data from amplicon sequencing information from either primer pools.

In terms of clinical samples (typically nasopharyngeal swabs), the presence of sgmRNAs will generally be due to the presence of infected cells. This has been seen as indicative of active viral RNA synthesis at the time of sampling[5, 31, 32], although these have also been postulated to be present through resistant structures after infection has finished [33]. Analysis of inoculum indicated that leader-TRS-gene junctions could be identified (Supplementary Figure 5) but that these were not in the same ratio as found in cells infected in culture (e.g., Figure 2A, B and 2C). Thus, if the abundance of leader-TRS-gene junctions follows an expected pattern of the leader-TRS nucleoprotein gene junction being the most abundant followed by a general gradient in sequence data from nasopharyngeal samples, then this may be indicative of an active infection – and the presence of infected cells in a sample.

In the absence of a human challenge model, NHP models that closely resemble COVID-19 disease in humans can be used to study SARS-CoV-2 infection from a very defined initial exposure. RNA was sequenced from longitudinal nasopharyngeal samples from two NHP models, rhesus and

cynomolgus macaques [26]. LeTRS was used to identify the abundance of the leader-TRS-gene junctions in this data. The analysis indicated a phasic pattern of sgmRNA synthesis with a large drop off after 8 or 9 dpi in both NHP models. This phasic pattern may be explained by an initial synchronous infection of respiratory epithelial cells followed by cell death. Released virus then goes on to infect new epithelial cells, with virus infection increasing exponentially in waves but becoming asynchronous. The decline in sgmRNA from 8 or 9 dpi overlaps with IgG seroconversion and humoral immunity in both species [26], and follows similar kinetics to serology profiles measured in patients with COVID-19.

The identification of sgmRNAs in nasopharyngeal samples and their kinetics has implications for nucleic acid-based diagnostics (many of which have three targets, one in the orf1a/b region and two which are shared between the genome and sgmRNAs – the nucleoprotein and the spike genes). The phasic nature of leader-TRS-gene junctions in the longitudinal samples, and by implication sgmRNAs, and overt abundance of the leader-TRS nucleoprotein gene junction found in many of the human samples, suggests that it may not be possible to precisely identify where in infection an individual is based on the abundance of sgmRNAs. Likewise, assuming equivalency between the targets, if the nucleoprotein target is found to be more abundant than the spike target than the genomic target, then this would suggest infected cells are present in the sample. Decreases in Ct values associated with emerging variants could equally be explained by sloughed cells being present in a nasopharyngeal sample as well as by increases in the amount of virions/viral load. Therefore, we would caution that a decrease in Ct associated with RT-qPCR based assays may not just be reflective of higher viral loads but also may be indicative of more

infected cells being present. These possibilities may be resolved by considering the relative ratios of sgmRNAs identified.

METHODS

Data input

LeTRS was designed to analyse FASTQ files derived from Illumina paired-end or Nanopore sequencing data derived from a SARS-CoV-2 amplicon protocol, or standard Nanopore SARS-CoV-2 dRNAseq data (Figure 1). The Illumina/Nanopore FASTQ sequencing data were cleaned to remove adapters and low-quality reads before input. Sequencing data derived from other sequencing modes or platforms can also be analysed by LeTRS via input of a BAM file produced by a custom splicing alignment method with a SARS-CoV-2 genome (NC_045512.2) as a reference (Figure 1). This can also be rapidly adapted for other coronaviruses.

Library preparations and sequencing

We sequenced the 15 samples from human patients with Nanopore. Total RNA was isolated using a QIAamp Viral RNA Mini Kit (Qiagen, Manchester, UK) by spin-column procedure according to the manufacturer's instructions. Clinical samples were extracted with Trizol LS as described[4]. All RNA samples were treated with Turbo DNase (Invitrogen). SuperScript IV (Invitrogen) was used to generate single-strand cDNA using random primer mix (NEB, Hitchin, UK). ARTIC V3 PCR amplicons from the single-strand cDNA were generated following the Nanopore Protocol of PCR tiling of SARS-CoV-2 virus (Version: PTC_9096_v109_revL_06Feb2020). Amplicons generated by ARTIC PCR were purified and normalised to 200 fmol before DNA end preparation and barcode and adapter ligation. Library was loaded onto a FLO-MIN106 flow cell and sequencing reads were called with Guppy using the high-accuracy calling parameters.

The NHP samples and their inoculum, and our laboratory experiments conducted in cells were sequenced with Illumina. The amplicons products for Illumina sequencing were prepared as per the Nanopore sequencing above and then used in Illumina NEBNext Ultra II DNA Library preparation. Following 4 cycles of amplification the library was purified using Ampure XP beads and quantified using Qubit and the size distribution assessed using the Fragment analyzer. Finally, the ARTIC library was sequenced on the Illumina® NovaSeq 6000 platform (Illumina®, San Diego, USA, RRID:SCR_016387) following the standard workflow. The generated raw FastQ files (2 x 250 bp) were trimmed to remove Illumina adapter sequences using Cutadapt v1.2.1 (RRID:SCR_011841)[34]. The option "−O 3" was set, so the that 3' end of any reads which matched the adapter sequence with greater than 3 bp was trimmed off. The reads were further trimmed to remove low quality bases, using Sickle v1.200 [35] with a minimum window quality score of 20. After trimming, reads shorter than 10 bp were removed.

The LeTRS was also tested with a combined Nanopore-ARTIC v3 amplicon dataset of 7 published viral cell culture samples (barcode01-barcode07) [16], and a dataset from a published direct RNA Nanopore sequencing analysis Vero cells infected with SARS-CoV-2 or an uninfected negative control [2].

Sequencing data alignment and basic filtering

LeTRS controlled Hisat2 v2.1.0 (RRID:SCR_015530)[36] to map the paired-end Illumina reads against the SARS-CoV-2 reference genome (NC_045512.2) with the default setting, and Minimap2 v2.1 [19] to align the Nanopore cDNA reads and direct RNA-seq reads on the viral

genome using Minimap2 with "–ax splice" and "-ax splice -uf -k14" parameters, respectively. LeTRS provided 10 known leader-TRS junctions to improve alignment accuracy by using "--known-splicesite-infile" function in Hisat2 and "--junc-bed" function in Minimap2, but this application could be optionally switched off by users. In order to remove low mapping quality and mis-mapped reads before searching the leader-TRS junction sites, LeTRS used Samtools v1.9 (RRID:SCR_002105)[37] to have basic filtering for the reads in the output Sam/Bam files according to their alignment states as shown (Table 9 - basic filtering).

Searching the leader-TRS motifs

After the mapping and basic filtering step, LeTRS searched aligned reads spanning the leader-TRS junctions in the SARS-CoV-2 reference genome (Supplementary Figure 1). For the known leader-TRS junctions, LeTRS searched the reads including the leader-TRS junctions within a given interval around the known leader and TRS junctions sites. The leader break site interval is ±10 nts, and the TRS breaking sites interval is -20 nts to the 1 nt before the first known AUG in the default setting (the intervals can be changed to custom values to investigate heterogeneity). LeTRS then reported a peak count that was the number of reads carrying the most common leader-TRS junctions within the given leader and TRS breaking sites intervals, and a cluster count that was the number of all reads carrying leader-TRS junctions within the given leader and TRS breaking sites intervals (Tables 1-6). LeTRS also searched the junctions out of the given intervals (the genomic position of leader breaking site < 80) and reported the number of reads (>10 by default) with novel leader-TRS junctions. These number of read counts were also reported by number of reads in 1000000 as normalisation. The read including the known and novel leader-TRS junctions

could be optionally outputted in FastA format. Based on identified known and novel leader-TRS junctions, LeTRS could report 20 nucleotides towards the 3' end of the leader sequence, the TRS and translated the first orf of sgmRNAs sequence, and find the conserved ACGAAC sequences in the TRS (Table S1-S6).

Advance filtering

Based on the alignment possibilities illustrated in Figure 2 and discussed, LeTRS further filters the identified reads with known and novel leader-TRS junctions. This step is named as advance filtering and can only applied when the input data is from Illumina paired-end reads, Nanopore cDNA reads or Nanopore RNA reads (Table 2). If a BAM file is used as input data, the advanced filtering step would be automatically skipped (Table 2). The number of reads including the known and novel leader-TRS junctions, and the number of reads filtered with corresponding advance filtering criteria were outputted into two tables in tab format (Tables 1-6).

Leader-TRS junction plotting

LeTRS-plot was developed as an automatic plotting tool that interfaces with the R package ggplot2 v3.3.3 to view the leader-TRS junctions in the tables generated by LeTRS (Figure 3-5). The plot shows peak count, filtered peak count, normalized peak count and normalized filtered peak count for known leader-TRS junctions, and novel junction counts, filtered novel junction count, normalized novel junction count and filtered normalized novel junction for novel leader-TRS junctions.

Simulation of Illumina and Nanopore reads

To assess the performance of LeTRS and other tools, simulated Illumina reads were generated using ART (v2.5.8) [38] and Nanopore reads were generated using NanoSim (v2.6.0, RRID:SCR_018243) [39]. The real reads generated by the ARTIC-Nanopore approach, ARTIC-Illumina approach and Nanopore dRNAseq approach for the hACE2-A549 cells infected with SARS-CoV-2 were used to create custom Illumina and Nanopore read quality/error profiles with ART and NanoSim. Illumina paired reads (2x250 bp) and Nanopore cDNA-1D read for both ARTIC and sgmRNA amplicons were simulated at 50000 × coverage for each amplicon and 2,000,000 reads in total, respectively. Nanopore dRNAseq reads (2,000,000) of the sgmRNA and viral genome were generated using transcriptome mode.

RNA modifications

Total RNA extracted from cultured cells at 6, 12 and 24 hours were collected for Oxford Nanopore direct RNA sequence. LeTRS was then run with a parameter of "extractfasta" to extract subgenomic mRNAs reads in sequenced samples. The fast5 files that corresponds to the extracted subgenomic mRNAs reads were withdrawn using fast5_subset in Oxford Nanopore ont_fast5_api package (v0.3.2, https://github.com/nanoporetech/ont_fast5_api). The re-squiggle algorithm in Tombo analysis pipelines (v1.5.1, https://github.com/nanoporetech/tombo) defines a new assignment from raw signals to reference sequence with "--num-most-common-errors 5" option. The resquiggled raw signals were further processed using "detect_modifications alternative_model" functions in Tombo by setting "--rna and --alternate-bases 5mC" to identify 5-methylcytosine (5mC), and "predict_sites"

in Nanom6A package (v2021_10_22) [40] with default setting to identify N6-methyladenosine (6mA) in the subgenomic mRNAs reads.

Ethics approval and consent to participate

All experimental work on NHPs was conducted under the authority of a UK Home Office approved project license (PDC57C033) that had been subject to local ethical review at PHE Porton Down by the Animal Welfare and Ethical Review Body (AWERB) and approved as required by the Home Office Animals (Scientific Procedures) Act 1986 and the full ethics and NHP model are described.

Consent for publication

Not applicable

Data Availability

Illumina and Nanopore test data sets are available under NCBI PRJNA699398. Snapshots of the code are available in the *GigaScience* GigaDB repository[41].

Availability and requirements

- Project name: LeTRS
- Project home page: https://github.com/Hiscox-lab/LeTRS
- Operating system(s): Platform independent
- Programming language: Perl
- Other requirements: samtools(>=1.11), hisat2(=2.1.0), minimap2(=2.17), portcullis(>=1.1.2)
- License: Apache 2.0
- RRID:SCR_022138

Competing interests

The authors declare that they have no competing interests

Funding

This work was predominately funded by U.S. Food and Drug Administration Medical Countermeasures Initiative contract (75F40120C00085) awarded to JAH. The article reflects the views of the authors and does not represent the views or policies of the FDA. This work was also supported by the MRC (MR/W005611/1) G2P-UK: A national virology consortium to address phenotypic consequences of SARS-CoV-2 genomic variation (co-I JAH). JAH is also funded by the Centre of Excellence in Infectious Diseases Research (CEIDR) and the Alder Hey Charity. The non-human primate work was funded by the Coalition of Epidemic Preparedness Innovations (CEPI) and the Medical Research Council Project CV220-060, Development of an NHP model of infection and ADE with COVID-19 (SARS-CoV-2) both awarded to MWC. The ISARIC4C sample collection and sequencing in this study was supported by a grants from the Medical Research Council (grant MC_PC_19059), the National Institute for Health Research (NIHR; award CO-CIN-01), the Medical Research Council (MRC; grant MC_PC_19059), and by the NIHR Health Protection Research Unit (HPRU) in Emerging and Zoonotic Infections at University of Liverpool in partnership with Public Health England (PHE), in collaboration with Liverpool School of Tropical Medicine and the University of Oxford (award 200907), NIHR HPRU in Respiratory Infections at Imperial College London with PHE (award 200927), Wellcome Trust and Department for International Development (DID; 215091/Z/18/Z), the Bill and Melinda Gates Foundation (OPP1209135), Liverpool Experimental Cancer Medicine Centre (grant reference C18616/A25153), NIHR Biomedical Research Centre at Imperial College London (IS-BRC-1215-20013), PJMO is supported by a NIHR senior investigator award (201385). The views expressed are those of the authors and not necessarily those of the Department of Health and Social Care, DID, NIHR, MRC, Wellcome

Trust, or PHE. The funders had no role in the study design; in the collection, analysis, and interpretation of data; in the writing of the report; or in the decision to submit the article for publication.

Authors' contributions

X.D. developed the LeTRS software and performed the informatics analysis. X.D., A.D. and J.A.H. analysed the data. J.S., J.T. and M.W.C. co-ordinated the NHP work and sample processing. R.P.-R., J.P.S., H.G., T.P. and N.R. were involved in sequencing and informatics analysis of the NHP samples with D.A.M. A.D. oversaw sequencing of the human clinical samples with E.V. and C.N for the COG-UK data. R.P.-R. and J.A.H. oversaw sequencing of samples under the auspices of ISARIC-4C with clinical samples collected and managed by J.K.B, L.T., M.G.S. and P.J.M.O. J.A.H. and M.W.C. initiated and led the study and wrote the manuscript with X.D., R.P.-R., A.D. with other authors involved in editing the final version.

Acknowledgments

We would like to thank all members of the Hiscox Laboratory and the Centre for Genome Research for supporting SARS-CoV-2/COVID-19 sequencing research. We would like to acknowledge members of the COG-UK and ISARIC4C consortia for acquisition of the human samples used in this study.

References

1. Davidson, A.D., et al., *Characterisation of the transcriptome and proteome of SARS-CoV-2 reveals a cell passage induced in-frame deletion of the furin-like cleavage site from the spike glycoprotein.* Genome Med, 2020. **12**(1): p. 68.
2. Kim, D., et al., *The Architecture of SARS-CoV-2 Transcriptome.* Cell, 2020. **181**(4): p. 914-921 e10.
3. Nasir, J.A., et al., *A Comparison of Whole Genome Sequencing of SARS-CoV-2 Using Amplicon-Based Sequencing, Random Hexamers, and Bait Capture.* Viruses, 2020. **12**(8).
4. Moore, S.C., et al., *Amplicon-Based Detection and Sequencing of SARS-CoV-2 in Nasopharyngeal Swabs from Patients With COVID-19 and Identification of Deletions in the Viral Genome That Encode Proteins Involved in Interferon Antagonism.* Viruses, 2020. **12**(10).
5. Dorward, D.A., et al., *Tissue-Specific Immunopathology in Fatal COVID-19.* Am J Respir Crit Care Med, 2021. **203**(2): p. 192-201.
6. Graham, R.L., et al., *SARS coronavirus replicase proteins in pathogenesis.* Virus Res, 2008. **133**(1): p. 88-100.
7. Pyrc, K., et al., *Genome structure and transcriptional regulation of human coronavirus NL63.* Virol J, 2004. **1**: p. 7.
8. Hiscox, J.A., D. Cavanagh, and P. Britton, *Quantification of individual subgenomic mRNA species during replication of the coronavirus transmissible gastroenteritis virus.* Virus Res, 1995. **36**(2-3): p. 119-30.

9. Hiscox, J.A., et al., *Investigation of the control of coronavirus subgenomic mRNA transcription by using T7-generated negative-sense RNA transcripts.* J Virol, 1995. **69**(10): p. 6219-27.

10. van Marle, G., et al., *Regulation of coronavirus mRNA transcription.* J Virol, 1995. **69**(12): p. 7851-6.

11. La Monica, N., K. Yokomori, and M.M. Lai, *Coronavirus mRNA synthesis: identification of novel transcription initiation signals which are differentially regulated by different leader sequences.* Virology, 1992. **188**(1): p. 402-7.

12. Alonso, S., et al., *Transcription regulatory sequences and mRNA expression levels in the coronavirus transmissible gastroenteritis virus.* J Virol, 2002. **76**(3): p. 1293-308.

13. Sawicki, S.G., D.L. Sawicki, and S.G. Siddell, *A contemporary view of coronavirus transcription.* J Virol, 2007. **81**(1): p. 20-9.

14. Jeong, Y.S. and S. Makino, *Evidence for coronavirus discontinuous transcription.* J Virol, 1994. **68**(4): p. 2615-23.

15. Cevik, M., et al., *Virology, transmission, and pathogenesis of SARS-CoV-2.* BMJ, 2020. **371**: p. m3862.

16. Tyson, J.R., et al., *Improvements to the ARTIC multiplex PCR method for SARS-CoV-2 genome sequencing using nanopore.* bioRxiv, 2020. https://doi.org/10.1101/2020.09.04.283077

17. Freed, N.E., et al., *Rapid and inexpensive whole-genome sequencing of SARS-CoV-2 using 1200 bp tiled amplicons and Oxford Nanopore Rapid Barcoding.* Biology Methods and Protocols, 2020. **5**(1): p. bpaa014.

18. !!! INVALID CITATION !!! [2].

19. Li, H., *Minimap2: pairwise alignment for nucleotide sequences.* Bioinformatics, 2018. **34**(18): p. 3094-3100.

20. Young, B.E., et al., *Effects of a major deletion in the SARS-CoV-2 genome on the severity of infection and the inflammatory response: an observational cohort study.* The Lancet, 2020. **396**(10251): p. 603-611.

21. Parker, M.D., et al., *Subgenomic RNA identification in SARS-CoV-2 genomic sequencing data.* Genome research, 2021. **31**(4): p. 645-658.

22. Yang, Y., et al., *Characterizing transcriptional regulatory sequences in coronaviruses and their role in recombination.* Molecular Biology and Evolution, 2021. **38**(4): p. 1241-1248.

23. Anders, S., et al., *Count-based differential expression analysis of RNA sequencing data using R and Bioconductor.* Nature protocols, 2013. **8**(9): p. 1765-1786.

24. Alexandersen, S., A. Chamings, and T.R. Bhatta, *SARS-CoV-2 genomic and subgenomic RNAs in diagnostic samples are not an indicator of active replication.* Nature communications, 2020. **11**(1): p. 1-13.

25. Ross, M.G., et al., *Characterizing and measuring bias in sequence data.* Genome biology, 2013. **14**(5): p. 1-20.

26. Salguero, F.J., et al., *Comparison of rhesus and cynomolgus macaques as an infection model for COVID-19.* Nat Commun, 2021. **12**(1): p. 1260.

27. Ryan, K.A., et al., *Dose-dependent response to infection with SARS-CoV-2 in the ferret model and evidence of protective immunity.* Nat Commun, 2021. **12**(1): p. 81.

28. Taiaroa, G., et al., *Direct RNA sequencing and early evolution of SARS-CoV-2.* BioRxiv, 2020.
29. Keep, S., et al., *Multiple novel non-canonically transcribed sub-genomic mRNAs produced by avian coronavirus infectious bronchitis virus.* J Gen Virol, 2020. **101**(10): p. 1103-1118.
30. Nomburg, J., M. Meyerson, and J.A. DeCaprio, *Pervasive generation of non-canonical subgenomic RNAs by SARS-CoV-2.* Genome Med, 2020. **12**(1): p. 108.
31. Corbett, K.S., et al., *Evaluation of the mRNA-1273 Vaccine against SARS-CoV-2 in Nonhuman Primates.* N Engl J Med, 2020. **383**(16): p. 1544-1555.
32. Yu, J., et al., *DNA vaccine protection against SARS-CoV-2 in rhesus macaques.* Science, 2020. **369**(6505): p. 806-811.
33. Alexandersen, S., A. Chamings, and T.R. Bhatta, *SARS-CoV-2 genomic and subgenomic RNAs in diagnostic samples are not an indicator of active replication.* Nat Commun, 2020. **11**(1): p. 6059.
34. Martin, M., *Cutadapt removes adapter sequences from high-throughput sequencing reads.* EMBnet.journal, 2011. **17**: p. https://doi.org/10.14806/ej.17.1.200.
35. Joshi, N.A. and J.N. Fass, *Sickle: A sliding-window, adaptive, quality-based trimming tool for FastQ files*
(Version 1.33). 2011: p. https://github.com/najoshi/sickle.
36. Kim, D., B. Langmead, and S.L. Salzberg, *HISAT: a fast spliced aligner with low memory requirements.* Nat Methods, 2015. **12**(4): p. 357-60.
37. Li, H., et al., *The Sequence Alignment/Map format and SAMtools.* Bioinformatics, 2009. **25**(16): p. 2078-9.

38. Huang, W., et al., *ART: a next-generation sequencing read simulator.* Bioinformatics, 2012. **28**(4): p. 593-594.

39. Yang, C., et al., *NanoSim: nanopore sequence read simulator based on statistical characterization.* GigaScience, 2017. **6**(4): p. gix010.

40. Gao, Y., et al., *Quantitative profiling of N 6-methyladenosine at single-base resolution in stem-differentiating xylem of Populus trichocarpa using Nanopore direct RNA sequencing.* Genome Biology, 2021. **22**(1): p. 1-17.

41. Dong X; Penrice-Randal R; Goldswain H; Prince T; Randle N; Donavan-Banfield I; Salguero FJ; Tree J; Vamos E; Nelson C; Clark J; Ryan Y; Stewart JP; Semple MG; Baillie JK; M Openshaw PJ; Turtle L; Matthews DA; Carroll MW; Darby AC; Hiscox JA (2022): Supporting data for"Identification and quantification of SARS-CoV-2 leader subgenomic mRNA gene junctions in nasopharyngeal samples shows phasic transcription in animal models of COVID-19 and dysregulation at later time points that can also be identified in humans" GigaScience Database. http://dx.doi.org/10.5524/102209

Ethics approval and consent to participate

All experimental work on NHPs was conducted under the authority of a UK Home Office approved project license (PDC57C033) that had been subject to local ethical review at PHE Porton Down by the Animal Welfare and Ethical Review Body (AWERB) and approved as required by the Home Office Animals (Scientific Procedures) Act 1986 and the full ethics and NHP model are described.

Consent for publication

Not applicable

Data Availability

Illumina and Nanopore test data sets are available under NCBI PRJNA699398. Snapshots of the code are available in the *GigaScience* GigaDB repository[41].

LeTRS is available at https://github.com/xiaofengdong83/LeTRS.

Competing interests

The authors declare that they have no competing interests

Funding

This work was predominately funded by U.S. Food and Drug Administration Medical Countermeasures Initiative contract (75F40120C00085) awarded to JAH. The article reflects the views of the authors and does not represent the views or policies of the FDA. This work was also supported by the MRC (MR/W005611/1) G2P-UK: A national virology consortium to address phenotypic consequences of SARS-CoV-2 genomic variation (co-I JAH). JAH is also funded by the

Centre of Excellence in Infectious Diseases Research (CEIDR) and the Alder Hey Charity. The non-human primate work was funded by the Coalition of Epidemic Preparedness Innovations (CEPI) and the Medical Research Council Project CV220-060, Development of an NHP model of infection and ADE with COVID-19 (SARS-CoV-2) both awarded to MWC. The ISARIC4C sample collection and sequencing in this study was supported by a grants from the Medical Research Council (grant MC_PC_19059), the National Institute for Health Research (NIHR; award CO-CIN-01), the Medical Research Council (MRC; grant MC_PC_19059), and by the NIHR Health Protection Research Unit (HPRU) in Emerging and Zoonotic Infections at University of Liverpool in partnership with Public Health England (PHE), in collaboration with Liverpool School of Tropical Medicine and the University of Oxford (award 200907), NIHR HPRU in Respiratory Infections at Imperial College London with PHE (award 200927), Wellcome Trust and Department for International Development (DID; 215091/Z/18/Z), the Bill and Melinda Gates Foundation (OPP1209135), Liverpool Experimental Cancer Medicine Centre (grant reference C18616/A25153), NIHR Biomedical Research Centre at Imperial College London (IS-BRC-1215-20013), PJMO is supported by a NIHR senior investigator award (201385). The views expressed are those of the authors and not necessarily those of the Department of Health and Social Care, DID, NIHR, MRC, Wellcome Trust, or PHE. The funders had no role in the study design; in the collection, analysis, and interpretation of data; in the writing of the report; or in the decision to submit the article for publication.

Authors' contributions

X.D. developed the LeTRS software and performed the informatics analysis. X.D., A.D. and J.A.H. analysed the data. J.S., J.T. and M.W.C. co-ordinated the NHP work and sample processing. R.P.-R., J.P.S., H.G., T.P. and N.R. were involved in sequencing and informatics analysis of the NHP samples with D.A.M. A.D. oversaw sequencing of the human clinical samples with E.V. and C.N for the COG-UK data. R.P.-R. and J.A.H. oversaw sequencing of samples under the auspices of ISARIC-4C with clinical samples collected and managed by J.K.B, L.T., M.G.S. and P.J.M.O. J.A.H. and M.W.C. initiated and led the study and wrote the manuscript with X.D., R.P.-R., A.D. with other authors involved in editing the final version.

Acknowledgments

We would like to thank all members of the Hiscox Laboratory and the Centre for Genome Research for supporting SARS-CoV-2/COVID-19 sequencing research. We would like to acknowledge members of the COG-UK and ISARIC4C consortia for acquisition of the human samples used in this study.

Table 1. Comparison of other Tools with LeTRS.

		LeTRS	Periscope	SARS-CoV-2-leader	SuPER
Input files		fastq	fastq	bam/sam	sam
Consideration of amplicon primer information used		yes	yes	no	no
Consideration of paired-end Illumina data		yes	no	no	no
Consideration of amplicon primer pool		yes	no	no	no
Consideration of the ACGAAC box		yes	no	no	yes
Support of amplicon Illumina data		yes	yes	yes	yes
Support of amplicon Nanopore data		yes	yes	yes	yes
Support of Nanopore dRNAseq data		yes	no	yes	yes
Method		Fusion site searching	Sequences tag searching	Sequences tag searching	Fusion site searching
Accuracy	ARTIC-Illumina	1.0000	0.9998	0.9998	0.9996
	ARTIC-Nanopore	0.9985	0.9981	0.9980	0.9979
	Nanopore dRNAseq	0.9982	-	0.9948	0.9937
Sensitivity	ARTIC-Illumina	0.9997	0.9498	0.9644	0.9230
	ARTIC-Nanopore	0.6294	0.5326	0.5154	0.4843
	Nanopore dRNAseq	0.8448	-	0.5949	0.4817
Specificity	ARTIC-Illumina	1.0000	1.0000	1.0000	1.0000
	ARTIC-Nanopore	1.0000	1.0000	1.0000	1.0000
	Nanopore dRNAseq	1.0000	-	1.0000	1.0000
F-measure	ARTIC-Illumina	0.9998	0.9499	0.9655	0.9243
	ARTIC-Nanopore	0.7621	0.6699	0.6611	0.6215
	Nanopore dRNAseq	0.9157	-	0.7140	0.5934

Accuracy, sensitivity, specificity and F-measure score were calculated with simulated Illumina and Nanopore sequencing reads for the known sgmRNAs.

Table 2. The criteria of basic and advanced filtering for four different types of input data for LeTRS.

	Output Filters	Illumina paired-end amplicon reads	Nanopore amplicon reads	Nanopore dRNAseq reads	Bam
Basic filtering	MAPQ > 10	●	●	●	●
	Read only one splicing junction	●	●	●	●
	Primary alignment only	●	●	●	●
	No supplementary alignment	●	●	●	●
	Read mapped in pair	●			
	No read reverse strand			●	
Advance filtering	Read aligment5' end includes forward primer	●	●		
	Read aligment3' end includes reverse primer	●	●		
	Read aligment5' end includes forward primer and 3' end includes reverse primer	●	●		
	Paired read including at least one primer in each have same leader-TRS junction in alignments	●	●		
	Read aligment3' with > 1ployA		●	●	

Read aligment3' with > 5ployA	●	●

Figures

Figure 1. (A). Illustration of reads derived from sgmRNAs mapped onto the SARS-CoV-2 reference genome with a splicing method. We note that splicing does not occur in coronaviruses but this is the apparent observation of a fusion event between different parts of the genome. (B and C). Illustration of the possible type of reads mapped on the SARS-CoV-2 reference genome for the paired-end Illumina amplicon sequencing, where the lines with same colour implied paired reads, (D) Nanopore amplicon sequencing and (E) Nanopore dRNAseq of the SARS-CoV-2 genome and sgmRNAs. L and B in the boxes indicate the leader-TRS breaking sites on the leader side and TRS side, respectively. Although we note these are where the apparent fusion site occurs. Yellow colour indicates the leader region, black is the TRS and gene sequence, the red indicates a sequence read that maps to SARS-CoV-2 sequence. Blue is a sequence that is present between the leader sequence and the TRS. For (B) and (C) the same colour (brown, green and pink) indicates that same paired read. For (B) the paired read contains both primers. For (C) the grey and light blue colour is a paired read, but only contains one primer sequence at any end. The vertical hash lines on (B, C, and D) indicates the position of a primer.

Figure 2. Analysis of reads mapping to the leader TRS-gene junctions with at least one primer sequence at either end in sequencing data from hACE2-A549 cells infected with SARS-CoV-2 and sequenced using either (A) an ARTIC-Nanopore approach, (B) an ARTIC-Illumina approach and (C) a Nanopore dRNAseq approach. The data corresponds to that shown in detailed in Supplementary Tables 1, 2 and 3. The standard deviation of a binomial distribution was calculated to generate error bars. The data is presented as a histogram with a normalised count for each

sgmRNA starting at a particular position in the leader sequence as indicated in the line diagram underneath. For each panel (A, B and C) the expected sgmRNA pattern is shown on the left and novel sgmRNAs are shown on the right.

Figure 3. An X-Y/scatter plot using normalized counts of sgmRNAs (with greater than 5 A residues at the 3' end – indicative of a polyA tail for the dRNAseq data). To generate the scatter plots Nanopore dRNAseq data was plotted against the either the normalized count (at least one primer sequence) of sgmRNAs with (A) ARTIC-Nanopore sequencing data and (C) ARTIC-Illumina sequencing data or provided as ratio (B) and (D), respectively for S:orf3:E:M:orf6:orf7a:orf7b:orf8:N:orf10 (using data from Supplementary Tables 3, 4 and 5).

Figure 4. Analysis of the abundance of reads mapping to the leader TRS-gene junctions that have at least one primer sequence at either end in longitudinal nasopharyngeal samples taken from two non-human primate models infected with SARS-CoV-2. The time post-infection in days is indicated on the x-axis. The normalised count (read count/total number of reads mapped on the reference genome)*1,000,000) of the leader TRS-gene junction abundance is shown on the left-hand Y-axis with each unique leader TRS-gene junction colour coded. The right-hand Y axis is a measure of the total depth of coverage for SARS-CoV-2 in that sample. Note the two scales are different. SARS-CoV-2 was amplified and sequenced by ARTIC-Illumina. The data is organised into groups of animals for the cynomolgus macaque groups 1 and 2 (A/E and B/F), and rhesus macaque groups 1 and 2 (C/G and D/H). E, F, G and H zoom in to see the details of A, B, C and D

for Day1 to Day9. The data corresponds to that shown in Supplementary Table 7. Standard deviation of a binomial distribution was calculated to provide error bars.

Figure 5. Plots of normalised peak counts (A) and peak counts (B) of leader-TRS gene junctions of reads with at least one primer sequences at either end derived from sequence data from 15 human patients sequenced with the ARTIC-Illumina approach and analysed by using sequence derived from pool 1 primers. The data correspond to that shown in Supplementary Table 8. Standard deviation of a binomial distribution was calculated to provide error bars.

Figure 6. Plots of normalised peak counts (A) and peak counts (B) of leader-TRS gene junctions of reads with at least one primer sequence at either end derived from sequence data from 15 human patients sequenced with the ARTIC-Nanopore approach and analysed by using sequence derived from pool 1 primers. The data correspond to that shown in Supplementary Table 9. Standard deviation of a binomial distribution was calculated to provide error bars.

Figure 7. (A). Diagram of novel leader-TRS junctions centred around the known gene orf but out of the search interval in the analysis of SARS-CoV-2 RNA from cell culture, non-human primate and human sequencing data. Many novel junctions map to the leader-TRS membrane gene junctions. (B). Venn diagram showing the overlap of novel leader-TRS gene junctions present in SARS-CoV-2 infected cynomolgus and rhesus macaques, human patients, and Vero cells. Data was obtained using the ATRIC-Illumina method (Supplementary Table 15). (C) Virus genome position of the start of the fusion site (Y-axis) in the leader sequence plotted against the fusion

site present in the gene to show the potential positions of the novel leader-TRS junctions along the SARS-CoV-2 genome (indicated above). A shown the colours present the novel leader-TRS junctions identified in the different experimental condition (cynomolgus and rhesus macaques, human patients, and Vero cells).

Supplementary Figures

Supplementary Figure 1. Bioinformatics pipeline for the identification of leader-TRS junctions in sequencing data from SARS-CoV-2 infected material with LeTRS. This can be rapidly adapted for other coronaviruses such as MERS-CoV and any newly emerged coronavirus. LeTRS can work from Nanopore or Illumina amplicon data or more unbiased approaches such as direct RNA sequencing, metagenomic or Illumina sequencing by using a BAM file.

Supplementary Figure 2. Novel (leader dependent noncanonical) fusions (count >=2) found in the cell culture test sample sequenced by (A) ARTIC-Nanopore, (B) ARTIC-Illumina and (C) Nanopore dRNAseq approaches; leader independent long-distance (>5,000 nt) fusions (count >=2) found in the cell culture test sample sequenced by (D) ARTIC-Nanopore, (E) ARTIC-Illumina and (F) Nanopore dRNAseq approaches; leader independent local joining yielding a deletion between proximal sites (20–5,000 nt distance) fusions (count >=2) found in the cell culture test sample sequenced by (G) ARTIC-Nanopore, (H) ARTIC-Illumina and (I) Nanopore dRNAseq approaches. The data correspond to that shown Supplementary Tables 1, 2 and 3.

Supplementary Figure 3. Comparison of different tools and LeTRS to evaluate sequencing data to identify the unique sequencing features of SARS-CoV-2 sgmRNAs. Number of reads were evaluated by LeTRS (all peak count), SARS-COV-2-leader, SuPER or periscope (High Quality count) with the cell culture test sample sequenced by (A) ARTIC-Nanopore, (B) ARTIC-Illumina and (C) Nanopore dRNAseq approaches; (D) Ratio of sgmRNAs (S:orf3:E:M:orf6:orf7a:orf7b:orf8:N:orf10) identified by LeTRS (all peak count), SARS-COV-2-

leader, SuPER or periscope (HQ count) with the cell culture test sample sequenced by ARTIC-Nanopore, ARTIC-Illumina and Nanopore dRNAseq approaches. The data are corresponded to that shown in Supplementary Tables 1, 2 and 3.

Supplementary Figure 4. Comparison of the ratio of reads in amplicon sequencing approaches based on the ARTIC approach, with the forward primer only, reads with reverse primer only and reads with both primers in sgmRNAs to the overall ratio of reads with the forward primer only, reads with reverse primer only and reads with both primers in all reads amplified by pool 1 primers, pool 2 primers and both pools of primers for the cell culture test sample sequenced by (A) ARTIC-Nanopore and (B) ARTIC-Illumina approaches.

Supplementary Figure 5. Raw (A and C) and normalised (B and D) canonical (upper) and novel (lower) leader-TRS gene junctions count in RNA purified from the inoculum of SARS-CoV-2 used to infect either the cynomolgus or rhesus macaques. The RNA was sequenced by the ARTIC-Illumina method (Supplementary Table 6). Standard deviation of a binomial distribution was calculated to provide error bars.

Supplementary Figure 6. Novel leader-TRS gene junctions (count > 10) identified in RNA purified from nasopharyngeal swabs taken daily from cynomolgus macaques infected with SARS-CoV-2 (Supplementary Table 7). The number before "-Day" indicated the group of cynomolgus macaques. Standard deviation of a binomial distribution was calculated to provide error bars.

Supplementary Figure 7. Novel leader-TRS gene junctions (count > 10) identified in RNA purified from nasopharyngeal swabs taken daily from from rhesus macaques (Supplementary Table 7). The number before “-Day” indicated the group of cynomolgus macaques. Standard deviation of a binomial distribution was calculated to provide error bars.

Supplementary Figure 8. Comparison of the fraction of 6mA modification (right-hand Y-axis) of each site in sgmRNA at 6, 12 and 24 hours after post infection using direct RNA sequencing from RNA purified from SARS-CoV-2 infected cells. Only the sites with modification in at least one of the 6hpi, 12hpi and 24hpi were analysed.

Supplementary Figure 9. Comparison of the fraction of 5mC modification (right-hand Y-axis) of each site in sgmRNA at 6, 12 and 24 hours after post infection using direct RNA sequencing from RNA purified from SARS-CoV-2 infected cells. Only the sites with modification in at least one of the 6hpi, 12hpi and 24hpi were analysed.

Supplementary Tables

Table S1. The LeTRS output tables for known sgmRNA, details of known sgmRNA, novel sgmRNA (count >=2), details of novel sgmRNA, and leader independent long-distance and local fusions (count >=2) evaluated in the cell culture test sample sequenced by the ARTIC-Nanopore approach.

Table S2. The LeTRS output tables for known sgmRNA, details of known sgmRNA, novel sgmRNA (count >=2), details of novel sgmRNA, and leader independent long-distance and local fusions (count >=2) evaluated in the cell culture test sample sequenced by the ARTIC-Illumina approach.

Table S3. The LeTRS output tables for known sgmRNA, details of known sgmRNA, novel sgmRNA (count >=2), details of novel sgmRNA, and leader independent long-distance and local fusions (count >=2) evaluated in the cell culture test sample sequenced by the Nanopore dRNAseq approach.

Table S4. The LeTRS output table for known sgmRNA evaluated by primers of pool 1 and 2 in the cell culture test sample sequenced by the ARTIC-Nanopore approach.

Table S5. The LeTRS output tables for known sgmRNA evaluated by primers of pool 1 and 2 in the cell culture test sample sequenced by the ARTIC-Illumina approach.

Table S6. The LeTRS output tables for known sgmRNA and details of known sgmRNA with pool 1 primers, and novel sgmRNA (count > 10) and details of novel sgmRNA with both pools' primers in the infecting SARS-CoV-2 inoculum source used for the NHP study, sequenced by the ARTIC-Illumina method.

Table S7. The LeTRS output tables for known sgmRNA and details of known sgmRNA with pool 1 primers, and novel sgmRNA (count > 10) and details of novel sgmRNA with both pools' primers in longitudinal nasopharyngeal samples taken from two non-human primate models (cynomolgus and rhesus macaques) of SARS-CoV-2 in groups. SARS-CoV-2 was amplified using the ARTIC approach and sequenced by Illumina. The data is organised into groups of animals for the cynomolgus macaque groups 1 and 2 that were with "-1" and "-2" in the excel sheets.

Table S8. The LeTRS output tables for known sgmRNA and details of known sgmRNA in pool 1, and novel sgmRNA (count > 10) and details of novel sgmRNA with both pools' primers from 15 human patients sequenced with ARTIC-Illumina.

Table S9. The LeTRS output tables for known sgmRNA and details of known sgmRNA in pool 1 from 15 human patients sequenced with ARTIC-Nanopore.

Table S10. The spreadsheet for the 15 human patients sequenced with the ARTIC-Nanopore detailed in Table S9.

Table S11. Re-analysis of reads for known sgmRNAs in the (NCBI assession No. PRJNA636225) [24].

Table S12. Summary of normalized count, number of modification sites and average modification fraction in of each gmRNA at 6hpi, 12hpi and 24hpi.

Table S13. Evaluation of the difference of modification by the paired samples one-sided Wilcoxon test to calculate p-value by treating the same nucleotides between any two time points as paired data.

Table S14. The LeTRS output table for novel sgmRNA (count > 10) and details of novel sgmRNA with both primer pools from VeroE6 cells infected in culture with SARS-CoV-2 (SCV2-006) sequenced by ARTIC-Illumina primers. This sample is different from the one Table S2.

Table S15. Novel leader-TRS junctions centred around the known gene open reading frame but out of the search interval in the analysis of cell culture, non-human primate and human sequencing data.

 Click here to access/download;Figure;All_figures.pptx

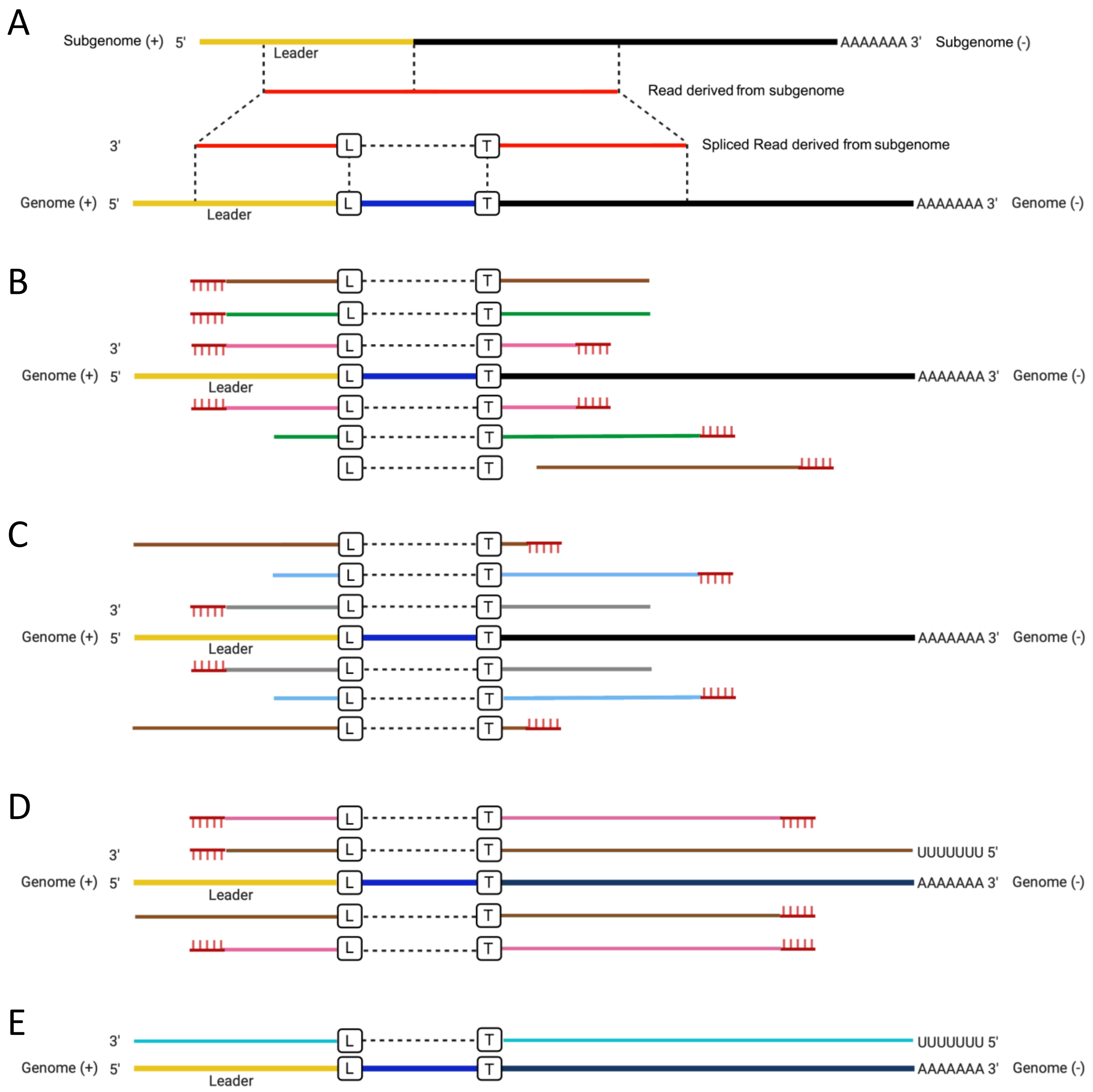

Figure 1

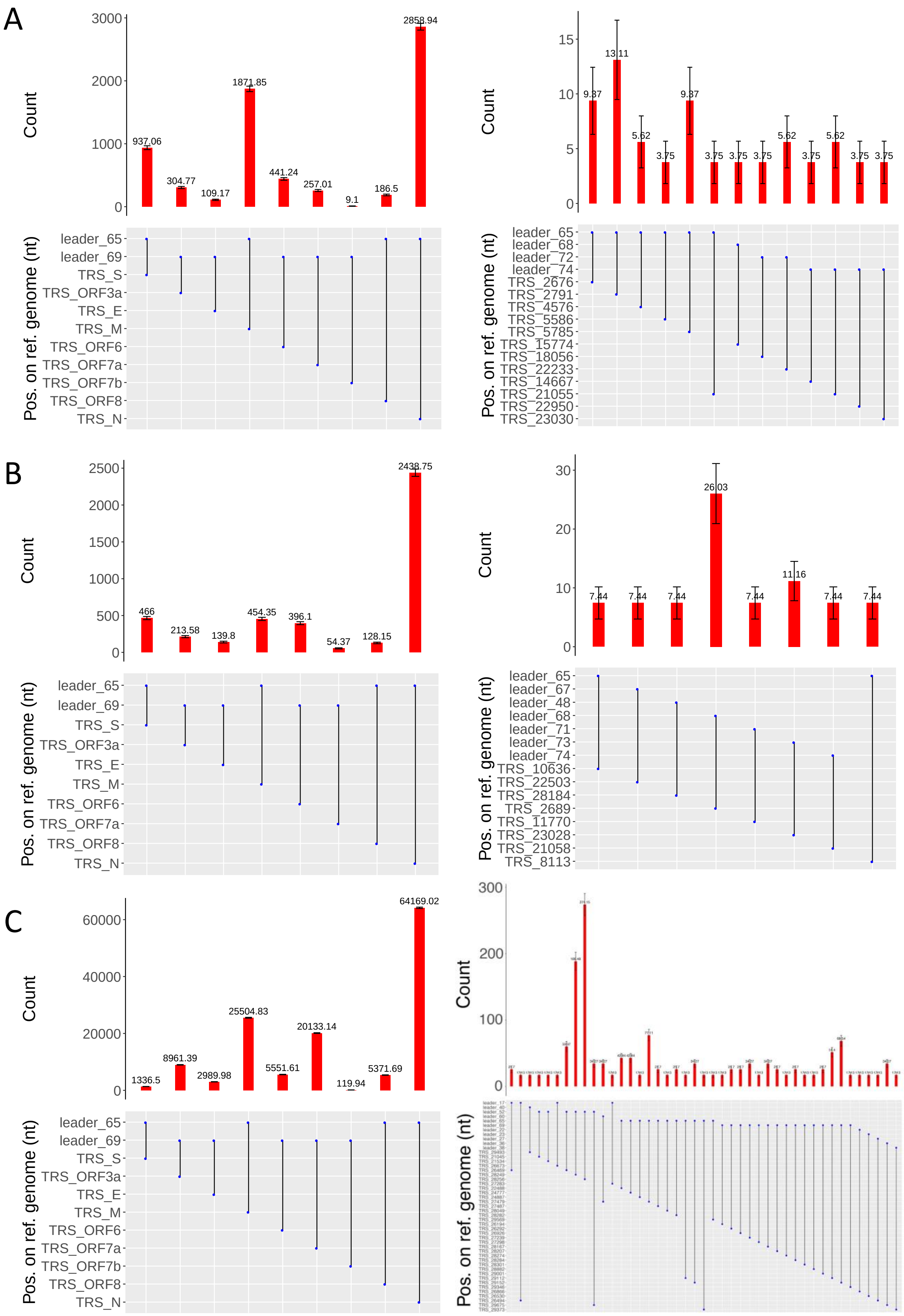
A
B
C
Count
Pos. on ref. genome (nt)
leader_65
leader_69
TRS_S
TRS_ORF3a
TRS_E
TRS_M
TRS_ORF6
TRS_ORF7a
TRS_ORF7b
TRS_ORF8
TRS_N
937.06
304.77
109.17
1871.85
441.24
257.01
9.1
186.5
2858.94
13.11
9.37
5.62
3.75
leader_68
leader_72
leader_74
TRS_2676
TRS_2791
TRS_4576
TRS_5586
TRS_5785
TRS_15774
TRS_18056
TRS_22233
TRS_14667
TRS_21055
TRS_22950
TRS_23030
466
213.58
139.8
454.35
396.1
54.37
128.15
2438.75
26.03
11.16
7.44
leader_67
leader_48
leader_71
leader_73
TRS_10636
TRS_22503
TRS_28184
TRS_2689
TRS_11770
TRS_23028
TRS_21058
TRS_8113
1336.5
8961.39
2989.98
25504.83
5551.61
20133.14
119.94
5371.69
64169.02

Figure 2

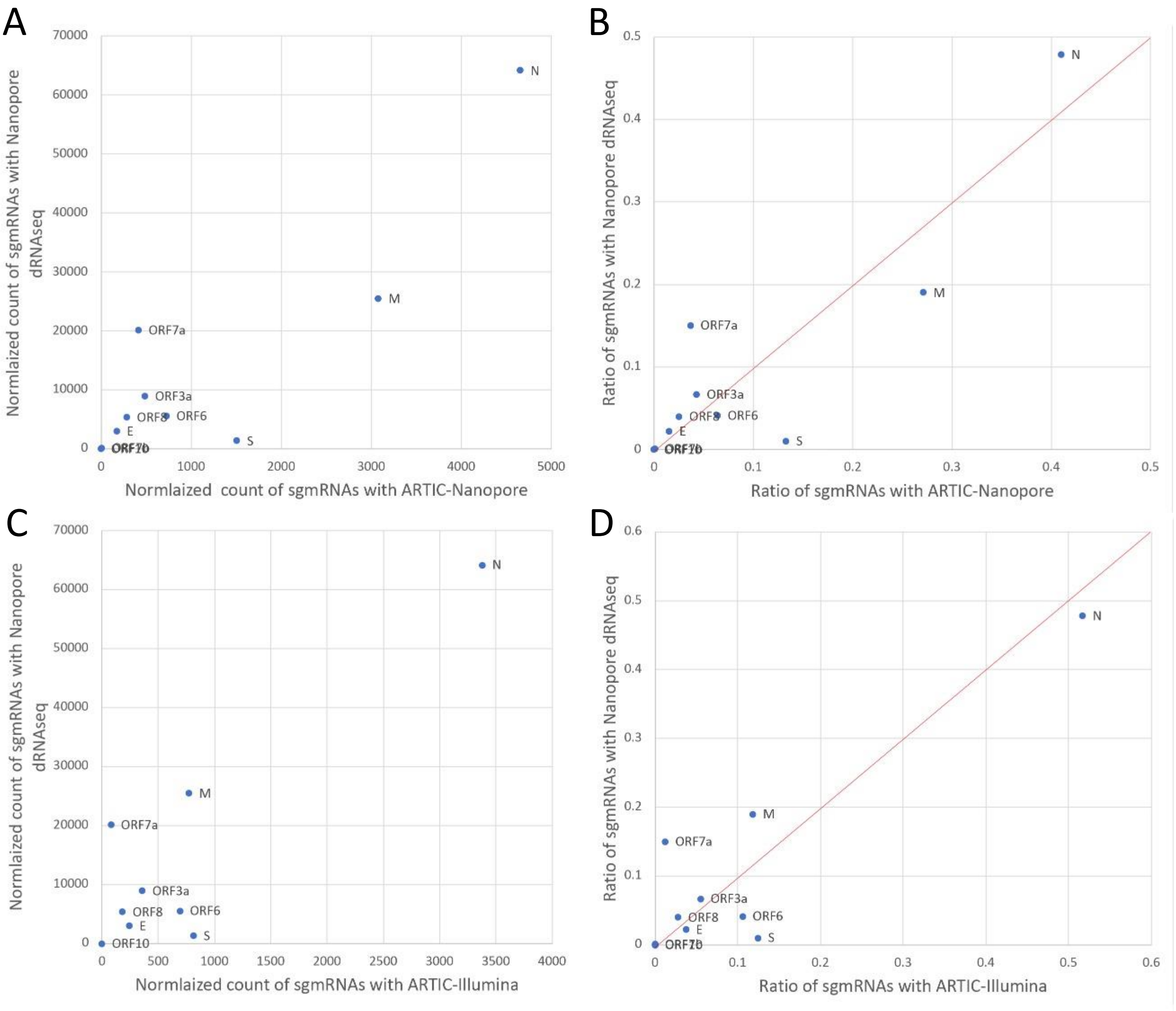
A
Normlaized count of sgmRNAs with Nanopore dRNAseq
Normlaized count of sgmRNAs with ARTIC-Nanopore
N
M
ORF7a
ORF3a
ORF8
ORF6
E
S
ORF10
B
Ratio of sgmRNAs with Nanopore dRNAseq
Ratio of sgmRNAs with ARTIC-Nanopore
C
Normlaized count of sgmRNAs with ARTIC-Illumina
D
Ratio of sgmRNAs with ARTIC-Illumina

Figure 3

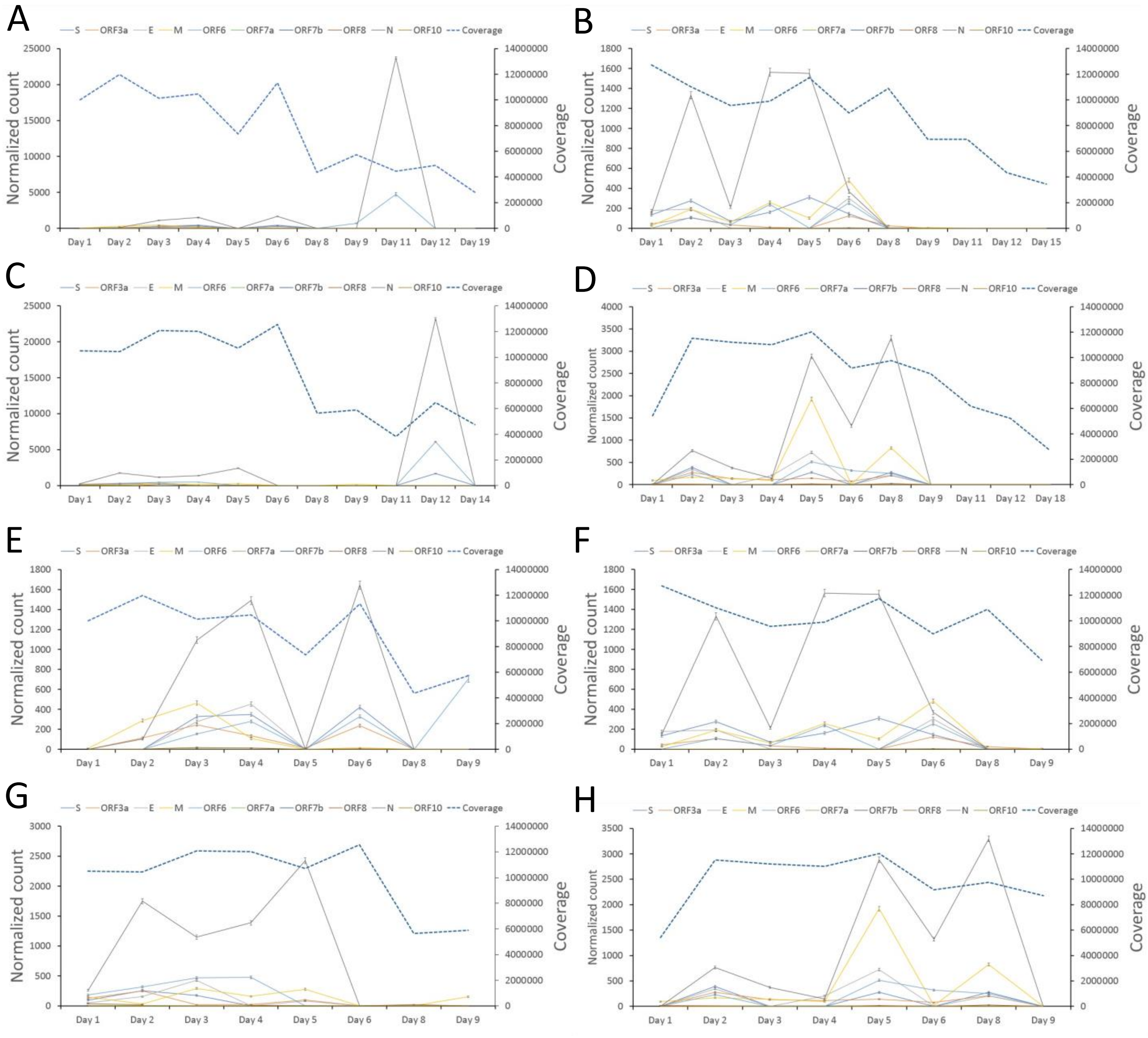
A
S ORF3a E M ORF6 ORF7a ORF7b ORF8 N ORF10 Coverage
Normalized count
Coverage
Day 1 Day 2 Day 3 Day 4 Day 5 Day 6 Day 8 Day 9 Day 11 Day 12 Day 19
B
S ORF3a E M ORF6 ORF7a ORF7b ORF8 N ORF10 Coverage
Normalized count
Coverage
Day 1 Day 2 Day 3 Day 4 Day 5 Day 6 Day 8 Day 9 Day 11 Day 12 Day 15
C
S ORF3a E M ORF6 ORF7a ORF7b ORF8 N ORF10 Coverage
Normalized count
Coverage
Day 1 Day 2 Day 3 Day 4 Day 5 Day 6 Day 8 Day 9 Day 11 Day 12 Day 14
D
S ORF3a E M ORF6 ORF7a ORF7b ORF8 N ORF10 Coverage
Normalized count
Coverage
Day 1 Day 2 Day 3 Day 4 Day 5 Day 6 Day 8 Day 9 Day 11 Day 12 Day 18
E
S ORF3a E M ORF6 ORF7a ORF7b ORF8 N ORF10 Coverage
Normalized count
Coverage
Day 1 Day 2 Day 3 Day 4 Day 5 Day 6 Day 8 Day 9
F
S ORF3a E M ORF6 ORF7a ORF7b ORF8 N ORF10 Coverage
Normalized count
Coverage
Day 1 Day 2 Day 3 Day 4 Day 5 Day 6 Day 8 Day 9
G
S ORF3a E M ORF6 ORF7a ORF7b ORF8 N ORF10 Coverage
Normalized count
Coverage
Day 1 Day 2 Day 3 Day 4 Day 5 Day 6 Day 8 Day 9
H
S ORF3a E M ORF6 ORF7a ORF7b ORF8 N ORF10 Coverage
Normalized count
Coverage
Day 1 Day 2 Day 3 Day 4 Day 5 Day 6 Day 8 Day 9

Figure 4

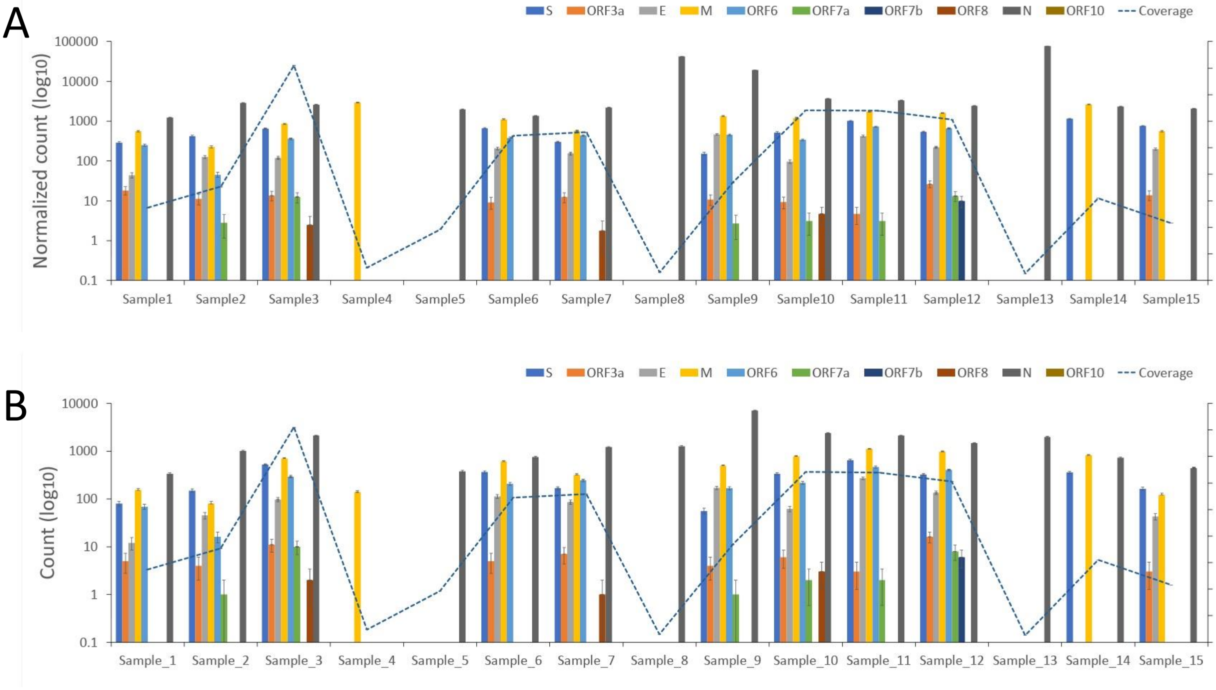
A
S
ORF3a
E
M
ORF6
ORF7a
ORF7b
ORF8
N
ORF10
Coverage
Normalized count (log10)
Coverage
Sample1
Sample2
Sample3
Sample4
Sample5
Sample6
Sample7
Sample8
Sample9
Sample10
Sample11
Sample12
Sample13
Sample14
Sample15
B
Count (log10)
Sample_1
Sample_2
Sample_3
Sample_4
Sample_5
Sample_6
Sample_7
Sample_8
Sample_9
Sample_10
Sample_11
Sample_12
Sample_13
Sample_14
Sample_15

Figure 5

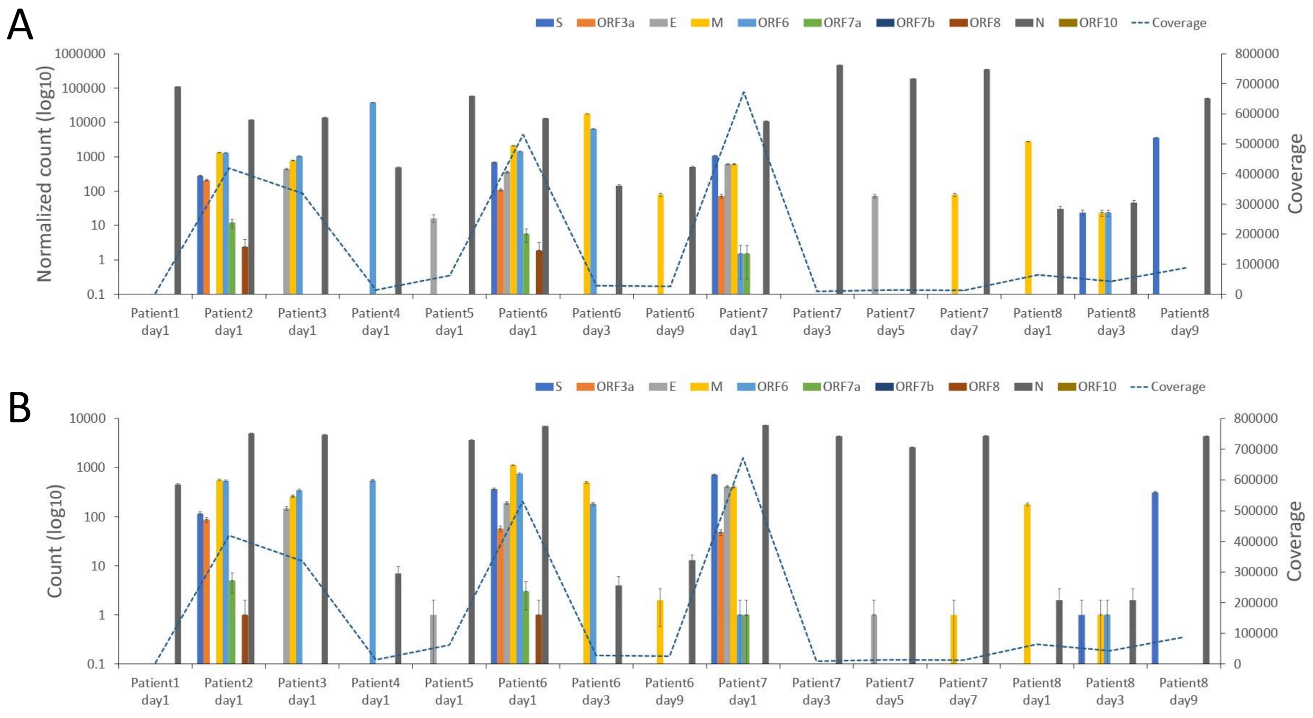
A
S ORF3a E M ORF6 ORF7a ORF7b ORF8 N ORF10 Coverage
Normalized count (log10)
1000000
100000
10000
1000
100
10
1
0.1
Coverage
800000
700000
600000
500000
400000
300000
200000
100000
0
Patient1 day1
Patient2 day1
Patient3 day1
Patient4 day1
Patient5 day1
Patient6 day1
Patient6 day3
Patient6 day9
Patient7 day1
Patient7 day3
Patient7 day5
Patient7 day7
Patient8 day1
Patient8 day3
Patient8 day9
B
S ORF3a E M ORF6 ORF7a ORF7b ORF8 N ORF10 Coverage
Count (log10)
10000
1000
100
10
1
0.1
Coverage
800000
700000
600000
500000
400000
300000
200000
100000
0
Patient1 day1
Patient2 day1
Patient3 day1
Patient4 day1
Patient5 day1
Patient6 day1
Patient6 day3
Patient6 day9
Patient7 day1
Patient7 day3
Patient7 day5
Patient7 day7
Patient8 day1
Patient8 day3
Patient8 day9

Figure 6

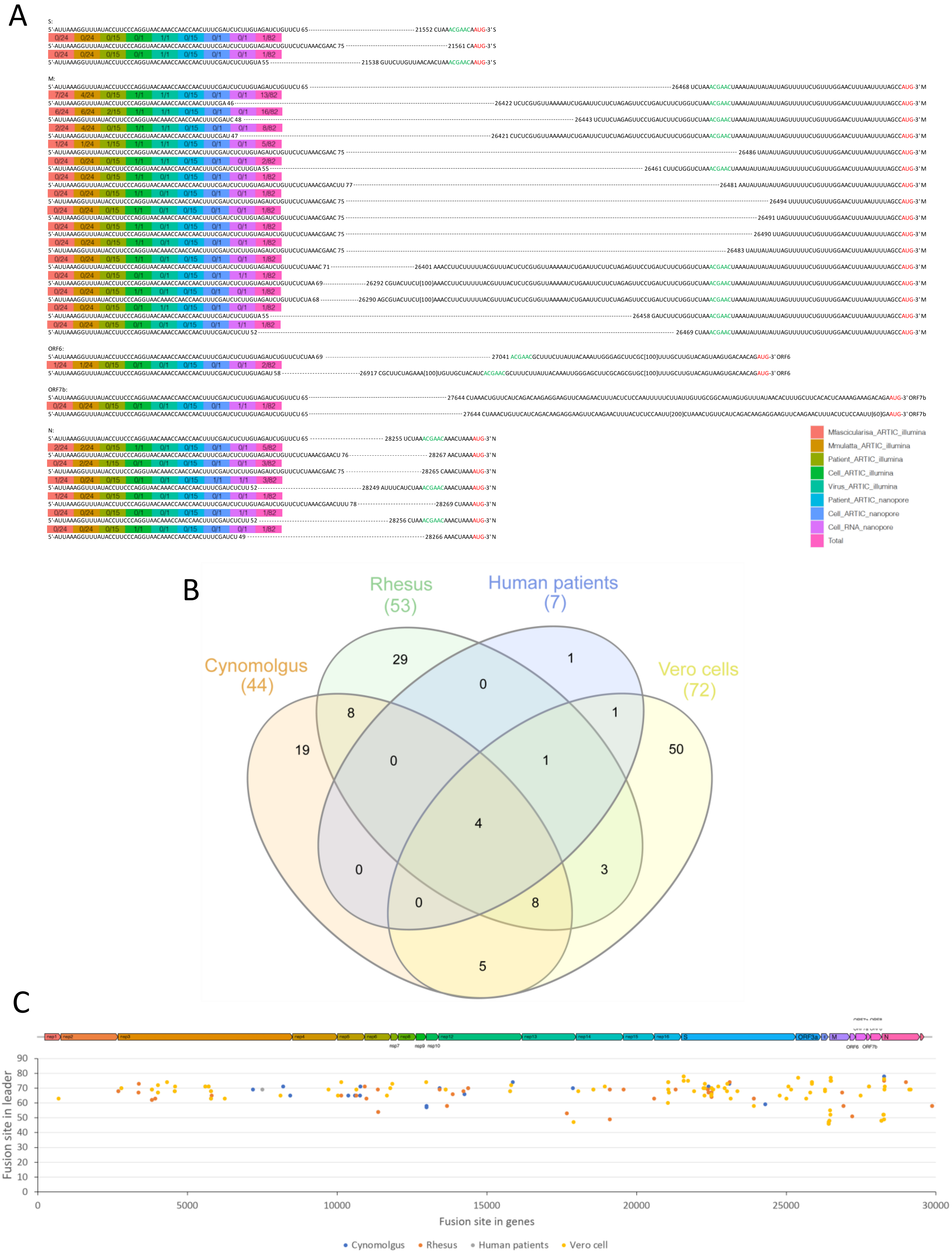
A
S:
M:
ORF6:
ORF7b:
N:
Mfascicularisa_ARTIC_illumina
Mmulatta_ARTIC_illumina
Patient_ARTIC_illumina
Cell_ARTIC_illumina
Virus_ARTIC_illumina
Patient_ARTIC_nanopore
Cell_ARTIC_nanopore
Cell_RNA_nanopore
Total
B
Rhesus (53)
Human patients (7)
Cynomolgus (44)
Vero cells (72)
29
1
0
8
1
19
0
1
50
4
0
3
0
8
5
C
Fusion site in leader
Fusion site in genes
Cynomolgus
Rhesus
Human patients
Vero cell

Figure 7

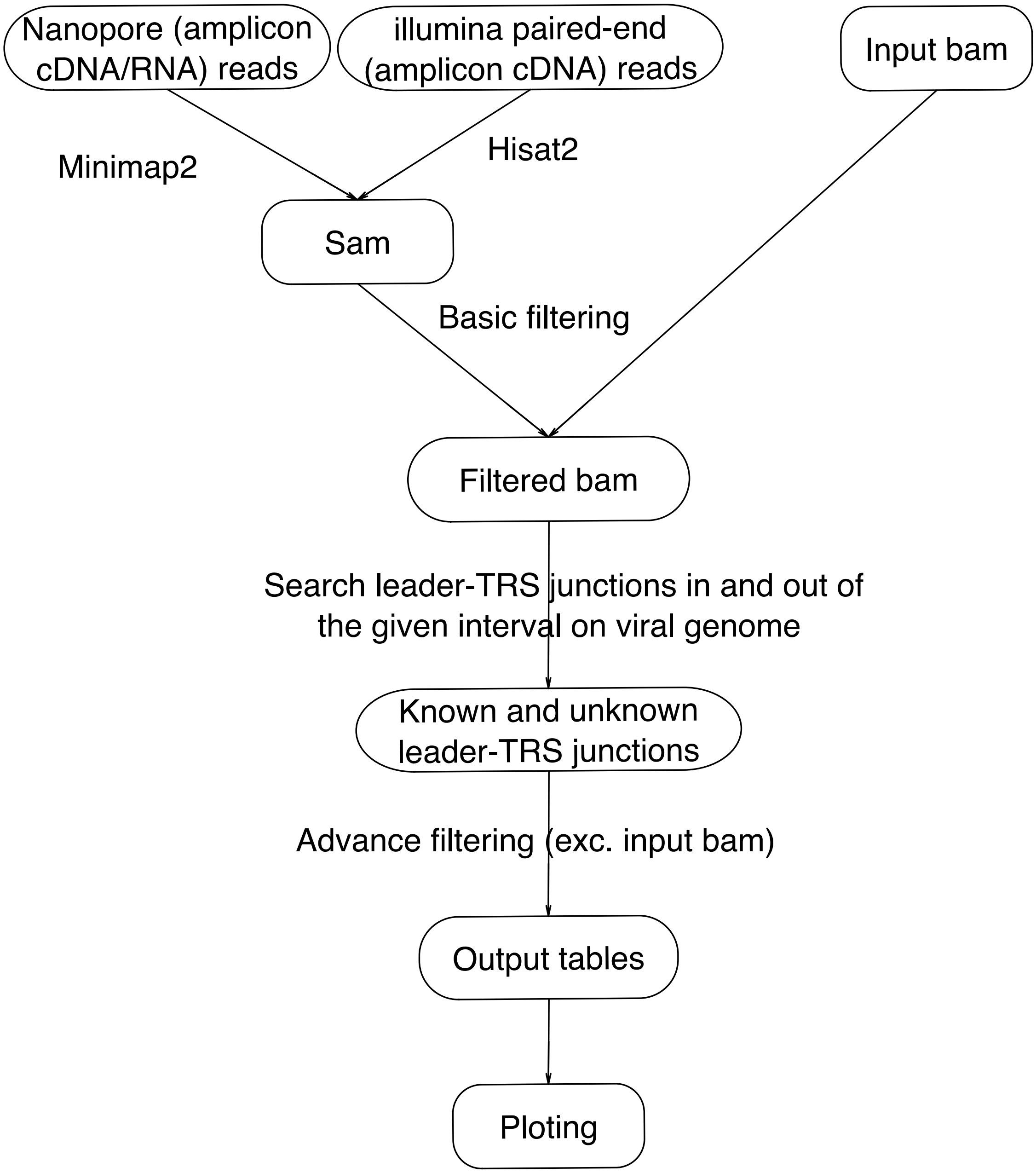

Supplementary Figure 1

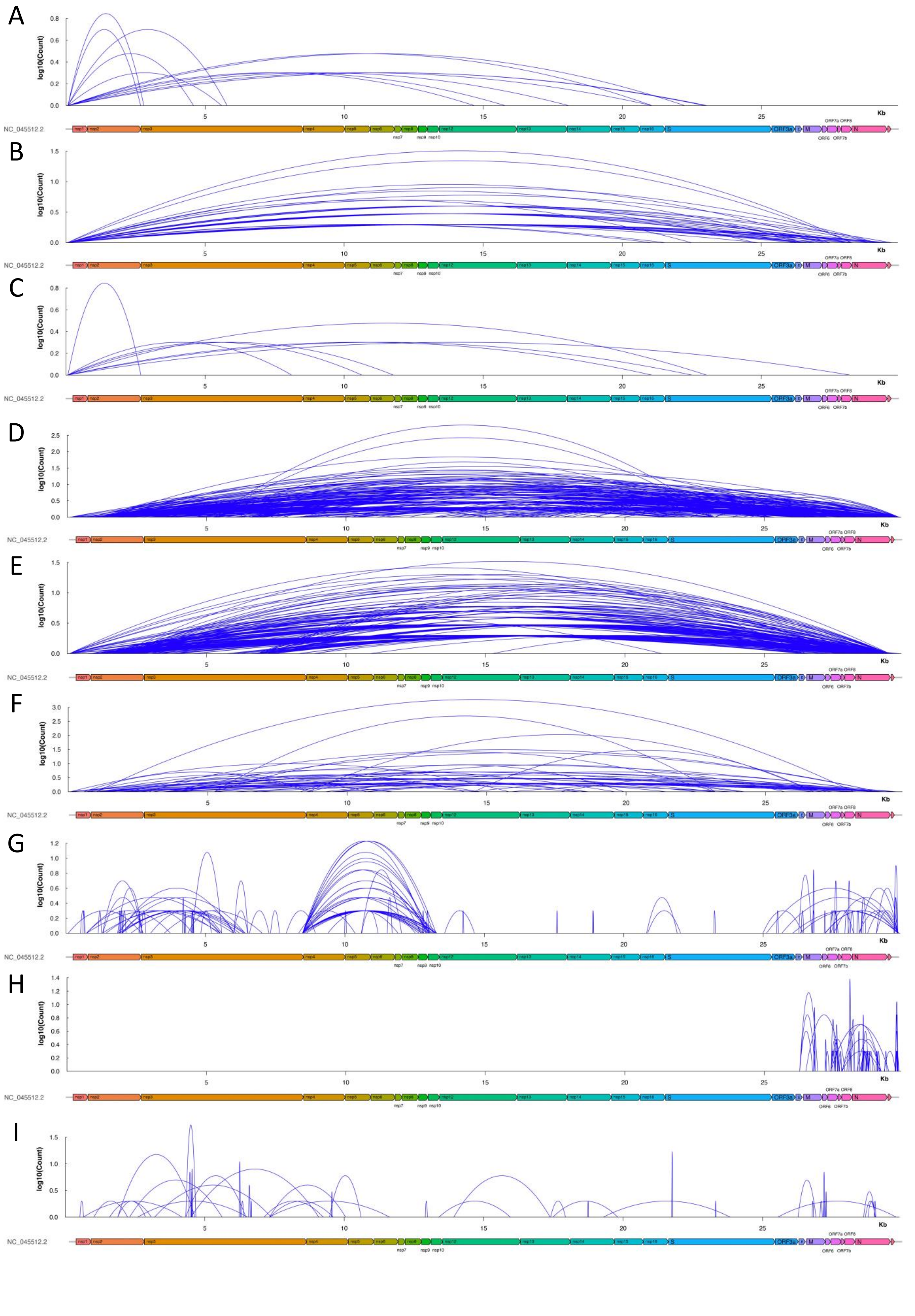

Supplementary Figure 2

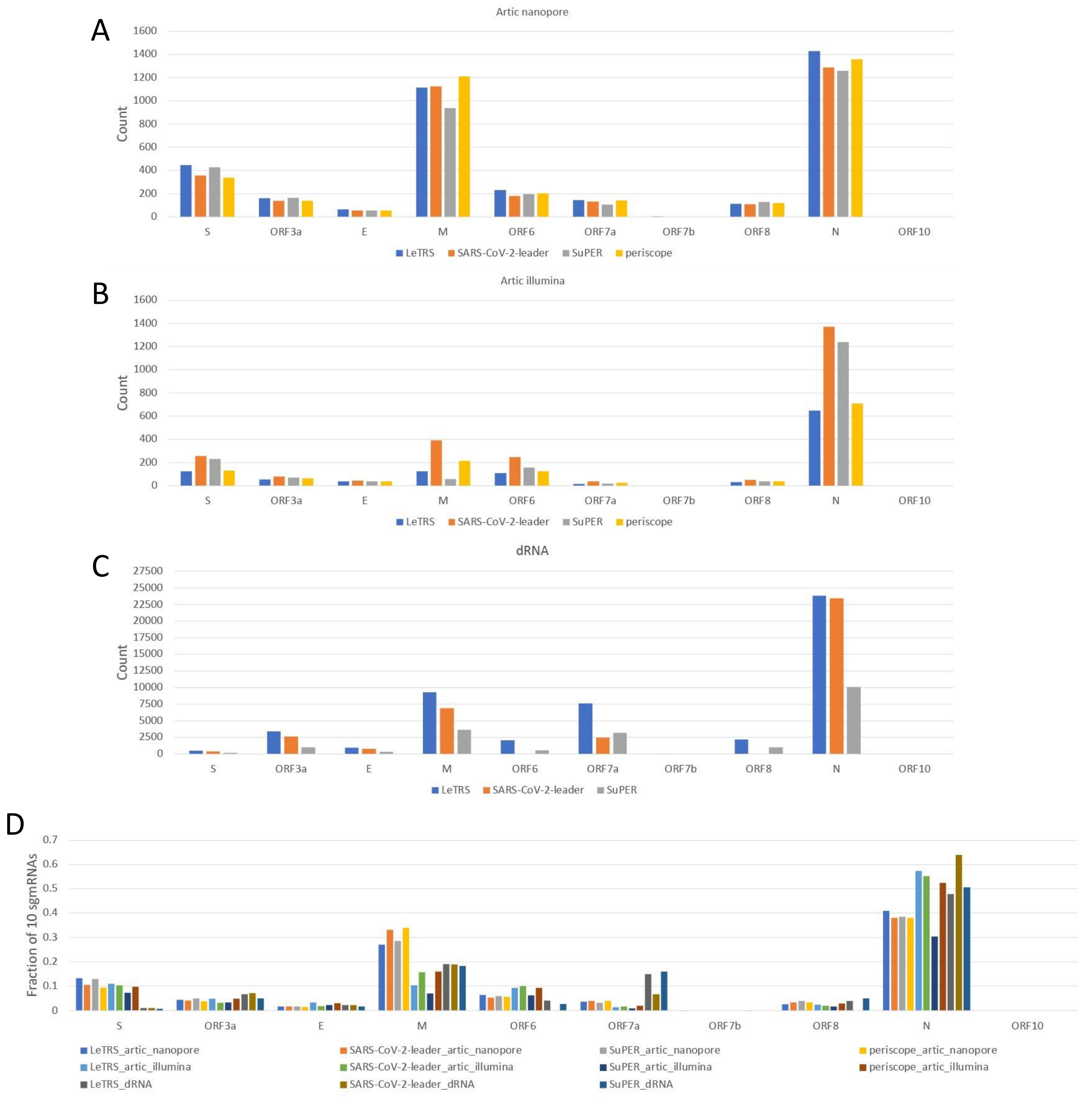

Supplementary Figure 3

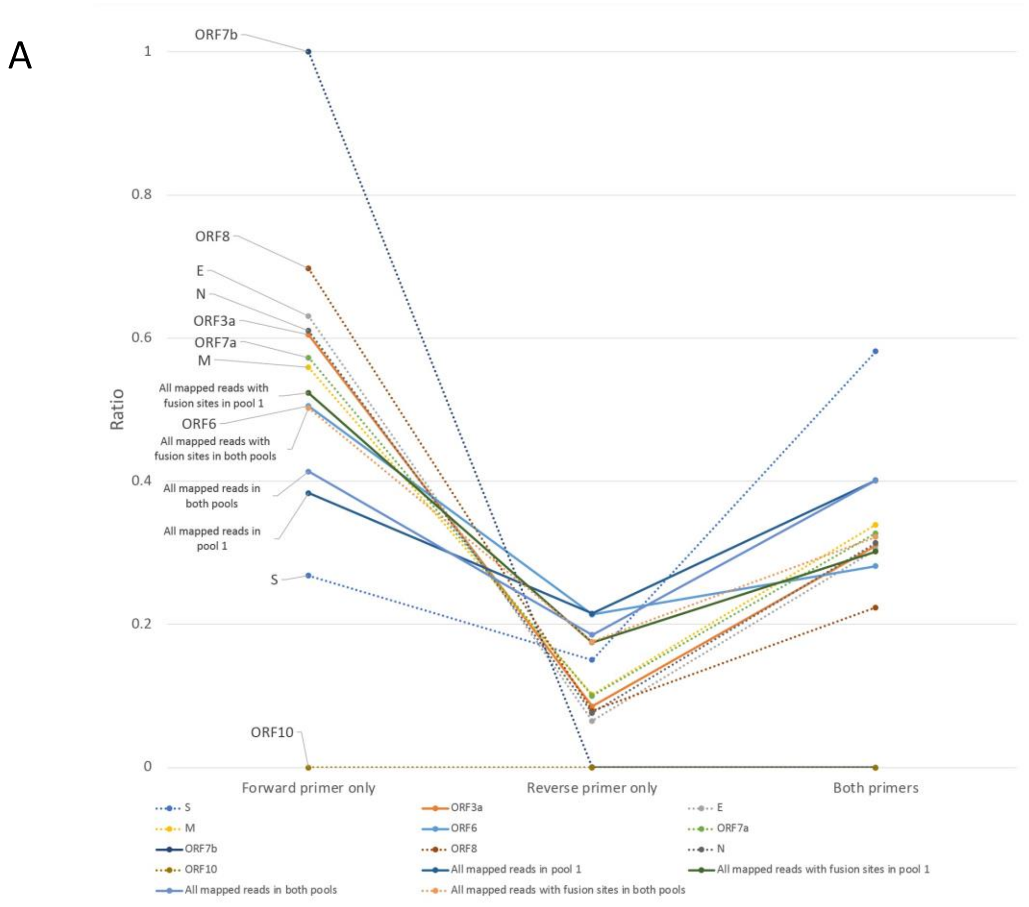

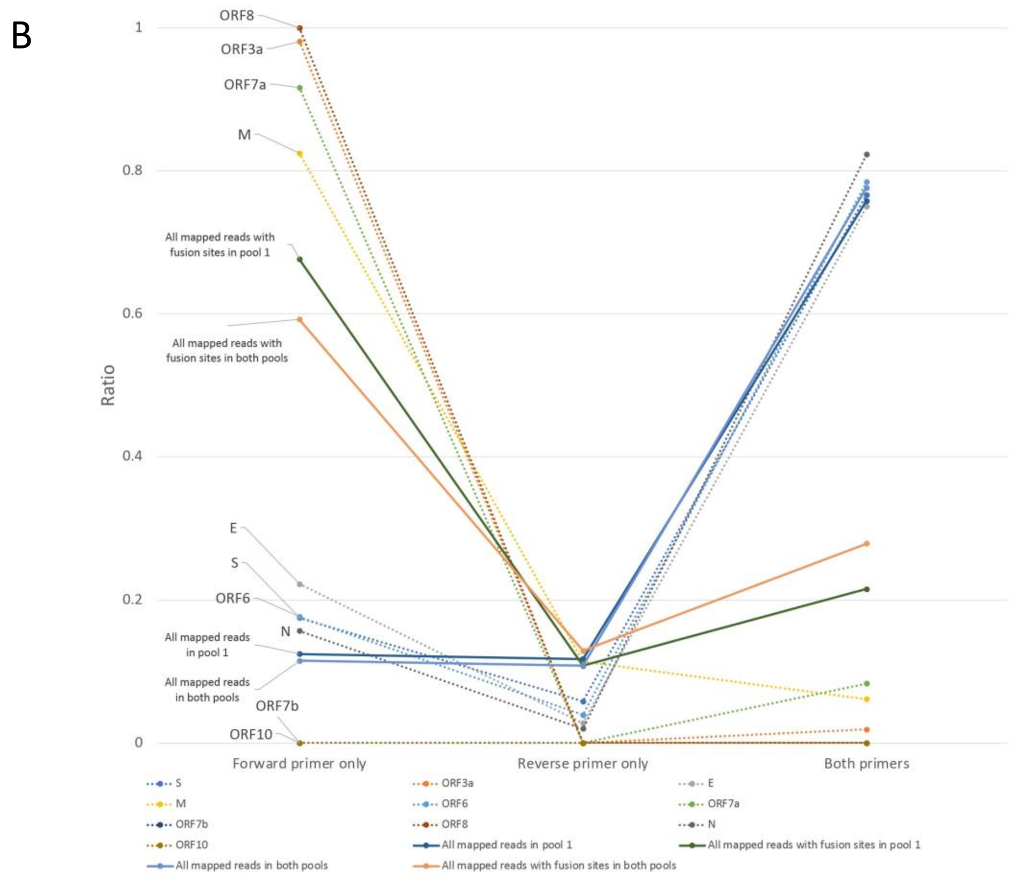

Supplementary Figure 4

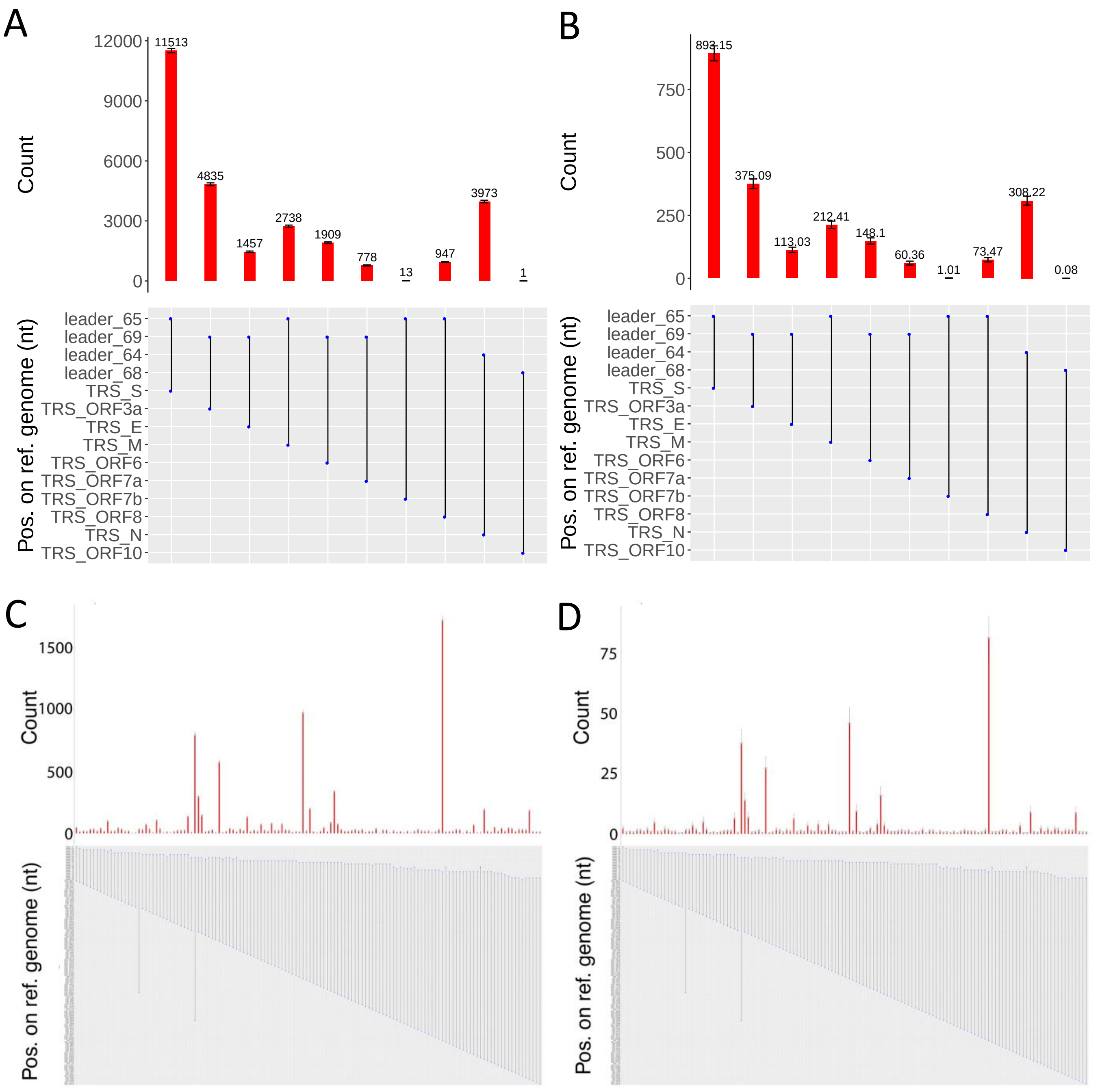

Supplementary Figure 5

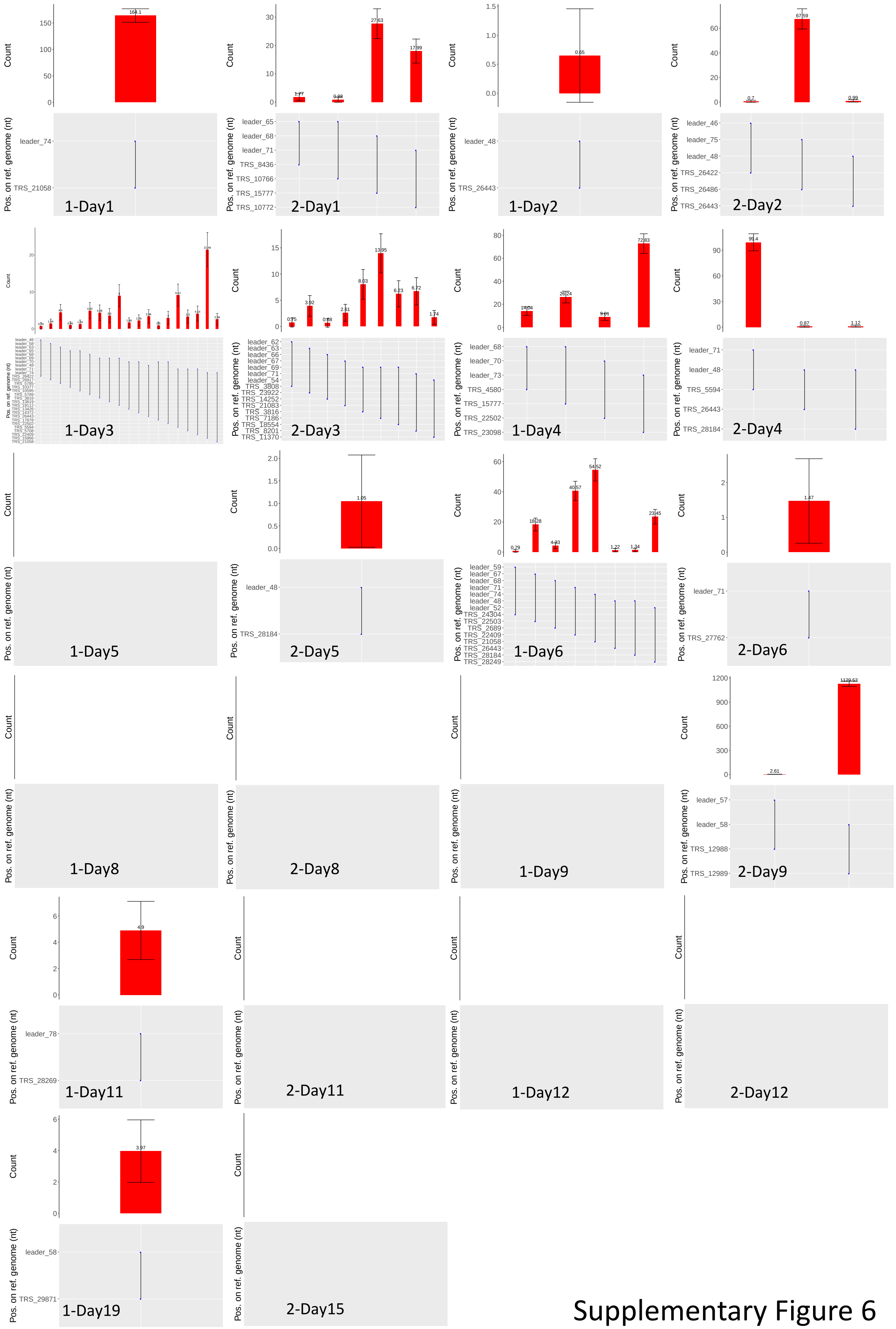

Supplementary Figure 6

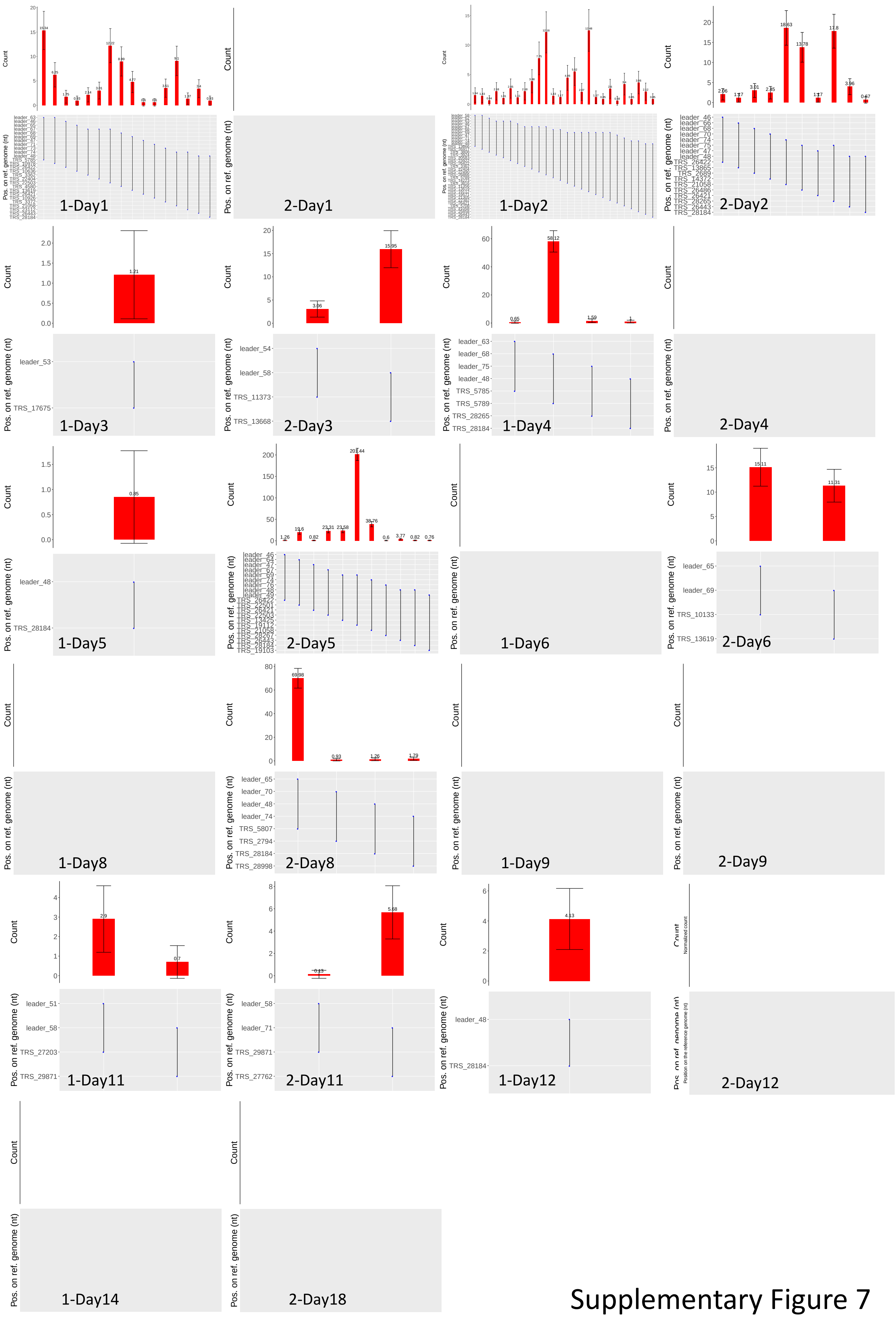

Supplementary Figure 7

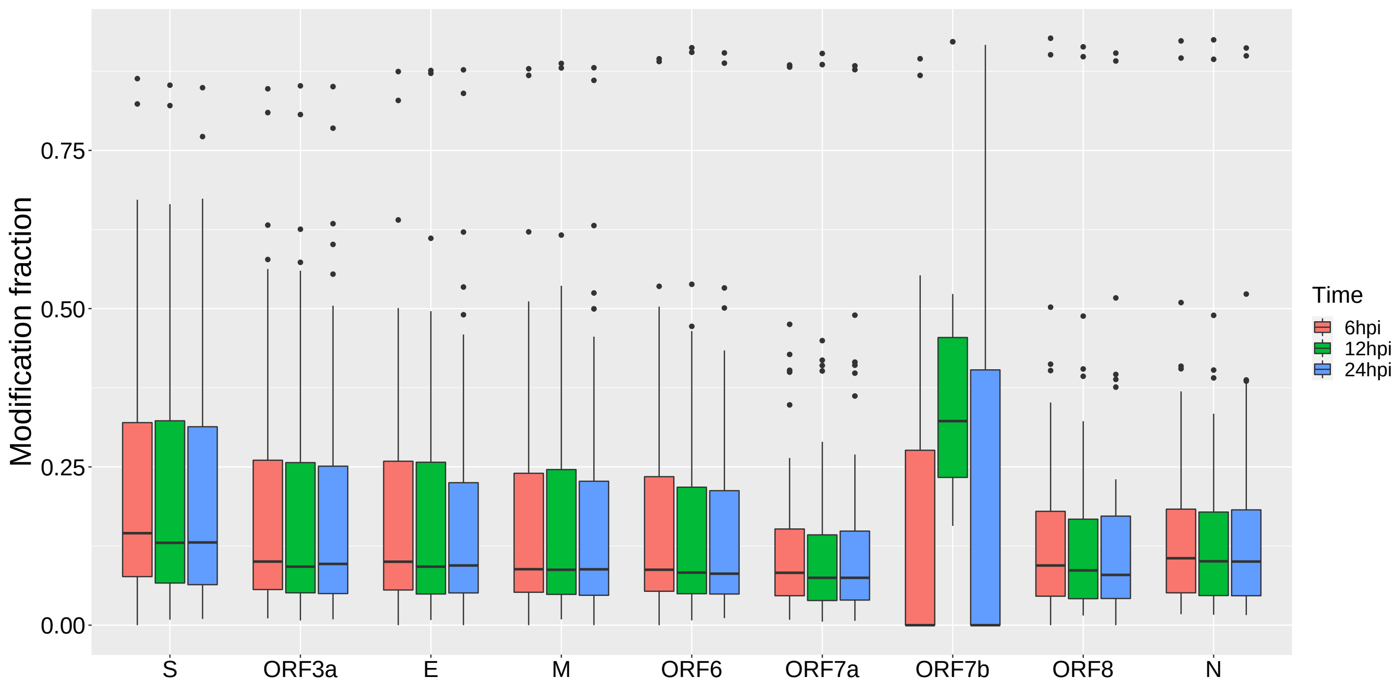

Supplementary Figure 8

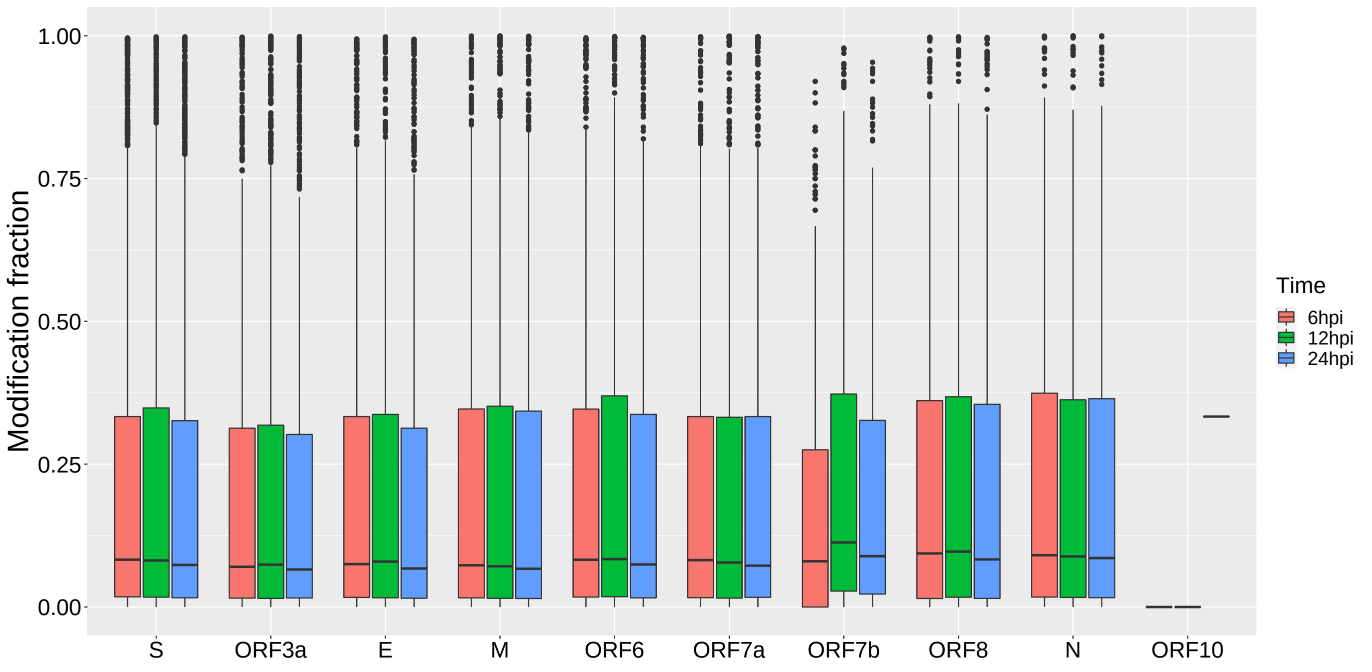

Supplementary Figure 9

Supplementary Table

Click here to access/download
Supplementary Material
Supplementary_Table_1.xlsx

Supplementary Table

Click here to access/download
Supplementary Material
Supplementary_Table_2.xlsx

Supplementary Table

Click here to access/download
Supplementary Material
Supplementary_Table_3.xlsx

Supplementary Table

Click here to access/download
Supplementary Material
Supplementary_Table_4.xlsx

Supplementary Table

Click here to access/download
Supplementary Material
Supplementary_Table_5.xlsx

Supplementary Table

Click here to access/download
Supplementary Material
Supplementary_Table_6.xlsx

Click here to access/download
Supplementary Material
Supplementary_Table_7.xlsx

Supplementary Table

Click here to access/download
Supplementary Material
Supplementary_Table_8.xlsx

Supplementary Table

Click here to access/download
Supplementary Material
Supplementary_Table_9.xlsx

Click here to access/download
Supplementary Material
Supplementary_Table_10.xlsx

Supplementary Table

Click here to access/download
Supplementary Material
Supplementary_Table_11.xlsx

Supplementary Table

Click here to access/download
Supplementary Material
Supplementary_Table_12.xlsx

Supplementary Table

Click here to access/download
Supplementary Material
Supplementary_Table_13.xlsx

Click here to access/download
Supplementary Material
Supplementary_Table_14.xlsx

Click here to access/download
Supplementary Material
Supplementary_Table_15.xlsx

reply of reviewers questions
Click here to access/download;Personal Cover;Reviewer_reports_letter_2nd.docx

Prof. Julian A. Hiscox

Chair in Infection and Global Health

Deputy Associate Pro-Vice Chancellor Research and Impact (FHLS)

The University of Liverpool
Department of Infection Biology
Institute of Infection, Veterinary and Ecological Sciences
Liverpool Science Park IC2
146 Brownlow Hill
Liverpool
L3 5RF

Tel: +44 (0)7812238359.
Email: julian.hiscox@liverpool.ac.uk

Dear GigaScience

Many thanks for reviewing our manuscript describing a bioinformatic tool we developed to study coronavirus biology, specifically demonstrated on clinical and model samples infected with SARS-CoV-2. We very much appreciate the very constructive and detailed reviews. Below we detail our point-by-point responses (in red) to the thoughts and suggestions of the reviewers (in black). We have acted on all these new comments and conducted the additional experiments that the reviewers wanted. We provide a marked-up manuscript of the initial revised version showing alterations from the original submitted version and a clean version with all changes etc accepted.

Yours sincerely,

Julian A. Hiscox.

Prof. Julian A. Hiscox.

Reviewer reports:

Reviewer #1: Comments: It is an important study. Except for a few minor points, the authors have addressed most of the reviewers' suggestions. This manuscript will be considered for acceptance after addressing the following minor suggestions:

1. The authors have compared the algorithm design, input, and output, and the counts of predicted sgmRNA across four tools. However, it would be nice if the authors could compare these tools' performances regarding prediction accuracy, F-measure, sensitivity, and specific scores. These will let the readers and potential users have a better sense of choosing a different tool for different purposes.

We have added the prediction accuracy, F-measure, sensitivity, and specific scores, calculated based on simulated Illumina and nanopore reads, in the Table 1.

2. It is unclear what the red line means in Supplemental Figure 8-9.

The red lines in Supplemental Figure 8 and 9 are for the normalized count of sgmRNA identified by LeTRS. We have moved this to Supplementary Table 12.

3. On page 18, lines 364-370. The analysis and significance that the authors stated in that paragraph do not show the apparent trends in Supplemental Figure 9. Would the authors update the figure types to reflect the results of their statistical tests?

We have updated the boxplots in Supplemental Figures 8 and 9. We used a paired samples one-sided Wilcoxon test that takes account the difference at each modification site of two compared sgmRNAs in different time points. A large amount of modification sites with differences resulted a low p-value even the trends in boxplots are not very large.

4. On page 18, line 370. The author mentioned that "The abundance of most sgmRNAs decreased with time, and both of these factors could account for the frequency of methylation." Based on the context, it seems that the conclusion could not be derived. Because the methylation frequency is a ratio, then it may not correlate with the abundance of the sgmRNAs.

We have removed this sentence to reflect the reviewer’s content.

Reviewer #2: Happy with revisions, no further comments
