# Supplementary material for: Analysis of SARS-CoV-2 known and novel subgenomic mRNAs in cell culture, animal model, and clinical samples using LeTRS, a bioinformatic tool to identify unique sequence identifiers
Source: Gigascience. 2022 May 26;11:giac045. doi: 10.1093/gigascience/giac045 (PMC9154083; doi:10.1093/gigascience/giac045)
Supplement: giac045_Response_to_Reviewer_Comments_Original_Submission [file giac045_response_to_reviewer_comments_original_submission.pdf]

Author's Response To Reviewer Comments

Close

Reviewer #1: Comments: In this manuscript, the authors sequenced the SARS-CoV-2 transcriptomes of nasopharyngeal samples from 15 patients using both illumina sequencing and nanopore ARTIC primer3 aplicom sequencing, and developed a computational-pipeline called LeTRS to identify the junctions between the leader sequences in the 5' end of viral genome and the transcriptional regulatory sequence (TRS) within the viral genome (leader-TRS-junction). They first tested and applied their LeTRS tool in several published Nanopore RNA-sequencing data and their own sequencing data to analyses leader-TRS sequence information. They showed that the expression abundance and populations of viral subgenomic mRNA (sgmRNAs) with leader-TRS varies along the time points of post-infection. This study is important to understanding SARS-CoV-2 pathology. However, this article needs many improvements. My major suggestions are as follows:

1. There are two types of leader sequences found in the SARS-CoV-2 sgmRNAs (Dongwan Kim et al., Cell 2020): leader with or without a TRS inside. In the current manuscript, the authors has used their LeTRS tool to identify the sgmRNAs with typical leader with TRS, but did not find the sgmRNAs with non-canonical leaders which do not include TRS inside (TRS-L-independent). I would suggest authors to further extend the studies to sgmRNAs with non-canonical leaders.
Of note, the junctions in these noncanonical transcripts are not derived from a known TRS-B. Some junctions show short sequences (3–4 nt) common between the 50 and 30 sites, suggesting a partial complementarity-guided template switching (‘‘polymerase jumping’’). However, the majority do not have any obvious sequences. Thus, we cannot exclude a possibility that at least some of these transcripts are generated through a different mechanism(s).

[Respond to comment 1: We have added a function in LeTRS to find sgmRNAs with non-conical leaders (TRS-L-independent) with the "-TRSLindependent" function. This function has been evaluated with the test sample (sequencing RNA from cells infected with SARS-CoV-2) as shown in Supplementary figure 2.]

2. SARS-CoV2 genomic and subgenomic mRNAs has multiple types of RNA modifications, such as m6A, 5mC, etc. These modifications has been shown to be regulated and relevant to their polyA tail lengths in sgmRNAs (Kim et al., Cell 2020). I would suggest authors to address if and how RNA modifications levels or types will be dynamically relevant to sgmRNA expression at different time points of post-infection. Aso any preference of RNA modifications in certain types of sgmRNAs (e.g. sgmRNA: S which encodes spike-proteins).

[Respond to comment 2: We have direct RNA sequenced the cell cultural samples infected with SARS-CoV-2 at three time points for investigating the relationship between RNA modifications to sgmRNA expression as shown in Supplementary Figures 8 and 9 and Supplementary Table 12. We specifically searched for two different types of methylation. We note that we can only sequenced RNA from cell culture using direct RNA sequencing on the Nanopore. We have found that RNA concentration and quality in clinical samples was insufficient for direct RNA sequencing.]

3. I would suggest the authors to compare and evaluate the performance of their LeTRS tools with other similar tools, such as SuPER (Yang Y. et al., Mol. Biol. Evol. 2020), and SARS-CoV-2-leader (Alexandersen S. et al., Nature Communications 2020), to discuss the strength and weakness of their tool, though the authors has compared their LeTRS tool with another one (Periscope).

[Respond to comment 3: We have compared LeTRS with the tools listed by the reviewer using our test data (total RNA from cells infected with SARS-CoV-2) sequenced by three different approaches –ARTIC-Nanopore, ARTIC-Illumina and direct RNA sequencing. This data is presented in Table 1 and Supplementary Figure 3 A, B, C and D. We compare and contrast what the different tools have in common in terms of analysis function and what data types they can function with.]

4. I would suggest the authors to re-analyze the public patient's seq data (NCBI PRJNA636225) to

examine if the same conclusion about the dysregulation of sgmRNAs at later time points could be derived in different groups of patients.

[Respond to comment 4: We have reanalyzed sequencing data from a longitudinal study in two patients (NCBI PRJNA636225) using LeTRSs. The results also indicated a dysregulation of sgmRNAs in late infection from the two patients (Supplementary Table 11). Apart from nuclease resistance and protection by cellular membranes, a phasic pattern of sgmRNA synthesis may also contribute to the presence of sgmRNAs at later time points.]

5. It would be nice to have a table to summary the samples and individual information in this study, such as clinical symptoms of patients, gender and age group, and sample collection time point after infection.

[Respond to comment 5: Due to the different pathways clinical samples were obtained patient identifying information was not available. For example, samples sequenced using ARTIC-Nanopore were obtained via ISARIC-4C and some patient information was obtained (likely due to these being hospitalized cases – either for treatment or isolation). This is shown in Supplementary Table 10. Samples sequencing using ARTIC-Illumina were sequenced under the auspices of COG-UK and identifying information was not available.]

6. The dataset ID provided by this paper (NCBI PRJNA699398) could not be found in the NCBI database. Please the authors address this problem and make the dataset available for the public with a correct ID.

[Respond to comment 6: There is a link provided for reviewers:
https://dataview.ncbi.nlm.nih.gov/object/PRJNA699398?reviewer=tro3da1gmld1kk6mdjndh7pg0o
We will release the data if the paper is accepted.]

7. The overall presentation, Figures, Tables and language of the paper could need some substantial improvement. The current manuscript includes many misused words, misused punctuation, grammatical errors, and mislabeling.
For examples:
(1) the title is too long. The author should conceive a title with concise but to the key-point.

[Respond to comment 7-1: We have shortened the title.]

(2) on page 4, the sentence "for SARS-CoV-2 the core motif is ACGAAC"could be revised as "The core motif of the TRS in SARS-CoV-2 is ACGAAC".

[Respond to comment 7-2: We have changed this.]

(3) on page 5, "cell infected in culture" is inaccurate. It could be expressed as "cultured cells with infection".

[Respond to comment 7-3: We have changed this.]

(4) on page 13, the word "commonality" might be replaced by "Common properties/features".

[Respond to comment 7-4: We have changed this.]

(5) the last sentence on page 13 also need language editing.

[Respond to comment 7-5: We have changed this.]

(6) on page 21, the subtitle "search leader-TRS" would be "searching leader-TRS". Pls keep the subtitle to be a short phrase, rather than beginning with a verb.

[Respond to comment 7-6: We have changed this.]

(7) pls keep the references in a consistent format. Pls correct the format of Ref. 26, 29 and 30 on page 25-26.

[Respond to comment 7-7: We have changed this.]

(8) The authors just need to acknowledge the COG-UK consortia and ISARIC4C consortia, rather than list names of all members in the consortia which occupy 8 pages' space.

[Respond to comment 7-8: We have removed these, apologies this was due to original rules around the consortium authorship statements/acknowledgements.]

(9) The x or y bar label and scales in most figures/suppl figures are too small to read.

[Respond to comment 7-9: We have increased the font on the labels.]

(10) The Figure legends of all figures are not clear enough and does not provide enough illustrations and explanations for the figures (e.g. Fig 1).

[Respond to comment 7-10: We have changed and expanded the Figure legends.]

(11) Supplemental Fig1 could be re-designed to be more clear. For instance, the authors can merge the same steps after the step of or , to avoid redundant information.

[Respond to comment 7-11: We have changed this.]

(12) The legend of table 8 seems exactly same as the legend of table 2. Pls check it.

[Respond to comment 7-12: We have changed this, Tables 1-8 have been moved to Supplementary Tables 1, 2 and 3.]

Reviewer #2: "Identification and quantification of SARS-CoV-2 leader subgenomic mRNA gene junctions in nasopharyngeal samples shows phasic transcription in animal models of COVID-19 and dysregulation at later time points that can also be identified in humans"

In this paper, Dong et al describe a new pipeline for identifying subgenomic mRNA from multiple types of sequence data, including amplicon (Illumina and Nanopore) as well as long read nanopore direct RNA or cDNA sequencing. It is useful to have a bioinformatics pipeline which can rapidly identify sgRNA in multiple types of sequence data and has the potential to open large amplicon datasets in particular for further analysis of sgRNA abundance. However, I believe that more validation of the accuracy of abundance estimates from amplicon data is required in order to give the research community more confidence in its use (and limitations).

Major comments:

1. More explanation/detail on methodology would be useful. The authors say that they find the most common peak for the break points of the disjunction site amongst all reads with a break point within a 20bp window of the expected breakpoint. Is there a threshold applied in terms of the difference between the most common and next-most-common breakpoint? Also for the novel sites, is there a clustering algorithm applied, or any site with more than 10 reads is reported?

[Respond to comment 1: We used the 20bp window (±10bp) of the true splicing sites (known) splicing sites for searching the known sgmRNAs. As noted in the manuscript although we refer to splicing – this is a fusion event. As the minimap2 paper indicated "When INDEL sequencing errors are frequent, it is difficult to find precise splicing sites in this case. If we allow up to 10 bp distance from true splicing sites, 98.4% of aligned introns are approximately correct." (https://doi.org/10.1093/bioinformatics/bty191). Because the known breakpoints are far from each other, the threshold was not defined between the most common and next-most-common breakpoint for the known breakpoints.
We used the coverage cut-off (>10 by default) for the novel sites because we found the novel sites usually have low sequence coverage and don't have a cluster like the known sgmRNAs. Alternatively, these novel sites could be due to RT and sequencing errors, and we note this in the manuscript. LeTRS reports these unknown sites as potential novel sites for future research as all other novel sgmRNAs in the research data.]

2. I would like a more direct comparison of sgRNA abundances estimated from amplicon based approach, vs using nanopore amplicon free approach? Its possible to do this only by comparing different tables. It would be easier to digest if there was a x-y plot comparing abundances from different approaches on the same sample. This would help give confidence that the amplicon based approach can provide good estimates. From looking at the tables 1 and 2, it seems that the amplicon approach estimates a lot less sgRNA than the amplicon free approach overall (in terms of normalized counts per million mapped reads). This is to be expected as most of the reads from the amplicon sequencing would be expected to come from the genome. It would be good to see which ORFs are under- and over-represented in the amplicon data, as I imagine this would also relate to which primer pairs are in the same amplicon pool in the arctic design.
Related to this, it would be good to have an analysis of how the primer design impacts detection of sgRNA. For example, I thought that only one of the primer pools includes a leader primer.

[Respond to comment 2 part 1: To address this question we infected cells in culture with SARS-CoV-2 and sequenced the viral RNA using three different approaches. Two were amplicon based – based around the ARTIC protocol (an amplicon based system) and also by direct RNA sequencing. This data is shown in Supplementary Tables 1, 2 and 3 to replace the old test data in the Tables 1-8.
With the Artic V3 pipeline, we used two primer pools for the PCR reactions in the whole virus amplification. Please find the primers used in the primer pool 1 and pool 2 at https://github.com/artic-network/artic-ncov2019/blob/master/primer_schemes/nCoV-2019/V3/nCoV-2019.tsv. For the Artic V3 pipeline, only the pool 1 includes a 5'(forward) primer located within the leader region (about < 80) on the genome (please find the position of Artic V3 primers on the virus genome at https://github.com/artic-network/artic-ncov2019/blob/master/primer_schemes/nCoV-2019/V3/nCoV-2019.primer.bed). The LeTRS (v2.0.1) has been modified to only identify the reads with primers in the pool 1, pool 2 or both pools. We compared the read counts evaluated by LeTRS in both ARTIC-Nanopore and ARTIC-Illumina test data for pool 1 and 2, and found only very few reads/read pairs contained the reverse primers with primer pool 2 (Supplementary Table 4 and 5), suggesting the primers in Artic pool 2 are almost not involved the sequencing of leader-TRS regions.
We have done the x-y plot as showed in Figure 3A and C for the reads with at least a primer sequence comparing abundances from different approaches on the same sample. The normalized counts showed a linear relationship between the amplicon based method to the direct sequencing method, while The Artic-Nanopore and Artic-Illumina showed same ratio of known sgmRNA as the nanopore direct RNA sequencing approach, except S and orf7a (Figure 3B and D for the reads with at least a primer sequence). This suggested an amplicon based approach can provide good estimates for most of the sgmRNAs, especially for N. This normalization method has been applied by https://doi.org/10.1101/gr.268110.120 and https://doi.org/10.1038/s41467-020-19883-7.
PCR based approaches boosted value of denominator reduced the normalized count because a full length of mRNA is counted once with direct RNA sequencing approach will be counted many times with its the small amplicons. Artic illumina got even smaller normalized counts than Artic nanopore approach due to the probably the sequencing bias of illumina during bridge PCR (https://doi.org/10.1186/gb-2013-14-5-r51). Therefore, the normalized counts can only be used for the comparison of samples sequenced by same approach when that resulted same PCR and sequencing machine effects. The difference of normalized counts in the samples from amplicon based methods only indicate the relative difference.]

Further related to this, it would be good to have a plot which shows the proportion of read counts which are derived from left-primer only, right-primer only or both primers for each sgRNA, and how this compares to the overall ratio of left-only and right-only primers. It seemed odd to me at first glance that there are so many one-sided amplifications, but I imagine this is a small proportion overall, but a sizeable proportion of the reads which can identify sgRNA, due to the lack of primer pairs for many of the sgRNA. Based on this analysis, it would also be interesting to estimate what is the best depth of coverage of the amplicon panels to get reliable estimates of sgRNA abundance across the different ORFs.

[Respond to comment 2 part 2: We compared the ratio of reads with forward primers only and reverse primers only and both primers for each sgmRNAs to the overall ratios of reads with forward primers only and reverse primers only and both primers in all mapped reads of pool 1 and pool 2 and the mapped reads with any fusion sites in pool 1 and pool 2, found overall ratios showed abundant reads showed same pattern as the reads for sgmRNAs (Supplementary Figure 4). This suggested the mass of one side amplification is a nature of amplicon sequencing.]

3. It would be good to compare the novel breakpoints with those previously reported, e.g. in Taiorara et al, figure 2 and supplementary figure 6 (https://doi.org/10.1101/2020.03.05.976167). I can see that many of them line up with those you report in table 4, and I believe this sup

[Respond to comment 3: Taiorara et al didn’t attach the exact breakpoints positions with their figure, but we generated a similar figure for comparison (Figure 7c). Figure 7c showed some similar breakpoints positions with Figure 2 of Taiorara et al’s paper.]

4. Is there much overlap in the novel break points detected using nanopore amplicon ARTIC v3 vs nanopore dRNA? It would be good to have an extra column in Table 8 and table 4 indicating which of the breakpoints discovered in dRNA were also discovered in amplicon sequencing and vice versa. This will hopefully shed light on relative strengths of the two approaches. Similarly it would be useful to compare nanopore ARTIC and illumina ARTIC in this regard

[Respond to comment 4: As described above we have moved the new test data from a unique cell culture sample to Supplementary Tables 1, 2 and 3 for Artic-Nanopore, Artic-Illumina and nanopore direct RNA sequencing. We didn’t find any exactly the same novel fusion sites in these three approaches. To note in the publication describing minimap2 the paper details “In general, minimap2 is more consistent with existing annotations. It finds more junctions with a higher percentage being exactly or approximately correct” and “When INDEL sequencing errors are frequent, it is difficult to find precise splicing sites in this case. If we allow up to 10 bp distance from true splicing sites, 98.4% of aligned introns are approximately correct.” (https://doi.org/10.1093/bioinformatics/bty191). Therefore, it is very difficult to identify the exact novel fusion sites. Novel leader-TRS junctions were also known as leader dependent noncanonical fusions. LeTRS also has a function to identify leader independent long-distance (>5,000 nt) fusion and local joining yielding a deletion between proximal sites (20–5,000 nt distance) in the sequencing reads. If we look at the pattern of the fusion sites, some of the novel leader-TRS junctions (noncanonical fusions) and leader independent fusions in the test sample were supported by all three sequencing methods (Supplementary figure 2) with similar fusion sites.
The strength of LeTRS to identify the known breakpoints is much stronger than identifying novel sites, because LeTRS controls the aligner to search the known breakpoints with the guide of known annotations. As the paper said “In general, minimap2 is more consistent with existing annotations. It finds more junctions with a higher percentage being exactly or approximately correct” (https://doi.org/10.1093/bioinformatics/bty191).]

5. Its hard to assess the evidence supporing the biphasic expression without having some idea of the error in the abundance estimates (also commented on this more below);

[Respond to comment 5: We have calculated the standard deviation of a binomial distribution as error bar. The data supports that biphasic expression/abundance of sgmRNAs occurs.]

6. The conclusion of dysregulation in samples taken from patients many days into their infection is made only on a small number of samples. Also in Figure 4, the time post sample is not indicated. I presume the information is in one of the supplementary tables, but the submitted pdf has messed up these tables (its somewhere in the 729 page pdf) . Nevertheless, it seems that the data supporting this conclusion is a bit thin, and I would be cautious in including that observation in the title of the paper.

[Respond to comment 6: We have changed the title to reflect this comment.]

Minor comments:

1. In figure legends (e.g. figure 1) you say the numbers in brackets are:reads with left primers, reads with right primers, reads with both primers. I can see from the numbers that these are not exclusive, but it might be easier to digest if you showed left-only, right-only and both

[Respond to minor comments 1: We modified the LeTRS to show forward-only, reverse-only and both primers.]

2. You make a point in the paper about whether the left break occurs at position 64 or 69. One thing I would worry about is that microhomology between TRS-L and TRS-R might make it difficult to be exactly sure of the breakpoint (because the sgRNA includes only one copy of the TRS, but its hard to know if it's the left or the right which is included, the aligner could equally well align to TRS-L and skip TRS-R or vice versa, and this would shift the coordinates slightly. Are the enough snp differences in TRS-L or TRS-R to be confident either way, and if so, does this have implications for whether TRS-L or TRS-R is retained in the sgRNA?

[Respond to minor comments 2: For the known sgmRNA, we used the known annotation of breakpoints to guide the alignments and allowing a (±10bp) window of the true splicing/fusion sites for searching the breakpoints - if this would shift the coordinates slightly. Even if TRS-L or TRS-R is retained in the sgmRNAs, the implications will be random and equal to all samples with same sequencing approach and alignment tool. This should not affect the evaluation of the ratio of sgmRNAs and relative abundance across samples. We have also compared the number of reads for sgmRNAs with the other methods (tool called SARS-CoV-2-leader) that is to search a tag sequence within leader in reads but not the breakpoints of reads. SARS-CoV-2-leader produced a similar read count as LeTRS for the Artic-Nanopore (Supplementary Figure 3A) and Nanopore direct sequencing (Supplementary Figure 3C). SARS-CoV-2-leader produced more counts than LeTRS for Artic-Illumina, because LeTRS counts the read pairs but not reads (Supplementary Figure 3B). There are difficulties in searching for novel breakpoints, although we treat novel breakpoints as a potential sign of novel sgmRNAs for future research.]

3. Figure 1 panels B,C,D were a bit confusing. Why is the reference sequence in the middle. It would be good if the caption could be expaned to help the reader understand these panels in particular.

[Respond to minor comments 3: The figure legend has been changed but we would like to keep the reference sequence in the middle to show the forward and reverse amplification possibilities.]

4. The tables (table 1 to 8) and the figure 1A represent a lot of the same information, but the numbers don't line up exactly, because in the figures you only use counts which have both primers. It would be best to decide which to represent because it's confusing to have the same data presented twice essentially but in slightly different ways.

[Respond to minor comments 4: We have changed this and now consistently only used the reads containing at least one primer to plot data.]

5. In figure 1 you present the normalized abundance to 2 decimal places, but its very unlikely that you have that level of precision. It would be good if you could add error bars to estimate the uncertainty in the abundance estimate (e.g. calculated using a binomial distribution).

[Respond to minor comments 5: We have calculated the standard deviation of a binomial distribution as an error bar.]

6. In figure 3, its hard to know how much error there is in each of the measurements. By showing the normalized value, its also hard to see what is the absolute change in the read counts. Ideally you would show either the read counts, or show error bars around the abundance estimates.

[Respond to minor comments 6: We now show error bars.]

7. Is there a mistake in the title of Table 8: "The LeTRS output table for novel sgmRNA in the tested Nanopore ARTIC v3 primers." Because the title of table 2 seems the same: Table 2. The LeTRS output table for novel sgmRNA in the tested Nanopore ARTIC v3 primers". One of these approaches does not seem to find novel breakpoints, but the other does, presumably Table 8 should be illumina based on the ordering?

[Respond to minor comments 7: We have changed this. Tables 1-8 have been moved to Supplementary Tables 1, 2 and 3.]

8. Error in caption of table 1: " Normalized count=(Read count-Total number of read mapped on reference genome)*1000000"

[Respond to minor comments 8: We have changed this. Tables 1-8 have been moved to Supplementary Tables 1, 2 and 3.]

9. In the supplementary figures, the captions you saay:" Supplementary Figure 3. Raw (A and C) and normalised (B and D) expected (upper) and novel (lower) leader-TRS gene junctions count in the infecting SARS-CoV-2 inoculum source used for NHP study, sequenced by Illumina ARTIC method (Supplementary Table 8)."
I found the use of "expected" here confusing, because it implied to me that you had estimated expected counts. I would prefer the use of the term canonical, or something like that.

[Respond to minor comments 9: We have changed "expected" to “canonical”. Supplementary Figure 3 has become Supplementary Figure 5.]

Close
