# Supplementary material for: Analysis of SARS-CoV-2 known and novel subgenomic mRNAs in cell culture, animal model, and clinical samples using LeTRS, a bioinformatic tool to identify unique sequence identifiers
Source: Gigascience. 2022 May 26;11:giac045. doi: 10.1093/gigascience/giac045 (PMC9154083; doi:10.1093/gigascience/giac045)
Supplement: giac045_Response_to_Reviewer_Comments_Revision_1 [file giac045_response_to_reviewer_comments_revision_1.pdf]

Author's Response To Reviewer Comments

Close

Reviewer reports:
Reviewer #1: Comments: It is an important study. Except for a few minor points, the authors have addressed most of the reviewers' suggestions. This manuscript will be considered for acceptance after addressing the following minor suggestions:

1. The authors have compared the algorithm design, input, and output, and the counts of predicted sgmRNA across four tools. However, it would be nice if the authors could compare these tools' performances regarding prediction accuracy, F-measure, sensitivity, and specific scores. These will let the readers and potential users have a better sense of choosing a different tool for different purposes.

[We have added the prediction accuracy, F-measure, sensitivity, and specific scores, calculated based on simulated Illumina and Nanopore reads, in the Table 1.]

2. It is unclear what the red line means in Supplemental Figure 8-9.

[The red lines in Supplemental Figure 8 and 9 are for the normalized count of sgmRNA identified by LeTRS. We have moved this to Supplementary Table 12.]

3. On page 18, lines 364-370. The analysis and significance that the authors stated in that paragraph do not show the apparent trends in Supplemental Figure 9. Would the authors update the figure types to reflect the results of their statistical tests?

[We have updated the boxplots in Supplemental Figures 8 and 9. We used a paired samples one-sided Wilcoxon test that takes account the difference at each modification site of two compared sgmRNAs in different time points. A large amount of modification sites with differences resulted a low p-value even the trends in boxplots are not very large.]

4. On page 18, line 370. The author mentioned that "The abundance of most sgmRNAs decreased with time, and both of these factors could account for the frequency of methylation." Based on the context, it seems that the conclusion could not be derived. Because the methylation frequency is a ratio, then it may not correlate with the abundance of the sgmRNAs.

[We have removed this sentence to reflect the reviewer’s content.]

Reviewer #2: Happy with revisions, no further comments

Close
