# Supplementary material for: Analysis of SARS-CoV-2 known and novel subgenomic mRNAs in cell culture, animal model, and clinical samples using LeTRS, a bioinformatic tool to identify unique sequence identifiers
Source: Gigascience. 2022 May 26;11:giac045. doi: 10.1093/gigascience/giac045 (PMC9154083; doi:10.1093/gigascience/giac045)
Supplement: giac045_Reviewer_1_Report_Original_Submission — Chan Zhou -- 6/17/2021 Reviewed [file giac045_reviewer_1_report_original_submission.pdf]

**Reviewer Report**

**Title: Analysis of SARS-CoV-2 known and novel subgenomic mRNAs in cell culture, animal model and clinical samples using LeTRS, a bioinformatic tool to identify unique sequence identifiers.**

**Version: Original Submission** **Date:** 6/17/2021

**Reviewer name: Chan Zhou**

**Reviewer Comments to Author:**

Comments: In this manuscript, the authors sequenced the SARS-CoV-2 transcriptomes of nasopharyngeal samples from 15 patients using both illumina sequencing and nanopore ARTIC primer3 aplicom sequencing, and developed a computational-pipeline called LeTRS to identify the junctions between the leader sequences in the 5' end of viral genome and the transcriptional regulatory sequence (TRS) within the viral genome (leader-TRS-junction). They first tested and applied their LeTRS tool in several published Nanopore RNA-sequencing data and their own sequencing data to analyses leader-TRS sequence information. They showed that the expression abundance and populations of viral subgenomic mRNA (sgmRNAs) with leader-TRS varies along the time points of post-infection. This study is important to understanding SARS-CoV-2 pathology. However, this article needs many improvements. My major suggestions are as follows:

1. There are two types of leader sequences found in the SARS-CoV-2 sgmRNAs (Dongwan Kim et al., Cell 2020): leader with or without a TRS inside. In the current manuscript, the authors has used their LeTRS tool to identify the sgmRNAs with typical leader with TRS, but did not find the sgmRNAs with non-canonical leaders which do not include TRS inside (TRS-L-independent). I would suggest authors to further extend the studies to sgmRNAs with non-canonical leaders.
2. SARS-CoV2 genomic and subgenomic mRNAs has multiple types of RNA modifications, such as m6A, 5mC, etc. These modifications has been shown to be regulated and relevant to their polyA tail lengths in sgmRNAs (Kim et al., Cell 2020). I would suggest authors to address if and how RNA modifications levels or types will be dynamically relevant to sgmRNA expression at different time points of post-infection. Aso any preference of RNA modifications in certain types of sgmRNAs (e.g. sgmRNA: S which encodes spike-proteins).
3. I would suggest the authors to compare and evaluate the performance of their LeTRS tools with other similar tools, such as SuPER (Yang Y. et al., Mol. Biol. Evol. 2020), and SARS-CoV-2-leader (Alexandersen S. et al., Nature Communications 2020), to discuss the strength and weakness of their tool, though the authors has compared their LeTRS tool with another one (Periscope).
4. I would suggest the authors to re-analyze the public patient's seq data (NCBI PRJNA636225) to examine if the same conclusion about the dysregulation of sgmRNAs at later time points could be derived in different groups of patients.
5. It would be nice to have a table to summary the samples and individual information in this study, such as clinical symptoms of patients, gender and age group, and sample collection time point after infection.
6. The dataset ID provided by this paper (NCBI PRJNA699398) could not be found in the NCBI database. Please the authors address this problem and make the dataset available for the public with a correct ID.

7. The overall presentation, Figures, Tables and language of the paper could need some substantial improvement. The current manuscript includes many misused words, misused punctuation, grammatical errors, and mislabeling.
For examples:
(1) the title is too long. The author should conceive a title with concise but to the key-point.
(2) on page 4, the sentence "for SARS-CoV-2 the core motif is ACGAAC"could be revised as "The core motif of the TRS in SARS-CoV-2 is ACGAAC".
(3) on page 5, "cell infected in culture" is inaccurate. It could be expressed as "cultured cells with infection".
(4) on page 13, the word "commonality" might be replaced by "Common properties/features".
(5) the last sentence on page 13 also need language editing.
(6) on page 21, the subtitle "search leader-TRS" would be "searching leader-TRS". Pls keep the subtitle to be a short phrase, rather than beginning with a verb.
(7) pls keep the references in a consistent format. Pls correct the format of Ref. 26, 29 and 30 on page 25-26.
(8) The authors just need to acknowledge the COG-UK consortia and ISARIC4C consortia, rather than list names of all members in the consortia which occupy 8 pages' space.
(9) The x or y bar label and scales in most figures/suppl figures are too small to read.
(10) The Figure legends of all figures are not clear enough and does not provide enough illustrations and explanations for the figures (e.g. Fig 1).
(11) Supplemental Fig1 could be re-designed to be more clear. For instance, the authors can merge the same steps after the step of <SAM> or <BAM>, to avoid redundant information.
(12) The legend of table 8 seems exactly same as the legend of table 2. Pls check it.

**Methods**

Are the methods appropriate to the aims of the study, are they well described, and are necessary controls included? Choose an item.

**Conclusions**

Are the conclusions adequately supported by the data shown? Choose an item.

**Reporting Standards**

Does the manuscript adhere to the journal's guidelines on minimum standards of reporting? Choose an item.

Choose an item.

**Statistics**

Are you able to assess all statistics in the manuscript, including the appropriateness of statistical tests used? Choose an item.

**Quality of Written English**

Please indicate the quality of language in the manuscript: Choose an item.

**Declaration of Competing Interests**

Please complete a declaration of competing interests, considering the following questions:

- Have you in the past five years received reimbursements, fees, funding, or salary from an organisation that may in any way gain or lose financially from the publication of this manuscript, either now or in the future?
- Do you hold any stocks or shares in an organisation that may in any way gain or lose financially from the publication of this manuscript, either now or in the future?
- Do you hold or are you currently applying for any patents relating to the content of the manuscript?
- Have you received reimbursements, fees, funding, or salary from an organization that holds or has applied for patents relating to the content of the manuscript?
- Do you have any other financial competing interests?
- Do you have any non-financial competing interests in relation to this paper?

If you can answer no to all of the above, write 'I declare that I have no competing interests' below. If your reply is yes to any, please give details below.

I declare that I have no competing interests.

I agree to the open peer review policy of the journal. I understand that my name will be included on my report to the authors and, if the manuscript is accepted for publication, my named report including any attachments I upload will be posted on the website along with the authors' responses. I agree for my report to be made available under an Open Access Creative Commons CC-BY license (http://creativecommons.org/licenses/by/4.0/). I understand that any comments which I do not wish to be included in my named report can be included as confidential comments to the editors, which will not be published.

Choose an item.

To further support our reviewers, we have joined with Publons, where you can gain additional credit to further highlight your hard work (see: https://publons.com/journal/530/gigascience). On publication of this paper, your review will be automatically added to Publons, you can then choose whether or not to claim your Publons credit. I understand this statement.

Yes Choose an item.
