# Supplementary material for: Analysis of SARS-CoV-2 known and novel subgenomic mRNAs in cell culture, animal model, and clinical samples using LeTRS, a bioinformatic tool to identify unique sequence identifiers
Source: Gigascience. 2022 May 26;11:giac045. doi: 10.1093/gigascience/giac045 (PMC9154083; doi:10.1093/gigascience/giac045)
Supplement: giac045_Reviewer_1_Report_Revision_1 — Chan Zhou -- 12/13/2021 Reviewed [file giac045_reviewer_1_report_revision_1.pdf]

Reviewer Report

Title: Analysis of SARS-CoV-2 known and novel subgenomic mRNAs in cell culture, animal model and clinical samples using LeTRS, a bioinformatic tool to identify unique sequence identifiers.

Version: Revision 1 **Date:** 12/13/2021

Reviewer name: Chan Zhou

Reviewer Comments to Author:

Comments: It is an important study. Except for a few minor points, the authors have addressed most of the reviewers' suggestions. This manuscript will be considered for acceptance after addressing the following minor suggestions:
1. The authors have compared the algorithm design, input, and output, and the counts of predicted sgmRNA across four tools. However, it would be nice if the authors could compare these tools' performances regarding prediction accuracy, F-measure, sensitivity, and specific scores. These will let the readers and potential users have a better sense of choosing a different tool for different purposes.
2. It is unclear what the red line means in Supplemental Figure 8-9.
3. On page 18, lines 364-370. The analysis and significance that the authors stated in that paragraph do not show the apparent trends in Supplemental Figure 9. Would the authors update the figure types to reflect the results of their statistical tests?
4. On page 18, line 370. The author mentioned that "The abundance of most sgmRNAs decreased with time, and both of these factors could account for the frequency of methylation." Based on the context, it seems that the conclusion could not be derived. Because the methylation frequency is a ratio, then it may not correlate with the abundance of the sgmRNAs.

Methods

Are the methods appropriate to the aims of the study, are they well described, and are necessary controls included? Choose an item.

Conclusions

Are the conclusions adequately supported by the data shown? Choose an item.

Reporting Standards

Does the manuscript adhere to the journal's guidelines on minimum standards of reporting? Choose an item.

Choose an item.

Statistics

Are you able to assess all statistics in the manuscript, including the appropriateness of statistical tests used? Choose an item.

Quality of Written English

Please indicate the quality of language in the manuscript: Choose an item.

Declaration of Competing Interests

Please complete a declaration of competing interests, considering the following questions:

- Have you in the past five years received reimbursements, fees, funding, or salary from an organisation that may in any way gain or lose financially from the publication of this manuscript, either now or in the future?
- Do you hold any stocks or shares in an organisation that may in any way gain or lose financially from the publication of this manuscript, either now or in the future?
- Do you hold or are you currently applying for any patents relating to the content of the manuscript?
- Have you received reimbursements, fees, funding, or salary from an organization that holds or has applied for patents relating to the content of the manuscript?
- Do you have any other financial competing interests?
- Do you have any non-financial competing interests in relation to this paper?

If you can answer no to all of the above, write 'I declare that I have no competing interests' below. If your reply is yes to any, please give details below.

I declare that I have no competing interests.

I agree to the open peer review policy of the journal. I understand that my name will be included on my report to the authors and, if the manuscript is accepted for publication, my named report including any attachments I upload will be posted on the website along with the authors' responses. I agree for my report to be made available under an Open Access Creative Commons CC-BY license (http://creativecommons.org/licenses/by/4.0/). I understand that any comments which I do not wish to be included in my named report can be included as confidential comments to the editors, which will not be published.

Choose an item.

To further support our reviewers, we have joined with Publons, where you can gain additional credit to further highlight your hard work (see: https://publons.com/journal/530/gigascience). On publication of this paper, your review will be automatically added to Publons, you can then choose whether or not to claim your Publons credit. I understand this statement.

Yes Choose an item.
