# Supplementary material for: Analysis of SARS-CoV-2 known and novel subgenomic mRNAs in cell culture, animal model, and clinical samples using LeTRS, a bioinformatic tool to identify unique sequence identifiers
Source: Gigascience. 2022 May 26;11:giac045. doi: 10.1093/gigascience/giac045 (PMC9154083; doi:10.1093/gigascience/giac045)
Supplement: giac045_Reviewer_2_Report_Original_Submission — Lachlan Coin -- 6/23/2021 Reviewed [file giac045_reviewer_2_report_original_submission.pdf]

Reviewer Report

Title: Analysis of SARS-CoV-2 known and novel subgenomic mRNAs in cell culture, animal model and clinical samples using LeTRS, a bioinformatic tool to identify unique sequence identifiers.

Version: Original Submission Date: 6/23/2021

Reviewer name: Lachlan Coin

Reviewer Comments to Author:

"Identification and quantification of SARS-CoV-2 leader subgenomic mRNA gene junctions in nasopharyngeal samples shows phasic transcription in animal models of COVID-19 and dysregulation at later time points that can also be identified in humans"

In this paper, Dong et al describe a new pipeline for identifying subgenomic mRNA from multiple types of sequence data, including amplicon (Illumina and Nanopore) as well as long read nanopore direct RNA or cDNA sequencing. It is useful to have a bioinformatics pipeline which can rapidly identify sgRNA in multiple types of sequence data and has the potential to open large amplicon datasets in particular for further analysis of sgRNA abundance. However, I believe that more validation of the accuracy of abundance estimates from amplicon data is required in order to give the research community more confidence in its use (and limitations).

Major comments:

1. More explanation/detail on methodology would be useful. The authors say that they find the most common peak for the break points of the disjunction site amongst all reads with a break point within a 20bp window of the expected breakpoint. Is there a threshold applied in terms of the difference between the most common and next-most-common breakpoint? Also for the novel sites, is there a clustering algorithm applied, or any site with more than 10 reads is reported?

2. I would like a more direct comparison of sgRNA abundances estimated from amplicon based approach, vs using nanopore amplicon free approach? Its possible to do this only by comparing different tables. It would be easier to digest if there was a x-y plot comparing abundances from different approaches on the same sample. This would help give confidence that the amplicon based approach can provide good estimates. From looking at the tables 1 and 2, it seems that the amplicon approach estimates a lot less sgRNA than the amplicon free approach overall (in terms of normalized counts per million mapped reads). This is to be expected as most of the reads from the amplicon sequencing would be expected to come from the genome. It would be good to see which ORFs are under- and over-represented in the amplicon data, as I imagine this would also relate to which primer pairs are in the same amplicon pool in the arctic design.

Related to this, it would be good to have an analysis of how the primer design impacts detection of sgRNA. For example, I thought that only one of the primer pools includes a leader primer.

Further related to this, it would be good to have a plot which shows the proportion of read counts which are derived from left-primer only, right-primer only or both primers for each sgRNA, and how this compares to the overall ratio of left-only and right-only primers. It seemed odd to me at first glance that there are so many one-sided amplifications, but I imagine this is a small proportion overall, but a

sizeable proportion of the reads which can identify sgRNA, due to the lack of primer pairs for many of the sgRNA. Based on this analysis, it would also be interesting to estimate what is the best depth of coverage of the amplicon panels to get reliable estimates of sgRNA abundance across the different ORFs.

3. It would be good to compare the novel breakpoints with those previously reported, e.g. in Taiorara et al, figure 2 and supplementary figure 6 (https://doi.org/10.1101/2020.03.05.976167). I can see that many of them line up with those you report in table 4, and I believe this sup

4. Is there much overlap in the novel break points detected using nanopore amplicon ARTIC v3 vs nanopore dRNA? It would be good to have an extra column in Table 8 and table 4 indicating which of the breakpoints discovered in dRNA were also discovered in amplicon sequencing and vice versa. This will hopefully shed light on relative strengths of the two approaches. Similarly it would be useful to compare nanopore ARTIC and illumina ARTIC in this regard

5. Its hard to assess the evidence supporing the biphasic expression without having some idea of the error in the abundance estimates (also commented on this more below);

6. The conclusion of dysregulation in samples taken from patients many days into their infection is made only on a small number of samples. Also in Figure 4, the time post sample is not indicated. I presume the information is in one of the supplementary tables, but the submitted pdf has messed up these tables (its somewhere in the 729 page pdf) . Nevertheless, it seems that the data supporting this conclusion is a bit thin, and I would be cautious in including that observation in the title of the paper.

Minor comments:

1. In figure legends (e.g. figure 1) you say the numbers in brackets are:reads with left primers, reads with right primers, reads with both primers. I can see from the numbers that these are not exclusive, but it might be easier to digest if you showed left-only, right-only and both

2. You make a point in the paper about whether the left break occurs at position 64 or 69. One thing I would worry about is that microhomology between TRS-L and TRS-R might make it difficult to be exactly sure of the breakpoint (because the sgRNA includes only one copy of the TRS, but its hard to know if it's the left or the right which is included, the aligner could equally well align to TRS-L and skip TRS-R or vice versa, and this would shift the coordinates slightly. Are the enough snp differences in TRS-L or TRS-R to be confident either way, and if so, does this have implications for whether TRS-L or TRS-R is retained in the sgRNA?

3. Figure 1 panels B,C,D were a bit confusing. Why is the reference sequence in the middle. It would be good if the caption could be expaned to help the reader understand these panels in particular.

4. The tables (table 1 to 8) and the figure 1A represent a lot of the same information, but the numbers don't line up exactly, because in the figures you only use counts which have both primers. It would be best to decide which to represent because it's confusing to have the same data presented twice essentially but in slightly different ways.

5. In figure 1 you present the normalized abundance to 2 decimal places, but its very unlikely that you have that level of precision. It would be good if you could add error bars to estimate the uncertainty in the abundance estimate (e.g. calculated using a binomial distribution).

6. In figure 3, its hard to know how much error there is in each of the measurements. By showing the normalized value, its also hard to see what is the absolute change in the read counts. Ideally you would show either the read counts, or show error bars around the abundance estimates.

7. Is there a mistake in the title of Table 8: "The LeTRS output table for novel sgmRNA in the tested Nanopore ARTIC v3 primers." Because the title of table 2 seems the same: Table 2. The LeTRS output table for novel sgmRNA in the tested Nanopore ARTIC v3 primers" . One of these approaches does not seem to find novel breakpoints, but the other does, presumably Table 8 should be illumina based on the ordering?
8. Error in caption of table 1: " Normalized count=(Read count-Total number of read mapped on reference genome)*1000000"
9. In the supplementary figures, the captions you saay:" Supplementary Figure 3. Raw (A and C) and normalised (B and D) expected (upper) and novel (lower) leader-TRS gene junctions count in the infecting SARS-CoV-2 inoculum source used for NHP study, sequenced by Illumina ARTIC method (Supplementary Table 8)."
I found the use of "expected" here confusing, because it implied to me that you had estimated expected counts. I would prefer the use of the term canonical, or something like that.

Methods

Are the methods appropriate to the aims of the study, are they well described, and are necessary controls included? Choose an item.

Conclusions

Are the conclusions adequately supported by the data shown? Choose an item.

Reporting Standards

Does the manuscript adhere to the journal's guidelines on [minimum standards of reporting?]() Choose an item.

Choose an item.

Statistics

Are you able to assess all statistics in the manuscript, including the appropriateness of statistical tests used? Choose an item.

Quality of Written English

Please indicate the quality of language in the manuscript: Choose an item.

Declaration of Competing Interests

Please complete a declaration of competing interests, considering the following questions:

- Have you in the past five years received reimbursements, fees, funding, or salary from an organisation that may in any way gain or lose financially from the publication of this manuscript, either now or in the future?
- Do you hold any stocks or shares in an organisation that may in any way gain or lose financially from the publication of this manuscript, either now or in the future?
- Do you hold or are you currently applying for any patents relating to the content of the manuscript?
- Have you received reimbursements, fees, funding, or salary from an organization that holds or has applied for patents relating to the content of the manuscript?
- Do you have any other financial competing interests?
- Do you have any non-financial competing interests in relation to this paper?

If you can answer no to all of the above, write 'I declare that I have no competing interests' below. If your reply is yes to any, please give details below.

No

I agree to the open peer review policy of the journal. I understand that my name will be included on my report to the authors and, if the manuscript is accepted for publication, my named report including any attachments I upload will be posted on the website along with the authors' responses. I agree for my report to be made available under an Open Access Creative Commons CC-BY license (http://creativecommons.org/licenses/by/4.0/). I understand that any comments which I do not wish to be included in my named report can be included as confidential comments to the editors, which will not be published.

Choose an item.

To further support our reviewers, we have joined with Publons, where you can gain additional credit to further highlight your hard work (see: https://publons.com/journal/530/gigascience). On publication of this paper, your review will be automatically added to Publons, you can then choose whether or not to claim your Publons credit. I understand this statement.

Yes Choose an item.
